# Microbial Community Structure of Relict Niter-Beds Previously Used for Saltpeter Production

**DOI:** 10.1371/journal.pone.0104752

**Published:** 2014-08-11

**Authors:** Takashi Narihiro, Hideyuki Tamaki, Aya Akiba, Kazuto Takasaki, Koichiro Nakano, Yoichi Kamagata, Satoshi Hanada, Taizo Maji

**Affiliations:** 1 Bioproduction Research Institute, National Institute of Advanced Industrial Science and Technology (AIST), Tsukuba, Ibaraki, Japan; 2 FASMAC Co., Ltd., Atsugi, Kanagawa, Japan; 3 Bioproduction Research Institute, National Institute of Advanced Industrial Science and Technology (AIST), Sapporo, Hokkaido, Japan; 4 Shubun University, Ichinomiya, Aichi, Japan; U. S. Salinity Lab, United States of America

## Abstract

From the 16th to the 18th centuries in Japan, saltpeter was produced using a biological niter-bed process and was formed under the floor of gassho-style houses in the historic villages of Shirakawa-go and Gokayama, which are classified as United Nations Educational, Scientific and Cultural Organization (UNESCO) World Heritage Sites. The relict niter-beds are now conserved in the underfloor space of gassho-style houses, where they are isolated from destabilizing environmental factors and retain the ability to produce nitrate. However, little is known about the nitrifying microbes in such relict niter-bed ecosystems. In this study, the microbial community structures within nine relict niter-bed soils were investigated using 454 pyrotag analysis targeting the 16S rRNA gene and the bacterial and archaeal ammonia monooxygenase gene (*amoA*). The 16S rRNA gene pyrotag analysis showed that members of the phyla *Proteobacteria*, *Actinobacteria*, *Bacteroidetes, Chloroflexi, Firmicutes*, *Gemmatimonadetes*, and *Planctomycetes* were major microbial constituents, and principal coordinate analysis showed that the NO_3_^−^, Cl^−^, K^+^, and Na^+^ contents were potential determinants of the structures of entire microbial communities in relict niter-bed soils. The bacterial and archaeal *amoA* libraries indicated that members of the *Nitrosospira*-type ammonia-oxidizing bacteria (AOB) and “*Ca*. Nitrososphaera”-type ammonia-oxidizing archaea (AOA), respectively, predominated in relict niter-bed soils. In addition, soil pH and organic carbon content were important factors for the ecological niche of AOB and AOA in relict niter-bed soil ecosystems.

## Introduction

Until the early 20th century, saltpeter (i.e., niter or potassium nitrate [KNO_3_]), which is known as the main source of gunpowder, was produced via an artificial bioprocess, the so-called “niter-bed” [1], [2]. The basic concept of saltpeter production via niter-bed involves the nitrification of ammonia-containing wastes (e.g., plant residue, horse manure, and human urine) mixed with a certain amount of soil [1], [2]. This nitrification reaction was thought to be performed by ammonia-oxidizing and nitrite-oxidizing microbes [1], [2]. The first description of a niter-bed was found in the military technological manual “Bellifortis,” which was written by Konrad Kyeser and published in 1405. This manual contains brief instructions describing how to construct a niter-bed [3], [4], and this process was employed for saltpeter production in European countries [1],[5],[6], India [7], [8], and Japan [9], [10] until the discovery of a large natural deposit in Chile [11]. In Finland, the export of saltpeter produced by niter-bed reached a maximum level (433 tons) in 1816 during the last stages of the Napoleonic wars [6]. In India, a large amount of saltpeter was produced annually (20,000 tons/year) using the niter-bed methodology in the early 20th century [7]. Thus, the niter-bed process is a historically important industry in several nations.

From the 16th to the 18th centuries in Japan, saltpeter was produced for military purposes using the niter-bed formed under the floor of gassho-style houses [10], which is a specific architectural style used in the historic villages of Shirakawa-go and Gokayama, which are listed as UNESCO World Heritage Sites [12]. Because these villages were located in the mountainous area of the former Kaga domain in Japan (currently the Toyama and Ishikawa prefectures), there was limited land for the production of agricultural crops. Hence, people in this area faced economic difficulties. To overcome this issue, the people practiced sericulture to produce silk as an alternative income source in the loft spaces of gassho-style houses [10]. Moreover, according to the historical records, the people produced saltpeter in the underfloor space of gassho-style houses using ammonia-containing wastes such as plant residues (e.g., the stems of Japanese millet, buckwheat, and tobacco), silkworm feces from sericulture, and human urine as primary substrates [10]. These substrates were mixed with soil under the floor of the gassho-style house to create a niter-bed, and nitrate (NO_3_) was produced via a microbial nitrification reaction. The resulting nitrate contained in the niter-bed soil was further extracted with water and purified by adding wood ash to remove unnecessary minerals (e.g., Ca, Mg, K, and Na). Purified saltpeter was used as the raw material for gunpowder production, and this gunpowder also became an important source of income for people who lived in this area [9], [10]. Thus, saltpeter production via the niter-bed process was a cornerstone of the lifestyle in this area.

Although saltpeter production in the villages of Shirakawa-go and Gokayama ended more than 100 years ago, the relict niter-beds are now conserved in the underfloor space of gassho-style houses, where they are isolated from rainfall and other destabilizing factors [10]. Surprisingly, bulk soils collected from relict niter-beds in Shirakawa-go village retained nitrification activity [13]. This finding implies that nitrifying microbes have persisted in the relict niter-bed soils; however, there is no information available regarding the microbial community structure responsible for nitrification in the relict niter-bed ecosystem. In this study, we investigated the microbial community structure of nine soil samples obtained from the underfloor areas of three gassho-style houses by using 454 pyrotag analysis targeting the 16S rRNA gene and the ammonium monooxygenase subunit A gene (*amoA*) of ammonia-oxidizing bacteria (AOB) and ammonia-oxidizing archaea (AOA). In addition, a previously reported *amoA* pyrotag dataset [14] was used to elucidate the ecological niche of AOB and AOA in soil environments.

## Materials and Methods

### Soil sample

A total of nine soil samples were collected from three relict niter-beds under the floors of gassho-style houses located in Shirakawa Village, referred to as OVA, OVB, and OVC. We collected five, two, and two soil samples from the OVA, OVB, and OVC houses; these samples are referred to as OVA1–5, OVB2 and OVB3, and OVC1 and OVC2, respectively. Each 500–1,000 g of soil sample was collected from relict niter-beds (for OVA and OVC, a depth of ca. 10 cm from the surface; for OVB, a depth of ca. 20 cm from the surface) by permission from the education board of Shirakawa-Village, Gifu, Japan. Detailed physicochemical parameters, including pH, organic carbon content, and ion contents, are summarized in Table S1 [13], [15]–[17]. Pebbles were removed from the samples using a 30-mesh filter, and the samples were subjected to DNA extraction.

### DNA extraction, PCR, and pyrosequencing

DNA was extracted from the soil samples using the FastDNA SPIN Kit for Soil (MP Biomedicals, Carlsbad, CA, USA) according to the manufacturer’s instructions. Approximately 50 ng of template DNA was used for PCR amplification in a reaction volume of 50 µL. The 16S rRNA, bacterial *amoA*, and archaeal *amoA* genes were amplified with the forward/reverse primer sets Univ519F/Univ926R [18], amoA-1F/amoA-2R [19], and Arch-amoAF/Arch-amoAR [20], respectively. The forward and reverse primers were barcoded with fusion A (FA; 5′-CGTATCGCCTCCCTCGCGCCATCAG-3′) and fusion B (FB; 5′-CTATGCGCCTTGCCAGCCCGCTCAG-3′) adaptor sequences, respectively. The primers also included Multiplex Identifier (MID) sequences. Detailed information regarding the primer sequences is provided in Table S2. These genes were amplified with PrimeSTAR HS DNA Polymerase (TaKaRa, Otsu, Japan) using the following thermocycling conditions: the appropriate number of cycles (25 cycles for 16S rRNA, 40 cycles for bacterial *amoA*, and 35 cycles for archaeal *amoA* genes) of denaturation at 98°C for 10 sec, annealing at 55°C for 15 sec, and extension at 72°C for 1 min. PCR amplicons were purified using a Wizard SV Gel and PCR Clean-Up System (Promega, Fitchburg, WI, USA) and pooled for subsequent 454 pyrotag analysis. Pyrosequencing was performed using the GS-FLX Titanium platform (Roche/454 Life Sciences, Branford, CT, USA) at the FASMAC Co., Ltd. (Atugi, Japan). Sequencing was performed using 1/2-region gaskets. For the 16S rRNA gene, we used pyrotag reads obtained from both fusion sides (A and B) because the 454 platform used in this study generated full-length amplicons (approximately 370 bp). For bacterial and archaeal *amoA* genes, we used pyrotag reads obtained from only the fusion A side because the length of each sequenced read did not cover the entire region of the amplicon.

### Operational taxonomic unit (OTU) analysis for 16S rRNA gene pyrotags

Raw 16S rRNA gene pyrotag sequences were qualitatively screened and trimmed with QIIME 1.7.0 [21], maintaining the sequence length at ≥300 nt. The sequences had an average quality score of ≥25. A denoising function was applied to the filtered sequence data [22]. The denoised sequence data were grouped into OTUs with the UCLUST algorithm [23]. The sequences exhibiting >97% identities were grouped into one OTU. The representative sequences of each OTU were aligned with PyNAST [24], and chimeric sequences were detected and removed using ChimeraSlayer [25]. The taxonomic assignment of each OTU was performed with the RDP classifier trained by a dataset from Greengenes at a minimum confidence level of 0.8 [26]. The sequence data were compared with the 16S rRNA database of RDP release 10 [27] using the BLAST+ program [28].

### OTU analysis for *amoA* pyrotags

The procedures for quality screening, trimming, denoising, and OTU selection were same as those described above for 16S rRNA gene pyrotag analysis, except that sequence identity (>85%) was determined according to the threshold of Pester *et al*. [14]. The filtered sequences were manually checked after aligning with MEGA program [29]. Chimeric sequences were detected and removed using the UCHIME algorithm [30] on the FunGene platform [27]. Sequence data were compared with the GenBank database using the BLAST+ program [28].

### Statistical and phylogenetic analysis

After taxonomy assignments, α-diversity indices (e.g., Chao1, Shannon, and Simpson) of the 16S rRNA and *amoA* genes pyrotag libraries were calculated by QIIME [21] and EstimateS (version 9.1.0) [31] software, respectively. The coverage values were calculated using equation [1– (*n/N*)], where *n* is the number of OTUs in a single read (singleton) and *N* is the total number of reads analyzed [32]. The weighted UniFrac distances were calculated and used for principal coordinate analysis (PCoA) [33]. Phylogenetic trees for the bacterial and archaeal *amoA* libraries for Fast UniFrac analysis were constructed with the MEGA program [29] based on the neighbor-joining algorithm [34]. PCoA and jackknife clustering analyses were performed on weighted and normalized data with QIIME and the Fast UniFrac program [33], respectively. To identify OTUs that exhibited significant differences in abundance between different soil samples, Welch’s *t*-test (confidence interval method: Welch’s inverted, [*p*<0.05] for two groups [definition of each group was described in the Results and Discussion]) having possibly unequal variances was performed with STAMP software [35], [36].

The distance matrix tree of the bacterial and archaeal *amoA* genes, which is based on the maximum likelihood method, was constructed with the MEGA program [29]. The topology of the trees was estimated by 1,000 bootstrap replicates [37]. Previously reported *amoA* pyrotag sequences retrieved from soil environments in Austria, Costa Rica, Greenland, and Namibia were downloaded from the Sequence Read Archive (SRA) at NCBI (accession no. SRA047303) and used for the statistical and phylogenetic comparisons [14]. Subclusters within the “*Ca.* Nitrososphaera” and “*Ca*. Nitrosopumilus” were defined according to Pester *et al*. [14].

### Nucleotide sequence accession numbers

The pyrosequence data obtained in this study have been deposited under DDBJ/EMBL/GenBank accession no. DRA001202.

## Results and Discussion

### 16S rRNA gene pyrotag analysis

In this study, nine soil samples from three gassho-style houses, referred to as OVA (samples OVA1–5), OVB (samples OVB2 and OVB3), and OVC (samples OVC1 and OVC2), were used for microbial community analysis using 454 pyrosequencing. We retrieved a total of 173,016 16S rRNA gene pyrotags and found 3,146 OTUs with the criterion of >97% sequence identity (Table 1). Although the rarefaction curves showed that the accumulation of the 16S rRNA gene OTUs was insufficient to achieve the goal of saturation (Fig. S1A), the coverage values were calculated to be 96.99–98.59%, suggesting that the OTUs retrieved here were sufficient for estimating the microbial diversity in the relict niter-bed ecosystems. According to the Chao1 nonparametric estimators, the soil samples contained approximately 1.38–1.63-fold more OTUs than what was detected in our analyses.

**Table 1:** Pyrosequencing results of 16S rRNA, bacterial *amoA*, and archaeal *amoA* genes and observed diversity indexes.

SampleID	16SrRNA						Bacterial*amoA*						Archaeal*amoA*					
	Total reads	OTU	Chao 1	Shannon	Simpson	Coverage (%)	Total reads	OTU	Chao 1	Shannon	Simpson	Coverage (%)	Total reads	OTU	Chao 1	Shannon	Simpson	Coverage (%)
OVA1	7,770	514	757.8	4.57	37.42	97.52	N.D.^a^						4,240	6	7.00	1.16	1.06	99.95
OVA2	17,480	641	1,042.8	4.63	47.06	98.59	N.D.^a^						3,455	9	10.00	1.70	1.34	99.91
OVA3	16,615	990	1,485.7	4.92	33.83	97.80	7,826	6	6.00	0.07	1.02	99.99	3,872	6	7.00	2.18	1.83	99.95
OVA4	19,266	1,496	2,248.9	5.60	84.90	96.99	10,197	5	5.00	0.15	1.07	99.99	2,705	9	10.50	2.37	1.87	99.89
OVA5	18,029	1,360	2,124.7	5.50	77.00	97.06	14,558	6	6.00	0.22	1.10	99.99	3,670	8	8.33	2.02	1.72	99.95
OVB2	19,304	1,020	1,411.7	5.06	44.17	98.23	4,606	6	6.00	0.32	1.19	99.98	2,957	4	4.00	2.02	1.96	100.00
OVB3	22,764	1,028	1,538.0	4.99	36.75	98.42	6,896	9	10.50	0.32	1.17	99.96	3,695	8	8.33	2.13	1.94	99.95
OVC1	23,172	1,144	1,816.1	4.56	17.29	98.09	8,016	3	3.00	0.40	1.23	100.00	3,550	10	10.00	2.13	1.55	99.97
OVC2	28,616	1,238	1,824.5	4.66	20.59	98.42	7,305	5	5.00	0.75	1.89	100.00	4,943	12	18.00	1.85	1.48	99.92
Total	173,016	3,146					59,404	13					33,087	16				

a N.D., not detected.

The OTUs belonging to the phyla *Proteobacteria* (especially the classes *Alphaproteobacteria* and *Gammaproteobacteria*), *Actinobacteria*, *Bacteroidetes*, *Chloroflexi*, *Firmicutes*, *Gemmatimonadetes*, and *Planctomycetes* predominated and were detected in all samples (Table S3 and S4). Such microbial taxa are frequently found in soil environments [38]. In each sample, only a few number of the 16S rRNA gene pyrotags (<0.8% of the total populations) were associated with previously known ammonia-oxidizing microorganisms at the genus level: for AOB, three OTUs (nos. 1267, 2435, and 2701) of the *Nitrosococcus*, one OTU (no. 2080) of the *Nitrosomonas*, and one OTU (no. 673) of the *Nitrosospira*; for AOA, four OTUs (nos. 1207, 1897, 1946, and 3119) of the “*Ca*. Nitrososphaera” (Table S5). Given that soil samples from the relict niter-beds have retained microbial nitrification activity [13], these nitrifying microbes may contribute to the nitrification at low population densities; alternatively, there may have been unidentified nitrifying microbes in the relict niter-bed soils. Although the relative abundances of nitrifying microbes were in good agreement with the microbial compositions of several types of soil ecosystems [38], we could not fully elucidate the biodiversity and ecological niche of ammonia-oxidizing microbes in the relict niter-bed ecosystems based on the results of 16S rRNA gene pyrotag analysis (e.g., there are no significant relationship between the ratio of the abundance ratio of AOB to AOA and clustering of the communities in PCoA and jackknife analysis described below) (Table S5). These observations emphasize the need to implement further analysis targeting *amoA* gene that enable specific detection of ammonia-oxidizing microbes.

**Figure 1:**
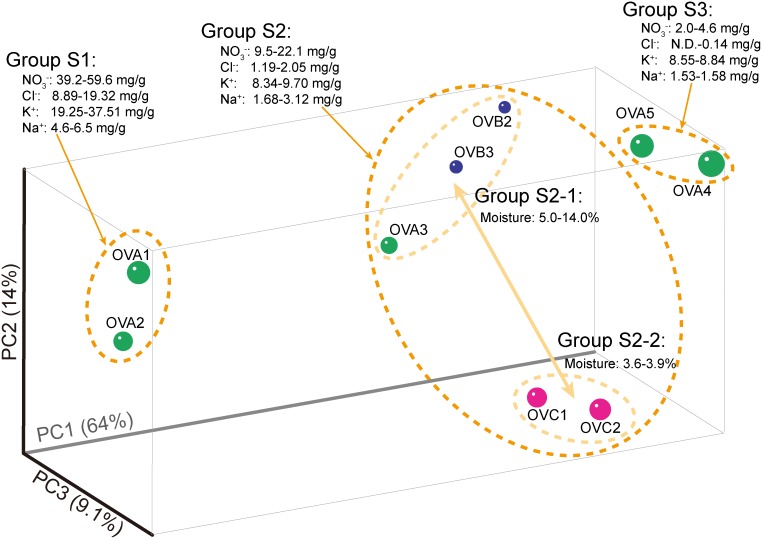
Principal coordinate analysis based on the abundances of 16S rRNA gene OTUs (weighted UniFrac) and separating soils according to their ion contents. For this analysis, observed 16S rRNA gene OTUs were normalized to 7,770 reads per soil.

The similarity of microbial compositions among the nine soil samples was compared on the basis of 16S rRNA gene pyrotag sequences. According to the PCoA and jackknife analyses, the microbial community structures of the relict niter-bed soil were classified into three groups (Fig. 1 and S2A). Group S1, consisting of OVA1 and OVA2, was distinct from the other two groups. Based on previously reported physicochemical characterization [15]–[17], relatively high contents of NO_3_^−^, Cl^−^, K^+^, and Na^+^ were found in OVA1 and OVA2 compared with the samples clustered into groups S2 and S3 (Table S1). Because the soils in group S1 were not subjected to the extraction step of saltpeter production, the ion contents were higher than those in the other relict niter-bed soil samples [17]. In addition, the NO_3_^−^ and Cl^−^ contents were relatively low in the soils clustered in group S3 compared with those in group S2. Based on Welch’s *t*-test, we found a total of 21 OTUs that were significantly different in relative abundances among the groups and were associated with dominant taxa (e.g., *Alphaproteobacteria*, *Gammaproteobacteria*, *Actinobacteria*, *Bacteroidetes*, *Chloroflexi, Firmicutes*, and *Gemmatimonadetes*) (Fig. S3). Among them, *Gammaproteobacteria*-type OTU (NB_16S_507) and *Chloroflexi*-type OTU (NB_16S_1415) were involved in the differentiation of groups S1, S2, and S3. The samples in group S2 were further separated according to their moisture contents (groups S2-1 and S2-2), and the relative abundances of six OTUs (NB_16S_1093, 1144, 1409, 1415, 2046, and 2058) were significantly affected by the moisture contents (Fig. S3). Several types of ion species and moisture contents have been recognized as potential factors in the determination of soil microbial constituents [39]–[42]. Thus, the NO_3_^−^, Cl^−^, K^+^, Na^+^, and moisture contents may affect the microbial community structures of relict niter-bed ecosystems.

### Bacterial *amoA* gene pyrotag analysis

As described above, the proportions of 16S rRNA gene pyrotags associated with AOB and AOA were very low in the relict niter-bed soils. To further elucidate the microbial constituents responsible for ammonia oxidation, we performed pyrotag analysis targeting bacterial and archaeal *amoA* genes. As a result, we obtained a total of 59,404 bacterial *amoA* pyrotags and found 13 OTUs in relict niter-bed soils (Table 1). However, the bacterial *amoA* gene fragment could not be amplified from OVA1 and OVA2 using the primer set described in this study, suggesting that there were either low levels of AOB in the samples or there were some AOB with significantly different types of *amoA* genes. Instead, the archaeal *amoA* gene fragments were successfully amplified from OVA1 and OVA2 samples, suggesting that AOA may be a major player in ammonia oxidation in these soils (details described below). Although the rarefaction curves for the bacterial *amoA* libraries shorted to reach a plateau (except for OVC1 and OVC2), the coverage values were calculated to be 99.96–100%, indicating that the OTUs retrieved here were sufficient for estimating the biodiversity of AOB in seven relict niter-bed soils (Table 1 and Fig. S1B).

**Figure 2:**
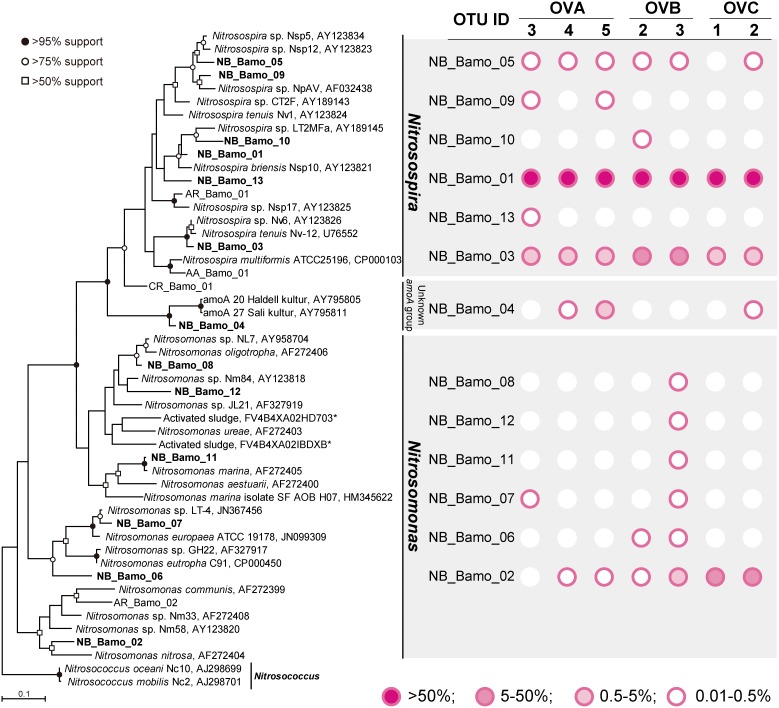
Distance matrix tree of bacterial *amoA* gene sequences retrieved from niter-bed soils based on the maximum likelihood method. Boldface indicates the sequences obtained in this study. The *Nitrosococcus amoA* gene sequences (AJ298699 and AJ298701) were used as outgroups. The bar indicates 10% base substitution. Branching points supported probabilities >95%, >75%, and >50% by bootstrap analyses (based on 1,000 replicates, estimated using the maximum likelihood method) are indicated by solid circle, open circles, and open square, respectively.

The most abundant AOB belonged to the genus *Nitrosospira* (6 OTUs, 68.13–99.96% of the total reads in each sample), and *Nitrosospira briensis*-type OTU (NB_Bamo_01) predominated in all samples (except for OVA1 and OVA2, in which there were no bacterial *amoA* gene amplification) (Table S6). This result is in good accordance with 16S rRNA gene pyrotag analysis that found a *Nitrosospira briensis*-type OTU (no. 673) across the samples. An OTU (NB_Bamo_01) related to the *Nitrosospira briensis* strain Nsp10 (95.5% *amoA* gene sequence identity) was detected as a major AOB population in all niter-bed soils (Fig. 2 and Table S6). *Nitrosospira briensis* are enriched in and have been isolated from soil samples taken from sandy and agriculturally uncultivated areas of Europe [43]. To the best of our knowledge, only two reports have described the clear appearance of *Nitrosospira briensis* populations: water columns of the Chesapeake Bay [44] and soil samples of the Cascade Mountains in Oregon [45] in the U.S. Interestingly, Bollmann *et al*. [46] reported that under ammonia-starved conditions, the cells of *Nitrosospira briensis* had the ability to immediately recover ammonia oxidation activity upon exposure to ammonia-rich conditions. Although the mechanism behind this phenomenon is unclear, *Nitrosospira briensis*-type AOB may reserve their activity in ammonia-limited environments, such as the relict niter-bed soils. Within the genus *Nitrosospira*, an OTU (NB_Bamo_03) that is closely related to the *Nitrosospira tenuis* strain Nv-12 (96.2% sequence identity) was also detected in all bacterial *amoA* gene pytotag libraries; however, its ecophysiological traits (other than ammonia oxidation) remain unclear.

In addition to the *Nitrosospira* populations, six *Nitrosomonas*-type OTUs were found in seven niter-bed soils (Fig. 2 and Table S6). In contrast to the *Nitrosospira*-type OTUs, 16S pyrotags related to *Nitrosomonas* were found in OVA3 and OVA4; however, the proportions were quite low (0.005–0.006% of total 16S rRNA gene pyrotags). This finding may also support the utility of *amoA*-based pyrotag analysis for relict niter-bed soils. An OTU (NB_Bamo_02) associated with *Nitrosomonas nitrosa* (87.2% sequence identity) was observed with significant abundance in OVC1 and OVC2 (5.6% and 31.8%, respectively) (Table S6). According to previous reports, *Nitrosomonas nitrosa*-type AOB have frequently been found in estuary soils [47], nitrification bioreactors [48], [49], and freshwater ecosystems [50]. In addition to these taxa, an OTU (NB_Bamo_04) related to the unidentified *amoA* gene cluster consisting of clones retrieved from potash-polluted marsh soil [51] was found in three relict niter-bed soils (Fig. 2 and Table S6). This cluster has a significantly low identity with previously reported bacterial *amoA* gene sequences (e.g., NB_BamoA_04 had 77.4% identity with *Nitrosospira multiformis* strain C-71, accession no. X90822), suggesting that phylogenetically novel AOB may play a role in ammonia oxidation in some niter-bed ecosystems.

**Figure 3:**
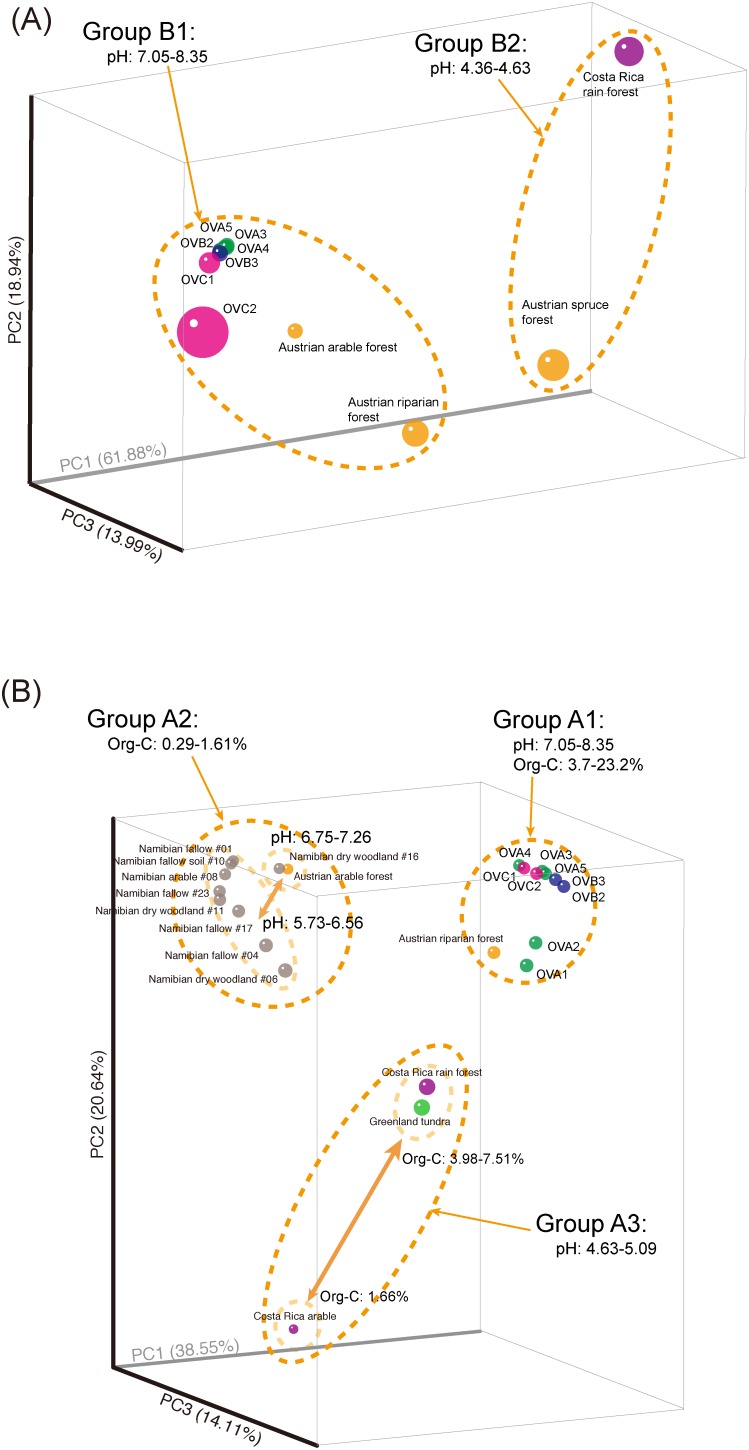
Principal coordinate analysis based on the abundances of (A) bacterial and (B) archaeal *amoA* gene OTUs (weighted UniFrac) and separating soils according to soil pH and organic carbon contents. For this analysis, *amoA* pyrotag libraries reported by Pester et al. [14] were used for the comparison, and bacterial and archaeal *amoA* OTUs observed here were normalized to 2,320 and 2,130 reads per soil, respectively.

The similarity of AOB compositions in relict niter-bed soils was compared with previously reported *amoA* gene-based microbial community analysis [14]. PCoA and jackknife analyses showed that soil bacterial *amoA* libraries were separated into two groups according to their pH: seven niter-bed soils were clustered into group B1 with two Austrian arable and riparian forest soils (pH 7.05–8.35), and the remaining two soils, from an Austrian spruce forest and a Costa-Rican rain forest, were clustered into group B2 (pH 4.36–4.63) (Fig. 3A). pH is generally to be a key parameter in defining the abundance of AOB populations in soil ecosystems [52], [53] and bioreactors [54], [55]. The niter-bed soils in group B1 were further divided into two subgroups (Fig. S2B and S3A); however, there was no clear explanation for the differentiation between OVC2 and the other soils based on the physicochemical parameters analyzed herein. In short, soil pH is one of the important factors affecting the AOB populations in relict niter-bed ecosystems.

### Archaeal *amoA* gene pyrotag analysis

A total of 33,087 archaeal *amoA* gene sequences were retrieved from nine relict niter-bed soils, and we found 16 OTUs with the criterion of >85% sequence identity. The archaeal *amoA* diversity was almost fully covered according to the rarefaction curves, coverage values, and Chao1 diversity index (Table 1 and Fig. S1C).

**Figure 4:**
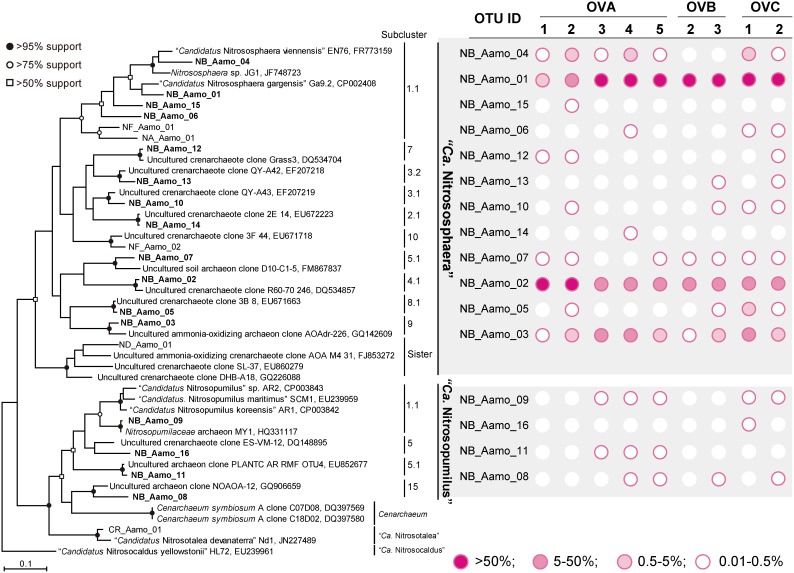
Distance matrix tree of archaeal *amoA* gene sequences retrieved from niter-bed soils based on the maximum likelihood method. Boldface indicates the sequences obtained in this study. The *amoA* gene sequence of the “*Ca*. Nitrosocaldus yellowstonii” strain HL72 (EU239961) was used as outgroup. The bar indicates 10% base substitution. Branching points supported probabilities >95%, >75%, and >50% by bootstrap analyses (based on 1,000 replicates, estimated using the maximum likelihood method) are indicated by solid circle, open circles, and open square, respectively.

Members of the genus “*Ca*. Nitrososphaera” were detected as major AOA populations (12 OTUs, 99.23–100% of the total reads in each sample), whereas those of “*Ca*. Nitrosopumilus” were absent or few (<0.28% of the total reads in each sample) in all samples tested (Fig. 4 and Table S6). Based on the 16S rRNA gene pyrotag analysis, only a few OTUs (accounting for 0.16–1.1% of the total prokaryotic population) associated with “*Ca*. Nitrososphaera” were identified as AOA populations in relict niter-bed soils (Table S5). Thus, the results from *amoA*-based pyrotag analysis expanded the biodiversity of AOA in relict niter-bed soils. The most abundant OTU in OVA1 and OVA2 soils was NB_Aamo_02, which was categorized into subcluster 4.1 of “*Ca.* Nitrososphaera” and was distantly related to previously characterized AOA (77.8% sequence identity with “*Ca.* Nitrososphaera viennensis” strain EN76, accession no. FR773159), whereas the most abundant OTU in the other seven samples (NB_Aamo_01) was closely related to “*Ca.* Nitrososphaera gargensis” strain Ga9.2 (90.6% sequence identity, accession no. CP002408) and categorized into subcluster 1.1. This marked difference in a major AOA population within OVA soils was clearly associated with the maximum nitrification rate of bulk soil (Table S1) [13]. This finding also implies that the nitrification activity and/or affinity for ammonia may differ between subclusters 1.1 and 1.4. The *amoA* fragments associated with “*Ca.* Nitrososphaera” subcluster 1.4 have been observed in diverse environments, including soil [20], [56], [57], salt marsh sediment [58], and marine sediment [20], [59]. In addition to the major AOA, several subclusters of “*Ca*. Nitrososphaera” were detected as minor AOA populations. For example, an OTU (NB_Aamo_03) assigned to subcluster 9 was commonly observed in the relict niter-bed soils (Fig. 4). Although information regarding the ecological traits of “*Ca*. Nitrososphaera” subcluster 9 organisms is limited, these organisms were widely distributed in natural environments, such as marine sediment [60] and soil [20]. Further studies using metagenomic and single-cell genomic approaches are necessary to elucidate the ecological functions of unidentified AOA in relict niter-bed ecosystems.

PCoA and jackknife analyses clearly showed that archaeal *amoA* pyrotag libraries were separated into three clusters according to soil pH and organic carbon concentration (Fig. 3B and S2C). The niter-bed soils formed group A1 with an Austrian riparian forest soil. Group A1 soils had a weak alkaline pH (7.05–8.35) and relatively high organic carbon content (3.7–23.2%). In addition, relict niter-bed soils were further separated into two subgroups according to their NO_3_^−^, Cl^−^, K^+^, and Na^+^ contents in addition to the 16S rRNA gene-based comparison (Fig. S4B). The samples clustered into group A2 were mainly composed of Namibian soils, which had relatively low organic carbon contents (0.29–1.61%) and a weakly acidic pH (5.7–6.8). The samples categorized into group A3 (soil samples from a Costa-Rican rain forest, a Costa-Rican arable forest, and Greenland tundra) were defined as having a relatively high organic carbon content (1.66–7.51%) and an acidic soil pH (4.63–5.09). Interestingly, there was a significant correlation between the Chao1 diversity index and organic carbon content, with higher richness at lower levels of organic carbon (Fig. S5A). Moreover, a relatively weak correlation between the Chao1 index and soil pH was also observed, with the highest diversity at a pH of approximately 6 (Fig. S5B). These results were in good agreement with the previously reported *amoA* gene-based pyrotag analysis [14], and they provided further evidence of the importance of soil pH and organic carbon for defining the ecological niche of the AOA community in soil ecosystems, including relict niter-beds.

## Conclusions

16S rRNA and *amoA* gene-based pyrotag analyses revealed that microbes responsible for ammonia oxidation (both AOB and AOA) still persist in relict niter-bed soils, although saltpeter production ceased more than 100 years ago. Our study provided the first insights into the microbial diversity in relict niter-bed ecosystems. Our main conclusions are as follows. (i) Based on 16S rRNA gene pyrotag analysis, members of the phyla *Proteobacteria*, *Actinobacteria*, *Bacteroidetes, Chloroflexi, Firmicutes*, *Gemmatimonadetes*, and *Planctomycetes* were major microbial constituents in the niter-bed soil samples. The NO_3_^−^, Cl^−^, K^+^, Na^+^, and moisture contents were potential factors for the microbial compositions of the relict niter-bed soils. (ii) Members of *Nitrosospira* and “*Ca*. Nitrososphaera” were the major AOB and AOA populations, respectively, in the niter-bed soils. Soil pH, organic carbon content, and NO_3_^−^, Cl^−^, K^+^, and Na^+^ contents were important factors for the ecological niche of AOB and AOA in the relict niter-bed soils.

## Supporting Information

Figure S1 **Rarefaction curves of (A) 16S rRNA, (B) bacterial** ***amoA*** **, and (C) archaeal** ***amoA*** **gene sequences of nine niter-bed soils.**
(TIF)

Figure S2 **Jackknife clustering of (A) 16S rRNA, (B) bacterial** ***amoA*** **, and (C) archaeal** ***amoA*** **gene pyrotag libraries from the relict niter-bed soils (weighted UniFrac, normalized to 7,770, 2,320, and 2,130 reads per soil, respectively).**
(TIF)

Figure S3 **Extended error bar plot showing the OTUs that have significantly (** ***p*** **<0.05) different abundances in the group.** Groups were defined on the basis of principle coordinate analysis (Fig. 1) and Jackknife clustering of 16S rRNA gene pyrotag analysis (Fig. S2A). The OTUs overrepresented in upper group have a positive difference between relative abundances, and those overrepresented in bottom group have a negative difference between relative abundances. Only OTUs with mean proportions more than 0.01% in the total populations were shown in the plot.
(TIF)

Figure S4 **Principal coordinate analysis based on the abundances of (A) bacterial and (B) archaeal** ***amoA*** **gene OTUs (weighted UniFrac) only for niter-bed soils.** For this analysis, bacterial and archaeal *amoA* OTUs observed here were normalized to 2,320 and 2,130 reads per soil, respectively.
(TIF)

Figure S5 **Correlation analysis of organic carbon contents and soil pH to Chao1 diversity indexes.** Previously reported archaeal *amoA* pyrotag data from various soil environments [14] were combined with those from niter-bed soils.
(TIF)

Table S1 **Physicochemical parameters of niter-bed soil samples.**
(PDF)

Table S2 **Primer sequences for pyrotag analysis.**
(PDF)

Table S3 **The 16S rRNA gene pyrotag libraries of relict niter-bed soil.**
(PDF)

Table S4 **The OTU table of 16S rRNA gene pyrotag libraries.**
(PDF)

Table S5 **The relative abundances of ammonia-oxidizing bacteria and archaea in 16S rRNA gene pyrotag libraries.**
(PDF)

Table S6 **Phylogenetic identification of OTUs derived from bacterial and archaeal** ***amoA*** **gene sequences.**
(PDF)

## References

[1] Williams AR (1975) The production of saltpetre in the middle ages. Ambix 22: 125–133.

[2] Lockyer WJS (1908) Modern nitre beds. Nature 77: 513–515.

[3] Kyeser K (1967) Umschrift und Ubersetzung nebst Erlauterungen (German translation). Düsseldorf, Germany: Verlag des Vereins Deutscher Ingenieure.

[4] Kyeser K (1967) Facsimile-Ausgabe der Pergamenthandschrift, Cod. Ms. philos. 63 der Universititsbibliothek, Gottingen (German translation). Düsseldorf, Germany: Verlag des Vereins Deutscher Ingenieure.

[5] Multhauf RP (1971) The French Crash Program for Saltpeter Production, 1776–94. Technol Cult 12: 163–181.

[6] Kunnas J (2007) Potash, saltpeter and tar - Production, exports and use of wood in Finland in the 19th century. Scand J Hist 32: 281–311.

[7] Anonymous (1912) The Indian saltpetre industry. Nature 88: 330–331.

[8] Thorpe TE (1917) Indian saltpetre. Nature 99: 447–448.

[9] Maji T (2009) Transaction on saltpeter of Shirakawa Village in the second half of the 18th century described by old letters found from Shinshoji-temple. Annu Rep Fac Edu, Gifu Univ, Humanit Soc Sci (in Japanese with English abstract) 57: 121–126.

[10] Maji T, Maji A (2007) The Unknown History of Shirakawago: The Circulation of Nitrogen in Relation with Urine, Saltpeter and Rice, and The Daily Life in the Edo Era. Nagoya, Japan: Fubaisha Press.

[11] Anonymous (1912) The condition of the Chilean saltpeter industry. J Ind Eng Chem-Us 4: 687–687.

[12] Jimura T (2014) The impact of world heritage site designation on local communities - A case study of Ogimachi, Shirakawa-mura, Japan. Tourism Manage 32: 288–296.

[13] Maji T (2009) Efficiency of nitrification and activity of nitrite production by soil in niter-bed. Sci Rep Fac Ed Gifu Univ Natur Sci (in Japanese with English abstract) 33: 103–108.

[14] Pester M, Rattei T, Flechl S, Grongroft A, Richter A, et al (2012) *amoA*-based consensus phylogeny of ammonia-oxidizing archaea and deep sequencing of *amoA* genes from soils of four different geographic regions. Environ Microbiol 14: 525–539. 22141924 10.1111/j.1462-2920.2011.02666.x PMC3328746

[15] Maji T (2006) A study of saltpeter production at Shirakawa Village in Edo era. Part 3: an estimation of contribution of urinay nitrogen to nitrate-N in niter-bed. Kagakushi (in Japanese with English abstract) 33: 1–14.

[16] Maji T (2005) A study of saltpeter production at Shirakawa Village in Edo era. Part 2: productivity of saltpeter from artificial niter-bed soil: an estimation from extraction yield of nitrite. Kagakushi (in Japanese with English abstract) 32: 137–143.

[17] Maji T (2005) A study of saltpeter production at Shirakawa Village in Edo era. Part 1: an analysis of constituents of soil gathered from niter-bed and the starting materials. Kagakushi (in Japanese with English abstract) 32: 75–84.

[18] Baker GC, Smith JJ, Cowan DA (2003) Review and re-analysis of domain-specific 16S primers. J Microbiol Methods 55: 541–555. 14607398 10.1016/j.mimet.2003.08.009

[19] Rotthauwe JH, Witzel KP, Liesack W (1997) The ammonia monooxygenase structural gene amoA as a functional marker: Molecular fine-scale analysis of natural ammonia-oxidizing populations. Appl Environ Microbiol 63: 4704–4712. 9406389 10.1128/aem.63.12.4704-4712.1997 PMC168793

[20] Francis CA, Roberts KJ, Beman JM, Santoro AE, Oakley BB (2005) Ubiquity and diversity of ammonia-oxidizing archaea in water columns and sediments of the ocean. Proc Natl Acad Sci USA 102: 14683–14688. 16186488 10.1073/pnas.0506625102 PMC1253578

[21] Caporaso JG, Kuczynski J, Stombaugh J, Bittinger K, Bushman FD, et al (2010) QIIME allows analysis of high-throughput community sequencing data. Nat Methods 7: 335–336. 20383131 10.1038/nmeth.f.303 PMC3156573

[22] Reeder J, Knight R (2010) Rapidly denoising pyrosequencing amplicon reads by exploiting rank-abundance distributions. Nat Methods 7: 668–669. 20805793 10.1038/nmeth0910-668b PMC2945879

[23] Edgar RC (2010) Search and clustering orders of magnitude faster than BLAST. Bioinformatics 26: 2460–2461. 20709691 10.1093/bioinformatics/btq461

[24] Caporaso JG, Bittinger K, Bushman FD, DeSantis TZ, Andersen GL, et al (2010) PyNAST: a flexible tool for aligning sequences to a template alignment. Bioinformatics 26: 266–267. 19914921 10.1093/bioinformatics/btp636 PMC2804299

[25] Haas BJ, Gevers D, Earl AM, Feldgarden M, Ward DV, et al (2011) Chimeric 16S rRNA sequence formation and detection in Sanger and 454-pyrosequenced PCR amplicons. Genome Res 21: 494–504. 21212162 10.1101/gr.112730.110 PMC3044863

[26] Wang Y, Qian PY (2009) Conservative fragments in bacterial 16S rRNA genes and primer design for 16S ribosomal DNA amplicons in metagenomic studies. PLoS One 4: e7401. 19816594 10.1371/journal.pone.0007401 PMC2754607

[27] Cole JR, Wang Q, Cardenas E, Fish J, Chai B, et al (2009) The Ribosomal Database Project: improved alignments and new tools for rRNA analysis. Nucleic Acids Res 37: D141–D145. 19004872 10.1093/nar/gkn879 PMC2686447

[28] Camacho C, Coulouris G, Avagyan V, Ma N, Papadopoulos J, et al (2009) BLAST+: architecture and applications. BMC Bioinformatics 10: 421. 20003500 10.1186/1471-2105-10-421 PMC2803857

[29] Tamura K, Peterson D, Peterson N, Stecher G, Nei M, et al (2011) MEGA5: Molecular Evolutionary Genetics Analysis Using Maximum Likelihood, Evolutionary Distance, and Maximum Parsimony Methods. Mol Biol Evol 28: 2731–2739. 21546353 10.1093/molbev/msr121 PMC3203626

[30] Edgar RC, Haas BJ, Clemente JC, Quince C, Knight R (2011) UCHIME improves sensitivity and speed of chimera detection. Bioinformatics 27: 2194–2200. 21700674 10.1093/bioinformatics/btr381 PMC3150044

[31] Colwell RK (2013) EstimateS: Statistical estimation of species richness and shared species from samples. Version 9 and earlier. User’s Guide and application.

[32] Good IJ (1953) The population frequencies of species and the estimation of population parameters. Biometrika 40: 237–264.

[33] Hamady M, Lozupone C, Knight R (2010) Fast UniFrac: facilitating high-throughput phylogenetic analyses of microbial communities including analysis of pyrosequencing and PhyloChip data. ISME J 4: 17–27. 19710709 10.1038/ismej.2009.97 PMC2797552

[34] Saitou N, Nei M (1987) The neighbor-joining method - a new method for reconstructing phylogenetic trees. Mol Biol Evol 4: 406–425. 3447015 10.1093/oxfordjournals.molbev.a040454

[35] Welch BL (1947) The generalization of students problem when several different population variances are involved. Biometrika 34: 28–35. 20287819 10.1093/biomet/34.1-2.28

[36] Parks DH, Beiko RG (2010) Identifying biologically relevant differences between metagenomic communities. Bioinformatics 26: 715–721. 20130030 10.1093/bioinformatics/btq041

[37] Felsenstein J (1985) Confidence-limits on phylogenies - an approach using the bootstrap. Evolution 39: 783–791. 28561359 10.1111/j.1558-5646.1985.tb00420.x

[38] Mao YJ, Yannarell AC, Mackie RI (2011) Changes in N-transforming archaea and bacteria in soil during the establishment of bioenergy crops. PLoS One 6: e24750. 21935454 10.1371/journal.pone.0024750 PMC3173469

[39] Xue K, Wu LY, Deng Y, He ZL, Van Nostrand J, et al (2013) Functional gene differences in soil microbial communities from conventional, low-input, and organic farmlands. Appl Environ Microbiol 79: 1284–1292. 23241975 10.1128/AEM.03393-12 PMC3568620

[40] Grossman JM, O’Neill BE, Tsai SM, Liang BQ, Neves E, et al (2010) Amazonian anthrosols support similar microbial communities that differ distinctly from those extant in adjacent, unmodified soils of the same mineralogy. Microb Ecol 60: 192–205. 20574826 10.1007/s00248-010-9689-3

[41] Corneo PE, Pellegrini A, Cappellin L, Roncador M, Chierici M, et al (2013) Microbial community structure in vineyard soils across altitudinal gradients and in different seasons. FEMS Microbiol Ecol 84: 588–602. 23398556 10.1111/1574-6941.12087

[42] Van Horn DJ, Van Horn ML, Barrett JE, Gooseff MN, Altrichter AE, et al (2013) Factors controlling soil microbial biomass and bacterial diversity and community composition in a cold desert ecosystem: role of geographic scale. PLoS One 8: e66103. 23824063 10.1371/journal.pone.0066103 PMC3688848

[43] Watson SW (1971) Reisolation of *Nitrosospira briensis* Winogradsky, S and Winogradsky, H 1933. Arch Mikrobiol 75: 179–188. 5541686 10.1007/BF00408979

[44] Ward BB, Eveillard D, Kirshtein JD, Nelson JD, Voytek MA, et al (2007) Ammonia-oxidizing bacterial community composition in estuarine and oceanic environments assessed using a functional gene microarray. Environ Microbiol 9: 2522–2538. 17803777 10.1111/j.1462-2920.2007.01371.x

[45] Mintie AT, Heichen RS, Cromack K, Myrold DD, Bottomley PJ (2003) Ammonia-oxidizing bacteria along meadow-to-forest transects in the oregon cascade mountains. Appl Environ Microbiol 69: 3129–3136. 12788707 10.1128/AEM.69.6.3129-3136.2003 PMC161520

[46] Bollmann A, Schmidt I, Saunders AM, Nicolaisen MH (2005) Influence of starvation on potential ammonia-oxidizing activity and *amoA* mRNA levels of *Nitrosospira briensis* . Appl Environ Microbiol 71: 1276–1282. 15746329 10.1128/AEM.71.3.1276-1282.2005 PMC1065156

[47] Li XR, Xiao YP, Ren WW, Liu ZF, Shi JH, et al (2012) Abundance and composition of ammonia-oxidizing bacteria and archaea in different types of soil in the Yangtze River estuary. J Zhejiang Univ-Sc B 13: 769–782. 10.1631/jzus.B1200013 PMC3468820 23024044

[48] Fukushima T, Whang LM, Chiang TY, Lin YH, Chevalier LR, et al (2013) Nitrifying bacterial community structures and their nitrification performance under sufficient and limited inorganic carbon conditions. Appl Microbiol Biotechnol 97: 6513–6523. 23053088 10.1007/s00253-012-4436-y

[49] Shore JL, M’Coy WS, Gunsch CK, Deshusses MA (2012) Application of a moving bed biofilm reactor for tertiary ammonia treatment in high temperature industrial wastewater. Bioresour Technol 112: 51–60. 22444639 10.1016/j.biortech.2012.02.045

[50] Avrahami S, Jia ZJ, Neufeld JD, Murrell JC, Conrad R, et al (2011) Active autotrophic ammonia-oxidizing bacteria in biofilm enrichments from simulated creek ecosystems at two ammonium concentrations respond to temperature manipulation. Appl Environ Microbiol 77: 7329–7338. 21890674 10.1128/AEM.05864-11 PMC3194856

[51] Eilmus S, Rosch C, Bothe H (2007) Prokaryotic life in a potash-polluted marsh with emphasis on N-metabolizing microorganisms. Environ Pollut 146: 478–491. 16979273 10.1016/j.envpol.2006.07.008

[52] Gubry-Rangin C, Nicol GW, Prosser JI (2010) Archaea rather than bacteria control nitrification in two agricultural acidic soils. FEMS Microbiol Ecol 74: 566–574. 21039653 10.1111/j.1574-6941.2010.00971.x

[53] Shen JP, Zhang LM, Zhu YG, Zhang JB, He JZ (2008) Abundance and composition of ammonia-oxidizing bacteria and ammonia-oxidizing archaea communities of an alkaline sandy loam. Environ Microbiol 10: 1601–1611. 18336563 10.1111/j.1462-2920.2008.01578.x

[54] Tarre S, Shlafman E, Beliavski M, Green M (2007) Changes in ammonia oxidiser population during transition to low pH in a biofilm reactor starting with *Nitrosomonas europaea* . Water Sci Technol 55: 363–368. 17547006 10.2166/wst.2007.278

[55] Yasuda T, Waki M, Kuroda K, Hanajima D, Fukumoto Y, et al (2013) Responses of community structure of amoA-encoding archaea and ammonia-oxidizing bacteria in ammonia biofilter with rockwool mixtures to the gradual increases in ammonium and nitrate. J Appl Microbiol 114: 746–761. 23198809 10.1111/jam.12091

[56] Leininger S, Urich T, Schloter M, Schwark L, Qi J, et al (2006) Archaea predominate among ammonia-oxidizing prokaryotes in soils. Nature 442: 806–809. 16915287 10.1038/nature04983

[57] Jia ZJ, Conrad R (2009) *Bacteria* rather than *Archaea* dominate microbial ammonia oxidation in an agricultural soil. Environ Microbiol 11: 1658–1671. 19236445 10.1111/j.1462-2920.2009.01891.x

[58] Moin NS, Nelson KA, Bush A, Bernhard AE (2009) Distribution and diversity of archaeal and bacterial ammonia oxidizers in salt marsh sediments. Appl Environ Microbiol 75: 7461–7468. 19801456 10.1128/AEM.01001-09 PMC2786404

[59] Dang HY, Zhang XX, Sun J, Li TG, Zhang ZN, et al (2008) Diversity and spatial distribution of sediment ammonia-oxidizing crenarchaeota in response to estuarine and environmental gradients in the Changjiang Estuary and East China Sea. Microbiology-SGM 154: 2084–2095. 10.1099/mic.0.2007/013581-0 18599836

[60] Park HD, Wells GF, Bae H, Criddle CS, Francis CA (2006) Occurrence of ammonia-oxidizing archaea in wastewater treatment plant bioreactors. Appl Environ Microbiol 72: 5643–5647. 16885322 10.1128/AEM.00402-06 PMC1538709

