# Supplementary figures and images for: Microbial Community Structure of Relict Niter-Beds Previously Used for Saltpeter Production
Source: PLoS One. 2014 Aug 11;9(8):e104752. doi: 10.1371/journal.pone.0104752 (PMC4128746; doi:10.1371/journal.pone.0104752)

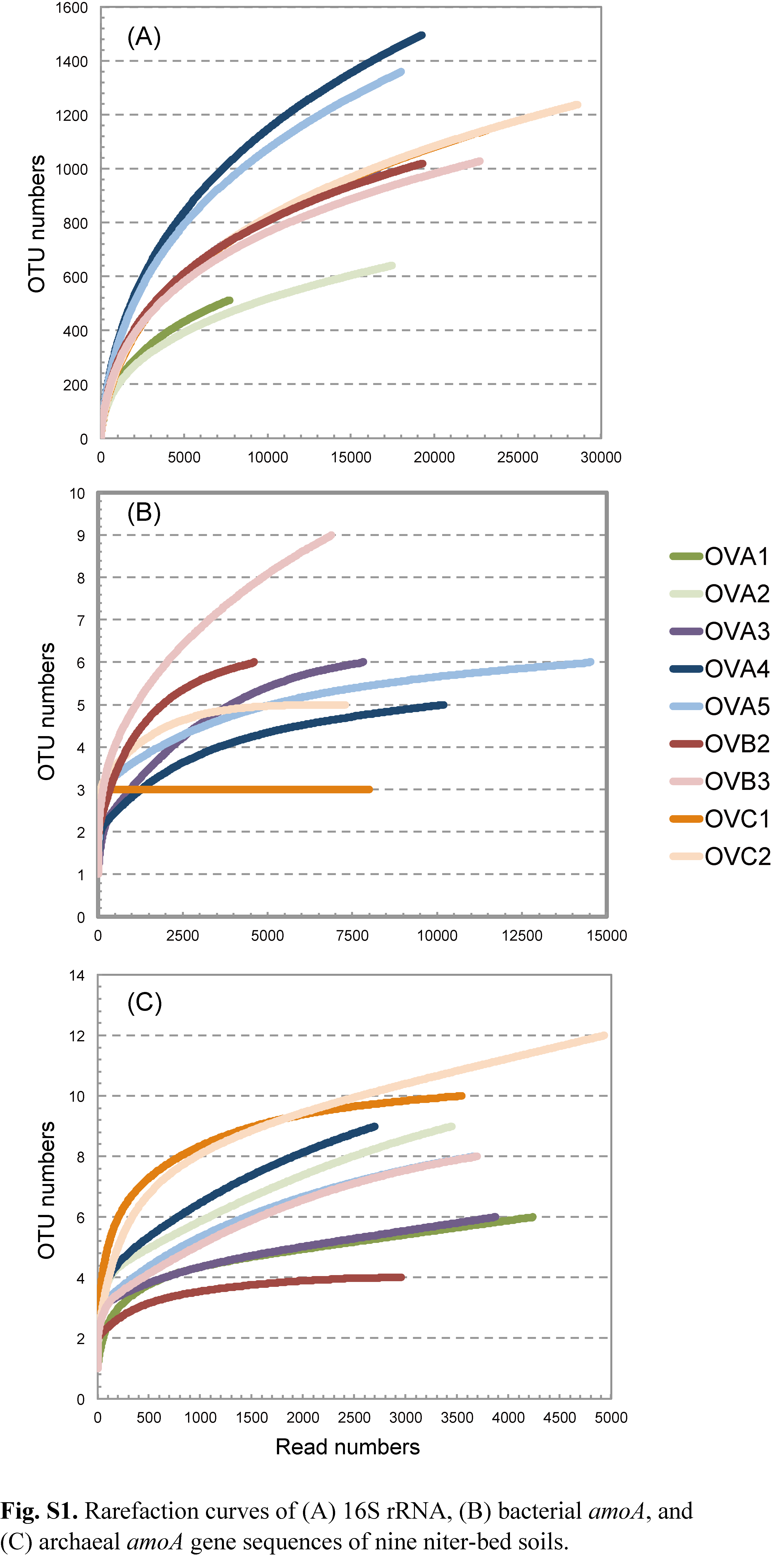

Supplement: Figure S1 — Rarefaction curves of (A) 16S rRNA, (B) bacterial amoA , and (C) archaeal amoA gene sequences of nine niter-bed soils. (TIF) [file pone.0104752.s001.tif]

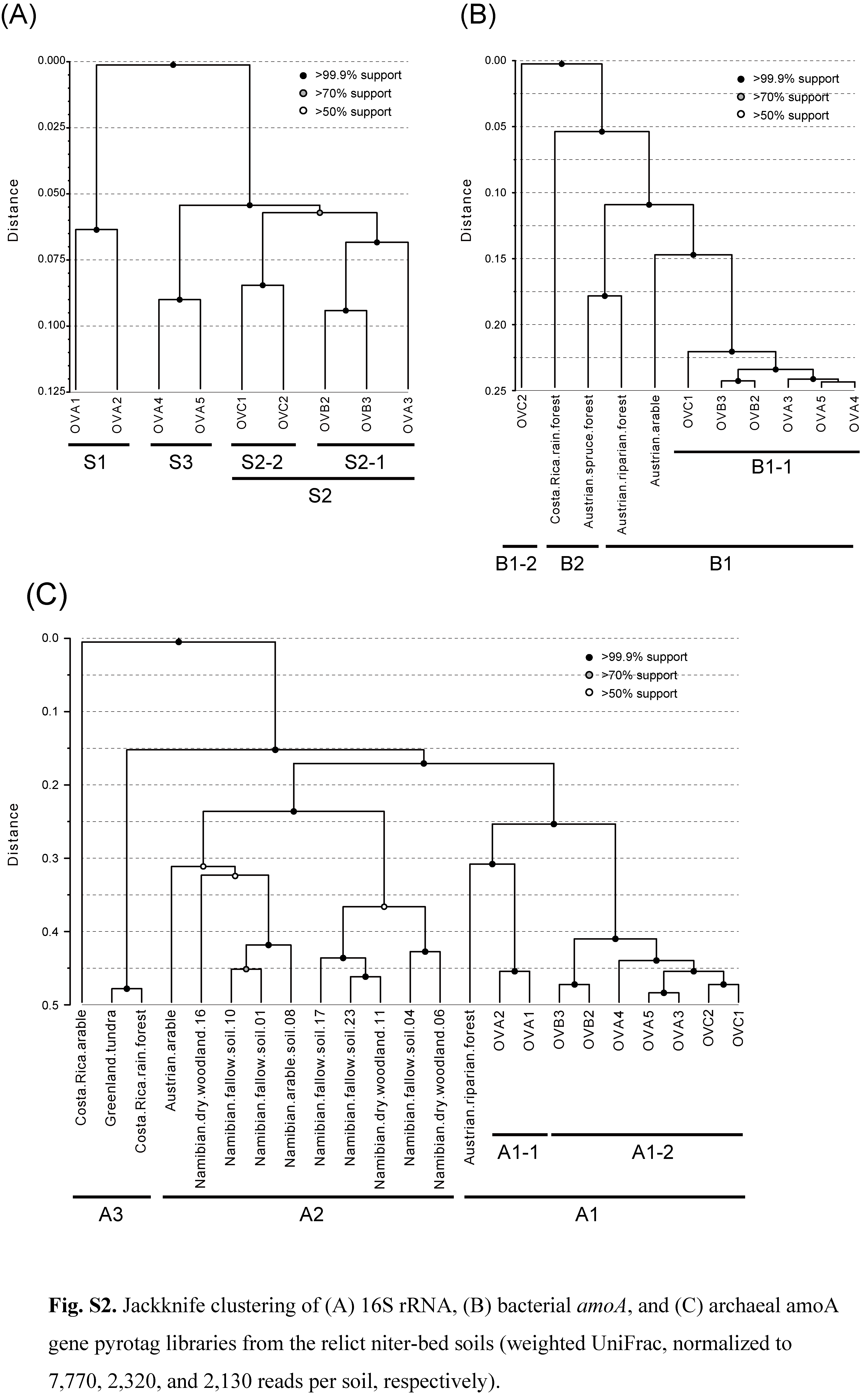

Supplement: Figure S2 — Jackknife clustering of (A) 16S rRNA, (B) bacterial amoA , and (C) archaeal amoA gene pyrotag libraries from the relict niter-bed soils (weighted UniFrac, normalized to 7,770, 2,320, and 2,130 reads per soil, respectively). (TIF) [file pone.0104752.s002.tif]

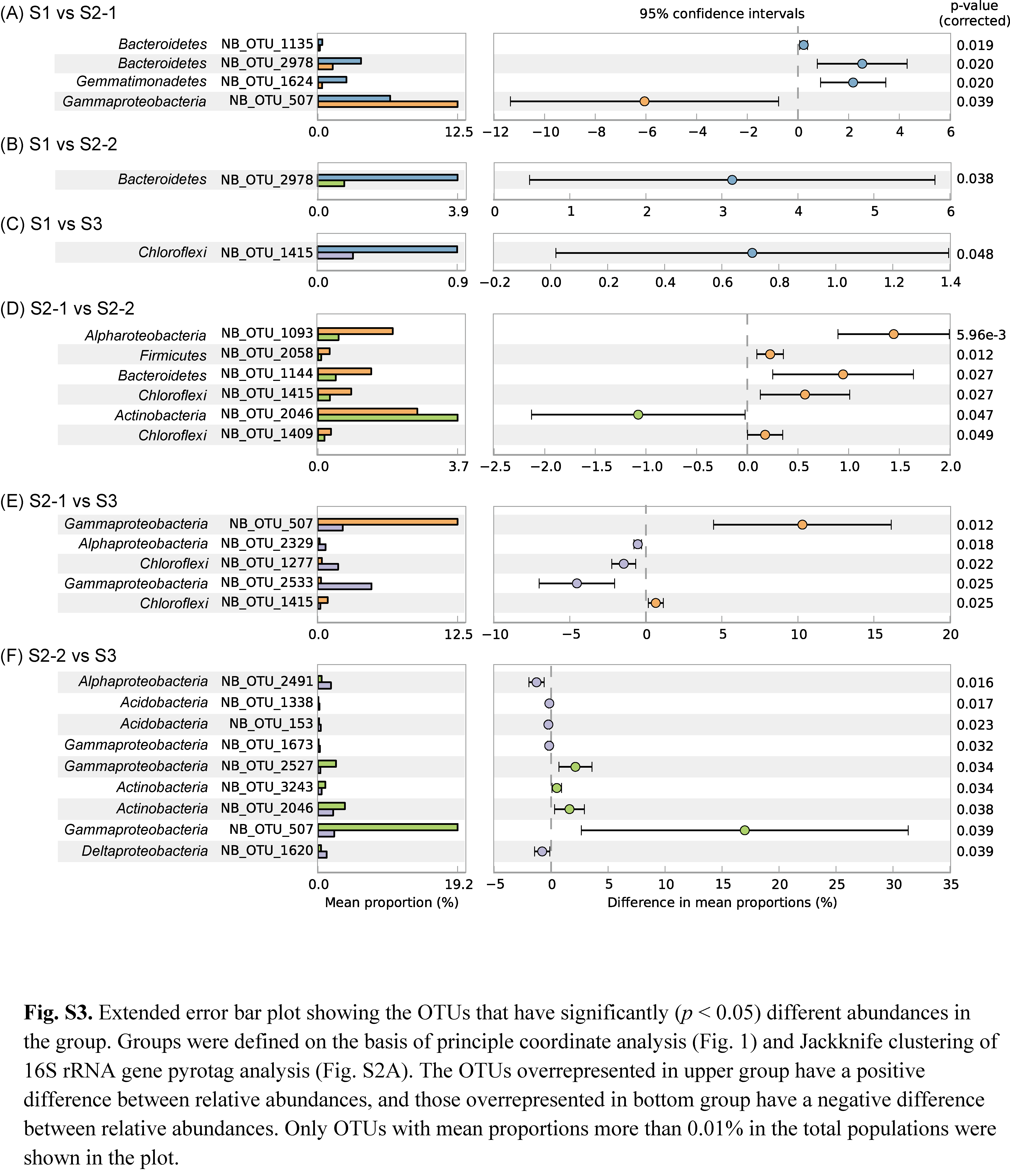

Supplement: Figure S3 — Extended error bar plot showing the OTUs that have significantly ( p <0.05) different abundances in the group. Groups were defined on the basis of principle coordinate analysis (Fig. 1) and Jackknife clustering of 16S rRNA gene pyrotag analysis (Fig. S2A). The OTUs overrepresented in upper group have a positive difference between relative abundances, and those overrepresented in bottom group have a negative difference between relative abundances. Only OTUs with mean proportions more than 0.01% in the total populations were shown in the plot. (TIF) [file pone.0104752.s003.tif]

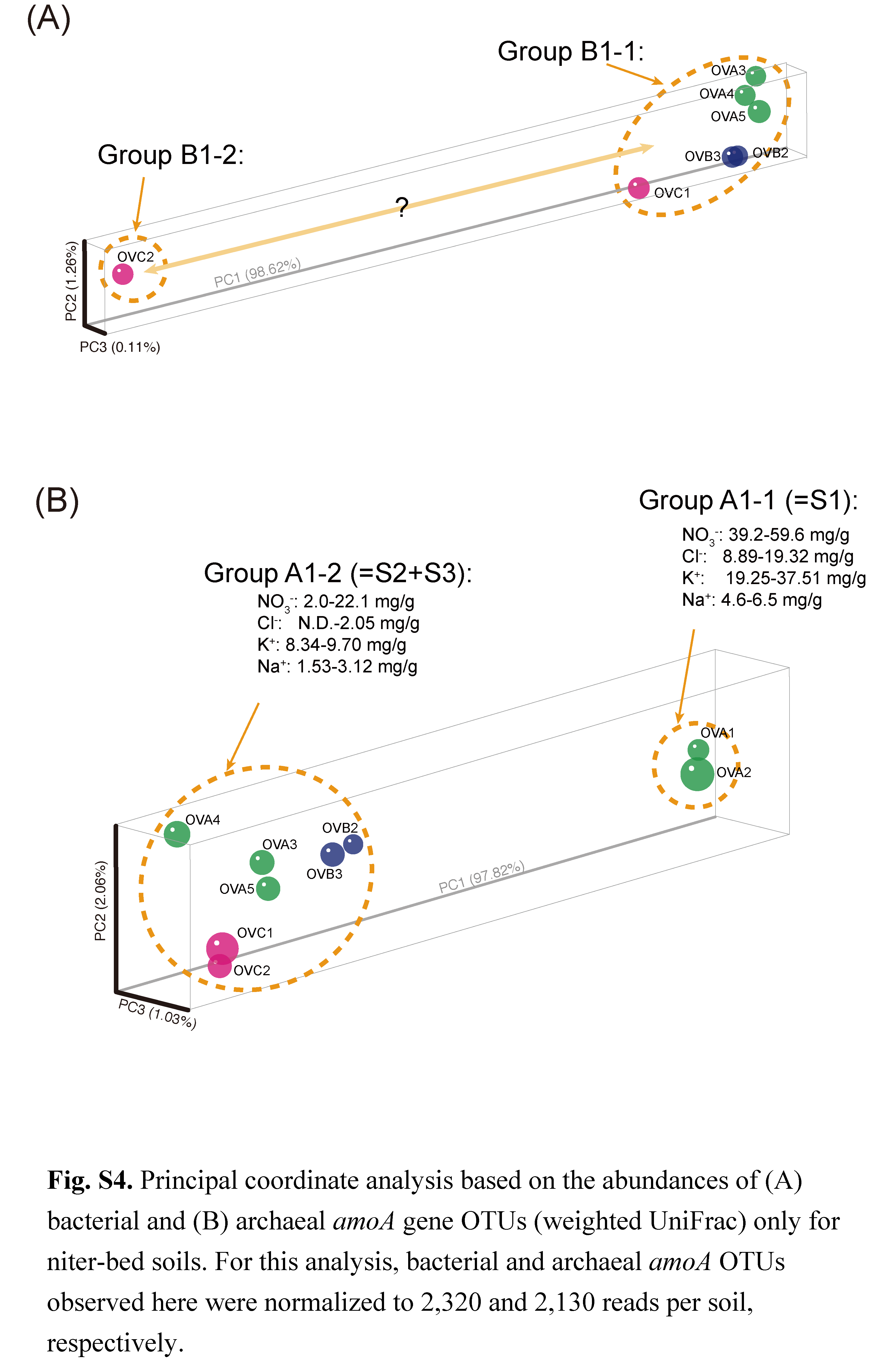

Supplement: Figure S4 — Principal coordinate analysis based on the abundances of (A) bacterial and (B) archaeal amoA gene OTUs (weighted UniFrac) only for niter-bed soils. For this analysis, bacterial and archaeal amoA OTUs observed here were normalized to 2,320 and 2,130 reads per soil, respectively. (TIF) [file pone.0104752.s004.tif]

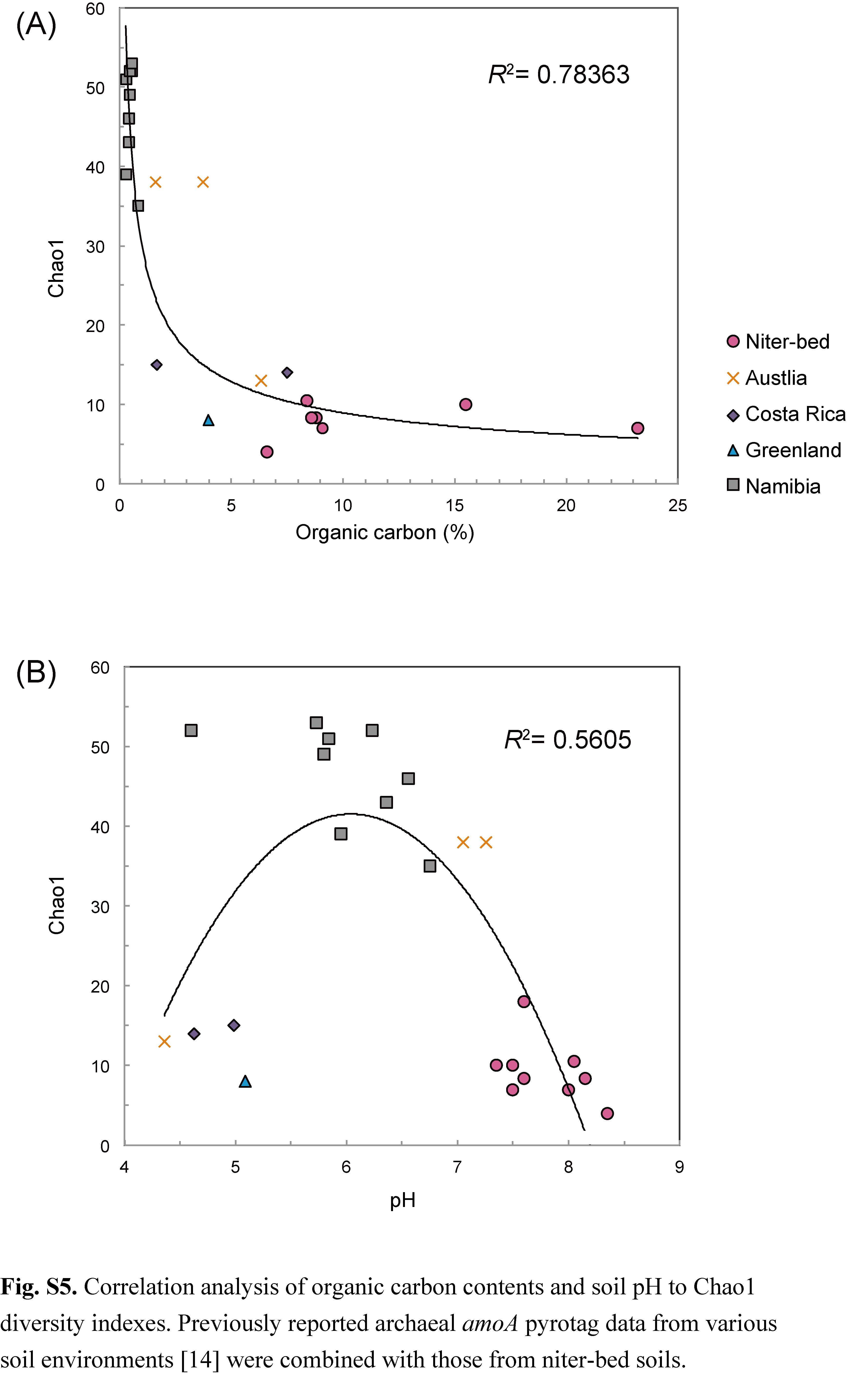

Supplement: Figure S5 — Correlation analysis of organic carbon contents and soil pH to Chao1 diversity indexes. Previously reported archaeal amoA pyrotag data from various soil environments [14] were combined with those from niter-bed soils. (TIF) [file pone.0104752.s005.tif]
