# Supplementary material for: Microbial Community Structure of Relict Niter-Beds Previously Used for Saltpeter Production
Source: PLoS One. 2014 Aug 11;9(8):e104752. doi: 10.1371/journal.pone.0104752 (PMC4128746; doi:10.1371/journal.pone.0104752)
Supplement: Table S1 — Physicochemical parameters of niter-bed soil samples. (PDF) [file pone.0104752.s006.pdf]

Table S1. Physicochemical parameters of niter-bed soil samples[a]

| Sample ID | Soil pH | Moisture (%) | Ignission loss (%) | Organic compound (%) | Insoluble minerals (%) | Maximum nitrification rate (μg/g soil/day) | Inorganic ion concentration (mg/g-soil) | | | | | | | | | | |
|---|---|---|---|---|---|---|---|---|---|---|---|---|---|---|---|---|---|
| | | | | | | | $NO_3$ | Cl | $SiO_2$ | $P_2O_5$ | K | Na | Ca | Mg | Al | $SO_3$ | Fe |
| OVA1 | 7.50 | 21.2 | 44.4 | 23.2 | 29.6 | 5.6 | 59.6 | 19.32 | 109.4 | 28.3 | 37.51 | 6.50 | 57.8 | 9.35 | 9.10 | 12.0 | 8.34 |
| OVA2 | 7.50 | 16.0 | 32.5 | 15.5 | 23.6 | 0.5 | 39.2 | 8.89 | 89.7 | 37.1 | 19.25 | 4.60 | 136.1 | 17.06 | 9.87 | 10.7 | 7.29 |
| OVA3 | 8.00 | 14.0 | 23.1 | 9.1 | 27.9 | 184.4 | 22.1 | 1.78 | 114.7 | 36.7 | 9.70 | 2.17 | 121.7 | 15.64 | 11.02 | 6.8 | 6.72 |
| OVA4 | 8.05 | 19.8 | 27.2 | 8.4 | 45.6 | 120.0 | 2.0 | N.D. | 100.9 | 32.2 | 8.84 | 1.58 | 78.1 | 6.49 | 18.07 | 4.6 | 11.44 |
| OVA5 | 8.15 | 12.1 | 19.9 | 8.8 | 31.6 | 664.2 | 4.6 | 0.14 | 114.4 | 37.9 | 8.55 | 1.53 | 119.0 | 13.38 | 11.85 | 4.3 | 7.05 |
| OVB2 | 8.35 | 5.0 | 11.6 | 6.6 | 57.0 | N.T. | 11.1 | 1.37 | 71.3 | 30.2 | 8.36 | 1.68 | 65.4 | 7.19 | 10.75 | 2.4 | 6.44 |
| OVB3 | 7.60 | 5.2 | 13.9 | 8.6 | 54.6 | N.T. | 14.9 | 1.61 | 67.8 | 19.4 | 8.69 | 2.31 | 74.7 | 9.82 | 12.10 | 2.4 | 5.27 |
| OVC1 | 7.35 | 3.6 | N.T. | N.T. | N.T. | N.T. | 11.9 | 2.05 | 69.4 | N.T. | 8.34 | 3.12 | 75.3 | 11.54 | N.T. | N.T. | N.T. |
| OVC2 | 7.60 | 3.9 | N.T. | N.T. | N.T. | N.T. | 9.5 | 1.19 | 101.6 | 13.8 | 8.93 | 2.21 | 88.6 | 13.60 | N.T. | N.T. | N.T. |

[a] Data were adopted from previous reports [13, 17]; N.T., not tested; N.D., not detected
