# Supplementary material for: Microbial Community Structure of Relict Niter-Beds Previously Used for Saltpeter Production
Source: PLoS One. 2014 Aug 11;9(8):e104752. doi: 10.1371/journal.pone.0104752 (PMC4128746; doi:10.1371/journal.pone.0104752)
Supplement: Table S2 — Primer sequences for pyrotag analysis. (PDF) [file pone.0104752.s007.pdf]

Table S2. Primer sequences for pyrotag analysis

Target	Soil sample	Primer name	5'-fusion-MID-target sequence-3'
16S rRNA	OVA1	Aside fusion-MID11-Univ519F	CGTATCGCCTCCCTCGCGCCATCAG-TGATACGTCT-CAGCMGCCGCGGTAATWC
		Bside fusion-MID11-Univ926R	CTATGCGCCTTGCCAGCCCGCTCAG-TGATACGTCT-CCGTCAATTCMTTTRAGTT
	OVA2	Aside fusion-MID14-Univ519F	CGTATCGCCTCCCTCGCGCCATCAG-CGAGAGATAC-CAGCMGCCGCGGTAATWC
		Bside fusion-MID14-Univ926R	CTATGCGCCTTGCCAGCCCGCTCAG-CGAGAGATAC-CCGTCAATTCMTTTRAGTT
	OVA3	Aside fusion-MID18-Univ519F	CGTATCGCCTCCCTCGCGCCATCAG-TCTACGTAGC-CAGCMGCCGCGGTAATWC
		Bside fusion-MID18-Univ926R	CTATGCGCCTTGCCAGCCCGCTCAG-TCTACGTAGC-CCGTCAATTCMTTTRAGTT
	OVA4	Aside fusion-MID28-Univ519F	CGTATCGCCTCCCTCGCGCCATCAG-ACTACTATGT-CAGCMGCCGCGGTAATWC
		Bside fusion-MID28-Univ926R	CTATGCGCCTTGCCAGCCCGCTCAG-ACTACTATGT-CCGTCAATTCMTTTRAGTT
	OVA5	Aside fusion-MID31-Univ519F	CGTATCGCCTCCCTCGCGCCATCAG-AGCGTCGTCT-CAGCMGCCGCGGTAATWC
		Bside fusion-MID31-Univ926R	CTATGCGCCTTGCCAGCCCGCTCAG-AGCGTCGTCT-CCGTCAATTCMTTTRAGTT
	OVB2	Aside fusion-MID46-Univ519F	CGTATCGCCTCCCTCGCGCCATCAG-TGACGTATGT-CAGCMGCCGCGGTAATWC
		Bside fusion-MID46-Univ926R	CTATGCGCCTTGCCAGCCCGCTCAG-TGACGTATGT-CCGTCAATTCMTTTRAGTT
	OVB3	Aside fusion-MID48-Univ519F	CGTATCGCCTCCCTCGCGCCATCAG-ACAGTATATA-CAGCMGCCGCGGTAATWC
		Bside fusion-MID48-Univ926R	CTATGCGCCTTGCCAGCCCGCTCAG-ACAGTATATA-CCGTCAATTCMTTTRAGTT
	OVC1	Aside fusion-MID32-Univ519F	CGTATCGCCTCCCTCGCGCCATCAG-AGTACGCTAT-CAGCMGCCGCGGTAATWC
		Bside fusion-MID32-Univ926R	CTATGCGCCTTGCCAGCCCGCTCAG-AGTACGCTAT-CCGTCAATTCMTTTRAGTT
	OVC2	Aside fusion-MID38-Univ519F	CGTATCGCCTCCCTCGCGCCATCAG-TACACGTGAT-CAGCMGCCGCGGTAATWC
		Bside fusion-MID38-Univ926R	CTATGCGCCTTGCCAGCCCGCTCAG-TACACGTGAT-CCGTCAATTCMTTTRAGTT
Bacterial amoA	OVA3	Aside fusion-MID03-amoA1F	CGTATCGCCTCCCTCGCGCCATCAG-AGACGCACTC-GGGGTTTCTACTGGTGGT
		Bside fusion-MID03-amoA2R	CTATGCGCCTTGCCAGCCCGCTCAG-AGACGCACTC-CCCCTCKGSAAAGCCTTCTTC
	OVA4	Aside fusion-MID04-amoA1F	CGTATCGCCTCCCTCGCGCCATCAG-AGCACTGTAG-GGGGTTTCTACTGGTGGT
		Bside fusion-MID04-amoA2R	CTATGCGCCTTGCCAGCCCGCTCAG-AGCACTGTAG-CCCCTCKGSAAAGCCTTCTTC
	OVA5	Aside fusion-MID05-amoA1F	CGTATCGCCTCCCTCGCGCCATCAG-ATCAGACACG-GGGGTTTCTACTGGTGGT
		Bside fusion-MID05-amoA2R	CTATGCGCCTTGCCAGCCCGCTCAG-ATCAGACACG-CCCCTCKGSAAAGCCTTCTTC
	OVB2	Aside fusion-MID08-amoA1F	CGTATCGCCTCCCTCGCGCCATCAG-CTCGCGTGTC-GGGGTTTCTACTGGTGGT
		Bside fusion-MID08-amoA2R	CTATGCGCCTTGCCAGCCCGCTCAG-CTCGCGTGTC-CCCCTCKGSAAAGCCTTCTTC
	OVB3	Aside fusion-MID10-amoA1F	CGTATCGCCTCCCTCGCGCCATCAG-TCTCTATGCG-GGGGTTTCTACTGGTGGT
		Bside fusion-MID10-amoA2R	CTATGCGCCTTGCCAGCCCGCTCAG-TCTCTATGCG-CCCCTCKGSAAAGCCTTCTTC
	OVC1	Aside fusion-MID06-amoA1F	CGTATCGCCTCCCTCGCGCCATCAG-ATATCGCGAG-GGGGTTTCTACTGGTGGT
		Bside fusion-MID06-amoA2R	CTATGCGCCTTGCCAGCCCGCTCAG-ATATCGCGAG-CCCCTCKGSAAAGCCTTCTTC
	OVC2	Aside fusion-MID07-amoA1F	CGTATCGCCTCCCTCGCGCCATCAG-CGTGTCTCTA-GGGGTTTCTACTGGTGGT
		Bside fusion-MID07-amoA2R	CTATGCGCCTTGCCAGCCCGCTCAG-CGTGTCTCTA-CCCCTCKGSAAAGCCTTCTTC
Archaeal amoA	OVA1	Aside fusion-MID16-Arch amoAF	CGTATCGCCTCCCTCGCGCCATCAG-TCACGTACTA-STAATGGTCTGGCTTAGACG
		Bside fusion-MID16-Arch amoAR	CTATGCGCCTTGCCAGCCCGCTCAG-TCACGTACTA-GCGGCCATCCATCTGTATG
	OVA2	Aside fusion-MID17-Arch amoAF	CGTATCGCCTCCCTCGCGCCATCAG-CGTCTAGTAC-STAATGGTCTGGCTTAGACG
		Bside fusion-MID17-Arch amoAR	CTATGCGCCTTGCCAGCCCGCTCAG-CGTCTAGTAC-GCGGCCATCCATCTGTATG
	OVA3	Aside fusion-MID19-Arch amoAF	CGTATCGCCTCCCTCGCGCCATCAG-TGTACTACTC-STAATGGTCTGGCTTAGACG
		Bside fusion-MID19-Arch amoAR	CTATGCGCCTTGCCAGCCCGCTCAG-TGTACTACTC-GCGGCCATCCATCTGTATG
	OVA4	Aside fusion-MID21-Arch amoAF	CGTATCGCCTCCCTCGCGCCATCAG-CGTAGACTAG-STAATGGTCTGGCTTAGACG
		Bside fusion-MID21-Arch amoAR	CTATGCGCCTTGCCAGCCCGCTCAG-CGTAGACTAG-GCGGCCATCCATCTGTATG
	OVA5	Aside fusion-MID22-Arch amoAF	CGTATCGCCTCCCTCGCGCCATCAG-TACGAGTATG-STAATGGTCTGGCTTAGACG
		Bside fusion-MID22-Arch amoAR	CTATGCGCCTTGCCAGCCCGCTCAG-TACGAGTATG-GCGGCCATCCATCTGTATG
	OVB2	Aside fusion-MID25-Arch amoAF	CGTATCGCCTCCCTCGCGCCATCAG-TCGTCGCTCG-STAATGGTCTGGCTTAGACG
		Bside fusion-MID25-Arch amoAR	CTATGCGCCTTGCCAGCCCGCTCAG-TCGTCGCTCG-GCGGCCATCCATCTGTATG
	OVB3	Aside fusion-MID26-Arch amoAF	CGTATCGCCTCCCTCGCGCCATCAG-ACATACGCGT-STAATGGTCTGGCTTAGACG
		Bside fusion-MID26-Arch amoAR	CTATGCGCCTTGCCAGCCCGCTCAG-ACATACGCGT-GCGGCCATCCATCTGTATG
	OVC1	Aside fusion-MID23-Arch amoAF	CGTATCGCCTCCCTCGCGCCATCAG-TACTCTCGTG-STAATGGTCTGGCTTAGACG
		Bside fusion-MID23-Arch amoAR	CTATGCGCCTTGCCAGCCCGCTCAG-TACTCTCGTG-GCGGCCATCCATCTGTATG
	OVC2	Aside fusion-MID24-Arch amoAF	CGTATCGCCTCCCTCGCGCCATCAG-TAGAGACGAG-STAATGGTCTGGCTTAGACG
		Bside fusion-MID24-Arch amoAR	CTATGCGCCTTGCCAGCCCGCTCAG-TAGAGACGAG-GCGGCCATCCATCTGTATG
