# Supplementary material for: Microbial Community Structure of Relict Niter-Beds Previously Used for Saltpeter Production
Source: PLoS One. 2014 Aug 11;9(8):e104752. doi: 10.1371/journal.pone.0104752 (PMC4128746; doi:10.1371/journal.pone.0104752)
Supplement: Table S3 — The 16S rRNA gene pyrotag libraries of relict niter-bed soil. (PDF) [file pone.0104752.s008.pdf]

Table S3. The 16S rRNA gene pyrotag libraries of relict niter-bed soil

Phylogeny assigned	Relative abundance (%)								
	OVA1	OVA2	OVA3	OVA4	OVA5	OVB2	OVB3	OVC1	OVC2
Domain *Bacteria*									
Proteobacteria									
Alphaproteobacteria	7.838	8.170	12.669	16.985	17.458	18.971	15.919	12.098	12.831
Betaproteobacteria	0.026		0.138	2.631	1.813	1.279	0.716	0.992	1.439
Gammaproteobacteria	17.181	22.504	24.803	15.054	16.937	17.464	21.665	36.144	32.068
Deltaproteobacteria	0.463	0.343	0.590	2.356	2.357	5.024	3.782	1.048	1.216
Other *Proteobacteria*	0.051	0.023	0.054	0.057	0.022	0.010	0.035	0.022	0.017
Acidobacteria			0.367	3.337	2.817	3.558	2.087	0.742	1.097
Actinobacteria	12.214	15.369	21.878	21.671	21.573	17.542	19.873	17.969	25.535
Armatimonadetes	0.360	0.417	0.253	0.784	0.621	0.689	0.483	0.246	0.269
Bacteroidetes	33.346	21.664	8.823	1.681	1.797	4.729	6.277	4.688	3.493
Chlamydiae			0.012	0.005					0.003
Chlorobi			0.036	0.234	0.094	0.026	0.009	0.151	0.122
Chloroflexi	6.100	6.181	8.649	13.643	14.219	11.591	11.654	8.687	9.142
Cyanobacteria			0.006	0.010		0.005	0.009	0.069	0.056
Elusimicrobia			0.006	0.052	0.039	0.010		0.004	0.010
Firmicutes	5.946	4.077	6.903	5.776	5.180	7.717	6.022	4.999	3.570
Gemmatimonadetes	3.758	4.282	4.129	8.583	6.777	3.926	3.400	4.865	4.017
Nitrospirae				0.239	0.311	0.342	0.154	0.198	0.241
Planctomycetes	7.439	7.250	6.675	4.063	4.686	3.972	5.087	3.852	2.333
Tenericutes		0.006	0.006		0.006				
Thermi	3.089	5.026	1.932	0.618	1.569	0.730	0.861	1.622	1.034
Verrucomicrobia	0.013		0.042	0.052	0.033	0.010	0.009	0.026	0.042
BRC1	0.013			0.026	0.011				0.003
CCM11b			0.006		0.017			0.022	0.014
GAL15								0.039	0.031
GN04				0.016	0.006			0.004	0.007
NKB19		0.006	0.054	0.026	0.033	0.005	0.009	0.035	0.010
OD1		0.017		0.005					
OP11			0.006	0.010	0.022			0.009	0.038
OP3			0.006	0.026	0.028	0.005			
OP9		0.006							
PAUC34f									0.003
pMC2A15						0.005			
SBR1093			0.006	0.016		0.021		0.065	0.035
SPAM		0.006	0.090	0.654	0.444	1.398	0.795	0.699	0.706
TG3		0.006	0.006		0.011				
TM6	0.579	2.121	0.494	0.125	0.133	0.311	0.356	0.138	0.220
TM7	0.051	0.034	0.163	0.623	0.505	0.005	0.026	0.354	0.119
WPS-2					0.006				
WS3				0.228	0.067	0.016	0.009	0.030	0.049
WS6				0.021		0.005	0.004	0.004	
Unknown cluster			0.006	0.213	0.111	0.347	0.347	0.060	0.028
Domain *Archaea*									
Crenarchaeota	0.090	0.063	0.072	0.156	0.161	0.124	0.180	0.069	0.157
Euryarchaeota	1.441	2.430	1.119	0.026	0.139	0.161	0.233	0.052	0.038
DHVE3									0.003
