# Supplementary material for: Microbial Community Structure of Relict Niter-Beds Previously Used for Saltpeter Production
Source: PLoS One. 2014 Aug 11;9(8):e104752. doi: 10.1371/journal.pone.0104752 (PMC4128746; doi:10.1371/journal.pone.0104752)
Supplement: Table S4 — The OTU table of 16S rRNA gene pyrotag libraries. (PDF) [file pone.0104752.s009.pdf]

Table S4. The OTU table of 16S rRNA gene pyrotag libraries

OTU ID	OVA1	OVA2	OVA3	OVA4	OVA5	OVB2	OVB3	OVC1	OVC2	Phylogeny assigned within:					BLAST analysis with RDP database			
										Phylum	Class	Order	Family	Genus	Closest strain	Identity (%)	e-value	Bit score
2898	0	0	0	0	0	1	4	0	0	Acidobacteria					S001933336 Candidatus Methylomirabilis oxyfera Lineage=Root;rootrank;Bacteria;domain;unclassified_Bacteria;	87.19	7.00E-116	418
1959	0	0	0	5	1	0	0	0	0	Acidobacteria					S003287266 Desulfomonile tiedjei DSM 6799 Lineage=Root;rootrank;Bacteria;domain;"Proteobacteria";phylum;Deltaproteobacteria;class;Syntrophobacterales;order;Syntrophaceae;family;Desulfomonile;genus	87.09	1.00E-113	411
1205	0	0	0	0	0	1	0	1	2	Acidobacteria					S003287266 Desulfomonile tiedjei DSM 6799 Lineage=Root;rootrank;Bacteria;domain;"Proteobacteria";phylum;Deltaproteobacteria;class;Syntrophobacterales;order;Syntrophaceae;family;Desulfomonile;genus	86.65	7.00E-111	401
1731	0	0	0	3	1	0	0	0	0	Acidobacteria					S003610437 Pelobacter carbinolicus (T); DSM 2380 Lineage=Root;rootrank;Bacteria;domain;"Proteobacteria";phylum;Deltaproteobacteria;class;Desulfuromonadales;order;Desulfuromonadaceae;family;Pelobacter;genus	86.76	1.00E-112	407
597	0	0	0	2	1	0	0	0	1	Acidobacteria					S003610437 Pelobacter carbinolicus (T); DSM 2380 Lineage=Root;rootrank;Bacteria;domain;"Proteobacteria";phylum;Deltaproteobacteria;class;Desulfuromonadales;order;Desulfuromonadaceae;family;Pelobacter;genus	87.18	2.00E-121	436
2621	0	0	0	0	2	0	0	1	0	Acidobacteria					S003747590 Ureibacillus suwonensis; FZWP-21 Lineage=Root;rootrank;Bacteria;domain;Firmicutes;phylum;Bacilli;class;Bacillales;order;Planococcaceae;family;Ureibacillus;genus	87.72	7.00E-126	451
1582	0	0	0	0	0	0	0	6	7	Acidobacteria					S002288402 Candidatus Koribacter versatilis Ellin345 Lineage=Root;rootrank;Bacteria;domain;"Acidobacteria";phylum;Acidobacteria_Gp1;class;Gp1;genus	94.86	1.00E-163	577
321	0	0	0	6	4	1	0	7	0	Acidobacteria	Acidobacteria	Acidobacteriales			S000900189 Vitis hybrid cultivar; 4M1_F12 Lineage=Root;rootrank;Bacteria;domain;"Acidobacteria";phylum;Acidobacteria_Gp6;class;Gp6;genus	92.81	5.00E-117	422
1449	0	0	0	7	0	0	0	1	0	Acidobacteria	Acidobacteria	Acidobacteriales			S000900189 Vitis hybrid cultivar; 4M1_F12 Lineage=Root;rootrank;Bacteria;domain;"Acidobacteria";phylum;Acidobacteria_Gp6;class;Gp6;genus	93.49	3.00E-120	433
2318	0	0	0	2	1	2	0	2	1	Acidobacteria	Acidobacteria	Acidobacteriales			S000900189 Vitis hybrid cultivar; 4M1_F12 Lineage=Root;rootrank;Bacteria;domain;"Acidobacteria";phylum;Acidobacteria_Gp6;class;Gp6;genus	97.97	3.00E-144	512
739	0	0	0	4	3	0	0	0	0	Acidobacteria	Acidobacteria	Acidobacteriales			S000900189 Vitis hybrid cultivar; 4M1_F12 Lineage=Root;rootrank;Bacteria;domain;"Acidobacteria";phylum;Acidobacteria_Gp6;class;Gp6;genus	93.92	3.00E-124	446
1269	0	0	0	0	0	2	1	0	0	Acidobacteria	Acidobacteria	Acidobacteriales			S000900189 Vitis hybrid cultivar; 4M1_F12 Lineage=Root;rootrank;Bacteria;domain;"Acidobacteria";phylum;Acidobacteria_Gp6;class;Gp6;genus	91.64	1.00E-113	411
1256	0	0	0	1	1	0	0	0	0	Acidobacteria	Acidobacteria	Acidobacteriales			S000900189 Vitis hybrid cultivar; 4M1_F12 Lineage=Root;rootrank;Bacteria;domain;"Acidobacteria";phylum;Acidobacteria_Gp6;class;Gp6;genus	93.22	2.00E-120	433
2305	0	0	0	1	0	0	0	0	0	Acidobacteria	Acidobacteria	Acidobacteriales			S000900189 Vitis hybrid cultivar; 4M1_F12 Lineage=Root;rootrank;Bacteria;domain;"Acidobacteria";phylum;Acidobacteria_Gp6;class;Gp6;genus	96.96	3.00E-139	496
1803	0	0	0	0	0	0	1	0	0	Acidobacteria	Acidobacteria	Acidobacteriales			S000900189 Vitis hybrid cultivar; 4M1_F12 Lineage=Root;rootrank;Bacteria;domain;"Acidobacteria";phylum;Acidobacteria_Gp6;class;Gp6;genus	90.6	1.00E-108	394
153	0	0	6	77	73	224	119	37	52	Acidobacteria	Acidobacteria	Acidobacteriales			S000900272 Verticillium dahliae; 4S2_F08 Lineage=Root;rootrank;Bacteria;domain;"Acidobacteria";phylum;Acidobacteria_Gp6;class;Gp6;genus	96.97	2.00E-140	499
2184	0	0	2	24	51	4	3	16	37	Acidobacteria	Acidobacteria	Acidobacteriales			S000900272 Verticillium dahliae; 4S2_F08 Lineage=Root;rootrank;Bacteria;domain;"Acidobacteria";phylum;Acidobacteria_Gp6;class;Gp6;genus	92.59	1.00E-118	427
498	0	0	4	23	18	3	2	4	5	Acidobacteria	Acidobacteria	Acidobacteriales			S000900272 Verticillium dahliae; 4S2_F08 Lineage=Root;rootrank;Bacteria;domain;"Acidobacteria";phylum;Acidobacteria_Gp6;class;Gp6;genus	96.63	1.00E-138	494
2517	0	0	3	14	9	3	6	0	3	Acidobacteria	Acidobacteria	Acidobacteriales			S000900272 Verticillium dahliae; 4S2_F08 Lineage=Root;rootrank;Bacteria;domain;"Acidobacteria";phylum;Acidobacteria_Gp6;class;Gp6;genus	94.61	1.00E-128	460
1892	0	0	0	0	2	5	16	0	2	Acidobacteria	Acidobacteria	Acidobacteriales			S000900272 Verticillium dahliae; 4S2_F08 Lineage=Root;rootrank;Bacteria;domain;"Acidobacteria";phylum;Acidobacteria_Gp6;class;Gp6;genus	94.22	8.00E-125	448
241	0	0	0	7	8	1	2	2	0	Acidobacteria	Acidobacteria	Acidobacteriales			S000900272 Verticillium dahliae; 4S2_F08 Lineage=Root;rootrank;Bacteria;domain;"Acidobacteria";phylum;Acidobacteria_Gp6;class;Gp6;genus	96.97	8.00E-140	497
2097	0	0	1	1	10	0	0	0	0	Acidobacteria	Acidobacteria	Acidobacteriales			S000900272 Verticillium dahliae; 4S2_F08 Lineage=Root;rootrank;Bacteria;domain;"Acidobacteria";phylum;Acidobacteria_Gp6;class;Gp6;genus	93.94	2.00E-125	449
521	0	0	0	2	3	0	0	1	1	Acidobacteria	Acidobacteria	Acidobacteriales			S000900272 Verticillium dahliae; 4S2_F08 Lineage=Root;rootrank;Bacteria;domain;"Acidobacteria";phylum;Acidobacteria_Gp6;class;Gp6;genus	93.6	1.00E-123	444
1666	0	0	2	4	0	0	0	0	0	Acidobacteria	Acidobacteria	Acidobacteriales			S000900272 Verticillium dahliae; 4S2_F08 Lineage=Root;rootrank;Bacteria;domain;"Acidobacteria";phylum;Acidobacteria_Gp6;class;Gp6;genus	96.63	4.00E-138	492
484	0	0	0	1	0	1	1	2	2	Acidobacteria	Acidobacteria	Acidobacteriales			S000900272 Verticillium dahliae; 4S2_F08 Lineage=Root;rootrank;Bacteria;domain;"Acidobacteria";phylum;Acidobacteria_Gp6;class;Gp6;genus	96.6	5.00E-137	488
46	0	0	0	1	1	0	0	0	0	Acidobacteria	Acidobacteria	Acidobacteriales			S000900272 Verticillium dahliae; 4S2_F08 Lineage=Root;rootrank;Bacteria;domain;"Acidobacteria";phylum;Acidobacteria_Gp6;class;Gp6;genus	93.56	5.00E-122	438
609	0	0	1	0	0	0	0	0	1	Acidobacteria	Acidobacteria	Acidobacteriales			S000900272 Verticillium dahliae; 4S2_F08 Lineage=Root;rootrank;Bacteria;domain;"Acidobacteria";phylum;Acidobacteria_Gp6;class;Gp6;genus	97.32	5.00E-142	505
1902	0	0	0	1	0	0	0	0	0	Acidobacteria	Acidobacteria	Acidobacteriales			S000900272 Verticillium dahliae; 4S2_F08 Lineage=Root;rootrank;Bacteria;domain;"Acidobacteria";phylum;Acidobacteria_Gp6;class;Gp6;genus	96	7.00E-136	484
845	0	0	0	0	0	0	0	1	0	Acidobacteria	Acidobacteria	Acidobacteriales			S000900272 Verticillium dahliae; 4S2_F08 Lineage=Root;rootrank;Bacteria;domain;"Acidobacteria";phylum;Acidobacteria_Gp6;class;Gp6;genus	97.31	5.00E-142	505
2394	0	0	0	0	0	0	0	1	0	Acidobacteria	Acidobacteria	Acidobacteriales	Acidobacteriaceae		S002288402 Candidatus Koribacter versatilis Ellin345 Lineage=Root;rootrank;Bacteria;domain;"Acidobacteria";phylum;Acidobacteria_Gp1;class;Gp1;genus	88.24	3.00E-129	462
1100	0	0	1	0	0	0	0	0	0	Acidobacteria	Acidobacteria-5				S002350431 Edaphobacter modestus (T); Jbg-1 Lineage=Root;rootrank;Bacteria;domain;"Acidobacteria";phylum;Acidobacteria_Gp1;class;Edaphobacter;genus	86.83	1.00E-113	411
2981	0	0	0	0	1	0	0	0	0	Acidobacteria	Acidobacteria-5				S002350431 Edaphobacter modestus (T); Jbg-1 Lineage=Root;rootrank;Bacteria;domain;"Acidobacteria";phylum;Acidobacteria_Gp1;class;Edaphobacter;genus	86.41	2.00E-117	424
22	0	0	0	3	0	0	1	0	0	Acidobacteria	AT-s2-57				S000015698 Desulfovibrio indonesiensis (T) Lineage=Root;rootrank;Bacteria;domain;"Proteobacteria";phylum;Deltaproteobacteria;class;Desulfovibrionales;order;Desulfovibrionaceae;family;Desulfovibrio;genus	85.46	2.00E-111	403
934	0	0	0	0	1	0	0	0	0	Acidobacteria	AT-s54				S001933336 Candidatus Methylomirabilis oxyfera Lineage=Root;rootrank;Bacteria;domain;unclassified_Bacteria;	88.43	2.00E-131	470
3033	0	0	0	1	0	0	2	1	2	Acidobacteria	BPC02				S000434673 Thermovenabulum ferriorganovorum (T); Z-9801 Lineage=Root;rootrank;Bacteria;domain;Firmicutes;phylum;Clostridia;class;Clostridiales;order;Clostridiales_Incertae Sedis III;family;Thermovenabulum;genus	84.95	9.00E-110	398
1715	0	0	0	1	0	0	0	0	0	Acidobacteria	Chloracidobacteria				S000130885 Thermoleophilum album (T); type strain: ATCC 35263 Lineage=Root;rootrank;Bacteria;domain;"Actinobacteria";phylum;Actinobacteria;class;Rubrobacteridae;subclass;Thermoleophilales;order;Thermoleophilaceae;family;Thermoleophilum;genus	86.33	2.00E-111	403
1538	0	0	0	0	2	0	0	0	0	Acidobacteria	Chloracidobacteria				S000701761 Edaphobacter aggregans (T); Wbg-1 Lineage=Root;rootrank;Bacteria;domain;"Acidobacteria";phylum;Acidobacteria_Gp1;class;Edaphobacter;genus	82.23	3.00E-84	313
1338	0	0	3	42	42	89	53	21	19	Acidobacteria	Chloracidobacteria				S003619637 Blastocatella fastidiosa; A2-16 Lineage=Root;rootrank;Bacteria;domain;"Acidobacteria";phylum;Acidobacteria_Gp4;class;Gp4;genus	89.59	3.00E-129	462
2968	0	0	0	17	8	4	6	1	2	Acidobacteria	Chloracidobacteria				S003619637 Blastocatella fastidiosa; A2-16 Lineage=Root;rootrank;Bacteria;domain;"Acidobacteria";phylum;Acidobacteria_Gp4;class;Gp4;genus	90.88	2.00E-140	499
2240	0	0	0	7	5	0	0	0	0	Acidobacteria	Chloracidobacteria				S003619637 Blastocatella fastidiosa; A2-16 Lineage=Root;rootrank;Bacteria;domain;"Acidobacteria";phylum;Acidobacteria_Gp4;class;Gp4;genus	88.83	8.00E-125	448
2636	0	0	0	2	1	3	3	0	0	Acidobacteria	Chloracidobacteria				S003619637 Blastocatella fastidiosa; A2-16 Lineage=Root;rootrank;Bacteria;domain;"Acidobacteria";phylum;Acidobacteria_Gp4;class;Gp4;genus	89.62	8.00E-130	464
1982	0	0	0	0	1	0	0	0	0	Acidobacteria	Chloracidobacteria				S003619637 Blastocatella fastidiosa; A2-16 Lineage=Root;rootrank;Bacteria;domain;"Acidobacteria";phylum;Acidobacteria_Gp4;class;Gp4;genus	91.94	5.00E-147	521
2213	0	0	0	0	1	0	0	0	0	Acidobacteria	Chloracidobacteria				S003619637 Blastocatella fastidiosa; A2-16 Lineage=Root;rootrank;Bacteria;domain;"Acidobacteria";phylum;Acidobacteria_Gp4;class;Gp4;genus	90.08	8.00E-135	481
2766	0	0	0	0	0	0	0	0	1	Acidobacteria	Chloracidobacteria				S003619637 Blastocatella fastidiosa; A2-16 Lineage=Root;rootrank;Bacteria;domain;"Acidobacteria";phylum;Acidobacteria_Gp4;class;Gp4;genus	91.05	1.00E-148	527
1118	0	0	0	0	0	0	0	0	2	Acidobacteria	iii-8	32-20			S000437417 Desulfuromonas palmitatis (T); SDBY1 Lineage=Root;rootrank;Bacteria;domain;"Proteobacteria";phylum;Deltaproteobacteria;class;Desulfuromonadales;order;Desulfuromonadaceae;family;Desulfuromonas;genus	86.79	3.00E-114	412
1013	0	0	0	1	0	0	0	0	0	Acidobacteria	iii-8	32-20			S000437417 Desulfuromonas palmitatis (T); SDBY1 Lineage=Root;rootrank;Bacteria;domain;"Proteobacteria";phylum;Deltaproteobacteria;class;Desulfuromonadales;order;Desulfuromonadaceae;family;Desulfuromonas;genus	86.76	3.00E-114	412
259	0	0	2	20	14	3	1	3	12	Acidobacteria	iii-8	DS-8			S000262080 Pelotomaculum propionicicum (T); MGP Lineage=Root;rootrank;Bacteria;domain;Firmicutes;phylum;Clostridia;class;Clostridiales;order;Peptococcaceae 2;family;Pelotomaculum;genus	87.87	7.00E-121	435
3195	0	0	1	3	13	0	0	0	0	Acidobacteria	iii-8	DS-8			S000366775 Desulfotomaculum geothermicum; type strain: B2 Lineage=Root;rootrank;Bacteria;domain;Firmicutes;phylum;Clostridia;class;Clostridiales;order;Peptococcaceae 2;family;Desulfotomaculum;genus	87.06	2.00E-115	416
395	0	0	1	8	40	0	2	1	3	Acidobacteria	iii-8	DS-8			S000569921 Pelotomaculum isophthalicicum (T); JI Lineage=Root;rootrank;Bacteria;domain;Firmicutes;phylum;Clostridia;class;Clostridiales;order;Peptococcaceae 2;family;Pelotomaculum;genus	87.1	7.00E-116	418
1838	0	0	0	22	18	10	6	1	19	Acidobacteria	iii-8	DS-8			S002933273 Desulfotomaculum carboxydivorans CO-1-SRB Lineage=Root;rootrank;Bacteria;domain;Firmicutes;phylum;Clostridia;class;Clostridiales;order;Peptococcaceae 2;family;Desulfotomaculum;genus	88.95	1.00E-127	457
2451	0	0	0	1	1	0	0	0	0	Acidobacteria	MVS-40				S001933336 Candidatus Methylomirabilis oxyfera Lineage=Root;rootrank;Bacteria;domain;unclassified_Bacteria;	86.68	2.00E-111	403
2358	0	0	0	0	1	0	0	0	0	Acidobacteria	RB25				S000626776 Geoalkalibacter ferrihydriticus (T); Z-0531 Lineage=Root;rootrank;Bacteria;domain;"Proteobacteria";phylum;Deltaproteobacteria;class;Desulfuromonadales;order;Geobacteraceae;family;Geoalkalibacter;genus	86.96	5.00E-122	438
2143	0	0	1	6	1	0	0	0	0	Acidobacteria	RB25				S001933336 Candidatus Methylomirabilis oxyfera Lineage=Root;rootrank;Bacteria;domain;unclassified_Bacteria;	86.99	9.00E-115	414
1662	0	0	0	5	0	0	0	0	1	Acidobacteria	RB25				S001933336 Candidatus Methylomirabilis oxyfera Lineage=Root;rootrank;Bacteria;domain;unclassified_Bacteria;	87.69	7.00E-126	451
163	0	0	0	0	1	0	0	0	2	Acidobacteria	RB25				S002288637 Anaeromyxobacter dehalogenans 2CP-C Lineage=Root;rootrank;Bacteria;domain;"Proteobacteria";phylum;Deltaproteobacteria;class;Myxococcales;order;Cystobacterineae;suborder;Cystobacteraceae;family;Anaeromyxobacter;genus	88.62	2.00E-125	449
539	0	0	0	0	2	0	0	0	0	Acidobacteria	RB25				S002288637 Anaeromyxobacter dehalogenans 2CP-C Lineage=Root;rootrank;Bacteria;domain;"Proteobacteria";phylum;Deltaproteobacteria;class;Myxococcales;order;Cystobacterineae;suborder;Cystobacteraceae;family;Anaeromyxobacter;genus	89.16	4.00E-128	459
880	0	0	0	1	0	0	0	0	0	Acidobacteria	S035				S000436031 Achromatium oxaliferum; Rydal; 8 Lineage=Root;rootrank;Bacteria;domain;"Proteobacteria";phylum;Gammaproteobacteria;class;unclassified_Gammaproteobacteria;	83.72	3.00E-100	366
1658	0	0	0	0	1	0	0	0	0	Acidobacteria	Solibacteres	Solibacterales	Solibacteraceae	CandidatusSolibacter	S000040796 Actinomycetales; MC 27 Lineage=Root;rootrank;Bacteria;domain;"Acidobacteria";phylum;Acidobacteria_Gp3;class;Gp3;genus	93.57	9.00E-165	580
288	0	0	0	7	7	5	5	6	12	Acidobacteria	Solibacteres	Solibacterales	Solibacteraceae	CandidatusSolibacter	S000040899 Actinomycetales; MC 9 Lineage=Root;rootrank;Bacteria;domain;"Acidobacteria";phylum;Acidobacteria_Gp3;class;Gp3;genus	94.62	1.00E-162	573
2805	0	0	0	20	5	4	1	7	1	Acidobacteria	Solibacteres	Solibacterales	Solibacteraceae	CandidatusSolibacter	S000040899 Actinomycetales; MC 9 Lineage=Root;rootrank;Bacteria;domain;"Acidobacteria";phylum;Acidobacteria_Gp3;class;Gp3;genus	93.28	3.00E-154	545
817	0	0	2	1	5	0	1	0	1	Acidobacteria	Solibacteres	Solibacterales	Solibacteraceae	CandidatusSolibacter	S000040899 Actinomycetales; MC 9 Lineage=Root;rootrank;Bacteria;domain;"Acidobacteria";phylum;Acidobacteria_Gp3;class;Gp3;genus	92.82	2.00E-160	566
1463	0	0	2	24	19	11	8	10	20	Acidobacteria	Solibacteres	Solibacterales	Solibacteraceae	CandidatusSolibacter	S001568508 Candidatus Solibacter usitatus; P1s-321 Lineage=Root;rootrank;Bacteria;domain;"Acidobacteria";phylum;Acidobacteria_Gp3;class;Gp3;genus	91.4	1.00E-142	507
2477	0	0	0	4	3	0	0	0	0	Acidobacteria	Solibacteres	Solibacterales	Solibacteraceae	CandidatusSolibacter	S001568508 Candidatus Solibacter usitatus; P1s-321 Lineage=Root;rootrank;Bacteria;domain;"Acidobacteria";phylum;Acidobacteria_Gp3;class;Gp3;genus	91.4	1.00E-142	507
2777	0	0	1	13	4	4	4	2	3	Acidobacteria	Solibacteres	Solibacterales	Solibacteraceae	CandidatusSolibacter	S001568576 Candidatus Solibacter usitatus; P1s-370 Lineage=Root;rootrank;Bacteria;domain;"Acidobacteria";phylum;Acidobacteria_Gp3;class;Gp3;genus	90.88	3.00E-139	496
3128	0	0	0	1	1	0	0	1	2	Acidobacteria	Solibacteres	Solibacterales	Solibacteraceae	CandidatusSolibacter	S001568576 Candidatus Solibacter usitatus; P1s-370 Lineage=Root;rootrank;Bacteria;domain;"Acidobacteria";phylum;Acidobacteria_Gp3;class;Gp3;genus	93.51	8.00E-155	547
2222	0	0	0	0	0	0	0	0	2	Acidobacteria	Solibacteres	Solibacterales	Solibacteraceae	CandidatusSolibacter	S001568576 Candidatus Solibacter usitatus; P1s-370 Lineage=Root;rootrank;Bacteria;domain;"Acidobacteria";phylum;Acidobacteria_Gp3;class;Gp3;genus	88.97	9.00E-135	481
2444	0	0	0	38	9	230	167	4	18	Acidobacteria	Solibacteres	Solibacterales	Solibacteraceae	CandidatusSolibacter	S002288402 Candidatus Koribacter versatilis Ellin345 Lineage=Root;rootrank;Bacteria;domain;"Acidobacteria";phylum;Acidobacteria_Gp1;class;Gp1;genus	84.76	2.00E-100	366
866	0	0	0	0	0	0	0	0	2	Acidobacteria	Solibacteres	Solibacterales	Solibacteraceae	CandidatusSolibacter	S002288402 Candidatus Koribacter versatilis Ellin345 Lineage=Root;rootrank;Bacteria;domain;"Acidobacteria";phylum;Acidobacteria_Gp1;class;Gp1;genus	84.95	2.00E-101	370
83	0	0	0	1	0	0	0	0	0	Acidobacteria	Solibacteres	Solibacterales	Solibacteraceae	CandidatusSolibacter	S002288402 Candidatus Koribacter versatilis Ellin345 Lineage=Root;rootrank;Bacteria;domain;"Acidobacteria";phylum;Acidobacteria_Gp1;class;Gp1;genus	85.64	2.00E-112	407
3019	0	0	4	12	8	0	0	0	4	Acidobacteria	Solibacteres	Solibacterales	Solibacteraceae	CandidatusSolibacter	S002290405 Candidatus Solibacter usitatus Ellin6076 Lineage=Root;rootrank;Bacteria;domain;"Acidobacteria";phylum;Acidobacteria_Gp3;class;Gp3;genus	89.54	6.00E-131	468
1524	0	0	0	6	3	0	0	0	0	Acidobacteria	Solibacteres	Solibacterales	Solibacteraceae	CandidatusSolibacter	S002290405 Candidatus Solibacter usitatus Ellin6076 Lineage=Root;rootrank;Bacteria;domain;"Acidobacteria";phylum;Acidobacteria_Gp3;class;Gp3;genus	88.44	1.00E-123	444
483	0	0	0	0	1	1	0	1	4	Acidobacteria	Solibacteres	Solibacterales	Solibacteraceae	CandidatusSolibacter	S002351528 Terriglobus saanensis; SP1PR5 Lineage=Root;rootrank;Bacteria;domain;"Acidobacteria";phylum;Acidobacteria_Gp1;class;Terriglobus;genus	84.95	2.00E-101	370
2925	0	0	0	1	0	0	0	0	0	Acidobacteria	Sva0725	Sva0725			S000392255 Desulfuromonas michiganensis (T); BB1 Lineage=Root;rootrank;Bacteria;domain;"Proteobacteria";phylum;Deltaproteobacteria;class;Desulfuromonadales;order;Desulfuromonadaceae;family;Desulfuromonas;genus	87.53	7.00E-126	451
595	0	0	0	0	0	1	6	1	0	Acidobacteria	Sva0725	Sva0725			S000410447 Desulfomicrobium thermophilum (T); P6.2 Lineage=Root;rootrank;Bacteria;domain;"Proteobacteria";phylum;Deltaproteobacteria;class;Desulfovibrionales;order;Desulfomicrobiaceae;family;Desulfomicrobium;genus	87.43	1.00E-117	424
485	0	0	0	2	2	1	0	1	1	Acidobacteria	Sva0725	Sva0725			S000626776 Geoalkalibacter ferrihydriticus (T); Z-0531 Lineage=Root;rootrank;Bacteria;domain;"Proteobacteria";phylum;Deltaproteobacteria;class;Desulfuromonadales;order;Geobacteraceae;family;Geoalkalibacter;genus	87.3	5.00E-117	422
1149	0	0	4	70	8	0	1	1	14	Acidobacteria	Sva0725	Sva0725			S001568551 endosymbiont of Ridgeia piscesae; P1s-297 Lineage=Root;rootrank;Bacteria;domain;"Proteobacteria";phylum;unclassified_"Proteobacteria";	90.79	5.00E-147	521
1822	0	0	2	20	14	2	5	1	0	Acidobacteria	Sva0725	Sva0725			S001568551 endosymbiont of Ridgeia piscesae; P1s-297 Lineage=Root;rootrank;Bacteria;domain;"Proteobacteria";phylum;unclassified_"Proteobacteria";	89.67	6.00E-131	468
3196	0	0	12	10	6	0	0	3	1	Acidobacteria	Sva0725	Sva0725			S001568551 endosymbiont of Ridgeia piscesae; P1s-297 Lineage=Root;rootrank;Bacteria;domain;"Proteobacteria";phylum;unclassified_"Proteobacteria";	90.49	6.00E-136	484
2209	0	0	0	5	0	1	1	0	1	Acidobacteria	Sva0725	Sva0725			S001568551 endosymbiont of Ridgeia piscesae; P1s-297 Lineage=Root;rootrank;Bacteria;domain;"Proteobacteria";phylum;unclassified_"Proteobacteria";	89.4	3.00E-129	462
562	0	0	0	0	0	4	1	0	0	Acidobacteria	Sva0725	Sva0725			S001568551 endosymbiont of Ridgeia piscesae; P1s-297 Lineage=Root;rootrank;Bacteria;domain;"Proteobacteria";phylum;unclassified_"Proteobacteria";	91.3	2.00E-150	532
2549	0	0	0	0	1	0	0	0	0	Acidobacteria	Sva0725	Sva0725			S001568551 endosymbiont of Ridgeia piscesae; P1s-297 Lineage=Root;rootrank;Bacteria;domain;"Proteobacteria";phylum;unclassified_"Proteobacteria";	90.36	9.00E-145	514
2521	0	0	1	43	22	4	0	0	2	Acidobacteria	Sva0725	Sva0725			S002288637 Anaeromyxobacter dehalogenans 2CP-C Lineage=Root;rootrank;Bacteria;domain;"Proteobacteria";phylum;Deltaproteobacteria;class;Myxococcales;order;Cystobacterineae;suborder;Cystobacteraceae;family;Anaeromyxobacter;genus	87.8	2.00E-120	433
3082	0	0	0	2	26	0	0	1	3	Acidobacteria	Sva0725	Sva0725			S002350761 Desulfovibrio giganteus; type strain: DSM 4123 Lineage=Root;rootrank;Bacteria;domain;"Proteobacteria";phylum;Deltaproteobacteria;class;Desulfovibrionales;order;Desulfovibrionaceae;family;Desulfovibrio;genus	88.77	7.00E-126	451
1668	0	0	0	8	0	0	0	0	0	Acidobacteria	Sva0725	Sva0725			S002350761 Desulfovibrio giganteus; type strain: DSM 4123 Lineage=Root;rootrank;Bacteria;domain;"Proteobacteria";phylum;Deltaproteobacteria;class;Desulfovibrionales;order;Desulfovibrionaceae;family;Desulfovibrio;genus	87.67	3.00E-119	429
2492	0	0	0	1	0	0	0	0	0	Acidobacteria	Sva0725	Sva0725			S002350761 Desulfovibrio giganteus; type strain: DSM 4123 Lineage=Root;rootrank;Bacteria;domain;"Proteobacteria";phylum;Deltaproteobacteria;class;Desulfovibrionales;order;Desulfovibrionaceae;family;Desulfovibrio;genus	87.02	2.00E-121	436
2487	0	0	0	2	0	0	0	0	0	Acidobacteria					S000041020 Actinomycetales; MC 13 Lineage=Root;rootrank;Bacteria;domain;"Acidobacteria";phylum;Acidobacteria_Gp1;class;Gp1;genus	87.44	1.00E-123	444
3191	0	0	0	1	0	0	0	0	0	Acidobacteria					S002224495 Polyangium sorediatum; Pl s12 Lineage=Root;rootrank;Bacteria;domain;"Proteobacteria";phylum;Deltaproteobacteria;class;Myxococcales;order;Sorangiineae;suborder;Polyangiaceae;family;unclassified_Polyangiaceae;	89.43	5.00E-137	488
721	0	0	4	15	1	1	0	7	1	Acidobacteria					S002288231 Candidatus Desulforudis audaxviator MP104C Lineage=Root;rootrank;Bacteria;domain;Firmicutes;phylum;Clostridia;class;unclassified_Clostridia;	84.22	5.00E-97	355
710	0	0	1	10	20	61	46	16	45	Acidobacteria					S002288402 Candidatus Koribacter versatilis Ellin345 Lineage=Root;rootrank;Bacteria;domain;"Acidobacteria";phylum;Acidobacteria_Gp1;class;Gp1;genus	88.41	3.00E-124	446
1104	1	0	1	2	1	0	2	2	9	Actinobacteria	Actinobacteria				S000130885 Thermoleophilum album (T); type strain: ATCC 35263 Lineage=Root;rootrank;Bacteria;domain;"Actinobacteria";phylum;Actinobacteria;class;Rubrobacteridae;subclass;Thermoleophilales;order;Thermoleophilaceae;family;Thermoleophilum;genus	89.6	5.00E-132	472
2948	2	0	10	24	22	32	35	15	26	Actinobacteria	Actinobacteria				S002440614 Conexibacter arvalis; KV-963 Lineage=Root;rootrank;Bacteria;domain;"Actinobacteria";phylum;Actinobacteria;class;Rubrobacteridae;subclass;Solirubrobacterales;order;Conexibacteraceae;family;Conexibacter;genus	89.09	7.00E-136	484
3133	0	0	0	0	0	0	0	3	0	Actinobacteria	Actinobacteria	039-7L4			S000653281 Candidatus Streptomyces philanthi biovar gibbosus Lineage=Root;rootrank;Bacteria;domain;"Actinobacteria";phylum;Actinobacteria;class;Actinobacteridae;subclass;Actinomycetales;order;Streptomycineae;suborder;Streptomycetaceae;family;Streptomyces;genus	89.63	1.00E-132	473
1455	0	0	0	2	0	0	0	0	0	Actinobacteria	Actinobacteria	039-7L4			S000769689 Kribbella aluminosa; HKI 0480; 2423-007 Lineage=Root;rootrank;Bacteria;domain;"Actinobacteria";phylum;Actinobacteria;class;Actinobacteridae;subclass;Actinomycetales;order;Propionibacterineae;suborder;Nocardioidaceae;family;Kribbella;genus	88.77	1.00E-127	457
624	0	0	0	7	3	2	0	14	36	Actinobacteria	Actinobacteria	039-7L4			S000841843 Rubrobacter taiwanensis (T); LS-293 Lineage=Root;rootrank;Bacteria;domain;"Actinobacteria";phylum;Actinobacteria;class;Rubrobacteridae;subclass;Rubrobacterales;order;"Rubrobacterineae";suborder;Rubrobacteraceae;family;Rubrobacter;genus	88.74	5.00E-127	455
1759	0	0	0	0	0	0	0	1	0	Actinobacteria	Actinobacteria	039-7L4			S000841843 Rubrobacter taiwanensis (T); LS-293 Lineage=Root;rootrank;Bacteria;domain;"Actinobacteria";phylum;Actinobacteria;class;Rubrobacteridae;subclass;Rubrobacterales;order;"Rubrobacterineae";suborder;Rubrobacteraceae;family;Rubrobacter;genus	87.63	2.00E-120	433
2177	0	0	3	19	12	7	4	2	4	Actinobacteria	Actinobacteria	039-7L4			S001093253 Streptomyces inaequalis; NBRC 13604 Lineage=Root;rootrank;Bacteria;domain;"Actinobacteria";phylum;Actinobacteria;class;Actinobacteridae;subclass;Actinomycetales;order;Streptomycineae;suborder;Streptomycetaceae;family;Streptomyces;genus	89.07	3.00E-129	462
3237	0	0	0	0	0	0	0	5	6	Actinobacteria	Actinobacteria	039-7L4			S001243792 Streptomyces radiopugnans; HBUM174007 Lineage=Root;rootrank;Bacteria;domain;"Actinobacteria";phylum;Actinobacteria;class;Actinobacteridae;subclass;Actinomycetales;order;Streptomycineae;suborder;Streptomycetaceae;family;Streptomyces;genus	88.33	8.00E-125	448
2244	0	0	0	1	0	0	0	0	0	Actinobacteria	Actinobacteria	039-7L4			S001243905 Amycolatopsis coloradensis; HBUM173696 Lineage=Root;rootrank;Bacteria;domain;"Actinobacteria";phylum;Actinobacteria;class;Actinobacteridae;subclass;Actinomycetales;order;Pseudonocardineae;suborder;Pseudonocardiaceae;family;Amycolatopsis;genus	88.89	9.00E-135	481
2735	0	0	1	10	17	115	86	0	0	Actinobacteria	Actinobacteria	039-7L4			S002288714 Thermotoga naphthophila RKU-10 Lineage=Root;rootrank;Bacteria;domain;"Thermotogae";phylum;Thermotogae;class;Thermotogales;order;Thermotogaceae;family;Thermotoga;genus	83.2	9.00E-90	331
2430	0	0	0	0	1	0	0	0	0	Actinobacteria	Actinobacteria	039-7L4			S002440613 Conexibacter arvalis; KV-962 Lineage=Root;rootrank;Bacteria;domain;"Actinobacteria";phylum;Actinobacteria;class;Rubrobacteridae;subclass;Solirubrobacterales;order;Conexibacteraceae;family;Conexibacter;genus	84.37	1.00E-97	357
2611	0	0	0	12	2	1	3	11	4	Actinobacteria	Actinobacteria	039-7L4			S002965243 Streptomyces venezuelae; ACT1 Lineage=Root;rootrank;Bacteria;domain;"Actinobacteria";phylum;Actinobacteria;class;Actinobacteridae;subclass;Actinomycetales;order;Streptomycineae;suborder;Streptomycetaceae;family;Streptomyces;genus	88.8	5.00E-127	455
3138	0	0	0	1	0	0	0	2	0	Actinobacteria	Actinobacteria	039-7L4			S002965243 Streptomyces venezuelae; ACT1 Lineage=Root;rootrank;Bacteria;domain;"Actinobacteria";phylum;Actinobacteria;class;Actinobacteridae;subclass;Actinomycetales;order;Streptomycineae;suborder;Streptomycetaceae;family;Streptomyces;genus	89.15	3.00E-129	462
1209	0	0	2	6	3	3	1	5	6	Actinobacteria	Actinobacteria	039-7L4			S002967240 Streptomyces panacagri; DLS-65 Lineage=Root;rootrank;Bacteria;domain;"Actinobacteria";phylum;Actinobacteria;class;Actinobacteridae;subclass;Actinomycetales;order;Streptomycineae;suborder;Streptomycetaceae;family;Streptomyces;genus	89.63	1.00E-132	473
2188	0	0	0	0	0	30	32	0	0	Actinobacteria	Actinobacteria	039-7L4			S000841843 Rubrobacter taiwanensis (T); LS-293 Lineage=Root;rootrank;Bacteria;domain;"Actinobacteria";phylum;Actinobacteria;class;Rubrobacteridae;subclass;Rubrobacterales;order;"Rubrobacterineae";suborder;Rubrobacteraceae;family;Rubrobacter;genus	84.45	9.00E-100	364
2781	0	0	2	3	0	0	0	0	0	Actinobacteria	Actinobacteria	039-7L4			S000841843 Rubrobacter taiwanensis (T); LS-293 Lineage=Root;rootrank;Bacteria;domain;"Actinobacteria";phylum;Actinobacteria;class;Rubrobacteridae;subclass;Rubrobacterales;order;"Rubrobacterineae";suborder;Rubrobacteraceae;family;Rubrobacter;genus	84.68	2.00E-101	370
458	0	0	1	0	0	0	0	0	0	Actinobacteria	Actinobacteria	039-7L4			S000841843 Rubrobacter taiwanensis (T); LS-293 Lineage=Root;rootrank;Bacteria;domain;"Actinobacteria";phylum;Actinobacteria;class;Rubrobacteridae;subclass;Rubrobacterales;order;"Rubrobacterineae";suborder;Rubrobacteraceae;family;Rubrobacter;genus	84.76	2.00E-101	370
2722	0	0	0	1	0	0	0	0	0	Actinobacteria	Actinobacteria	039-7L4			S000841843 Rubrobacter taiwanensis (T); LS-293 Lineage=Root;rootrank;Bacteria;domain;"Actinobacteria";phylum;Actinobacteria;class;Rubrobacteridae;subclass;Rubrobacterales;order;"Rubrobacterineae";suborder;Rubrobacteraceae;family;Rubrobacter;genus	84.91	1.00E-108	394
2410	0	0	0	0	0	0	0	3	0	Actinobacteria	Actinobacteria	039-7L4			S001548570 Euzebya tangerina (T) Lineage=Root;rootrank;Bacteria;domain;"Actinobacteria";phylum;Actinobacteria;class;Nitriliruptoridae;subclass;Euzebyales;order;Euzebyaceae;family;Euzebya;genus	87.63	7.00E-126	451
3100	0	0	0	0	2	0	0	0	0	Actinobacteria	Actinobacteria	039-7L4			S001577994 Nocardia miyunensis Lineage=Root;rootrank;Bacteria;domain;"Actinobacteria";phylum;Actinobacteria;class;Actinobacteridae;subclass;Actinomycetales;order;Corynebacterineae;suborder;Nocardiaceae;family;Nocardia;genus	82.62	3.00E-89	329
636	0	0	2	1	1	17	9	0	8	Actinobacteria	Actinobacteria	039-7L4			S002290712 Rubrobacter xylanophilus (T); DSM 9941 Lineage=Root;rootrank;Bacteria;domain;"Actinobacteria";phylum;Actinobacteria;class;Rubrobacteridae;subclass;Rubrobacterales;order;"Rubrobacterineae";suborder;Rubrobacteraceae;family;Rubrobacter;genus	82.09	9.00E-85	315
2633	0	0	1	0	0	3	4	0	2	Actinobacteria	Actinobacteria	039-7L4			S002290712 Rubrobacter xylanophilus (T); DSM 9941 Lineage=Root;rootrank;Bacteria;domain;"Actinobacteria";phylum;Actinobacteria;class;Rubrobacteridae;subclass;Rubrobacterales;order;"Rubrobacterineae";suborder;Rubrobacteraceae;family;Rubrobacter;genus	83.16	2.00E-91	337
921	0	0	1	3	0	0	0	0	0	Actinobacteria	Actinobacteria	039-7L4			S002290712 Rubrobacter xylanophilus (T); DSM 9941 Lineage=Root;rootrank;Bacteria;domain;"Actinobacteria";phylum;Actinobacteria;class;Rubrobacteridae;subclass;Rubrobacterales;order;"Rubrobacterineae";suborder;Rubrobacteraceae;family;Rubrobacter;genus	83.69	2.00E-95	350
2239	0	0	0	1	0	0	0	0	0	Actinobacteria	Actinobacteria	039-7L4			S002290712 Rubrobacter xylanophilus (T); DSM 9941 Lineage=Root;rootrank;Bacteria;domain;"Actinobacteria";phylum;Actinobacteria;class;Rubrobacteridae;subclass;Rubrobacterales;order;"Rubrobacterineae";suborder;Rubrobacteraceae;family;Rubrobacter;genus	85.03	4.00E-103	375
2200	0	0	0	1	4	0	0	0	0	Actinobacteria	Actinobacteria	039-7L4			S002290894 Carboxydothermus hydrogenoformans (T); Z-2901 Lineage=Root;rootrank;Bacteria;domain;Firmicutes;phylum;Clostridia;class;Thermoanaerobacterales;order;Thermoanaerobacteraceae;family;Carboxydothermus;genus	83.7	1.00E-93	344
445	0	0	2	40	53	3	5	8	6	Actinobacteria	Actinobacteria	039-7L4			S002440613 Conexibacter arvalis; KV-962 Lineage=Root;rootrank;Bacteria;domain;"Actinobacteria";phylum;Actinobacteria;class;Rubrobacteridae;subclass;Solirubrobacterales;order;Conexibacteraceae;family;Conexibacter;genus	83.16	2.00E-91	337
1488	0	0	0	1	1	0	0	23	25	Actinobacteria	Actinobacteria	039-7L4			S002440613 Conexibacter arvalis; KV-962 Lineage=Root;rootrank;Bacteria;domain;"Actinobacteria";phylum;Actinobacteria;class;Rubrobacteridae;subclass;Solirubrobacterales;order;Conexibacteraceae;family;Conexibacter;genus	84.41	1.00E-97	357
307	0	0	0	0	0	0	0	1	2	Actinobacteria	Actinobacteria	039-7L4			S003287741 Rubrobacter bracarensis; CECT 7926 = VFA70612_S5 Lineage=Root;rootrank;Bacteria;domain;"Actinobacteria";phylum;Actinobacteria;class;Rubrobacteridae;subclass;Rubrobacterales;order;"Rubrobacterineae";suborder;Rubrobacteraceae;family;Rubrobacter;genus	84.95	1.00E-102	374
3040	0	0	0	2	5	1	0	0	1	Actinobacteria	Actinobacteria	039-7L4			S003610437 Pelobacter carbinolicus (T); DSM 2380 Lineage=Root;rootrank;Bacteria;domain;"Proteobacteria";phylum;Deltaproteobacteria;class;Desulfuromonadales;order;Desulfuromonadaceae;family;Pelobacter;genus	83.24	5.00E-92	339
2070	0	0	3	2	0	0	0	1	2	Actinobacteria	Actinobacteria	Acidimicrobiales			S001014085 Iamia majanohamensis (T); NBRC 102561 Lineage=Root;rootrank;Bacteria;domain;"Actinobacteria";phylum;Actinobacteria;class;Acidimicrobidae;subclass;Acidimicrobiales;order;"Acidimicrobineae";suborder;Iamiaceae;family;Iamia;genus	93.05	8.00E-155	547
1392	0	0	0	1	1	0	0	0	0	Actinobacteria	Actinobacteria	Acidimicrobiales			S001014085 Iamia majanohamensis (T); NBRC 102561 Lineage=Root;rootrank;Bacteria;domain;"Actinobacteria";phylum;Actinobacteria;class;Acidimicrobidae;subclass;Acidimicrobiales;order;"Acidimicrobineae";suborder;Iamiaceae;family;Iamia;genus	94.67	1.00E-173	610
361	0	0	0	1	0	0	0	0	0	Actinobacteria	Actinobacteria	Acidimicrobiales			S001014085 Iamia majanohamensis (T); NBRC 102561 Lineage=Root;rootrank;Bacteria;domain;"Actinobacteria";phylum;Actinobacteria;class;Acidimicrobidae;subclass;Acidimicrobiales;order;"Acidimicrobineae";suborder;Iamiaceae;family;Iamia;genus	92.55	5.00E-152	538
2134	0	0	5	148	38	0	2	7	18	Actinobacteria	Actinobacteria	Acidimicrobiales			S002164967 Aciditerrimonas ferrireducens (T); IC-180 Lineage=Root;rootrank;Bacteria;domain;"Actinobacteria";phylum;Actinobacteria;class;Acidimicrobidae;subclass;Acidimicrobiales;order;"Acidimicrobineae";suborder;"Acidimicrobineae"_incertae_sedis;family;Aciditerrimonas;genus	94.39	4.00E-163	575

2203	0	3	11	45	36	46	39	11	36	Actinobacteria	Actinobacteria	Acidimicrobiales			S002164967 Aciditerrimonas ferrireducens (T); IC-180 Lineage=Root;rootrank;Bacteria;domain;"Actinobacteria";phylum;Actinobacteria;class;Acidimicrobidae;subclass;Acidimicrobiales;order;"Acidimicrobineae";suborder;"Acidimicrobineae"_incertae_sedis;family;Aciditerrimonas;genus	93.33	2.00E-156	553
1203	5	43	8	0	0	2	1	2	3	Actinobacteria	Actinobacteria	Acidimicrobiales			S002164967 Aciditerrimonas ferrireducens (T); IC-180 Lineage=Root;rootrank;Bacteria;domain;"Actinobacteria";phylum;Actinobacteria;class;Acidimicrobidae;subclass;Acidimicrobiales;order;"Acidimicrobineae";suborder;"Acidimicrobineae"_incertae_sedis;family;Aciditerrimonas;genus	93.09	8.00E-155	547
508	1	1	3	9	11	2	12	9	15	Actinobacteria	Actinobacteria	Acidimicrobiales			S002164967 Aciditerrimonas ferrireducens (T); IC-180 Lineage=Root;rootrank;Bacteria;domain;"Actinobacteria";phylum;Actinobacteria;class;Acidimicrobidae;subclass;Acidimicrobiales;order;"Acidimicrobineae";suborder;"Acidimicrobineae"_incertae_sedis;family;Aciditerrimonas;genus	91.71	2.00E-146	520
682	0	0	1	16	5	0	0	0	6	Actinobacteria	Actinobacteria	Acidimicrobiales			S002164967 Aciditerrimonas ferrireducens (T); IC-180 Lineage=Root;rootrank;Bacteria;domain;"Actinobacteria";phylum;Actinobacteria;class;Acidimicrobidae;subclass;Acidimicrobiales;order;"Acidimicrobineae";suborder;"Acidimicrobineae"_incertae_sedis;family;Aciditerrimonas;genus	95.19	1.00E-167	590
10	0	0	1	9	6	0	1	1	3	Actinobacteria	Actinobacteria	Acidimicrobiales			S002164967 Aciditerrimonas ferrireducens (T); IC-180 Lineage=Root;rootrank;Bacteria;domain;"Actinobacteria";phylum;Actinobacteria;class;Acidimicrobidae;subclass;Acidimicrobiales;order;"Acidimicrobineae";suborder;"Acidimicrobineae"_incertae_sedis;family;Aciditerrimonas;genus	91.71	2.00E-146	520
1368	0	0	1	4	6	1	1	2	2	Actinobacteria	Actinobacteria	Acidimicrobiales			S002164967 Aciditerrimonas ferrireducens (T); IC-180 Lineage=Root;rootrank;Bacteria;domain;"Actinobacteria";phylum;Actinobacteria;class;Acidimicrobidae;subclass;Acidimicrobiales;order;"Acidimicrobineae";suborder;"Acidimicrobineae"_incertae_sedis;family;Aciditerrimonas;genus	91.25	4.00E-143	508
3118	0	0	0	4	7	0	0	0	0	Actinobacteria	Actinobacteria	Acidimicrobiales			S002164967 Aciditerrimonas ferrireducens (T); IC-180 Lineage=Root;rootrank;Bacteria;domain;"Actinobacteria";phylum;Actinobacteria;class;Acidimicrobidae;subclass;Acidimicrobiales;order;"Acidimicrobineae";suborder;"Acidimicrobineae"_incertae_sedis;family;Aciditerrimonas;genus	93.33	2.00E-156	553
667	0	0	0	8	2	0	0	0	0	Actinobacteria	Actinobacteria	Acidimicrobiales			S002164967 Aciditerrimonas ferrireducens (T); IC-180 Lineage=Root;rootrank;Bacteria;domain;"Actinobacteria";phylum;Actinobacteria;class;Acidimicrobidae;subclass;Acidimicrobiales;order;"Acidimicrobineae";suborder;"Acidimicrobineae"_incertae_sedis;family;Aciditerrimonas;genus	94.16	2.00E-170	599
885	0	0	1	3	4	0	0	0	0	Actinobacteria	Actinobacteria	Acidimicrobiales			S002164967 Aciditerrimonas ferrireducens (T); IC-180 Lineage=Root;rootrank;Bacteria;domain;"Actinobacteria";phylum;Actinobacteria;class;Acidimicrobidae;subclass;Acidimicrobiales;order;"Acidimicrobineae";suborder;"Acidimicrobineae"_incertae_sedis;family;Aciditerrimonas;genus	94.13	2.00E-161	569
843	0	0	2	0	1	0	0	4	0	Actinobacteria	Actinobacteria	Acidimicrobiales			S002164967 Aciditerrimonas ferrireducens (T); IC-180 Lineage=Root;rootrank;Bacteria;domain;"Actinobacteria";phylum;Actinobacteria;class;Acidimicrobidae;subclass;Acidimicrobiales;order;"Acidimicrobineae";suborder;"Acidimicrobineae"_incertae_sedis;family;Aciditerrimonas;genus	92.53	2.00E-151	536
2750	0	0	0	0	0	0	0	3	6	Actinobacteria	Actinobacteria	Acidimicrobiales			S002164967 Aciditerrimonas ferrireducens (T); IC-180 Lineage=Root;rootrank;Bacteria;domain;"Actinobacteria";phylum;Actinobacteria;class;Acidimicrobidae;subclass;Acidimicrobiales;order;"Acidimicrobineae";suborder;"Acidimicrobineae"_incertae_sedis;family;Aciditerrimonas;genus	95.45	3.00E-169	595
300	0	0	0	2	3	0	0	0	1	Actinobacteria	Actinobacteria	Acidimicrobiales			S002164967 Aciditerrimonas ferrireducens (T); IC-180 Lineage=Root;rootrank;Bacteria;domain;"Actinobacteria";phylum;Actinobacteria;class;Acidimicrobidae;subclass;Acidimicrobiales;order;"Acidimicrobineae";suborder;"Acidimicrobineae"_incertae_sedis;family;Aciditerrimonas;genus	91.73	2.00E-146	520
2185	0	0	0	2	1	0	0	0	0	Actinobacteria	Actinobacteria	Acidimicrobiales			S002164967 Aciditerrimonas ferrireducens (T); IC-180 Lineage=Root;rootrank;Bacteria;domain;"Actinobacteria";phylum;Actinobacteria;class;Acidimicrobidae;subclass;Acidimicrobiales;order;"Acidimicrobineae";suborder;"Acidimicrobineae"_incertae_sedis;family;Aciditerrimonas;genus	89.57	4.00E-133	475
729	0	0	0	0	0	0	0	1	3	Actinobacteria	Actinobacteria	Acidimicrobiales			S002164967 Aciditerrimonas ferrireducens (T); IC-180 Lineage=Root;rootrank;Bacteria;domain;"Actinobacteria";phylum;Actinobacteria;class;Acidimicrobidae;subclass;Acidimicrobiales;order;"Acidimicrobineae";suborder;"Acidimicrobineae"_incertae_sedis;family;Aciditerrimonas;genus	94.42	5.00E-172	604
2140	1	0	0	0	0	0	0	0	0	Actinobacteria	Actinobacteria	Acidimicrobiales			S002164967 Aciditerrimonas ferrireducens (T); IC-180 Lineage=Root;rootrank;Bacteria;domain;"Actinobacteria";phylum;Actinobacteria;class;Acidimicrobidae;subclass;Acidimicrobiales;order;"Acidimicrobineae";suborder;"Acidimicrobineae"_incertae_sedis;family;Aciditerrimonas;genus	94.92	2.00E-166	586
370	0	0	0	1	0	0	0	0	0	Actinobacteria	Actinobacteria	Acidimicrobiales			S002164967 Aciditerrimonas ferrireducens (T); IC-180 Lineage=Root;rootrank;Bacteria;domain;"Actinobacteria";phylum;Actinobacteria;class;Acidimicrobidae;subclass;Acidimicrobiales;order;"Acidimicrobineae";suborder;"Acidimicrobineae"_incertae_sedis;family;Aciditerrimonas;genus	93.15	1.00E-163	577
1746	0	1	6	20	11	15	12	8	15	Actinobacteria	Actinobacteria	Acidimicrobiales			S002222525 Ferrimicrobium acidiphilum; FeSo-D1-6-CH Lineage=Root;rootrank;Bacteria;domain;"Actinobacteria";phylum;Actinobacteria;class;Acidimicrobidae;subclass;Acidimicrobiales;order;"Acidimicrobineae";suborder;Acidimicrobiaceae;family;Ferrimicrobium;genus	92.55	2.00E-151	536
3029	0	0	2	0	0	0	1	1	0	Actinobacteria	Actinobacteria	Acidimicrobiales			S002222525 Ferrimicrobium acidiphilum; FeSo-D1-6-CH Lineage=Root;rootrank;Bacteria;domain;"Actinobacteria";phylum;Actinobacteria;class;Acidimicrobidae;subclass;Acidimicrobiales;order;"Acidimicrobineae";suborder;Acidimicrobiaceae;family;Ferrimicrobium;genus	92.27	8.00E-150	531
1025	0	0	0	0	2	0	0	0	0	Actinobacteria	Actinobacteria	Acidimicrobiales			S002222525 Ferrimicrobium acidiphilum; FeSo-D1-6-CH Lineage=Root;rootrank;Bacteria;domain;"Actinobacteria";phylum;Actinobacteria;class;Acidimicrobidae;subclass;Acidimicrobiales;order;"Acidimicrobineae";suborder;Acidimicrobiaceae;family;Ferrimicrobium;genus	92	4.00E-148	525
1394	0	0	0	0	0	10	20	0	0	Actinobacteria	Actinobacteria	Acidimicrobiales			S003315216 Iamia majanohamensis; F12 Lineage=Root;rootrank;Bacteria;domain;"Actinobacteria";phylum;Actinobacteria;class;Acidimicrobidae;subclass;Acidimicrobiales;order;"Acidimicrobineae";suborder;unclassified_"Acidimicrobineae";	93.11	8.00E-150	531
2509	0	2	9	9	14	18	21	3	2	Actinobacteria	Actinobacteria	Acidimicrobiales	AKIW874		S002164967 Aciditerrimonas ferrireducens (T); IC-180 Lineage=Root;rootrank;Bacteria;domain;"Actinobacteria";phylum;Actinobacteria;class;Acidimicrobidae;subclass;Acidimicrobiales;order;"Acidimicrobineae";suborder;"Acidimicrobineae"_incertae_sedis;family;Aciditerrimonas;genus	91.98	4.00E-148	525
3230	0	0	0	0	0	3	6	1	0	Actinobacteria	Actinobacteria	Acidimicrobiales	AKIW874		S002164967 Aciditerrimonas ferrireducens (T); IC-180 Lineage=Root;rootrank;Bacteria;domain;"Actinobacteria";phylum;Actinobacteria;class;Acidimicrobidae;subclass;Acidimicrobiales;order;"Acidimicrobineae";suborder;"Acidimicrobineae"_incertae_sedis;family;Aciditerrimonas;genus	90.91	2.00E-141	503
1383	0	0	0	0	0	1	0	0	0	Actinobacteria	Actinobacteria	Acidimicrobiales	AKIW874		S002164967 Aciditerrimonas ferrireducens (T); IC-180 Lineage=Root;rootrank;Bacteria;domain;"Actinobacteria";phylum;Actinobacteria;class;Acidimicrobidae;subclass;Acidimicrobiales;order;"Acidimicrobineae";suborder;"Acidimicrobineae"_incertae_sedis;family;Aciditerrimonas;genus	91.86	9.00E-155	547
2368	1	8	13	6	24	10	20	46	30	Actinobacteria	Actinobacteria	Acidimicrobiales	AKIW874		S003315216 Iamia majanohamensis; F12 Lineage=Root;rootrank;Bacteria;domain;"Actinobacteria";phylum;Actinobacteria;class;Acidimicrobidae;subclass;Acidimicrobiales;order;"Acidimicrobineae";suborder;unclassified_"Acidimicrobineae";	90.63	8.00E-135	481
3060	1	3	10	48	9	3	5	7	9	Actinobacteria	Actinobacteria	Acidimicrobiales	CL500-29		S001014081 Ilumatobacter fluminis (T); YM22-133 Lineage=Root;rootrank;Bacteria;domain;"Actinobacteria";phylum;Actinobacteria;class;Acidimicrobidae;subclass;Acidimicrobiales;order;"Acidimicrobineae";suborder;Acidimicrobiaceae;family;Ilumatobacter;genus	96.82	1.00E-178	627
1005	0	0	8	9	15	20	21	4	9	Actinobacteria	Actinobacteria	Acidimicrobiales	CL500-29		S001014081 Ilumatobacter fluminis (T); YM22-133 Lineage=Root;rootrank;Bacteria;domain;"Actinobacteria";phylum;Actinobacteria;class;Acidimicrobidae;subclass;Acidimicrobiales;order;"Acidimicrobineae";suborder;Acidimicrobiaceae;family;Ilumatobacter;genus	95.45	8.00E-170	597
2279	0	1	0	0	0	0	0	0	0	Actinobacteria	Actinobacteria	Acidimicrobiales	CL500-29		S001014081 Ilumatobacter fluminis (T); YM22-133 Lineage=Root;rootrank;Bacteria;domain;"Actinobacteria";phylum;Actinobacteria;class;Acidimicrobidae;subclass;Acidimicrobiales;order;"Acidimicrobineae";suborder;Acidimicrobiaceae;family;Ilumatobacter;genus	94.65	8.00E-165	580
677	0	0	0	0	0	0	1	4	1	Actinobacteria	Actinobacteria	Acidimicrobiales	CL500-29		S003710631 Ilumatobacter coccineum YM16-304 Lineage=Root;rootrank;Bacteria;domain;"Actinobacteria";phylum;Actinobacteria;class;Acidimicrobidae;subclass;Acidimicrobiales;order;"Acidimicrobineae";suborder;Acidimicrobiaceae;family;Ilumatobacter;genus	97.06	8.00E-180	630
2229	10	5	165	149	217	63	129	1	5	Actinobacteria	Actinobacteria	Acidimicrobiales	EB07		S002164967 Aciditerrimonas ferrireducens (T); IC-180 Lineage=Root;rootrank;Bacteria;domain;"Actinobacteria";phylum;Actinobacteria;class;Acidimicrobidae;subclass;Acidimicrobiales;order;"Acidimicrobineae";suborder;"Acidimicrobineae"_incertae_sedis;family;Aciditerrimonas;genus	92.8	4.00E-153	542
443	1	0	18	20	107	39	44	21	27	Actinobacteria	Actinobacteria	Acidimicrobiales	EB07		S002164967 Aciditerrimonas ferrireducens (T); IC-180 Lineage=Root;rootrank;Bacteria;domain;"Actinobacteria";phylum;Actinobacteria;class;Acidimicrobidae;subclass;Acidimicrobiales;order;"Acidimicrobineae";suborder;"Acidimicrobineae"_incertae_sedis;family;Aciditerrimonas;genus	92.8	4.00E-153	542
2839	0	0	0	5	15	16	13	22	37	Actinobacteria	Actinobacteria	Acidimicrobiales	EB07		S002164967 Aciditerrimonas ferrireducens (T); IC-180 Lineage=Root;rootrank;Bacteria;domain;"Actinobacteria";phylum;Actinobacteria;class;Acidimicrobidae;subclass;Acidimicrobiales;order;"Acidimicrobineae";suborder;"Acidimicrobineae"_incertae_sedis;family;Aciditerrimonas;genus	92.39	1.00E-158	560
985	0	1	2	4	2	1	2	8	29	Actinobacteria	Actinobacteria	Acidimicrobiales	EB07		S002164967 Aciditerrimonas ferrireducens (T); IC-180 Lineage=Root;rootrank;Bacteria;domain;"Actinobacteria";phylum;Actinobacteria;class;Acidimicrobidae;subclass;Acidimicrobiales;order;"Acidimicrobineae";suborder;"Acidimicrobineae"_incertae_sedis;family;Aciditerrimonas;genus	93.15	1.00E-163	577
3192	0	0	2	4	4	6	13	2	6	Actinobacteria	Actinobacteria	Acidimicrobiales	EB07		S002164967 Aciditerrimonas ferrireducens (T); IC-180 Lineage=Root;rootrank;Bacteria;domain;"Actinobacteria";phylum;Actinobacteria;class;Acidimicrobidae;subclass;Acidimicrobiales;order;"Acidimicrobineae";suborder;"Acidimicrobineae"_incertae_sedis;family;Aciditerrimonas;genus	91.62	1.00E-153	544
503	0	0	2	11	2	0	1	0	3	Actinobacteria	Actinobacteria	Acidimicrobiales	EB07		S002164967 Aciditerrimonas ferrireducens (T); IC-180 Lineage=Root;rootrank;Bacteria;domain;"Actinobacteria";phylum;Actinobacteria;class;Acidimicrobidae;subclass;Acidimicrobiales;order;"Acidimicrobineae";suborder;"Acidimicrobineae"_incertae_sedis;family;Aciditerrimonas;genus	92.27	8.00E-150	531
3236	0	0	2	3	1	4	4	0	1	Actinobacteria	Actinobacteria	Acidimicrobiales	EB07		S002164967 Aciditerrimonas ferrireducens (T); IC-180 Lineage=Root;rootrank;Bacteria;domain;"Actinobacteria";phylum;Actinobacteria;class;Acidimicrobidae;subclass;Acidimicrobiales;order;"Acidimicrobineae";suborder;"Acidimicrobineae"_incertae_sedis;family;Aciditerrimonas;genus	92.53	2.00E-151	536
1631	0	1	0	5	0	2	0	1	2	Actinobacteria	Actinobacteria	Acidimicrobiales	EB07		S002164967 Aciditerrimonas ferrireducens (T); IC-180 Lineage=Root;rootrank;Bacteria;domain;"Actinobacteria";phylum;Actinobacteria;class;Acidimicrobidae;subclass;Acidimicrobiales;order;"Acidimicrobineae";suborder;"Acidimicrobineae"_incertae_sedis;family;Aciditerrimonas;genus	93.33	2.00E-156	553
586	0	0	0	0	2	1	1	1	0	Actinobacteria	Actinobacteria	Acidimicrobiales	EB07		S002164967 Aciditerrimonas ferrireducens (T); IC-180 Lineage=Root;rootrank;Bacteria;domain;"Actinobacteria";phylum;Actinobacteria;class;Acidimicrobidae;subclass;Acidimicrobiales;order;"Acidimicrobineae";suborder;"Acidimicrobineae"_incertae_sedis;family;Aciditerrimonas;genus	93.07	8.00E-155	547
1730	0	0	0	0	1	0	0	0	0	Actinobacteria	Actinobacteria	Acidimicrobiales	EB07		S002164967 Aciditerrimonas ferrireducens (T); IC-180 Lineage=Root;rootrank;Bacteria;domain;"Actinobacteria";phylum;Actinobacteria;class;Acidimicrobidae;subclass;Acidimicrobiales;order;"Acidimicrobineae";suborder;"Acidimicrobineae"_incertae_sedis;family;Aciditerrimonas;genus	91.39	5.00E-152	538
800	0	0	0	1	0	0	0	0	0	Actinobacteria	Actinobacteria	Acidimicrobiales	EB07		S002164967 Aciditerrimonas ferrireducens (T); IC-180 Lineage=Root;rootrank;Bacteria;domain;"Actinobacteria";phylum;Actinobacteria;class;Acidimicrobidae;subclass;Acidimicrobiales;order;"Acidimicrobineae";suborder;"Acidimicrobineae"_incertae_sedis;family;Aciditerrimonas;genus	92.27	3.00E-149	529
2820	0	0	0	1	0	0	0	0	0	Actinobacteria	Actinobacteria	Acidimicrobiales	EB07		S002164967 Aciditerrimonas ferrireducens (T); IC-180 Lineage=Root;rootrank;Bacteria;domain;"Actinobacteria";phylum;Actinobacteria;class;Acidimicrobidae;subclass;Acidimicrobiales;order;"Acidimicrobineae";suborder;"Acidimicrobineae"_incertae_sedis;family;Aciditerrimonas;genus	91.67	3.00E-154	545
2655	0	0	0	0	0	0	0	1	0	Actinobacteria	Actinobacteria	Acidimicrobiales	EB07		S002164967 Aciditerrimonas ferrireducens (T); IC-180 Lineage=Root;rootrank;Bacteria;domain;"Actinobacteria";phylum;Actinobacteria;class;Acidimicrobidae;subclass;Acidimicrobiales;order;"Acidimicrobineae";suborder;"Acidimicrobineae"_incertae_sedis;family;Aciditerrimonas;genus	93.62	4.00E-158	558
435	106	59	424	120	175	66	100	25	71	Actinobacteria	Actinobacteria	Acidimicrobiales	EB07		S002222525 Ferrimicrobium acidiphilum; FeSo-D1-6-CH Lineage=Root;rootrank;Bacteria;domain;"Actinobacteria";phylum;Actinobacteria;class;Acidimicrobidae;subclass;Acidimicrobiales;order;"Acidimicrobineae";suborder;Acidimicrobiaceae;family;Ferrimicrobium;genus	92.02	4.00E-148	525
614	0	0	17	158	62	15	12	11	12	Actinobacteria	Actinobacteria	Acidimicrobiales	Iamiaceae	Iamia	S002164967 Aciditerrimonas ferrireducens (T); IC-180 Lineage=Root;rootrank;Bacteria;domain;"Actinobacteria";phylum;Actinobacteria;class;Acidimicrobidae;subclass;Acidimicrobiales;order;"Acidimicrobineae";suborder;"Acidimicrobineae"_incertae_sedis;family;Aciditerrimonas;genus	93.85	8.00E-160	564
1961	0	0	0	1	0	0	0	0	0	Actinobacteria	Actinobacteria	Acidimicrobiales	Microthrixaceae		S002164967 Aciditerrimonas ferrireducens (T); IC-180 Lineage=Root;rootrank;Bacteria;domain;"Actinobacteria";phylum;Actinobacteria;class;Acidimicrobidae;subclass;Acidimicrobiales;order;"Acidimicrobineae";suborder;"Acidimicrobineae"_incertae_sedis;family;Aciditerrimonas;genus	90.91	2.00E-141	503
1830	0	0	1	0	0	0	0	0	0	Actinobacteria	Actinobacteria	Acidimicrobiales			S000775888 Streptomyces vastus (T); NRRL B-12232T Lineage=Root;rootrank;Bacteria;domain;"Actinobacteria";phylum;Actinobacteria;class;Actinobacteridae;subclass;Actinomycetales;order;Streptomycineae;suborder;Streptomycetaceae;family;Streptomyces;genus	85.45	2.00E-105	383
303	0	0	1	4	0	1	0	0	1	Actinobacteria	Actinobacteria	Acidimicrobiales			S001014081 Ilumatobacter fluminis (T); YM22-133 Lineage=Root;rootrank;Bacteria;domain;"Actinobacteria";phylum;Actinobacteria;class;Acidimicrobidae;subclass;Acidimicrobiales;order;"Acidimicrobineae";suborder;Acidimicrobiaceae;family;Ilumatobacter;genus	90.93	2.00E-141	503
2421	0	0	0	1	0	0	0	0	0	Actinobacteria	Actinobacteria	Acidimicrobiales			S001014081 Ilumatobacter fluminis (T); YM22-133 Lineage=Root;rootrank;Bacteria;domain;"Actinobacteria";phylum;Actinobacteria;class;Acidimicrobidae;subclass;Acidimicrobiales;order;"Acidimicrobineae";suborder;Acidimicrobiaceae;family;Ilumatobacter;genus	93.57	1.00E-157	556
590	2	2	44	33	75	40	51	9	14	Actinobacteria	Actinobacteria	Acidimicrobiales			S001014085 Iamia majanohamensis (T); NBRC 102561 Lineage=Root;rootrank;Bacteria;domain;"Actinobacteria";phylum;Actinobacteria;class;Acidimicrobidae;subclass;Acidimicrobiales;order;"Acidimicrobineae";suborder;Iamiaceae;family;Iamia;genus	93.35	2.00E-156	553
2021	0	4	3	2	5	0	5	1	5	Actinobacteria	Actinobacteria	Acidimicrobiales			S001014085 Iamia majanohamensis (T); NBRC 102561 Lineage=Root;rootrank;Bacteria;domain;"Actinobacteria";phylum;Actinobacteria;class;Acidimicrobidae;subclass;Acidimicrobiales;order;"Acidimicrobineae";suborder;Iamiaceae;family;Iamia;genus	92.8	4.00E-153	542
2154	0	0	3	5	1	1	1	1	3	Actinobacteria	Actinobacteria	Acidimicrobiales			S001014085 Iamia majanohamensis (T); NBRC 102561 Lineage=Root;rootrank;Bacteria;domain;"Actinobacteria";phylum;Actinobacteria;class;Acidimicrobidae;subclass;Acidimicrobiales;order;"Acidimicrobineae";suborder;Iamiaceae;family;Iamia;genus	92.51	2.00E-151	536
1905	0	0	4	0	0	1	1	0	0	Actinobacteria	Actinobacteria	Acidimicrobiales			S001014085 Iamia majanohamensis (T); NBRC 102561 Lineage=Root;rootrank;Bacteria;domain;"Actinobacteria";phylum;Actinobacteria;class;Acidimicrobidae;subclass;Acidimicrobiales;order;"Acidimicrobineae";suborder;Iamiaceae;family;Iamia;genus	93.58	4.00E-158	558
2087	0	0	1	0	0	0	0	0	0	Actinobacteria	Actinobacteria	Acidimicrobiales			S001014085 Iamia majanohamensis (T); NBRC 102561 Lineage=Root;rootrank;Bacteria;domain;"Actinobacteria";phylum;Actinobacteria;class;Acidimicrobidae;subclass;Acidimicrobiales;order;"Acidimicrobineae";suborder;Iamiaceae;family;Iamia;genus	93.87	8.00E-160	564
2224	0	0	1	1	6	28	34	2	4	Actinobacteria	Actinobacteria	Acidimicrobiales			S002164967 Aciditerrimonas ferrireducens (T); IC-180 Lineage=Root;rootrank;Bacteria;domain;"Actinobacteria";phylum;Actinobacteria;class;Acidimicrobidae;subclass;Acidimicrobiales;order;"Acidimicrobineae";suborder;"Acidimicrobineae"_incertae_sedis;family;Aciditerrimonas;genus	93.05	3.00E-154	545
57	0	0	2	34	28	0	0	0	0	Actinobacteria	Actinobacteria	Acidimicrobiales			S002164967 Aciditerrimonas ferrireducens (T); IC-180 Lineage=Root;rootrank;Bacteria;domain;"Actinobacteria";phylum;Actinobacteria;class;Acidimicrobidae;subclass;Acidimicrobiales;order;"Acidimicrobineae";suborder;"Acidimicrobineae"_incertae_sedis;family;Aciditerrimonas;genus	92.25	3.00E-149	529
2204	4	11	6	4	3	10	13	4	3	Actinobacteria	Actinobacteria	Acidimicrobiales			S002164967 Aciditerrimonas ferrireducens (T); IC-180 Lineage=Root;rootrank;Bacteria;domain;"Actinobacteria";phylum;Actinobacteria;class;Acidimicrobidae;subclass;Acidimicrobiales;order;"Acidimicrobineae";suborder;"Acidimicrobineae"_incertae_sedis;family;Aciditerrimonas;genus	93.88	8.00E-160	564
2207	0	1	4	1	14	17	10	1	4	Actinobacteria	Actinobacteria	Acidimicrobiales			S002164967 Aciditerrimonas ferrireducens (T); IC-180 Lineage=Root;rootrank;Bacteria;domain;"Actinobacteria";phylum;Actinobacteria;class;Acidimicrobidae;subclass;Acidimicrobiales;order;"Acidimicrobineae";suborder;"Acidimicrobineae"_incertae_sedis;family;Aciditerrimonas;genus	92.8	4.00E-153	542
1697	0	0	0	21	4	4	3	2	8	Actinobacteria	Actinobacteria	Acidimicrobiales			S002164967 Aciditerrimonas ferrireducens (T); IC-180 Lineage=Root;rootrank;Bacteria;domain;"Actinobacteria";phylum;Actinobacteria;class;Acidimicrobidae;subclass;Acidimicrobiales;order;"Acidimicrobineae";suborder;"Acidimicrobineae"_incertae_sedis;family;Aciditerrimonas;genus	91.44	8.00E-145	514
2685	0	11	0	0	0	0	1	0	0	Actinobacteria	Actinobacteria	Acidimicrobiales			S002164967 Aciditerrimonas ferrireducens (T); IC-180 Lineage=Root;rootrank;Bacteria;domain;"Actinobacteria";phylum;Actinobacteria;class;Acidimicrobidae;subclass;Acidimicrobiales;order;"Acidimicrobineae";suborder;"Acidimicrobineae"_incertae_sedis;family;Aciditerrimonas;genus	91.18	1.00E-142	507
2715	0	0	0	2	5	0	0	0	0	Actinobacteria	Actinobacteria	Acidimicrobiales			S002164967 Aciditerrimonas ferrireducens (T); IC-180 Lineage=Root;rootrank;Bacteria;domain;"Actinobacteria";phylum;Actinobacteria;class;Acidimicrobidae;subclass;Acidimicrobiales;order;"Acidimicrobineae";suborder;"Acidimicrobineae"_incertae_sedis;family;Aciditerrimonas;genus	91.37	2.00E-151	536
1545	0	0	0	4	1	0	0	1	0	Actinobacteria	Actinobacteria	Acidimicrobiales			S002164967 Aciditerrimonas ferrireducens (T); IC-180 Lineage=Root;rootrank;Bacteria;domain;"Actinobacteria";phylum;Actinobacteria;class;Acidimicrobidae;subclass;Acidimicrobiales;order;"Acidimicrobineae";suborder;"Acidimicrobineae"_incertae_sedis;family;Aciditerrimonas;genus	90.11	2.00E-136	486
1653	0	0	0	2	0	2	0	0	0	Actinobacteria	Actinobacteria	Acidimicrobiales			S002164967 Aciditerrimonas ferrireducens (T); IC-180 Lineage=Root;rootrank;Bacteria;domain;"Actinobacteria";phylum;Actinobacteria;class;Acidimicrobidae;subclass;Acidimicrobiales;order;"Acidimicrobineae";suborder;"Acidimicrobineae"_incertae_sedis;family;Aciditerrimonas;genus	93.58	4.00E-158	558
267	0	0	0	0	0	0	1	1	0	Actinobacteria	Actinobacteria	Acidimicrobiales			S002164967 Aciditerrimonas ferrireducens (T); IC-180 Lineage=Root;rootrank;Bacteria;domain;"Actinobacteria";phylum;Actinobacteria;class;Acidimicrobidae;subclass;Acidimicrobiales;order;"Acidimicrobineae";suborder;"Acidimicrobineae"_incertae_sedis;family;Aciditerrimonas;genus	91.88	2.00E-155	549
513	0	0	0	0	1	0	0	0	0	Actinobacteria	Actinobacteria	Acidimicrobiales			S002164967 Aciditerrimonas ferrireducens (T); IC-180 Lineage=Root;rootrank;Bacteria;domain;"Actinobacteria";phylum;Actinobacteria;class;Acidimicrobidae;subclass;Acidimicrobiales;order;"Acidimicrobineae";suborder;"Acidimicrobineae"_incertae_sedis;family;Aciditerrimonas;genus	91.88	2.00E-155	549
2640	0	0	0	1	0	0	0	0	0	Actinobacteria	Actinobacteria	Acidimicrobiales			S002164967 Aciditerrimonas ferrireducens (T); IC-180 Lineage=Root;rootrank;Bacteria;domain;"Actinobacteria";phylum;Actinobacteria;class;Acidimicrobidae;subclass;Acidimicrobiales;order;"Acidimicrobineae";suborder;"Acidimicrobineae"_incertae_sedis;family;Aciditerrimonas;genus	93.68	8.00E-160	564
2854	0	0	0	1	0	0	0	0	0	Actinobacteria	Actinobacteria	Acidimicrobiales			S002164967 Aciditerrimonas ferrireducens (T); IC-180 Lineage=Root;rootrank;Bacteria;domain;"Actinobacteria";phylum;Actinobacteria;class;Acidimicrobidae;subclass;Acidimicrobiales;order;"Acidimicrobineae";suborder;"Acidimicrobineae"_incertae_sedis;family;Aciditerrimonas;genus	88.11	5.00E-122	438
1300	0	0	0	0	0	0	0	1	0	Actinobacteria	Actinobacteria	Acidimicrobiales			S002164967 Aciditerrimonas ferrireducens (T); IC-180 Lineage=Root;rootrank;Bacteria;domain;"Actinobacteria";phylum;Actinobacteria;class;Acidimicrobidae;subclass;Acidimicrobiales;order;"Acidimicrobineae";suborder;"Acidimicrobineae"_incertae_sedis;family;Aciditerrimonas;genus	91.88	2.00E-155	549
2546	0	0	1	0	0	7	3	1	2	Actinobacteria	Actinobacteria	Acidimicrobiales			S002222525 Ferrimicrobium acidiphilum; FeSo-D1-6-CH Lineage=Root;rootrank;Bacteria;domain;"Actinobacteria";phylum;Actinobacteria;class;Acidimicrobidae;subclass;Acidimicrobiales;order;"Acidimicrobineae";suborder;Acidimicrobiaceae;family;Ferrimicrobium;genus	93.33	2.00E-156	553
635	0	1	0	2	1	2	0	0	0	Actinobacteria	Actinobacteria	Acidimicrobiales			S002222525 Ferrimicrobium acidiphilum; FeSo-D1-6-CH Lineage=Root;rootrank;Bacteria;domain;"Actinobacteria";phylum;Actinobacteria;class;Acidimicrobidae;subclass;Acidimicrobiales;order;"Acidimicrobineae";suborder;Acidimicrobiaceae;family;Ferrimicrobium;genus	91.73	2.00E-146	520
2743	0	0	0	0	0	0	0	0	1	Actinobacteria	Actinobacteria	Acidimicrobiales			S002222525 Ferrimicrobium acidiphilum; FeSo-D1-6-CH Lineage=Root;rootrank;Bacteria;domain;"Actinobacteria";phylum;Actinobacteria;class;Acidimicrobidae;subclass;Acidimicrobiales;order;"Acidimicrobineae";suborder;Acidimicrobiaceae;family;Ferrimicrobium;genus	91.14	2.00E-150	532
1677	0	0	1	21	21	3	3	2	3	Actinobacteria	Actinobacteria	Acidimicrobiales			S002288218 Acidimicrobium ferrooxidans DSM 10331 Lineage=Root;rootrank;Bacteria;domain;"Actinobacteria";phylum;Actinobacteria;class;Acidimicrobidae;subclass;Acidimicrobiales;order;"Acidimicrobineae";suborder;Acidimicrobiaceae;family;Acidimicrobium;genus	91.25	4.00E-143	508
2614	0	0	0	0	0	0	1	0	0	Actinobacteria	Actinobacteria	Acidimicrobiales			S002290453 Nitrosococcus watsoni C-113 Lineage=Root;rootrank;Bacteria;domain;"Proteobacteria";phylum;Gammaproteobacteria;class;Chromatiales;order;Chromatiaceae;family;Nitrosococcus;genus	86.04	9.00E-115	414
1063	0	0	0	0	0	0	0	1	2	Actinobacteria	Actinobacteria	Acidimicrobiales			S003315216 Iamia majanohamensis; F12 Lineage=Root;rootrank;Bacteria;domain;"Actinobacteria";phylum;Actinobacteria;class;Acidimicrobidae;subclass;Acidimicrobiales;order;"Acidimicrobineae";suborder;unclassified_"Acidimicrobineae";	93.94	8.00E-155	547
1034	0	0	0	0	0	1	0	0	0	Actinobacteria	Actinobacteria	Acidimicrobiales			S003315216 Iamia majanohamensis; F12 Lineage=Root;rootrank;Bacteria;domain;"Actinobacteria";phylum;Actinobacteria;class;Acidimicrobidae;subclass;Acidimicrobiales;order;"Acidimicrobineae";suborder;unclassified_"Acidimicrobineae";	90.63	8.00E-135	481
2708	0	0	0	0	0	0	0	0	1	Actinobacteria	Actinobacteria	Acidimicrobiales			S003315216 Iamia majanohamensis; F12 Lineage=Root;rootrank;Bacteria;domain;"Actinobacteria";phylum;Actinobacteria;class;Acidimicrobidae;subclass;Acidimicrobiales;order;"Acidimicrobineae";suborder;unclassified_"Acidimicrobineae";	88.69	1.00E-93	344
3091	0	0	0	1	0	0	0	0	0	Actinobacteria	Actinobacteria	Acidimicrobiales			S003315233 Ilumatobacter fluminis; YM22-133 Lineage=Root;rootrank;Bacteria;domain;"Actinobacteria";phylum;Actinobacteria;class;Acidimicrobidae;subclass;Acidimicrobiales;order;"Acidimicrobineae";suborder;Acidimicrobiaceae;family;Ilumatobacter;genus	92.33	2.00E-145	516
1952	0	0	0	1	0	0	0	0	0	Actinobacteria	Actinobacteria	Actinomycetales			S000429095 Crossiella cryophila (T); NRRL B-16238 Lineage=Root;rootrank;Bacteria;domain;"Actinobacteria";phylum;Actinobacteria;class;Actinobacteridae;subclass;Actinomycetales;order;Pseudonocardineae;suborder;Pseudonocardiaceae;family;Crossiella;genus	95.99	1.00E-172	606
2149	0	0	2	17	71	1	0	0	0	Actinobacteria	Actinobacteria	Actinomycetales			S003305356 Modestobacter multiseptatus; CI1-39 Lineage=Root;rootrank;Bacteria;domain;"Actinobacteria";phylum;Actinobacteria;class;Actinobacteridae;subclass;Actinomycetales;order;Frankineae;suborder;Geodermatophilaceae;family;Modestobacter;genus	94.67	4.00E-173	608
2044	0	0	0	0	0	0	0	0	1	Actinobacteria	Actinobacteria	Actinomycetales	Actinopolysporaceae		S000925481 Amycolatopsis taiwanensis; TT 99-52 (= NBRC 103393) Lineage=Root;rootrank;Bacteria;domain;"Actinobacteria";phylum;Actinobacteria;class;Actinobacteridae;subclass;Actinomycetales;order;Pseudonocardineae;suborder;Pseudonocardiaceae;family;Amycolatopsis;genus	92.59	5.00E-152	538
1683	0	2	0	0	0	0	0	0	0	Actinobacteria	Actinobacteria	Actinomycetales	Actinopolysporaceae	Actinopolyspora	S003721284 Pseudoclavibacter bifida; HHZL-00027 Lineage=Root;rootrank;Bacteria;domain;"Actinobacteria";phylum;Actinobacteria;class;Actinobacteridae;subclass;Actinomycetales;order;Micrococcineae;suborder;Microbacteriaceae;family;Zimmermannella;genus	90.59	2.00E-146	520
3114	0	0	0	8	4	0	0	0	0	Actinobacteria	Actinobacteria	Actinomycetales	Actinosynnemataceae		S003259590 Lentzea flaviverrucosa; DSS10 Lineage=Root;rootrank;Bacteria;domain;"Actinobacteria";phylum;Actinobacteria;class;Actinobacteridae;subclass;Actinomycetales;order;Pseudonocardineae;suborder;Pseudonocardiaceae;family;Lentzea;genus	99.49	0	712
319	0	27	23	49	14	20	23	5	96	Actinobacteria	Actinobacteria	Actinomycetales	Actinosynnemataceae	Actinokineospora	S001020079 Alloactinosynnema album (T); 03-9939 Lineage=Root;rootrank;Bacteria;domain;"Actinobacteria";phylum;Actinobacteria;class;Actinobacteridae;subclass;Actinomycetales;order;Pseudonocardineae;suborder;Pseudonocardiaceae;family;Alloactinosynnema;genus	97.86	0	643
531	0	0	0	2	3	0	1	0	0	Actinobacteria	Actinobacteria	Actinomycetales	Actinosynnemataceae		S003283408 actinomycete A4 Lineage=Root;rootrank;Bacteria;domain;"Actinobacteria";phylum;Actinobacteria;class;Actinobacteridae;subclass;Actinomycetales;order;Pseudonocardineae;suborder;Pseudonocardiaceae;family;Saccharothrix;genus	98.39	0	654
487	0	2	0	1	1	9	4	1	11	Actinobacteria	Actinobacteria	Actinomycetales	Brevibacteriaceae	Brevibacterium	S002034453 Nesterenkonia sandarakina; M1S6-15 Lineage=Root;rootrank;Bacteria;domain;"Actinobacteria";phylum;Actinobacteria;class;Actinobacteridae;subclass;Actinomycetales;order;Micrococcineae;suborder;Micrococcaceae;family;Nesterenkonia;genus	93.3	2.00E-155	549
2245	0	0	0	0	0	0	0	0	1	Actinobacteria	Actinobacteria	Actinomycetales	Brevibacteriaceae	Brevibacterium	S002287649 Beutenbergia cavernae DSM 12333 Lineage=Root;rootrank;Bacteria;domain;"Actinobacteria";phylum;Actinobacteria;class;Actinobacteridae;subclass;Actinomycetales;order;Micrococcineae;suborder;Beutenbergiaceae;family;Beutenbergia;genus	95.71	6.00E-171	601
988	0	0	0	0	0	0	0	1	0	Actinobacteria	Actinobacteria	Actinomycetales	Cellulomonadaceae	Demequina	S002949604 Demequina aurantiaca; NBRC 106265 Lineage=Root;rootrank;Bacteria;domain;"Actinobacteria";phylum;Actinobacteria;class;Actinobacteridae;subclass;Actinomycetales;order;Micrococcineae;suborder;Demequinaceae;family;Demequina;genus	98.73	0	697
3099	0	1	0	0	0	0	0	0	0	Actinobacteria	Actinobacteria	Actinomycetales	Corynebacteriaceae	Corynebacterium	S001294708 Dietzia aerolata (T); type strain: Sj14a Lineage=Root;rootrank;Bacteria;domain;"Actinobacteria";phylum;Actinobacteria;class;Actinobacteridae;subclass;Actinomycetales;order;Corynebacterineae;suborder;Dietziaceae;family;Dietzia;genus	94.15	9.00E-170	597
2315	0	4	2	1	1	3	1	1	1	Actinobacteria	Actinobacteria	Actinomycetales	Dermabacteraceae	Brachybacterium	S001328309 Helcobacillus massiliensis (T); 6401990 Lineage=Root;rootrank;Bacteria;domain;"Actinobacteria";phylum;Actinobacteria;class;Actinobacteridae;subclass;Actinomycetales;order;Micrococcineae;suborder;Dermabacteraceae;family;Helcobacillus;genus	97.59	0	640
1584	0	0	0	0	0	0	1	0	0	Actinobacteria	Actinobacteria	Actinomycetales	Dietziaceae	Dietzia	S003719262 Dietzia maris; 4BN03U-1 Lineage=Root;rootrank;Bacteria;domain;"Actinobacteria";phylum;Actinobacteria;class;Actinobacteridae;subclass;Actinomycetales;order;Corynebacterineae;suborder;Dietziaceae;family;Dietzia;genus	100	0	688
2053	0	1	0	0	0	0	0	0	0	Actinobacteria	Actinobacteria	Actinomycetales	Frankiaceae	Frankia	S003305348 Blastococcus saxobsidens; DD13 Lineage=Root;rootrank;Bacteria;domain;"Actinobacteria";phylum;Actinobacteria;class;Actinobacteridae;subclass;Actinomycetales;order;Frankineae;suborder;Geodermatophilaceae;family;Blastococcus;genus	99.73	0	684
3059	0	0	2	3	3	0	0	0	0	Actinobacteria	Actinobacteria	Actinomycetales	Geodermatophilaceae		S003618516 Blastococcus aggregatus; CB-281465 Lineage=Root;rootrank;Bacteria;domain;"Actinobacteria";phylum;Actinobacteria;class;Actinobacteridae;subclass;Actinomycetales;order;Frankineae;suborder;Geodermatophilaceae;family;Blastococcus;genus	100	0	689
2810	0	0	1	9	1	0	0	2	0	Actinobacteria	Actinobacteria	Actinomycetales	Geodermatophilaceae	Geodermatophilus	S003305341 Blastococcus saxobsidens; CI1-1 Lineage=Root;rootrank;Bacteria;domain;"Actinobacteria";phylum;Actinobacteria;class;Actinobacteridae;subclass;Actinomycetales;order;Frankineae;suborder;Geodermatophilaceae;family;Blastococcus;genus	100	0	689
2471	0	0	0	6	0	1	4	6	3	Actinobacteria	Actinobacteria	Actinomycetales	Glycomycetaceae	Glycomyces	S000485491 Glycomyces arizonensis (T); NRRL B-16153 Lineage=Root;rootrank;Bacteria;domain;"Actinobacteria";phylum;Actinobacteria;class;Actinobacteridae;subclass;Actinomycetales;order;Glycomycineae;suborder;Glycomycetaceae;family;Glycomyces;genus	99.2	0	673
1856	0	0	0	2	0	1	3	2	3	Actinobacteria	Actinobacteria	Actinomycetales	Glycomycetaceae	Glycomyces	S001243981 Glycomyces harbinensis; HBUM174695 Lineage=Root;rootrank;Bacteria;domain;"Actinobacteria";phylum;Actinobacteria;class;Actinobacteridae;subclass;Actinomycetales;order;Glycomycineae;suborder;Glycomycetaceae;family;Glycomyces;genus	99.73	0	684
2936	0	7	0	0	0	1	0	0	0	Actinobacteria	Actinobacteria	Actinomycetales	Glycomycetaceae	Glycomyces	S001243981 Glycomyces harbinensis; HBUM174695 Lineage=Root;rootrank;Bacteria;domain;"Actinobacteria";phylum;Actinobacteria;class;Actinobacteridae;subclass;Actinomycetales;order;Glycomycineae;suborder;Glycomycetaceae;family;Glycomyces;genus	95.44	4.00E-168	592
805	0	0	31	20	69	0	1	16	42	Actinobacteria	Actinobacteria	Actinomycetales	Kineosporiaceae		S000504795 Cellulomonas denverensis; W6117 Lineage=Root;rootrank;Bacteria;domain;"Actinobacteria";phylum;Actinobacteria;class;Actinobacteridae;subclass;Actinomycetales;order;Micrococcineae;suborder;Cellulomonadaceae;family;Cellulomonas;genus	96.78	1.00E-177	623
1214	0	0	0	2	8	2	0	0	6	Actinobacteria	Actinobacteria	Actinomycetales	Microbacteriaceae		S001151882 Glaciibacter superstes (T); AHU1791 Lineage=Root;rootrank;Bacteria;domain;"Actinobacteria";phylum;Actinobacteria;class;Actinobacteridae;subclass;Actinomycetales;order;Micrococcineae;suborder;Microbacteriaceae;family;Glaciibacter;genus	100	0	689
2624	0	0	4	22	5	0	1	4	0	Actinobacteria	Actinobacteria	Actinomycetales	Microbacteriaceae	Agromyces	S002449661 Agromyces ramosus; HRT25 Lineage=Root;rootrank;Bacteria;domain;"Actinobacteria";phylum;Actinobacteria;class;Actinobacteridae;subclass;Actinomycetales;order;Micrococcineae;suborder;Microbacteriaceae;family;Agromyces;genus	100	0	689
912	0	0	0	0	0	0	0	0	1	Actinobacteria	Actinobacteria	Actinomycetales	Microbacteriaceae	Leucobacter	S001264950 Leucobacter komagatae; tu17-2 Lineage=Root;rootrank;Bacteria;domain;"Actinobacteria";phylum;Actinobacteria;class;Actinobacteridae;subclass;Actinomycetales;order;Micrococcineae;suborder;Microbacteriaceae;family;Leucobacter;genus	98.47	0	691
1656	0	0	0	2	3	0	0	0	1	Actinobacteria	Actinobacteria	Actinomycetales	Microbacteriaceae	Leucobacter	S003616126 Leucobacter tardus; IARI-S-23 Lineage=Root;rootrank;Bacteria;domain;"Actinobacteria";phylum;Actinobacteria;class;Actinobacteridae;subclass;Actinomycetales;order;Micrococcineae;suborder;Microbacteriaceae;family;Leucobacter;genus	98.38	0	651
2467	1	0	2	2	2	7	3	3	9	Actinobacteria	Actinobacteria	Actinomycetales	Microbacteriaceae	Microbacterium	S002448082 Mycetocola reblochoni; WA4-11 Lineage=Root;rootrank;Bacteria;domain;"Actinobacteria";phylum;Actinobacteria;class;Actinobacteridae;subclass;Actinomycetales;order;Micrococcineae;suborder;Microbacteriaceae;family;unclassified_Microbacteriaceae;	97.59	0	640
1950	0	0	9	73	26	2	0	2	1	Actinobacteria	Actinobacteria	Actinomycetales	Microbacteriaceae		S000610461 Leifsonia rubra; SE44 Lineage=Root;rootrank;Bacteria;domain;"Actinobacteria";phylum;Actinobacteria;class;Actinobacteridae;subclass;Actinomycetales;order;Micrococcineae;suborder;Microbacteriaceae;family;unclassified_Microbacteriaceae;	98.72	0	697
2016	0	0	0	1	0	0	0	0	0	Actinobacteria	Actinobacteria	Actinomycetales	Microbacteriaceae		S001906239 Olsenella profusa; WWC_C5AKM122 Lineage=Root;rootrank;Bacteria;domain;"Actinobacteria";phylum;Actinobacteria;class;Coriobacteridae;subclass;Coriobacteriales;order;"Coriobacterineae";suborder;Coriobacteriaceae;family;Olsenella;genus	77.15	3.00E-54	213
1172	18	1	0	0	0	3	6	0	0	Actinobacteria	Actinobacteria	Actinomycetales	Microbacteriaceae		S002442625 Leucobacter tardus; 5-C-1 Lineage=Root;rootrank;Bacteria;domain;"Actinobacteria";phylum;Actinobacteria;class;Actinobacteridae;subclass;Actinomycetales;order;Micrococcineae;suborder;Microbacteriaceae;family;Leucobacter;genus	97.06	3.00E-179	628
1141	0	4	2	33	8	4	2	7	5	Actinobacteria	Actinobacteria	Actinomycetales	Micrococcaceae	Arthrobacter	S002352232 Arthrobacter sulfureus; BZ73 Lineage=Root;rootrank;Bacteria;domain;"Actinobacteria";phylum;Actinobacteria;class;Actinobacteridae;subclass;Actinomycetales;order;Micrococcineae;suborder;Micrococcaceae;family;Arthrobacter;genus	99.74	0	719
1376	0	4	1	0	8	2	8	3	10	Actinobacteria	Actinobacteria	Actinomycetales	Micrococcaceae	Arthrobacter	S003752102 Arthrobacter nicotianae; Cl-19 Lineage=Root;rootrank;Bacteria;domain;"Actinobacteria";phylum;Actinobacteria;class;Actinobacteridae;subclass;Actinomycetales;order;Micrococcineae;suborder;Micrococcaceae;family;Arthrobacter;genus	100	0	725
2662	0	7	6	0	1	3	2	1	1	Actinobacteria	Actinobacteria	Actinomycetales	Micrococcaceae	Nesterenkonia	S001098707 Nesterenkonia halobia; REG13 Lineage=Root;rootrank;Bacteria;domain;"Actinobacteria";phylum;Actinobacteria;class;Actinobacteridae;subclass;Actinomycetales;order;Micrococcineae;suborder;Micrococcaceae;family;Nesterenkonia;genus	100	0	689
2511	0	0	0	1	0	0	0	0	0	Actinobacteria	Actinobacteria	Actinomycetales	Micrococcaceae		S003745355 Arthrobacter crystallopoietes; Tibetlhz-04 Lineage=Root;rootrank;Bacteria;domain;"Actinobacteria";phylum;Actinobacteria;class;Actinobacteridae;subclass;Actinomycetales;order;Micrococcineae;suborder;Micrococcaceae;family;Arthrobacter;genus	98.98	0	704
62	0	0	0	0	0	1	0	0	0	Actinobacteria	Actinobacteria	Actinomycetales	Micromonosporaceae		S003305348 Blastococcus saxobsidens; DD13 Lineage=Root;rootrank;Bacteria;domain;"Actinobacteria";phylum;Actinobacteria;class;Actinobacteridae;subclass;Actinomycetales;order;Frankineae;suborder;Geodermatophilaceae;family;Blastococcus;genus	95.19	1.00E-167	590
2999	0	0	0	7	2	0	3	1	0	Actinobacteria	Actinobacteria	Actinomycetales	Micromonosporaceae	Catellatospora	S000925478 Amycolatopsis sacchari; NBRC 100339 Lineage=Root;rootrank;Bacteria;domain;"Actinobacteria";phylum;Actinobacteria;class;Actinobacteridae;subclass;Actinomycetales;order;Pseudonocardineae;suborder;Pseudonocardiaceae;family;Amycolatopsis;genus	94.13	2.00E-160	566
1184	4	0	0	0	0	1	0	0	0	Actinobacteria	Actinobacteria	Actinomycetales	Micromonosporaceae		S000978796 Goodfellowia coeruleoviolacea; NBRC 14988 Lineage=Root;rootrank;Bacteria;domain;"Actinobacteria";phylum;Actinobacteria;class;Actinobacteridae;subclass;Actinomycetales;order;Pseudonocardineae;suborder;Pseudonocardiaceae;family;Goodfellowiella;genus	93.88	3.00E-159	562
1575	20	47	34	34	27	28	52	6	6	Actinobacteria	Actinobacteria	Actinomycetales	Mycobacteriaceae	Mycobacterium	S002933346 Amycolicicoccus subflavus DQS3-9A1 Lineage=Root;rootrank;Bacteria;domain;"Actinobacteria";phylum;Actinobacteria;class;Actinobacteridae;subclass;Actinomycetales;order;Corynebacterineae;suborder;Mycobacteriaceae;family;Amycolicicoccus;genus	96.51	2.00E-175	616
2403	0	0	0	0	0	0	0	2	0	Actinobacteria	Actinobacteria	Actinomycetales	Nocardiaceae	Nocardia	S002916894 Nocardia carnea; 166207 Lineage=Root;rootrank;Bacteria;domain;"Actinobacteria";phylum;Actinobacteria;class;Actinobacteridae;subclass;Actinomycetales;order;Corynebacterineae;suborder;Nocardiaceae;family;Nocardia;genus	98.66	0	662
2577	0	0	0	0	0	1	0	1	0	Actinobacteria	Actinobacteria	Actinomycetales	Nocardiaceae	Rhodococcus	S000384429 Rhodococcus tukisamuensis (T); Mb8 Lineage=Root;rootrank;Bacteria;domain;"Actinobacteria";phylum;Actinobacteria;class;Actinobacteridae;subclass;Actinomycetales;order;Corynebacterineae;suborder;Nocardiaceae;family;Rhodococcus;genus	99.23	0	704
1164	0	0	0	0	3	0	0	0	0	Actinobacteria	Actinobacteria	Actinomycetales	Nocardiaceae	Rhodococcus	S000387216 Rhodococcus koreensis (T); DNP505 Lineage=Root;rootrank;Bacteria;domain;"Actinobacteria";phylum;Actinobacteria;class;Actinobacteridae;subclass;Actinomycetales;order;Corynebacterineae;suborder;Nocardiaceae;family;Rhodococcus;genus	98.47	0	689
2913	0	0	0	0	0	0	1	0	0	Actinobacteria	Actinobacteria	Actinomycetales	Nocardiaceae	Rhodococcus	S000893586 Rhodococcus erythropolis; M17-3 Lineage=Root;rootrank;Bacteria;domain;"Actinobacteria";phylum;Actinobacteria;class;Actinobacteridae;subclass;Actinomycetales;order;Corynebacterineae;suborder;unclassified_Corynebacterineae;	100	0	723
997	0	0	0	0	0	0	0	1	0	Actinobacteria	Actinobacteria	Actinomycetales	Nocardiaceae	Rhodococcus	S002350778 Nocardia globerula; type strain: DSM 44596 Lineage=Root;rootrank;Bacteria;domain;"Actinobacteria";phylum;Actinobacteria;class;Actinobacteridae;subclass;Actinomycetales;order;Corynebacterineae;suborder;Nocardiaceae;family;Rhodococcus;genus	99.74	0	717
2146	0	0	0	0	1	1	4	1	1	Actinobacteria	Actinobacteria	Actinomycetales	Nocardiaceae	Rhodococcus	S003716451 Rhodococcus equi; KUDC1815 Lineage=Root;rootrank;Bacteria;domain;"Actinobacteria";phylum;Actinobacteria;class;Actinobacteridae;subclass;Actinomycetales;order;Corynebacterineae;suborder;Nocardiaceae;family;Rhodococcus;genus	100	0	689
674	0	0	0	0	0	0	1	0	0	Actinobacteria	Actinobacteria	Actinomycetales	Nocardiaceae		S001154907 Streptomyces gardneri; HBUM174916 Lineage=Root;rootrank;Bacteria;domain;"Actinobacteria";phylum;Actinobacteria;class;Actinobacteridae;subclass;Actinomycetales;order;Corynebacterineae;suborder;Nocardiaceae;family;Nocardia;genus	98.67	0	665
1387	4	0	3	0	0	3	14	1	1	Actinobacteria	Actinobacteria	Actinomycetales	Nocardioidaceae		S000843257 Nocardioides mesophilus (T); MSL 22 Lineage=Root;rootrank;Bacteria;domain;"Actinobacteria";phylum;Actinobacteria;class;Actinobacteridae;subclass;Actinomycetales;order;Propionibacterineae;suborder;Nocardioidaceae;family;Nocardioides;genus	96.26	1.00E-173	610
1339	0	4	3	0	0	2	0	0	0	Actinobacteria	Actinobacteria	Actinomycetales	Nocardioidaceae	Actinopolymorpha	S000871716 Actinopolymorpha rutila (T); YIM 45725 Lineage=Root;rootrank;Bacteria;domain;"Actinobacteria";phylum;Actinobacteria;class;Actinobacteridae;subclass;Actinomycetales;order;Propionibacterineae;suborder;Nocardioidaceae;family;Actinopolymorpha;genus	95.16	5.00E-167	588
1508	4	2	1	115	13	0	1	0	0	Actinobacteria	Actinobacteria	Actinomycetales	Nocardioidaceae	Aeromicrobium	S000841836 Aeromicrobium erythreum (T); NRRL B-3381 Lineage=Root;rootrank;Bacteria;domain;"Actinobacteria";phylum;Actinobacteria;class;Actinobacteridae;subclass;Actinomycetales;order;Propionibacterineae;suborder;Nocardioidaceae;family;Aeromicrobium;genus	99.46	0	676
1858	0	0	0	5	0	0	0	0	0	Actinobacteria	Actinobacteria	Actinomycetales	Nocardioidaceae	Kribbella	S003745368 Kribbella sandramycini; Tibetlhz-17 Lineage=Root;rootrank;Bacteria;domain;"Actinobacteria";phylum;Actinobacteria;class;Actinobacteridae;subclass;Actinomycetales;order;Propionibacterineae;suborder;Nocardioidaceae;family;Kribbella;genus	99.49	0	713
512	0	0	0	5	3	4	1	0	0	Actinobacteria	Actinobacteria	Actinomycetales	Nocardioidaceae	Nocardioides	S002949797 Nocardioides terrigena; S32569 Lineage=Root;rootrank;Bacteria;domain;"Actinobacteria";phylum;Actinobacteria;class;Actinobacteridae;subclass;Actinomycetales;order;Propionibacterineae;suborder;Nocardioidaceae;family;Nocardioides;genus	98.66	0	662
782	0	0	0	0	1	0	0	0	0	Actinobacteria	Actinobacteria	Actinomycetales	Nocardioidaceae	Nocardioides	S003287795 Nocardioides albertani; CD40127 Lineage=Root;rootrank;Bacteria;domain;"Actinobacteria";phylum;Actinobacteria;class;Actinobacteridae;subclass;Actinomycetales;order;Propionibacterineae;suborder;Nocardioidaceae;family;Nocardioides;genus	100	0	689
2413	0	4	3	0	1	13	31	3	1	Actinobacteria	Actinobacteria	Actinomycetales	Nocardioidaceae	Pimelobacter	S001351114 Nocardioides panacisoli (T); GSoil 346 Lineage=Root;rootrank;Bacteria;domain;"Actinobacteria";phylum;Actinobacteria;class;Actinobacteridae;subclass;Actinomycetales;order;Propionibacterineae;suborder;Nocardioidaceae;family;Nocardioides;genus	98.39	0	656

1840	4	7	2	0	1	10	10	0	0	Actinobacteria	Actinobacteria	Actinomycetales	Nocardioidaceae		S000438006 Nocardioides pyridinolyticus (T); OS4 Lineage=Root;rootrank;Bacteria;domain;"Actinobacteria";phylum;Actinobacteria;class;Actinobacteridae;subclass;Actinomycetales;order;Propionibacterineae;suborder;Nocardioidaceae;family;Nocardioides;genus	97.06	3.00E-179	628
3002	2	2	8	2	11	2	1	2	0	Actinobacteria	Actinobacteria	Actinomycetales	Nocardioidaceae		S000964849 Aeromicrobium ponti (T); type strain: HSW-1 Lineage=Root;rootrank;Bacteria;domain;"Actinobacteria";phylum;Actinobacteria;class;Actinobacteridae;subclass;Actinomycetales;order;Propionibacterineae;suborder;Nocardioidaceae;family;Aeromicrobium;genus	95.17	5.00E-167	588
818	0	0	3	8	11	50	22	9	6	Actinobacteria	Actinobacteria	Actinomycetales	Nocardioidaceae		S002222202 Marmoricola scoriae; type strain: Sco-D01 Lineage=Root;rootrank;Bacteria;domain;"Actinobacteria";phylum;Actinobacteria;class;Actinobacteridae;subclass;Actinomycetales;order;Propionibacterineae;suborder;Nocardioidaceae;family;Marmoricola;genus	99.73	0	684
1408	0	0	0	0	0	1	0	0	0	Actinobacteria	Actinobacteria	Actinomycetales	Nocardioidaceae		S002229402 Kribbella jejuensis; XMU218 Lineage=Root;rootrank;Bacteria;domain;"Actinobacteria";phylum;Actinobacteria;class;Actinobacteridae;subclass;Actinomycetales;order;Propionibacterineae;suborder;Nocardioidaceae;family;Kribbella;genus	93.94	3.00E-169	595
1667	0	1	12	65	88	2	1	5	19	Actinobacteria	Actinobacteria	Actinomycetales	Nocardioidaceae		S003618593 Nocardioides caricicola; CB-281545 Lineage=Root;rootrank;Bacteria;domain;"Actinobacteria";phylum;Actinobacteria;class;Actinobacteridae;subclass;Actinomycetales;order;Propionibacterineae;suborder;Nocardioidaceae;family;Nocardioides;genus	98.66	0	662
830	0	4	0	0	0	0	0	0	0	Actinobacteria	Actinobacteria	Actinomycetales	Nocardiopsaceae		S001094392 Dermacoccus nishinomiyaensis; PC IW01 Lineage=Root;rootrank;Bacteria;domain;"Actinobacteria";phylum;Actinobacteria;class;Actinobacteridae;subclass;Actinomycetales;order;Micrococcineae;suborder;Dermacoccaceae;family;Dermacoccus;genus	93.3	2.00E-155	549
1877	0	3	0	0	0	0	0	0	3	Actinobacteria	Actinobacteria	Actinomycetales	Nocardiopsaceae		S001097276 Amycolatopsis thermoflava; 173573 Lineage=Root;rootrank;Bacteria;domain;"Actinobacteria";phylum;Actinobacteria;class;Actinobacteridae;subclass;Actinomycetales;order;Pseudonocardineae;suborder;Pseudonocardiaceae;family;Amycolatopsis;genus	93.05	1.00E-153	544
2605	0	5	4	1	0	1	6	31	22	Actinobacteria	Actinobacteria	Actinomycetales	Nocardiopsaceae	Nocardiopsis	S000871698 Nesterenkonia terrae; YIM 80556 Lineage=Root;rootrank;Bacteria;domain;"Actinobacteria";phylum;Actinobacteria;class;Actinobacteridae;subclass;Actinomycetales;order;Micrococcineae;suborder;Micrococcaceae;family;Nesterenkonia;genus	93.87	1.00E-158	560
3050	0	19	10	0	0	4	12	0	1	Actinobacteria	Actinobacteria	Actinomycetales	Nocardiopsaceae	Streptomonospora	S001097276 Amycolatopsis thermoflava; 173573 Lineage=Root;rootrank;Bacteria;domain;"Actinobacteria";phylum;Actinobacteria;class;Actinobacteridae;subclass;Actinomycetales;order;Pseudonocardineae;suborder;Pseudonocardiaceae;family;Amycolatopsis;genus	93.13	5.00E-162	571
1195	0	1	1	2	1	1	2	6	3	Actinobacteria	Actinobacteria	Actinomycetales	Promicromonosporaceae	Myceligenerans	S000127486 Cellulomonadaceae str. W6 Lineage=Root;rootrank;Bacteria;domain;"Actinobacteria";phylum;Actinobacteria;class;Actinobacteridae;subclass;Actinomycetales;order;Micrococcineae;suborder;Cellulomonadaceae;family;unclassified_Cellulomonadaceae;	95.15	2.00E-176	619
2066	0	1	0	5	0	2	0	1	8	Actinobacteria	Actinobacteria	Actinomycetales	Promicromonosporaceae		S000009040 Rarobacter incanus (T); IFO 16558 Lineage=Root;rootrank;Bacteria;domain;"Actinobacteria";phylum;Actinobacteria;class;Actinobacteridae;subclass;Actinomycetales;order;Micrococcineae;suborder;Rarobacteraceae;family;Rarobacter;genus	95.15	2.00E-176	619
1316	0	0	0	0	0	0	0	0	1	Actinobacteria	Actinobacteria	Actinomycetales	Propionibacteriaceae	Microlunatus	S001351454 Microlunatus soli (T); CC-012602 Lineage=Root;rootrank;Bacteria;domain;"Actinobacteria";phylum;Actinobacteria;class;Actinobacteridae;subclass;Actinomycetales;order;Propionibacterineae;suborder;Propionibacteriaceae;family;Microlunatus;genus	98.66	0	662
2447	0	0	0	1	0	0	0	0	0	Actinobacteria	Actinobacteria	Actinomycetales	Propionibacteriaceae	Propionibacterium	S000711767 Propionicicella superfundia (T); BL-10 Lineage=Root;rootrank;Bacteria;domain;"Actinobacteria";phylum;Actinobacteria;class;Actinobacteridae;subclass;Actinomycetales;order;Propionibacterineae;suborder;Propionibacteriaceae;family;Propionicicella;genus	92.78	1.00E-152	540
2472	0	0	0	0	0	0	0	0	1	Actinobacteria	Actinobacteria	Actinomycetales	Propionibacteriaceae		S001416768 Microlunatus aurantiacus; 13629F Lineage=Root;rootrank;Bacteria;domain;"Actinobacteria";phylum;Actinobacteria;class;Actinobacteridae;subclass;Actinomycetales;order;Propionibacterineae;suborder;Propionibacteriaceae;family;Microlunatus;genus	97.05	3.00E-179	628
2449	0	0	0	0	0	1	0	0	0	Actinobacteria	Actinobacteria	Actinomycetales	Propionibacteriaceae		S003716770 Naumannella halotolerans; XFB-AF Lineage=Root;rootrank;Bacteria;domain;"Actinobacteria";phylum;Actinobacteria;class;Actinobacteridae;subclass;Actinomycetales;order;Propionibacterineae;suborder;Propionibacteriaceae;family;Microlunatus;genus	96.25	3.00E-174	612
1350	14	94	7	8	13	8	15	81	98	Actinobacteria	Actinobacteria	Actinomycetales	Pseudonocardiaceae		S002156865 Jiangella muralis (T); 15-Je-017 Lineage=Root;rootrank;Bacteria;domain;"Actinobacteria";phylum;Actinobacteria;class;Actinobacteridae;subclass;Actinomycetales;order;Jiangellineae;suborder;Jiangellaceae;family;Jiangella;genus	99.46	0	678
1189	0	0	1	0	0	0	0	0	0	Actinobacteria	Actinobacteria	Actinomycetales	Pseudonocardiaceae	Prauserella	S001264016 Prauserella sediminis (T); YIM 90694 Lineage=Root;rootrank;Bacteria;domain;"Actinobacteria";phylum;Actinobacteria;class;Actinobacteridae;subclass;Actinomycetales;order;Pseudonocardineae;suborder;Pseudonocardiaceae;family;Prauserella;genus	99.73	0	688
2794	0	1	2	36	7	1	1	7	13	Actinobacteria	Actinobacteria	Actinomycetales	Pseudonocardiaceae	Pseudonocardia	S001156502 Pseudonocardia acaciae (T); GMKU095 Lineage=Root;rootrank;Bacteria;domain;"Actinobacteria";phylum;Actinobacteria;class;Actinobacteridae;subclass;Actinomycetales;order;Pseudonocardineae;suborder;Pseudonocardiaceae;family;Pseudonocardia;genus	98.4	0	658
2893	0	0	0	0	1	0	0	0	0	Actinobacteria	Actinobacteria	Actinomycetales	Pseudonocardiaceae	Pseudonocardia	S001793132 Pseudonocardia halophobica; NRRL B-16514 Lineage=Root;rootrank;Bacteria;domain;"Actinobacteria";phylum;Actinobacteria;class;Actinobacteridae;subclass;Actinomycetales;order;Pseudonocardineae;suborder;Pseudonocardiaceae;family;Pseudonocardia;genus	97.61	0	643
1393	10	54	20	86	65	18	13	23	24	Actinobacteria	Actinobacteria	Actinomycetales	Pseudonocardiaceae	Pseudonocardia	S002916906 Pseudonocardia alaniniphila; 14613 Lineage=Root;rootrank;Bacteria;domain;"Actinobacteria";phylum;Actinobacteria;class;Actinobacteridae;subclass;Actinomycetales;order;Pseudonocardineae;suborder;Pseudonocardiaceae;family;Pseudonocardia;genus	99.2	0	675
1434	0	1	0	0	0	0	0	0	0	Actinobacteria	Actinobacteria	Actinomycetales	Pseudonocardiaceae	Thermobispora	S000626965 Thermobifida fusca; K22 Lineage=Root;rootrank;Bacteria;domain;"Actinobacteria";phylum;Actinobacteria;class;Actinobacteridae;subclass;Actinomycetales;order;Jiangellineae;suborder;Jiangellaceae;family;Jiangella;genus	90.1	4.00E-143	508
638	0	0	1	0	0	0	0	0	0	Actinobacteria	Actinobacteria	Actinomycetales	Pseudonocardiaceae	Thermobispora	S002156865 Jiangella muralis (T); 15-Je-017 Lineage=Root;rootrank;Bacteria;domain;"Actinobacteria";phylum;Actinobacteria;class;Actinobacteridae;subclass;Actinomycetales;order;Jiangellineae;suborder;Jiangellaceae;family;Jiangella;genus	90.08	4.00E-143	508
1051	14	15	39	1	0	64	214	2	4	Actinobacteria	Actinobacteria	Actinomycetales	Pseudonocardiaceae		S000978796 Goodfellowia coeruleoviolacea; NBRC 14988 Lineage=Root;rootrank;Bacteria;domain;"Actinobacteria";phylum;Actinobacteria;class;Actinobacteridae;subclass;Actinomycetales;order;Pseudonocardineae;suborder;Pseudonocardiaceae;family;Goodfellowiella;genus	97.05	3.00E-179	628
162	0	0	2	0	0	0	0	0	1	Actinobacteria	Actinobacteria	Actinomycetales	Pseudonocardiaceae		S001577946 Haloechinothrix alba (T); YIM 93221 Lineage=Root;rootrank;Bacteria;domain;"Actinobacteria";phylum;Actinobacteria;class;Actinobacteridae;subclass;Actinomycetales;order;Pseudonocardineae;suborder;Pseudonocardiaceae;family;Haloechinothrix;genus	99.46	0	676
2596	1	22	3	1	0	2	8	0	0	Actinobacteria	Actinobacteria	Actinomycetales	Pseudonocardiaceae		S002032683 Yuhushiella deserti (T); RA45 Lineage=Root;rootrank;Bacteria;domain;"Actinobacteria";phylum;Actinobacteria;class;Actinobacteridae;subclass;Actinomycetales;order;Pseudonocardineae;suborder;Pseudonocardiaceae;family;Yuhushiella;genus	94.91	6.00E-166	584
1412	0	10	0	0	0	0	1	2	2	Actinobacteria	Actinobacteria	Actinomycetales	Pseudonocardiaceae		S002032683 Yuhushiella deserti (T); RA45 Lineage=Root;rootrank;Bacteria;domain;"Actinobacteria";phylum;Actinobacteria;class;Actinobacteridae;subclass;Actinomycetales;order;Pseudonocardineae;suborder;Pseudonocardiaceae;family;Yuhushiella;genus	95.73	2.00E-171	603
1148	4	12	4	0	0	0	2	0	0	Actinobacteria	Actinobacteria	Actinomycetales	Pseudonocardiaceae		S002156865 Jiangella muralis (T); 15-Je-017 Lineage=Root;rootrank;Bacteria;domain;"Actinobacteria";phylum;Actinobacteria;class;Actinobacteridae;subclass;Actinomycetales;order;Jiangellineae;suborder;Jiangellaceae;family;Jiangella;genus	95.98	1.00E-172	606
2584	0	0	0	4	2	0	0	4	14	Actinobacteria	Actinobacteria	Actinomycetales	Pseudonocardiaceae		S002221822 Actinophytocola xinjiangensis; QAIII60 Lineage=Root;rootrank;Bacteria;domain;"Actinobacteria";phylum;Actinobacteria;class;Actinobacteridae;subclass;Actinomycetales;order;Pseudonocardineae;suborder;Pseudonocardiaceae;family;Actinophytocola;genus	98.39	0	654
2297	0	0	0	0	0	0	0	0	4	Actinobacteria	Actinobacteria	Actinomycetales	Pseudonocardiaceae		S002221822 Actinophytocola xinjiangensis; QAIII60 Lineage=Root;rootrank;Bacteria;domain;"Actinobacteria";phylum;Actinobacteria;class;Actinobacteridae;subclass;Actinomycetales;order;Pseudonocardineae;suborder;Pseudonocardiaceae;family;Actinophytocola;genus	97.05	1.00E-178	627
2508	0	12	2	0	0	2	2	4	21	Actinobacteria	Actinobacteria	Actinomycetales	Pseudonocardiaceae		S003719568 Amycolatopsis pretoriensis; SB12 Lineage=Root;rootrank;Bacteria;domain;"Actinobacteria";phylum;Actinobacteria;class;Actinobacteridae;subclass;Actinomycetales;order;Pseudonocardineae;suborder;Pseudonocardiaceae;family;Amycolatopsis;genus	94.92	1.00E-173	610
1938	0	1	31	44	30	1	13	14	22	Actinobacteria	Actinobacteria	Actinomycetales	Sporichthyaceae	Sporichthya	S000022213 Sporichthya polymorpha (T); IFO 12702 Lineage=Root;rootrank;Bacteria;domain;"Actinobacteria";phylum;Actinobacteria;class;Actinobacteridae;subclass;Actinomycetales;order;Frankineae;suborder;Sporichthyaceae;family;Sporichthya;genus	99.46	0	678
2144	0	0	2	3	0	6	4	2	7	Actinobacteria	Actinobacteria	Actinomycetales	Streptomycetaceae		S002035390 Streptomyces cavourensis; YY01-17 Lineage=Root;rootrank;Bacteria;domain;"Actinobacteria";phylum;Actinobacteria;class;Actinobacteridae;subclass;Actinomycetales;order;Streptomycineae;suborder;Streptomycetaceae;family;unclassified_Streptomycetaceae;	100	0	689
2485	1	3	32	54	88	17	62	80	138	Actinobacteria	Actinobacteria	Actinomycetales	Streptomycetaceae		S003301347 Streptomyces lilacinofulvus; BAB407_bhwr818 Lineage=Root;rootrank;Bacteria;domain;"Actinobacteria";phylum;Actinobacteria;class;Actinobacteridae;subclass;Actinomycetales;order;Streptomycineae;suborder;Streptomycetaceae;family;unclassified_Streptomycetaceae;	99.2	0	673
1518	0	0	1	0	0	0	0	0	0	Actinobacteria	Actinobacteria	Actinomycetales	Streptosporangiaceae		S002156865 Jiangella muralis (T); 15-Je-017 Lineage=Root;rootrank;Bacteria;domain;"Actinobacteria";phylum;Actinobacteria;class;Actinobacteridae;subclass;Actinomycetales;order;Jiangellineae;suborder;Jiangellaceae;family;Jiangella;genus	92.68	2.00E-160	566
161	0	1	0	0	0	0	0	0	0	Actinobacteria	Actinobacteria	Actinomycetales	Streptosporangiaceae	Microbispora	S001243905 Amycolatopsis coloradensis; HBUM173696 Lineage=Root;rootrank;Bacteria;domain;"Actinobacteria";phylum;Actinobacteria;class;Actinobacteridae;subclass;Actinomycetales;order;Pseudonocardineae;suborder;Pseudonocardiaceae;family;Amycolatopsis;genus	92.62	9.00E-160	564
2674	0	0	0	0	0	0	0	0	1	Actinobacteria	Actinobacteria	Actinomycetales	Streptosporangiaceae	Microbispora	S001243905 Amycolatopsis coloradensis; HBUM173696 Lineage=Root;rootrank;Bacteria;domain;"Actinobacteria";phylum;Actinobacteria;class;Actinobacteridae;subclass;Actinomycetales;order;Pseudonocardineae;suborder;Pseudonocardiaceae;family;Amycolatopsis;genus	92.33	1.00E-157	556
2437	0	0	1	0	0	2	0	0	0	Actinobacteria	Actinobacteria	Actinomycetales	Streptosporangiaceae	Nonomuraea	S001243905 Amycolatopsis coloradensis; HBUM173696 Lineage=Root;rootrank;Bacteria;domain;"Actinobacteria";phylum;Actinobacteria;class;Actinobacteridae;subclass;Actinomycetales;order;Pseudonocardineae;suborder;Pseudonocardiaceae;family;Amycolatopsis;genus	91.86	3.00E-154	545
926	0	1	0	0	0	0	1	0	1	Actinobacteria	Actinobacteria	Actinomycetales	Streptosporangiaceae	Sphaerisporangium	S001243905 Amycolatopsis coloradensis; HBUM173696 Lineage=Root;rootrank;Bacteria;domain;"Actinobacteria";phylum;Actinobacteria;class;Actinobacteridae;subclass;Actinomycetales;order;Pseudonocardineae;suborder;Pseudonocardiaceae;family;Amycolatopsis;genus	92.02	5.00E-147	521
180	3	2	4	0	2	1	5	1	0	Actinobacteria	Actinobacteria	Actinomycetales	Streptosporangiaceae		S000626965 Thermobifida fusca; K22 Lineage=Root;rootrank;Bacteria;domain;"Actinobacteria";phylum;Actinobacteria;class;Actinobacteridae;subclass;Actinomycetales;order;Jiangellineae;suborder;Jiangellaceae;family;Jiangella;genus	91.69	2.00E-145	516
1648	21	5	3	8	3	7	8	5	0	Actinobacteria	Actinobacteria	Actinomycetales	Thermomonosporaceae	Actinomadura	S000860164 Kutzneria albida (T); NRRL B-24060 Lineage=Root;rootrank;Bacteria;domain;"Actinobacteria";phylum;Actinobacteria;class;Actinobacteridae;subclass;Actinomycetales;order;Pseudonocardineae;suborder;Pseudonocardiaceae;family;Kutzneria;genus	93.11	1.00E-162	573
1696	0	0	0	0	0	0	0	1	1	Actinobacteria	Actinobacteria	Actinomycetales	Thermomonosporaceae	Actinomadura	S002951220 Streptomyces geldanamycininus; BC55 Lineage=Root;rootrank;Bacteria;domain;"Actinobacteria";phylum;Actinobacteria;class;Actinobacteridae;subclass;Actinomycetales;order;Streptomycineae;suborder;Streptomycetaceae;family;Streptomyces;genus	91.76	6.00E-146	518
773	0	1	0	0	1	0	0	0	0	Actinobacteria	Actinobacteria	Actinomycetales	Yaniellaceae	Yaniella	S001264263 Yaniella soli; JSM 070026 Lineage=Root;rootrank;Bacteria;domain;"Actinobacteria";phylum;Actinobacteria;class;Actinobacteridae;subclass;Actinomycetales;order;Micrococcineae;suborder;Micrococcaceae;family;Yaniella;genus	99.2	0	673
3035	0	0	0	0	0	0	1	0	0	Actinobacteria	Actinobacteria	Actinomycetales			S000000995 Geodermatophilus; A7S, basalt outcrop, Sonora desert, USA Lineage=Root;rootrank;Bacteria;domain;"Actinobacteria";phylum;Actinobacteria;class;Actinobacteridae;subclass;Actinomycetales;order;Frankineae;suborder;Geodermatophilaceae;family;Geodermatophilus;genus	87.63	9.00E-120	431
2807	0	0	1	0	0	0	0	0	0	Actinobacteria	Actinobacteria	Actinomycetales			S000022213 Sporichthya polymorpha (T); IFO 12702 Lineage=Root;rootrank;Bacteria;domain;"Actinobacteria";phylum;Actinobacteria;class;Actinobacteridae;subclass;Actinomycetales;order;Frankineae;suborder;Sporichthyaceae;family;Sporichthya;genus	95.44	3.00E-169	595
3184	0	0	1	0	0	0	0	0	0	Actinobacteria	Actinobacteria	Actinomycetales			S000091708 Allokutzneria albata (T); type strain:DSM 44149 Lineage=Root;rootrank;Bacteria;domain;"Actinobacteria";phylum;Actinobacteria;class;Actinobacteridae;subclass;Actinomycetales;order;Pseudonocardineae;suborder;Pseudonocardiaceae;family;Allokutzneria;genus	90.89	1.00E-148	527
734	0	0	2	10	0	0	1	0	0	Actinobacteria	Actinobacteria	Actinomycetales			S000322802 Actinomycetales str. Ellin143 Lineage=Root;rootrank;Bacteria;domain;"Actinobacteria";phylum;Actinobacteria;class;Actinobacteridae;subclass;Actinomycetales;order;Frankineae;suborder;unclassified_Frankineae;	96.78	1.00E-177	623
102	0	1	0	0	0	0	0	0	0	Actinobacteria	Actinobacteria	Actinomycetales			S000382510 Umezawaea tangerina (T); MK27-91F2 Lineage=Root;rootrank;Bacteria;domain;"Actinobacteria";phylum;Actinobacteria;class;Actinobacteridae;subclass;Actinomycetales;order;Pseudonocardineae;suborder;Pseudonocardiaceae;family;Umezawaea;genus	88.77	2.00E-126	453
2744	0	1	2	0	0	0	0	1	0	Actinobacteria	Actinobacteria	Actinomycetales			S000428089 Amycolatopsis sulphurea; DSM 46092T Lineage=Root;rootrank;Bacteria;domain;"Actinobacteria";phylum;Actinobacteria;class;Actinobacteridae;subclass;Actinomycetales;order;Pseudonocardineae;suborder;Pseudonocardiaceae;family;Amycolatopsis;genus	95.19	5.00E-177	621
2688	59	404	377	523	438	400	544	1420	3193	Actinobacteria	Actinobacteria	Actinomycetales			S000429095 Crossiella cryophila (T); NRRL B-16238 Lineage=Root;rootrank;Bacteria;domain;"Actinobacteria";phylum;Actinobacteria;class;Actinobacteridae;subclass;Actinomycetales;order;Pseudonocardineae;suborder;Pseudonocardiaceae;family;Crossiella;genus	96.51	6.00E-176	617
584	0	0	1	0	0	0	0	0	0	Actinobacteria	Actinobacteria	Actinomycetales			S000429095 Crossiella cryophila (T); NRRL B-16238 Lineage=Root;rootrank;Bacteria;domain;"Actinobacteria";phylum;Actinobacteria;class;Actinobacteridae;subclass;Actinomycetales;order;Pseudonocardineae;suborder;Pseudonocardiaceae;family;Crossiella;genus	93.44	5.00E-157	555
712	0	0	0	0	1	0	0	0	0	Actinobacteria	Actinobacteria	Actinomycetales			S000435344 Amycolatopsis australiensis; GY286 Lineage=Root;rootrank;Bacteria;domain;"Actinobacteria";phylum;Actinobacteria;class;Actinobacteridae;subclass;Actinomycetales;order;Pseudonocardineae;suborder;Pseudonocardiaceae;family;Amycolatopsis;genus	91.47	1.00E-143	510
841	64	109	31	2	15	8	13	0	0	Actinobacteria	Actinobacteria	Actinomycetales			S000504628 Jiangella gansuensis (T); YIM 002 Lineage=Root;rootrank;Bacteria;domain;"Actinobacteria";phylum;Actinobacteria;class;Actinobacteridae;subclass;Actinomycetales;order;Jiangellineae;suborder;Jiangellaceae;family;Jiangella;genus	96.51	6.00E-176	617
2985	0	0	0	0	0	1	0	0	0	Actinobacteria	Actinobacteria	Actinomycetales			S000626965 Thermobifida fusca; K22 Lineage=Root;rootrank;Bacteria;domain;"Actinobacteria";phylum;Actinobacteria;class;Actinobacteridae;subclass;Actinomycetales;order;Jiangellineae;suborder;Jiangellaceae;family;Jiangella;genus	95.18	2.00E-176	619
957	0	0	0	0	0	0	2	0	0	Actinobacteria	Actinobacteria	Actinomycetales			S000640727 Ruania albidiflava (T); AS 4.3142; 3-6 Lineage=Root;rootrank;Bacteria;domain;"Actinobacteria";phylum;Actinobacteria;class;Actinobacteridae;subclass;Actinomycetales;order;Micrococcineae;suborder;Ruaniaceae;family;Ruania;genus	96.68	0	652
400	0	0	0	0	0	1	0	0	0	Actinobacteria	Actinobacteria	Actinomycetales			S000640727 Ruania albidiflava (T); AS 4.3142; 3-6 Lineage=Root;rootrank;Bacteria;domain;"Actinobacteria";phylum;Actinobacteria;class;Actinobacteridae;subclass;Actinomycetales;order;Micrococcineae;suborder;Ruaniaceae;family;Ruania;genus	98.21	0	684
2692	0	0	0	0	1	0	0	0	0	Actinobacteria	Actinobacteria	Actinomycetales			S000640797 Pseudonocardia ailaonensis (T); YIM 45505 Lineage=Root;rootrank;Bacteria;domain;"Actinobacteria";phylum;Actinobacteria;class;Actinobacteridae;subclass;Actinomycetales;order;Pseudonocardineae;suborder;Pseudonocardiaceae;family;Pseudonocardia;genus	94.15	6.00E-161	568
2060	0	4	0	0	1	2	2	0	0	Actinobacteria	Actinobacteria	Actinomycetales			S000841835 Kribbella sandramycini; ATCC 39419 Lineage=Root;rootrank;Bacteria;domain;"Actinobacteria";phylum;Actinobacteria;class;Actinobacteridae;subclass;Actinomycetales;order;Propionibacterineae;suborder;Nocardioidaceae;family;Kribbella;genus	94.91	6.00E-166	584
1442	0	0	0	0	0	0	1	0	0	Actinobacteria	Actinobacteria	Actinomycetales			S000843250 Nocardioides sediminis (T); MSL-01 Lineage=Root;rootrank;Bacteria;domain;"Actinobacteria";phylum;Actinobacteria;class;Actinobacteridae;subclass;Actinomycetales;order;Propionibacterineae;suborder;Nocardioidaceae;family;Nocardioides;genus	92.29	3.00E-149	529
2264	0	0	0	1	0	0	0	0	0	Actinobacteria	Actinobacteria	Actinomycetales			S000860164 Kutzneria albida (T); NRRL B-24060 Lineage=Root;rootrank;Bacteria;domain;"Actinobacteria";phylum;Actinobacteria;class;Actinobacteridae;subclass;Actinomycetales;order;Pseudonocardineae;suborder;Pseudonocardiaceae;family;Kutzneria;genus	94.64	1.00E-172	606
2802	11	15	8	0	1	2	12	0	0	Actinobacteria	Actinobacteria	Actinomycetales			S000871716 Actinopolymorpha rutila (T); YIM 45725 Lineage=Root;rootrank;Bacteria;domain;"Actinobacteria";phylum;Actinobacteria;class;Actinobacteridae;subclass;Actinomycetales;order;Propionibacterineae;suborder;Nocardioidaceae;family;Actinopolymorpha;genus	93.82	1.00E-158	560
145	0	2	3	0	1	0	0	0	0	Actinobacteria	Actinobacteria	Actinomycetales			S000871716 Actinopolymorpha rutila (T); YIM 45725 Lineage=Root;rootrank;Bacteria;domain;"Actinobacteria";phylum;Actinobacteria;class;Actinobacteridae;subclass;Actinomycetales;order;Propionibacterineae;suborder;Nocardioidaceae;family;Actinopolymorpha;genus	92.86	2.00E-161	569
1289	3	165	2	0	0	0	0	0	0	Actinobacteria	Actinobacteria	Actinomycetales			S000925479 Amycolatopsis helveola (T); TT 00-43 (= NBRC 103394) Lineage=Root;rootrank;Bacteria;domain;"Actinobacteria";phylum;Actinobacteria;class;Actinobacteridae;subclass;Actinomycetales;order;Pseudonocardineae;suborder;Pseudonocardiaceae;family;Amycolatopsis;genus	93.32	6.00E-156	551
2571	0	0	0	0	1	0	0	0	0	Actinobacteria	Actinobacteria	Actinomycetales			S000964849 Aeromicrobium ponti (T); type strain: HSW-1 Lineage=Root;rootrank;Bacteria;domain;"Actinobacteria";phylum;Actinobacteria;class;Actinobacteridae;subclass;Actinomycetales;order;Propionibacterineae;suborder;Nocardioidaceae;family;Aeromicrobium;genus	94.15	3.00E-169	595
2023	0	0	0	0	0	0	0	1	0	Actinobacteria	Actinobacteria	Actinomycetales			S000978795 Streptomyces tenebrarius; NBRC 16177 Lineage=Root;rootrank;Bacteria;domain;"Actinobacteria";phylum;Actinobacteria;class;Actinobacteridae;subclass;Actinomycetales;order;Pseudonocardineae;suborder;Pseudonocardiaceae;family;Streptoalloteichus;genus	92.76	5.00E-152	538
1801	0	0	0	0	0	0	1	0	0	Actinobacteria	Actinobacteria	Actinomycetales			S000978796 Goodfellowia coeruleoviolacea; NBRC 14988 Lineage=Root;rootrank;Bacteria;domain;"Actinobacteria";phylum;Actinobacteria;class;Actinobacteridae;subclass;Actinomycetales;order;Pseudonocardineae;suborder;Pseudonocardiaceae;family;Goodfellowiella;genus	90.88	6.00E-141	501
37	0	1	0	0	0	0	0	0	0	Actinobacteria	Actinobacteria	Actinomycetales			S000979842 Arthrobacter cumminsii; 742 Lineage=Root;rootrank;Bacteria;domain;"Actinobacteria";phylum;Actinobacteria;class;Actinobacteridae;subclass;Actinomycetales;order;Micrococcineae;suborder;Micrococcaceae;family;Arthrobacter;genus	95.74	0	638
3116	75	130	14	2	14	42	35	0	0	Actinobacteria	Actinobacteria	Actinomycetales			S000996340 Actinophytocola oryzae (T); GMKU 367 Lineage=Root;rootrank;Bacteria;domain;"Actinobacteria";phylum;Actinobacteria;class;Actinobacteridae;subclass;Actinomycetales;order;Pseudonocardineae;suborder;Pseudonocardiaceae;family;Actinophytocola;genus	94.91	2.00E-165	582
2214	0	0	0	0	1	0	0	0	0	Actinobacteria	Actinobacteria	Actinomycetales			S000996340 Actinophytocola oryzae (T); GMKU 367 Lineage=Root;rootrank;Bacteria;domain;"Actinobacteria";phylum;Actinobacteria;class;Actinobacteridae;subclass;Actinomycetales;order;Pseudonocardineae;suborder;Pseudonocardiaceae;family;Actinophytocola;genus	94.64	1.00E-163	577
1496	0	0	0	1	0	0	0	0	0	Actinobacteria	Actinobacteria	Actinomycetales			S000996340 Actinophytocola oryzae (T); GMKU 367 Lineage=Root;rootrank;Bacteria;domain;"Actinobacteria";phylum;Actinobacteria;class;Actinobacteridae;subclass;Actinomycetales;order;Pseudonocardineae;suborder;Pseudonocardiaceae;family;Actinophytocola;genus	93.32	2.00E-155	549
2366	0	0	0	4	1	0	0	0	0	Actinobacteria	Actinobacteria	Actinomycetales			S000996416 Kaistibacter ginsenosidimutans; BXN7-7 Lineage=Root;rootrank;Bacteria;domain;"Actinobacteria";phylum;Actinobacteria;class;Actinobacteridae;subclass;Actinomycetales;order;Frankineae;suborder;unclassified_Frankineae;	94.64	4.00E-173	608
1329	2	0	0	0	0	1	0	0	0	Actinobacteria	Actinobacteria	Actinomycetales			S001097276 Amycolatopsis thermoflava; 173573 Lineage=Root;rootrank;Bacteria;domain;"Actinobacteria";phylum;Actinobacteria;class;Actinobacteridae;subclass;Actinomycetales;order;Pseudonocardineae;suborder;Pseudonocardiaceae;family;Amycolatopsis;genus	95.15	7.00E-176	617
355	0	0	0	0	0	1	0	0	0	Actinobacteria	Actinobacteria	Actinomycetales			S001168680 Serinibacter salmoneus (T); Kis4-28 Lineage=Root;rootrank;Bacteria;domain;"Actinobacteria";phylum;Actinobacteria;class;Actinobacteridae;subclass;Actinomycetales;order;Micrococcineae;suborder;Beutenbergiaceae;family;Serinibacter;genus	96.44	0	647
1706	0	0	0	0	0	2	0	0	0	Actinobacteria	Actinobacteria	Actinomycetales			S001329599 Georgenia halophila (T); YIM 93316 Lineage=Root;rootrank;Bacteria;domain;"Actinobacteria";phylum;Actinobacteria;class;Actinobacteridae;subclass;Actinomycetales;order;Micrococcineae;suborder;Bogoriellaceae;family;Georgenia;genus	98.67	0	664
2977	0	1	0	0	0	0	1	0	1	Actinobacteria	Actinobacteria	Actinomycetales			S001331143 Leifsonia poae; L85 Lineage=Root;rootrank;Bacteria;domain;"Actinobacteria";phylum;Actinobacteria;class;Actinobacteridae;subclass;Actinomycetales;order;Micrococcineae;suborder;Microbacteriaceae;family;Leifsonia;genus	88.2	1.00E-123	444
2220	0	0	0	0	0	0	1	0	0	Actinobacteria	Actinobacteria	Actinomycetales			S001577946 Haloechinothrix alba (T); YIM 93221 Lineage=Root;rootrank;Bacteria;domain;"Actinobacteria";phylum;Actinobacteria;class;Actinobacteridae;subclass;Actinomycetales;order;Pseudonocardineae;suborder;Pseudonocardiaceae;family;Haloechinothrix;genus	94.37	7.00E-171	601
425	265	504	236	15	56	244	266	5	7	Actinobacteria	Actinobacteria	Actinomycetales			S002156865 Jiangella muralis (T); 15-Je-017 Lineage=Root;rootrank;Bacteria;domain;"Actinobacteria";phylum;Actinobacteria;class;Actinobacteridae;subclass;Actinomycetales;order;Jiangellineae;suborder;Jiangellaceae;family;Jiangella;genus	95.71	6.00E-171	601
1268	0	0	4	4	2	19	6	0	0	Actinobacteria	Actinobacteria	Actinomycetales			S002221822 Actinophytocola xinjiangensis; QAIII60 Lineage=Root;rootrank;Bacteria;domain;"Actinobacteria";phylum;Actinobacteria;class;Actinobacteridae;subclass;Actinomycetales;order;Pseudonocardineae;suborder;Pseudonocardiaceae;family;Actinophytocola;genus	93.03	1.00E-153	544
2637	0	0	1	0	0	0	0	0	0	Actinobacteria	Actinobacteria	Actinomycetales			S002287801 Acidothermus cellulolyticus (T); 11B; ATCC 43068 Lineage=Root;rootrank;Bacteria;domain;"Actinobacteria";phylum;Actinobacteria;class;Actinobacteridae;subclass;Actinomycetales;order;Frankineae;suborder;Acidothermaceae;family;Acidothermus;genus	94.92	6.00E-166	584
641	0	0	2	0	0	0	0	0	0	Actinobacteria	Actinobacteria	Actinomycetales			S002288444 Tropheryma whipplei str. Twist Lineage=Root;rootrank;Bacteria;domain;"Actinobacteria";phylum;Actinobacteria;class;Actinobacteridae;subclass;Actinomycetales;order;Micrococcineae;suborder;Cellulomonadaceae;family;Tropheryma;genus	90.15	1.00E-143	510
2907	0	76	64	0	4	11	36	0	0	Actinobacteria	Actinobacteria	Actinomycetales			S002291206 Frankia alni ACN14a; ACN14A Lineage=Root;rootrank;Bacteria;domain;"Actinobacteria";phylum;Actinobacteria;class;Actinobacteridae;subclass;Actinomycetales;order;Frankineae;suborder;unclassified_Frankineae;	94.64	1.00E-163	577
510	0	4	0	0	0	0	0	0	6	Actinobacteria	Actinobacteria	Actinomycetales			S002306167 Leifsonia xyli; X4 Lineage=Root;rootrank;Bacteria;domain;"Actinobacteria";phylum;Actinobacteria;class;Actinobacteridae;subclass;Actinomycetales;order;Micrococcineae;suborder;Microbacteriaceae;family;Leifsonia;genus	88.01	3.00E-129	462
362	5	10	7	1	2	0	3	1	0	Actinobacteria	Actinobacteria	Actinomycetales			S002441462 Pseudonocardia dioxanivorans CB1190 Lineage=Root;rootrank;Bacteria;domain;"Actinobacteria";phylum;Actinobacteria;class;Actinobacteridae;subclass;Actinomycetales;order;Pseudonocardineae;suborder;Pseudonocardiaceae;family;Pseudonocardia;genus	94.69	3.00E-164	579
1331	0	0	0	1	0	0	0	0	0	Actinobacteria	Actinobacteria	Actinomycetales			S002441462 Pseudonocardia dioxanivorans CB1190 Lineage=Root;rootrank;Bacteria;domain;"Actinobacteria";phylum;Actinobacteria;class;Actinobacteridae;subclass;Actinomycetales;order;Pseudonocardineae;suborder;Pseudonocardiaceae;family;Pseudonocardia;genus	92.37	1.00E-157	556
542	0	0	0	1	0	0	0	0	0	Actinobacteria	Actinobacteria	Actinomycetales			S002911908 Actinotalea fermentans; KNUC9060 Lineage=Root;rootrank;Bacteria;domain;"Actinobacteria";phylum;Actinobacteria;class;Actinobacteridae;subclass;Actinomycetales;order;Micrococcineae;suborder;Cellulomonadaceae;family;unclassified_Cellulomonadaceae;	95.74	6.00E-171	601
1600	0	0	1	4	4	2	0	2	0	Actinobacteria	Actinobacteria	Actinomycetales			S002949598 Actinotalea fermentans; NBRC 105374 Lineage=Root;rootrank;Bacteria;domain;"Actinobacteria";phylum;Actinobacteria;class;Actinobacteridae;subclass;Actinomycetales;order;Micrococcineae;suborder;Cellulomonadaceae;family;Actinotalea;genus	98.93	0	667
863	0	0	0	0	0	0	0	1	1	Actinobacteria	Actinobacteria	Actinomycetales			S002950459 Frankia symbiont of Datisca glomerata; 4085684 Lineage=Root;rootrank;Bacteria;domain;"Actinobacteria";phylum;Actinobacteria;class;Actinobacteridae;subclass;Actinomycetales;order;Frankineae;suborder;unclassified_Frankineae;	88.33	1.00E-123	444
828	0	0	0	13	0	0	0	0	0	Actinobacteria	Actinobacteria	Actinomycetales			S003288299 Amycolatopsis vancoresmycina; S-12 Lineage=Root;rootrank;Bacteria;domain;"Actinobacteria";phylum;Actinobacteria;class;Actinobacteridae;subclass;Actinomycetales;order;Pseudonocardineae;suborder;Pseudonocardiaceae;family;Amycolatopsis;genus	92.86	2.00E-160	566
662	7	1	0	0	0	1	2	0	0	Actinobacteria	Actinobacteria	Actinomycetales			S003305348 Blastococcus saxobsidens; DD13 Lineage=Root;rootrank;Bacteria;domain;"Actinobacteria";phylum;Actinobacteria;class;Actinobacteridae;subclass;Actinomycetales;order;Frankineae;suborder;Geodermatophilaceae;family;Blastococcus;genus	93.88	1.00E-167	590
3065	2	0	0	1	0	0	0	0	0	Actinobacteria	Actinobacteria	Actinomycetales			S003710613 Georgenia muralis; SU28 Lineage=Root;rootrank;Bacteria;domain;"Actinobacteria";phylum;Actinobacteria;class;Actinobacteridae;subclass;Actinomycetales;order;Micrococcineae;suborder;Bogoriellaceae;family;Georgenia;genus	94.13	3.00E-169	595
1791	6	63	196	5	91	67	137	23	7	Actinobacteria	Actinobacteria	Euzebiales	Euzebiaceae		S000824847 Nitriliruptor alkaliphilus (T); ANL-iso2 Lineage=Root;rootrank;Bacteria;domain;"Actinobacteria";phylum;Actinobacteria;class;Nitriliruptoridae;subclass;Nitriliruptorales;order;Nitriliruptoraceae;family;Nitriliruptor;genus	93.55	5.00E-157	555
2042	2	81	72	4	6	30	47	69	36	Actinobacteria	Actinobacteria	Euzebiales	Euzebiaceae		S000824847 Nitriliruptor alkaliphilus (T); ANL-iso2 Lineage=Root;rootrank;Bacteria;domain;"Actinobacteria";phylum;Actinobacteria;class;Nitriliruptoridae;subclass;Nitriliruptorales;order;Nitriliruptoraceae;family;Nitriliruptor;genus	90.88	2.00E-140	499
2858	2	29	13	1	6	27	46	15	5	Actinobacteria	Actinobacteria	Euzebiales	Euzebiaceae		S000824847 Nitriliruptor alkaliphilus (T); ANL-iso2 Lineage=Root;rootrank;Bacteria;domain;"Actinobacteria";phylum;Actinobacteria;class;Nitriliruptoridae;subclass;Nitriliruptorales;order;Nitriliruptoraceae;family;Nitriliruptor;genus	93.55	5.00E-157	555
1680	2	11	5	2	3	1	0	6	16	Actinobacteria	Actinobacteria	Euzebiales	Euzebiaceae		S000824847 Nitriliruptor alkaliphilus (T); ANL-iso2 Lineage=Root;rootrank;Bacteria;domain;"Actinobacteria";phylum;Actinobacteria;class;Nitriliruptoridae;subclass;Nitriliruptorales;order;Nitriliruptoraceae;family;Nitriliruptor;genus	95.98	1.00E-172	606
2147	0	0	0	1	0	0	0	2	5	Actinobacteria	Actinobacteria	Euzebiales	Euzebiaceae		S000824847 Nitriliruptor alkaliphilus (T); ANL-iso2 Lineage=Root;rootrank;Bacteria;domain;"Actinobacteria";phylum;Actinobacteria;class;Nitriliruptoridae;subclass;Nitriliruptorales;order;Nitriliruptoraceae;family;Nitriliruptor;genus	96.77	5.00E-177	621
1754	0	0	4	0	0	1	0	0	0	Actinobacteria	Actinobacteria	Euzebiales	Euzebiaceae		S000824847 Nitriliruptor alkaliphilus (T); ANL-iso2 Lineage=Root;rootrank;Bacteria;domain;"Actinobacteria";phylum;Actinobacteria;class;Nitriliruptoridae;subclass;Nitriliruptorales;order;Nitriliruptoraceae;family;Nitriliruptor;genus	93.38	9.00E-165	580
2998	0	0	0	0	1	0	0	1	3	Actinobacteria	Actinobacteria	Euzebiales	Euzebiaceae		S000824847 Nitriliruptor alkaliphilus (T); ANL-iso2 Lineage=Root;rootrank;Bacteria;domain;"Actinobacteria";phylum;Actinobacteria;class;Nitriliruptoridae;subclass;Nitriliruptorales;order;Nitriliruptoraceae;family;Nitriliruptor;genus	95.65	3.00E-179	628
747	0	0	0	0	1	1	0	0	0	Actinobacteria	Actinobacteria	Euzebiales	Euzebiaceae		S000824847 Nitriliruptor alkaliphilus (T); ANL-iso2 Lineage=Root;rootrank;Bacteria;domain;"Actinobacteria";phylum;Actinobacteria;class;Nitriliruptoridae;subclass;Nitriliruptorales;order;Nitriliruptoraceae;family;Nitriliruptor;genus	94.37	7.00E-171	601
1928	0	0	1	0	0	0	0	0	0	Actinobacteria	Actinobacteria	Euzebiales	Euzebiaceae		S000824847 Nitriliruptor alkaliphilus (T); ANL-iso2 Lineage=Root;rootrank;Bacteria;domain;"Actinobacteria";phylum;Actinobacteria;class;Nitriliruptoridae;subclass;Nitriliruptorales;order;Nitriliruptoraceae;family;Nitriliruptor;genus	97.96	0	678
1701	0	0	0	0	1	0	0	0	0	Actinobacteria	Actinobacteria	Euzebiales	Euzebiaceae		S000824847 Nitriliruptor alkaliphilus (T); ANL-iso2 Lineage=Root;rootrank;Bacteria;domain;"Actinobacteria";phylum;Actinobacteria;class;Nitriliruptoridae;subclass;Nitriliruptorales;order;Nitriliruptoraceae;family;Nitriliruptor;genus	92.2	4.00E-148	525
1069	0	0	0	0	0	0	0	0	1	Actinobacteria	Actinobacteria	Euzebiales	Euzebiaceae		S000824847 Nitriliruptor alkaliphilus (T); ANL-iso2 Lineage=Root;rootrank;Bacteria;domain;"Actinobacteria";phylum;Actinobacteria;class;Nitriliruptoridae;subclass;Nitriliruptorales;order;Nitriliruptoraceae;family;Nitriliruptor;genus	95.03	2.00E-170	599
2917	0	0	0	0	0	0	0	1	0	Actinobacteria	Actinobacteria	Euzebiales	Euzebiaceae	Euzebia	S000769575 Thiohalomonas denitrificans; HLD 14 Lineage=Root;rootrank;Bacteria;domain;"Proteobacteria";phylum;Gammaproteobacteria;class;Gammaproteobacteria_incertae_sedis;order;Thiohalomonas;genus	87.76	2.00E-127	457
429	19	22	10	3	7	7	8	6	10	Actinobacteria	Actinobacteria	Euzebiales	Euzebiaceae	Euzebia	S001548570 Euzebya tangerina (T) Lineage=Root;rootrank;Bacteria;domain;"Actinobacteria";phylum;Actinobacteria;class;Nitriliruptoridae;subclass;Euzebyales;order;Euzebyaceae;family;Euzebya;genus	91.98	5.00E-147	521
2228	6	30	9	0	6	3	3	2	7	Actinobacteria	Actinobacteria	Euzebiales	Euzebiaceae	Euzebia	S001548570 Euzebya tangerina (T) Lineage=Root;rootrank;Bacteria;domain;"Actinobacteria";phylum;Actinobacteria;class;Nitriliruptoridae;subclass;Euzebyales;order;Euzebyaceae;family;Euzebya;genus	91.37	2.00E-151	536
1032	0	0	4	3	6	0	0	50	19	Actinobacteria	Actinobacteria	Euzebiales	Euzebiaceae	Euzebia	S001548570 Euzebya tangerina (T) Lineage=Root;rootrank;Bacteria;domain;"Actinobacteria";phylum;Actinobacteria;class;Nitriliruptoridae;subclass;Euzebyales;order;Euzebyaceae;family;Euzebya;genus	97.59	0	640
274	0	0	1	2	0	0	0	44	41	Actinobacteria	Actinobacteria	Euzebiales	Euzebiaceae	Euzebia	S001548570 Euzebya tangerina (T) Lineage=Root;rootrank;Bacteria;domain;"Actinobacteria";phylum;Actinobacteria;class;Nitriliruptoridae;subclass;Euzebyales;order;Euzebyaceae;family;Euzebya;genus	91.98	1.00E-147	523
608	1	1	4	11	6	5	6	7	15	Actinobacteria	Actinobacteria	Euzebiales	Euzebiaceae	Euzebia	S001548570 Euzebya tangerina (T) Lineage=Root;rootrank;Bacteria;domain;"Actinobacteria";phylum;Actinobacteria;class;Nitriliruptoridae;subclass;Euzebyales;order;Euzebyaceae;family;Euzebya;genus	91.71	2.00E-145	516
2556	0	1	12	5	2	1	2	0	12	Actinobacteria	Actinobacteria	Euzebiales	Euzebiaceae	Euzebia	S001548570 Euzebya tangerina (T) Lineage=Root;rootrank;Bacteria;domain;"Actinobacteria";phylum;Actinobacteria;class;Nitriliruptoridae;subclass;Euzebyales;order;Euzebyaceae;family;Euzebya;genus	93.83	3.00E-159	562
1688	0	0	2	4	5	4	4	1	5	Actinobacteria	Actinobacteria	Euzebiales	Euzebiaceae	Euzebia	S001548570 Euzebya tangerina (T) Lineage=Root;rootrank;Bacteria;domain;"Actinobacteria";phylum;Actinobacteria;class;Nitriliruptoridae;subclass;Euzebyales;order;Euzebyaceae;family;Euzebya;genus	93.13	4.00E-163	575
1402	1	3	1	0	1	0	0	2	1	Actinobacteria	Actinobacteria	Euzebiales	Euzebiaceae	Euzebia	S001548570 Euzebya tangerina (T) Lineage=Root;rootrank;Bacteria;domain;"Actinobacteria";phylum;Actinobacteria;class;Nitriliruptoridae;subclass;Euzebyales;order;Euzebyaceae;family;Euzebya;genus	92.84	1.00E-152	540
1885	0	0	0	0	2	0	0	0	0	Actinobacteria	Actinobacteria	Euzebiales	Euzebiaceae	Euzebia	S001548570 Euzebya tangerina (T) Lineage=Root;rootrank;Bacteria;domain;"Actinobacteria";phylum;Actinobacteria;class;Nitriliruptoridae;subclass;Euzebyales;order;Euzebyaceae;family;Euzebya;genus	92.11	2.00E-156	553
131	0	0	0	0	0	1	0	0	1	Actinobacteria	Actinobacteria	Euzebiales	Euzebiaceae	Euzebia	S001548570 Euzebya tangerina (T) Lineage=Root;rootrank;Bacteria;domain;"Actinobacteria";phylum;Actinobacteria;class;Nitriliruptoridae;subclass;Euzebyales;order;Euzebyaceae;family;Euzebya;genus	91.98	1.00E-147	523
1222	0	0	1	0	0	0	0	0	0	Actinobacteria	Actinobacteria	Euzebiales	Euzebiaceae	Euzebia	S001548570 Euzebya tangerina (T) Lineage=Root;rootrank;Bacteria;domain;"Actinobacteria";phylum;Actinobacteria;class;Nitriliruptoridae;subclass;Euzebyales;order;Euzebyaceae;family;Euzebya;genus	93.05	3.00E-154	545
3260	0	0	1	0	0	0	0	0	0	Actinobacteria	Actinobacteria	Euzebiales	Euzebiaceae	Euzebia	S001548570 Euzebya tangerina (T) Lineage=Root;rootrank;Bacteria;domain;"Actinobacteria";phylum;Actinobacteria;class;Nitriliruptoridae;subclass;Euzebyales;order;Euzebyaceae;family;Euzebya;genus	95.44	1.00E-168	593
3137	0	0	0	0	0	0	0	1	0	Actinobacteria	Actinobacteria	Euzebiales	Euzebiaceae	Euzebia	S001548570 Euzebya tangerina (T) Lineage=Root;rootrank;Bacteria;domain;"Actinobacteria";phylum;Actinobacteria;class;Nitriliruptoridae;subclass;Euzebyales;order;Euzebyaceae;family;Euzebya;genus	91.6	1.00E-152	540
1643	0	0	0	2	0	13	12	0	2	Actinobacteria	Actinobacteria	Euzebiales	Euzebiaceae		S000824847 Nitriliruptor alkaliphilus (T); ANL-iso2 Lineage=Root;rootrank;Bacteria;domain;"Actinobacteria";phylum;Actinobacteria;class;Nitriliruptoridae;subclass;Nitriliruptorales;order;Nitriliruptoraceae;family;Nitriliruptor;genus	88.98	1.00E-128	460
2124	0	0	0	1	0	0	2	1	5	Actinobacteria	Actinobacteria	Euzebiales	Euzebiaceae		S000824847 Nitriliruptor alkaliphilus (T); ANL-iso2 Lineage=Root;rootrank;Bacteria;domain;"Actinobacteria";phylum;Actinobacteria;class;Nitriliruptoridae;subclass;Nitriliruptorales;order;Nitriliruptoraceae;family;Nitriliruptor;genus	90.03	1.00E-142	507
1033	0	0	0	0	0	1	2	0	0	Actinobacteria	Actinobacteria	koll3			S000091708 Allokutzneria albata (T); type strain:DSM 44149 Lineage=Root;rootrank;Bacteria;domain;"Actinobacteria";phylum;Actinobacteria;class;Actinobacteridae;subclass;Actinomycetales;order;Pseudonocardineae;suborder;Pseudonocardiaceae;family;Allokutzneria;genus	85.64	1.00E-107	390
766	0	0	0	2	0	0	0	0	0	Actinobacteria	Actinobacteria	koll3			S000130885 Thermoleophilum album (T); type strain: ATCC 35263 Lineage=Root;rootrank;Bacteria;domain;"Actinobacteria";phylum;Actinobacteria;class;Rubrobacteridae;subclass;Thermoleophilales;order;Thermoleophilaceae;family;Thermoleophilum;genus	85.37	2.00E-106	387
1095	0	0	0	0	0	0	0	1	0	Actinobacteria	Actinobacteria	koll3			S000610985 Parastreptomyces abscessus; B20 Lineage=Root;rootrank;Bacteria;domain;"Actinobacteria";phylum;Actinobacteria;class;Actinobacteridae;subclass;Actinomycetales;order;Streptomycineae;suborder;Streptomycetaceae;family;unclassified_Streptomycetaceae;	88.56	2.00E-126	453
2375	0	0	4	34	15	12	10	21	8	Actinobacteria	Actinobacteria	koll3			S000652087 Streptomyces griseoluteus; NBRC 13375 Lineage=Root;rootrank;Bacteria;domain;"Actinobacteria";phylum;Actinobacteria;class;Actinobacteridae;subclass;Actinomycetales;order;Streptomycineae;suborder;Streptomycetaceae;family;Streptomyces;genus	89.95	8.00E-135	481
2519	0	0	0	0	0	0	0	0	2	Actinobacteria	Actinobacteria	koll3			S000841843 Rubrobacter taiwanensis (T); LS-293 Lineage=Root;rootrank;Bacteria;domain;"Actinobacteria";phylum;Actinobacteria;class;Rubrobacteridae;subclass;Rubrobacterales;order;"Rubrobacterineae";suborder;Rubrobacteraceae;family;Rubrobacter;genus	86.72	3.00E-119	429
3151	0	0	6	28	37	2	1	37	53	Actinobacteria	Actinobacteria	koll3			S001014085 Iamia majanohamensis (T); NBRC 102561 Lineage=Root;rootrank;Bacteria;domain;"Actinobacteria";phylum;Actinobacteria;class;Acidimicrobidae;subclass;Acidimicrobiales;order;"Acidimicrobineae";suborder;Iamiaceae;family;Iamia;genus	88.06	4.00E-123	442
2377	0	0	0	1	1	3	3	0	4	Actinobacteria	Actinobacteria	koll3			S001154799 Lechevalieria aerocolonigenes; HBUM174553 Lineage=Root;rootrank;Bacteria;domain;"Actinobacteria";phylum;Actinobacteria;class;Actinobacteridae;subclass;Actinomycetales;order;Pseudonocardineae;suborder;Pseudonocardiaceae;family;unclassified_Pseudonocardiaceae;	85.6	1.00E-107	390
2534	3	3	269	374	455	466	566	277	515	Actinobacteria	Actinobacteria	koll3			S002164967 Aciditerrimonas ferrireducens (T); IC-180 Lineage=Root;rootrank;Bacteria;domain;"Actinobacteria";phylum;Actinobacteria;class;Acidimicrobidae;subclass;Acidimicrobiales;order;"Acidimicrobineae";suborder;"Acidimicrobineae"_incertae_sedis;family;Aciditerrimonas;genus	89.92	8.00E-135	481
3243	72	125	172	87	106	114	190	222	313	Actinobacteria	Actinobacteria	koll3			S002164967 Aciditerrimonas ferrireducens (T); IC-180 Lineage=Root;rootrank;Bacteria;domain;"Actinobacteria";phylum;Actinobacteria;class;Acidimicrobidae;subclass;Acidimicrobiales;order;"Acidimicrobineae";suborder;"Acidimicrobineae"_incertae_sedis;family;Aciditerrimonas;genus	89.63	4.00E-133	475

1049	0	0	0	1	1	2	0	0	1	Actinobacteria	Actinobacteria	koll3		S002164967 Aciditerrimonas ferrireducens (T); IC-180 Lineage=Root;rootrank;Bacteria;domain;"Actinobacteria";phylum;Actinobacteria;class;Acidimicrobidae;subclass;Acidimicrobiales;order;"Acidimicrobineae";suborder;"Acidimicrobineae"_incertae_sedis;family;Aciditerrimonas;genus	87.63	6.00E-127	455
2280	0	0	0	0	0	0	0	1	3	Actinobacteria	Actinobacteria	koll3		S002164967 Aciditerrimonas ferrireducens (T); IC-180 Lineage=Root;rootrank;Bacteria;domain;"Actinobacteria";phylum;Actinobacteria;class;Acidimicrobidae;subclass;Acidimicrobiales;order;"Acidimicrobineae";suborder;"Acidimicrobineae"_incertae_sedis;family;Aciditerrimonas;genus	89.12	8.00E-130	464
2046	7	16	506	391	394	408	636	823	1117	Actinobacteria	Actinobacteria	koll3		S003305355 Blastococcus saxobsidens; DS18 Lineage=Root;rootrank;Bacteria;domain;"Actinobacteria";phylum;Actinobacteria;class;Actinobacteridae;subclass;Actinomycetales;order;Frankineae;suborder;Geodermatophilaceae;family;Blastococcus;genus	88	4.00E-123	442
2790	0	0	5	40	42	7	8	16	207	Actinobacteria	Actinobacteria	koll3		S003717423 Streptomyces variabilis; PO-178 Lineage=Root;rootrank;Bacteria;domain;"Actinobacteria";phylum;Actinobacteria;class;Actinobacteridae;subclass;Actinomycetales;order;Streptomycineae;suborder;Streptomycetaceae;family;Streptomyces;genus	88.62	2.00E-126	453
1778	0	0	0	4	5	0	0	0	2	Actinobacteria	Actinobacteria	koll3		S003717423 Streptomyces variabilis; PO-178 Lineage=Root;rootrank;Bacteria;domain;"Actinobacteria";phylum;Actinobacteria;class;Actinobacteridae;subclass;Actinomycetales;order;Streptomycineae;suborder;Streptomycetaceae;family;Streptomyces;genus	87.85	1.00E-128	460
198	4	1	35	81	65	169	190	122	220	Actinobacteria	Actinobacteria	MC47		S002447326 Gaiella occulta; F2-233 Lineage=Root;rootrank;Bacteria;domain;"Actinobacteria";phylum;Actinobacteria;class;Rubrobacteridae;subclass;Solirubrobacterales;order;Conexibacteraceae;family;Conexibacter;genus	93.55	5.00E-157	555
1786	0	0	10	272	49	56	40	124	108	Actinobacteria	Actinobacteria	MC47		S002447326 Gaiella occulta; F2-233 Lineage=Root;rootrank;Bacteria;domain;"Actinobacteria";phylum;Actinobacteria;class;Rubrobacteridae;subclass;Solirubrobacterales;order;Conexibacteraceae;family;Conexibacter;genus	95.7	2.00E-170	599
1860	0	0	4	36	20	19	26	25	32	Actinobacteria	Actinobacteria	MC47		S002447326 Gaiella occulta; F2-233 Lineage=Root;rootrank;Bacteria;domain;"Actinobacteria";phylum;Actinobacteria;class;Rubrobacteridae;subclass;Solirubrobacterales;order;Conexibacteraceae;family;Conexibacter;genus	93.35	3.00E-164	579
923	0	0	1	26	22	0	0	0	2	Actinobacteria	Actinobacteria	MC47		S002447326 Gaiella occulta; F2-233 Lineage=Root;rootrank;Bacteria;domain;"Actinobacteria";phylum;Actinobacteria;class;Rubrobacteridae;subclass;Solirubrobacterales;order;Conexibacteraceae;family;Conexibacter;genus	93.01	1.00E-153	544
1900	0	0	1	9	8	9	3	4	12	Actinobacteria	Actinobacteria	MC47		S002447326 Gaiella occulta; F2-233 Lineage=Root;rootrank;Bacteria;domain;"Actinobacteria";phylum;Actinobacteria;class;Rubrobacteridae;subclass;Solirubrobacterales;order;Conexibacteraceae;family;Conexibacter;genus	93.82	4.00E-158	558
1807	0	0	1	22	0	2	0	2	0	Actinobacteria	Actinobacteria	MC47		S002447326 Gaiella occulta; F2-233 Lineage=Root;rootrank;Bacteria;domain;"Actinobacteria";phylum;Actinobacteria;class;Rubrobacteridae;subclass;Solirubrobacterales;order;Conexibacteraceae;family;Conexibacter;genus	96.24	1.00E-173	610
2597	0	0	0	5	3	0	0	10	3	Actinobacteria	Actinobacteria	MC47		S002447326 Gaiella occulta; F2-233 Lineage=Root;rootrank;Bacteria;domain;"Actinobacteria";phylum;Actinobacteria;class;Rubrobacteridae;subclass;Solirubrobacterales;order;Conexibacteraceae;family;Conexibacter;genus	94.89	2.00E-165	582
467	0	0	0	2	0	0	1	6	13	Actinobacteria	Actinobacteria	MC47		S002447326 Gaiella occulta; F2-233 Lineage=Root;rootrank;Bacteria;domain;"Actinobacteria";phylum;Actinobacteria;class;Rubrobacteridae;subclass;Solirubrobacterales;order;Conexibacteraceae;family;Conexibacter;genus	94.12	3.00E-169	595
740	0	0	0	2	1	1	1	2	9	Actinobacteria	Actinobacteria	MC47		S002447326 Gaiella occulta; F2-233 Lineage=Root;rootrank;Bacteria;domain;"Actinobacteria";phylum;Actinobacteria;class;Rubrobacteridae;subclass;Solirubrobacterales;order;Conexibacteraceae;family;Conexibacter;genus	93.28	8.00E-155	547
2547	0	0	2	5	0	3	0	0	1	Actinobacteria	Actinobacteria	MC47		S002447326 Gaiella occulta; F2-233 Lineage=Root;rootrank;Bacteria;domain;"Actinobacteria";phylum;Actinobacteria;class;Rubrobacteridae;subclass;Solirubrobacterales;order;Conexibacteraceae;family;Conexibacter;genus	95.7	2.00E-170	599
2934	0	0	0	0	2	0	0	6	1	Actinobacteria	Actinobacteria	MC47		S002447326 Gaiella occulta; F2-233 Lineage=Root;rootrank;Bacteria;domain;"Actinobacteria";phylum;Actinobacteria;class;Rubrobacteridae;subclass;Solirubrobacterales;order;Conexibacteraceae;family;Conexibacter;genus	92.49	2.00E-150	532
1650	0	0	0	5	0	0	0	1	2	Actinobacteria	Actinobacteria	MC47		S002447326 Gaiella occulta; F2-233 Lineage=Root;rootrank;Bacteria;domain;"Actinobacteria";phylum;Actinobacteria;class;Rubrobacteridae;subclass;Solirubrobacterales;order;Conexibacteraceae;family;Conexibacter;genus	97.19	0	662
598	0	0	0	0	1	0	0	2	0	Actinobacteria	Actinobacteria	MC47		S002447326 Gaiella occulta; F2-233 Lineage=Root;rootrank;Bacteria;domain;"Actinobacteria";phylum;Actinobacteria;class;Rubrobacteridae;subclass;Solirubrobacterales;order;Conexibacteraceae;family;Conexibacter;genus	94.88	3.00E-174	612
1452	0	0	0	2	0	0	0	0	0	Actinobacteria	Actinobacteria	MC47		S002447326 Gaiella occulta; F2-233 Lineage=Root;rootrank;Bacteria;domain;"Actinobacteria";phylum;Actinobacteria;class;Rubrobacteridae;subclass;Solirubrobacterales;order;Conexibacteraceae;family;Conexibacter;genus	94.09	2.00E-160	566
548	0	0	0	1	0	0	0	0	0	Actinobacteria	Actinobacteria	MC47		S002447326 Gaiella occulta; F2-233 Lineage=Root;rootrank;Bacteria;domain;"Actinobacteria";phylum;Actinobacteria;class;Rubrobacteridae;subclass;Solirubrobacterales;order;Conexibacteraceae;family;Conexibacter;genus	93.09	8.00E-155	547
2196	0	0	0	1	0	0	0	0	0	Actinobacteria	Actinobacteria	MC47		S002447326 Gaiella occulta; F2-233 Lineage=Root;rootrank;Bacteria;domain;"Actinobacteria";phylum;Actinobacteria;class;Rubrobacteridae;subclass;Solirubrobacterales;order;Conexibacteraceae;family;Conexibacter;genus	94.89	2.00E-165	582
330	0	1	0	0	0	0	0	0	0	Actinobacteria	Actinobacteria	Rubrobacterales	Rubrobacteraceae	S000841843 Rubrobacter taiwanensis (T); LS-293 Lineage=Root;rootrank;Bacteria;domain;"Actinobacteria";phylum;Actinobacteria;class;Rubrobacteridae;subclass;Rubrobacterales;order;"Rubrobacterineae";suborder;Rubrobacteraceae;family;Rubrobacter;genus	89.25	2.00E-130	466
1821	0	0	0	9	0	3	0	1	0	Actinobacteria	Actinobacteria	Solirubrobacterales		S000040793 Actinomycetales; MC 19 Lineage=Root;rootrank;Bacteria;domain;"Actinobacteria";phylum;Actinobacteria;class;Rubrobacteridae;subclass;unclassified_Rubrobacteridae;	92.66	1.00E-148	527
228	10	4	2	0	0	7	2	0	0	Actinobacteria	Actinobacteria	Solirubrobacterales		S000130885 Thermoleophilum album (T); type strain: ATCC 35263 Lineage=Root;rootrank;Bacteria;domain;"Actinobacteria";phylum;Actinobacteria;class;Rubrobacteridae;subclass;Thermoleophilales;order;Thermoleophilaceae;family;Thermoleophilum;genus	92.76	5.00E-152	538
2851	7	25	7	3	12	59	94	0	4	Actinobacteria	Actinobacteria	Solirubrobacterales		S000627870 Solirubrobacter soli (T); Gsoil 355 Lineage=Root;rootrank;Bacteria;domain;"Actinobacteria";phylum;Actinobacteria;class;Rubrobacteridae;subclass;Solirubrobacterales;order;Solirubrobacteraceae;family;Solirubrobacter;genus	92.74	5.00E-152	538
1453	0	0	2	35	14	11	8	3	18	Actinobacteria	Actinobacteria	Solirubrobacterales		S000627870 Solirubrobacter soli (T); Gsoil 355 Lineage=Root;rootrank;Bacteria;domain;"Actinobacteria";phylum;Actinobacteria;class;Rubrobacteridae;subclass;Solirubrobacterales;order;Solirubrobacteraceae;family;Solirubrobacter;genus	94.88	3.00E-174	612
2646	0	2	5	3	15	20	22	0	0	Actinobacteria	Actinobacteria	Solirubrobacterales		S000627870 Solirubrobacter soli (T); Gsoil 355 Lineage=Root;rootrank;Bacteria;domain;"Actinobacteria";phylum;Actinobacteria;class;Rubrobacteridae;subclass;Solirubrobacterales;order;Solirubrobacteraceae;family;Solirubrobacter;genus	94.37	5.00E-162	571
1940	0	0	0	1	0	23	31	4	10	Actinobacteria	Actinobacteria	Solirubrobacterales		S000627870 Solirubrobacter soli (T); Gsoil 355 Lineage=Root;rootrank;Bacteria;domain;"Actinobacteria";phylum;Actinobacteria;class;Rubrobacteridae;subclass;Solirubrobacterales;order;Solirubrobacteraceae;family;Solirubrobacter;genus	93.28	2.00E-155	549
951	0	0	0	4	8	10	8	3	2	Actinobacteria	Actinobacteria	Solirubrobacterales		S000627870 Solirubrobacter soli (T); Gsoil 355 Lineage=Root;rootrank;Bacteria;domain;"Actinobacteria";phylum;Actinobacteria;class;Rubrobacteridae;subclass;Solirubrobacterales;order;Solirubrobacteraceae;family;Solirubrobacter;genus	94.12	3.00E-169	595
1956	0	0	0	3	2	0	1	0	0	Actinobacteria	Actinobacteria	Solirubrobacterales		S000627870 Solirubrobacter soli (T); Gsoil 355 Lineage=Root;rootrank;Bacteria;domain;"Actinobacteria";phylum;Actinobacteria;class;Rubrobacteridae;subclass;Solirubrobacterales;order;Solirubrobacteraceae;family;Solirubrobacter;genus	94.12	1.00E-168	593
569	0	0	0	0	0	2	1	0	0	Actinobacteria	Actinobacteria	Solirubrobacterales		S000627870 Solirubrobacter soli (T); Gsoil 355 Lineage=Root;rootrank;Bacteria;domain;"Actinobacteria";phylum;Actinobacteria;class;Rubrobacteridae;subclass;Solirubrobacterales;order;Solirubrobacteraceae;family;Solirubrobacter;genus	93.01	1.00E-153	544
1506	0	0	0	0	0	0	0	1	0	Actinobacteria	Actinobacteria	Solirubrobacterales		S000627870 Solirubrobacter soli (T); Gsoil 355 Lineage=Root;rootrank;Bacteria;domain;"Actinobacteria";phylum;Actinobacteria;class;Rubrobacteridae;subclass;Solirubrobacterales;order;Solirubrobacteraceae;family;Solirubrobacter;genus	90.61	2.00E-145	516
3226	1	0	4	40	19	0	0	0	4	Actinobacteria	Actinobacteria	Solirubrobacterales		S002290817 Conexibacter woesei (T); DSM 14684 Lineage=Root;rootrank;Bacteria;domain;"Actinobacteria";phylum;Actinobacteria;class;Rubrobacteridae;subclass;Solirubrobacterales;order;Conexibacteraceae;family;Conexibacter;genus	92.2	1.00E-148	527
1364	0	0	3	1	3	0	0	4	3	Actinobacteria	Actinobacteria	Solirubrobacterales		S002290817 Conexibacter woesei (T); DSM 14684 Lineage=Root;rootrank;Bacteria;domain;"Actinobacteria";phylum;Actinobacteria;class;Rubrobacteridae;subclass;Solirubrobacterales;order;Conexibacteraceae;family;Conexibacter;genus	91.13	5.00E-142	505
1302	1	0	0	0	0	0	0	0	0	Actinobacteria	Actinobacteria	Solirubrobacterales		S002290817 Conexibacter woesei (T); DSM 14684 Lineage=Root;rootrank;Bacteria;domain;"Actinobacteria";phylum;Actinobacteria;class;Rubrobacteridae;subclass;Solirubrobacterales;order;Conexibacteraceae;family;Conexibacter;genus	92.09	7.00E-156	551
2374	1	0	0	0	0	0	0	0	0	Actinobacteria	Actinobacteria	Solirubrobacterales		S002290817 Conexibacter woesei (T); DSM 14684 Lineage=Root;rootrank;Bacteria;domain;"Actinobacteria";phylum;Actinobacteria;class;Rubrobacteridae;subclass;Solirubrobacterales;order;Conexibacteraceae;family;Conexibacter;genus	91.94	5.00E-147	521
1971	0	0	0	0	0	0	0	1	0	Actinobacteria	Actinobacteria	Solirubrobacterales		S002290817 Conexibacter woesei (T); DSM 14684 Lineage=Root;rootrank;Bacteria;domain;"Actinobacteria";phylum;Actinobacteria;class;Rubrobacteridae;subclass;Solirubrobacterales;order;Conexibacteraceae;family;Conexibacter;genus	92.27	1.00E-148	527
532	0	0	0	12	5	0	0	0	0	Actinobacteria	Actinobacteria	Solirubrobacterales		S002440613 Conexibacter arvalis; KV-962 Lineage=Root;rootrank;Bacteria;domain;"Actinobacteria";phylum;Actinobacteria;class;Rubrobacteridae;subclass;Solirubrobacterales;order;Conexibacteraceae;family;Conexibacter;genus	95.43	1.00E-168	593
1610	0	0	3	3	4	3	3	1	0	Actinobacteria	Actinobacteria	Solirubrobacterales		S002440613 Conexibacter arvalis; KV-962 Lineage=Root;rootrank;Bacteria;domain;"Actinobacteria";phylum;Actinobacteria;class;Rubrobacteridae;subclass;Solirubrobacterales;order;Conexibacteraceae;family;Conexibacter;genus	95.44	1.00E-168	593
253	3	172	286	77	142	17	21	12	4	Actinobacteria	Actinobacteria	Solirubrobacterales		S002440614 Conexibacter arvalis; KV-963 Lineage=Root;rootrank;Bacteria;domain;"Actinobacteria";phylum;Actinobacteria;class;Rubrobacteridae;subclass;Solirubrobacterales;order;Conexibacteraceae;family;Conexibacter;genus	94.64	1.00E-163	577
1427	24	1	11	0	0	0	2	0	0	Actinobacteria	Actinobacteria	Solirubrobacterales		S002440614 Conexibacter arvalis; KV-963 Lineage=Root;rootrank;Bacteria;domain;"Actinobacteria";phylum;Actinobacteria;class;Rubrobacteridae;subclass;Solirubrobacterales;order;Conexibacteraceae;family;Conexibacter;genus	93.28	2.00E-155	549
1490	5	2	5	2	3	3	3	7	1	Actinobacteria	Actinobacteria	Solirubrobacterales		S002440614 Conexibacter arvalis; KV-963 Lineage=Root;rootrank;Bacteria;domain;"Actinobacteria";phylum;Actinobacteria;class;Rubrobacteridae;subclass;Solirubrobacterales;order;Conexibacteraceae;family;Conexibacter;genus	94.89	2.00E-165	582
889	1	0	0	0	7	1	1	9	4	Actinobacteria	Actinobacteria	Solirubrobacterales		S002440614 Conexibacter arvalis; KV-963 Lineage=Root;rootrank;Bacteria;domain;"Actinobacteria";phylum;Actinobacteria;class;Rubrobacteridae;subclass;Solirubrobacterales;order;Conexibacteraceae;family;Conexibacter;genus	93.82	1.00E-158	560
3104	0	2	4	0	4	0	2	0	0	Actinobacteria	Actinobacteria	Solirubrobacterales		S002440614 Conexibacter arvalis; KV-963 Lineage=Root;rootrank;Bacteria;domain;"Actinobacteria";phylum;Actinobacteria;class;Rubrobacteridae;subclass;Solirubrobacterales;order;Conexibacteraceae;family;Conexibacter;genus	93.11	4.00E-163	575
678	0	0	0	0	0	1	1	0	0	Actinobacteria	Actinobacteria	Solirubrobacterales		S002440614 Conexibacter arvalis; KV-963 Lineage=Root;rootrank;Bacteria;domain;"Actinobacteria";phylum;Actinobacteria;class;Rubrobacteridae;subclass;Solirubrobacterales;order;Conexibacteraceae;family;Conexibacter;genus	90.88	2.00E-140	499
2151	0	0	1	6	0	0	0	1	2	Actinobacteria	Actinobacteria	Solirubrobacterales	Patulibacteraceae	S000040793 Actinomycetales; MC 19 Lineage=Root;rootrank;Bacteria;domain;"Actinobacteria";phylum;Actinobacteria;class;Rubrobacteridae;subclass;unclassified_Rubrobacteridae;	93.19	5.00E-152	538
2702	1	0	0	0	0	1	0	0	0	Actinobacteria	Actinobacteria	Solirubrobacterales	Patulibacteraceae	S002290817 Conexibacter woesei (T); DSM 14684 Lineage=Root;rootrank;Bacteria;domain;"Actinobacteria";phylum;Actinobacteria;class;Rubrobacteridae;subclass;Solirubrobacterales;order;Conexibacteraceae;family;Conexibacter;genus	95.71	2.00E-180	632
1140	0	0	0	1	0	0	0	0	0	Actinobacteria	Actinobacteria	Solirubrobacterales	Patulibacteraceae	S002290817 Conexibacter woesei (T); DSM 14684 Lineage=Root;rootrank;Bacteria;domain;"Actinobacteria";phylum;Actinobacteria;class;Rubrobacteridae;subclass;Solirubrobacterales;order;Conexibacteraceae;family;Conexibacter;genus	94.13	3.00E-169	595
2630	4	19	3	3	4	3	7	2	7	Actinobacteria	Actinobacteria	Solirubrobacterales	Patulibacteraceae	S002440614 Conexibacter arvalis; KV-963 Lineage=Root;rootrank;Bacteria;domain;"Actinobacteria";phylum;Actinobacteria;class;Rubrobacteridae;subclass;Solirubrobacterales;order;Conexibacteraceae;family;Conexibacter;genus	96.24	1.00E-173	610
2778	0	0	2	16	5	1	1	2	4	Actinobacteria	Actinobacteria	Solirubrobacterales	Patulibacteraceae	S002440614 Conexibacter arvalis; KV-963 Lineage=Root;rootrank;Bacteria;domain;"Actinobacteria";phylum;Actinobacteria;class;Rubrobacteridae;subclass;Solirubrobacterales;order;Conexibacteraceae;family;Conexibacter;genus	94.88	3.00E-174	612
189	0	0	1	3	1	0	0	0	0	Actinobacteria	Actinobacteria	Solirubrobacterales	Patulibacteraceae	S002440614 Conexibacter arvalis; KV-963 Lineage=Root;rootrank;Bacteria;domain;"Actinobacteria";phylum;Actinobacteria;class;Rubrobacteridae;subclass;Solirubrobacterales;order;Conexibacteraceae;family;Conexibacter;genus	95.43	1.00E-168	593
537	0	0	0	0	2	0	0	0	0	Actinobacteria	Actinobacteria	Solirubrobacterales	Patulibacteraceae	S002440614 Conexibacter arvalis; KV-963 Lineage=Root;rootrank;Bacteria;domain;"Actinobacteria";phylum;Actinobacteria;class;Rubrobacteridae;subclass;Solirubrobacterales;order;Conexibacteraceae;family;Conexibacter;genus	96.51	2.00E-175	616
961	0	0	0	0	0	0	1	0	0	Actinobacteria	Actinobacteria	Solirubrobacterales	Patulibacteraceae	S002440614 Conexibacter arvalis; KV-963 Lineage=Root;rootrank;Bacteria;domain;"Actinobacteria";phylum;Actinobacteria;class;Rubrobacteridae;subclass;Solirubrobacterales;order;Conexibacteraceae;family;Conexibacter;genus	93.98	9.00E-170	597
186	0	2	4	5	3	0	2	0	1	Actinobacteria	Actinobacteria	Solirubrobacterales	Solirubrobacteraceae	S000627870 Solirubrobacter soli (T); Gsoil 355 Lineage=Root;rootrank;Bacteria;domain;"Actinobacteria";phylum;Actinobacteria;class;Rubrobacteridae;subclass;Solirubrobacterales;order;Solirubrobacteraceae;family;Solirubrobacter;genus	99.19	0	671
1896	2	0	0	0	0	0	0	0	0	Actinobacteria	Actinobacteria	Solirubrobacterales		S002290817 Conexibacter woesei (T); DSM 14684 Lineage=Root;rootrank;Bacteria;domain;"Actinobacteria";phylum;Actinobacteria;class;Rubrobacteridae;subclass;Solirubrobacterales;order;Conexibacteraceae;family;Conexibacter;genus	91.4	1.00E-143	510
505	1	2	7	10	7	3	4	3	11	Actinobacteria	Actinobacteria	Solirubrobacterales		S002440613 Conexibacter arvalis; KV-962 Lineage=Root;rootrank;Bacteria;domain;"Actinobacteria";phylum;Actinobacteria;class;Rubrobacteridae;subclass;Solirubrobacterales;order;Conexibacteraceae;family;Conexibacter;genus	95.15	2.00E-176	619
1816	3	1	0	2	1	0	2	4	1	Actinobacteria	Actinobacteria	Solirubrobacterales		S002440614 Conexibacter arvalis; KV-963 Lineage=Root;rootrank;Bacteria;domain;"Actinobacteria";phylum;Actinobacteria;class;Rubrobacteridae;subclass;Solirubrobacterales;order;Conexibacteraceae;family;Conexibacter;genus	98.39	0	654
3037	1	2	0	0	0	0	0	0	0	Actinobacteria	Actinobacteria	Solirubrobacterales		S002440614 Conexibacter arvalis; KV-963 Lineage=Root;rootrank;Bacteria;domain;"Actinobacteria";phylum;Actinobacteria;class;Rubrobacteridae;subclass;Solirubrobacterales;order;Conexibacteraceae;family;Conexibacter;genus	95.43	1.00E-168	593
340	0	0	0	0	0	0	0	1	0	Actinobacteria	Actinobacteria	Solirubrobacterales		S002440614 Conexibacter arvalis; KV-963 Lineage=Root;rootrank;Bacteria;domain;"Actinobacteria";phylum;Actinobacteria;class;Rubrobacteridae;subclass;Solirubrobacterales;order;Conexibacteraceae;family;Conexibacter;genus	95.4	5.00E-177	621
2961	0	0	0	0	0	0	0	1	0	Actinobacteria	Actinobacteria	Solirubrobacterales		S002440614 Conexibacter arvalis; KV-963 Lineage=Root;rootrank;Bacteria;domain;"Actinobacteria";phylum;Actinobacteria;class;Rubrobacteridae;subclass;Solirubrobacterales;order;Conexibacteraceae;family;Conexibacter;genus	94.63	1.00E-172	606
1421	0	0	0	0	0	0	0	0	1	Actinobacteria	Actinobacteria	Solirubrobacterales		S002440614 Conexibacter arvalis; KV-963 Lineage=Root;rootrank;Bacteria;domain;"Actinobacteria";phylum;Actinobacteria;class;Rubrobacteridae;subclass;Solirubrobacterales;order;Conexibacteraceae;family;Conexibacter;genus	94.67	3.00E-164	579
421	0	0	0	0	1	0	0	0	1	Actinobacteria	Actinobacteria			S000002406 Sporichthya polymorpha Lineage=Root;rootrank;Bacteria;domain;"Actinobacteria";phylum;Actinobacteria;class;Actinobacteridae;subclass;Actinomycetales;order;Frankineae;suborder;Sporichthyaceae;family;Sporichthya;genus	88.64	1.00E-133	477
1646	0	0	0	0	0	1	0	0	0	Actinobacteria	Actinobacteria			S000128481 Thermoleophilum album; type strain: ATCC 35266 Lineage=Root;rootrank;Bacteria;domain;"Actinobacteria";phylum;Actinobacteria;class;Rubrobacteridae;subclass;Thermoleophilales;order;Thermoleophilaceae;family;Thermoleophilum;genus	88.55	4.00E-133	475
685	2	40	14	0	8	6	10	53	26	Actinobacteria	Actinobacteria			S000824847 Nitriliruptor alkaliphilus (T); ANL-iso2 Lineage=Root;rootrank;Bacteria;domain;"Actinobacteria";phylum;Actinobacteria;class;Nitriliruptoridae;subclass;Nitriliruptorales;order;Nitriliruptoraceae;family;Nitriliruptor;genus	90.91	5.00E-137	488
2691	1	11	1	0	0	8	6	0	0	Actinobacteria	Actinobacteria			S000824847 Nitriliruptor alkaliphilus (T); ANL-iso2 Lineage=Root;rootrank;Bacteria;domain;"Actinobacteria";phylum;Actinobacteria;class;Nitriliruptoridae;subclass;Nitriliruptorales;order;Nitriliruptoraceae;family;Nitriliruptor;genus	90.41	1.00E-133	477
2095	0	9	6	0	2	1	2	1	2	Actinobacteria	Actinobacteria			S000824847 Nitriliruptor alkaliphilus (T); ANL-iso2 Lineage=Root;rootrank;Bacteria;domain;"Actinobacteria";phylum;Actinobacteria;class;Nitriliruptoridae;subclass;Nitriliruptorales;order;Nitriliruptoraceae;family;Nitriliruptor;genus	92.43	3.00E-154	545
3041	0	0	0	1	0	0	0	0	0	Actinobacteria	Actinobacteria			S000824847 Nitriliruptor alkaliphilus (T); ANL-iso2 Lineage=Root;rootrank;Bacteria;domain;"Actinobacteria";phylum;Actinobacteria;class;Nitriliruptoridae;subclass;Nitriliruptorales;order;Nitriliruptoraceae;family;Nitriliruptor;genus	91.36	1.00E-147	523
264	0	0	0	2	0	0	0	1	0	Actinobacteria	Actinobacteria			S000841843 Rubrobacter taiwanensis (T); LS-293 Lineage=Root;rootrank;Bacteria;domain;"Actinobacteria";phylum;Actinobacteria;class;Rubrobacteridae;subclass;Rubrobacterales;order;"Rubrobacterineae";suborder;Rubrobacteraceae;family;Rubrobacter;genus	89.62	3.00E-140	499
98	0	0	0	1	1	0	0	0	1	Actinobacteria	Actinobacteria			S000860564 Streptomyces sulphureus L180 Lineage=Root;rootrank;Bacteria;domain;"Actinobacteria";phylum;Actinobacteria;class;Actinobacteridae;subclass;Actinomycetales;order;Streptomycineae;suborder;Streptomycetaceae;family;Streptomyces;genus	88.92	3.00E-135	483
1441	0	0	0	1	0	0	0	0	0	Actinobacteria	Actinobacteria			S000860564 Streptomyces sulphureus L180 Lineage=Root;rootrank;Bacteria;domain;"Actinobacteria";phylum;Actinobacteria;class;Actinobacteridae;subclass;Actinomycetales;order;Streptomycineae;suborder;Streptomycetaceae;family;Streptomyces;genus	87.59	2.00E-126	453
2362	3	21	16	1	5	4	18	0	0	Actinobacteria	Actinobacteria			S001263702 actinomycete M1yB2 Lineage=Root;rootrank;Bacteria;domain;"Actinobacteria";phylum;Actinobacteria;class;Actinobacteridae;subclass;Actinomycetales;order;Streptomycineae;suborder;Streptomycetaceae;family;Streptomyces;genus	86.61	1.00E-113	411
668	0	0	0	0	0	0	1	0	0	Actinobacteria	Actinobacteria			S001548570 Euzebya tangerina (T) Lineage=Root;rootrank;Bacteria;domain;"Actinobacteria";phylum;Actinobacteria;class;Nitriliruptoridae;subclass;Euzebyales;order;Euzebyaceae;family;Euzebya;genus	88.77	1.00E-127	457
1903	0	0	0	3	0	0	0	0	0	Actinobacteria	Actinobacteria			S002164967 Aciditerrimonas ferrireducens (T); IC-180 Lineage=Root;rootrank;Bacteria;domain;"Actinobacteria";phylum;Actinobacteria;class;Acidimicrobidae;subclass;Acidimicrobiales;order;"Acidimicrobineae";suborder;"Acidimicrobineae"_incertae_sedis;family;Aciditerrimonas;genus	90.1	1.00E-143	510
835	0	0	0	2	0	0	0	0	0	Actinobacteria	Actinobacteria			S002164967 Aciditerrimonas ferrireducens (T); IC-180 Lineage=Root;rootrank;Bacteria;domain;"Actinobacteria";phylum;Actinobacteria;class;Acidimicrobidae;subclass;Acidimicrobiales;order;"Acidimicrobineae";suborder;"Acidimicrobineae"_incertae_sedis;family;Aciditerrimonas;genus	92.13	5.00E-157	555
1837	0	0	0	0	1	25	24	1	4	Actinobacteria	Actinobacteria			S002287801 Acidothermus cellulolyticus (T); 11B; ATCC 43068 Lineage=Root;rootrank;Bacteria;domain;"Actinobacteria";phylum;Actinobacteria;class;Actinobacteridae;subclass;Actinomycetales;order;Frankineae;suborder;Acidothermaceae;family;Acidothermus;genus	89.07	1.00E-128	460
73	0	0	0	2	0	0	0	0	0	Actinobacteria	Actinobacteria			S002290817 Conexibacter woesei (T); DSM 14684 Lineage=Root;rootrank;Bacteria;domain;"Actinobacteria";phylum;Actinobacteria;class;Rubrobacteridae;subclass;Solirubrobacterales;order;Conexibacteraceae;family;Conexibacter;genus	91.18	5.00E-142	505
2754	0	0	0	0	1	0	0	0	0	Actinobacteria	Actinobacteria			S002290817 Conexibacter woesei (T); DSM 14684 Lineage=Root;rootrank;Bacteria;domain;"Actinobacteria";phylum;Actinobacteria;class;Rubrobacteridae;subclass;Solirubrobacterales;order;Conexibacteraceae;family;Conexibacter;genus	90.59	7.00E-146	518
1707	0	0	0	0	0	0	0	0	1	Actinobacteria	Actinobacteria			S002447326 Gaiella occulta; F2-233 Lineage=Root;rootrank;Bacteria;domain;"Actinobacteria";phylum;Actinobacteria;class;Rubrobacteridae;subclass;Solirubrobacterales;order;Conexibacteraceae;family;Conexibacter;genus	89.37	1.00E-138	494
605	0	0	0	1	0	0	0	0	0	Actinobacteria	Actinobacteria			S002950459 Frankia symbiont of Datisca glomerata; 4085684 Lineage=Root;rootrank;Bacteria;domain;"Actinobacteria";phylum;Actinobacteria;class;Actinobacteridae;subclass;Actinomycetales;order;Frankineae;suborder;unclassified_Frankineae;	90.13	4.00E-143	508
1751	0	5	0	0	3	5	3	0	0	Actinobacteria	Actinobacteria			S003283412 actinomycete C12 Lineage=Root;rootrank;Bacteria;domain;"Actinobacteria";phylum;Actinobacteria;class;Actinobacteridae;subclass;Actinomycetales;order;Pseudonocardineae;suborder;Pseudonocardiaceae;family;Lechevalieria;genus	90.69	1.00E-138	494
129	0	0	1	0	0	0	0	0	0	Actinobacteria	Actinobacteria			S003305355 Blastococcus saxobsidens; DS18 Lineage=Root;rootrank;Bacteria;domain;"Actinobacteria";phylum;Actinobacteria;class;Actinobacteridae;subclass;Actinomycetales;order;Frankineae;suborder;Geodermatophilaceae;family;Blastococcus;genus	91.12	2.00E-150	532
3014	0	0	0	0	0	1	0	0	0	Actinobacteria	Actinobacteria			S003315216 Iamia majanohamensis; F12 Lineage=Root;rootrank;Bacteria;domain;"Actinobacteria";phylum;Actinobacteria;class;Acidimicrobidae;subclass;Acidimicrobiales;order;"Acidimicrobineae";suborder;unclassified_"Acidimicrobineae";	88.56	1.00E-122	440
506	0	0	0	2	6	2	4	0	7	Actinobacteria	Actinobacteria			S003719507 Streptomyces radiopugnans; M27 Lineage=Root;rootrank;Bacteria;domain;"Actinobacteria";phylum;Actinobacteria;class;Actinobacteridae;subclass;Actinomycetales;order;Streptomycineae;suborder;Streptomycetaceae;family;Streptomyces;genus	88.3	3.00E-124	446
1729	0	0	0	0	0	0	1	0	1	Actinobacteria	Actinobacteria			S003719507 Streptomyces radiopugnans; M27 Lineage=Root;rootrank;Bacteria;domain;"Actinobacteria";phylum;Actinobacteria;class;Actinobacteridae;subclass;Actinomycetales;order;Streptomycineae;suborder;Streptomycetaceae;family;Streptomyces;genus	87.63	2.00E-126	453
1160	0	0	0	7	8	3	6	4	6	Actinobacteria	Actinobacteria			S003746586 Streptomyces flavovariabilis; NB-2 Lineage=Root;rootrank;Bacteria;domain;"Actinobacteria";phylum;Actinobacteria;class;Actinobacteridae;subclass;Actinomycetales;order;Streptomycineae;suborder;Streptomycetaceae;family;Streptomyces;genus	88.56	7.00E-126	451
2659	0	0	0	0	0	0	0	1	0	Actinobacteria				S000436484 Moorella thermoacetica Lineage=Root;rootrank;Bacteria;domain;Firmicutes;phylum;Clostridia;class;Thermoanaerobacterales;order;Thermoanaerobacteraceae;family;Moorella;genus	85.91	3.00E-109	396
1262	0	0	0	0	0	0	0	0	1	Actinobacteria				S002288846 Spirochaeta smaragdinae DSM 11293 Lineage=Root;rootrank;Bacteria;domain;"Spirochaetes";phylum;Spirochaetes;class;Spirochaetales;order;Spirochaetaceae;family;Spirochaeta;genus	81.46	2.00E-81	303
2256	0	0	0	0	0	0	0	1	0	Actinobacteria				S002952177 planctomycete MSF146 Lineage=Root;rootrank;Bacteria;domain;"Planctomycetes";phylum;"Planctomycetacia";class;Planctomycetales;order;Planctomycetaceae;family;Blastopirellula;genus	80.59	4.00E-73	276
928	0	0	0	3	1	3	4	0	2	Armatimonadetes	5B-8			S000040793 Actinomycetales; MC 19 Lineage=Root;rootrank;Bacteria;domain;"Actinobacteria";phylum;Actinobacteria;class;Rubrobacteridae;subclass;unclassified_Rubrobacteridae;	87.23	9.00E-115	414
195	0	0	4	16	38	55	40	8	17	Armatimonadetes	5B-8			S000390567 Thermolithobacter ferrireducens (T); KA2a Lineage=Root;rootrank;Bacteria;domain;Firmicutes;phylum;Thermolithobacteria;class;Thermolithobacterales;order;Thermolithobacteraceae;family;Thermolithobacter;genus	85.41	2.00E-105	383
2123	0	0	0	3	3	30	22	2	0	Armatimonadetes	5B-8			S000390568 Thermolithobacter ferrireducens; JW/KA-2; KA2b Lineage=Root;rootrank;Bacteria;domain;Firmicutes;phylum;Thermolithobacteria;class;Thermolithobacterales;order;Thermolithobacteraceae;family;Thermolithobacter;genus	86.25	9.00E-110	398
1544	0	0	0	5	0	2	1	1	2	Armatimonadetes	5B-8			S000390568 Thermolithobacter ferrireducens; JW/KA-2; KA2b Lineage=Root;rootrank;Bacteria;domain;Firmicutes;phylum;Thermolithobacteria;class;Thermolithobacterales;order;Thermolithobacteraceae;family;Thermolithobacter;genus	86.33	5.00E-112	405
1239	0	0	1	33	8	0	0	1	0	Armatimonadetes	CH2			S001577623 Fimbriimonas ginsengisoli Gsoil 348 Lineage=Root;rootrank;Bacteria;domain;"Armatimonadetes";phylum;Armatimonadetes_gp5;genus	87.57	1.00E-118	427
24	0	0	0	24	2	0	0	0	0	Armatimonadetes	CH2			S001577623 Fimbriimonas ginsengisoli Gsoil 348 Lineage=Root;rootrank;Bacteria;domain;"Armatimonadetes";phylum;Armatimonadetes_gp5;genus	86.6	1.00E-113	411
846	0	0	0	0	2	0	0	1	2	Armatimonadetes	CH2			S001577623 Fimbriimonas ginsengisoli Gsoil 348 Lineage=Root;rootrank;Bacteria;domain;"Armatimonadetes";phylum;Armatimonadetes_gp5;genus	88.47	2.00E-125	449
2001	0	0	0	2	0	0	0	0	0	Armatimonadetes	CH2			S001577623 Fimbriimonas ginsengisoli Gsoil 348 Lineage=Root;rootrank;Bacteria;domain;"Armatimonadetes";phylum;Armatimonadetes_gp5;genus	92.25	1.00E-148	527
1955	0	0	0	1	0	0	0	1	0	Armatimonadetes	CH2			S001577623 Fimbriimonas ginsengisoli Gsoil 348 Lineage=Root;rootrank;Bacteria;domain;"Armatimonadetes";phylum;Armatimonadetes_gp5;genus	89.87	1.00E-133	477
2846	0	0	0	1	0	0	0	0	0	Armatimonadetes	CH2			S001577623 Fimbriimonas ginsengisoli Gsoil 348 Lineage=Root;rootrank;Bacteria;domain;"Armatimonadetes";phylum;Armatimonadetes_gp5;genus	87.37	1.00E-118	427
2861	0	0	0	1	0	0	0	0	0	Armatimonadetes	CH2			S001577623 Fimbriimonas ginsengisoli Gsoil 348 Lineage=Root;rootrank;Bacteria;domain;"Armatimonadetes";phylum;Armatimonadetes_gp5;genus	91.98	5.00E-147	521
2051	0	0	0	1	0	0	0	2	2	Armatimonadetes	Sa-H			S000383946 Thermanaeromonas toyohensis (T); ToBE Lineage=Root;rootrank;Bacteria;domain;Firmicutes;phylum;Clostridia;class;Thermoanaerobacterales;order;Thermoanaerobacteraceae;family;Thermanaeromonas;genus	85.56	5.00E-107	388
1678	0	0	0	2	0	0	0	0	0	Armatimonadetes	Sa-H			S002291168 Syntrophomonas wolfei (T); Goettingen Lineage=Root;rootrank;Bacteria;domain;Firmicutes;phylum;Clostridia;class;Clostridiales;order;Syntrophomonadaceae;family;Syntrophomonas;genus	85.35	7.00E-111	401
3145	0	0	3	25	20	24	12	1	20	Armatimonadetes	Sa-H			S003259712 Syntrophothermus lipocalidus DSM 12680; TGB-C1 Lineage=Root;rootrank;Bacteria;domain;Firmicutes;phylum;Clostridia;class;Clostridiales;order;Syntrophomonadaceae;family;Syntrophothermus;genus	86.51	5.00E-112	405
363	0	0	1	16	9	0	1	5	4	Armatimonadetes	Sa-H			S003259712 Syntrophothermus lipocalidus DSM 12680; TGB-C1 Lineage=Root;rootrank;Bacteria;domain;Firmicutes;phylum;Clostridia;class;Clostridiales;order;Syntrophomonadaceae;family;Syntrophothermus;genus	85.56	5.00E-107	388
220	0	0	1	0	0	0	0	0	0	Armatimonadetes	Sa-H			S003259712 Syntrophothermus lipocalidus DSM 12680; TGB-C1 Lineage=Root;rootrank;Bacteria;domain;Firmicutes;phylum;Clostridia;class;Clostridiales;order;Syntrophomonadaceae;family;Syntrophothermus;genus	85.56	2.00E-106	387
3049	0	0	0	1	2	0	0	0	0	Armatimonadetes	Sa-H			S003614554 Aneurinibacillus danicus; M35 Lineage=Root;rootrank;Bacteria;domain;Firmicutes;phylum;Bacilli;class;Bacillales;order;Paenibacillaceae 2;family;Aneurinibacillus;genus	85.03	1.00E-103	377
1357	0	0	0	0	0	0	1	0	0	Armatimonadetes				S000000782 Ammoniphilus oxalaticus (T); RAOx1 Lineage=Root;rootrank;Bacteria;domain;Firmicutes;phylum;Bacilli;class;Bacillales;order;Paenibacillaceae 2;family;Ammoniphilus;genus	85.79	4.00E-113	409
441	2	1	3	0	3	1	5	0	0	Armatimonadetes				S000128481 Thermoleophilum album; type strain: ATCC 35266 Lineage=Root;rootrank;Bacteria;domain;"Actinobacteria";phylum;Actinobacteria;class;Rubrobacteridae;subclass;Thermoleophilales;order;Thermoleophilaceae;family;Thermoleophilum;genus	83.42	3.00E-94	346
3239	2	6	0	0	0	0	0	1	0	Armatimonadetes				S000128481 Thermoleophilum album; type strain: ATCC 35266 Lineage=Root;rootrank;Bacteria;domain;"Actinobacteria";phylum;Actinobacteria;class;Rubrobacteridae;subclass;Thermoleophilales;order;Thermoleophilaceae;family;Thermoleophilum;genus	83.16	1.00E-92	340
553	0	3	1	0	0	1	1	1	1	Armatimonadetes				S000128481 Thermoleophilum album; type strain: ATCC 35266 Lineage=Root;rootrank;Bacteria;domain;"Actinobacteria";phylum;Actinobacteria;class;Rubrobacteridae;subclass;Thermoleophilales;order;Thermoleophilaceae;family;Thermoleophilum;genus	83.72	9.00E-100	364
3204	0	0	0	0	0	0	1	0	0	Armatimonadetes				S000128481 Thermoleophilum album; type strain: ATCC 35266 Lineage=Root;rootrank;Bacteria;domain;"Actinobacteria";phylum;Actinobacteria;class;Rubrobacteridae;subclass;Thermoleophilales;order;Thermoleophilaceae;family;Thermoleophilum;genus	83.46	1.00E-98	361
2883	0	0	0	2	1	0	0	1	2	Armatimonadetes				S000550678 Moorella thermoacetica; AMP Lineage=Root;rootrank;Bacteria;domain;Firmicutes;phylum;Clostridia;class;Thermoanaerobacterales;order;Thermoanaerobacteraceae;family;Moorella;genus	87.2	5.00E-117	422
2077	0	0	0	0	0	0	0	1	0	Armatimonadetes				S000550678 Moorella thermoacetica; AMP Lineage=Root;rootrank;Bacteria;domain;Firmicutes;phylum;Clostridia;class;Thermoanaerobacterales;order;Thermoanaerobacteraceae;family;Moorella;genus	85.53	2.00E-112	407
1520	0	1	0	0	1	0	0	0	1	Armatimonadetes				S000569921 Pelotomaculum isophthalicicum (T); JI Lineage=Root;rootrank;Bacteria;domain;Firmicutes;phylum;Clostridia;class;Clostridiales;order;Peptococcaceae 2;family;Pelotomaculum;genus	87.8	3.00E-119	429
526	0	0	0	0	2	0	0	0	0	Armatimonadetes				S000721549 Gracilibacter thermotolerans; JW/YJL-S1; 5 Lineage=Root;rootrank;Bacteria;domain;Firmicutes;phylum;Clostridia;class;Clostridiales;order;Gracilibacteraceae;family;Gracilibacter;genus	86.51	5.00E-112	405
3062	0	0	0	0	0	0	0	1	0	Armatimonadetes				S000752077 Moorella perchloratireducens; An10 Lineage=Root;rootrank;Bacteria;domain;Firmicutes;phylum;Clostridia;class;Thermoanaerobacterales;order;Thermoanaerobacteraceae;family;Moorella;genus	86.77	7.00E-121	435
1304	22	59	22	2	14	5	8	26	15	Armatimonadetes				S000841843 Rubrobacter taiwanensis (T); LS-293 Lineage=Root;rootrank;Bacteria;domain;"Actinobacteria";phylum;Actinobacteria;class;Rubrobacteridae;subclass;Rubrobacterales;order;"Rubrobacterineae";suborder;Rubrobacteraceae;family;Rubrobacter;genus	87.67	2.00E-120	433
2452	0	0	0	4	1	0	1	0	2	Armatimonadetes				S000870713 Calditerricola yamamurae (T); YMO722 Lineage=Root;rootrank;Bacteria;domain;Firmicutes;phylum;Bacilli;class;Bacillales;order;Bacillales_incertae_sedis;family;Calditerricola;genus	83.69	1.00E-93	344
2312	1	0	1	0	1	0	0	0	0	Armatimonadetes				S000870713 Calditerricola yamamurae (T); YMO722 Lineage=Root;rootrank;Bacteria;domain;Firmicutes;phylum;Bacilli;class;Bacillales;order;Bacillales_incertae_sedis;family;Calditerricola;genus	88.04	1.00E-128	460
1515	0	0	0	0	0	0	1	0	1	Armatimonadetes				S000870713 Calditerricola yamamurae (T); YMO722 Lineage=Root;rootrank;Bacteria;domain;Firmicutes;phylum;Bacilli;class;Bacillales;order;Bacillales_incertae_sedis;family;Calditerricola;genus	86.99	2.00E-121	436
1780	0	0	0	1	2	6	5	3	5	Armatimonadetes				S001152076 Thermaerobacter composti (T); Ni80 Lineage=Root;rootrank;Bacteria;domain;Firmicutes;phylum;Clostridia;class;Clostridiales;order;Clostridiales_Incertae Sedis XVII;family;Thermaerobacter;genus	86.7	7.00E-121	435
2219	0	0	0	4	2	0	0	0	0	Armatimonadetes				S001577623 Fimbriimonas ginsengisoli Gsoil 348 Lineage=Root;rootrank;Bacteria;domain;"Armatimonadetes";phylum;Armatimonadetes_gp5;genus	87.94	5.00E-122	438
2553	0	0	0	1	0	0	0	0	0	Armatimonadetes				S001577623 Fimbriimonas ginsengisoli Gsoil 348 Lineage=Root;rootrank;Bacteria;domain;"Armatimonadetes";phylum;Armatimonadetes_gp5;genus	88.19	3.00E-129	462
1354	1	3	3	0	0	4	6	1	0	Armatimonadetes				S002350110 Thermaerobacter marianensis DSM 12885 Lineage=Root;rootrank;Bacteria;domain;Firmicutes;phylum;Clostridia;class;Clostridiales;order;Clostridiales_Incertae Sedis XVII;family;Thermaerobacter;genus	86.51	1.00E-118	427
3069	0	0	0	1	0	0	1	0	0	Armatimonadetes				S002350110 Thermaerobacter marianensis DSM 12885 Lineage=Root;rootrank;Bacteria;domain;Firmicutes;phylum;Clostridia;class;Clostridiales;order;Clostridiales_Incertae Sedis XVII;family;Thermaerobacter;genus	84.76	2.00E-100	366
140	0	0	2	1	0	2	0	0	1	Armatimonadetes				S003259708 Thermomicrobium roseum; P-2 Lineage=Root;rootrank;Bacteria;domain;"Chloroflexi";phylum;Thermomicrobia;class;Thermomicrobiales;order;Thermomicrobiaceae;family;Thermomicrobium;genus	83.08	2.00E-96	353

2829	0	0	0	1	0	0	0	0	0	Armatimonadetes					S003287421 Amycolatopsis mediterranei S699 Lineage=Root;rootrank;Bacteria;domain;"Actinobacteria";phylum;Actinobacteria;class;Actinobacteridae;subclass;Actinomycetales;order;Pseudonocardineae;suborder;Pseudonocardiaceae;family;Amycolatopsis;genus	80.81	2.00E-81	303
1853	0	0	0	0	0	0	1	1	0	Bacteroidetes				CandidatusAmoebophilus	S001548608 Candidatus Amoebophilus asiaticus Lineage=Root;rootrank;Bacteria;domain;"Bacteroidetes";phylum;"Sphingobacteria";class;"Sphingobacteriales";order;unclassified_"Sphingobacteriales";	96.63	0	641
152	0	0	0	0	1	0	0	0	0	Bacteroidetes	Bacteroidia	Bacteroidales	Rikenellaceae		S002165119 Alistipes onderdonkii; JCM 16771 Lineage=Root;rootrank;Bacteria;domain;"Bacteroidetes";phylum;"Bacteroidia";class;"Bacteroidales";order;"Rikenellaceae";family;Alistipes;genus	92.01	1.00E-153	544
2638	2	3	1	2	0	0	0	3	3	Bacteroidetes	Flavobacteria				S002968465 Owenweeksia hongkongensis DSM 17368 Lineage=Root;rootrank;Bacteria;domain;"Bacteroidetes";phylum;Flavobacteria;class;"Flavobacteriales";order;Cryomorphaceae;family;Owenweeksia;genus	88.69	2.00E-132	473
1090	0	0	0	0	0	0	0	1	4	Bacteroidetes	Flavobacteria				S002968465 Owenweeksia hongkongensis DSM 17368 Lineage=Root;rootrank;Bacteria;domain;"Bacteroidetes";phylum;Flavobacteria;class;"Flavobacteriales";order;Cryomorphaceae;family;Owenweeksia;genus	88.46	5.00E-122	438
2518	0	0	0	2	0	0	0	0	0	Bacteroidetes	Flavobacteria				S002968465 Owenweeksia hongkongensis DSM 17368 Lineage=Root;rootrank;Bacteria;domain;"Bacteroidetes";phylum;Flavobacteria;class;"Flavobacteriales";order;Cryomorphaceae;family;Owenweeksia;genus	97.93	0	671
1175	0	0	0	0	1	0	0	0	0	Bacteroidetes	Flavobacteria				S002968465 Owenweeksia hongkongensis DSM 17368 Lineage=Root;rootrank;Bacteria;domain;"Bacteroidetes";phylum;Flavobacteria;class;"Flavobacteriales";order;Cryomorphaceae;family;Owenweeksia;genus	94.34	1.00E-168	593
1296	0	0	0	0	1	0	0	0	0	Bacteroidetes	Flavobacteria				S002968465 Owenweeksia hongkongensis DSM 17368 Lineage=Root;rootrank;Bacteria;domain;"Bacteroidetes";phylum;Flavobacteria;class;"Flavobacteriales";order;Cryomorphaceae;family;Owenweeksia;genus	89.95	4.00E-133	475
410	0	0	1	1	0	0	0	0	0	Bacteroidetes	Flavobacteria				S003264146 Wandonia haliotis; NBRC 105642 Lineage=Root;rootrank;Bacteria;domain;"Bacteroidetes";phylum;Flavobacteria;class;"Flavobacteriales";order;Cryomorphaceae;family;Wandonia;genus	90.72	9.00E-145	514
1190	5	0	0	0	0	3	0	0	0	Bacteroidetes	Flavobacteria	Flavobacteriales	Cryomorphaceae		S000712476 Brumimicrobium mesophilum; YH207 Lineage=Root;rootrank;Bacteria;domain;"Bacteroidetes";phylum;Flavobacteria;class;"Flavobacteriales";order;Cryomorphaceae;family;Brumimicrobium;genus	93.75	2.00E-156	553
2268	0	0	0	0	0	0	0	0	1	Bacteroidetes	Flavobacteria	Flavobacteriales	Cryomorphaceae		S000966625 Lishizhenia tianjinensis (T); H6 Lineage=Root;rootrank;Bacteria;domain;"Bacteroidetes";phylum;Flavobacteria;class;"Flavobacteriales";order;Cryomorphaceae;family;Lishizhenia;genus	94.29	8.00E-160	564
720	0	0	0	0	0	0	0	1	0	Bacteroidetes	Flavobacteria	Flavobacteriales	Cryomorphaceae		S002235745 Salinirepens amamiensis; NBRC 101268 Lineage=Root;rootrank;Bacteria;domain;"Bacteroidetes";phylum;Flavobacteria;class;"Flavobacteriales";order;Cryomorphaceae;family;unclassified_Cryomorphaceae;	96.41	0	640
3179	0	0	0	0	0	0	0	0	1	Bacteroidetes	Flavobacteria	Flavobacteriales	Flavobacteriaceae		S000374973 Yeosuana yeosuensis; NC1-5 Lineage=Root;rootrank;Bacteria;domain;"Bacteroidetes";phylum;Flavobacteria;class;"Flavobacteriales";order;Flavobacteriaceae;family;Yeosuana;genus	95.03	6.00E-161	568
684	0	0	0	0	0	0	2	0	1	Bacteroidetes	Flavobacteria	Flavobacteriales	Flavobacteriaceae		S000980065 Lutaonella thermophila (T); CC-MHSW-2 Lineage=Root;rootrank;Bacteria;domain;"Bacteroidetes";phylum;Flavobacteria;class;"Flavobacteriales";order;Flavobacteriaceae;family;Lutaonella;genus	91.56	1.00E-152	540
909	0	0	0	16	0	0	0	7	1	Bacteroidetes	Flavobacteria	Flavobacteriales	Flavobacteriaceae	Arenibacter	S000368220 Arenibacter certesii (T); KMM 3941 Lineage=Root;rootrank;Bacteria;domain;"Bacteroidetes";phylum;Flavobacteria;class;"Flavobacteriales";order;Flavobacteriaceae;family;Arenibacter;genus	98.91	0	658
1820	0	0	0	2	0	0	0	0	0	Bacteroidetes	Flavobacteria	Flavobacteriales	Flavobacteriaceae	Arenibacter	S003284963 Maribacter polysiphoniae; bI1 Lineage=Root;rootrank;Bacteria;domain;"Bacteroidetes";phylum;Flavobacteria;class;"Flavobacteriales";order;Flavobacteriaceae;family;Maribacter;genus	93.59	2.00E-165	582
3258	0	0	0	0	0	0	0	1	0	Bacteroidetes	Flavobacteria	Flavobacteriales	Flavobacteriaceae	Flavobacterium	S000979969 Myroides profundi (T); D25 Lineage=Root;rootrank;Bacteria;domain;"Bacteroidetes";phylum;Flavobacteria;class;"Flavobacteriales";order;Flavobacteriaceae;family;Myroides;genus	90.38	4.00E-133	475
2963	0	0	0	0	0	0	1	0	0	Bacteroidetes	Flavobacteria	Flavobacteriales	Flavobacteriaceae	Flavobacterium	S001169952 Arenibacter nanhaiticus (T); NH36A Lineage=Root;rootrank;Bacteria;domain;"Bacteroidetes";phylum;Flavobacteria;class;"Flavobacteriales";order;Flavobacteriaceae;family;Arenibacter;genus	90.41	1.00E-133	477
3142	0	0	0	1	0	0	0	0	0	Bacteroidetes	Flavobacteria	Flavobacteriales	Flavobacteriaceae	Flavobacterium	S002409094 Myroides odoratimimus; RHH50 Lineage=Root;rootrank;Bacteria;domain;"Bacteroidetes";phylum;Flavobacteria;class;"Flavobacteriales";order;Flavobacteriaceae;family;Myroides;genus	90.75	2.00E-145	516
61	0	0	0	0	0	1	0	0	0	Bacteroidetes	Flavobacteria	Flavobacteriales	Flavobacteriaceae	Flavobacterium	S002967422 Imtechella halotolerans K1; type strain: K1 Lineage=Root;rootrank;Bacteria;domain;"Bacteroidetes";phylum;Flavobacteria;class;"Flavobacteriales";order;Flavobacteriaceae;family;unclassified_Flavobacteriaceae;	89.34	1.00E-128	460
3152	0	0	0	1	0	1	0	0	0	Bacteroidetes	Flavobacteria	Flavobacteriales	Flavobacteriaceae	Flavobacterium	S003263702 Myroides pelagicus; NBRC 102469 Lineage=Root;rootrank;Bacteria;domain;"Bacteroidetes";phylum;Flavobacteria;class;"Flavobacteriales";order;Flavobacteriaceae;family;Myroides;genus	90.54	4.00E-143	508
3129	0	1	0	0	0	0	0	2	0	Bacteroidetes	Flavobacteria	Flavobacteriales	Flavobacteriaceae	Gillisia	S003263093 Gillisia mitskevichiae; NBRC 100590 Lineage=Root;rootrank;Bacteria;domain;"Bacteroidetes";phylum;Flavobacteria;class;"Flavobacteriales";order;Flavobacteriaceae;family;Gillisia;genus	99.18	0	662
2572	0	0	0	4	1	0	1	0	4	Bacteroidetes	Flavobacteria	Flavobacteriales	Flavobacteriaceae	Zhouia	S003719661 Joostella marina; LAMA 908 Lineage=Root;rootrank;Bacteria;domain;"Bacteroidetes";phylum;Flavobacteria;class;"Flavobacteriales";order;Flavobacteriaceae;family;Joostella;genus	94.57	2.00E-161	569
1281	0	0	0	1	0	0	0	0	0	Bacteroidetes	Flavobacteria	Flavobacteriales	Flavobacteriaceae		S000390145 abyssal strain AIII4 Lineage=Root;rootrank;Bacteria;domain;"Bacteroidetes";phylum;Flavobacteria;class;"Flavobacteriales";order;Flavobacteriaceae;family;Gillisia;genus	93.78	2.00E-156	553
2772	41	9	0	1	0	1	0	1	0	Bacteroidetes	Flavobacteria	Flavobacteriales	Flavobacteriaceae		S000434565 Aequorivita lipolytica (T); Y10-2T Lineage=Root;rootrank;Bacteria;domain;"Bacteroidetes";phylum;Flavobacteria;class;"Flavobacteriales";order;Flavobacteriaceae;family;Aequorivita;genus	89.04	1.00E-128	460
468	0	1	1	0	0	0	0	0	0	Bacteroidetes	Flavobacteria	Flavobacteriales	Flavobacteriaceae		S000434565 Aequorivita lipolytica (T); Y10-2T Lineage=Root;rootrank;Bacteria;domain;"Bacteroidetes";phylum;Flavobacteria;class;"Flavobacteriales";order;Flavobacteriaceae;family;Aequorivita;genus	87.44	7.00E-126	451
2129	0	0	0	0	0	0	0	1	0	Bacteroidetes	Flavobacteria	Flavobacteriales	Flavobacteriaceae		S000434565 Aequorivita lipolytica (T); Y10-2T Lineage=Root;rootrank;Bacteria;domain;"Bacteroidetes";phylum;Flavobacteria;class;"Flavobacteriales";order;Flavobacteriaceae;family;Aequorivita;genus	87.12	4.00E-123	442
3044	0	0	0	4	1	0	0	4	33	Bacteroidetes	Flavobacteria	Flavobacteriales	Flavobacteriaceae		S000606018 Pseudozobellia thermophila (T); KMM 3531 Lineage=Root;rootrank;Bacteria;domain;"Bacteroidetes";phylum;Flavobacteria;class;"Flavobacteriales";order;Flavobacteriaceae;family;Pseudozobellia;genus	92.45	3.00E-149	529
2269	0	0	0	0	0	0	0	0	1	Bacteroidetes	Flavobacteria	Flavobacteriales	Flavobacteriaceae		S000610435 Aequorivita antarctica; S4-8 Lineage=Root;rootrank;Bacteria;domain;"Bacteroidetes";phylum;Flavobacteria;class;"Flavobacteriales";order;Flavobacteriaceae;family;Aequorivita;genus	90.93	2.00E-141	503
244	0	0	2	2	3	0	0	7	0	Bacteroidetes	Flavobacteria	Flavobacteriales	Flavobacteriaceae		S000610465 Maribacter arcticus (T); KOPRI 20941 Lineage=Root;rootrank;Bacteria;domain;"Bacteroidetes";phylum;Flavobacteria;class;"Flavobacteriales";order;Flavobacteriaceae;family;Maribacter;genus	91.03	6.00E-141	501
3020	0	0	0	1	0	0	0	11	3	Bacteroidetes	Flavobacteria	Flavobacteriales	Flavobacteriaceae		S000980087 Aequorivita capsosiphonis (T); A71 Lineage=Root;rootrank;Bacteria;domain;"Bacteroidetes";phylum;Flavobacteria;class;"Flavobacteriales";order;Flavobacteriaceae;family;Aequorivita;genus	90.91	6.00E-141	501
177	0	0	0	1	0	0	0	0	0	Bacteroidetes	Flavobacteria	Flavobacteriales	Flavobacteriaceae		S001020264 Muriicola jejuensis (T); EM44 Lineage=Root;rootrank;Bacteria;domain;"Bacteroidetes";phylum;Flavobacteria;class;"Flavobacteriales";order;Flavobacteriaceae;family;Muriicola;genus	95.17	6.00E-166	584
1831	2	0	7	2	2	0	4	105	177	Bacteroidetes	Flavobacteria	Flavobacteriales	Flavobacteriaceae		S002290219 Gramella forsetii KT0803 Lineage=Root;rootrank;Bacteria;domain;"Bacteroidetes";phylum;Flavobacteria;class;"Flavobacteriales";order;Flavobacteriaceae;family;Gramella;genus	90.91	2.00E-140	499
1047	0	0	0	0	0	1	0	0	0	Bacteroidetes	Flavobacteria	Flavobacteriales	Flavobacteriaceae		S002290219 Gramella forsetii KT0803 Lineage=Root;rootrank;Bacteria;domain;"Bacteroidetes";phylum;Flavobacteria;class;"Flavobacteriales";order;Flavobacteriaceae;family;Gramella;genus	90.21	2.00E-136	486
2112	0	0	0	0	0	1	0	0	0	Bacteroidetes	Flavobacteria	Flavobacteriales	Flavobacteriaceae		S002967422 Imtechella halotolerans K1; type strain: K1 Lineage=Root;rootrank;Bacteria;domain;"Bacteroidetes";phylum;Flavobacteria;class;"Flavobacteriales";order;Flavobacteriaceae;family;unclassified_Flavobacteriaceae;	92.27	9.00E-155	547
719	4	18	2	0	0	4	9	9	3	Bacteroidetes	Flavobacteria	Flavobacteriales	Flavobacteriaceae		S003263006 Vitellibacter vladivostokensis; NBRC 16718 Lineage=Root;rootrank;Bacteria;domain;"Bacteroidetes";phylum;Flavobacteria;class;"Flavobacteriales";order;Flavobacteriaceae;family;Vitellibacter;genus	87.66	2.00E-120	433
2354	0	0	2	45	4	0	0	0	0	Bacteroidetes	Flavobacteria	Flavobacteriales	Flavobacteriaceae		S003263006 Vitellibacter vladivostokensis; NBRC 16718 Lineage=Root;rootrank;Bacteria;domain;"Bacteroidetes";phylum;Flavobacteria;class;"Flavobacteriales";order;Flavobacteriaceae;family;Vitellibacter;genus	91.18	4.00E-143	508
424	0	0	1	0	0	0	0	0	0	Bacteroidetes	Flavobacteria	Flavobacteriales	Flavobacteriaceae		S003263006 Vitellibacter vladivostokensis; NBRC 16718 Lineage=Root;rootrank;Bacteria;domain;"Bacteroidetes";phylum;Flavobacteria;class;"Flavobacteriales";order;Flavobacteriaceae;family;Vitellibacter;genus	91.98	1.00E-147	523
2334	1	2	2	5	0	0	0	0	0	Bacteroidetes	Flavobacteria	Flavobacteriales	Flavobacteriaceae		S003263383 Sediminibacter furfurosus; NBRC 101622 Lineage=Root;rootrank;Bacteria;domain;"Bacteroidetes";phylum;Flavobacteria;class;"Flavobacteriales";order;Flavobacteriaceae;family;Sediminibacter;genus	89.95	3.00E-134	479
1388	0	0	1	0	0	0	0	0	0	Bacteroidetes	Flavobacteria	Flavobacteriales	Flavobacteriaceae		S003263383 Sediminibacter furfurosus; NBRC 101622 Lineage=Root;rootrank;Bacteria;domain;"Bacteroidetes";phylum;Flavobacteria;class;"Flavobacteriales";order;Flavobacteriaceae;family;Sediminibacter;genus	89.17	2.00E-136	486
2676	0	0	0	1	0	0	0	0	0	Bacteroidetes	Flavobacteria	Flavobacteriales	Flavobacteriaceae		S003284963 Maribacter polysiphoniae; bI1 Lineage=Root;rootrank;Bacteria;domain;"Bacteroidetes";phylum;Flavobacteria;class;"Flavobacteriales";order;Flavobacteriaceae;family;Maribacter;genus	93.72	8.00E-155	547
1319	0	0	0	1	1	0	0	0	0	Bacteroidetes	Flavobacteria	Flavobacteriales	Flavobacteriaceae		S003284979 Ulvibacter antarcticus; p8 Lineage=Root;rootrank;Bacteria;domain;"Bacteroidetes";phylum;Flavobacteria;class;"Flavobacteriales";order;Flavobacteriaceae;family;Ulvibacter;genus	90.82	2.00E-146	520
790	0	0	0	3	1	0	0	14	57	Bacteroidetes	Flavobacteria	Flavobacteriales	Flavobacteriaceae		S003610731 Vitellibacter aestuarii (T); JC2436 Lineage=Root;rootrank;Bacteria;domain;"Bacteroidetes";phylum;Flavobacteria;class;"Flavobacteriales";order;Flavobacteriaceae;family;Vitellibacter;genus	91.22	4.00E-143	508
0	1	0	0	0	0	0	0	0	0	Bacteroidetes	Flavobacteria	Flavobacteriales	Flavobacteriaceae		S003610731 Vitellibacter aestuarii (T); JC2436 Lineage=Root;rootrank;Bacteria;domain;"Bacteroidetes";phylum;Flavobacteria;class;"Flavobacteriales";order;Flavobacteriaceae;family;Vitellibacter;genus	83.82	2.00E-96	353
838	1	0	0	0	0	0	0	0	0	Bacteroidetes	Flavobacteria	Flavobacteriales	Flavobacteriaceae		S003610731 Vitellibacter aestuarii (T); JC2436 Lineage=Root;rootrank;Bacteria;domain;"Bacteroidetes";phylum;Flavobacteria;class;"Flavobacteriales";order;Flavobacteriaceae;family;Vitellibacter;genus	87.78	4.00E-128	459
2294	0	0	0	0	0	0	1	0	0	Bacteroidetes	Flavobacteria	Flavobacteriales	Flavobacteriaceae		S003610731 Vitellibacter aestuarii (T); JC2436 Lineage=Root;rootrank;Bacteria;domain;"Bacteroidetes";phylum;Flavobacteria;class;"Flavobacteriales";order;Flavobacteriaceae;family;Vitellibacter;genus	86.77	9.00E-115	414
1636	1	0	0	0	0	2	2	0	0	Bacteroidetes	Flavobacteria	Flavobacteriales	Flavobacteriaceae		S003719310 Maribacter aquivivus; 15BN09U-1 Lineage=Root;rootrank;Bacteria;domain;"Bacteroidetes";phylum;Flavobacteria;class;"Flavobacteriales";order;Flavobacteriaceae;family;Maribacter;genus	88.86	4.00E-128	459
2947	0	9	0	0	0	0	0	10	1	Bacteroidetes	Flavobacteria	Flavobacteriales			S000335575 Lishizhenia caseinilytica (T); UST040201-001 Lineage=Root;rootrank;Bacteria;domain;"Bacteroidetes";phylum;Flavobacteria;class;"Flavobacteriales";order;Cryomorphaceae;family;Lishizhenia;genus	87.6	1.00E-117	424
1042	0	0	0	1	0	0	0	0	0	Bacteroidetes	Sphingobacteria	Sphingobacteriales			S000539145 Tuber borchii symbiont b-1BO Lineage=Root;rootrank;Bacteria;domain;"Bacteroidetes";phylum;unclassified_"Bacteroidetes";	92.16	2.00E-146	520
939	0	0	1	0	0	0	0	7	6	Bacteroidetes	Sphingobacteria	Sphingobacteriales			S000652883 Cytophaga arvensicola; Scyt01 Lineage=Root;rootrank;Bacteria;domain;"Bacteroidetes";phylum;"Sphingobacteria";class;"Sphingobacteriales";order;Chitinophagaceae;family;Chitinophaga;genus	91.28	6.00E-141	501
2923	0	0	0	1	0	0	0	1	0	Bacteroidetes	Sphingobacteria	Sphingobacteriales			S000652883 Cytophaga arvensicola; Scyt01 Lineage=Root;rootrank;Bacteria;domain;"Bacteroidetes";phylum;"Sphingobacteria";class;"Sphingobacteriales";order;Chitinophagaceae;family;Chitinophaga;genus	90.7	2.00E-145	516
1276	0	0	0	1	0	0	0	0	0	Bacteroidetes	Sphingobacteria	Sphingobacteriales			S001153623 Solitalea koreensis (T); R2A36-4 Lineage=Root;rootrank;Bacteria;domain;"Bacteroidetes";phylum;"Sphingobacteria";class;"Sphingobacteriales";order;Sphingobacteriaceae;family;Solitalea;genus	89.7	2.00E-131	470
2695	0	0	0	0	0	0	0	1	0	Bacteroidetes	Sphingobacteria	Sphingobacteriales			S003262899 Chitinophaga pinensis; NBRC 15968 Lineage=Root;rootrank;Bacteria;domain;"Bacteroidetes";phylum;"Sphingobacteria";class;"Sphingobacteriales";order;Chitinophagaceae;family;Chitinophaga;genus	90.08	5.00E-132	472
806	0	0	0	1	0	0	0	0	0	Bacteroidetes	Sphingobacteria	Sphingobacteriales			S003262934 Chitinophaga japonensis; NBRC 16041 Lineage=Root;rootrank;Bacteria;domain;"Bacteroidetes";phylum;"Sphingobacteria";class;"Sphingobacteriales";order;Chitinophagaceae;family;Chitinophaga;genus	86.15	7.00E-106	385
1280	0	0	0	1	0	0	0	0	0	Bacteroidetes	Sphingobacteria	Sphingobacteriales			S003264243 Flavihumibacter petaseus; NBRC 106054 Lineage=Root;rootrank;Bacteria;domain;"Bacteroidetes";phylum;"Sphingobacteria";class;"Sphingobacteriales";order;Chitinophagaceae;family;Flavihumibacter;genus	89.13	4.00E-128	459
814	0	0	0	1	0	1	0	0	0	Bacteroidetes	Sphingobacteria	Sphingobacteriales			S003279232 Hydrotalea flava; FJLY16 Lineage=Root;rootrank;Bacteria;domain;"Bacteroidetes";phylum;"Sphingobacteria";class;"Sphingobacteriales";order;Chitinophagaceae;family;unclassified_Chitinophagaceae;	89.53	1.00E-128	460
2092	0	0	0	2	0	0	0	0	1	Bacteroidetes	Sphingobacteria	Sphingobacteriales			S000650534 Terrimonas lutea (T); DY Lineage=Root;rootrank;Bacteria;domain;"Bacteroidetes";phylum;"Sphingobacteria";class;"Sphingobacteriales";order;Chitinophagaceae;family;Terrimonas;genus	97.83	0	634
1612	781	107	2	12	0	15	3	5	0	Bacteroidetes	Sphingobacteria	Sphingobacteriales	Balneolaceae		S000594445 Balneola vulgaris (T); 13IX/A01/164 Lineage=Root;rootrank;Bacteria;domain;"Bacteroidetes";phylum;"Sphingobacteria";class;"Sphingobacteriales";order;Chitinophagaceae;family;Balneola;genus	83.57	2.00E-90	333
2978	276	733	362	6	13	118	276	276	81	Bacteroidetes	Sphingobacteria	Sphingobacteriales	Balneolaceae		S003710953 Gracilimonas tropica (T); CL-CB462 Lineage=Root;rootrank;Bacteria;domain;"Bacteroidetes";phylum;"Sphingobacteria";class;"Sphingobacteriales";order;Chitinophagaceae;family;Gracilimonas;genus	88.8	2.00E-121	436
1144	308	486	285	1	36	218	324	103	146	Bacteroidetes	Sphingobacteria	Sphingobacteriales	Balneolaceae		S003710953 Gracilimonas tropica (T); CL-CB462 Lineage=Root;rootrank;Bacteria;domain;"Bacteroidetes";phylum;"Sphingobacteria";class;"Sphingobacteriales";order;Chitinophagaceae;family;Gracilimonas;genus	92.16	2.00E-141	503
919	23	144	71	4	42	83	98	53	32	Bacteroidetes	Sphingobacteria	Sphingobacteriales	Balneolaceae		S003710953 Gracilimonas tropica (T); CL-CB462 Lineage=Root;rootrank;Bacteria;domain;"Bacteroidetes";phylum;"Sphingobacteria";class;"Sphingobacteriales";order;Chitinophagaceae;family;Gracilimonas;genus	90.53	5.00E-132	472
1485	75	15	0	7	0	12	17	129	94	Bacteroidetes	Sphingobacteria	Sphingobacteriales	Balneolaceae		S003710953 Gracilimonas tropica (T); CL-CB462 Lineage=Root;rootrank;Bacteria;domain;"Bacteroidetes";phylum;"Sphingobacteria";class;"Sphingobacteriales";order;Chitinophagaceae;family;Gracilimonas;genus	90.78	1.00E-132	473
2588	134	0	0	1	0	5	1	1	0	Bacteroidetes	Sphingobacteria	Sphingobacteriales	Balneolaceae		S003710953 Gracilimonas tropica (T); CL-CB462 Lineage=Root;rootrank;Bacteria;domain;"Bacteroidetes";phylum;"Sphingobacteria";class;"Sphingobacteriales";order;Chitinophagaceae;family;Gracilimonas;genus	89.08	4.00E-123	442
1634	112	16	0	2	0	5	1	0	0	Bacteroidetes	Sphingobacteria	Sphingobacteriales	Balneolaceae		S003710953 Gracilimonas tropica (T); CL-CB462 Lineage=Root;rootrank;Bacteria;domain;"Bacteroidetes";phylum;"Sphingobacteria";class;"Sphingobacteriales";order;Chitinophagaceae;family;Gracilimonas;genus	91.34	2.00E-136	486
2118	67	34	0	0	0	4	3	0	0	Bacteroidetes	Sphingobacteria	Sphingobacteriales	Balneolaceae		S003710953 Gracilimonas tropica (T); CL-CB462 Lineage=Root;rootrank;Bacteria;domain;"Bacteroidetes";phylum;"Sphingobacteria";class;"Sphingobacteriales";order;Chitinophagaceae;family;Gracilimonas;genus	90.76	4.00E-133	475
1464	34	11	13	1	6	25	34	7	15	Bacteroidetes	Sphingobacteria	Sphingobacteriales	Balneolaceae		S003710953 Gracilimonas tropica (T); CL-CB462 Lineage=Root;rootrank;Bacteria;domain;"Bacteroidetes";phylum;"Sphingobacteria";class;"Sphingobacteriales";order;Chitinophagaceae;family;Gracilimonas;genus	91.36	5.00E-137	488
1152	22	41	14	0	1	8	6	26	16	Bacteroidetes	Sphingobacteria	Sphingobacteriales	Balneolaceae		S003710953 Gracilimonas tropica (T); CL-CB462 Lineage=Root;rootrank;Bacteria;domain;"Bacteroidetes";phylum;"Sphingobacteria";class;"Sphingobacteriales";order;Chitinophagaceae;family;Gracilimonas;genus	90.48	2.00E-131	470
1599	17	3	0	1	0	2	0	0	0	Bacteroidetes	Sphingobacteria	Sphingobacteriales	Balneolaceae		S003710953 Gracilimonas tropica (T); CL-CB462 Lineage=Root;rootrank;Bacteria;domain;"Bacteroidetes";phylum;"Sphingobacteria";class;"Sphingobacteriales";order;Chitinophagaceae;family;Gracilimonas;genus	84.65	3.00E-104	379
1842	0	1	14	0	0	13	21	0	0	Bacteroidetes	Sphingobacteria	Sphingobacteriales	Balneolaceae		S003710953 Gracilimonas tropica (T); CL-CB462 Lineage=Root;rootrank;Bacteria;domain;"Bacteroidetes";phylum;"Sphingobacteria";class;"Sphingobacteriales";order;Chitinophagaceae;family;Gracilimonas;genus	88.37	3.00E-119	429
900	2	13	1	0	0	1	0	4	0	Bacteroidetes	Sphingobacteria	Sphingobacteriales	Balneolaceae		S003710953 Gracilimonas tropica (T); CL-CB462 Lineage=Root;rootrank;Bacteria;domain;"Bacteroidetes";phylum;"Sphingobacteria";class;"Sphingobacteriales";order;Chitinophagaceae;family;Gracilimonas;genus	86.43	2.00E-106	387
1915	6	1	0	0	0	0	0	0	0	Bacteroidetes	Sphingobacteria	Sphingobacteriales	Balneolaceae		S003710953 Gracilimonas tropica (T); CL-CB462 Lineage=Root;rootrank;Bacteria;domain;"Bacteroidetes";phylum;"Sphingobacteria";class;"Sphingobacteriales";order;Chitinophagaceae;family;Gracilimonas;genus	91.33	2.00E-141	503
2107	6	0	0	0	0	0	0	0	0	Bacteroidetes	Sphingobacteria	Sphingobacteriales	Balneolaceae		S003710953 Gracilimonas tropica (T); CL-CB462 Lineage=Root;rootrank;Bacteria;domain;"Bacteroidetes";phylum;"Sphingobacteria";class;"Sphingobacteriales";order;Chitinophagaceae;family;Gracilimonas;genus	85	4.00E-98	359
3172	0	5	0	0	0	1	0	0	0	Bacteroidetes	Sphingobacteria	Sphingobacteriales	Balneolaceae		S003710953 Gracilimonas tropica (T); CL-CB462 Lineage=Root;rootrank;Bacteria;domain;"Bacteroidetes";phylum;"Sphingobacteria";class;"Sphingobacteriales";order;Chitinophagaceae;family;Gracilimonas;genus	85.4	2.00E-100	366
2139	1	1	0	0	0	0	0	0	0	Bacteroidetes	Sphingobacteria	Sphingobacteriales	Balneolaceae		S003710953 Gracilimonas tropica (T); CL-CB462 Lineage=Root;rootrank;Bacteria;domain;"Bacteroidetes";phylum;"Sphingobacteria";class;"Sphingobacteriales";order;Chitinophagaceae;family;Gracilimonas;genus	84.12	4.00E-93	342
1954	0	0	0	0	0	2	1	0	0	Bacteroidetes	Sphingobacteria	Sphingobacteriales	Balneolaceae		S003710953 Gracilimonas tropica (T); CL-CB462 Lineage=Root;rootrank;Bacteria;domain;"Bacteroidetes";phylum;"Sphingobacteria";class;"Sphingobacteriales";order;Chitinophagaceae;family;Gracilimonas;genus	85.6	2.00E-101	370
1186	0	0	0	0	0	0	1	0	0	Bacteroidetes	Sphingobacteria	Sphingobacteriales	Balneolaceae		S003710953 Gracilimonas tropica (T); CL-CB462 Lineage=Root;rootrank;Bacteria;domain;"Bacteroidetes";phylum;"Sphingobacteria";class;"Sphingobacteriales";order;Chitinophagaceae;family;Gracilimonas;genus	85.38	3.00E-110	399
848	0	0	0	0	0	0	0	0	1	Bacteroidetes	Sphingobacteria	Sphingobacteriales	Balneolaceae		S003710953 Gracilimonas tropica (T); CL-CB462 Lineage=Root;rootrank;Bacteria;domain;"Bacteroidetes";phylum;"Sphingobacteria";class;"Sphingobacteriales";order;Chitinophagaceae;family;Gracilimonas;genus	89.39	2.00E-137	490
1305	0	0	0	0	0	0	0	0	1	Bacteroidetes	Sphingobacteria	Sphingobacteriales	Balneolaceae		S003710953 Gracilimonas tropica (T); CL-CB462 Lineage=Root;rootrank;Bacteria;domain;"Bacteroidetes";phylum;"Sphingobacteria";class;"Sphingobacteriales";order;Chitinophagaceae;family;Gracilimonas;genus	91.05	1.00E-147	523
1798	0	0	14	2	2	4	2	2	3	Bacteroidetes	Sphingobacteria	Sphingobacteriales	Balneolaceae	Balneola	S000893789 Balneola alkaliphila (T); CM41_14b Lineage=Root;rootrank;Bacteria;domain;"Bacteroidetes";phylum;"Sphingobacteria";class;"Sphingobacteriales";order;Chitinophagaceae;family;Balneola;genus	95.88	3.00E-179	628
227	1	0	0	0	0	0	0	23	28	Bacteroidetes	Sphingobacteria	Sphingobacteriales	Balneolaceae	Balneola	S003710953 Gracilimonas tropica (T); CL-CB462 Lineage=Root;rootrank;Bacteria;domain;"Bacteroidetes";phylum;"Sphingobacteria";class;"Sphingobacteriales";order;Chitinophagaceae;family;Gracilimonas;genus	94.59	2.00E-170	599
949	1	0	0	0	0	0	0	0	0	Bacteroidetes	Sphingobacteria	Sphingobacteriales	Balneolaceae	KSA	S002234457 Salinibacter luteus; DGO Lineage=Root;rootrank;Bacteria;domain;"Bacteroidetes";phylum;"Sphingobacteria";class;"Sphingobacteriales";order;"Rhodothermaceae";family;Salinibacter;genus	79.67	4.00E-68	259
318	0	5	0	0	0	3	6	0	0	Bacteroidetes	Sphingobacteria	Sphingobacteriales	Balneolaceae	KSA	S003710953 Gracilimonas tropica (T); CL-CB462 Lineage=Root;rootrank;Bacteria;domain;"Bacteroidetes";phylum;"Sphingobacteria";class;"Sphingobacteriales";order;Chitinophagaceae;family;Gracilimonas;genus	87.63	3.00E-125	449
38	0	1	0	0	0	0	0	0	0	Bacteroidetes	Sphingobacteria	Sphingobacteriales	Cyclobacteriaceae		S001873713 Litoribacter ruber (T); YIM CH208 Lineage=Root;rootrank;Bacteria;domain;"Bacteroidetes";phylum;"Sphingobacteria";class;"Sphingobacteriales";order;Cytophagaceae;family;Litoribacter;genus	99.49	0	706
1003	0	0	5	11	11	0	0	9	13	Bacteroidetes	Sphingobacteria	Sphingobacteriales	Cyclobacteriaceae		S002442579 Rhodonellum psychrophilum; 4-5-25; XJEEM 10320 Lineage=Root;rootrank;Bacteria;domain;"Bacteroidetes";phylum;"Sphingobacteria";class;"Sphingobacteriales";order;Cytophagaceae;family;Litoribacter;genus	94.85	1.00E-163	577
2493	0	0	0	0	0	1	0	0	1	Bacteroidetes	Sphingobacteria	Sphingobacteriales	Cyclobacteriaceae	Algoriphagus	S000575557 Algoriphagus locisalis; MSS-171 Lineage=Root;rootrank;Bacteria;domain;"Bacteroidetes";phylum;"Sphingobacteria";class;"Sphingobacteriales";order;"Cyclobacteriaceae";family;Algoriphagus;genus	96.91	0	651
1924	0	0	0	0	0	0	1	0	0	Bacteroidetes	Sphingobacteria	Sphingobacteriales	Flammeovirgaceae		S000384732 Flexibacter aggregans; IFO 15974 Lineage=Root;rootrank;Bacteria;domain;"Bacteroidetes";phylum;"Sphingobacteria";class;"Sphingobacteriales";order;"Flammeovirgaceae";family;Fulvivirga;genus	91.47	2.00E-150	532
1553	0	0	0	0	2	0	0	3	32	Bacteroidetes	Sphingobacteria	Sphingobacteriales	Flammeovirgaceae		S001239104 Fulvivirga kasyanovii; TISTR1781 Lineage=Root;rootrank;Bacteria;domain;"Bacteroidetes";phylum;"Sphingobacteria";class;"Sphingobacteriales";order;"Flammeovirgaceae";family;Fulvivirga;genus	92.8	1.00E-158	560
730	0	0	1	2	3	0	0	0	10	Bacteroidetes	Sphingobacteria	Sphingobacteriales	Flammeovirgaceae		S002304326 Marivirga tractuosa DSM 4126 Lineage=Root;rootrank;Bacteria;domain;"Bacteroidetes";phylum;"Sphingobacteria";class;"Sphingobacteriales";order;"Flammeovirgaceae";family;Marivirga;genus	92.93	2.00E-151	536
888	0	0	1	1	0	0	1	2	9	Bacteroidetes	Sphingobacteria	Sphingobacteriales	Flammeovirgaceae		S002304326 Marivirga tractuosa DSM 4126 Lineage=Root;rootrank;Bacteria;domain;"Bacteroidetes";phylum;"Sphingobacteria";class;"Sphingobacteriales";order;"Flammeovirgaceae";family;Marivirga;genus	92.12	2.00E-145	516
2274	0	0	0	0	0	0	0	0	1	Bacteroidetes	Sphingobacteria	Sphingobacteriales	Flammeovirgaceae		S002440634 Porifericola rhodea; N5EA6-3A2B Lineage=Root;rootrank;Bacteria;domain;"Bacteroidetes";phylum;"Sphingobacteria";class;"Sphingobacteriales";order;unclassified_"Sphingobacteriales";	93.5	8.00E-155	547
2054	0	4	0	1	0	2	1	1	0	Bacteroidetes	Sphingobacteria	Sphingobacteriales	Flammeovirgaceae		S000384732 Flexibacter aggregans; IFO 15974 Lineage=Root;rootrank;Bacteria;domain;"Bacteroidetes";phylum;"Sphingobacteria";class;"Sphingobacteriales";order;"Flammeovirgaceae";family;Fulvivirga;genus	90.63	2.00E-135	483
2570	0	0	2	1	1	0	0	0	0	Bacteroidetes	Sphingobacteria	Sphingobacteriales	Flammeovirgaceae		S000384732 Flexibacter aggregans; IFO 15974 Lineage=Root;rootrank;Bacteria;domain;"Bacteroidetes";phylum;"Sphingobacteria";class;"Sphingobacteriales";order;"Flammeovirgaceae";family;Fulvivirga;genus	94.85	1.00E-172	606
3021	0	0	0	2	0	0	0	0	1	Bacteroidetes	Sphingobacteria	Sphingobacteriales	Flammeovirgaceae		S000384732 Flexibacter aggregans; IFO 15974 Lineage=Root;rootrank;Bacteria;domain;"Bacteroidetes";phylum;"Sphingobacteria";class;"Sphingobacteriales";order;"Flammeovirgaceae";family;Fulvivirga;genus	95.12	8.00E-165	580
1963	0	0	0	0	0	0	0	0	1	Bacteroidetes	Sphingobacteria	Sphingobacteriales	Flammeovirgaceae		S000384732 Flexibacter aggregans; IFO 15974 Lineage=Root;rootrank;Bacteria;domain;"Bacteroidetes";phylum;"Sphingobacteria";class;"Sphingobacteriales";order;"Flammeovirgaceae";family;Fulvivirga;genus	92.51	2.00E-156	553
1889	1	0	7	24	5	10	23	8	12	Bacteroidetes	Sphingobacteria	Sphingobacteriales	Flammeovirgaceae		S001589379 Adhaeribacter aerophilus (T); 6424S-25 Lineage=Root;rootrank;Bacteria;domain;"Bacteroidetes";phylum;"Sphingobacteria";class;"Sphingobacteriales";order;Cytophagaceae;family;Adhaeribacter;genus	90.79	4.00E-138	492
2789	0	0	0	0	0	0	0	0	2	Bacteroidetes	Sphingobacteria	Sphingobacteriales	Flammeovirgaceae		S002304326 Marivirga tractuosa DSM 4126 Lineage=Root;rootrank;Bacteria;domain;"Bacteroidetes";phylum;"Sphingobacteria";class;"Sphingobacteriales";order;"Flammeovirgaceae";family;Marivirga;genus	100	0	680
1972	0	0	0	0	0	0	0	0	6	Bacteroidetes	Sphingobacteria	Sphingobacteriales	Flammeovirgaceae		S002440634 Porifericola rhodea; N5EA6-3A2B Lineage=Root;rootrank;Bacteria;domain;"Bacteroidetes";phylum;"Sphingobacteria";class;"Sphingobacteriales";order;unclassified_"Sphingobacteriales";	97.02	2.00E-176	619
3267	0	0	1	5	2	0	0	14	25	Bacteroidetes	Sphingobacteria	Sphingobacteriales	Flammeovirgaceae		S003291914 Roseivirga ehrenbergii; HME8272 Lineage=Root;rootrank;Bacteria;domain;"Bacteroidetes";phylum;"Sphingobacteria";class;"Sphingobacteriales";order;"Flammeovirgaceae";family;Roseivirga;genus	90.72	2.00E-145	516
1258	0	0	0	0	1	0	0	2	2	Bacteroidetes	Sphingobacteria	Sphingobacteriales	Flammeovirgaceae		S003619184 Nafulsella turpanensis ZLM-10 Lineage=Root;rootrank;Bacteria;domain;"Bacteroidetes";phylum;"Sphingobacteria";class;"Sphingobacteriales";order;"Flammeovirgaceae";family;unclassified_"Flammeovirgaceae";	96.48	1.00E-173	610
3058	0	0	0	0	1	0	0	0	0	Bacteroidetes	Sphingobacteria	Sphingobacteriales	Flexibacteraceae	Adhaeribacter	S000539472 Adhaeribacter aquaticus (T); type strain: MBRG1.5 Lineage=Root;rootrank;Bacteria;domain;"Bacteroidetes";phylum;"Sphingobacteria";class;"Sphingobacteriales";order;Cytophagaceae;family;Adhaeribacter;genus	97.42	0	658
2008	0	0	4	29	17	6	1	1	0	Bacteroidetes	Sphingobacteria	Sphingobacteriales	Flexibacteraceae	Cytophaga	S001745410 Ohtaekwangia koreensis (T); 3B-2 Lineage=Root;rootrank;Bacteria;domain;"Bacteroidetes";phylum;"Bacteroidetes"_incertae_sedis;class;Ohtaekwangia;genus	95.11	8.00E-165	580
533	0	0	0	3	4	0	0	0	0	Bacteroidetes	Sphingobacteria	Sphingobacteriales	Flexibacteraceae	Cytophaga	S001745410 Ohtaekwangia koreensis (T); 3B-2 Lineage=Root;rootrank;Bacteria;domain;"Bacteroidetes";phylum;"Bacteroidetes"_incertae_sedis;class;Ohtaekwangia;genus	94.86	4.00E-163	575
801	0	0	0	1	0	1	0	1	0	Bacteroidetes	Sphingobacteria	Sphingobacteriales	Flexibacteraceae	Cytophaga	S001745410 Ohtaekwangia koreensis (T); 3B-2 Lineage=Root;rootrank;Bacteria;domain;"Bacteroidetes";phylum;"Bacteroidetes"_incertae_sedis;class;Ohtaekwangia;genus	92.93	2.00E-151	536
343	0	0	0	0	1	0	0	0	0	Bacteroidetes	Sphingobacteria	Sphingobacteriales	Flexibacteraceae	Cytophaga	S001745410 Ohtaekwangia koreensis (T); 3B-2 Lineage=Root;rootrank;Bacteria;domain;"Bacteroidetes";phylum;"Bacteroidetes"_incertae_sedis;class;Ohtaekwangia;genus	86.86	2.00E-111	403
2516	0	0	0	1	0	0	0	0	0	Bacteroidetes	Sphingobacteria	Sphingobacteriales	Flexibacteraceae	Cytophaga	S001745410 Ohtaekwangia koreensis (T); 3B-2 Lineage=Root;rootrank;Bacteria;domain;"Bacteroidetes";phylum;"Bacteroidetes"_incertae_sedis;class;Ohtaekwangia;genus	92.31	7.00E-156	551
2422	0	0	0	1	0	3	0	0	0	Bacteroidetes	Sphingobacteria	Sphingobacteriales	Flexibacteraceae	Cytophaga	S001745411 Ohtaekwangia kribbensis (T); 10AO Lineage=Root;rootrank;Bacteria;domain;"Bacteroidetes";phylum;"Bacteroidetes"_incertae_sedis;class;Ohtaekwangia;genus	93.22	4.00E-153	542
1293	0	1	0	0	0	0	0	0	0	Bacteroidetes	Sphingobacteria	Sphingobacteriales	Flexibacteraceae	Dyadobacter	S003289752 Dyadobacter fermentans; WS17 Lineage=Root;rootrank;Bacteria;domain;"Bacteroidetes";phylum;"Sphingobacteria";class;"Sphingobacteriales";order;Cytophagaceae;family;Dyadobacter;genus	98.45	0	684
1937	0	0	0	0	0	0	0	1	0	Bacteroidetes	Sphingobacteria	Sphingobacteriales	Flexibacteraceae	Hymenobacter	S002167664 Hymenobacter rigui; A1-12 Lineage=Root;rootrank;Bacteria;domain;"Bacteroidetes";phylum;"Sphingobacteria";class;"Sphingobacteriales";order;Cytophagaceae;family;Hymenobacter;genus	98.37	0	647
2770	0	0	0	0	0	0	0	0	1	Bacteroidetes	Sphingobacteria	Sphingobacteriales	Flexibacteraceae	Hymenobacter	S002167664 Hymenobacter rigui; A1-12 Lineage=Root;rootrank;Bacteria;domain;"Bacteroidetes";phylum;"Sphingobacteria";class;"Sphingobacteriales";order;Cytophagaceae;family;Hymenobacter;genus	91.21	1.00E-148	527
743	0	0	0	0	0	0	0	0	2	Bacteroidetes	Sphingobacteria	Sphingobacteriales	Flexibacteraceae		S000825311 Pedobacter terricola (T); DS-45 Lineage=Root;rootrank;Bacteria;domain;"Bacteroidetes";phylum;"Sphingobacteria";class;"Sphingobacteriales";order;Sphingobacteriaceae;family;Pedobacter;genus	81.07	6.00E-82	305
3194	0	0	0	11	4	3	1	1	1	Bacteroidetes	Sphingobacteria	Sphingobacteriales	Flexibacteraceae		S001745410 Ohtaekwangia koreensis (T); 3B-2 Lineage=Root;rootrank;Bacteria;domain;"Bacteroidetes";phylum;"Bacteroidetes"_incertae_sedis;class;Ohtaekwangia;genus	89.8	1.00E-138	494
1064	0	0	0	0	1	0	0	0	0	Bacteroidetes	Sphingobacteria	Sphingobacteriales	Flexibacteraceae		S003264049 Adhaeribacter terreus; NBRC 104235 Lineage=Root;rootrank;Bacteria;domain;"Bacteroidetes";phylum;"Sphingobacteria";class;"Sphingobacteriales";order;Cytophagaceae;family;Adhaeribacter;genus	97.29	4.00E-178	625
2975	0	0	0	0	0	0	0	0	1	Bacteroidetes	Sphingobacteria	Sphingobacteriales	ML65J-4		S000893789 Balneola alkaliphila (T); CM41_14b Lineage=Root;rootrank;Bacteria;domain;"Bacteroidetes";phylum;"Sphingobacteria";class;"Sphingobacteriales";order;Chitinophagaceae;family;Balneola;genus	86.65	3.00E-119	429
48	0	0	1	3	5	1	2	7	8	Bacteroidetes	Sphingobacteria	Sphingobacteriales	Rhodothermaceae		S001351089 Rhodothermus profundi (T); PRI2902 Lineage=Root;rootrank;Bacteria;domain;"Bacteroidetes";phylum;"Sphingobacteria";class;"Sphingobacteriales";order;"Rhodothermaceae";family;Rhodothermus;genus	88.95	3.00E-134	479
1139	0	0	0	0	0	0	0	0	4	Bacteroidetes	Sphingobacteria	Sphingobacteriales	Rhodothermaceae		S001351089 Rhodothermus profundi (T); PRI2902 Lineage=Root;rootrank;Bacteria;domain;"Bacteroidetes";phylum;"Sphingobacteria";class;"Sphingobacteriales";order;"Rhodothermaceae";family;Rhodothermus;genus	88.8	3.00E-124	446
1921	0	0	0	0	1	0	0	0	0	Bacteroidetes	Sphingobacteria	Sphingobacteriales	Rhodothermaceae		S001351089 Rhodothermus profundi (T); PRI2902 Lineage=Root;rootrank;Bacteria;domain;"Bacteroidetes";phylum;"Sphingobacteria";class;"Sphingobacteriales";order;"Rhodothermaceae";family;Rhodothermus;genus	89	9.00E-135	481
2901	1	0	4	1	2	0	0	0	2	Bacteroidetes	Sphingobacteria	Sphingobacteriales	Rhodothermaceae		S002034283 Salisaeta longa; LYG10 Lineage=Root;rootrank;Bacteria;domain;"Bacteroidetes";phylum;"Sphingobacteria";class;"Sphingobacteriales";order;"Rhodothermaceae";family;Salisaeta;genus	90.57	1.00E-137	490
2970	0	0	0	1	1	0	0	4	4	Bacteroidetes	Sphingobacteria	Sphingobacteriales	Rhodothermaceae		S002034283 Salisaeta longa; LYG10 Lineage=Root;rootrank;Bacteria;domain;"Bacteroidetes";phylum;"Sphingobacteria";class;"Sphingobacteriales";order;"Rhodothermaceae";family;Salisaeta;genus	89.23	2.00E-136	486
2559	1	0	1	3	1	2	5	23	12	Bacteroidetes	Sphingobacteria	Sphingobacteriales	Rhodothermaceae		S002234457 Salinibacter luteus; DGO Lineage=Root;rootrank;Bacteria;domain;"Bacteroidetes";phylum;"Sphingobacteria";class;"Sphingobacteriales";order;"Rhodothermaceae";family;Salinibacter;genus	90.51	3.00E-144	512
714	0	0	0	0	0	0	1	0	0	Bacteroidetes	Sphingobacteria	Sphingobacteriales	Rhodothermaceae		S002234457 Salinibacter luteus; DGO Lineage=Root;rootrank;Bacteria;domain;"Bacteroidetes";phylum;"Sphingobacteria";class;"Sphingobacteriales";order;"Rhodothermaceae";family;Salinibacter;genus	86.96	2.00E-121	436
3073	0	0	0	0	0	0	0	1	0	Bacteroidetes	Sphingobacteria	Sphingobacteriales	Rhodothermaceae		S002235816 Rubricoccus marinus; SG-29 Lineage=Root;rootrank;Bacteria;domain;"Bacteroidetes";phylum;"Sphingobacteria";class;"Sphingobacteriales";order;"Rhodothermaceae";family;Salisaeta;genus	92.29	2.00E-156	553
1253	0	0	0	7	2	3	1	10	6	Bacteroidetes	Sphingobacteria	Sphingobacteriales	Rhodothermaceae		S002288772 Rhodothermus marinus DSM 4252 Lineage=Root;rootrank;Bacteria;domain;"Bacteroidetes";phylum;"Sphingobacteria";class;"Sphingobacteriales";order;"Rhodothermaceae";family;Rhodothermus;genus	90.96	4.00E-138	492
1916	0	0	0	0	0	7	5	2	1	Bacteroidetes	Sphingobacteria	Sphingobacteriales	Rhodothermaceae		S002288772 Rhodothermus marinus DSM 4252 Lineage=Root;rootrank;Bacteria;domain;"Bacteroidetes";phylum;"Sphingobacteria";class;"Sphingobacteriales";order;"Rhodothermaceae";family;Rhodothermus;genus	89.59	3.00E-129	462
769	0	0	0	0	0	0	0	0	1	Bacteroidetes	Sphingobacteria	Sphingobacteriales	Rhodothermaceae		S002288772 Rhodothermus marinus DSM 4252 Lineage=Root;rootrank;Bacteria;domain;"Bacteroidetes";phylum;"Sphingobacteria";class;"Sphingobacteriales";order;"Rhodothermaceae";family;Rhodothermus;genus	87.4	3.00E-124	446
2973	1	0	73	21	28	11	23	23	17	Bacteroidetes	Sphingobacteria	Sphingobacteriales	Rhodothermaceae		S002906604 Rhodothermus marinus SG0.5JP17-172 Lineage=Root;rootrank;Bacteria;domain;"Bacteroidetes";phylum;"Sphingobacteria";class;"Sphingobacteriales";order;"Rhodothermaceae";family;Rhodothermus;genus	89.65	8.00E-130	464

1129	24	5	18	3	8	6	13	26	25	Bacteroidetes	Sphingobacteria	Sphingobacteriales	Rhodothermaceae		S002906604 Rhodothermus marinus SG0.5JP17-172 Lineage=Root;rootrank;Bacteria;domain;"Bacteroidetes";phylum;"Sphingobacteria";class;"Sphingobacteriales";order;"Rhodothermaceae";family;Rhodothermus;genus	91.58	2.00E-141	503
2311	0	1	3	5	5	2	6	2	5	Bacteroidetes	Sphingobacteria	Sphingobacteriales	Rhodothermaceae		S002906604 Rhodothermus marinus SG0.5JP17-172 Lineage=Root;rootrank;Bacteria;domain;"Bacteroidetes";phylum;"Sphingobacteria";class;"Sphingobacteriales";order;"Rhodothermaceae";family;Rhodothermus;genus	90.71	6.00E-136	484
2962	0	0	0	10	1	1	0	7	5	Bacteroidetes	Sphingobacteria	Sphingobacteriales	Rhodothermaceae		S002906604 Rhodothermus marinus SG0.5JP17-172 Lineage=Root;rootrank;Bacteria;domain;"Bacteroidetes";phylum;"Sphingobacteria";class;"Sphingobacteriales";order;"Rhodothermaceae";family;Rhodothermus;genus	87.4	7.00E-116	418
1161	0	0	0	3	2	0	0	5	4	Bacteroidetes	Sphingobacteria	Sphingobacteriales	Rhodothermaceae		S002906604 Rhodothermus marinus SG0.5JP17-172 Lineage=Root;rootrank;Bacteria;domain;"Bacteroidetes";phylum;"Sphingobacteria";class;"Sphingobacteriales";order;"Rhodothermaceae";family;Rhodothermus;genus	89.07	2.00E-126	453
2276	0	0	0	1	0	0	0	0	0	Bacteroidetes	Sphingobacteria	Sphingobacteriales	Rhodothermaceae		S002906604 Rhodothermus marinus SG0.5JP17-172 Lineage=Root;rootrank;Bacteria;domain;"Bacteroidetes";phylum;"Sphingobacteria";class;"Sphingobacteriales";order;"Rhodothermaceae";family;Rhodothermus;genus	88.69	1.00E-132	473
2265	0	0	0	0	0	0	0	0	1	Bacteroidetes	Sphingobacteria	Sphingobacteriales	Rhodothermaceae		S002906604 Rhodothermus marinus SG0.5JP17-172 Lineage=Root;rootrank;Bacteria;domain;"Bacteroidetes";phylum;"Sphingobacteria";class;"Sphingobacteriales";order;"Rhodothermaceae";family;Rhodothermus;genus	89.32	1.00E-127	457
2796	52	420	81	0	12	48	54	29	3	Bacteroidetes	Sphingobacteria	Sphingobacteriales	Rhodothermaceae		S001351089 Rhodothermus profundi (T); PRI2902 Lineage=Root;rootrank;Bacteria;domain;"Bacteroidetes";phylum;"Sphingobacteria";class;"Sphingobacteriales";order;"Rhodothermaceae";family;Rhodothermus;genus	88.14	5.00E-122	438
2908	23	300	160	5	48	51	111	18	6	Bacteroidetes	Sphingobacteria	Sphingobacteriales	Rhodothermaceae		S001351089 Rhodothermus profundi (T); PRI2902 Lineage=Root;rootrank;Bacteria;domain;"Bacteroidetes";phylum;"Sphingobacteria";class;"Sphingobacteriales";order;"Rhodothermaceae";family;Rhodothermus;genus	87.63	3.00E-119	429
1882	57	401	107	0	7	41	80	1	2	Bacteroidetes	Sphingobacteria	Sphingobacteriales	Rhodothermaceae		S002234457 Salinibacter luteus; DGO Lineage=Root;rootrank;Bacteria;domain;"Bacteroidetes";phylum;"Sphingobacteria";class;"Sphingobacteriales";order;"Rhodothermaceae";family;Salinibacter;genus	92.2	1.00E-147	523
1942	3	22	3	0	0	12	23	3	0	Bacteroidetes	Sphingobacteria	Sphingobacteriales	Rhodothermaceae		S002234457 Salinibacter luteus; DGO Lineage=Root;rootrank;Bacteria;domain;"Bacteroidetes";phylum;"Sphingobacteria";class;"Sphingobacteriales";order;"Rhodothermaceae";family;Salinibacter;genus	91.4	1.00E-142	507
2345	0	0	10	0	1	1	10	5	1	Bacteroidetes	Sphingobacteria	Sphingobacteriales	Rhodothermaceae		S002288772 Rhodothermus marinus DSM 4252 Lineage=Root;rootrank;Bacteria;domain;"Bacteroidetes";phylum;"Sphingobacteria";class;"Sphingobacteriales";order;"Rhodothermaceae";family;Rhodothermus;genus	87.67	9.00E-120	431
3259	0	1	0	0	0	0	0	0	1	Bacteroidetes	Sphingobacteria	Sphingobacteriales	Rhodothermaceae		S002288772 Rhodothermus marinus DSM 4252 Lineage=Root;rootrank;Bacteria;domain;"Bacteroidetes";phylum;"Sphingobacteria";class;"Sphingobacteriales";order;"Rhodothermaceae";family;Rhodothermus;genus	89.69	1.00E-138	494
2626	187	339	78	1	9	64	100	1	0	Bacteroidetes	Sphingobacteria	Sphingobacteriales	Rhodothermaceae		S002290734 Salinibacter ruber (T); DSM 13855; M31 Lineage=Root;rootrank;Bacteria;domain;"Bacteroidetes";phylum;"Sphingobacteria";class;"Sphingobacteriales";order;"Rhodothermaceae";family;Salinibacter;genus	89.25	3.00E-129	462
983	10	18	15	0	1	8	13	9	4	Bacteroidetes	Sphingobacteria	Sphingobacteriales	Rhodothermaceae		S002290734 Salinibacter ruber (T); DSM 13855; M31 Lineage=Root;rootrank;Bacteria;domain;"Bacteroidetes";phylum;"Sphingobacteria";class;"Sphingobacteriales";order;"Rhodothermaceae";family;Salinibacter;genus	87.53	5.00E-117	422
190	2	61	7	0	0	2	1	0	0	Bacteroidetes	Sphingobacteria	Sphingobacteriales	Rhodothermaceae		S002290734 Salinibacter ruber (T); DSM 13855; M31 Lineage=Root;rootrank;Bacteria;domain;"Bacteroidetes";phylum;"Sphingobacteria";class;"Sphingobacteriales";order;"Rhodothermaceae";family;Salinibacter;genus	88.25	7.00E-121	435
504	0	0	0	0	0	10	13	0	0	Bacteroidetes	Sphingobacteria	Sphingobacteriales	Rhodothermaceae		S002290734 Salinibacter ruber (T); DSM 13855; M31 Lineage=Root;rootrank;Bacteria;domain;"Bacteroidetes";phylum;"Sphingobacteria";class;"Sphingobacteriales";order;"Rhodothermaceae";family;Salinibacter;genus	87.23	7.00E-116	418
2049	0	1	0	0	0	0	0	0	0	Bacteroidetes	Sphingobacteria	Sphingobacteriales	Rhodothermaceae		S002906604 Rhodothermus marinus SG0.5JP17-172 Lineage=Root;rootrank;Bacteria;domain;"Bacteroidetes";phylum;"Sphingobacteria";class;"Sphingobacteriales";order;"Rhodothermaceae";family;Rhodothermus;genus	88.86	8.00E-125	448
1077	0	0	0	0	0	0	0	0	1	Bacteroidetes	Sphingobacteria	Sphingobacteriales	Rhodothermaceae		S002906604 Rhodothermus marinus SG0.5JP17-172 Lineage=Root;rootrank;Bacteria;domain;"Bacteroidetes";phylum;"Sphingobacteria";class;"Sphingobacteriales";order;"Rhodothermaceae";family;Rhodothermus;genus	87.06	2.00E-115	416
1710	0	0	0	0	0	0	0	0	1	Bacteroidetes	Sphingobacteria	Sphingobacteriales	Rhodothermaceae		S002906604 Rhodothermus marinus SG0.5JP17-172 Lineage=Root;rootrank;Bacteria;domain;"Bacteroidetes";phylum;"Sphingobacteria";class;"Sphingobacteriales";order;"Rhodothermaceae";family;Rhodothermus;genus	86.15	2.00E-116	420
3208	0	0	0	3	0	0	0	0	0	Bacteroidetes	Sphingobacteria	Sphingobacteriales	Saprospiraceae		S000721195 Olivibacter soli (T); Gsoil 034 Lineage=Root;rootrank;Bacteria;domain;"Bacteroidetes";phylum;"Sphingobacteria";class;"Sphingobacteriales";order;Sphingobacteriaceae;family;Olivibacter;genus	84.64	9.00E-100	364
697	0	0	0	3	1	0	0	0	0	Bacteroidetes	Sphingobacteria	Sphingobacteriales	Saprospiraceae		S002151032 Pedobacter xinjiangensis; 12157 Lineage=Root;rootrank;Bacteria;domain;"Bacteroidetes";phylum;"Sphingobacteria";class;"Sphingobacteriales";order;Sphingobacteriaceae;family;Pedobacter;genus	84.14	6.00E-102	372
157	0	0	0	1	0	0	1	0	0	Bacteroidetes	Sphingobacteria	Sphingobacteriales	Saprospiraceae		S001153195 Pedobacter glucosidilyticus (T); 1-2 Lineage=Root;rootrank;Bacteria;domain;"Bacteroidetes";phylum;"Sphingobacteria";class;"Sphingobacteriales";order;Sphingobacteriaceae;family;Pedobacter;genus	83.84	1.00E-93	344
263	1	0	0	0	0	0	0	0	0	Bacteroidetes	Sphingobacteria	Sphingobacteriales	Saprospiraceae		S002167842 Pedobacter arcticus A12 Lineage=Root;rootrank;Bacteria;domain;"Bacteroidetes";phylum;"Sphingobacteria";class;"Sphingobacteriales";order;Sphingobacteriaceae;family;Pedobacter;genus	83.59	4.00E-98	359
478	0	0	1	6	3	0	0	0	0	Bacteroidetes	Sphingobacteria	Sphingobacteriales	Sphingobacteriaceae		S000721200 Pedobacter composti (T); TR6-06 Lineage=Root;rootrank;Bacteria;domain;"Bacteroidetes";phylum;"Sphingobacteria";class;"Sphingobacteriales";order;Sphingobacteriaceae;family;Pedobacter;genus	98.1	0	641
2891	0	0	0	3	0	0	0	0	0	Bacteroidetes	Sphingobacteria	Sphingobacteriales	Sphingobacteriaceae		S001575468 Pedobacter bauzanensis (T); BZ42 Lineage=Root;rootrank;Bacteria;domain;"Bacteroidetes";phylum;"Sphingobacteria";class;"Sphingobacteriales";order;Sphingobacteriaceae;family;Pedobacter;genus	95.61	5.00E-177	621
2657	0	0	0	0	0	0	0	0	1	Bacteroidetes	Sphingobacteria	Sphingobacteriales	Sphingobacteriaceae		S001575468 Pedobacter bauzanensis (T); BZ42 Lineage=Root;rootrank;Bacteria;domain;"Bacteroidetes";phylum;"Sphingobacteria";class;"Sphingobacteriales";order;Sphingobacteriaceae;family;Pedobacter;genus	98.1	0	641
1495	0	0	0	1	1	0	0	0	0	Bacteroidetes	Sphingobacteria	Sphingobacteriales	Sphingobacteriaceae		S001588688 Mucilaginibacter frigoritolerans (T); FT2.2 Lineage=Root;rootrank;Bacteria;domain;"Bacteroidetes";phylum;"Sphingobacteria";class;"Sphingobacteriales";order;Sphingobacteriaceae;family;Mucilaginibacter;genus	95.38	2.00E-166	586
2167	0	0	0	0	0	0	0	1	0	Bacteroidetes	Sphingobacteria	Sphingobacteriales	Sphingobacteriaceae	Parapedobacter	S001330458 Parapedobacter luteus (T); 4M29 Lineage=Root;rootrank;Bacteria;domain;"Bacteroidetes";phylum;"Sphingobacteria";class;"Sphingobacteriales";order;Sphingobacteriaceae;family;Parapedobacter;genus	96.12	2.00E-180	632
203	0	0	0	0	1	0	0	0	0	Bacteroidetes	Sphingobacteria	Sphingobacteriales	Sphingobacteriaceae	Pedobacter	S001152601 Pedobacter nyackensis (T); NWG-II14 Lineage=Root;rootrank;Bacteria;domain;"Bacteroidetes";phylum;"Sphingobacteria";class;"Sphingobacteriales";order;Sphingobacteriaceae;family;Pedobacter;genus	100	0	680
2102	0	0	0	0	0	0	0	1	0	Bacteroidetes	Sphingobacteria	Sphingobacteriales	Sphingobacteriaceae	Pedobacter	S003658537 Nubsella zeaxanthinifaciens; AS80 Lineage=Root;rootrank;Bacteria;domain;"Bacteroidetes";phylum;"Sphingobacteria";class;"Sphingobacteriales";order;Sphingobacteriaceae;family;Nubsella;genus	98.71	0	688
1795	0	0	0	0	0	0	0	0	2	Bacteroidetes	Sphingobacteria	Sphingobacteriales	Sphingobacteriaceae	Sphingobacterium	S000322819 Sphingobacteriaceae str. Ellin160 Lineage=Root;rootrank;Bacteria;domain;"Bacteroidetes";phylum;"Sphingobacteria";class;"Sphingobacteriales";order;Sphingobacteriaceae;family;Mucilaginibacter;genus	92.14	2.00E-146	520
2454	0	0	0	0	0	1	0	0	0	Bacteroidetes	Sphingobacteria	Sphingobacteriales	Sphingobacteriaceae		S000721202 Pedobacter daechungensis (T); Dae 13 Lineage=Root;rootrank;Bacteria;domain;"Bacteroidetes";phylum;"Sphingobacteria";class;"Sphingobacteriales";order;Sphingobacteriaceae;family;Pedobacter;genus	90.36	1.00E-133	477
1102	0	0	0	1	0	0	0	0	0	Bacteroidetes	Sphingobacteria	Sphingobacteriales	Sphingobacteriaceae		S000721974 Parapedobacter koreensis (T); Jip14 Lineage=Root;rootrank;Bacteria;domain;"Bacteroidetes";phylum;"Sphingobacteria";class;"Sphingobacteriales";order;Sphingobacteriaceae;family;Parapedobacter;genus	93.8	2.00E-165	582
875	0	0	0	0	1	0	0	0	0	Bacteroidetes	Sphingobacteria	Sphingobacteriales	Sphingobacteriaceae		S001575468 Pedobacter bauzanensis (T); BZ42 Lineage=Root;rootrank;Bacteria;domain;"Bacteroidetes";phylum;"Sphingobacteria";class;"Sphingobacteriales";order;Sphingobacteriaceae;family;Pedobacter;genus	94.57	2.00E-170	599
2330	0	0	1	0	0	1	0	0	0	Bacteroidetes	Sphingobacteria	Sphingobacteriales			S000384732 Flexibacter aggregans; IFO 15974 Lineage=Root;rootrank;Bacteria;domain;"Bacteroidetes";phylum;"Sphingobacteria";class;"Sphingobacteriales";order;"Flammeovirgaceae";family;Fulvivirga;genus	92.53	1.00E-157	556
1299	0	0	0	0	1	0	0	0	0	Bacteroidetes	Sphingobacteria	Sphingobacteriales			S000384732 Flexibacter aggregans; IFO 15974 Lineage=Root;rootrank;Bacteria;domain;"Bacteroidetes";phylum;"Sphingobacteria";class;"Sphingobacteriales";order;"Flammeovirgaceae";family;Fulvivirga;genus	92.01	1.00E-143	510
1062	2	40	2	0	1	12	6	0	0	Bacteroidetes	Sphingobacteria	Sphingobacteriales			S003710953 Gracilimonas tropica (T); CL-CB462 Lineage=Root;rootrank;Bacteria;domain;"Bacteroidetes";phylum;"Sphingobacteria";class;"Sphingobacteriales";order;Chitinophagaceae;family;Gracilimonas;genus	85.17	3.00E-109	396
759	6	16	2	1	2	1	1	3	1	Bacteroidetes	Sphingobacteria	Sphingobacteriales			S003710953 Gracilimonas tropica (T); CL-CB462 Lineage=Root;rootrank;Bacteria;domain;"Bacteroidetes";phylum;"Sphingobacteria";class;"Sphingobacteriales";order;Chitinophagaceae;family;Gracilimonas;genus	85.56	5.00E-102	372
1233	0	0	0	0	0	4	10	0	1	Bacteroidetes	Sphingobacteria	Sphingobacteriales			S003710953 Gracilimonas tropica (T); CL-CB462 Lineage=Root;rootrank;Bacteria;domain;"Bacteroidetes";phylum;"Sphingobacteria";class;"Sphingobacteriales";order;Chitinophagaceae;family;Gracilimonas;genus	90.54	2.00E-136	486
3199	0	0	0	0	0	0	0	15	11	Bacteroidetes	Sphingobacteria	Sphingobacteriales			S003721932 Pontibacter salisaro; AL-3 Lineage=Root;rootrank;Bacteria;domain;"Bacteroidetes";phylum;"Sphingobacteria";class;"Sphingobacteriales";order;Cytophagaceae;family;Pontibacter;genus	86.49	2.00E-111	403
1008	0	0	0	1	4	0	0	0	0	Bacteroidetes					S000539145 Tuber borchii symbiont b-1BO Lineage=Root;rootrank;Bacteria;domain;"Bacteroidetes";phylum;unclassified_"Bacteroidetes";	84.74	9.00E-100	364
3057	0	0	1	0	0	0	1	0	0	Bacteroidetes					S000721974 Parapedobacter koreensis (T); Jip14 Lineage=Root;rootrank;Bacteria;domain;"Bacteroidetes";phylum;"Sphingobacteria";class;"Sphingobacteriales";order;Sphingobacteriaceae;family;Parapedobacter;genus	90	4.00E-133	475
3256	0	0	0	0	0	1	0	0	0	Bacteroidetes					S000769385 Coccinistipes vermicola; IMCC1411 Lineage=Root;rootrank;Bacteria;domain;"Bacteroidetes";phylum;Flavobacteria;class;"Flavobacteriales";order;Cryomorphaceae;family;Owenweeksia;genus	91.75	1.00E-152	540
3074	0	0	0	2	0	1	1	0	0	Bacteroidetes					S000825310 Pedobacter lentus (T); DS-40 Lineage=Root;rootrank;Bacteria;domain;"Bacteroidetes";phylum;"Sphingobacteria";class;"Sphingobacteriales";order;Sphingobacteriaceae;family;Pedobacter;genus	90.38	3.00E-134	479
2686	5	43	4	0	1	0	2	0	0	Bacteroidetes					S000966625 Lishizhenia tianjinensis (T); H6 Lineage=Root;rootrank;Bacteria;domain;"Bacteroidetes";phylum;Flavobacteria;class;"Flavobacteriales";order;Cryomorphaceae;family;Lishizhenia;genus	80.65	4.00E-73	276
2009	0	9	0	0	0	1	0	0	0	Bacteroidetes					S001239104 Fulvivirga kasyanovii; TISTR1781 Lineage=Root;rootrank;Bacteria;domain;"Bacteroidetes";phylum;"Sphingobacteria";class;"Sphingobacteriales";order;"Flammeovirgaceae";family;Fulvivirga;genus	85.97	9.00E-115	414
2780	2	0	0	0	0	0	0	0	0	Bacteroidetes					S002156957 Legionella taurinensis; ucsc11 Lineage=Root;rootrank;Bacteria;domain;"Proteobacteria";phylum;Gammaproteobacteria;class;Legionellales;order;Legionellaceae;family;Legionella;genus	79.24	8.00E-71	268
2718	0	0	2	0	1	0	0	0	0	Bacteroidetes					S002304326 Marivirga tractuosa DSM 4126 Lineage=Root;rootrank;Bacteria;domain;"Bacteroidetes";phylum;"Sphingobacteria";class;"Sphingobacteriales";order;"Flammeovirgaceae";family;Marivirga;genus	86.7	1.00E-118	427
850	0	0	0	0	0	0	0	0	1	Bacteroidetes					S002304326 Marivirga tractuosa DSM 4126 Lineage=Root;rootrank;Bacteria;domain;"Bacteroidetes";phylum;"Sphingobacteria";class;"Sphingobacteriales";order;"Flammeovirgaceae";family;Marivirga;genus	89.74	1.00E-138	494
1914	5	0	0	0	0	0	0	0	0	Bacteroidetes					S002968465 Owenweeksia hongkongensis DSM 17368 Lineage=Root;rootrank;Bacteria;domain;"Bacteroidetes";phylum;Flavobacteria;class;"Flavobacteriales";order;Cryomorphaceae;family;Owenweeksia;genus	89.4	8.00E-130	464
1512	0	1	4	0	0	0	2	0	0	Bacteroidetes					S002968465 Owenweeksia hongkongensis DSM 17368 Lineage=Root;rootrank;Bacteria;domain;"Bacteroidetes";phylum;Flavobacteria;class;"Flavobacteriales";order;Cryomorphaceae;family;Owenweeksia;genus	87.89	2.00E-127	457
1244	0	0	0	0	0	0	0	5	21	Bacteroidetes					S003262915 Marinoscillum furvescens; NBRC 15994 Lineage=Root;rootrank;Bacteria;domain;"Bacteroidetes";phylum;"Sphingobacteria";class;"Sphingobacteriales";order;"Flammeovirgaceae";family;Marinoscillum;genus	90.08	5.00E-132	472
868	0	0	0	0	0	0	0	0	1	Bacteroidetes					S003262915 Marinoscillum furvescens; NBRC 15994 Lineage=Root;rootrank;Bacteria;domain;"Bacteroidetes";phylum;"Sphingobacteria";class;"Sphingobacteriales";order;"Flammeovirgaceae";family;Marinoscillum;genus	96.47	1.00E-172	606
1654	0	0	0	0	0	0	1	0	0	Bacteroidetes					S003262943 Chitinophaga arvensicola; NBRC 16138 Lineage=Root;rootrank;Bacteria;domain;"Bacteroidetes";phylum;"Sphingobacteria";class;"Sphingobacteriales";order;Chitinophagaceae;family;Chitinophaga;genus	86.82	3.00E-120	433
611	0	0	0	1	1	0	0	0	0	Bacteroidetes					S003264049 Adhaeribacter terreus; NBRC 104235 Lineage=Root;rootrank;Bacteria;domain;"Bacteroidetes";phylum;"Sphingobacteria";class;"Sphingobacteriales";order;Cytophagaceae;family;Adhaeribacter;genus	86.6	1.00E-118	427
3048	0	26	4	0	0	5	6	3	0	Bacteroidetes					S003264256 Adhaeribacter aerolatus; NBRC 106133 Lineage=Root;rootrank;Bacteria;domain;"Bacteroidetes";phylum;"Sphingobacteria";class;"Sphingobacteriales";order;Cytophagaceae;family;Adhaeribacter;genus	86.41	2.00E-111	403
328	1	1	0	0	0	1	0	0	0	Bacteroidetes					S003277397 Solitalea canadensis DSM 3403 Lineage=Root;rootrank;Bacteria;domain;"Bacteroidetes";phylum;"Sphingobacteria";class;"Sphingobacteriales";order;Sphingobacteriaceae;family;Solitalea;genus	87.53	1.00E-117	424
2082	0	0	1	0	0	0	0	0	0	Bacteroidetes					S003277397 Solitalea canadensis DSM 3403 Lineage=Root;rootrank;Bacteria;domain;"Bacteroidetes";phylum;"Sphingobacteria";class;"Sphingobacteriales";order;Sphingobacteriaceae;family;Solitalea;genus	86.76	1.00E-112	407
2896	0	0	0	1	0	0	0	0	0	Bacteroidetes					S003277397 Solitalea canadensis DSM 3403 Lineage=Root;rootrank;Bacteria;domain;"Bacteroidetes";phylum;"Sphingobacteria";class;"Sphingobacteriales";order;Sphingobacteriaceae;family;Solitalea;genus	87.67	4.00E-118	425
3183	0	0	0	1	0	0	0	0	0	Bacteroidetes					S003277397 Solitalea canadensis DSM 3403 Lineage=Root;rootrank;Bacteria;domain;"Bacteroidetes";phylum;"Sphingobacteria";class;"Sphingobacteriales";order;Sphingobacteriaceae;family;Solitalea;genus	92.12	2.00E-146	520
3071	10	32	2	0	1	12	10	5	0	Bacteroidetes					S003619184 Nafulsella turpanensis ZLM-10 Lineage=Root;rootrank;Bacteria;domain;"Bacteroidetes";phylum;"Sphingobacteria";class;"Sphingobacteriales";order;"Flammeovirgaceae";family;unclassified_"Flammeovirgaceae";	88.43	7.00E-131	468
2100	227	296	4	1	2	12	16	1	0	Bacteroidetes					S003710953 Gracilimonas tropica (T); CL-CB462 Lineage=Root;rootrank;Bacteria;domain;"Bacteroidetes";phylum;"Sphingobacteria";class;"Sphingobacteriales";order;Chitinophagaceae;family;Gracilimonas;genus	84.68	2.00E-96	353
1135	30	75	42	0	0	21	41	0	0	Bacteroidetes					S003710953 Gracilimonas tropica (T); CL-CB462 Lineage=Root;rootrank;Bacteria;domain;"Bacteroidetes";phylum;"Sphingobacteria";class;"Sphingobacteriales";order;Chitinophagaceae;family;Gracilimonas;genus	84.44	9.00E-95	348
3011	15	1	12	0	0	8	22	0	0	Bacteroidetes					S003710953 Gracilimonas tropica (T); CL-CB462 Lineage=Root;rootrank;Bacteria;domain;"Bacteroidetes";phylum;"Sphingobacteria";class;"Sphingobacteriales";order;Chitinophagaceae;family;Gracilimonas;genus	85.43	2.00E-101	370
3080	0	2	5	0	0	2	3	18	13	Bacteroidetes					S003710953 Gracilimonas tropica (T); CL-CB462 Lineage=Root;rootrank;Bacteria;domain;"Bacteroidetes";phylum;"Sphingobacteria";class;"Sphingobacteriales";order;Chitinophagaceae;family;Gracilimonas;genus	85.64	5.00E-102	372
2808	0	12	0	0	0	0	2	0	0	Bacteroidetes					S003710953 Gracilimonas tropica (T); CL-CB462 Lineage=Root;rootrank;Bacteria;domain;"Bacteroidetes";phylum;"Sphingobacteria";class;"Sphingobacteriales";order;Chitinophagaceae;family;Gracilimonas;genus	83.8	7.00E-101	368
554	1	0	0	0	0	0	0	0	0	Bacteroidetes					S003710953 Gracilimonas tropica (T); CL-CB462 Lineage=Root;rootrank;Bacteria;domain;"Bacteroidetes";phylum;"Sphingobacteria";class;"Sphingobacteriales";order;Chitinophagaceae;family;Gracilimonas;genus	85.42	3.00E-110	399
733	0	1	0	0	0	0	0	0	0	Bacteroidetes					S003710953 Gracilimonas tropica (T); CL-CB462 Lineage=Root;rootrank;Bacteria;domain;"Bacteroidetes";phylum;"Sphingobacteria";class;"Sphingobacteriales";order;Chitinophagaceae;family;Gracilimonas;genus	86.73	1.00E-118	427
1979	0	0	0	0	0	0	0	0	1	BRC	PRR-				S000428939 Ruminococcus flavefaciens; AR72 Lineage=Root;rootrank;Bacteria;domain;Firmicutes;phylum;Clostridia;class;Clostridiales;order;Ruminococcaceae;family;Ruminococcus;genus	83.97	2.00E-101	370
2848	0	0	0	1	0	0	0	0	0	BRC	PRR-				S000437600 Pelobacter venetianus (T) Lineage=Root;rootrank;Bacteria;domain;"Proteobacteria";phylum;Deltaproteobacteria;class;Desulfuromonadales;order;Desulfuromonadaceae;family;Pelobacter;genus	84.99	2.00E-101	370
1704	0	0	0	0	1	0	0	0	0	BRC	PRR-				S001328337 Actinomyces timonensis (T); 7400942 Lineage=Root;rootrank;Bacteria;domain;"Actinobacteria";phylum;Actinobacteria;class;Actinobacteridae;subclass;Actinomycetales;order;Actinomycineae;suborder;Actinomycetaceae;family;Actinomyces;genus	83.83	3.00E-94	346
2628	0	0	0	1	0	0	0	0	0	BRC	PRR-				S002164967 Aciditerrimonas ferrireducens (T); IC-180 Lineage=Root;rootrank;Bacteria;domain;"Actinobacteria";phylum;Actinobacteria;class;Acidimicrobidae;subclass;Acidimicrobiales;order;"Acidimicrobineae";suborder;"Acidimicrobineae"_incertae_sedis;family;Aciditerrimonas;genus	83.65	4.00E-93	342
810	0	0	0	1	0	0	0	0	0	BRC	PRR-				S002290442 Veillonella parvula DSM 2008 Lineage=Root;rootrank;Bacteria;domain;Firmicutes;phylum;Negativicutes;class;Selenomonadales;order;Veillonellaceae;family;Veillonella;genus	84.41	1.00E-98	361
781	0	0	0	0	1	0	0	0	0	BRC	PRR-				S002440493 Armatimonas rosea (T); YO-36 Lineage=Root;rootrank;Bacteria;domain;"Armatimonadetes";phylum;Armatimonadia;class;Armatimonadales;order;Armatimonadaceae;family;Armatimonas/Armatimonadetes_gp1;genus	82.75	9.00E-90	331
2372	1	0	0	1	0	0	0	0	0	BRC	PRR-				S002949677 Selenomonas flueggei; JCM 8544 Lineage=Root;rootrank;Bacteria;domain;Firmicutes;phylum;Negativicutes;class;Selenomonadales;order;Veillonellaceae;family;Selenomonas;genus	81.23	3.00E-79	296
3067	0	0	0	1	0	0	0	0	0	BRC	PRR-				S000569921 Pelotomaculum isophthalicicum (T); JI Lineage=Root;rootrank;Bacteria;domain;Firmicutes;phylum;Clostridia;class;Clostridiales;order;Peptococcaceae 2;family;Pelotomaculum;genus	86.41	4.00E-118	425
3052	0	0	1	0	3	0	0	3	4	CCMb					S000626776 Geoalkalibacter ferrihydriticus (T); Z-0531 Lineage=Root;rootrank;Bacteria;domain;"Proteobacteria";phylum;Deltaproteobacteria;class;Desulfuromonadales;order;Geobacteraceae;family;Geoalkalibacter;genus	86.93	3.00E-120	433
2698	0	0	0	0	0	0	0	2	0	CCMb					S002350761 Desulfovibrio giganteus; type strain: DSM 4123 Lineage=Root;rootrank;Bacteria;domain;"Proteobacteria";phylum;Deltaproteobacteria;class;Desulfovibrionales;order;Desulfovibrionaceae;family;Desulfovibrio;genus	85.52	7.00E-106	385
2295	0	0	0	0	0	0	0	0	1	Chlamydiae	Chlamydiae	Chlamydiales	Chlamydiaceae		S003289868 Candidatus Amphibiichlamydia ranarum; AMCS11/3 Lineage=Root;rootrank;Bacteria;domain;"Chlamydiae";phylum;Chlamydiae;class;Chlamydiales;order;Chlamydiaceae;family;Chlamydophila;genus	90.68	7.00E-146	518
1787	0	0	1	1	0	0	0	0	0	Chlamydiae	Chlamydiae	Chlamydiales	Rhabdochlamydiaceae	CandidatusRhabdochlamydia	S003711545 Candidatus Rhabdochlamydia porcellionis; 15C Lineage=Root;rootrank;Bacteria;domain;"Chlamydiae";phylum;Chlamydiae;class;Chlamydiales;order;unclassified_Chlamydiales;	91.69	2.00E-145	516
3214	0	0	1	0	0	0	0	0	0	Chlamydiae	Chlamydiae	Chlamydiales			S002907130 Parachlamydia acanthamoebae UV-7 Lineage=Root;rootrank;Bacteria;domain;"Chlamydiae";phylum;Chlamydiae;class;Chlamydiales;order;Parachlamydiaceae;family;Parachlamydia;genus	90.31	9.00E-145	514
325	0	0	0	1	0	0	0	0	0	Chlorobi	BSV9				S001550376 Caldithrix palaeochoryensis (T); MC Lineage=Root;rootrank;Bacteria;domain;"Deferribacteres";phylum;Deferribacteres;class;Deferribacterales;order;Deferribacterales_incertae_sedis;family;Caldithrix;genus	85.71	2.00E-82	307
1017	0	0	0	0	1	0	0	0	0	Chlorobi	BSV9				S003259712 Syntrophothermus lipocalidus DSM 12680; TGB-C1 Lineage=Root;rootrank;Bacteria;domain;Firmicutes;phylum;Clostridia;class;Clostridiales;order;Syntrophomonadaceae;family;Syntrophothermus;genus	86.21	1.00E-83	311
3165	0	0	6	3	0	1	0	22	0	Chlorobi	Ignavibacteria	Ignavibacteriales	Ignavibacteriaceae		S000549637 Desulfonatronum cooperativum (T); Z-7999 Lineage=Root;rootrank;Bacteria;domain;"Proteobacteria";phylum;Deltaproteobacteria;class;Desulfovibrionales;order;Desulfonatronaceae;family;Desulfonatronum;genus	84.97	5.00E-102	372
858	0	0	0	25	11	4	2	10	33	Chlorobi	Ignavibacteria	Ignavibacteriales	Ignavibacteriaceae		S000775342 Chlorobium phaeobacteroides; Glu Lineage=Root;rootrank;Bacteria;domain;"Chlorobi";phylum;"Chlorobia";class;Chlorobiales;order;Chlorobiaceae;family;Chlorobium;genus	84.7	5.00E-97	355
1260	0	0	0	4	0	0	0	0	0	Chlorobi	Ignavibacteria	Ignavibacteriales	Ignavibacteriaceae		S002288943 Chlorobaculum parvum NCIB 8327 Lineage=Root;rootrank;Bacteria;domain;"Chlorobi";phylum;"Chlorobia";class;Chlorobiales;order;Chlorobiaceae;family;Chlorobium;genus	84.14	9.00E-95	348
20	0	0	0	2	0	0	0	0	0	Chlorobi	Ignavibacteria	Ignavibacteriales	Ignavibacteriaceae		S002290428 Desulfovibrio vulgaris str. 'Miyazaki F' Lineage=Root;rootrank;Bacteria;domain;"Proteobacteria";phylum;Deltaproteobacteria;class;Desulfovibrionales;order;Desulfovibrionaceae;family;Desulfovibrio;genus	83.98	7.00E-101	368
1743	0	0	0	1	0	0	0	0	0	Chlorobi	SJA-28				S002288943 Chlorobaculum parvum NCIB 8327 Lineage=Root;rootrank;Bacteria;domain;"Chlorobi";phylum;"Chlorobia";class;Chlorobiales;order;Chlorobiaceae;family;Chlorobium;genus	84.9	2.00E-106	387
489	0	0	0	2	0	0	0	1	0	Chlorobi	SMB09				S000650636 Rhodothermus clarus; AR3 Lineage=Root;rootrank;Bacteria;domain;"Bacteroidetes";phylum;"Sphingobacteria";class;"Sphingobacteriales";order;"Rhodothermaceae";family;Rhodothermus;genus	82.82	1.00E-93	344
2399	0	0	0	1	2	0	0	0	0	Chlorobi	SMB09				S002906604 Rhodothermus marinus SG0.5JP17-172 Lineage=Root;rootrank;Bacteria;domain;"Bacteroidetes";phylum;"Sphingobacteria";class;"Sphingobacteriales";order;"Rhodothermaceae";family;Rhodothermus;genus	84.24	5.00E-97	355
3232	0	0	0	5	3	0	0	0	1	Chlorobi	SMH02				S002914871 Ruminococcus flavefaciens; H25 Lineage=Root;rootrank;Bacteria;domain;Firmicutes;phylum;Clostridia;class;Clostridiales;order;Ruminococcaceae;family;Ruminococcus;genus	86.46	1.00E-83	311
2287	0	0	0	0	0	0	0	0	1	Chlorobi	SMH02				S002914871 Ruminococcus flavefaciens; H25 Lineage=Root;rootrank;Bacteria;domain;Firmicutes;phylum;Clostridia;class;Clostridiales;order;Ruminococcaceae;family;Ruminococcus;genus	86.46	1.00E-83	311
2007	0	0	0	1	0	0	0	0	0	Chlorobi					S000711252 Rhodobium gokarnense (T); type strain: JA173 Lineage=Root;rootrank;Bacteria;domain;"Proteobacteria";phylum;Alphaproteobacteria;class;Rhizobiales;order;Rhodobiaceae;family;Rhodobium;genus	83.25	3.00E-94	346
1131	0	0	0	0	0	0	0	2	0	Chlorobi					S000870804 Chlorobaculum parvum; 5Sg Lineage=Root;rootrank;Bacteria;domain;"Chlorobi";phylum;"Chlorobia";class;Chlorobiales;order;Chlorobiaceae;family;Chlorobium;genus	83.72	3.00E-99	363
3087	0	0	0	1	0	0	0	0	0	Chloroflexi					S002988186 Desulfosoma profundi; SPDX02-08 Lineage=Root;rootrank;Bacteria;domain;"Proteobacteria";phylum;Deltaproteobacteria;class;Syntrophobacterales;order;Syntrophobacteraceae;family;Desulfosoma;genus	86.79	4.00E-113	409
1130	0	0	1	2	1	0	1	0	0	Chloroflexi	Anaerolineae	A3	S47		S002225629 Caldilinea tarbellica; D1-25-10-4 Lineage=Root;rootrank;Bacteria;domain;"Chloroflexi";phylum;Caldilineae;class;Caldilineales;order;Caldilineaceae;family;Caldilinea;genus	82.97	1.00E-87	324
1057	0	0	0	0	2	0	0	0	0	Chloroflexi	Anaerolineae	A3	S47		S003291140 Litorilinea aerophila; PRI-4131 Lineage=Root;rootrank;Bacteria;domain;"Chloroflexi";phylum;Caldilineae;class;Caldilineales;order;Caldilineaceae;family;Caldilinea;genus	81.89	5.00E-82	305
3175	0	0	0	0	0	0	0	0	1	Chloroflexi	Anaerolineae	A4b			S000125079 Levilinea saccharolytica (T); KIBI-1 Lineage=Root;rootrank;Bacteria;domain;"Chloroflexi";phylum;Anaerolineae;class;Anaerolineales;order;Anaerolineaceae;family;Levilinea;genus	82.23	7.00E-86	318
2119	0	0	1	2	5	0	2	5	0	Chloroflexi	Anaerolineae	A4b			S000125963 Anaerolinea thermolimosa (T); IMO-1 Lineage=Root;rootrank;Bacteria;domain;"Chloroflexi";phylum;Anaerolineae;class;Anaerolineales;order;Anaerolineaceae;family;Anaerolinea;genus	78.78	3.00E-64	246
2271	0	0	0	0	0	0	0	0	1	Chloroflexi	Anaerolineae	A4b			S000125963 Anaerolinea thermolimosa (T); IMO-1 Lineage=Root;rootrank;Bacteria;domain;"Chloroflexi";phylum;Anaerolineae;class;Anaerolineales;order;Anaerolineaceae;family;Anaerolinea;genus	80.8	2.00E-77	291
2669	0	0	3	5	5	0	0	0	3	Chloroflexi	Anaerolineae	A4b			S000438737 Methylocaldum gracile (T); VKM-14L Lineage=Root;rootrank;Bacteria;domain;"Proteobacteria";phylum;Gammaproteobacteria;class;Methylococcales;order;Methylococcaceae;family;Methylocaldum;genus	81.4	7.00E-76	285
2005	0	0	0	0	0	0	0	1	4	Chloroflexi	Anaerolineae	A4b			S000438737 Methylocaldum gracile (T); VKM-14L Lineage=Root;rootrank;Bacteria;domain;"Proteobacteria";phylum;Gammaproteobacteria;class;Methylococcales;order;Methylococcaceae;family;Methylocaldum;genus	81.37	2.00E-77	291
630	0	0	0	0	0	0	1	0	1	Chloroflexi	Anaerolineae	A4b			S000622967 Longilinea arvoryzae (T); KOME-1 Lineage=Root;rootrank;Bacteria;domain;"Chloroflexi";phylum;Anaerolineae;class;Anaerolineales;order;Anaerolineaceae;family;Longilinea;genus	79.58	3.00E-69	263
2003	0	0	0	1	0	0	0	0	0	Chloroflexi	Anaerolineae	A4b			S000622967 Longilinea arvoryzae (T); KOME-1 Lineage=Root;rootrank;Bacteria;domain;"Chloroflexi";phylum;Anaerolineae;class;Anaerolineales;order;Anaerolineaceae;family;Longilinea;genus	81.15	3.00E-79	296
2767	0	0	0	1	0	0	0	0	0	Chloroflexi	Anaerolineae	A4b			S000622967 Longilinea arvoryzae (T); KOME-1 Lineage=Root;rootrank;Bacteria;domain;"Chloroflexi";phylum;Anaerolineae;class;Anaerolineales;order;Anaerolineaceae;family;Longilinea;genus	80.56	1.00E-79	298
268	0	1	9	17	10	26	24	4	16	Chloroflexi	Anaerolineae	A4b			S000629396 Paucisalibacillus globulus (T); type strain: B22 Lineage=Root;rootrank;Bacteria;domain;Firmicutes;phylum;Bacilli;class;Bacillales;order;Bacillaceae 2;family;Paucisalibacillus;genus	84.45	2.00E-71	270
2117	0	0	1	6	4	0	0	1	2	Chloroflexi	Anaerolineae	A4b			S000629396 Paucisalibacillus globulus (T); type strain: B22 Lineage=Root;rootrank;Bacteria;domain;Firmicutes;phylum;Bacilli;class;Bacillales;order;Bacillaceae 2;family;Paucisalibacillus;genus	82.45	2.00E-67	257
952	0	0	1	2	0	0	0	0	0	Chloroflexi	Anaerolineae	A4b			S000769575 Thiohalomonas denitrificans; HLD 14 Lineage=Root;rootrank;Bacteria;domain;"Proteobacteria";phylum;Gammaproteobacteria;class;Gammaproteobacteria_incertae_sedis;order;Thiohalomonas;genus	80.98	2.00E-76	287
3225	0	1	7	5	4	5	6	3	8	Chloroflexi	Anaerolineae	A4b			S002228285 Thermoanaerobacter uzonensis; CF1 Lineage=Root;rootrank;Bacteria;domain;Firmicutes;phylum;Clostridia;class;Thermoanaerobacterales;order;Thermoanaerobacteraceae;family;Thermoanaerobacter;genus	79.78	2.00E-66	254
1084	0	0	0	1	0	0	0	1	3	Chloroflexi	Anaerolineae	A4b			S002232103 Thermanaerothrix daxensis; GNS-1 Lineage=Root;rootrank;Bacteria;domain;"Chloroflexi";phylum;Anaerolineae;class;Anaerolineales;order;Anaerolineaceae;family;Bellilinea;genus	85.31	1.00E-78	294
1021	0	1	0	0	1	0	0	0	0	Chloroflexi	Anaerolineae	A4b			S002350028 Anaerolinea thermophila UNI-1 Lineage=Root;rootrank;Bacteria;domain;"Chloroflexi";phylum;Anaerolineae;class;Anaerolineales;order;Anaerolineaceae;family;Anaerolinea;genus	80.95	2.00E-77	291
2639	0	0	0	3	5	0	0	1	1	Chloroflexi	Anaerolineae	A4b			S003291140 Litorilinea aerophila; PRI-4131 Lineage=Root;rootrank;Bacteria;domain;"Chloroflexi";phylum;Caldilineae;class;Caldilineales;order;Caldilineaceae;family;Caldilinea;genus	80.05	2.00E-72	274
2717	0	0	0	1	0	0	0	0	0	Chloroflexi	Anaerolineae	A4b			S000618933 Cycloclasticus pugetii Lineage=Root;rootrank;Bacteria;domain;"Proteobacteria";phylum;Gammaproteobacteria;class;Thiotrichales;order;Piscirickettsiaceae;family;Cycloclasticus;genus	82.86	6.00E-92	339
2950	0	0	0	1	1	6	4	3	1	Chloroflexi	Anaerolineae	A4b			S002232103 Thermanaerothrix daxensis; GNS-1 Lineage=Root;rootrank;Bacteria;domain;"Chloroflexi";phylum;Anaerolineae;class;Anaerolineales;order;Anaerolineaceae;family;Bellilinea;genus	81.43	7.00E-81	302
2481	0	0	0	0	0	0	0	0	1	Chloroflexi	Anaerolineae	Caldilineales	Caldilineaceae		S002225629 Caldilinea tarbellica; D1-25-10-4 Lineage=Root;rootrank;Bacteria;domain;"Chloroflexi";phylum;Caldilineae;class;Caldilineales;order;Caldilineaceae;family;Caldilinea;genus	90.32	5.00E-137	488
2993	0	0	5	4	0	3	4	2	0	Chloroflexi	Anaerolineae	Caldilineales	Caldilineaceae		S003291140 Litorilinea aerophila; PRI-4131 Lineage=Root;rootrank;Bacteria;domain;"Chloroflexi";phylum;Caldilineae;class;Caldilineales;order;Caldilineaceae;family;Caldilinea;genus	93.28	8.00E-155	547
1252	0	0	0	0	0	0	1	1	0	Chloroflexi	Anaerolineae	Caldilineales	Caldilineaceae	Caldilinea	S000012674 Ammoniphilus oxalaticus; RAOx-FF Lineage=Root;rootrank;Bacteria;domain;Firmicutes;phylum;Bacilli;class;Bacillales;order;Paenibacillaceae 2;family;Ammoniphilus;genus	81.38	1.00E-78	294
973	0	0	0	5	4	2	0	0	1	Chloroflexi	Anaerolineae	Caldilineales	Caldilineaceae	Caldilinea	S000133470 Candidatus Thiodictyon syntrophicum str. Cad16; type strain: Cad16 Lineage=Root;rootrank;Bacteria;domain;"Proteobacteria";phylum;Gammaproteobacteria;class;Chromatiales;order;Chromatiaceae;family;Thiodictyon;genus	81.82	9.00E-80	298
1720	0	0	0	1	0	0	0	0	0	Chloroflexi	Anaerolineae	Caldilineales	Caldilineaceae	Caldilinea	S001611362 Thiococcus pfennigii; 4252 = DSM 228 Lineage=Root;rootrank;Bacteria;domain;"Proteobacteria";phylum;Gammaproteobacteria;class;Chromatiales;order;Chromatiaceae;family;Thioflavicoccus;genus	81.72	2.00E-81	303
1119	0	0	1	19	38	101	69	31	19	Chloroflexi	Anaerolineae	Caldilineales	Caldilineaceae	Caldilinea	S003291140 Litorilinea aerophila; PRI-4131 Lineage=Root;rootrank;Bacteria;domain;"Chloroflexi";phylum;Caldilineae;class;Caldilineales;order;Caldilineaceae;family;Caldilinea;genus	90.59	1.00E-138	494
1616	0	0	14	37	16	8	10	21	16	Chloroflexi	Anaerolineae	Caldilineales	Caldilineaceae	Caldilinea	S003291140 Litorilinea aerophila; PRI-4131 Lineage=Root;rootrank;Bacteria;domain;"Chloroflexi";phylum;Caldilineae;class;Caldilineales;order;Caldilineaceae;family;Caldilinea;genus	92.33	1.00E-157	556
164	0	0	0	31	24	14	30	11	6	Chloroflexi	Anaerolineae	Caldilineales	Caldilineaceae	Caldilinea	S003291140 Litorilinea aerophila; PRI-4131 Lineage=Root;rootrank;Bacteria;domain;"Chloroflexi";phylum;Caldilineae;class;Caldilineales;order;Caldilineaceae;family;Caldilinea;genus	91.13	2.00E-141	503
1925	0	0	2	19	6	6	3	2	12	Chloroflexi	Anaerolineae	Caldilineales	Caldilineaceae	Caldilinea	S003291140 Litorilinea aerophila; PRI-4131 Lineage=Root;rootrank;Bacteria;domain;"Chloroflexi";phylum;Caldilineae;class;Caldilineales;order;Caldilineaceae;family;Caldilinea;genus	90.82	5.00E-147	521
2415	0	0	0	2	10	8	11	7	5	Chloroflexi	Anaerolineae	Caldilineales	Caldilineaceae	Caldilinea	S003291140 Litorilinea aerophila; PRI-4131 Lineage=Root;rootrank;Bacteria;domain;"Chloroflexi";phylum;Caldilineae;class;Caldilineales;order;Caldilineaceae;family;Caldilinea;genus	90.31	3.00E-144	512
337	0	0	2	5	13	2	4	2	2	Chloroflexi	Anaerolineae	Caldilineales	Caldilineaceae	Caldilinea	S003291140 Litorilinea aerophila; PRI-4131 Lineage=Root;rootrank;Bacteria;domain;"Chloroflexi";phylum;Caldilineae;class;Caldilineales;order;Caldilineaceae;family;Caldilinea;genus	85.79	1.00E-108	394

1580	0	0	0	4	15	4	2	2	2	Chloroflexi	Anaerolineae	Caldilineales	Caldilineaceae	Caldilinea	S003291140 Litorilinea aerophila; PRI-4131 Lineage=Root;rootrank;Bacteria;domain;"Chloroflexi";phylum;Caldilineae;class;Caldilineales;order;Caldilineaceae;family;Caldilinea;genus	95.44	1.00E-168	593
3182	0	0	2	9	4	4	6	0	3	Chloroflexi	Anaerolineae	Caldilineales	Caldilineaceae	Caldilinea	S003291140 Litorilinea aerophila; PRI-4131 Lineage=Root;rootrank;Bacteria;domain;"Chloroflexi";phylum;Caldilineae;class;Caldilineales;order;Caldilineaceae;family;Caldilinea;genus	93.86	1.00E-167	590
67	0	0	1	7	7	1	1	7	0	Chloroflexi	Anaerolineae	Caldilineales	Caldilineaceae	Caldilinea	S003291140 Litorilinea aerophila; PRI-4131 Lineage=Root;rootrank;Bacteria;domain;"Chloroflexi";phylum;Caldilineae;class;Caldilineales;order;Caldilineaceae;family;Caldilinea;genus	91.71	2.00E-145	516
3240	0	0	1	1	4	0	0	2	1	Chloroflexi	Anaerolineae	Caldilineales	Caldilineaceae	Caldilinea	S003291140 Litorilinea aerophila; PRI-4131 Lineage=Root;rootrank;Bacteria;domain;"Chloroflexi";phylum;Caldilineae;class;Caldilineales;order;Caldilineaceae;family;Caldilinea;genus	92.78	5.00E-152	538
613	0	0	0	1	1	0	0	0	2	Chloroflexi	Anaerolineae	Caldilineales	Caldilineaceae	Caldilinea	S003291140 Litorilinea aerophila; PRI-4131 Lineage=Root;rootrank;Bacteria;domain;"Chloroflexi";phylum;Caldilineae;class;Caldilineales;order;Caldilineaceae;family;Caldilinea;genus	93.62	2.00E-165	582
1614	0	0	0	1	1	0	0	0	0	Chloroflexi	Anaerolineae	Caldilineales	Caldilineaceae	Caldilinea	S003291140 Litorilinea aerophila; PRI-4131 Lineage=Root;rootrank;Bacteria;domain;"Chloroflexi";phylum;Caldilineae;class;Caldilineales;order;Caldilineaceae;family;Caldilinea;genus	92.58	4.00E-158	558
1549	0	0	1	0	0	0	0	0	0	Chloroflexi	Anaerolineae	Caldilineales	Caldilineaceae	Caldilinea	S003291140 Litorilinea aerophila; PRI-4131 Lineage=Root;rootrank;Bacteria;domain;"Chloroflexi";phylum;Caldilineae;class;Caldilineales;order;Caldilineaceae;family;Caldilinea;genus	92.17	2.00E-156	553
334	0	0	0	1	0	0	0	0	0	Chloroflexi	Anaerolineae	Caldilineales	Caldilineaceae	Caldilinea	S003291140 Litorilinea aerophila; PRI-4131 Lineage=Root;rootrank;Bacteria;domain;"Chloroflexi";phylum;Caldilineae;class;Caldilineales;order;Caldilineaceae;family;Caldilinea;genus	89.57	5.00E-132	472
2396	0	0	0	0	0	0	1	0	0	Chloroflexi	Anaerolineae	Caldilineales	Caldilineaceae	Caldilinea	S003291140 Litorilinea aerophila; PRI-4131 Lineage=Root;rootrank;Bacteria;domain;"Chloroflexi";phylum;Caldilineae;class;Caldilineales;order;Caldilineaceae;family;Caldilinea;genus	94.13	1.00E-168	593
1917	0	0	0	3	0	0	0	0	0	Chloroflexi	Anaerolineae	Caldilineales	Caldilineaceae		S003291140 Litorilinea aerophila; PRI-4131 Lineage=Root;rootrank;Bacteria;domain;"Chloroflexi";phylum;Caldilineae;class;Caldilineales;order;Caldilineaceae;family;Caldilinea;genus	92.6	3.00E-159	562
2760	0	0	0	0	0	0	0	0	3	Chloroflexi	Anaerolineae	Caldilineales	Caldilineaceae		S003291140 Litorilinea aerophila; PRI-4131 Lineage=Root;rootrank;Bacteria;domain;"Chloroflexi";phylum;Caldilineae;class;Caldilineales;order;Caldilineaceae;family;Caldilinea;genus	93.28	2.00E-155	549
1995	0	0	0	6	1	0	0	0	0	Chloroflexi	Anaerolineae	Caldilineales			S000584753 Planifilum yunnanense (T); LA5 Lineage=Root;rootrank;Bacteria;domain;Firmicutes;phylum;Bacilli;class;Bacillales;order;Thermoactinomycetaceae 2;family;Planifilum;genus	84.49	3.00E-99	363
1385	0	0	1	2	6	0	0	1	0	Chloroflexi	Anaerolineae	Caldilineales			S003277205 Caldilinea aerophila DSM 14535 = NBRC 104270 Lineage=Root;rootrank;Bacteria;domain;"Chloroflexi";phylum;Caldilineae;class;Caldilineales;order;Caldilineaceae;family;Caldilinea;genus	83.6	1.00E-93	344
1444	0	0	0	1	0	0	0	0	0	Chloroflexi	Anaerolineae	CFB-26			S002225629 Caldilinea tarbellica; D1-25-10-4 Lineage=Root;rootrank;Bacteria;domain;"Chloroflexi";phylum;Caldilineae;class;Caldilineales;order;Caldilineaceae;family;Caldilinea;genus	82.74	4.00E-93	342
250	0	0	2	5	0	0	0	0	0	Chloroflexi	Anaerolineae	CFB-26			S002912019 Bacillus endophyticus; S139R Lineage=Root;rootrank;Bacteria;domain;Firmicutes;phylum;Bacilli;class;Bacillales;order;Bacillaceae 1;family;Bacillus;genus	81.65	9.00E-80	298
903	0	0	0	0	1	0	0	0	0	Chloroflexi	Anaerolineae	CFB-26			S003610541 Thermacetogenium phaeum DSM 12270 Lineage=Root;rootrank;Bacteria;domain;Firmicutes;phylum;Clostridia;class;Thermoanaerobacterales;order;Thermoanaerobacteraceae;family;Thermacetogenium;genus	81.3	1.00E-78	294
763	0	0	0	2	0	0	0	0	0	Chloroflexi	Anaerolineae	CFB-26			S002754539 Aeribacillus pallidus; C8 Lineage=Root;rootrank;Bacteria;domain;Firmicutes;phylum;Bacilli;class;Bacillales;order;Bacillaceae 1;family;Aeribacillus;genus	84.49	1.00E-98	361
3249	0	0	0	4	1	0	0	0	2	Chloroflexi	Anaerolineae	DRC3			S000434673 Thermovenabulum ferriorganovorum (T); Z-9801 Lineage=Root;rootrank;Bacteria;domain;Firmicutes;phylum;Clostridia;class;Clostridiales;order;Clostridiales_Incertae Sedis III;family;Thermovenabulum;genus	84.91	2.00E-101	370
2765	1	2	7	305	125	250	191	297	232	Chloroflexi	Anaerolineae	DRC3			S000622967 Longilinea arvoryzae (T); KOME-1 Lineage=Root;rootrank;Bacteria;domain;"Chloroflexi";phylum;Anaerolineae;class;Anaerolineales;order;Anaerolineaceae;family;Longilinea;genus	84.76	5.00E-102	372
1879	0	2	89	43	90	42	61	140	100	Chloroflexi	Anaerolineae	DRC3			S000622967 Longilinea arvoryzae (T); KOME-1 Lineage=Root;rootrank;Bacteria;domain;"Chloroflexi";phylum;Anaerolineae;class;Anaerolineales;order;Anaerolineaceae;family;Longilinea;genus	85.6	5.00E-107	388
1004	0	0	0	16	38	3	11	68	47	Chloroflexi	Anaerolineae	DRC3			S000622967 Longilinea arvoryzae (T); KOME-1 Lineage=Root;rootrank;Bacteria;domain;"Chloroflexi";phylum;Anaerolineae;class;Anaerolineales;order;Anaerolineaceae;family;Longilinea;genus	87.2	5.00E-117	422
1702	0	0	9	8	6	1	5	6	2	Chloroflexi	Anaerolineae	DRC3			S000622967 Longilinea arvoryzae (T); KOME-1 Lineage=Root;rootrank;Bacteria;domain;"Chloroflexi";phylum;Anaerolineae;class;Anaerolineales;order;Anaerolineaceae;family;Longilinea;genus	84.72	2.00E-101	370
1875	0	0	0	15	6	1	0	7	4	Chloroflexi	Anaerolineae	DRC3			S000622967 Longilinea arvoryzae (T); KOME-1 Lineage=Root;rootrank;Bacteria;domain;"Chloroflexi";phylum;Anaerolineae;class;Anaerolineales;order;Anaerolineaceae;family;Longilinea;genus	85.97	7.00E-116	418
2243	0	0	0	4	6	0	0	0	0	Chloroflexi	Anaerolineae	DRC3			S000622967 Longilinea arvoryzae (T); KOME-1 Lineage=Root;rootrank;Bacteria;domain;"Chloroflexi";phylum;Anaerolineae;class;Anaerolineales;order;Anaerolineaceae;family;Longilinea;genus	86.06	2.00E-110	399
245	0	0	1	5	1	0	0	2	0	Chloroflexi	Anaerolineae	DRC3			S000622967 Longilinea arvoryzae (T); KOME-1 Lineage=Root;rootrank;Bacteria;domain;"Chloroflexi";phylum;Anaerolineae;class;Anaerolineales;order;Anaerolineaceae;family;Longilinea;genus	85.79	1.00E-108	394
316	0	0	0	2	1	0	0	1	0	Chloroflexi	Anaerolineae	DRC3			S000622967 Longilinea arvoryzae (T); KOME-1 Lineage=Root;rootrank;Bacteria;domain;"Chloroflexi";phylum;Anaerolineae;class;Anaerolineales;order;Anaerolineaceae;family;Longilinea;genus	84.18	1.00E-98	361
1790	0	0	0	2	0	0	1	1	0	Chloroflexi	Anaerolineae	DRC3			S000622967 Longilinea arvoryzae (T); KOME-1 Lineage=Root;rootrank;Bacteria;domain;"Chloroflexi";phylum;Anaerolineae;class;Anaerolineales;order;Anaerolineaceae;family;Longilinea;genus	84.72	5.00E-102	372
2446	0	0	0	0	1	0	0	0	0	Chloroflexi	Anaerolineae	DRC3			S000622967 Longilinea arvoryzae (T); KOME-1 Lineage=Root;rootrank;Bacteria;domain;"Chloroflexi";phylum;Anaerolineae;class;Anaerolineales;order;Anaerolineaceae;family;Longilinea;genus	85.37	2.00E-105	383
2497	3	0	9	2	2	0	2	1	0	Chloroflexi	Anaerolineae	DRC3			S003290740 Nitrolancetus hollandicus Lb Lineage=Root;rootrank;Bacteria;domain;"Chloroflexi";phylum;Thermomicrobia;class;Sphaerobacteridae;subclass;Sphaerobacterales;order;"Sphaerobacterineae";suborder;Sphaerobacteraceae;family;Sphaerobacter;genus	83.65	2.00E-95	350
656	0	0	1	14	5	0	2	9	13	Chloroflexi	Anaerolineae	DRC3			S003291140 Litorilinea aerophila; PRI-4131 Lineage=Root;rootrank;Bacteria;domain;"Chloroflexi";phylum;Caldilineae;class;Caldilineales;order;Caldilineaceae;family;Caldilinea;genus	82.93	2.00E-90	333
3125	0	0	0	1	0	0	0	0	0	Chloroflexi	Anaerolineae	envOPS2			S000622966 Bellilinea caldifistulae (T); GOMI-1 Lineage=Root;rootrank;Bacteria;domain;"Chloroflexi";phylum;Anaerolineae;class;Anaerolineales;order;Anaerolineaceae;family;Bellilinea;genus	90.28	2.00E-131	470
2672	0	0	0	7	0	0	0	0	0	Chloroflexi	Anaerolineae	envOPS2			S000622967 Longilinea arvoryzae (T); KOME-1 Lineage=Root;rootrank;Bacteria;domain;"Chloroflexi";phylum;Anaerolineae;class;Anaerolineales;order;Anaerolineaceae;family;Longilinea;genus	88.77	5.00E-127	455
1288	0	0	0	1	0	0	0	0	0	Chloroflexi	Anaerolineae	H39			S000622967 Longilinea arvoryzae (T); KOME-1 Lineage=Root;rootrank;Bacteria;domain;"Chloroflexi";phylum;Anaerolineae;class;Anaerolineales;order;Anaerolineaceae;family;Longilinea;genus	83.15	3.00E-89	329
1417	0	0	0	1	20	0	0	0	5	Chloroflexi	Anaerolineae	H39			S002232103 Thermanaerothrix daxensis; GNS-1 Lineage=Root;rootrank;Bacteria;domain;"Chloroflexi";phylum;Anaerolineae;class;Anaerolineales;order;Anaerolineaceae;family;Bellilinea;genus	87.12	3.00E-114	412
1766	0	0	0	2	8	1	0	3	5	Chloroflexi	Anaerolineae	H39			S002232103 Thermanaerothrix daxensis; GNS-1 Lineage=Root;rootrank;Bacteria;domain;"Chloroflexi";phylum;Anaerolineae;class;Anaerolineales;order;Anaerolineaceae;family;Bellilinea;genus	87.4	7.00E-116	418
2755	0	0	0	10	5	1	0	1	0	Chloroflexi	Anaerolineae	H39			S002232103 Thermanaerothrix daxensis; GNS-1 Lineage=Root;rootrank;Bacteria;domain;"Chloroflexi";phylum;Anaerolineae;class;Anaerolineales;order;Anaerolineaceae;family;Bellilinea;genus	84.2	7.00E-96	351
724	0	0	0	4	1	1	0	0	4	Chloroflexi	Anaerolineae	H39			S002232103 Thermanaerothrix daxensis; GNS-1 Lineage=Root;rootrank;Bacteria;domain;"Chloroflexi";phylum;Anaerolineae;class;Anaerolineales;order;Anaerolineaceae;family;Bellilinea;genus	85.25	1.00E-102	374
121	0	0	0	2	1	0	1	0	0	Chloroflexi	Anaerolineae	S0208			S000125079 Levilinea saccharolytica (T); KIBI-1 Lineage=Root;rootrank;Bacteria;domain;"Chloroflexi";phylum;Anaerolineae;class;Anaerolineales;order;Anaerolineaceae;family;Levilinea;genus	83.73	1.00E-92	340
616	0	0	0	1	0	0	0	0	0	Chloroflexi	Anaerolineae	S0208			S002225629 Caldilinea tarbellica; D1-25-10-4 Lineage=Root;rootrank;Bacteria;domain;"Chloroflexi";phylum;Caldilineae;class;Caldilineales;order;Caldilineaceae;family;Caldilinea;genus	84.27	3.00E-99	363
3094	0	0	0	1	0	0	0	0	0	Chloroflexi	Anaerolineae	S0208			S002350110 Thermaerobacter marianensis DSM 12885 Lineage=Root;rootrank;Bacteria;domain;Firmicutes;phylum;Clostridia;class;Clostridiales;order;Clostridiales_Incertae Sedis XVII;family;Thermaerobacter;genus	84.99	7.00E-101	368
1036	0	0	0	2	0	0	0	0	0	Chloroflexi	Anaerolineae	S0208			S002988186 Desulfosoma profundi; SPDX02-08 Lineage=Root;rootrank;Bacteria;domain;"Proteobacteria";phylum;Deltaproteobacteria;class;Syntrophobacterales;order;Syntrophobacteraceae;family;Desulfosoma;genus	84.47	5.00E-97	355
1999	0	0	0	0	0	2	0	0	0	Chloroflexi	Anaerolineae	S0208			S003264045 Mechercharimyces mesophilus; NBRC 104191 Lineage=Root;rootrank;Bacteria;domain;Firmicutes;phylum;Bacilli;class;Bacillales;order;Thermoactinomycetaceae 2;family;Mechercharimyces;genus	84.41	1.00E-97	357
390	0	0	0	0	0	0	0	0	3	Chloroflexi	Anaerolineae	S0208			S003277205 Caldilinea aerophila DSM 14535 = NBRC 104270 Lineage=Root;rootrank;Bacteria;domain;"Chloroflexi";phylum;Caldilineae;class;Caldilineales;order;Caldilineaceae;family;Caldilinea;genus	83.6	9.00E-95	348
2739	0	0	5	14	6	1	4	2	3	Chloroflexi	Anaerolineae	S0208			S003291140 Litorilinea aerophila; PRI-4131 Lineage=Root;rootrank;Bacteria;domain;"Chloroflexi";phylum;Caldilineae;class;Caldilineales;order;Caldilineaceae;family;Caldilinea;genus	85.94	1.00E-108	394
218	0	0	0	2	1	2	0	5	0	Chloroflexi	Anaerolineae	S0208			S003291140 Litorilinea aerophila; PRI-4131 Lineage=Root;rootrank;Bacteria;domain;"Chloroflexi";phylum;Caldilineae;class;Caldilineales;order;Caldilineaceae;family;Caldilinea;genus	86.1	2.00E-110	399
2316	0	0	0	4	3	0	0	1	0	Chloroflexi	Anaerolineae	S0208			S003291140 Litorilinea aerophila; PRI-4131 Lineage=Root;rootrank;Bacteria;domain;"Chloroflexi";phylum;Caldilineae;class;Caldilineales;order;Caldilineaceae;family;Caldilinea;genus	85.98	1.00E-108	394
3120	0	0	0	2	0	0	0	0	0	Chloroflexi	Anaerolineae	S0208			S003291140 Litorilinea aerophila; PRI-4131 Lineage=Root;rootrank;Bacteria;domain;"Chloroflexi";phylum;Caldilineae;class;Caldilineales;order;Caldilineaceae;family;Caldilinea;genus	86.13	2.00E-110	399
336	0	0	1	0	0	0	0	0	0	Chloroflexi	Anaerolineae	SB-34			S002232103 Thermanaerothrix daxensis; GNS-1 Lineage=Root;rootrank;Bacteria;domain;"Chloroflexi";phylum;Anaerolineae;class;Anaerolineales;order;Anaerolineaceae;family;Bellilinea;genus	88.58	1.00E-133	477
1808	0	0	0	0	0	0	1	0	0	Chloroflexi	Anaerolineae	SJA-0			S000587182 Oscillochloris trichoides; C6 Lineage=Root;rootrank;Bacteria;domain;"Chloroflexi";phylum;"Chloroflexi";class;"Chloroflexales";order;"Chloroflexaceae";family;unclassified_"Chloroflexaceae";	77.66	3.00E-55	217
1126	0	0	1	1	3	0	0	0	0	Chloroflexi	Anaerolineae	SJA-0			S000622967 Longilinea arvoryzae (T); KOME-1 Lineage=Root;rootrank;Bacteria;domain;"Chloroflexi";phylum;Anaerolineae;class;Anaerolineales;order;Anaerolineaceae;family;Longilinea;genus	79.79	9.00E-70	265
1943	0	0	0	5	1	0	0	3	15	Chloroflexi	Anaerolineae				S000125078 Leptolinea tardivitalis (T); YMTK-2 Lineage=Root;rootrank;Bacteria;domain;"Chloroflexi";phylum;Anaerolineae;class;Anaerolineales;order;Anaerolineaceae;family;Leptolinea;genus	87.4	1.00E-118	427
1500	0	0	0	5	0	0	0	0	0	Chloroflexi	Anaerolineae				S000125078 Leptolinea tardivitalis (T); YMTK-2 Lineage=Root;rootrank;Bacteria;domain;"Chloroflexi";phylum;Anaerolineae;class;Anaerolineales;order;Anaerolineaceae;family;Leptolinea;genus	87.13	5.00E-117	422
2171	0	0	0	2	1	0	0	0	0	Chloroflexi	Anaerolineae				S000125078 Leptolinea tardivitalis (T); YMTK-2 Lineage=Root;rootrank;Bacteria;domain;"Chloroflexi";phylum;Anaerolineae;class;Anaerolineales;order;Anaerolineaceae;family;Leptolinea;genus	86.83	9.00E-115	414
147	0	0	0	0	0	0	0	0	1	Chloroflexi	Anaerolineae				S000125078 Leptolinea tardivitalis (T); YMTK-2 Lineage=Root;rootrank;Bacteria;domain;"Chloroflexi";phylum;Anaerolineae;class;Anaerolineales;order;Anaerolineaceae;family;Leptolinea;genus	87.94	5.00E-122	438
2347	0	0	0	2	1	0	0	4	5	Chloroflexi	Anaerolineae				S000125079 Levilinea saccharolytica (T); KIBI-1 Lineage=Root;rootrank;Bacteria;domain;"Chloroflexi";phylum;Anaerolineae;class;Anaerolineales;order;Anaerolineaceae;family;Levilinea;genus	90.59	1.00E-138	494
13	0	0	0	3	0	0	0	0	0	Chloroflexi	Anaerolineae				S000125079 Levilinea saccharolytica (T); KIBI-1 Lineage=Root;rootrank;Bacteria;domain;"Chloroflexi";phylum;Anaerolineae;class;Anaerolineales;order;Anaerolineaceae;family;Levilinea;genus	84.45	2.00E-100	366
1360	0	0	3	4	1	0	0	21	40	Chloroflexi	Anaerolineae				S000622967 Longilinea arvoryzae (T); KOME-1 Lineage=Root;rootrank;Bacteria;domain;"Chloroflexi";phylum;Anaerolineae;class;Anaerolineales;order;Anaerolineaceae;family;Longilinea;genus	82.01	6.00E-62	239
2380	0	0	0	4	4	2	1	0	4	Chloroflexi	Anaerolineae				S000622967 Longilinea arvoryzae (T); KOME-1 Lineage=Root;rootrank;Bacteria;domain;"Chloroflexi";phylum;Anaerolineae;class;Anaerolineales;order;Anaerolineaceae;family;Longilinea;genus	86.86	2.00E-115	416
1974	0	0	0	6	1	0	2	0	0	Chloroflexi	Anaerolineae				S000622967 Longilinea arvoryzae (T); KOME-1 Lineage=Root;rootrank;Bacteria;domain;"Chloroflexi";phylum;Anaerolineae;class;Anaerolineales;order;Anaerolineaceae;family;Longilinea;genus	80.27	4.00E-73	276
26	0	0	0	3	2	0	0	0	0	Chloroflexi	Anaerolineae				S000622967 Longilinea arvoryzae (T); KOME-1 Lineage=Root;rootrank;Bacteria;domain;"Chloroflexi";phylum;Anaerolineae;class;Anaerolineales;order;Anaerolineaceae;family;Longilinea;genus	86.45	1.00E-118	427
784	0	0	0	1	1	0	0	1	1	Chloroflexi	Anaerolineae				S000622967 Longilinea arvoryzae (T); KOME-1 Lineage=Root;rootrank;Bacteria;domain;"Chloroflexi";phylum;Anaerolineae;class;Anaerolineales;order;Anaerolineaceae;family;Longilinea;genus	80.82	4.00E-73	276
2236	0	0	0	0	0	1	0	0	0	Chloroflexi	Anaerolineae				S000629396 Paucisalibacillus globulus (T); type strain: B22 Lineage=Root;rootrank;Bacteria;domain;Firmicutes;phylum;Bacilli;class;Bacillales;order;Bacillaceae 2;family;Paucisalibacillus;genus	81.28	9.00E-80	298
2083	0	0	1	1	0	0	0	0	0	Chloroflexi	Anaerolineae				S002225629 Caldilinea tarbellica; D1-25-10-4 Lineage=Root;rootrank;Bacteria;domain;"Chloroflexi";phylum;Caldilineae;class;Caldilineales;order;Caldilineaceae;family;Caldilinea;genus	83.82	2.00E-95	350
811	0	0	0	1	0	0	0	0	0	Chloroflexi	Anaerolineae				S002232103 Thermanaerothrix daxensis; GNS-1 Lineage=Root;rootrank;Bacteria;domain;"Chloroflexi";phylum;Anaerolineae;class;Anaerolineales;order;Anaerolineaceae;family;Bellilinea;genus	87.47	1.00E-118	427
2190	0	0	0	0	0	0	0	0	1	Chloroflexi	Anaerolineae				S002232103 Thermanaerothrix daxensis; GNS-1 Lineage=Root;rootrank;Bacteria;domain;"Chloroflexi";phylum;Anaerolineae;class;Anaerolineales;order;Anaerolineaceae;family;Bellilinea;genus	88.77	5.00E-127	455
77	0	0	1	3	0	0	0	0	3	Chloroflexi	Anaerolineae				S003291140 Litorilinea aerophila; PRI-4131 Lineage=Root;rootrank;Bacteria;domain;"Chloroflexi";phylum;Caldilineae;class;Caldilineales;order;Caldilineaceae;family;Caldilinea;genus	88.2	1.00E-123	444
691	0	0	0	0	1	0	0	0	0	Chloroflexi	Anaerolineae				S003291140 Litorilinea aerophila; PRI-4131 Lineage=Root;rootrank;Bacteria;domain;"Chloroflexi";phylum;Caldilineae;class;Caldilineales;order;Caldilineaceae;family;Caldilinea;genus	87.47	7.00E-126	451
2935	0	0	2	14	11	9	7	4	10	Chloroflexi	Bljii2	AKYG885	5B-2		S000438509 Moorella glycerini (T); YS6 Lineage=Root;rootrank;Bacteria;domain;Firmicutes;phylum;Clostridia;class;Thermoanaerobacterales;order;Thermoanaerobacteraceae;family;Moorella;genus	86.56	1.00E-112	407
2439	0	0	0	0	1	0	0	0	0	Chloroflexi	Bljii2	AKYG885	5B-2		S000626776 Geoalkalibacter ferrihydriticus (T); Z-0531 Lineage=Root;rootrank;Bacteria;domain;"Proteobacteria";phylum;Deltaproteobacteria;class;Desulfuromonadales;order;Geobacteraceae;family;Geoalkalibacter;genus	84.84	7.00E-101	368
2933	0	0	0	0	1	0	0	0	0	Chloroflexi	Bljii2	AKYG885	5B-2		S003259712 Syntrophothermus lipocalidus DSM 12680; TGB-C1 Lineage=Root;rootrank;Bacteria;domain;Firmicutes;phylum;Clostridia;class;Clostridiales;order;Syntrophomonadaceae;family;Syntrophothermus;genus	84.99	1.00E-102	374
2270	0	0	0	0	2	0	0	0	0	Chloroflexi	Bljii2	AKYG885	Dolo_23		S000012674 Ammoniphilus oxalaticus; RAOx-FF Lineage=Root;rootrank;Bacteria;domain;Firmicutes;phylum;Bacilli;class;Bacillales;order;Paenibacillaceae 2;family;Ammoniphilus;genus	83.21	2.00E-97	357
2349	0	0	3	20	7	3	4	1	6	Chloroflexi	Bljii2	AKYG885	Dolo_23		S000022386 Thermoactinomyces sanguinis; CCUG 38657T Lineage=Root;rootrank;Bacteria;domain;Firmicutes;phylum;Bacilli;class;Bacillales;order;Thermoactinomycetaceae 1;family;unclassified_Thermoactinomycetaceae 1;	83.67	3.00E-100	366
1606	0	0	0	1	0	0	0	0	0	Chloroflexi	Bljii2	AKYG885	Dolo_23		S002166665 Chungangia koreensis; CAU 9163 Lineage=Root;rootrank;Bacteria;domain;Firmicutes;phylum;Bacilli;class;Bacillales;order;unclassified_Bacillales;	82.23	7.00E-91	335
349	0	0	0	0	2	0	0	0	0	Chloroflexi	Bljii2	AKYG885	Dolo_23		S003617114 Bacillus amyloliquefaciens; V1SED3a Lineage=Root;rootrank;Bacteria;domain;Firmicutes;phylum;Bacilli;class;Bacillales;order;Bacillaceae 1;family;Bacillus;genus	84.05	6.00E-102	372
3068	0	0	0	2	1	0	0	0	0	Chloroflexi	Bljii2	B07_WMSP	AKYH90		S001152076 Thermaerobacter composti (T); Ni80 Lineage=Root;rootrank;Bacteria;domain;Firmicutes;phylum;Clostridia;class;Clostridiales;order;Clostridiales_Incertae Sedis XVII;family;Thermaerobacter;genus	85.28	7.00E-111	401
2085	0	0	0	0	3	4	1	1	0	Chloroflexi	Bljii2	B07_WMSP	AKYH90		S002350110 Thermaerobacter marianensis DSM 12885 Lineage=Root;rootrank;Bacteria;domain;Firmicutes;phylum;Clostridia;class;Clostridiales;order;Clostridiales_Incertae Sedis XVII;family;Thermaerobacter;genus	86.36	7.00E-111	401
2091	0	0	0	7	0	0	1	1	0	Chloroflexi	Bljii2	B07_WMSP	AKYH90		S002350110 Thermaerobacter marianensis DSM 12885 Lineage=Root;rootrank;Bacteria;domain;Firmicutes;phylum;Clostridia;class;Clostridiales;order;Clostridiales_Incertae Sedis XVII;family;Thermaerobacter;genus	87.31	3.00E-124	446
2598	0	0	1	0	0	0	0	0	0	Chloroflexi	Bljii2	B07_WMSP	AKYH90		S002440614 Conexibacter arvalis; KV-963 Lineage=Root;rootrank;Bacteria;domain;"Actinobacteria";phylum;Actinobacteria;class;Rubrobacteridae;subclass;Solirubrobacterales;order;Conexibacteraceae;family;Conexibacter;genus	86.21	3.00E-109	396
2575	0	0	4	1	1	3	8	3	2	Chloroflexi	Bljii2	B07_WMSP	FFCH4570		S000383946 Thermanaeromonas toyohensis (T); ToBE Lineage=Root;rootrank;Bacteria;domain;Firmicutes;phylum;Clostridia;class;Thermoanaerobacterales;order;Thermoanaerobacteraceae;family;Thermanaeromonas;genus	84.53	3.00E-99	363
664	0	0	0	1	2	2	0	0	1	Chloroflexi	Bljii2	B07_WMSP	FFCH4570		S002350110 Thermaerobacter marianensis DSM 12885 Lineage=Root;rootrank;Bacteria;domain;Firmicutes;phylum;Clostridia;class;Clostridiales;order;Clostridiales_Incertae Sedis XVII;family;Thermaerobacter;genus	85.28	3.00E-110	399
1265	0	0	0	0	0	0	1	0	1	Chloroflexi	Bljii2	B07_WMSP	FFCH4570		S002350110 Thermaerobacter marianensis DSM 12885 Lineage=Root;rootrank;Bacteria;domain;Firmicutes;phylum;Clostridia;class;Clostridiales;order;Clostridiales_Incertae Sedis XVII;family;Thermaerobacter;genus	83.8	7.00E-101	368
2067	0	0	1	0	0	0	1	0	0	Chloroflexi	Bljii2				S000387930 Thermoactinomyces intermedius (T); KCTC 9646 (type strain) Lineage=Root;rootrank;Bacteria;domain;Firmicutes;phylum;Bacilli;class;Bacillales;order;Thermoactinomycetaceae 1;family;Thermoactinomyces;genus	83.42	7.00E-91	335
1314	0	0	0	1	0	0	0	0	0	Chloroflexi	Bljii2				S000390565 Desulfomonile limimaris; DCB-F Lineage=Root;rootrank;Bacteria;domain;"Proteobacteria";phylum;Deltaproteobacteria;class;Syntrophobacterales;order;Syntrophaceae;family;Desulfomonile;genus	86.41	2.00E-117	424
276	0	0	0	0	0	2	1	1	1	Chloroflexi	Bljii2				S000409563 Candidatus Chlorothrix halophila Lineage=Root;rootrank;Bacteria;domain;"Chloroflexi";phylum;unclassified_"Chloroflexi";	82.23	1.00E-89	331
908	0	0	0	5	3	0	0	2	0	Chloroflexi	Bljii2				S002350110 Thermaerobacter marianensis DSM 12885 Lineage=Root;rootrank;Bacteria;domain;Firmicutes;phylum;Clostridia;class;Clostridiales;order;Clostridiales_Incertae Sedis XVII;family;Thermaerobacter;genus	84.72	7.00E-101	368
293	0	0	0	1	0	0	0	0	0	Chloroflexi	Bljii2				S002350110 Thermaerobacter marianensis DSM 12885 Lineage=Root;rootrank;Bacteria;domain;Firmicutes;phylum;Clostridia;class;Clostridiales;order;Clostridiales_Incertae Sedis XVII;family;Thermaerobacter;genus	84	2.00E-95	350
2721	0	0	0	1	1	2	1	4	2	Chloroflexi	Bljii2				S003259708 Thermomicrobium roseum; P-2 Lineage=Root;rootrank;Bacteria;domain;"Chloroflexi";phylum;Thermomicrobia;class;Thermomicrobiales;order;Thermomicrobiaceae;family;Thermomicrobium;genus	84.3	3.00E-104	379
1863	0	0	7	5	6	2	3	3	2	Chloroflexi	Chloroflexi				S003264347 Ureibacillus thermophilus; NBRC 106535 Lineage=Root;rootrank;Bacteria;domain;Firmicutes;phylum;Bacilli;class;Bacillales;order;Planococcaceae;family;Ureibacillus;genus	80.9	2.00E-76	287
1029	1	1	15	14	14	14	33	15	18	Chloroflexi	Chloroflexi-4				S000018082 Gram-positive bacteria SOGA31 Lineage=Root;rootrank;Bacteria;domain;unclassified_Bacteria;	80.15	6.00E-77	289
1561	0	0	0	1	0	0	0	0	0	Chloroflexi	Chloroflexi-4				S000021406 Oxalophagus oxalicus (T); DSM 5503T Lineage=Root;rootrank;Bacteria;domain;Firmicutes;phylum;Bacilli;class;Bacillales;order;Paenibacillaceae 2;family;Oxalophagus;genus	82.41	7.00E-91	335
2414	0	0	0	0	0	0	1	0	0	Chloroflexi	Chloroflexi-4				S000021406 Oxalophagus oxalicus (T); DSM 5503T Lineage=Root;rootrank;Bacteria;domain;Firmicutes;phylum;Bacilli;class;Bacillales;order;Paenibacillaceae 2;family;Oxalophagus;genus	82.03	3.00E-89	329
2737	0	0	1	0	0	0	0	0	0	Chloroflexi	Chloroflexi-4				S000322757 Dehalobium chlorocoercia DF-1 Lineage=Root;rootrank;Bacteria;domain;"Chloroflexi";phylum;"Dehalococcoidetes";class;Dehalogenimonas;genus	86.22	4.00E-118	425
538	0	0	0	0	0	0	1	0	0	Chloroflexi	Chloroflexi-4				S000322757 Dehalobium chlorocoercia DF-1 Lineage=Root;rootrank;Bacteria;domain;"Chloroflexi";phylum;"Dehalococcoidetes";class;Dehalogenimonas;genus	85.42	2.00E-112	407
937	0	0	0	2	3	0	0	6	5	Chloroflexi	Chloroflexi-4				S000375954 Pelotomaculum terephthalicicum (T); JT Lineage=Root;rootrank;Bacteria;domain;Firmicutes;phylum;Clostridia;class;Clostridiales;order;Peptococcaceae 2;family;Pelotomaculum;genus	84.78	7.00E-101	368
155	0	0	0	0	0	0	1	0	0	Chloroflexi	Chloroflexi-4				S000375954 Pelotomaculum terephthalicicum (T); JT Lineage=Root;rootrank;Bacteria;domain;Firmicutes;phylum;Clostridia;class;Clostridiales;order;Peptococcaceae 2;family;Pelotomaculum;genus	84.55	9.00E-100	364
2784	0	0	3	0	6	1	1	0	0	Chloroflexi	Chloroflexi-4				S000414361 Thermoanaerobacter cellulolyticus; NA 10B Lineage=Root;rootrank;Bacteria;domain;Firmicutes;phylum;Clostridia;class;Clostridiales;order;Clostridiales_Incertae Sedis III;family;Caldicellulosiruptor;genus	84.72	3.00E-99	363
816	0	0	0	0	0	0	0	3	0	Chloroflexi	Chloroflexi-4				S000414361 Thermoanaerobacter cellulolyticus; NA 10B Lineage=Root;rootrank;Bacteria;domain;Firmicutes;phylum;Clostridia;class;Clostridiales;order;Clostridiales_Incertae Sedis III;family;Caldicellulosiruptor;genus	83.84	3.00E-100	366
2450	0	0	0	1	0	0	0	0	1	Chloroflexi	Chloroflexi-4				S000426636 Caldalkalibacillus thermarum (T); HA6 Lineage=Root;rootrank;Bacteria;domain;Firmicutes;phylum;Bacilli;class;Bacillales;order;Bacillales_incertae_sedis;family;Caldalkalibacillus;genus	82.94	4.00E-88	326
3178	0	0	0	0	1	0	0	0	2	Chloroflexi	Chloroflexi-4				S000438509 Moorella glycerini (T); YS6 Lineage=Root;rootrank;Bacteria;domain;Firmicutes;phylum;Clostridia;class;Thermoanaerobacterales;order;Thermoanaerobacteraceae;family;Moorella;genus	86.46	7.00E-116	418
1068	0	1	0	0	0	0	0	0	0	Chloroflexi	Chloroflexi-4				S000438509 Moorella glycerini (T); YS6 Lineage=Root;rootrank;Bacteria;domain;Firmicutes;phylum;Clostridia;class;Thermoanaerobacterales;order;Thermoanaerobacteraceae;family;Moorella;genus	85.95	7.00E-106	385
212	0	0	0	0	0	0	0	1	0	Chloroflexi	Chloroflexi-4				S000438509 Moorella glycerini (T); YS6 Lineage=Root;rootrank;Bacteria;domain;Firmicutes;phylum;Clostridia;class;Thermoanaerobacterales;order;Thermoanaerobacteraceae;family;Moorella;genus	87.27	2.00E-121	436
2578	3	2	1	0	0	3	3	0	2	Chloroflexi	Chloroflexi-4				S000850678 Moorella thermoacetica; AMP Lineage=Root;rootrank;Bacteria;domain;Firmicutes;phylum;Clostridia;class;Thermoanaerobacterales;order;Thermoanaerobacteraceae;family;Moorella;genus	87.87	7.00E-121	435
631	0	0	0	0	0	0	0	0	1	Chloroflexi	Chloroflexi-4				S000584753 Planifilum yunnanense (T); LA5 Lineage=Root;rootrank;Bacteria;domain;Firmicutes;phylum;Bacilli;class;Bacillales;order;Thermoactinomycetaceae 2;family;Planifilum;genus	84.91	7.00E-101	368
2197	0	0	0	0	2	0	0	4	3	Chloroflexi	Chloroflexi-4				S000752077 Moorella perchloratireducens; An10 Lineage=Root;rootrank;Bacteria;domain;Firmicutes;phylum;Clostridia;class;Thermoanaerobacterales;order;Thermoanaerobacteraceae;family;Moorella;genus	85.16	2.00E-107	390
1907	4	10	5	0	3	0	1	0	1	Chloroflexi	Chloroflexi-4				S000858360 Thermosulfidibacter takaii (T); ABI70S6 (= JCM13301, = DSM 17441) Lineage=Root;rootrank;Bacteria;domain;"Aquificae";phylum;Aquificae;class;Aquificales;order;Aquificales_incertae_sedis;family;Thermosulfidibacter;genus	82.51	3.00E-84	313
1941	0	0	0	1	1	10	8	4	10	Chloroflexi	Chloroflexi-4				S000858360 Thermosulfidibacter takaii (T); ABI70S6 (= JCM13301, = DSM 17441) Lineage=Root;rootrank;Bacteria;domain;"Aquificae";phylum;Aquificae;class;Aquificales;order;Aquificales_incertae_sedis;family;Thermosulfidibacter;genus	82.61	9.00E-85	315
387	0	1	0	0	0	0	3	0	0	Chloroflexi	Chloroflexi-4				S000858360 Thermosulfidibacter takaii (T); ABI70S6 (= JCM13301, = DSM 17441) Lineage=Root;rootrank;Bacteria;domain;"Aquificae";phylum;Aquificae;class;Aquificales;order;Aquificales_incertae_sedis;family;Thermosulfidibacter;genus	82.38	3.00E-89	329
175	0	0	13	20	76	64	48	21	29	Chloroflexi	Chloroflexi-4				S000858630 Pelotomaculum thermopropionicum SI Lineage=Root;rootrank;Bacteria;domain;Firmicutes;phylum;Clostridia;class;Clostridiales;order;Peptococcaceae 2;family;Pelotomaculum;genus	83.56	4.00E-93	342
433	0	1	0	0	0	8	1	0	0	Chloroflexi	Chloroflexi-4				S000891994 Carboxydocella ferrireducens; 019 Lineage=Root;rootrank;Bacteria;domain;Firmicutes;phylum;Clostridia;class;Clostridiales;order;Clostridiales_Incertae Sedis XVI;family;Carboxydocella;genus	86.62	3.00E-119	429
2094	6	28	8	0	1	3	4	0	0	Chloroflexi	Chloroflexi-4				S001199628 actinomycete Sa70 Lineage=Root;rootrank;Bacteria;domain;"Actinobacteria";phylum;Actinobacteria;class;Actinobacteridae;subclass;Actinomycetales;order;Pseudonocardineae;suborder;Pseudonocardiaceae;family;Saccharothrix;genus	80.81	2.00E-81	303
270	0	0	1	0	0	0	0	0	0	Chloroflexi	Chloroflexi-4				S001199628 actinomycete Sa70 Lineage=Root;rootrank;Bacteria;domain;"Actinobacteria";phylum;Actinobacteria;class;Actinobacteridae;subclass;Actinomycetales;order;Pseudonocardineae;suborder;Pseudonocardiaceae;family;Saccharothrix;genus	80.76	7.00E-81	302
696	1	5	4	1	2	3	2	8	10	Chloroflexi	Chloroflexi-4				S001350322 Gemmatimonas aurantiaca T-27; T-27 (= NBRC 100505) Lineage=Root;rootrank;Bacteria;domain;"Gemmatimonadetes";phylum;Gemmatimonadetes;class;Gemmatimonadales;order;Gemmatimonadaceae;family;Gemmatimonas;genus	82.99	1.00E-94	348
1178	2	1	7	0	2	1	3	0	0	Chloroflexi	Chloroflexi-4				S002288218 Acidimicrobium ferrooxidans DSM 10331 Lineage=Root;rootrank;Bacteria;domain;"Actinobacteria";phylum;Actinobacteria;class;Acidimicrobidae;subclass;Acidimicrobiales;order;"Acidimicrobineae";suborder;Acidimicrobiaceae;family;Acidimicrobium;genus	80.95	2.00E-77	291
2160	1	13	15	7	13	7	6	14	10	Chloroflexi	Chloroflexi-4				S002288402 Candidatus Koribacter versatilis Ellin345 Lineage=Root;rootrank;Bacteria;domain;"Acidobacteria";phylum;Acidobacteria_Gp1;class;Gp1;genus	83.78	9.00E-95	348
3206	0	0	0	1	0	0	0	0	0	Chloroflexi	Chloroflexi-4				S002957957 Mitsuokella jalaludinii; 1868 Lineage=Root;rootrank;Bacteria;domain;Firmicutes;phylum;Negativicutes;class;Selenomonadales;order;Veillonellaceae;family;Mitsuokella;genus	85.03	9.00E-110	398
1700	0	0	0	0	0	1	0	0	0	Chloroflexi	Chloroflexi-4				S002988186 Desulfosoma profundi; SPDX02-08 Lineage=Root;rootrank;Bacteria;domain;"Proteobacteria";phylum;Deltaproteobacteria;class;Syntrophobacterales;order;Syntrophobacteraceae;family;Desulfosoma;genus	85.2	9.00E-110	398
1451	0	0	0	0	0	0	1	0	3	Chloroflexi	Chloroflexi-4				S003264122 Alicyclobacillus aeris; NBRC 104953 Lineage=Root;rootrank;Bacteria;domain;Firmicutes;phylum;Bacilli;class;Bacillales;order;Alicyclobacillaceae;family;Alicyclobacillus;genus	82.99	3.00E-95	350
1415	83	145	185	28	61	137	193	88	75	Chloroflexi	Chloroflexi-4				S003286846 Moorella thermoacetica; Y73 Lineage=Root;rootrank;Bacteria;domain;Firmicutes;phylum;Clostridia;class;Thermoanaerobacterales;order;Thermoanaerobacteraceae;family;Moorella;genus	86.78	2.00E-111	403
2552	0	1	8	54	22	199	225	25	66	Chloroflexi	Chloroflexi-4				S003286846 Moorella thermoacetica; Y73 Lineage=Root;rootrank;Bacteria;domain;Firmicutes;phylum;Clostridia;class;Thermoanaerobacterales;order;Thermoanaerobacteraceae;family;Moorella;genus	87.06	7.00E-116	418
2225	0	0	0	6	1	5	0	5	0	Chloroflexi	Chloroflexi-4				S003286846 Moorella thermoacetica; Y73 Lineage=Root;rootrank;Bacteria;domain;Firmicutes;phylum;Clostridia;class;Thermoanaerobacterales;order;Thermoanaerobacteraceae;family;Moorella;genus	85.46	2.00E-112	407
2397	0	0	0	0	0	0	1	0	0	Chloroflexi	Chloroflexi-4				S003286846 Moorella thermoacetica; Y73 Lineage=Root;rootrank;Bacteria;domain;Firmicutes;phylum;Clostridia;class;Thermoanaerobacterales;order;Thermoanaerobacteraceae;family;Moorella;genus	87.5	7.00E-126	451
1484	0	0	0	0	0	0	0	0	1	Chloroflexi	Chloroflexi-4				S000626776 Geoalkalibacter ferrihydriticus (T); Z-0531 Lineage=Root;rootrank;Bacteria;domain;"Proteobacteria";phylum;Deltaproteobacteria;class;Desulfuromonadales;order;Geobacteraceae;family;Geoalkalibacter;genus	85.9	2.00E-111	403
2370	52	108	90	6	31	33	51	10	18	Chloroflexi	Chloroflexi-4				S000858360 Thermosulfidibacter takaii (T); ABI70S6 (= JCM13301, = DSM 17441) Lineage=Root;rootrank;Bacteria;domain;"Aquificae";phylum;Aquificae;class;Aquificales;order;Aquificales_incertae_sedis;family;Thermosulfidibacter;genus	84.3	9.00E-95	348
1088	0	0	0	0	0	1	2	0	0	Chloroflexi	Chloroflexi-4				S000858360 Thermosulfidibacter takaii (T); ABI70S6 (= JCM13301, = DSM 17441) Lineage=Root;rootrank;Bacteria;domain;"Aquificae";phylum;Aquificae;class;Aquificales;order;Aquificales_incertae_sedis;family;Thermosulfidibacter;genus	81.51	5.00E-83	309
1714	0	0	0	1	0	0	0	0	0	Chloroflexi	Chloroflexi-4				S000858360 Thermosulfidibacter takaii (T); ABI70S6 (= JCM13301, = DSM 17441) Lineage=Root;rootrank;Bacteria;domain;"Aquificae";phylum;Aquificae;class;Aquificales;order;Aquificales_incertae_sedis;family;Thermosulfidibacter;genus	80.87	3.00E-74	279

831	0	0	0	0	0	0	0	0	1	Chloroflexi	Chloroflexi-4		S002355241 Caldicellulosiruptor kronotskyensis 2002 Lineage=Root;rootrank;Bacteria;domain;Firmicutes;phylum;Clostridia;class;Clostridiales;order;Clostridiales_Incertae Sedis III;family;Caldicellulosiruptor;genus	85.17	3.00E-110	399
117	0	0	0	0	1	0	0	0	0	Chloroflexi	Chloroflexi-4		S002441759 Acidicapsa ligni; WH120 Lineage=Root;rootrank;Bacteria;domain;"Acidobacteria";phylum;Acidobacteria_Gp1;class;Gp1;genus	81.42	1.00E-78	294
938	0	0	0	0	0	0	0	1	0	Chloroflexi	Ktedonobacteria		S001587450 Ktedonobacter racemifer; DSM 44963 Lineage=Root;rootrank;Bacteria;domain;"Chloroflexi";phylum;Ktedonobacteria;class;Ktedonobacterales;order;Ktedonobacteraceae;family;Ktedonobacter;genus	85.03	3.00E-109	396
1548	0	0	0	0	0	0	0	1	0	Chloroflexi	Ktedonobacteria		S001587450 Ktedonobacter racemifer; DSM 44963 Lineage=Root;rootrank;Bacteria;domain;"Chloroflexi";phylum;Ktedonobacteria;class;Ktedonobacterales;order;Ktedonobacteraceae;family;Ktedonobacter;genus	90.82	1.00E-147	523
1931	0	0	0	0	0	0	0	1	0	Chloroflexi	Ktedonobacteria		S001587450 Ktedonobacter racemifer; DSM 44963 Lineage=Root;rootrank;Bacteria;domain;"Chloroflexi";phylum;Ktedonobacteria;class;Ktedonobacterales;order;Ktedonobacteraceae;family;Ktedonobacter;genus	81.91	1.00E-83	311
1817	0	3	3	1	2	3	3	2	2	Chloroflexi	RA3C7		S000128481 Thermoleophilum album; type strain: ATCC 35266 Lineage=Root;rootrank;Bacteria;domain;"Actinobacteria";phylum;Actinobacteria;class;Rubrobacteridae;subclass;Thermoleophilales;order;Thermoleophilaceae;family;Thermoleophilum;genus	86.36	5.00E-112	405
2356	0	0	0	0	0	1	0	0	0	Chloroflexi	RA3C7		S001152076 Thermaerobacter composti (T); Ni80 Lineage=Root;rootrank;Bacteria;domain;Firmicutes;phylum;Clostridia;class;Clostridiales;order;Clostridiales_Incertae Sedis XVII;family;Thermaerobacter;genus	85.5	2.00E-112	407
1277	5	1	75	332	348	59	81	61	233	Chloroflexi	SOGA3		S000018082 Gram-positive bacteria SOGA31 Lineage=Root;rootrank;Bacteria;domain;unclassified_Bacteria;	89.28	2.00E-130	466
629	1	0	60	192	137	101	128	66	120	Chloroflexi	SOGA3		S000018082 Gram-positive bacteria SOGA31 Lineage=Root;rootrank;Bacteria;domain;unclassified_Bacteria;	91.42	1.00E-143	510
2540	0	0	0	13	4	21	34	4	44	Chloroflexi	SOGA3		S000018082 Gram-positive bacteria SOGA31 Lineage=Root;rootrank;Bacteria;domain;unclassified_Bacteria;	90.08	2.00E-135	483
417	0	0	8	24	13	19	20	6	16	Chloroflexi	SOGA3		S000018082 Gram-positive bacteria SOGA31 Lineage=Root;rootrank;Bacteria;domain;unclassified_Bacteria;	97.31	2.00E-180	632
2367	1	0	1	18	9	2	8	11	16	Chloroflexi	SOGA3		S000018082 Gram-positive bacteria SOGA31 Lineage=Root;rootrank;Bacteria;domain;unclassified_Bacteria;	94.09	2.00E-160	566
172	0	0	1	22	14	5	6	1	1	Chloroflexi	SOGA3		S000018082 Gram-positive bacteria SOGA31 Lineage=Root;rootrank;Bacteria;domain;unclassified_Bacteria;	93.55	5.00E-157	555
2423	0	1	0	11	0	3	7	11	8	Chloroflexi	SOGA3		S000018082 Gram-positive bacteria SOGA31 Lineage=Root;rootrank;Bacteria;domain;unclassified_Bacteria;	90.86	2.00E-140	499
247	0	0	0	13	5	0	0	0	2	Chloroflexi	SOGA3		S000018082 Gram-positive bacteria SOGA31 Lineage=Root;rootrank;Bacteria;domain;unclassified_Bacteria;	93.28	2.00E-155	549
2749	5	0	0	0	1	0	0	0	0	Chloroflexi	SOGA3		S000018082 Gram-positive bacteria SOGA31 Lineage=Root;rootrank;Bacteria;domain;unclassified_Bacteria;	90.56	7.00E-146	518
1336	0	0	0	0	0	0	1	3	1	Chloroflexi	SOGA3		S000018082 Gram-positive bacteria SOGA31 Lineage=Root;rootrank;Bacteria;domain;unclassified_Bacteria;	87.63	2.00E-120	433
3066	0	0	0	2	0	0	0	0	0	Chloroflexi	SOGA3		S000018082 Gram-positive bacteria SOGA31 Lineage=Root;rootrank;Bacteria;domain;unclassified_Bacteria;	85.17	7.00E-111	401
1573	0	0	0	0	0	0	0	0	2	Chloroflexi	SOGA3		S000018082 Gram-positive bacteria SOGA31 Lineage=Root;rootrank;Bacteria;domain;unclassified_Bacteria;	94.09	2.00E-160	566
2218	0	0	0	4	0	0	0	0	0	Chloroflexi	SOGA3		S002288227 Thermobaculum terrenum ATCC BAA-798 Lineage=Root;rootrank;Bacteria;domain;"Chloroflexi";phylum;Thermomicrobia;class;unclassified_Thermomicrobia;	84.76	5.00E-102	372
1165	0	0	1	3	11	1	0	1	1	Chloroflexi	SOGA3		S000995672 Thermovorax subterraneus; 70B Lineage=Root;rootrank;Bacteria;domain;Firmicutes;phylum;Clostridia;class;Clostridiales;order;Incertae Sedis III;family;unclassified_Incertae Sedis III;	82.91	3.00E-95	350
2716	0	0	0	5	8	0	0	0	0	Chloroflexi	SOGA3		S001328273 Fervidicola ferrireducens (T); Y170 Lineage=Root;rootrank;Bacteria;domain;Firmicutes;phylum;Clostridia;class;Clostridiales;order;Incertae Sedis III;family;Fervidicola;genus	83.47	4.00E-93	342
1428	0	32	1	0	1	0	1	2	0	Chloroflexi	Thermomicrobia		S002289810 Sphaerobacter thermophilus DSM 20745 Lineage=Root;rootrank;Bacteria;domain;"Chloroflexi";phylum;Thermomicrobia;class;Sphaerobacteridae;subclass;Sphaerobacterales;order;"Sphaerobacterineae";suborder;Sphaerobacteraceae;family;Sphaerobacter;genus	93.05	1.00E-153	544
2581	1	0	0	0	0	0	0	0	0	Chloroflexi	Thermomicrobia		S002289810 Sphaerobacter thermophilus DSM 20745 Lineage=Root;rootrank;Bacteria;domain;"Chloroflexi";phylum;Thermomicrobia;class;Sphaerobacteridae;subclass;Sphaerobacterales;order;"Sphaerobacterineae";suborder;Sphaerobacteraceae;family;Sphaerobacter;genus	100	0	688
1079	0	0	0	1	0	0	0	0	1	Chloroflexi	Thermomicrobia		S002289810 Sphaerobacter thermophilus DSM 20745 Lineage=Root;rootrank;Bacteria;domain;"Chloroflexi";phylum;Thermomicrobia;class;Sphaerobacteridae;subclass;Sphaerobacterales;order;"Sphaerobacterineae";suborder;Sphaerobacteraceae;family;Sphaerobacter;genus	94.89	8.00E-165	580
1493	0	1	0	0	0	0	0	0	0	Chloroflexi	Thermomicrobia		S002289810 Sphaerobacter thermophilus DSM 20745 Lineage=Root;rootrank;Bacteria;domain;"Chloroflexi";phylum;Thermomicrobia;class;Sphaerobacteridae;subclass;Sphaerobacterales;order;"Sphaerobacterineae";suborder;Sphaerobacteraceae;family;Sphaerobacter;genus	95.44	1.00E-168	593
480	0	0	1	2	3	0	1	2	3	Chloroflexi	Thermomicrobia		S003290740 Nitrolancetus hollandicus Lb Lineage=Root;rootrank;Bacteria;domain;"Chloroflexi";phylum;Thermomicrobia;class;Sphaerobacteridae;subclass;Sphaerobacterales;order;"Sphaerobacterineae";suborder;Sphaerobacteraceae;family;Sphaerobacter;genus	94.89	2.00E-165	582
3122	0	1	0	6	4	0	0	0	1	Chloroflexi	Thermomicrobia	HN-5	S000460056 Sphaerobacter thermophilus; 165-7 Lineage=Root;rootrank;Bacteria;domain;"Chloroflexi";phylum;Thermomicrobia;class;Sphaerobacteridae;subclass;Sphaerobacterales;order;"Sphaerobacterineae";suborder;Sphaerobacteraceae;family;Sphaerobacter;genus	96.54	1.00E-133	477
1223	0	0	0	2	0	2	0	0	0	Chloroflexi	Thermomicrobia	HN-5	S000460056 Sphaerobacter thermophilus; 165-7 Lineage=Root;rootrank;Bacteria;domain;"Chloroflexi";phylum;Thermomicrobia;class;Sphaerobacteridae;subclass;Sphaerobacterales;order;"Sphaerobacterineae";suborder;Sphaerobacteraceae;family;Sphaerobacter;genus	94.12	5.00E-122	438
3188	10	123	16	6	17	31	59	63	52	Chloroflexi	Thermomicrobia	HN-5	S000460057 Sphaerobacter thermophilus; 185-3 Lineage=Root;rootrank;Bacteria;domain;unclassified_Bacteria;	92.56	4.00E-133	475
2538	51	0	0	1	0	0	0	0	0	Chloroflexi	Thermomicrobia	HN-5	S000460057 Sphaerobacter thermophilus; 185-3 Lineage=Root;rootrank;Bacteria;domain;unclassified_Bacteria;	90.99	2.00E-125	449
1980	5	1	42	16	5	5	6	2	2	Chloroflexi	Thermomicrobia	HN-5	S000460057 Sphaerobacter thermophilus; 185-3 Lineage=Root;rootrank;Bacteria;domain;unclassified_Bacteria;	92.51	4.00E-133	475
2062	2	0	3	21	10	8	5	28	15	Chloroflexi	Thermomicrobia	HN-5	S000460057 Sphaerobacter thermophilus; 185-3 Lineage=Root;rootrank;Bacteria;domain;unclassified_Bacteria;	93.41	1.00E-138	494
969	17	11	0	0	0	2	5	0	0	Chloroflexi	Thermomicrobia	HN-5	S000460057 Sphaerobacter thermophilus; 185-3 Lineage=Root;rootrank;Bacteria;domain;unclassified_Bacteria;	91.42	5.00E-127	455
1491	0	0	3	5	7	12	27	5	3	Chloroflexi	Thermomicrobia	HN-5	S000460057 Sphaerobacter thermophilus; 185-3 Lineage=Root;rootrank;Bacteria;domain;unclassified_Bacteria;	91.29	5.00E-127	455
1962	0	2	4	7	3	1	5	1	6	Chloroflexi	Thermomicrobia	HN-5	S000460057 Sphaerobacter thermophilus; 185-3 Lineage=Root;rootrank;Bacteria;domain;unclassified_Bacteria;	93.71	2.00E-140	499
916	0	0	1	13	1	0	0	0	0	Chloroflexi	Thermomicrobia	HN-5	S000460057 Sphaerobacter thermophilus; 185-3 Lineage=Root;rootrank;Bacteria;domain;unclassified_Bacteria;	94.31	1.00E-143	510
1719	0	0	1	3	1	1	6	0	2	Chloroflexi	Thermomicrobia	HN-5	S000460057 Sphaerobacter thermophilus; 185-3 Lineage=Root;rootrank;Bacteria;domain;unclassified_Bacteria;	93.09	5.00E-137	488
2145	0	0	1	3	4	1	0	1	0	Chloroflexi	Thermomicrobia	HN-5	S000460057 Sphaerobacter thermophilus; 185-3 Lineage=Root;rootrank;Bacteria;domain;unclassified_Bacteria;	94.91	5.00E-147	521
1342	0	0	0	0	0	0	0	4	2	Chloroflexi	Thermomicrobia	HN-5	S000460057 Sphaerobacter thermophilus; 185-3 Lineage=Root;rootrank;Bacteria;domain;unclassified_Bacteria;	92.24	5.00E-132	472
1026	0	0	0	3	1	0	0	0	0	Chloroflexi	Thermomicrobia	HN-5	S000460057 Sphaerobacter thermophilus; 185-3 Lineage=Root;rootrank;Bacteria;domain;unclassified_Bacteria;	91.92	2.00E-130	466
1874	0	0	3	0	0	0	0	0	0	Chloroflexi	Thermomicrobia	HN-5	S000460057 Sphaerobacter thermophilus; 185-3 Lineage=Root;rootrank;Bacteria;domain;unclassified_Bacteria;	91.67	4.00E-128	459
777	0	0	0	0	2	0	0	0	0	Chloroflexi	Thermomicrobia	HN-5	S000460057 Sphaerobacter thermophilus; 185-3 Lineage=Root;rootrank;Bacteria;domain;unclassified_Bacteria;	92.24	2.00E-131	470
1123	0	0	0	1	0	0	0	0	0	Chloroflexi	Thermomicrobia	HN-5	S000460057 Sphaerobacter thermophilus; 185-3 Lineage=Root;rootrank;Bacteria;domain;unclassified_Bacteria;	94.29	1.00E-143	510
3149	0	0	0	1	0	0	0	0	0	Chloroflexi	Thermomicrobia	HN-5	S000460057 Sphaerobacter thermophilus; 185-3 Lineage=Root;rootrank;Bacteria;domain;unclassified_Bacteria;	92.24	2.00E-131	470
587	0	0	0	0	0	0	0	1	0	Chloroflexi	Thermomicrobia	HN-5	S000460057 Sphaerobacter thermophilus; 185-3 Lineage=Root;rootrank;Bacteria;domain;unclassified_Bacteria;	97.91	1.00E-163	577
2482	0	0	0	0	0	0	0	0	1	Chloroflexi	Thermomicrobia	HN-5	S000460057 Sphaerobacter thermophilus; 185-3 Lineage=Root;rootrank;Bacteria;domain;unclassified_Bacteria;	93.41	4.00E-138	492
896	1	2	7	35	43	14	12	5	10	Chloroflexi	Thermomicrobia	HN-5	S000460079 Thermomicrobium roseum; 125-2 Lineage=Root;rootrank;Bacteria;domain;"Chloroflexi";phylum;Thermomicrobia;class;Sphaerobacteridae;subclass;Sphaerobacterales;order;"Sphaerobacterineae";suborder;Sphaerobacteraceae;family;Sphaerobacter;genus	98.01	1.00E-172	606
3079	3	1	12	27	21	5	8	10	7	Chloroflexi	Thermomicrobia	HN-5	S000460079 Thermomicrobium roseum; 125-2 Lineage=Root;rootrank;Bacteria;domain;"Chloroflexi";phylum;Thermomicrobia;class;Sphaerobacteridae;subclass;Sphaerobacterales;order;"Sphaerobacterineae";suborder;Sphaerobacteraceae;family;Sphaerobacter;genus	95.11	3.00E-154	545
2180	0	9	7	1	6	16	32	7	0	Chloroflexi	Thermomicrobia	HN-5	S000460079 Thermomicrobium roseum; 125-2 Lineage=Root;rootrank;Bacteria;domain;"Chloroflexi";phylum;Thermomicrobia;class;Sphaerobacteridae;subclass;Sphaerobacterales;order;"Sphaerobacterineae";suborder;Sphaerobacteraceae;family;Sphaerobacter;genus	90.17	2.00E-125	449
2158	0	0	4	10	9	24	21	5	4	Chloroflexi	Thermomicrobia	HN-5	S000460079 Thermomicrobium roseum; 125-2 Lineage=Root;rootrank;Bacteria;domain;"Chloroflexi";phylum;Thermomicrobia;class;Sphaerobacteridae;subclass;Sphaerobacterales;order;"Sphaerobacterineae";suborder;Sphaerobacteraceae;family;Sphaerobacter;genus	92.07	1.00E-137	490
1264	0	0	1	2	4	6	5	3	0	Chloroflexi	Thermomicrobia	HN-5	S000460079 Thermomicrobium roseum; 125-2 Lineage=Root;rootrank;Bacteria;domain;"Chloroflexi";phylum;Thermomicrobia;class;Sphaerobacteridae;subclass;Sphaerobacterales;order;"Sphaerobacterineae";suborder;Sphaerobacteraceae;family;Sphaerobacter;genus	90.62	4.00E-128	459
234	0	0	1	1	5	0	1	7	5	Chloroflexi	Thermomicrobia	HN-5	S000460079 Thermomicrobium roseum; 125-2 Lineage=Root;rootrank;Bacteria;domain;"Chloroflexi";phylum;Thermomicrobia;class;Sphaerobacteridae;subclass;Sphaerobacterales;order;"Sphaerobacterineae";suborder;Sphaerobacteraceae;family;Sphaerobacter;genus	94.3	6.00E-151	534
374	0	6	0	0	0	1	1	0	0	Chloroflexi	Thermomicrobia	HN-5	S000460079 Thermomicrobium roseum; 125-2 Lineage=Root;rootrank;Bacteria;domain;"Chloroflexi";phylum;Thermomicrobia;class;Sphaerobacteridae;subclass;Sphaerobacterales;order;"Sphaerobacterineae";suborder;Sphaerobacteraceae;family;Sphaerobacter;genus	91.78	2.00E-135	483
1623	0	0	0	5	0	0	0	0	0	Chloroflexi	Thermomicrobia	HN-5	S000460079 Thermomicrobium roseum; 125-2 Lineage=Root;rootrank;Bacteria;domain;"Chloroflexi";phylum;Thermomicrobia;class;Sphaerobacteridae;subclass;Sphaerobacterales;order;"Sphaerobacterineae";suborder;Sphaerobacteraceae;family;Sphaerobacter;genus	93.2	3.00E-144	512
1592	0	0	0	0	0	1	0	1	0	Chloroflexi	Thermomicrobia	HN-5	S000460079 Thermomicrobium roseum; 125-2 Lineage=Root;rootrank;Bacteria;domain;"Chloroflexi";phylum;Thermomicrobia;class;Sphaerobacteridae;subclass;Sphaerobacterales;order;"Sphaerobacterineae";suborder;Sphaerobacteraceae;family;Sphaerobacter;genus	91.76	6.00E-136	484
2870	0	0	0	0	1	0	0	0	0	Chloroflexi	Thermomicrobia	HN-5	S000460079 Thermomicrobium roseum; 125-2 Lineage=Root;rootrank;Bacteria;domain;"Chloroflexi";phylum;Thermomicrobia;class;Sphaerobacteridae;subclass;Sphaerobacterales;order;"Sphaerobacterineae";suborder;Sphaerobacteraceae;family;Sphaerobacter;genus	89.49	2.00E-122	440
447	7	0	0	0	0	0	0	0	0	Chloroflexi	Thermomicrobia	HN-5	S001093971 Bacillus halochares (T); MSS4 Lineage=Root;rootrank;Bacteria;domain;Firmicutes;phylum;Bacilli;class;Bacillales;order;Bacillaceae 1;family;Bacillus;genus	98.12	0	649
2300	0	0	0	1	2	0	0	0	3	Chloroflexi	Thermomicrobia	HN-5	S002289810 Sphaerobacter thermophilus DSM 20745 Lineage=Root;rootrank;Bacteria;domain;"Chloroflexi";phylum;Thermomicrobia;class;Sphaerobacteridae;subclass;Sphaerobacterales;order;"Sphaerobacterineae";suborder;Sphaerobacteraceae;family;Sphaerobacter;genus	88.2	4.00E-123	442
423	91	348	159	2	56	24	31	71	18	Chloroflexi	Thermomicrobia	Thermomicrobiales	S002289810 Sphaerobacter thermophilus DSM 20745 Lineage=Root;rootrank;Bacteria;domain;"Chloroflexi";phylum;Thermomicrobia;class;Sphaerobacteridae;subclass;Sphaerobacterales;order;"Sphaerobacterineae";suborder;Sphaerobacteraceae;family;Sphaerobacter;genus	89.3	8.00E-130	464
1540	51	66	33	0	6	23	24	3	1	Chloroflexi	Thermomicrobia	Thermomicrobiales	S002289810 Sphaerobacter thermophilus DSM 20745 Lineage=Root;rootrank;Bacteria;domain;"Chloroflexi";phylum;Thermomicrobia;class;Sphaerobacteridae;subclass;Sphaerobacterales;order;"Sphaerobacterineae";suborder;Sphaerobacteraceae;family;Sphaerobacter;genus	90.59	4.00E-138	492
3143	0	1	11	11	31	56	57	8	6	Chloroflexi	Thermomicrobia	Thermomicrobiales	S002289810 Sphaerobacter thermophilus DSM 20745 Lineage=Root;rootrank;Bacteria;domain;"Chloroflexi";phylum;Thermomicrobia;class;Sphaerobacteridae;subclass;Sphaerobacterales;order;"Sphaerobacterineae";suborder;Sphaerobacteraceae;family;Sphaerobacter;genus	89.25	2.00E-130	466
2756	0	0	3	35	28	3	9	17	37	Chloroflexi	Thermomicrobia	Thermomicrobiales	S002289810 Sphaerobacter thermophilus DSM 20745 Lineage=Root;rootrank;Bacteria;domain;"Chloroflexi";phylum;Thermomicrobia;class;Sphaerobacteridae;subclass;Sphaerobacterales;order;"Sphaerobacterineae";suborder;Sphaerobacteraceae;family;Sphaerobacter;genus	89.81	1.00E-133	477
1361	2	0	0	2	0	19	30	1	16	Chloroflexi	Thermomicrobia	Thermomicrobiales	S002289810 Sphaerobacter thermophilus DSM 20745 Lineage=Root;rootrank;Bacteria;domain;"Chloroflexi";phylum;Thermomicrobia;class;Sphaerobacteridae;subclass;Sphaerobacterales;order;"Sphaerobacterineae";suborder;Sphaerobacteraceae;family;Sphaerobacter;genus	89.84	1.00E-133	477
725	0	4	10	10	4	0	0	6	9	Chloroflexi	Thermomicrobia	Thermomicrobiales	S002289810 Sphaerobacter thermophilus DSM 20745 Lineage=Root;rootrank;Bacteria;domain;"Chloroflexi";phylum;Thermomicrobia;class;Sphaerobacteridae;subclass;Sphaerobacterales;order;"Sphaerobacterineae";suborder;Sphaerobacteraceae;family;Sphaerobacter;genus	89.81	1.00E-133	477
2651	6	1	0	1	0	9	8	0	1	Chloroflexi	Thermomicrobia	Thermomicrobiales	S002289810 Sphaerobacter thermophilus DSM 20745 Lineage=Root;rootrank;Bacteria;domain;"Chloroflexi";phylum;Thermomicrobia;class;Sphaerobacteridae;subclass;Sphaerobacterales;order;"Sphaerobacterineae";suborder;Sphaerobacteraceae;family;Sphaerobacter;genus	89.78	4.00E-133	475
2496	0	0	1	1	2	1	3	6	4	Chloroflexi	Thermomicrobia	Thermomicrobiales	S002289810 Sphaerobacter thermophilus DSM 20745 Lineage=Root;rootrank;Bacteria;domain;"Chloroflexi";phylum;Thermomicrobia;class;Sphaerobacteridae;subclass;Sphaerobacterales;order;"Sphaerobacterineae";suborder;Sphaerobacteraceae;family;Sphaerobacter;genus	87.97	2.00E-121	436
182	0	0	4	1	0	0	0	0	0	Chloroflexi	Thermomicrobia	Thermomicrobiales	S002289810 Sphaerobacter thermophilus DSM 20745 Lineage=Root;rootrank;Bacteria;domain;"Chloroflexi";phylum;Thermomicrobia;class;Sphaerobacteridae;subclass;Sphaerobacterales;order;"Sphaerobacterineae";suborder;Sphaerobacteraceae;family;Sphaerobacter;genus	87.97	5.00E-122	438
617	0	0	0	0	0	0	0	3	2	Chloroflexi	Thermomicrobia	Thermomicrobiales	S002289810 Sphaerobacter thermophilus DSM 20745 Lineage=Root;rootrank;Bacteria;domain;"Chloroflexi";phylum;Thermomicrobia;class;Sphaerobacteridae;subclass;Sphaerobacterales;order;"Sphaerobacterineae";suborder;Sphaerobacteraceae;family;Sphaerobacter;genus	88.47	8.00E-125	448
1789	0	0	0	0	0	0	0	4	0	Chloroflexi	Thermomicrobia	Thermomicrobiales	S002289810 Sphaerobacter thermophilus DSM 20745 Lineage=Root;rootrank;Bacteria;domain;"Chloroflexi";phylum;Thermomicrobia;class;Sphaerobacteridae;subclass;Sphaerobacterales;order;"Sphaerobacterineae";suborder;Sphaerobacteraceae;family;Sphaerobacter;genus	88.24	1.00E-123	444
299	0	0	0	1	0	0	0	0	0	Chloroflexi	Thermomicrobia	Thermomicrobiales	S002289810 Sphaerobacter thermophilus DSM 20745 Lineage=Root;rootrank;Bacteria;domain;"Chloroflexi";phylum;Thermomicrobia;class;Sphaerobacteridae;subclass;Sphaerobacterales;order;"Sphaerobacterineae";suborder;Sphaerobacteraceae;family;Sphaerobacter;genus	87.23	1.00E-117	424
771	0	1	2	6	10	0	1	0	0	Chloroflexi	Thermomicrobia	Thermomicrobiales	S003290740 Nitrolancetus hollandicus Lb Lineage=Root;rootrank;Bacteria;domain;"Chloroflexi";phylum;Thermomicrobia;class;Sphaerobacteridae;subclass;Sphaerobacterales;order;"Sphaerobacterineae";suborder;Sphaerobacteraceae;family;Sphaerobacter;genus	87.9	5.00E-122	438
1827	1	0	0	0	0	0	0	0	0	Chloroflexi	Thermomicrobia		S000460057 Sphaerobacter thermophilus; 185-3 Lineage=Root;rootrank;Bacteria;domain;unclassified_Bacteria;	90.15	9.00E-120	431
993	0	0	0	4	1	0	0	0	0	Chloroflexi	Thermomicrobia		S002289810 Sphaerobacter thermophilus DSM 20745 Lineage=Root;rootrank;Bacteria;domain;"Chloroflexi";phylum;Thermomicrobia;class;Sphaerobacteridae;subclass;Sphaerobacterales;order;"Sphaerobacterineae";suborder;Sphaerobacteraceae;family;Sphaerobacter;genus	84.45	2.00E-100	366
353	0	0	0	0	1	0	0	0	1	Chloroflexi	Thermomicrobia		S002289810 Sphaerobacter thermophilus DSM 20745 Lineage=Root;rootrank;Bacteria;domain;"Chloroflexi";phylum;Thermomicrobia;class;Sphaerobacteridae;subclass;Sphaerobacterales;order;"Sphaerobacterineae";suborder;Sphaerobacteraceae;family;Sphaerobacter;genus	84.84	5.00E-102	372
1726	0	1	37	18	12	4	20	219	85	Chloroflexi	Thermomicrobia		S003290740 Nitrolancetus hollandicus Lb Lineage=Root;rootrank;Bacteria;domain;"Chloroflexi";phylum;Thermomicrobia;class;Sphaerobacteridae;subclass;Sphaerobacterales;order;"Sphaerobacterineae";suborder;Sphaerobacteraceae;family;Sphaerobacter;genus	88.17	1.00E-123	444
2483	0	0	0	0	0	0	0	1	7	Chloroflexi	Thermomicrobia		S003290740 Nitrolancetus hollandicus Lb Lineage=Root;rootrank;Bacteria;domain;"Chloroflexi";phylum;Thermomicrobia;class;Sphaerobacteridae;subclass;Sphaerobacterales;order;"Sphaerobacterineae";suborder;Sphaerobacteraceae;family;Sphaerobacter;genus	93.28	8.00E-155	547
1156	0	0	0	0	0	0	3	0	0	Chloroflexi	Thermomicrobia		S003290740 Nitrolancetus hollandicus Lb Lineage=Root;rootrank;Bacteria;domain;"Chloroflexi";phylum;Thermomicrobia;class;Sphaerobacteridae;subclass;Sphaerobacterales;order;"Sphaerobacterineae";suborder;Sphaerobacteraceae;family;Sphaerobacter;genus	92.76	5.00E-152	538
2462	0	0	0	0	0	0	0	1	0	Chloroflexi	Thermomicrobia		S003290740 Nitrolancetus hollandicus Lb Lineage=Root;rootrank;Bacteria;domain;"Chloroflexi";phylum;Thermomicrobia;class;Sphaerobacteridae;subclass;Sphaerobacterales;order;"Sphaerobacterineae";suborder;Sphaerobacteraceae;family;Sphaerobacter;genus	90.64	1.00E-138	494
1403	10	33	23	0	5	9	9	10	1	Chloroflexi	TK7		S000012674 Ammoniphilus oxalaticus; RAOx-FF Lineage=Root;rootrank;Bacteria;domain;Firmicutes;phylum;Bacilli;class;Bacillales;order;Paenibacillaceae 2;family;Ammoniphilus;genus	85.25	1.00E-103	377
2068	0	0	2	0	2	0	1	0	2	Chloroflexi	TK7		S000012674 Ammoniphilus oxalaticus; RAOx-FF Lineage=Root;rootrank;Bacteria;domain;Firmicutes;phylum;Bacilli;class;Bacillales;order;Paenibacillaceae 2;family;Ammoniphilus;genus	86.87	3.00E-120	433
1346	0	0	0	1	0	0	1	0	0	Chloroflexi	TK7		S000012674 Ammoniphilus oxalaticus; RAOx-FF Lineage=Root;rootrank;Bacteria;domain;Firmicutes;phylum;Bacilli;class;Bacillales;order;Paenibacillaceae 2;family;Ammoniphilus;genus	84.69	7.00E-106	385
501	7	39	17	4	5	7	9	9	17	Chloroflexi	TK7		S000013381 Ammoniphilus oxalivorans (T); RAOx-FS Lineage=Root;rootrank;Bacteria;domain;Firmicutes;phylum;Bacilli;class;Bacillales;order;Paenibacillaceae 2;family;Ammoniphilus;genus	85.56	2.00E-105	383
690	0	0	2	4	18	6	9	3	3	Chloroflexi	TK7		S000013381 Ammoniphilus oxalivorans (T); RAOx-FS Lineage=Root;rootrank;Bacteria;domain;Firmicutes;phylum;Bacilli;class;Bacillales;order;Paenibacillaceae 2;family;Ammoniphilus;genus	85.71	3.00E-114	412
3169	0	0	0	0	1	0	0	2	3	Chloroflexi	TK7		S000013381 Ammoniphilus oxalivorans (T); RAOx-FS Lineage=Root;rootrank;Bacteria;domain;Firmicutes;phylum;Bacilli;class;Bacillales;order;Paenibacillaceae 2;family;Ammoniphilus;genus	85.07	5.00E-102	372
1695	0	0	1	0	0	0	0	2	2	Chloroflexi	TK7		S000013381 Ammoniphilus oxalivorans (T); RAOx-FS Lineage=Root;rootrank;Bacteria;domain;Firmicutes;phylum;Bacilli;class;Bacillales;order;Paenibacillaceae 2;family;Ammoniphilus;genus	85.03	5.00E-102	372
904	0	0	0	2	0	0	0	1	1	Chloroflexi	TK7		S000013381 Ammoniphilus oxalivorans (T); RAOx-FS Lineage=Root;rootrank;Bacteria;domain;Firmicutes;phylum;Bacilli;class;Bacillales;order;Paenibacillaceae 2;family;Ammoniphilus;genus	85.61	6.00E-112	405
45	0	0	0	1	1	0	0	0	0	Chloroflexi	TK7		S000013381 Ammoniphilus oxalivorans (T); RAOx-FS Lineage=Root;rootrank;Bacteria;domain;Firmicutes;phylum;Bacilli;class;Bacillales;order;Paenibacillaceae 2;family;Ammoniphilus;genus	84.96	3.00E-109	396
31	0	0	0	1	0	0	0	0	0	Chloroflexi	TK7		S000013381 Ammoniphilus oxalivorans (T); RAOx-FS Lineage=Root;rootrank;Bacteria;domain;Firmicutes;phylum;Bacilli;class;Bacillales;order;Paenibacillaceae 2;family;Ammoniphilus;genus	85.68	2.00E-105	383
1910	0	0	0	1	0	0	0	0	0	Chloroflexi	TK7		S000021406 Oxalophagus oxalicus (T); DSM 5503T Lineage=Root;rootrank;Bacteria;domain;Firmicutes;phylum;Bacilli;class;Bacillales;order;Paenibacillaceae 2;family;Oxalophagus;genus	84.09	2.00E-101	370
2211	0	0	1	6	0	0	0	2	3	Chloroflexi	TK7		S000391487 Gelria glutamica (T); TGO Lineage=Root;rootrank;Bacteria;domain;Firmicutes;phylum;Clostridia;class;Thermoanaerobacterales;order;Thermoanaerobacteraceae;family;Gelria;genus	85.22	3.00E-104	379
2941	7	10	9	4	2	1	1	5	4	Chloroflexi	TK7		S000437163 Heliobacillus mobilis Lineage=Root;rootrank;Bacteria;domain;Firmicutes;phylum;Clostridia;class;Clostridiales;order;Heliobacteriaceae;family;Heliobacillus;genus	84.95	7.00E-111	401
1813	0	0	14	78	53	94	188	68	121	Chloroflexi	TK7		S000438509 Moorella glycerini (T); YS6 Lineage=Root;rootrank;Bacteria;domain;Firmicutes;phylum;Clostridia;class;Thermoanaerobacterales;order;Thermoanaerobacteraceae;family;Moorella;genus	86.33	7.00E-111	401
2966	0	0	0	3	2	0	0	20	10	Chloroflexi	TK7		S000438509 Moorella glycerini (T); YS6 Lineage=Root;rootrank;Bacteria;domain;Firmicutes;phylum;Clostridia;class;Thermoanaerobacterales;order;Thermoanaerobacteraceae;family;Moorella;genus	86.56	1.00E-112	407
2561	3	10	3	1	0	0	3	2	0	Chloroflexi	TK7		S000438509 Moorella glycerini (T); YS6 Lineage=Root;rootrank;Bacteria;domain;Firmicutes;phylum;Clostridia;class;Thermoanaerobacterales;order;Thermoanaerobacteraceae;family;Moorella;genus	84.34	9.00E-105	381
2020	0	0	1	4	4	3	1	1	12	Chloroflexi	TK7		S001745278 Bacillus saliphilus; M1S6-14 Lineage=Root;rootrank;Bacteria;domain;Firmicutes;phylum;Bacilli;class;Bacillales;order;Sporolactobacillaceae;family;Sporolactobacillaceae_incertae_sedis;genus	85.25	3.00E-104	379
1046	0	0	0	1	0	1	0	0	0	Chloroflexi	TK7		S002234876 Bacillus halodurans; TSLV1 Lineage=Root;rootrank;Bacteria;domain;Firmicutes;phylum;Bacilli;class;Bacillales;order;Bacillaceae 1;family;Bacillus;genus	84.59	9.00E-100	364
146	0	0	0	0	0	0	0	0	1	Chloroflexi	TK7		S002449032 Geobacillus stearothermophilus; P4 Lineage=Root;rootrank;Bacteria;domain;Firmicutes;phylum;Bacilli;class;Bacillales;order;Bacillaceae 1;family;Geobacillus;genus	85.11	5.00E-102	372
246	0	0	0	15	8	3	6	2	5	Chloroflexi	TK7		S003264045 Mechercharimyces mesophilus; NBRC 104191 Lineage=Root;rootrank;Bacteria;domain;Firmicutes;phylum;Bacilli;class;Bacillales;order;Thermoactinomycetaceae 2;family;Mechercharimyces;genus	86.02	1.00E-108	394
1601	0	0	0	0	1	0	0	0	0	Chloroflexi	TK7		S003614554 Aneurinibacillus danicus; M35 Lineage=Root;rootrank;Bacteria;domain;Firmicutes;phylum;Bacilli;class;Bacillales;order;Paenibacillaceae 2;family;Aneurinibacillus;genus	83.42	4.00E-93	342
1530	0	0	0	0	0	0	0	0	1	Chloroflexi	TK7		S003659903 Marinococcus luteus; KCTC 13214 Lineage=Root;rootrank;Bacteria;domain;Firmicutes;phylum;Bacilli;class;Bacillales;order;Bacillaceae 2;family;Marinococcus;genus	83.83	9.00E-95	348
3167	0	0	1	3	2	0	0	2	2	Chloroflexi	TK7		S003709934 Calderihabitans maritimus; KKC1 Lineage=Root;rootrank;Bacteria;domain;Firmicutes;phylum;Clostridia;class;Thermoanaerobacterales;order;Thermoanaerobacteraceae;family;unclassified_Thermoanaerobacteraceae;	85.9	1.00E-107	390
80	0	3	45	4	15	17	41	19	24	Chloroflexi	TK7		S003718041 Bacillus gibsonii; G1-6-20 Lineage=Root;rootrank;Bacteria;domain;Firmicutes;phylum;Bacilli;class;Bacillales;order;Bacillaceae 1;family;Bacillus;genus	85.25	3.00E-104	379
2065	5	5	51	20	37	20	21	18	9	Chloroflexi	TK7	mle-48	S000000782 Ammoniphilus oxalaticus (T); RAOx1 Lineage=Root;rootrank;Bacteria;domain;Firmicutes;phylum;Bacilli;class;Bacillales;order;Paenibacillaceae 2;family;Ammoniphilus;genus	84.57	1.00E-98	361
1571	0	0	1	27	16	31	28	9	8	Chloroflexi	TK7	mle-48	S000000782 Ammoniphilus oxalaticus (T); RAOx1 Lineage=Root;rootrank;Bacteria;domain;Firmicutes;phylum;Bacilli;class;Bacillales;order;Paenibacillaceae 2;family;Ammoniphilus;genus	86.17	1.00E-108	394
1617	0	0	0	6	34	9	10	2	16	Chloroflexi	TK7	mle-48	S000000782 Ammoniphilus oxalaticus (T); RAOx1 Lineage=Root;rootrank;Bacteria;domain;Firmicutes;phylum;Bacilli;class;Bacillales;order;Paenibacillaceae 2;family;Ammoniphilus;genus	84.62	1.00E-98	361
2198	0	0	0	6	2	0	0	0	3	Chloroflexi	TK7	mle-48	S000000782 Ammoniphilus oxalaticus (T); RAOx1 Lineage=Root;rootrank;Bacteria;domain;Firmicutes;phylum;Bacilli;class;Bacillales;order;Paenibacillaceae 2;family;Ammoniphilus;genus	85.1	1.00E-108	394
1775	0	0	0	0	0	0	0	0	1	Chloroflexi	TK7	mle-48	S000000782 Ammoniphilus oxalaticus (T); RAOx1 Lineage=Root;rootrank;Bacteria;domain;Firmicutes;phylum;Bacilli;class;Bacillales;order;Paenibacillaceae 2;family;Ammoniphilus;genus	87.2	2.00E-115	416
2038	0	0	0	0	0	0	0	0	1	Chloroflexi	TK7	mle-48	S000000782 Ammoniphilus oxalaticus (T); RAOx1 Lineage=Root;rootrank;Bacteria;domain;Firmicutes;phylum;Bacilli;class;Bacillales;order;Paenibacillaceae 2;family;Ammoniphilus;genus	85.87	5.00E-107	388
535	0	0	0	0	0	0	1	0	0	Chloroflexi	TK7	mle-48	S000426636 Caldalkalibacillus thermarum (T); HA6 Lineage=Root;rootrank;Bacteria;domain;Firmicutes;phylum;Bacilli;class;Bacillales;order;Bacillales_incertae_sedis;family;Caldalkalibacillus;genus	86.97	2.00E-121	436
1764	0	0	1	0	2	0	0	1	1	Chloroflexi	TK7	mle-48	S000434346 Desulfotomaculum thermobenzoicum (T); TPO Lineage=Root;rootrank;Bacteria;domain;Firmicutes;phylum;Clostridia;class;Clostridiales;order;Peptococcaceae 2;family;Desulfotomaculum;genus	85.56	7.00E-106	385
799	0	0	0	0	0	1	1	0	0	Chloroflexi	TK7	mle-48	S000626776 Geoalkalibacter ferrihydriticus (T); Z-0531 Lineage=Root;rootrank;Bacteria;domain;"Proteobacteria";phylum;Deltaproteobacteria;class;Desulfuromonadales;order;Geobacteraceae;family;Geoalkalibacter;genus	84.91	2.00E-101	370
461	0	0	0	0	1	0	0	0	0	Chloroflexi	TK7	mle-48	S000653177 Caldalkalibacillus uzonensis (T); JW/WZ-YB58 Lineage=Root;rootrank;Bacteria;domain;Firmicutes;phylum;Bacilli;class;Bacillales;order;Bacillales_incertae_sedis;family;Caldalkalibacillus;genus	86.58	3.00E-119	429
1312	0	0	0	2	2	1	0	0	1	Chloroflexi	TK7	mle-48	S000752077 Moorella perchloratireducens; An10 Lineage=Root;rootrank;Bacteria;domain;Firmicutes;phylum;Clostridia;class;Thermoanaerobacterales;order;Thermoanaerobacteraceae;family;Moorella;genus	87.13	9.00E-115	414
3255	1	2	0	0	0	0	0	0	0	Chloroflexi	TK7	mle-48	S000870713 Calditerricola yamamurae (T); YMO722 Lineage=Root;rootrank;Bacteria;domain;Firmicutes;phylum;Bacilli;class;Bacillales;order;Bacillales_incertae_sedis;family;Calditerricola;genus	86.93	7.00E-121	435
3148	0	0	0	0	0	0	1	0	0	Chloroflexi	TK7	mle-48	S000870713 Calditerricola yamamurae (T); YMO722 Lineage=Root;rootrank;Bacteria;domain;Firmicutes;phylum;Bacilli;class;Bacillales;order;Bacillales_incertae_sedis;family;Calditerricola;genus	87.23	2.00E-115	416
2525	4	9	3	1	4	1	3	3	2	Chloroflexi	TK7	mle-48	S001873684 Bacillus agaradhaerens; 2007 Lineage=Root;rootrank;Bacteria;domain;Firmicutes;phylum;Bacilli;class;Bacillales;order;Sporolactobacillaceae;family;Sporolactobacillaceae_incertae_sedis;genus	85.56	7.00E-106	385
280	0	0	1	2	0	0	0	0	1	Chloroflexi	TK7	mle-48	S002166665 Chungangia koreensis; CAU 9163 Lineage=Root;rootrank;Bacteria;domain;Firmicutes;phylum;Bacilli;class;Bacillales;order;unclassified_Bacillales;	85.86	1.00E-113	411
2832	0	0	0	0	0	0	0	2	0	Chloroflexi	TK7	mle-48	S002442168 Oceanobacillus profundus; CMS193 Lineage=Root;rootrank;Bacteria;domain;Firmicutes;phylum;Bacilli;class;Bacillales;order;Planococcaceae;family;Jeotgalibacillus;genus	86.08	3.00E-115	416
1717	0	0	0	1	0	0	0	0	0	Chloroflexi	TK7	mle-48	S003286846 Moorella thermoacetica; Y73 Lineage=Root;rootrank;Bacteria;domain;Firmicutes;phylum;Clostridia;class;Thermoanaerobacterales;order;Thermoanaerobacteraceae;family;Moorella;genus	87.67	3.00E-119	429
932	0	0	1	7	9	9	6	3	16	Chloroflexi	TK7	S085	S000018082 Gram-positive bacteria SOGA31 Lineage=Root;rootrank;Bacteria;domain;unclassified_Bacteria;	87.28	1.00E-123	444
2986	0	0	8	76	42	32	53	76	105	Chloroflexi	TK7	S085	S000022386 Thermoactinomyces sanguinis; CCUG 38657T Lineage=Root;rootrank;Bacteria;domain;Firmicutes;phylum;Bacilli;class;Bacillales;order;Thermoactinomycetaceae 1;family;unclassified_Thermoactinomycetaceae 1;	85.41	7.00E-106	385
3223	0	0	1	14	23	2	3	4	4	Chloroflexi	TK7	S085	S000390568 Thermolithobacter ferrireducens; JW/KA-2; KA2b Lineage=Root;rootrank;Bacteria;domain;Firmicutes;phylum;Thermolithobacteria;class;Thermolithobacterales;order;Thermolithobacteraceae;family;Thermolithobacter;genus	86.49	2.00E-111	403
1672	0	0	3	43	44	34	20	6	37	Chloroflexi	TK7	S085	S000438120 Rubrobacter radiotolerans (T); JCM 2153-T Lineage=Root;rootrank;Bacteria;domain;"Actinobacteria";phylum;Actinobacteria;class;Rubrobacteridae;subclass;Rubrobacterales;order;"Rubrobacterineae";suborder;Rubrobacteraceae;family;Rubrobacter;genus	86.9	2.00E-115	416
3008	0	0	0	1	1	0	2	2	2	Chloroflexi	TK7	S085	S000438120 Rubrobacter radiotolerans (T); JCM 2153-T Lineage=Root;rootrank;Bacteria;domain;"Actinobacteria";phylum;Actinobacteria;class;Rubrobacteridae;subclass;Rubrobacterales;order;"Rubrobacterineae";suborder;Rubrobacteraceae;family;Rubrobacter;genus	86.93	2.00E-115	416

494	0	0	1	13	15	62	75	9	17	Chloroflexi	TK7	S085			S000438509 Moorella glycerini (T); YS6 Lineage=Root;rootrank;Bacteria;domain;Firmicutes;phylum;Clostridia;class;Thermoanaerobacterales;order;Thermoanaerobacteraceae;family;Moorella;genus	85.9	1.00E-107	390
2991	0	0	2	28	15	17	19	10	12	Chloroflexi	TK7	S085			S000438509 Moorella glycerini (T); YS6 Lineage=Root;rootrank;Bacteria;domain;Firmicutes;phylum;Clostridia;class;Thermoanaerobacterales;order;Thermoanaerobacteraceae;family;Moorella;genus	86.17	3.00E-109	396
2138	1	0	10	19	4	16	16	12	13	Chloroflexi	TK7	S085			S000438509 Moorella glycerini (T); YS6 Lineage=Root;rootrank;Bacteria;domain;Firmicutes;phylum;Clostridia;class;Thermoanaerobacterales;order;Thermoanaerobacteraceae;family;Moorella;genus	86.4	7.00E-111	401
2298	0	0	0	1	2	1	0	0	1	Chloroflexi	TK7	S085			S000438509 Moorella glycerini (T); YS6 Lineage=Root;rootrank;Bacteria;domain;Firmicutes;phylum;Clostridia;class;Thermoanaerobacterales;order;Thermoanaerobacteraceae;family;Moorella;genus	87.17	2.00E-115	416
2712	0	0	0	1	0	0	0	0	0	Chloroflexi	TK7	S085			S000438509 Moorella glycerini (T); YS6 Lineage=Root;rootrank;Bacteria;domain;Firmicutes;phylum;Clostridia;class;Thermoanaerobacterales;order;Thermoanaerobacteraceae;family;Moorella;genus	85.53	2.00E-111	403
2806	0	0	1	1	2	1	1	0	1	Chloroflexi	TK7	S085			S000841843 Rubrobacter taiwanensis (T); LS-293 Lineage=Root;rootrank;Bacteria;domain;"Actinobacteria";phylum;Actinobacteria;class;Rubrobacteridae;subclass;Rubrobacterales;order;"Rubrobacterineae";suborder;Rubrobacteraceae;family;Rubrobacter;genus	87.4	1.00E-118	427
2875	0	0	0	0	1	0	0	0	0	Chloroflexi	TK7	S085			S000841843 Rubrobacter taiwanensis (T); LS-293 Lineage=Root;rootrank;Bacteria;domain;"Actinobacteria";phylum;Actinobacteria;class;Rubrobacteridae;subclass;Rubrobacterales;order;"Rubrobacterineae";suborder;Rubrobacteraceae;family;Rubrobacter;genus	86.1	2.00E-110	399
646	0	0	5	6	6	4	7	3	9	Chloroflexi	TK7	S085			S000858630 Pelotomaculum thermopropionicum SI Lineage=Root;rootrank;Bacteria;domain;Firmicutes;phylum;Clostridia;class;Clostridiales;order;Peptococcaceae 2;family;Pelotomaculum;genus	86.45	2.00E-111	403
2498	0	1	1	7	9	5	3	1	3	Chloroflexi	TK7	S085			S000870713 Calditerricola yamamurae (T); YMO722 Lineage=Root;rootrank;Bacteria;domain;Firmicutes;phylum;Bacilli;class;Bacillales;order;Bacillales_incertae_sedis;family;Calditerricola;genus	86.29	2.00E-110	399
2290	0	0	0	0	0	0	1	0	0	Chloroflexi	TK7	S085			S002231791 Enterococcus hirae; CAIM 1552 Lineage=Root;rootrank;Bacteria;domain;Firmicutes;phylum;Bacilli;class;Lactobacillales;order;Enterococcaceae;family;Enterococcus;genus	85.64	2.00E-105	383
1045	0	0	19	47	17	20	30	19	39	Chloroflexi	TK7	S085			S002290246 Dehalogenimonas lykanthroporepellens BL-DC-9 Lineage=Root;rootrank;Bacteria;domain;"Chloroflexi";phylum;"Dehalococcoidetes";class;Dehalogenimonas;genus	85.52	5.00E-107	388
2321	0	0	0	0	0	0	2	10	91	Chloroflexi	TK7	S085			S002350110 Thermaerobacter marianensis DSM 12885 Lineage=Root;rootrank;Bacteria;domain;Firmicutes;phylum;Clostridia;class;Clostridiales;order;Clostridiales_Incertae Sedis XVII;family;Thermaerobacter;genus	87.87	7.00E-121	435
2359	0	0	0	0	3	13	2	0	1	Chloroflexi	TK7	S085			S002959066 Sediminibacillus halophilus; SL35 Lineage=Root;rootrank;Bacteria;domain;Firmicutes;phylum;Bacilli;class;Bacillales;order;Bacillaceae 2;family;Sediminibacillus;genus	85.28	7.00E-111	401
1409	8	10	53	36	46	58	98	41	49	Chloroflexi	TK7	S085			S003259712 Syntrophothermus lipocalidus DSM 12680; TGB-C1 Lineage=Root;rootrank;Bacteria;domain;Firmicutes;phylum;Clostridia;class;Clostridiales;order;Syntrophomonadaceae;family;Syntrophothermus;genus	87.17	7.00E-116	418
2343	0	0	0	5	2	0	0	1	7	Chloroflexi	TK7	S085			S003259712 Syntrophothermus lipocalidus DSM 12680; TGB-C1 Lineage=Root;rootrank;Bacteria;domain;Firmicutes;phylum;Clostridia;class;Clostridiales;order;Syntrophomonadaceae;family;Syntrophothermus;genus	87.13	7.00E-116	418
3248	0	0	0	2	3	3	0	1	1	Chloroflexi	TK7	S085			S003259712 Syntrophothermus lipocalidus DSM 12680; TGB-C1 Lineage=Root;rootrank;Bacteria;domain;Firmicutes;phylum;Clostridia;class;Clostridiales;order;Syntrophomonadaceae;family;Syntrophothermus;genus	86.8	7.00E-121	435
70	0	0	0	0	2	0	0	0	0	Chloroflexi	TK7	S085			S003259712 Syntrophothermus lipocalidus DSM 12680; TGB-C1 Lineage=Root;rootrank;Bacteria;domain;Firmicutes;phylum;Clostridia;class;Clostridiales;order;Syntrophomonadaceae;family;Syntrophothermus;genus	86.7	1.00E-112	407
519	0	0	0	1	0	0	0	0	0	Chloroflexi	TK7	S085			S003259712 Syntrophothermus lipocalidus DSM 12680; TGB-C1 Lineage=Root;rootrank;Bacteria;domain;Firmicutes;phylum;Clostridia;class;Clostridiales;order;Syntrophomonadaceae;family;Syntrophothermus;genus	86.15	3.00E-115	416
1122	0	0	3	0	7	0	0	0	1	Chloroflexi	TK7	S085			S003709934 Calderihabitans maritimus; KKC1 Lineage=Root;rootrank;Bacteria;domain;Firmicutes;phylum;Clostridia;class;Thermoanaerobacterales;order;Thermoanaerobacteraceae;family;unclassified_Thermoanaerobacteraceae;	84.3	3.00E-104	379
886	0	0	1	0	3	0	0	0	0	Chloroflexi	TK7	S085			S003709934 Calderihabitans maritimus; KKC1 Lineage=Root;rootrank;Bacteria;domain;Firmicutes;phylum;Clostridia;class;Thermoanaerobacterales;order;Thermoanaerobacteraceae;family;unclassified_Thermoanaerobacteraceae;	87.3	7.00E-116	418
1333	0	0	0	0	0	0	0	4	1	Chloroflexi	TK7	S085			S003709934 Calderihabitans maritimus; KKC1 Lineage=Root;rootrank;Bacteria;domain;Firmicutes;phylum;Clostridia;class;Thermoanaerobacterales;order;Thermoanaerobacteraceae;family;unclassified_Thermoanaerobacteraceae;	87.2	7.00E-116	418
1948	0	0	0	1	0	0	0	0	0	Chloroflexi	TK7	S085			S003709934 Calderihabitans maritimus; KKC1 Lineage=Root;rootrank;Bacteria;domain;Firmicutes;phylum;Clostridia;class;Thermoanaerobacterales;order;Thermoanaerobacteraceae;family;unclassified_Thermoanaerobacteraceae;	87.2	2.00E-116	420
3189	0	0	0	1	0	0	0	0	0	Chloroflexi	TK7	S085			S000858632 Pelotomaculum thermopropionicum SI Lineage=Root;rootrank;Bacteria;domain;Firmicutes;phylum;Clostridia;class;Clostridiales;order;Peptococcaceae 2;family;Pelotomaculum;genus	86.52	2.00E-111	403
54	0	0	0	0	1	0	0	0	0	Chloroflexi	TK7				S000013381 Ammoniphilus oxalivorans (T); RAOx-FS Lineage=Root;rootrank;Bacteria;domain;Firmicutes;phylum;Bacilli;class;Bacillales;order;Paenibacillaceae 2;family;Ammoniphilus;genus	87.13	2.00E-115	416
2072	0	0	0	1	0	0	0	0	0	Chloroflexi	TK7				S000018082 Gram-positive bacteria SOGA31 Lineage=Root;rootrank;Bacteria;domain;unclassified_Bacteria;	84.22	1.00E-103	377
851	0	0	0	2	0	4	0	1	2	Chloroflexi	TK7				S000375954 Pelotomaculum terephthalicicum (T); JT Lineage=Root;rootrank;Bacteria;domain;Firmicutes;phylum;Clostridia;class;Clostridiales;order;Peptococcaceae 2;family;Pelotomaculum;genus	86.52	2.00E-111	403
394	0	0	3	2	9	14	11	0	3	Chloroflexi	TK7				S000383946 Thermanaeromonas toyohensis (T); ToBE Lineage=Root;rootrank;Bacteria;domain;Firmicutes;phylum;Clostridia;class;Thermoanaerobacterales;order;Thermoanaerobacteraceae;family;Thermanaeromonas;genus	86.1	1.00E-108	394
1027	0	0	0	3	1	1	0	0	0	Chloroflexi	TK7				S000438509 Moorella glycerini (T); YS6 Lineage=Root;rootrank;Bacteria;domain;Firmicutes;phylum;Clostridia;class;Thermoanaerobacterales;order;Thermoanaerobacteraceae;family;Moorella;genus	87.06	7.00E-116	418
1147	0	0	21	85	104	166	143	10	58	Chloroflexi	TK7				S000469501 Heliobacillus mobilis (T); DSM 6151 Lineage=Root;rootrank;Bacteria;domain;Firmicutes;phylum;Clostridia;class;Clostridiales;order;Heliobacteriaceae;family;Heliobacillus;genus	84.78	2.00E-101	370
1725	0	0	0	0	0	0	0	1	0	Chloroflexi	TK7				S000642543 Calditerricola satsumensis (T); YMO81 Lineage=Root;rootrank;Bacteria;domain;Firmicutes;phylum;Bacilli;class;Bacillales;order;Bacillales_incertae_sedis;family;Calditerricola;genus	87.94	7.00E-121	435
1652	0	0	0	0	0	0	2	0	0	Chloroflexi	TK7				S000650984 Desulfoglaeba alkanexedens (T); ALDC Lineage=Root;rootrank;Bacteria;domain;"Proteobacteria";phylum;Deltaproteobacteria;class;Syntrophobacterales;order;Syntrophobacteraceae;family;Desulfoglaeba;genus	82.99	3.00E-94	346
2863	0	0	16	12	102	25	24	5	52	Chloroflexi	TK7				S000752077 Moorella perchloratireducens; An10 Lineage=Root;rootrank;Bacteria;domain;Firmicutes;phylum;Clostridia;class;Thermoanaerobacterales;order;Thermoanaerobacteraceae;family;Moorella;genus	86.86	9.00E-115	414
1301	0	0	0	0	1	0	0	1	0	Chloroflexi	TK7				S000841843 Rubrobacter taiwanensis (T); LS-293 Lineage=Root;rootrank;Bacteria;domain;"Actinobacteria";phylum;Actinobacteria;class;Rubrobacteridae;subclass;Rubrobacterales;order;"Rubrobacterineae";suborder;Rubrobacteraceae;family;Rubrobacter;genus	86.86	2.00E-115	416
210	0	0	0	3	0	0	0	10	49	Chloroflexi	TK7				S000858630 Pelotomaculum thermopropionicum SI Lineage=Root;rootrank;Bacteria;domain;Firmicutes;phylum;Clostridia;class;Clostridiales;order;Peptococcaceae 2;family;Pelotomaculum;genus	88.08	7.00E-121	435
1052	0	0	0	0	0	1	0	0	0	Chloroflexi	TK7				S003263114 Enterococcus sulfureus; NBRC 100680 Lineage=Root;rootrank;Bacteria;domain;Firmicutes;phylum;Bacilli;class;Lactobacillales;order;Enterococcaceae;family;Enterococcus;genus	84.27	1.00E-98	361
2136	0	0	0	0	2	9	13	1	1	Chloroflexi	TK7				S003610437 Pelobacter carbinolicus (T); DSM 2380 Lineage=Root;rootrank;Bacteria;domain;"Proteobacteria";phylum;Deltaproteobacteria;class;Desulfuromonadales;order;Desulfuromonadaceae;family;Pelobacter;genus	85.25	3.00E-104	379
143	1	2	4	0	1	0	0	0	0	Chloroflexi	TK7				S003709934 Calderihabitans maritimus; KKC1 Lineage=Root;rootrank;Bacteria;domain;Firmicutes;phylum;Clostridia;class;Thermoanaerobacterales;order;Thermoanaerobacteraceae;family;unclassified_Thermoanaerobacteraceae;	86.63	1.00E-112	407
2041	0	0	0	3	0	0	0	0	0	Chloroflexi	TK7				S003709934 Calderihabitans maritimus; KKC1 Lineage=Root;rootrank;Bacteria;domain;Firmicutes;phylum;Clostridia;class;Thermoanaerobacterales;order;Thermoanaerobacteraceae;family;unclassified_Thermoanaerobacteraceae;	84.45	3.00E-99	363
3038	0	0	0	1	0	0	0	0	0	Chloroflexi	TK7				S003709934 Calderihabitans maritimus; KKC1 Lineage=Root;rootrank;Bacteria;domain;Firmicutes;phylum;Clostridia;class;Thermoanaerobacterales;order;Thermoanaerobacteraceae;family;unclassified_Thermoanaerobacteraceae;	87.53	7.00E-126	451
301	0	0	0	2	0	2	0	0	6	Chloroflexi					S000000782 Ammoniphilus oxalaticus (T); RAOx1 Lineage=Root;rootrank;Bacteria;domain;Firmicutes;phylum;Bacilli;class;Bacillales;order;Paenibacillaceae 2;family;Ammoniphilus;genus	86.29	7.00E-111	401
776	0	0	0	1	1	1	0	1	0	Chloroflexi					S000000782 Ammoniphilus oxalaticus (T); RAOx1 Lineage=Root;rootrank;Bacteria;domain;Firmicutes;phylum;Bacilli;class;Bacillales;order;Paenibacillaceae 2;family;Ammoniphilus;genus	85.25	1.00E-103	377
88	0	0	0	1	0	0	0	0	0	Chloroflexi					S000125079 Levilinea saccharolytica (T); KIBI-1 Lineage=Root;rootrank;Bacteria;domain;"Chloroflexi";phylum;Anaerolineae;class;Anaerolineales;order;Anaerolineaceae;family;Levilinea;genus	80.32	5.00E-72	272
1723	0	0	0	2	3	1	2	1	3	Chloroflexi					S000128481 Thermoleophilum album; type strain: ATCC 35266 Lineage=Root;rootrank;Bacteria;domain;"Actinobacteria";phylum;Actinobacteria;class;Rubrobacteridae;subclass;Thermoleophilales;order;Thermoleophilaceae;family;Thermoleophilum;genus	86.99	2.00E-122	440
2979	0	0	3	22	10	0	0	15	15	Chloroflexi					S000130885 Thermoleophilum album (T); type strain: ATCC 35263 Lineage=Root;rootrank;Bacteria;domain;"Actinobacteria";phylum;Actinobacteria;class;Rubrobacteridae;subclass;Thermoleophilales;order;Thermoleophilaceae;family;Thermoleophilum;genus	84.84	5.00E-102	372
1511	0	0	0	1	3	0	0	0	0	Chloroflexi					S000130885 Thermoleophilum album (T); type strain: ATCC 35263 Lineage=Root;rootrank;Bacteria;domain;"Actinobacteria";phylum;Actinobacteria;class;Rubrobacteridae;subclass;Thermoleophilales;order;Thermoleophilaceae;family;Thermoleophilum;genus	84.27	3.00E-99	363
2418	0	0	0	1	0	0	0	0	1	Chloroflexi					S000375954 Pelotomaculum terephthalicicum (T); JT Lineage=Root;rootrank;Bacteria;domain;Firmicutes;phylum;Clostridia;class;Clostridiales;order;Peptococcaceae 2;family;Pelotomaculum;genus	80.79	2.00E-76	287
546	0	0	0	2	0	0	0	0	0	Chloroflexi					S000383099 Pelotomaculum thermopropionicum (T); SI Lineage=Root;rootrank;Bacteria;domain;Firmicutes;phylum;Clostridia;class;Clostridiales;order;Peptococcaceae 2;family;Pelotomaculum;genus	84.82	9.00E-100	364
286	0	0	0	0	1	0	0	0	0	Chloroflexi					S000383099 Pelotomaculum thermopropionicum (T); SI Lineage=Root;rootrank;Bacteria;domain;Firmicutes;phylum;Clostridia;class;Clostridiales;order;Peptococcaceae 2;family;Pelotomaculum;genus	87.47	4.00E-118	425
2997	0	0	1	3	2	0	0	0	0	Chloroflexi					S000412192 Streptacidiphilus luteoalbus; IS096 Lineage=Root;rootrank;Bacteria;domain;"Actinobacteria";phylum;Actinobacteria;class;Actinobacteridae;subclass;Actinomycetales;order;Streptomycineae;suborder;Streptomycetaceae;family;Streptacidiphilus;genus	83.96	7.00E-96	351
2555	0	0	0	2	0	0	0	0	0	Chloroflexi					S000434673 Thermovenabulum ferriorganovorum (T); Z-9801 Lineage=Root;rootrank;Bacteria;domain;Firmicutes;phylum;Clostridia;class;Clostridiales;order;Clostridiales_Incertae Sedis III;family;Thermovenabulum;genus	80.7	2.00E-81	303
2731	0	0	0	1	0	3	0	0	0	Chloroflexi					S000436149 Desulfuromonas acetoxidans Lineage=Root;rootrank;Bacteria;domain;"Proteobacteria";phylum;Deltaproteobacteria;class;Desulfuromonadales;order;Desulfuromonadaceae;family;Desulfuromonas;genus	85.49	4.00E-113	409
1752	0	0	0	1	0	0	0	0	0	Chloroflexi					S000437489 Desulfotomaculum thermocisternum (T); ST90 Lineage=Root;rootrank;Bacteria;domain;Firmicutes;phylum;Clostridia;class;Clostridiales;order;Peptococcaceae 2;family;Desulfotomaculum;genus	85.75	1.00E-113	411
2216	0	0	1	3	10	3	5	1	9	Chloroflexi					S000438509 Moorella glycerini (T); YS6 Lineage=Root;rootrank;Bacteria;domain;Firmicutes;phylum;Clostridia;class;Thermoanaerobacterales;order;Thermoanaerobacteraceae;family;Moorella;genus	84.56	7.00E-106	385
1108	0	0	0	0	0	0	0	0	1	Chloroflexi					S000469501 Heliobacillus mobilis (T); DSM 6151 Lineage=Root;rootrank;Bacteria;domain;Firmicutes;phylum;Clostridia;class;Clostridiales;order;Heliobacteriaceae;family;Heliobacillus;genus	84.77	2.00E-107	390
2251	0	0	0	0	1	0	0	0	0	Chloroflexi					S000514473 Saccharofermentans acetigenes (T); P6 Lineage=Root;rootrank;Bacteria;domain;Firmicutes;phylum;Clostridia;class;Clostridiales;order;Ruminococcaceae;family;Saccharofermentans;genus	86.13	3.00E-109	396
1603	0	0	0	0	3	1	0	0	3	Chloroflexi					S000584753 Planifilum yunnanense (T); LA5 Lineage=Root;rootrank;Bacteria;domain;Firmicutes;phylum;Bacilli;class;Bacillales;order;Thermoactinomycetaceae 2;family;Planifilum;genus	85.71	4.00E-113	409
2523	0	1	18	102	146	1	7	10	97	Chloroflexi					S000622966 Bellilinea caldifistulae (T); GOMI-1 Lineage=Root;rootrank;Bacteria;domain;"Chloroflexi";phylum;Anaerolineae;class;Anaerolineales;order;Anaerolineaceae;family;Bellilinea;genus	80.27	3.00E-70	267
1211	0	0	0	6	1	9	8	2	2	Chloroflexi					S000622967 Longilinea arvoryzae (T); KOME-1 Lineage=Root;rootrank;Bacteria;domain;"Chloroflexi";phylum;Anaerolineae;class;Anaerolineales;order;Anaerolineaceae;family;Longilinea;genus	78.78	5.00E-62	239
1871	0	0	3	2	1	0	2	0	1	Chloroflexi					S000626776 Geoalkalibacter ferrihydriticus (T); Z-0531 Lineage=Root;rootrank;Bacteria;domain;"Proteobacteria";phylum;Deltaproteobacteria;class;Desulfuromonadales;order;Geobacteraceae;family;Geoalkalibacter;genus	85.18	3.00E-104	379
2429	0	0	0	4	1	0	2	0	1	Chloroflexi					S000626776 Geoalkalibacter ferrihydriticus (T); Z-0531 Lineage=Root;rootrank;Bacteria;domain;"Proteobacteria";phylum;Deltaproteobacteria;class;Desulfuromonadales;order;Geobacteraceae;family;Geoalkalibacter;genus	86.02	4.00E-108	392
1852	0	0	0	0	1	0	0	0	0	Chloroflexi					S000626776 Geoalkalibacter ferrihydriticus (T); Z-0531 Lineage=Root;rootrank;Bacteria;domain;"Proteobacteria";phylum;Deltaproteobacteria;class;Desulfuromonadales;order;Geobacteraceae;family;Geoalkalibacter;genus	84.65	3.00E-105	383
1769	0	0	0	0	2	0	0	0	0	Chloroflexi					S000858360 Thermosulfidibacter takaii (T); ABI70S6 (= JCM13301, = DSM 17441) Lineage=Root;rootrank;Bacteria;domain;"Aquificae";phylum;Aquificae;class;Aquificales;order;Aquificales_incertae_sedis;family;Thermosulfidibacter;genus	81.84	6.00E-87	322
2395	0	0	0	7	5	2	1	0	4	Chloroflexi					S000870713 Calditerricola yamamurae (T); YMO722 Lineage=Root;rootrank;Bacteria;domain;Firmicutes;phylum;Bacilli;class;Bacillales;order;Bacillales_incertae_sedis;family;Calditerricola;genus	84.76	2.00E-100	366
1328	0	0	0	1	0	0	0	0	0	Chloroflexi					S001152076 Thermaerobacter composti (T); Ni80 Lineage=Root;rootrank;Bacteria;domain;Firmicutes;phylum;Clostridia;class;Clostridiales;order;Clostridiales_Incertae Sedis XVII;family;Thermaerobacter;genus	83.8	3.00E-100	366
2442	0	0	0	0	0	0	0	0	1	Chloroflexi					S001152076 Thermaerobacter composti (T); Ni80 Lineage=Root;rootrank;Bacteria;domain;Firmicutes;phylum;Clostridia;class;Clostridiales;order;Clostridiales_Incertae Sedis XVII;family;Thermaerobacter;genus	86.92	3.00E-120	433
391	0	0	0	0	0	1	1	0	0	Chloroflexi					S001240760 Dehalogenimonas lykanthroporepellens (T); BL-DC-9 Lineage=Root;rootrank;Bacteria;domain;"Chloroflexi";phylum;"Dehalococcoidetes";class;Dehalogenimonas;genus	85.82	1.00E-113	411
925	0	0	1	0	0	0	0	0	0	Chloroflexi					S001587450 Ktedonobacter racemifer; DSM 44963 Lineage=Root;rootrank;Bacteria;domain;"Chloroflexi";phylum;Ktedonobacteria;class;Ktedonobacterales;order;Ktedonobacteraceae;family;Ktedonobacter;genus	87.63	2.00E-126	453
2308	0	0	0	0	1	0	0	0	0	Chloroflexi					S001933336 Candidatus Methylomirabilis oxyfera Lineage=Root;rootrank;Bacteria;domain;unclassified_Bacteria;	86.65	3.00E-119	429
94	0	0	3	2	3	4	3	2	5	Chloroflexi					S002289810 Sphaerobacter thermophilus DSM 20745 Lineage=Root;rootrank;Bacteria;domain;"Chloroflexi";phylum;Thermomicrobia;class;Sphaerobacteridae;subclass;Sphaerobacterales;order;"Sphaerobacterineae";suborder;Sphaerobacteraceae;family;Sphaerobacter;genus	82.99	9.00E-95	348
2453	0	0	0	0	0	2	0	0	0	Chloroflexi					S002289810 Sphaerobacter thermophilus DSM 20745 Lineage=Root;rootrank;Bacteria;domain;"Chloroflexi";phylum;Thermomicrobia;class;Sphaerobacteridae;subclass;Sphaerobacterales;order;"Sphaerobacterineae";suborder;Sphaerobacteraceae;family;Sphaerobacter;genus	83.38	7.00E-91	335
1590	0	0	0	0	1	0	0	0	0	Chloroflexi					S002289810 Sphaerobacter thermophilus DSM 20745 Lineage=Root;rootrank;Bacteria;domain;"Chloroflexi";phylum;Thermomicrobia;class;Sphaerobacteridae;subclass;Sphaerobacterales;order;"Sphaerobacterineae";suborder;Sphaerobacteraceae;family;Sphaerobacter;genus	85.2	3.00E-110	399
2895	0	0	0	0	1	0	0	0	0	Chloroflexi					S002289810 Sphaerobacter thermophilus DSM 20745 Lineage=Root;rootrank;Bacteria;domain;"Chloroflexi";phylum;Thermomicrobia;class;Sphaerobacteridae;subclass;Sphaerobacterales;order;"Sphaerobacterineae";suborder;Sphaerobacteraceae;family;Sphaerobacter;genus	83.65	9.00E-95	348
457	10	16	0	0	1	2	4	3	0	Chloroflexi					S002350110 Thermaerobacter marianensis DSM 12885 Lineage=Root;rootrank;Bacteria;domain;Firmicutes;phylum;Clostridia;class;Clostridiales;order;Clostridiales_Incertae Sedis XVII;family;Thermaerobacter;genus	87.63	3.00E-119	429
943	0	0	1	0	0	1	0	0	0	Chloroflexi					S002350110 Thermaerobacter marianensis DSM 12885 Lineage=Root;rootrank;Bacteria;domain;Firmicutes;phylum;Clostridia;class;Clostridiales;order;Clostridiales_Incertae Sedis XVII;family;Thermaerobacter;genus	85.44	7.00E-106	385
2554	0	0	0	0	2	0	0	0	0	Chloroflexi					S002350110 Thermaerobacter marianensis DSM 12885 Lineage=Root;rootrank;Bacteria;domain;Firmicutes;phylum;Clostridia;class;Clostridiales;order;Clostridiales_Incertae Sedis XVII;family;Thermaerobacter;genus	81.63	3.00E-85	316
1497	0	0	0	1	0	0	0	0	0	Chloroflexi					S002350110 Thermaerobacter marianensis DSM 12885 Lineage=Root;rootrank;Bacteria;domain;Firmicutes;phylum;Clostridia;class;Clostridiales;order;Clostridiales_Incertae Sedis XVII;family;Thermaerobacter;genus	81.89	1.00E-82	307
2169	0	0	1	0	1	0	1	0	0	Chloroflexi					S002959010 Lysinibacillus xylanilyticus; SL1_3A Lineage=Root;rootrank;Bacteria;domain;Firmicutes;phylum;Bacilli;class;Bacillales;order;Planococcaceae;family;Lysinibacillus;genus	82.74	3.00E-94	346
2333	0	0	1	0	1	1	0	0	0	Chloroflexi					S003259708 Thermomicrobium roseum; P-2 Lineage=Root;rootrank;Bacteria;domain;"Chloroflexi";phylum;Thermomicrobia;class;Thermomicrobiales;order;Thermomicrobiaceae;family;Thermomicrobium;genus	84.22	1.00E-103	377
2550	0	0	0	0	0	0	0	0	1	Chloroflexi					S003259708 Thermomicrobium roseum; P-2 Lineage=Root;rootrank;Bacteria;domain;"Chloroflexi";phylum;Thermomicrobia;class;Thermomicrobiales;order;Thermomicrobiaceae;family;Thermomicrobium;genus	84.52	7.00E-106	385
1884	0	0	0	1	0	2	1	1	0	Chloroflexi					S003264045 Mechercharimyces mesophilus; NBRC 104191 Lineage=Root;rootrank;Bacteria;domain;Firmicutes;phylum;Bacilli;class;Bacillales;order;Thermoactinomycetaceae 2;family;Mechercharimyces;genus	86.86	1.00E-113	411
2653	0	0	0	1	1	2	1	1	1	Chloroflexi					S003291140 Litorilinea aerophila; PRI-4131 Lineage=Root;rootrank;Bacteria;domain;"Chloroflexi";phylum;Caldilineae;class;Caldilineales;order;Caldilineaceae;family;Caldilinea;genus	87.28	4.00E-123	442
297	0	0	0	4	0	0	0	0	0	Chloroflexi					S003610437 Pelobacter carbinolicus (T); DSM 2380 Lineage=Root;rootrank;Bacteria;domain;"Proteobacteria";phylum;Deltaproteobacteria;class;Desulfuromonadales;order;Desulfuromonadaceae;family;Pelobacter;genus	84.76	9.00E-100	364
126	0	0	1	0	0	0	2	1	0	Chloroflexi					S003709934 Calderihabitans maritimus; KKC1 Lineage=Root;rootrank;Bacteria;domain;Firmicutes;phylum;Clostridia;class;Thermoanaerobacterales;order;Thermoanaerobacteraceae;family;unclassified_Thermoanaerobacteraceae;	86.62	3.00E-119	429
2660	0	0	0	0	0	0	0	1	1	Chloroflexi					S003709934 Calderihabitans maritimus; KKC1 Lineage=Root;rootrank;Bacteria;domain;Firmicutes;phylum;Clostridia;class;Thermoanaerobacterales;order;Thermoanaerobacteraceae;family;unclassified_Thermoanaerobacteraceae;	86.6	1.00E-112	407
2018	0	0	0	0	1	0	0	0	0	Chloroflexi					S003709934 Calderihabitans maritimus; KKC1 Lineage=Root;rootrank;Bacteria;domain;Firmicutes;phylum;Clostridia;class;Thermoanaerobacterales;order;Thermoanaerobacteraceae;family;unclassified_Thermoanaerobacteraceae;	85	5.00E-102	372
327	0	1	0	0	0	0	0	0	0	Crenarchaeota	C2	pGrfC26			S000995436 Octopus Spring nitrifying crenarchaeote OS70 Lineage=Root;rootrank;Archaea;domain;"Crenarchaeota";phylum;Thermoprotei;class;unclassified_Thermoprotei;	84.68	7.00E-101	368
918	0	0	0	0	0	0	0	0	1	Crenarchaeota	Thaumarchaeota	Cenarchaeales	Cenarchaeaceae		S003206769 uncltured Marine Benthic group C euryarchaeote; DH131A20 Lineage=Root;rootrank;Archaea;domain;unclassified_Archaea;	94.96	5.00E-167	588
1897	0	6	0	4	3	5	6	8	19	Crenarchaeota	Thaumarchaeota	Nitrososphaerales	Nitrososphaeraceae	CandidatusNitrososphaera	S002917523 Candidatus Nitrosotalea devanaterra; Nd1 Lineage=Root;rootrank;Archaea;domain;"Crenarchaeota";phylum;Thermoprotei;class;unclassified_Thermoprotei;	90.4	4.00E-138	492
1946	0	0	3	9	8	7	0	4	11	Crenarchaeota	Thaumarchaeota	Nitrososphaerales	Nitrososphaeraceae	CandidatusNitrososphaera	S002917523 Candidatus Nitrosotalea devanaterra; Nd1 Lineage=Root;rootrank;Archaea;domain;"Crenarchaeota";phylum;Thermoprotei;class;unclassified_Thermoprotei;	90.72	2.00E-140	499
3119	0	0	0	5	1	0	1	0	0	Crenarchaeota	Thaumarchaeota	Nitrososphaerales	Nitrososphaeraceae	CandidatusNitrososphaera	S002917523 Candidatus Nitrosotalea devanaterra; Nd1 Lineage=Root;rootrank;Archaea;domain;"Crenarchaeota";phylum;Thermoprotei;class;unclassified_Thermoprotei;	88.89	1.00E-128	460
1207	7	4	9	12	17	12	34	4	14	Crenarchaeota	Thaumarchaeota	Nitrososphaerales	Nitrososphaeraceae	CandidatusNitrososphaera	S003206766 uncltured Marine Benthic group A euryarchaeote; DH133A34 Lineage=Root;rootrank;Archaea;domain;"Crenarchaeota";phylum;Thermoprotei;class;unclassified_Thermoprotei;	89.39	5.00E-132	472
768	0	0	0	0	0	1	0	2	0	Cyanobacteria	4C0d-2	SMD			S002167714 Vampirovibrio chlorellavorus (T); ICPB 3707 Lineage=Root;rootrank;Bacteria;domain;"Proteobacteria";phylum;Deltaproteobacteria;class;Bdellovibrionales;order;Bdellovibrionaceae;family;Vampirovibrio;genus	90.19	1.00E-133	477
422	0	0	0	1	0	0	1	0	0	Cyanobacteria	4C0d-2	SMD			S002167714 Vampirovibrio chlorellavorus (T); ICPB 3707 Lineage=Root;rootrank;Bacteria;domain;"Proteobacteria";phylum;Deltaproteobacteria;class;Bdellovibrionales;order;Bdellovibrionaceae;family;Vampirovibrio;genus	94.01	1.00E-157	556
2504	0	0	0	0	0	0	1	0	0	Cyanobacteria	4C0d-2	SMD			S002167714 Vampirovibrio chlorellavorus (T); ICPB 3707 Lineage=Root;rootrank;Bacteria;domain;"Proteobacteria";phylum;Deltaproteobacteria;class;Bdellovibrionales;order;Bdellovibrionaceae;family;Vampirovibrio;genus	88.08	2.00E-121	436
581	0	0	0	1	0	0	0	0	0	Cyanobacteria	4C0d-2	SMD			S002167714 Vampirovibrio chlorellavorus (T); ICPB 3707 Lineage=Root;rootrank;Bacteria;domain;"Proteobacteria";phylum;Deltaproteobacteria;class;Bdellovibrionales;order;Bdellovibrionaceae;family;Vampirovibrio;genus	88.86	8.00E-125	448
463	0	0	1	0	0	0	0	0	0	Cyanobacteria	Chloroplast	Chlorophyta			S000641721 Prototheca wickerhamii; 263-11 Lineage=Root;rootrank;Bacteria;domain;Cyanobacteria/Chloroplast;phylum;Chloroplast;class;Chloroplast;family;Chlorophyta;genus	80	3.00E-70	267
84	0	0	0	0	0	0	0	1	0	Cyanobacteria	Chloroplast	Streptophyta			S000641771 Juniperus virginiana Lineage=Root;rootrank;Bacteria;domain;Cyanobacteria/Chloroplast;phylum;Chloroplast;class;Chloroplast;family;Streptophyta;genus	97.59	0	638
2336	0	0	0	0	0	0	0	0	1	Cyanobacteria	Chloroplast	Streptophyta			S001020551 Vitis vinifera (T) Lineage=Root;rootrank;Bacteria;domain;Cyanobacteria/Chloroplast;phylum;Chloroplast;class;Chloroplast;family;Streptophyta;genus	99.49	0	712
2529	0	0	0	0	0	0	0	1	2	Cyanobacteria	Nostocophycideae	Nostocales	Rivulariaceae	Calothrix	S000749730 Calothrix desertica PCC 7102 Lineage=Root;rootrank;Bacteria;domain;Cyanobacteria/Chloroplast;phylum;Cyanobacteria;class;Family I;family;GpI;genus	98.98	0	701
306	0	0	0	0	0	0	0	3	0	Cyanobacteria	Oscillatoriophycideae	Chroococcales	Xenococcaceae	Chroococcidiopsis	S002408151 Pleurocapsa cf. concharum 1d-08 Lineage=Root;rootrank;Bacteria;domain;Cyanobacteria/Chloroplast;phylum;Cyanobacteria;class;unclassified_Cyanobacteria;	94.64	1.00E-163	577
1958	0	0	0	0	0	0	0	1	2	Cyanobacteria	Oscillatoriophycideae	Chroococcales	Xenococcaceae	Chroococcidiopsis	S002408151 Pleurocapsa cf. concharum 1d-08 Lineage=Root;rootrank;Bacteria;domain;Cyanobacteria/Chloroplast;phylum;Cyanobacteria;class;unclassified_Cyanobacteria;	93.88	5.00E-167	588
2466	0	0	0	0	0	0	0	2	0	Cyanobacteria	Oscillatoriophycideae	Chroococcales	Xenococcaceae	Chroococcidiopsis	S002408151 Pleurocapsa cf. concharum 1d-08 Lineage=Root;rootrank;Bacteria;domain;Cyanobacteria/Chloroplast;phylum;Cyanobacteria;class;unclassified_Cyanobacteria;	94.35	5.00E-162	571
2266	0	0	0	0	0	0	0	0	2	Cyanobacteria	Oscillatoriophycideae	Chroococcales	Xenococcaceae	Chroococcidiopsis	S002408151 Pleurocapsa cf. concharum 1d-08 Lineage=Root;rootrank;Bacteria;domain;Cyanobacteria/Chloroplast;phylum;Cyanobacteria;class;unclassified_Cyanobacteria;	93.01	1.00E-153	544
2757	0	0	0	0	0	0	0	1	0	Cyanobacteria	Oscillatoriophycideae	Chroococcales	Xenococcaceae	Chroococcidiopsis	S002408151 Pleurocapsa cf. concharum 1d-08 Lineage=Root;rootrank;Bacteria;domain;Cyanobacteria/Chloroplast;phylum;Cyanobacteria;class;unclassified_Cyanobacteria;	93.89	4.00E-168	592
1773	0	0	0	0	0	0	0	0	1	Cyanobacteria	Oscillatoriophycideae	Chroococcales	Xenococcaceae	Chroococcidiopsis	S002408151 Pleurocapsa cf. concharum 1d-08 Lineage=Root;rootrank;Bacteria;domain;Cyanobacteria/Chloroplast;phylum;Cyanobacteria;class;unclassified_Cyanobacteria;	93.57	5.00E-157	555
124	0	0	0	0	0	0	0	2	1	Cyanobacteria	S5B-MN24				S003610437 Pelobacter carbinolicus (T); DSM 2380 Lineage=Root;rootrank;Bacteria;domain;"Proteobacteria";phylum;Deltaproteobacteria;class;Desulfuromonadales;order;Desulfuromonadaceae;family;Pelobacter;genus	87.84	9.00E-120	431
133	0	0	0	0	0	0	0	0	3	Cyanobacteria	Synechococcophycideae	Pseudanabaenales	Pseudanabaenaceae	Leptolyngbya	S000768634 Leptolyngbya frigida ANT.REIDJ.1 Lineage=Root;rootrank;Bacteria;domain;Cyanobacteria/Chloroplast;phylum;Cyanobacteria;class;unclassified_Cyanobacteria;	91.3	7.00E-151	534
1936	0	0	0	0	0	0	0	1	1	Cyanobacteria	Synechococcophycideae	Pseudanabaenales	Pseudanabaenaceae	Leptolyngbya	S003619397 Leptolyngbya boryana UAM 391 Lineage=Root;rootrank;Bacteria;domain;Cyanobacteria/Chloroplast;phylum;Cyanobacteria;class;Family V;family;GpV;genus	97.58	0	638
1796	0	0	0	0	0	0	0	0	3	Cyanobacteria					S000025240 Phormidium tenue; NIES30 Lineage=Root;rootrank;Bacteria;domain;Cyanobacteria/Chloroplast;phylum;Cyanobacteria;class;unclassified_Cyanobacteria;	91.94	5.00E-147	521
109	0	0	0	0	0	0	0	1	0	Cyanobacteria					S000675056 Scenedesmus obliquus (T); UTEX 393 Lineage=Root;rootrank;Bacteria;domain;Cyanobacteria/Chloroplast;phylum;Chloroplast;class;Chloroplast;family;Chlorophyta;genus	91.41	3.00E-139	496
113	0	0	0	0	0	0	0	1	0	Cyanobacteria					S002223392 Stigonema ocellatum SAMA 35 Lineage=Root;rootrank;Bacteria;domain;Cyanobacteria/Chloroplast;phylum;Cyanobacteria;class;Family I;family;GpI;genus	93.94	1.00E-153	544
354	0	0	0	0	0	0	0	0	1	DHVE3					S003206766 uncltured Marine Benthic group A euryarchaeote; DH133A34 Lineage=Root;rootrank;Archaea;domain;"Crenarchaeota";phylum;Thermoprotei;class;unclassified_Thermoprotei;	80.36	1.00E-39	165
2079	0	0	0	0	1	0	0	0	0	Elusimicrobia	Elusimicrobia	Elusimicrobiales			S002289633 Elusimicrobium minutum Pei191 Lineage=Root;rootrank;Bacteria;domain;"Elusimicrobia";phylum;Elusimicrobia;class;Elusimicrobiales;order;Elusimicrobiaceae;family;Elusimicrobium;genus	84.01	2.00E-95	350
1220	0	0	0	0	1	0	0	0	0	Elusimicrobia	Elusimicrobia	FAC88			S000417550 Acidobacteriaceae isolate WJ7 Lineage=Root;rootrank;Bacteria;domain;"Acidobacteria";phylum;Acidobacteria_Gp1;class;Gp1;genus	83.85	3.00E-99	363
1713	0	0	0	1	0	0	0	0	0	Elusimicrobia	Elusimicrobia	FAC88			S001153278 Methylogaea oryzae JCM 16910; E10 Lineage=Root;rootrank;Bacteria;domain;"Proteobacteria";phylum;Gammaproteobacteria;class;Methylococcales;order;Methylococcaceae;family;Methylococcus;genus	83.24	2.00E-90	333
1996	0	0	0	2	2	0	0	0	0	Elusimicrobia	Elusimicrobia	FAC88			S003287268 Terriglobus roseus DSM 18391 Lineage=Root;rootrank;Bacteria;domain;"Acidobacteria";phylum;Acidobacteria_Gp1;class;Terriglobus;genus	83.6	5.00E-92	339
2248	0	0	0	0	1	0	0	0	1	Elusimicrobia	Elusimicrobia	FAC88			S003287268 Terriglobus roseus DSM 18391 Lineage=Root;rootrank;Bacteria;domain;"Acidobacteria";phylum;Acidobacteria_Gp1;class;Terriglobus;genus	84.55	1.00E-98	361
842	0	0	0	1	0	0	0	0	0	Elusimicrobia	Elusimicrobia	FAC88			S003287268 Terriglobus roseus DSM 18391 Lineage=Root;rootrank;Bacteria;domain;"Acidobacteria";phylum;Acidobacteria_Gp1;class;Terriglobus;genus	82.31	1.00E-88	327
775	0	0	0	0	0	0	0	1	0	Elusimicrobia	Elusimicrobia	FAC88			S003287268 Terriglobus roseus DSM 18391 Lineage=Root;rootrank;Bacteria;domain;"Acidobacteria";phylum;Acidobacteria_Gp1;class;Terriglobus;genus	83.47	5.00E-92	339
3193	0	0	0	1	0	0	0	0	0	Elusimicrobia	Elusimicrobia	MVP-88			S000417550 Acidobacteriaceae isolate WJ7 Lineage=Root;rootrank;Bacteria;domain;"Acidobacteria";phylum;Acidobacteria_Gp1;class;Gp1;genus	80.91	3.00E-75	283
2086	0	0	1	0	0	0	0	0	0	Elusimicrobia	Elusimicrobia	MVP-88			S001198079 Thioprofundum lithotrophicum (T); 106 Lineage=Root;rootrank;Bacteria;domain;"Proteobacteria";phylum;Gammaproteobacteria;class;Gammaproteobacteria_incertae_sedis;order;Thioprofundum;genus	81.94	1.00E-82	307
661	0	0	0	1	0	0	0	0	0	Elusimicrobia	Elusimicrobia	MVP-88			S001795340 Pseudomonas fragi; CS11RH4 Lineage=Root;rootrank;Bacteria;domain;"Proteobacteria";phylum;Gammaproteobacteria;class;Pseudomonadales;order;Pseudomonadaceae;family;Pseudomonas;genus	82.21	1.00E-83	311
2890	0	0	0	1	0	0	0	0	0	Elusimicrobia	Elusimicrobia	MVP-88			S002289633 Elusimicrobium minutum Pei191 Lineage=Root;rootrank;Bacteria;domain;"Elusimicrobia";phylum;Elusimicrobia;class;Elusimicrobiales;order;Elusimicrobiaceae;family;Elusimicrobium;genus	82.38	2.00E-86	320
1111	0	0	0	0	0	0	0	0	1	Elusimicrobia	Elusimicrobia	MVP-88			S002440613 Conexibacter arvalis; KV-962 Lineage=Root;rootrank;Bacteria;domain;"Actinobacteria";phylum;Actinobacteria;class;Rubrobacteridae;subclass;Solirubrobacterales;order;Conexibacteraceae;family;Conexibacter;genus	82.29	2.00E-85	316
1709	0	0	0	2	0	2	0	0	0	Elusimicrobia	Elusimicrobia				S000484019 Olavius crassitunicatus delta-proteobacterial endosymbiont; d3-P12-1 Lineage=Root;rootrank;Bacteria;domain;"Proteobacteria";phylum;Deltaproteobacteria;class;Desulfobacterales;order;Desulfobacteraceae;family;unclassified_Desulfobacteraceae;	83.74	1.00E-93	344

132	0	0	0	1	2	0	0	0	0	Elusimicrobia					S001153278 Methylogaea oryzae JCM 16910; E10 Lineage=Root;rootrank;Bacteria;domain;"Proteobacteria";phylum;Gammaproteobacteria;class;Methylococcales;order;Methylococcaceae;family;Methylococcus;genus	82.78	2.00E-92	340
530	0	0	0	0	0	0	0	0	1	Elusimicrobia					S001198079 Thioprofundum lithotrophicum (T); 106 Lineage=Root;rootrank;Bacteria;domain;"Proteobacteria";phylum;Gammaproteobacteria;class;Gammaproteobacteria_incertae_sedis;order;Thioprofundum;genus	83.02	3.00E-89	329
194	9	61	50	0	7	5	10	2	2	Euryarchaeota	Halobacteria	Halobacteriales	Halobacteriaceae		S001292585 Haladaptatus cibarius (T); D43 Lineage=Root;rootrank;Archaea;domain;"Euryarchaeota";phylum;Halobacteria;class;Halobacteriales;order;Halobacteriaceae;family;Haladaptatus;genus	93.35	1.00E-157	556
3171	1	0	0	0	0	0	0	0	0	Euryarchaeota	Halobacteria	Halobacteriales	Halobacteriaceae		S001573772 Halopelagius inordinatus (T); RO5-2 Lineage=Root;rootrank;Archaea;domain;"Euryarchaeota";phylum;Halobacteria;class;Halobacteriales;order;Halobacteriaceae;family;Halopelagius;genus	96.2	0	647
2048	0	1	0	0	0	0	0	0	0	Euryarchaeota	Halobacteria	Halobacteriales	Halobacteriaceae		S001573772 Halopelagius inordinatus (T); RO5-2 Lineage=Root;rootrank;Archaea;domain;"Euryarchaeota";phylum;Halobacteria;class;Halobacteriales;order;Halobacteriaceae;family;Halopelagius;genus	93.09	6.00E-156	551
652	4	19	0	0	0	0	3	0	0	Euryarchaeota	Halobacteria	Halobacteriales	Halobacteriaceae		S002032663 Halorubrum aidingense (T); 31-hong Lineage=Root;rootrank;Archaea;domain;"Euryarchaeota";phylum;Halobacteria;class;Halobacteriales;order;Halobacteriaceae;family;Halorubrum;genus	92.68	5.00E-162	571
105	0	2	2	0	0	0	0	0	0	Euryarchaeota	Halobacteria	Halobacteriales	Halobacteriaceae		S002032663 Halorubrum aidingense (T); 31-hong Lineage=Root;rootrank;Archaea;domain;"Euryarchaeota";phylum;Halobacteria;class;Halobacteriales;order;Halobacteriaceae;family;Halorubrum;genus	93.65	3.00E-159	562
1179	1	5	4	0	0	0	1	0	0	Euryarchaeota	Halobacteria	Halobacteriales	Halobacteriaceae		S002235746 Halomarina oriensis (T); KeC-11 Lineage=Root;rootrank;Archaea;domain;"Euryarchaeota";phylum;Halobacteria;class;Halobacteriales;order;Halobacteriaceae;family;Halomarina;genus	92.71	5.00E-162	571
2595	0	1	0	0	0	0	0	0	0	Euryarchaeota	Halobacteria	Halobacteriales	Halobacteriaceae		S002235746 Halomarina oriensis (T); KeC-11 Lineage=Root;rootrank;Archaea;domain;"Euryarchaeota";phylum;Halobacteria;class;Halobacteriales;order;Halobacteriaceae;family;Halomarina;genus	93.94	2.00E-170	599
384	1	8	10	0	1	0	0	0	0	Euryarchaeota	Halobacteria	Halobacteriales	Halobacteriaceae		S002283757 Halorhabdus utahensis; SFF1F051 Lineage=Root;rootrank;Archaea;domain;"Euryarchaeota";phylum;Halobacteria;class;Halobacteriales;order;Halobacteriaceae;family;unclassified_Halobacteriaceae;	93.63	1.00E-158	560
1481	0	2	0	0	0	1	0	0	0	Euryarchaeota	Halobacteria	Halobacteriales	Halobacteriaceae		S002283757 Halorhabdus utahensis; SFF1F051 Lineage=Root;rootrank;Archaea;domain;"Euryarchaeota";phylum;Halobacteria;class;Halobacteriales;order;Halobacteriaceae;family;unclassified_Halobacteriaceae;	93.18	2.00E-165	582
2346	0	0	1	0	0	0	0	0	0	Euryarchaeota	Halobacteria	Halobacteriales	Halobacteriaceae		S002283757 Halorhabdus utahensis; SFF1F051 Lineage=Root;rootrank;Archaea;domain;"Euryarchaeota";phylum;Halobacteria;class;Halobacteriales;order;Halobacteriaceae;family;unclassified_Halobacteriaceae;	93.35	1.00E-157	556
11	0	10	2	0	0	0	3	0	1	Euryarchaeota	Halobacteria	Halobacteriales	Halobacteriaceae		S003286534 Halarchaeum acidiphilum (T); MH1-52-1 Lineage=Root;rootrank;Archaea;domain;"Euryarchaeota";phylum;Halobacteria;class;Halobacteriales;order;Halobacteriaceae;family;Halarchaeum;genus	93.09	6.00E-156	551
573	0	3	0	0	0	0	0	0	0	Euryarchaeota	Halobacteria	Halobacteriales	Halobacteriaceae		S003616486 Halococcus dombrowskii; 519 Lineage=Root;rootrank;Archaea;domain;"Euryarchaeota";phylum;Halobacteria;class;Halobacteriales;order;Halobacteriaceae;family;Halococcus;genus	92.82	3.00E-154	545
281	0	0	0	0	0	1	0	0	0	Euryarchaeota	Halobacteria	Halobacteriales	Halobacteriaceae		S003619892 Halovivax ruber XH-70 Lineage=Root;rootrank;Archaea;domain;"Euryarchaeota";phylum;Halobacteria;class;Halobacteriales;order;Halobacteriaceae;family;Halovivax;genus	94.44	3.00E-164	579
3131	1	1	0	0	1	0	0	0	0	Euryarchaeota	Halobacteria	Halobacteriales	Halobacteriaceae		S003659908 Halobellus salinus; JCM 14359 Lineage=Root;rootrank;Archaea;domain;"Euryarchaeota";phylum;Halobacteria;class;Halobacteriales;order;Halobacteriaceae;family;unclassified_Halobacteriaceae;	92.15	4.00E-158	558
1489	0	1	0	0	0	0	0	0	0	Euryarchaeota	Halobacteria	Halobacteriales	Halobacteriaceae		S003659908 Halobellus salinus; JCM 14359 Lineage=Root;rootrank;Archaea;domain;"Euryarchaeota";phylum;Halobacteria;class;Halobacteriales;order;Halobacteriaceae;family;unclassified_Halobacteriaceae;	98.94	0	673
1176	1	17	1	0	0	0	0	0	0	Euryarchaeota	Halobacteria	Halobacteriales	Halobacteriaceae		S003659910 Halomicrobium zhouii; JCM 17095 Lineage=Root;rootrank;Archaea;domain;"Euryarchaeota";phylum;Halobacteria;class;Halobacteriales;order;Halobacteriaceae;family;Halomicrobium;genus	96.3	6.00E-176	617
144	0	1	4	0	0	1	2	0	0	Euryarchaeota	Halobacteria	Halobacteriales	Halobacteriaceae		S003659910 Halomicrobium zhouii; JCM 17095 Lineage=Root;rootrank;Archaea;domain;"Euryarchaeota";phylum;Halobacteria;class;Halobacteriales;order;Halobacteriaceae;family;Halomicrobium;genus	94.68	6.00E-166	584
2707	0	2	5	0	0	0	0	0	0	Euryarchaeota	Halobacteria	Halobacteriales	Halobacteriaceae		S003659910 Halomicrobium zhouii; JCM 17095 Lineage=Root;rootrank;Archaea;domain;"Euryarchaeota";phylum;Halobacteria;class;Halobacteriales;order;Halobacteriaceae;family;Halomicrobium;genus	92.02	3.00E-149	529
100	0	3	1	0	0	0	0	0	0	Euryarchaeota	Halobacteria	Halobacteriales	Halobacteriaceae		S003659915 Salinarchaeum laminariae; JCM 17267 Lineage=Root;rootrank;Archaea;domain;"Euryarchaeota";phylum;Halobacteria;class;Halobacteriales;order;Halobacteriaceae;family;unclassified_Halobacteriaceae;	98.67	0	667
371	77	205	65	1	10	13	19	6	2	Euryarchaeota	Halobacteria	Halobacteriales	Halobacteriaceae		S003711373 Natronomonas moolapensis 8.8.11 Lineage=Root;rootrank;Archaea;domain;"Euryarchaeota";phylum;Halobacteria;class;Halobacteriales;order;Halobacteriaceae;family;Natronomonas;genus	94.15	1.00E-162	573
191	12	33	3	0	1	1	1	0	0	Euryarchaeota	Halobacteria	Halobacteriales	Halobacteriaceae		S003711373 Natronomonas moolapensis 8.8.11 Lineage=Root;rootrank;Archaea;domain;"Euryarchaeota";phylum;Halobacteria;class;Halobacteriales;order;Halobacteriaceae;family;Natronomonas;genus	94.68	6.00E-166	584
1411	0	12	0	0	0	0	0	1	0	Euryarchaeota	Halobacteria	Halobacteriales	Halobacteriaceae		S003711373 Natronomonas moolapensis 8.8.11 Lineage=Root;rootrank;Archaea;domain;"Euryarchaeota";phylum;Halobacteria;class;Halobacteriales;order;Halobacteriaceae;family;Natronomonas;genus	93.35	1.00E-157	556
1708	0	0	0	0	0	2	0	0	0	Euryarchaeota	Halobacteria	Halobacteriales	Halobacteriaceae		S003716328 Halogeometricum borinquense; BAB-2230 Lineage=Root;rootrank;Archaea;domain;"Euryarchaeota";phylum;Halobacteria;class;Halobacteriales;order;Halobacteriaceae;family;Halogeometricum;genus	92.93	3.00E-154	545
1073	0	1	3	0	0	0	2	0	0	Euryarchaeota	Halobacteria	Halobacteriales	Halobacteriaceae	Halalkalicoccus	S000394150 Halalkalicoccus tibetensis (T); DS12 Lineage=Root;rootrank;Archaea;domain;"Euryarchaeota";phylum;Halobacteria;class;Halobacteriales;order;Halobacteriaceae;family;Halalkalicoccus;genus	98.94	0	673
333	0	12	5	0	0	1	4	0	0	Euryarchaeota	Halobacteria	Halobacteriales	Halobacteriaceae	Halococcus	S000626945 Halococcus morrhuae; K16 Lineage=Root;rootrank;Archaea;domain;"Euryarchaeota";phylum;Halobacteria;class;Halobacteriales;order;Halobacteriaceae;family;Halococcus;genus	97.34	0	640
1355	0	6	3	0	0	0	2	0	0	Euryarchaeota	Halobacteria	Halobacteriales	Halobacteriaceae	Halococcus	S000626945 Halococcus morrhuae; K16 Lineage=Root;rootrank;Archaea;domain;"Euryarchaeota";phylum;Halobacteria;class;Halobacteriales;order;Halobacteriaceae;family;Halococcus;genus	96.02	3.00E-174	612
2607	1	0	0	0	0	2	0	0	0	Euryarchaeota	Halobacteria	Halobacteriales	Halobacteriaceae	Halococcus	S000858365 Halococcus hamelinensis; JCM 12892 Lineage=Root;rootrank;Archaea;domain;"Euryarchaeota";phylum;Halobacteria;class;Halobacteriales;order;Halobacteriaceae;family;Halococcus;genus	96.28	6.00E-176	617
104	0	1	1	0	0	0	0	0	0	Euryarchaeota	Halobacteria	Halobacteriales	Halobacteriaceae	Natronorubrum	S003659914 Natronococcus roseus; JCM 17958 Lineage=Root;rootrank;Archaea;domain;"Euryarchaeota";phylum;Halobacteria;class;Halobacteriales;order;Halobacteriaceae;family;Haloterrigena;genus	100	0	695
795	0	1	0	0	0	0	0	0	0	Euryarchaeota	Halobacteria	Halobacteriales	Halobacteriaceae		S001328035 Halalkalicoccus jeotgali; JCM 14584 Lineage=Root;rootrank;Archaea;domain;"Euryarchaeota";phylum;Halobacteria;class;Halobacteriales;order;Halobacteriaceae;family;Halalkalicoccus;genus	95.21	3.00E-169	595
1351	0	0	0	0	0	0	0	0	1	Euryarchaeota	Halobacteria	Halobacteriales	Halobacteriaceae		S001573165 Natrialba wudunaoensis; Sua-E42 Lineage=Root;rootrank;Archaea;domain;"Euryarchaeota";phylum;Halobacteria;class;Halobacteriales;order;Halobacteriaceae;family;Natrialba;genus	96.02	3.00E-174	612
2108	0	0	4	0	0	2	1	0	1	Euryarchaeota	Halobacteria	Halobacteriales	Halobacteriaceae		S001743244 Halostagnicola alkaliphila (T); 167-74 Lineage=Root;rootrank;Archaea;domain;"Euryarchaeota";phylum;Halobacteria;class;Halobacteriales;order;Halobacteriaceae;family;Halostagnicola;genus	98.23	0	691
3234	0	7	1	0	0	0	0	0	0	Euryarchaeota	Halobacteria	Halobacteriales	Halobacteriaceae		S002236183 Halorhabdus tiamatea; JCM 14471 Lineage=Root;rootrank;Archaea;domain;"Euryarchaeota";phylum;Halobacteria;class;Halobacteriales;order;Halobacteriaceae;family;Halorhabdus;genus	93.72	1.00E-168	593
479	1	0	0	0	0	0	0	0	0	Euryarchaeota	Halobacteria	Halobacteriales	Halobacteriaceae		S002236183 Halorhabdus tiamatea; JCM 14471 Lineage=Root;rootrank;Archaea;domain;"Euryarchaeota";phylum;Halobacteria;class;Halobacteriales;order;Halobacteriaceae;family;Halorhabdus;genus	94.7	9.00E-175	614
2283	0	1	0	0	0	0	0	0	0	Euryarchaeota	Halobacteria	Halobacteriales	Halobacteriaceae		S002236185 Halorubrum kocurii; JCM 14978 Lineage=Root;rootrank;Archaea;domain;"Euryarchaeota";phylum;Halobacteria;class;Halobacteriales;order;Halobacteriaceae;family;Halorubrum;genus	92.29	6.00E-151	534
1522	0	1	0	0	0	0	0	0	0	Euryarchaeota	Halobacteria	Halobacteriales	Halobacteriaceae		S003616487 Natrialba aegyptia; 603 Lineage=Root;rootrank;Archaea;domain;"Euryarchaeota";phylum;Halobacteria;class;Halobacteriales;order;Halobacteriaceae;family;Natrialba;genus	96.55	1.00E-177	623
1595	2	5	20	0	0	1	1	1	0	Euryarchaeota	Halobacteria	Halobacteriales	Halobacteriaceae		S003659910 Halomicrobium zhouii; JCM 17095 Lineage=Root;rootrank;Archaea;domain;"Euryarchaeota";phylum;Halobacteria;class;Halobacteriales;order;Halobacteriaceae;family;Halomicrobium;genus	97.47	0	676
14	1	1	0	0	0	0	0	0	0	Euryarchaeota	Halobacteria	Halobacteriales	Halobacteriaceae		S003659910 Halomicrobium zhouii; JCM 17095 Lineage=Root;rootrank;Archaea;domain;"Euryarchaeota";phylum;Halobacteria;class;Halobacteriales;order;Halobacteriaceae;family;Halomicrobium;genus	94.46	1.00E-173	610
1577	0	2	0	0	0	0	0	0	0	Euryarchaeota	Halobacteria	Halobacteriales	Halobacteriaceae		S003659910 Halomicrobium zhouii; JCM 17095 Lineage=Root;rootrank;Archaea;domain;"Euryarchaeota";phylum;Halobacteria;class;Halobacteriales;order;Halobacteriaceae;family;Halomicrobium;genus	94.21	5.00E-172	604
166	0	0	0	0	0	0	1	0	0	Euryarchaeota	Halobacteria	Halobacteriales	Halobacteriaceae		S003659910 Halomicrobium zhouii; JCM 17095 Lineage=Root;rootrank;Archaea;domain;"Euryarchaeota";phylum;Halobacteria;class;Halobacteriales;order;Halobacteriaceae;family;Halomicrobium;genus	98.23	0	691
1431	0	0	1	0	0	0	0	0	0	Euryarchaeota	Methanomicrobia	Methanomicrobiales	Methanomicrobiaceae	Methanoculleus	S000769576 Methanoculleus thermophilus; JB-1 Lineage=Root;rootrank;Archaea;domain;"Euryarchaeota";phylum;"Methanomicrobia";class;Methanomicrobiales;order;Methanomicrobiaceae;family;Methanoculleus;genus	98.74	0	704
434	0	0	0	0	2	0	0	0	0	Euryarchaeota	Thermoplasmata	pMC2A33			S003755043 Candidatus Methanomethylophilus alvus Mx1201; patient 3 Lineage=Root;rootrank;Archaea;domain;"Euryarchaeota";phylum;unclassified_"Euryarchaeota";	81.3	4.00E-84	313
911	0	0	0	0	0	0	1	1	0	Euryarchaeota	Thermoplasmata	E2			S002989688 Methanomassiliicoccus luminyensis B10 Lineage=Root;rootrank;Archaea;domain;"Euryarchaeota";phylum;Thermoplasmata;class;Thermoplasmatales;order;Thermoplasmatales_incertae_sedis;family;Thermogymnomonas;genus	85.29	1.00E-103	377
2273	0	0	0	0	0	1	2	0	1	Euryarchaeota	Thermoplasmata				S000358055 Lineage=Root;rootrank;Archaea;domain;"Euryarchaeota";phylum;Thermoplasmata;class;Thermoplasmatales;order;unclassified_Thermoplasmatales;	84.72	9.00E-100	364
2445	0	0	0	0	1	0	0	0	0	Euryarchaeota	Thermoplasmata				S002989688 Methanomassiliicoccus luminyensis B10 Lineage=Root;rootrank;Archaea;domain;"Euryarchaeota";phylum;Thermoplasmata;class;Thermoplasmatales;order;Thermoplasmatales_incertae_sedis;family;Thermogymnomonas;genus	85.56	9.00E-105	381
789	0	0	0	2	0	0	0	0	0	Euryarchaeota					S000335961 Lineage=Root;rootrank;Archaea;domain;"Euryarchaeota";phylum;unclassified_"Euryarchaeota";	83.38	4.00E-98	359
1155	0	0	0	0	0	0	0	1	3	Euryarchaeota					S000390165 crenarchaeote SRI-298 Lineage=Root;rootrank;Archaea;domain;"Euryarchaeota";phylum;Thermoplasmata;class;Thermoplasmatales;order;Thermoplasmatales_incertae_sedis;family;Thermogymnomonas;genus	84.83	4.00E-108	392
2566	0	0	0	1	1	0	0	0	0	Euryarchaeota					S000390165 crenarchaeote SRI-298 Lineage=Root;rootrank;Archaea;domain;"Euryarchaeota";phylum;Thermoplasmata;class;Thermoplasmatales;order;Thermoplasmatales_incertae_sedis;family;Thermogymnomonas;genus	82.91	7.00E-96	351
426	0	0	0	1	1	0	0	0	0	Euryarchaeota					S002989688 Methanomassiliicoccus luminyensis B10 Lineage=Root;rootrank;Archaea;domain;"Euryarchaeota";phylum;Thermoplasmata;class;Thermoplasmatales;order;Thermoplasmatales_incertae_sedis;family;Thermogymnomonas;genus	84.3	1.00E-103	377
2703	0	0	0	0	1	0	0	0	1	Firmicutes	Bacilli				S000642543 Calditerricola satsumensis (T); YMO81 Lineage=Root;rootrank;Bacteria;domain;Firmicutes;phylum;Bacilli;class;Bacillales;order;Bacillales_incertae_sedis;family;Calditerricola;genus	99.73	0	682
1106	2	1	3	0	7	1	4	6	5	Firmicutes	Bacilli	Bacillales	Alicyclobacillaceae	Alicyclobacillus	S000627911 Tumebacillus ginsengisoli; Gsoil 1105 Lineage=Root;rootrank;Bacteria;domain;Firmicutes;phylum;Bacilli;class;Bacillales;order;Alicyclobacillaceae;family;Tumebacillus;genus	98.11	0	645
1	1	0	1	0	2	2	0	0	0	Firmicutes	Bacilli	Bacillales	Alicyclobacillaceae	Alicyclobacillus	S000627911 Tumebacillus ginsengisoli; Gsoil 1105 Lineage=Root;rootrank;Bacteria;domain;Firmicutes;phylum;Bacilli;class;Bacillales;order;Alicyclobacillaceae;family;Tumebacillus;genus	98.1	0	643
3075	0	0	0	0	0	1	1	0	0	Firmicutes	Bacilli	Bacillales	Alicyclobacillaceae	Alicyclobacillus	S000627911 Tumebacillus ginsengisoli; Gsoil 1105 Lineage=Root;rootrank;Bacteria;domain;Firmicutes;phylum;Bacilli;class;Bacillales;order;Alicyclobacillaceae;family;Tumebacillus;genus	97.69	0	669
1298	0	0	0	0	1	0	0	0	0	Firmicutes	Bacilli	Bacillales	Alicyclobacillaceae	Alicyclobacillus	S000627911 Tumebacillus ginsengisoli; Gsoil 1105 Lineage=Root;rootrank;Bacteria;domain;Firmicutes;phylum;Bacilli;class;Bacillales;order;Alicyclobacillaceae;family;Tumebacillus;genus	94.89	3.00E-164	579
451	0	0	0	0	0	0	0	0	1	Firmicutes	Bacilli	Bacillales	Alicyclobacillaceae	Alicyclobacillus	S000627911 Tumebacillus ginsengisoli; Gsoil 1105 Lineage=Root;rootrank;Bacteria;domain;Firmicutes;phylum;Bacilli;class;Bacillales;order;Alicyclobacillaceae;family;Tumebacillus;genus	96.43	0	643
1307	0	0	0	0	1	0	0	4	5	Firmicutes	Bacilli	Bacillales	Alicyclobacillaceae	Alicyclobacillus	S000735020 Alicyclobacillus pohliae; MP2 Lineage=Root;rootrank;Bacteria;domain;Firmicutes;phylum;Bacilli;class;Bacillales;order;Alicyclobacillaceae;family;Alicyclobacillus;genus	96.68	0	651
798	0	0	0	1	0	0	0	0	0	Firmicutes	Bacilli	Bacillales	Alicyclobacillaceae	Alicyclobacillus	S000735020 Alicyclobacillus pohliae; MP2 Lineage=Root;rootrank;Bacteria;domain;Firmicutes;phylum;Bacilli;class;Bacillales;order;Alicyclobacillaceae;family;Alicyclobacillus;genus	95.7	2.00E-170	599
1379	0	0	0	0	0	0	1	0	2	Firmicutes	Bacilli	Bacillales	Alicyclobacillaceae	Alicyclobacillus	S003263158 Alicyclobacillus pomorum; NBRC 100861 Lineage=Root;rootrank;Bacteria;domain;Firmicutes;phylum;Bacilli;class;Bacillales;order;Alicyclobacillaceae;family;Alicyclobacillus;genus	98.92	0	662
1283	0	1	0	0	0	1	1	0	0	Firmicutes	Bacilli	Bacillales	Alicyclobacillaceae	Alicyclobacillus	S003263834 Alicyclobacillus kakegawensis; NBRC 103104 Lineage=Root;rootrank;Bacteria;domain;Firmicutes;phylum;Bacilli;class;Bacillales;order;Alicyclobacillaceae;family;Alicyclobacillus;genus	99.46	0	675
2959	0	0	0	0	0	0	0	0	1	Firmicutes	Bacilli	Bacillales	Alicyclobacillaceae	Alicyclobacillus	S003721676 Alicyclobacillus acidoterrestris; UFRRJAT1 Lineage=Root;rootrank;Bacteria;domain;Firmicutes;phylum;Bacilli;class;Bacillales;order;Alicyclobacillaceae;family;Alicyclobacillus;genus	97.44	0	664
1930	0	1	0	0	1	2	4	0	2	Firmicutes	Bacilli	Bacillales	Bacillaceae		S000319481 Bacillus horti; A62 Lineage=Root;rootrank;Bacteria;domain;Firmicutes;phylum;Bacilli;class;Bacillales;order;Bacillaceae 1;family;Bacillus;genus	99.49	0	712
448	0	0	1	0	0	0	0	0	0	Firmicutes	Bacilli	Bacillales	Bacillaceae		S001093971 Bacillus halochares (T); MSS4 Lineage=Root;rootrank;Bacteria;domain;Firmicutes;phylum;Bacilli;class;Bacillales;order;Bacillaceae 1;family;Bacillus;genus	97.37	4.00E-09	63.9
1054	1	54	11	0	5	15	14	9	23	Firmicutes	Bacilli	Bacillales	Bacillaceae		S001152025 Geomicrobium halophilum; HN5 Lineage=Root;rootrank;Bacteria;domain;Firmicutes;phylum;Bacilli;class;Bacillales;order;Bacillaceae 2;family;Geomicrobium;genus	100	0	688
1439	0	0	0	0	0	0	2	0	0	Firmicutes	Bacilli	Bacillales	Bacillaceae		S001199925 Bacillus persepolensis (T); HS-136 Lineage=Root;rootrank;Bacteria;domain;Firmicutes;phylum;Bacilli;class;Bacillales;order;Bacillaceae 1;family;Bacillus;genus	98.93	0	667
3103	0	2	2	0	2	0	1	11	21	Firmicutes	Bacilli	Bacillales	Bacillaceae		S001745278 Bacillus saliphilus; M1S6-14 Lineage=Root;rootrank;Bacteria;domain;Firmicutes;phylum;Bacilli;class;Bacillales;order;Sporolactobacillaceae;family;Sporolactobacillaceae_incertae_sedis;genus	96.77	5.00E-177	621
2912	1	12	6	26	3	21	5	22	52	Firmicutes	Bacilli	Bacillales	Bacillaceae		S001873684 Bacillus agaradhaerens; 2007 Lineage=Root;rootrank;Bacteria;domain;Firmicutes;phylum;Bacilli;class;Bacillales;order;Sporolactobacillaceae;family;Sporolactobacillaceae_incertae_sedis;genus	99.46	0	676
2904	0	7	9	1	2	1	0	0	2	Firmicutes	Bacilli	Bacillales	Bacillaceae		S003716713 Bacillus foraminis; S3-2 Lineage=Root;rootrank;Bacteria;domain;Firmicutes;phylum;Bacilli;class;Bacillales;order;Bacillaceae 1;family;Bacillus;genus	98.39	0	654
1760	0	0	0	0	0	0	0	1	1	Firmicutes	Bacilli	Bacillales	Bacillaceae	Alkalibacillus	S003263839 Alkalibacillus haloalkaliphilus; NBRC 103110 Lineage=Root;rootrank;Bacteria;domain;Firmicutes;phylum;Bacilli;class;Bacillales;order;Bacillaceae 2;family;Alkalibacillus;genus	100	0	684
899	4	0	0	0	2	1	0	0	0	Firmicutes	Bacilli	Bacillales	Bacillaceae	Anaerobacillus	S000473524 Bacillus patagoniensis (T); PAT 05 Lineage=Root;rootrank;Bacteria;domain;Firmicutes;phylum;Bacilli;class;Bacillales;order;Bacillaceae 1;family;Bacillus;genus	96.96	0	664
341	4	10	12	14	5	11	16	15	7	Firmicutes	Bacilli	Bacillales	Bacillaceae	Bacillus	S000967349 Bacillus firmus; TSG18 Lineage=Root;rootrank;Bacteria;domain;Firmicutes;phylum;Bacilli;class;Bacillales;order;Bacillaceae 1;family;Bacillus;genus	99.19	0	671
2340	39	100	274	166	127	217	220	111	173	Firmicutes	Bacilli	Bacillales	Bacillaceae	Bacillus	S002911293 Bacillus alkalitelluris; DT29-2 Lineage=Root;rootrank;Bacteria;domain;Firmicutes;phylum;Bacilli;class;Bacillales;order;Bacillaceae 1;family;Bacillus;genus	100	0	688
1465	0	0	0	0	0	1	0	0	0	Firmicutes	Bacilli	Bacillales	Bacillaceae	Geobacillus	S000410520 Anoxybacillus toebii; NS1-1 Lineage=Root;rootrank;Bacteria;domain;Firmicutes;phylum;Bacilli;class;Bacillales;order;unclassified_Bacillales;	99.23	0	702
2468	1	1	5	0	5	2	4	1	1	Firmicutes	Bacilli	Bacillales	Bacillaceae	Geobacillus	S002350822 Bacillus thermantarcticus; type strain: DSM 9572; 3 Lineage=Root;rootrank;Bacteria;domain;Firmicutes;phylum;Bacilli;class;Bacillales;order;Bacillaceae 1;family;Geobacillus;genus	100	0	686
783	0	0	0	0	1	0	0	0	0	Firmicutes	Bacilli	Bacillales	Bacillaceae	Geobacillus	S002354992 Geobacillus debilis; D33-8 Lineage=Root;rootrank;Bacteria;domain;Firmicutes;phylum;Bacilli;class;Bacillales;order;Bacillaceae 1;family;Geobacillus;genus	99.73	0	678
427	15	0	10	2	6	6	6	2	4	Firmicutes	Bacilli	Bacillales	Bacillaceae	Geobacillus	S003754539 Aeribacillus pallidus; C8 Lineage=Root;rootrank;Bacteria;domain;Firmicutes;phylum;Bacilli;class;Bacillales;order;Bacillaceae 1;family;Aeribacillus;genus	100	0	688
368	3	25	94	2	17	31	37	11	10	Firmicutes	Bacilli	Bacillales	Bacillaceae	Halobacillus	S003290377 Halobacillus alkaliphilus; ZSTB206 Lineage=Root;rootrank;Bacteria;domain;Firmicutes;phylum;Bacilli;class;Bacillales;order;Bacillaceae 2;family;Halobacillus;genus	100	0	688
2734	0	0	1	0	0	4	0	1	1	Firmicutes	Bacilli	Bacillales	Bacillaceae	Marinococcus	S003659903 Marinococcus luteus; KCTC 13214 Lineage=Root;rootrank;Bacteria;domain;Firmicutes;phylum;Bacilli;class;Bacillales;order;Bacillaceae 2;family;Marinococcus;genus	99.46	0	676
3197	0	0	0	0	0	0	1	0	0	Firmicutes	Bacilli	Bacillales	Bacillaceae	Marinococcus	S003659903 Marinococcus luteus; KCTC 13214 Lineage=Root;rootrank;Bacteria;domain;Firmicutes;phylum;Bacilli;class;Bacillales;order;Bacillaceae 2;family;Marinococcus;genus	90.64	1.00E-138	494
317	0	0	0	0	0	0	0	1	0	Firmicutes	Bacilli	Bacillales	Bacillaceae		S000319481 Bacillus horti; A62 Lineage=Root;rootrank;Bacteria;domain;Firmicutes;phylum;Bacilli;class;Bacillales;order;Bacillaceae 1;family;Bacillus;genus	94.89	2.00E-165	582
913	2	34	9	2	0	0	1	1	0	Firmicutes	Bacilli	Bacillales	Bacillaceae		S000446630 Virgibacillus carmonensis (T); type strain: LMG 20964 Lineage=Root;rootrank;Bacteria;domain;Firmicutes;phylum;Bacilli;class;Bacillales;order;Bacillaceae 2;family;Virgibacillus;genus	99.73	0	680
1217	0	0	0	0	0	0	1	0	0	Firmicutes	Bacilli	Bacillales	Bacillaceae		S000824198 Bacillus chagannorensis (T); type strain: CG-15 Lineage=Root;rootrank;Bacteria;domain;Firmicutes;phylum;Bacilli;class;Bacillales;order;Sporolactobacillaceae;family;Sporolactobacillaceae_incertae_sedis;genus	90.82	1.00E-147	523
1171	0	0	1	0	1	0	0	0	0	Firmicutes	Bacilli	Bacillales	Bacillaceae		S001352202 Bacillus beveridgei (T); MLTeJB Lineage=Root;rootrank;Bacteria;domain;Firmicutes;phylum;Bacilli;class;Bacillales;order;Sporolactobacillaceae;family;Sporolactobacillaceae_incertae_sedis;genus	95.41	4.00E-178	625
824	0	0	1	0	0	0	0	0	0	Firmicutes	Bacilli	Bacillales	Bacillaceae		S001744215 Ornithinibacillus contaminans (T); type strain: CCUG 53201 Lineage=Root;rootrank;Bacteria;domain;Firmicutes;phylum;Bacilli;class;Bacillales;order;Bacillaceae 2;family;Ornithinibacillus;genus	95.71	2.00E-170	599
1410	0	0	0	4	0	1	0	3	0	Firmicutes	Bacilli	Bacillales	Bacillaceae		S002222544 Bacillus acidicola; YE4-D4-1-CH Lineage=Root;rootrank;Bacteria;domain;Firmicutes;phylum;Bacilli;class;Bacillales;order;Bacillaceae 1;family;Bacillus;genus	97.86	0	643
1625	0	0	0	0	0	0	0	1	0	Firmicutes	Bacilli	Bacillales	Bacillaceae		S002440906 Oceanobacillus indicireducens; A21 Lineage=Root;rootrank;Bacteria;domain;Firmicutes;phylum;Bacilli;class;Bacillales;order;Bacillaceae 2;family;Oceanobacillus;genus	95.65	1.00E-178	627
1904	3	10	1	15	1	8	6	7	6	Firmicutes	Bacilli	Bacillales	Bacillaceae		S002918188 Bacillus alcalophilus; FJAT-10694 Lineage=Root;rootrank;Bacteria;domain;Firmicutes;phylum;Bacilli;class;Bacillales;order;Bacillaceae 1;family;Bacillus;genus	98.21	0	684
2164	2	66	23	68	18	46	29	25	107	Firmicutes	Bacilli	Bacillales	Bacillaceae		S003279980 Bacillus krulwichiae; CS1(1) Lineage=Root;rootrank;Bacteria;domain;Firmicutes;phylum;Bacilli;class;Bacillales;order;Bacillaceae 1;family;Bacillus;genus	99.49	0	712
708	1	0	1	0	0	0	0	0	0	Firmicutes	Bacilli	Bacillales	Bacillaceae		S003314167 Bacillus thermoamylovorans; ASC815 Lineage=Root;rootrank;Bacteria;domain;Firmicutes;phylum;Bacilli;class;Bacillales;order;Bacillaceae 1;family;Bacillus;genus	98.21	0	682
2058	6	28	51	25	68	72	59	26	18	Firmicutes	Bacilli	Bacillales	Bacillaceae		S003615208 Bacillus decolorationis; IARI-ABSL-2 Lineage=Root;rootrank;Bacteria;domain;Firmicutes;phylum;Bacilli;class;Bacillales;order;Bacillaceae 1;family;Bacillus;genus	100	0	684
1679	0	0	0	0	0	0	0	1	0	Firmicutes	Bacilli	Bacillales	Bacillaceae		S003721897 Virgibacillus koreensis; AL-37 Lineage=Root;rootrank;Bacteria;domain;Firmicutes;phylum;Bacilli;class;Bacillales;order;Bacillaceae 2;family;unclassified_Bacillaceae 2;	95.95	0	638
910	13	55	96	68	40	196	144	96	52	Firmicutes	Bacilli	Bacillales	Bacillaceae		S003745482 Bacillus infantis; CCNWQLS5 Lineage=Root;rootrank;Bacteria;domain;Firmicutes;phylum;Bacilli;class;Bacillales;order;unclassified_Bacillales;	98.66	0	660
184	0	0	2	0	4	1	0	5	3	Firmicutes	Bacilli	Bacillales	Paenibacillaceae		S003312190 Aneurinibacillus migulanus; G1 Lineage=Root;rootrank;Bacteria;domain;Firmicutes;phylum;Bacilli;class;Bacillales;order;Paenibacillaceae 2;family;Aneurinibacillus;genus	96.51	6.00E-176	617
1053	2	6	8	17	8	20	12	25	13	Firmicutes	Bacilli	Bacillales	Paenibacillaceae	Ammoniphilus	S000000782 Ammoniphilus oxalaticus (T); RAOx1 Lineage=Root;rootrank;Bacteria;domain;Firmicutes;phylum;Bacilli;class;Bacillales;order;Paenibacillaceae 2;family;Ammoniphilus;genus	99.19	0	669
1461	3	7	14	8	19	35	19	9	12	Firmicutes	Bacilli	Bacillales	Paenibacillaceae	Brevibacillus	S000278683 Brevibacillus limnophilus (T); DSM 6472 Lineage=Root;rootrank;Bacteria;domain;Firmicutes;phylum;Bacilli;class;Bacillales;order;Paenibacillaceae 1;family;Brevibacillus;genus	100	0	686
1542	0	3	6	1	3	2	2	1	1	Firmicutes	Bacilli	Bacillales	Paenibacillaceae	Brevibacillus	S000539548 Brevibacillus levickii; R-22013 Lineage=Root;rootrank;Bacteria;domain;Firmicutes;phylum;Bacilli;class;Bacillales;order;Paenibacillaceae 1;family;Brevibacillus;genus	96.77	2.00E-176	619
2205	0	2	2	0	0	0	0	0	7	Firmicutes	Bacilli	Bacillales	Paenibacillaceae	Brevibacillus	S000539548 Brevibacillus levickii; R-22013 Lineage=Root;rootrank;Bacteria;domain;Firmicutes;phylum;Bacilli;class;Bacillales;order;Paenibacillaceae 1;family;Brevibacillus;genus	97.19	0	658
675	1	1	6	4	3	6	4	6	5	Firmicutes	Bacilli	Bacillales	Paenibacillaceae	Brevibacillus	S003715580 Brevibacillus thermoruber; C Lineage=Root;rootrank;Bacteria;domain;Firmicutes;phylum;Bacilli;class;Bacillales;order;Paenibacillaceae 1;family;Brevibacillus;genus	100	0	686
1776	0	0	0	0	0	0	0	0	1	Firmicutes	Bacilli	Bacillales	Paenibacillaceae	Brevibacillus	S003715584 Brevibacillus thermoruber; I Lineage=Root;rootrank;Bacteria;domain;Firmicutes;phylum;Bacilli;class;Bacillales;order;Paenibacillaceae 1;family;Brevibacillus;genus	95.97	5.00E-172	604
456	3	0	2	1	1	9	5	2	6	Firmicutes	Bacilli	Bacillales	Paenibacillaceae	Cohnella	S001551420 Cohnella luojiensis (T); HY-22R Lineage=Root;rootrank;Bacteria;domain;Firmicutes;phylum;Bacilli;class;Bacillales;order;Paenibacillaceae 1;family;Cohnella;genus	100	0	684
2954	0	0	0	1	0	0	0	0	0	Firmicutes	Bacilli	Bacillales	Paenibacillaceae	Cohnella	S002410201 Cohnella fontinalis; NBM18 Lineage=Root;rootrank;Bacteria;domain;Firmicutes;phylum;Bacilli;class;Bacillales;order;Paenibacillaceae 1;family;Cohnella;genus	97.95	0	675
114	0	0	0	0	0	0	1	0	0	Firmicutes	Bacilli	Bacillales	Paenibacillaceae	Cohnella	S003264123 Cohnella fontinalis; NBRC 104957 Lineage=Root;rootrank;Bacteria;domain;Firmicutes;phylum;Bacilli;class;Bacillales;order;Paenibacillaceae 1;family;Cohnella;genus	99.23	0	702
3176	0	0	0	0	0	0	0	0	1	Firmicutes	Bacilli	Bacillales	Paenibacillaceae	Paenibacillus	S000089478 Paenibacillus koleovorans (T); TB Lineage=Root;rootrank;Bacteria;domain;Firmicutes;phylum;Bacilli;class;Bacillales;order;Paenibacillaceae 1;family;Paenibacillus;genus	95.72	2.00E-170	599
3244	0	0	0	0	1	0	0	0	0	Firmicutes	Bacilli	Bacillales	Paenibacillaceae	Paenibacillus	S000388421 Paenibacillus chinjuensis (T); WN9 Lineage=Root;rootrank;Bacteria;domain;Firmicutes;phylum;Bacilli;class;Bacillales;order;Paenibacillaceae 1;family;Paenibacillus;genus	95.7	8.00E-170	597
874	0	0	0	0	1	5	2	2	1	Firmicutes	Bacilli	Bacillales	Paenibacillaceae	Paenibacillus	S000483843 Paenibacillus mendelii (T); C/2=CCM 4839 Lineage=Root;rootrank;Bacteria;domain;Firmicutes;phylum;Bacilli;class;Bacillales;order;Paenibacillaceae 1;family;Paenibacillus;genus	99.46	0	675
953	1	0	0	1	1	1	3	0	0	Firmicutes	Bacilli	Bacillales	Paenibacillaceae	Paenibacillus	S000496374 Paenibacillus hodogayensis (T); SG Lineage=Root;rootrank;Bacteria;domain;Firmicutes;phylum;Bacilli;class;Bacillales;order;Paenibacillaceae 1;family;Paenibacillus;genus	95.42	5.00E-167	588
2693	0	0	2	0	1	1	0	0	0	Firmicutes	Bacilli	Bacillales	Paenibacillaceae	Paenibacillus	S000496374 Paenibacillus hodogayensis (T); SG Lineage=Root;rootrank;Bacteria;domain;Firmicutes;phylum;Bacilli;class;Bacillales;order;Paenibacillaceae 1;family;Paenibacillus;genus	97.18	0	660
2927	0	0	0	0	0	0	1	1	0	Firmicutes	Bacilli	Bacillales	Paenibacillaceae	Paenibacillus	S000538926 Paenibacillus ruminocola; G22 Lineage=Root;rootrank;Bacteria;domain;Firmicutes;phylum;Bacilli;class;Bacillales;order;Paenibacillaceae 1;family;Paenibacillus;genus	98.38	0	649
2361	0	0	0	0	1	0	0	0	0	Firmicutes	Bacilli	Bacillales	Paenibacillaceae	Paenibacillus	S000769678 Paenibacillus tarimensis (T); SA-7-6 Lineage=Root;rootrank;Bacteria;domain;Firmicutes;phylum;Bacilli;class;Bacillales;order;Paenibacillaceae 1;family;Paenibacillus;genus	96.46	0	652
1121	0	0	1	0	0	0	1	0	0	Firmicutes	Bacilli	Bacillales	Paenibacillaceae	Paenibacillus	S000892546 Paenibacillus contaminans (T); CKOBP-6 Lineage=Root;rootrank;Bacteria;domain;Firmicutes;phylum;Bacilli;class;Bacillales;order;Paenibacillaceae 1;family;Paenibacillus;genus	95.91	0	634
2210	0	0	0	0	0	0	1	0	0	Firmicutes	Bacilli	Bacillales	Paenibacillaceae	Paenibacillus	S000892546 Paenibacillus contaminans (T); CKOBP-6 Lineage=Root;rootrank;Bacteria;domain;Firmicutes;phylum;Bacilli;class;Bacillales;order;Paenibacillaceae 1;family;Paenibacillus;genus	95.64	4.00E-178	625
1727	0	0	0	0	0	0	0	1	0	Firmicutes	Bacilli	Bacillales	Paenibacillaceae	Paenibacillus	S001096866 Paenibacillus aestuarii (T); CJ25 Lineage=Root;rootrank;Bacteria;domain;Firmicutes;phylum;Bacilli;class;Bacillales;order;Paenibacillaceae 1;family;Paenibacillus;genus	97.04	4.00E-178	625
1750	0	0	0	0	0	0	1	1	3	Firmicutes	Bacilli	Bacillales	Paenibacillaceae	Paenibacillus	S001188283 Paenibacillus chitinolyticus; 146XG36 Lineage=Root;rootrank;Bacteria;domain;Firmicutes;phylum;Bacilli;class;Bacillales;order;Paenibacillaceae 1;family;unclassified_Paenibacillaceae 1;	95.16	2.00E-166	586
1022	0	0	0	0	1	0	0	0	0	Firmicutes	Bacilli	Bacillales	Paenibacillaceae	Paenibacillus	S001199466 Cohnella laeviribosi; 4-7 Lineage=Root;rootrank;Bacteria;domain;Firmicutes;phylum;Bacilli;class;Bacillales;order;Paenibacillaceae 1;family;Cohnella;genus	94.34	2.00E-160	566
1001	6	2	4	1	4	8	5	1	5	Firmicutes	Bacilli	Bacillales	Paenibacillaceae	Paenibacillus	S001292623 Paenibacillus pueri (T); b09i-3 Lineage=Root;rootrank;Bacteria;domain;Firmicutes;phylum;Bacilli;class;Bacillales;order;Paenibacillaceae 1;family;Paenibacillus;genus	100	0	688
466	0	0	0	0	0	0	0	0	1	Firmicutes	Bacilli	Bacillales	Paenibacillaceae	Paenibacillus	S001328965 Paenibacillus larvae; BMG 245 Lineage=Root;rootrank;Bacteria;domain;Firmicutes;phylum;Bacilli;class;Bacillales;order;Paenibacillaceae 1;family;Paenibacillus;genus	95.1	4.00E-163	575
1183	0	0	1	1	0	0	4	1	0	Firmicutes	Bacilli	Bacillales	Paenibacillaceae	Paenibacillus	S001353124 Paenibacillus residui; MC-247 Lineage=Root;rootrank;Bacteria;domain;Firmicutes;phylum;Bacilli;class;Bacillales;order;Paenibacillaceae 1;family;Paenibacillus;genus	95.69	8.00E-170	597
955	0	0	1	0	0	0	0	0	0	Firmicutes	Bacilli	Bacillales	Paenibacillaceae	Paenibacillus	S001353124 Paenibacillus residui; MC-247 Lineage=Root;rootrank;Bacteria;domain;Firmicutes;phylum;Bacilli;class;Bacillales;order;Paenibacillaceae 1;family;Paenibacillus;genus	95.98	2.00E-171	603
1366	0	0	0	0	0	0	1	0	0	Firmicutes	Bacilli	Bacillales	Paenibacillaceae	Paenibacillus	S001353124 Paenibacillus residui; MC-247 Lineage=Root;rootrank;Bacteria;domain;Firmicutes;phylum;Bacilli;class;Bacillales;order;Paenibacillaceae 1;family;Paenibacillus;genus	93.55	2.00E-156	553
797	0	0	0	1	0	0	0	0	0	Firmicutes	Bacilli	Bacillales	Paenibacillaceae	Paenibacillus	S001873198 Paenibacillus amylolyticus; KUDC1046 Lineage=Root;rootrank;Bacteria;domain;Firmicutes;phylum;Bacilli;class;Bacillales;order;Paenibacillaceae 1;family;Paenibacillus;genus	95.64	5.00E-167	588
2720	0	0	0	1	0	0	0	0	0	Firmicutes	Bacilli	Bacillales	Paenibacillaceae	Paenibacillus	S002223073 Paenibacillus ginsengisoli; LMG 23406 Lineage=Root;rootrank;Bacteria;domain;Firmicutes;phylum;Bacilli;class;Bacillales;order;Paenibacillaceae 1;family;Paenibacillus;genus	95.42	4.00E-168	592
748	11	2	3	9	45	14	11	13	15	Firmicutes	Bacilli	Bacillales	Paenibacillaceae	Paenibacillus	S002354952 Paenibacillus motobuensis; CCUG 60350 Lineage=Root;rootrank;Bacteria;domain;Firmicutes;phylum;Bacilli;class;Bacillales;order;Paenibacillaceae 1;family;Paenibacillus;genus	95.96	2.00E-171	603
1794	0	0	0	0	0	0	0	0	1	Firmicutes	Bacilli	Bacillales	Paenibacillaceae	Paenibacillus	S002354952 Paenibacillus motobuensis; CCUG 60350 Lineage=Root;rootrank;Bacteria;domain;Firmicutes;phylum;Bacilli;class;Bacillales;order;Paenibacillaceae 1;family;Paenibacillus;genus	96.41	0	643
323	0	0	0	0	0	0	0	1	0	Firmicutes	Bacilli	Bacillales	Paenibacillaceae	Paenibacillus	S002911715 Paenibacillus nanensis; XB-2S-8 Lineage=Root;rootrank;Bacteria;domain;Firmicutes;phylum;Bacilli;class;Bacillales;order;Paenibacillaceae 1;family;Paenibacillus;genus	96.51	8.00E-175	614
1389	2	1	9	3	3	7	6	3	5	Firmicutes	Bacilli	Bacillales	Paenibacillaceae	Paenibacillus	S002917639 Paenibacillus harenae; DHXJ21 Lineage=Root;rootrank;Bacteria;domain;Firmicutes;phylum;Bacilli;class;Bacillales;order;Paenibacillaceae 1;family;Paenibacillus;genus	99.23	0	708

486	0	0	0	0	0	1	0	0	0	Firmicutes	Bacilli	Bacillales	Paenibacillaceae	Paenibacillus	S003259643 Paenibacillus soli; GYR2 Lineage=Root;rootrank;Bacteria;domain;Firmicutes;phylum;Bacilli;class;Bacillales;order;Paenibacillaceae 1;family;Paenibacillus;genus	97.86	0	643
255	3	5	2	1	4	7	4	14	5	Firmicutes	Bacilli	Bacillales	Paenibacillaceae	Paenibacillus	S003259669 Paenibacillus agaridevorans; GYB23 Lineage=Root;rootrank;Bacteria;domain;Firmicutes;phylum;Bacilli;class;Bacillales;order;Paenibacillaceae 1;family;Paenibacillus;genus	99.19	0	669
1291	0	0	0	0	0	1	0	0	0	Firmicutes	Bacilli	Bacillales	Paenibacillaceae	Paenibacillus	S003262740 Paenibacillus chondroitinus; NBRC 15376 Lineage=Root;rootrank;Bacteria;domain;Firmicutes;phylum;Bacilli;class;Bacillales;order;Paenibacillaceae 1;family;Paenibacillus;genus	99.23	0	701
1086	0	0	0	1	0	3	1	1	0	Firmicutes	Bacilli	Bacillales	Paenibacillaceae	Paenibacillus	S003281481 Paenibacillus borealis; F12 Lineage=Root;rootrank;Bacteria;domain;Firmicutes;phylum;Bacilli;class;Bacillales;order;Paenibacillaceae 1;family;Paenibacillus;genus	95.69	8.00E-170	597
1918	0	2	1	6	4	1	6	6	2	Firmicutes	Bacilli	Bacillales	Paenibacillaceae	Paenibacillus	S003290119 Paenibacillus castaneae; TA14 Lineage=Root;rootrank;Bacteria;domain;Firmicutes;phylum;Bacilli;class;Bacillales;order;Paenibacillaceae 1;family;Paenibacillus;genus	100	0	686
2788	0	0	0	0	1	0	0	1	0	Firmicutes	Bacilli	Bacillales	Paenibacillaceae	Paenibacillus	S003302204 Paenibacillus borealis; 15 Lineage=Root;rootrank;Bacteria;domain;Firmicutes;phylum;Bacilli;class;Bacillales;order;Paenibacillaceae 1;family;Paenibacillus;genus	99.49	0	710
240	4	4	10	7	7	9	6	9	17	Firmicutes	Bacilli	Bacillales	Paenibacillaceae	Paenibacillus	S003313681 Paenibacillus cineris; HME8285 Lineage=Root;rootrank;Bacteria;domain;Firmicutes;phylum;Bacilli;class;Bacillales;order;Paenibacillaceae 1;family;Paenibacillus;genus	96.44	0	647
2949	0	0	1	0	1	0	1	0	0	Firmicutes	Bacilli	Bacillales	Paenibacillaceae	Paenibacillus	S003614745 Paenibacillus alginolyticus; BJC15-B35 Lineage=Root;rootrank;Bacteria;domain;Firmicutes;phylum;Bacilli;class;Bacillales;order;Paenibacillaceae 1;family;Paenibacillus;genus	94.62	2.00E-171	603
855	1	0	0	0	0	0	0	0	0	Firmicutes	Bacilli	Bacillales	Paenibacillaceae	Paenibacillus	S003614745 Paenibacillus alginolyticus; BJC15-B35 Lineage=Root;rootrank;Bacteria;domain;Firmicutes;phylum;Bacilli;class;Bacillales;order;Paenibacillaceae 1;family;Paenibacillus;genus	94.91	8.00E-175	614
987	1	0	0	0	0	0	0	0	0	Firmicutes	Bacilli	Bacillales	Paenibacillaceae	Paenibacillus	S003614745 Paenibacillus alginolyticus; BJC15-B35 Lineage=Root;rootrank;Bacteria;domain;Firmicutes;phylum;Bacilli;class;Bacillales;order;Paenibacillaceae 1;family;Paenibacillus;genus	94.39	2.00E-171	603
138	0	0	0	0	0	0	0	0	1	Firmicutes	Bacilli	Bacillales	Paenibacillaceae	Paenibacillus	S003614745 Paenibacillus alginolyticus; BJC15-B35 Lineage=Root;rootrank;Bacteria;domain;Firmicutes;phylum;Bacilli;class;Bacillales;order;Paenibacillaceae 1;family;Paenibacillus;genus	93.81	2.00E-165	582
2507	0	0	0	0	1	0	0	0	0	Firmicutes	Bacilli	Bacillales	Paenibacillaceae	Paenibacillus	S003614761 Paenibacillus mendelii; BJC15-B17 Lineage=Root;rootrank;Bacteria;domain;Firmicutes;phylum;Bacilli;class;Bacillales;order;Paenibacillaceae 1;family;Paenibacillus;genus	94.43	1.00E-172	606
2873	0	0	0	0	1	0	0	0	0	Firmicutes	Bacilli	Bacillales	Paenibacillaceae	Paenibacillus	S003616344 Paenibacillus macquariensis; BJC15-D17 Lineage=Root;rootrank;Bacteria;domain;Firmicutes;phylum;Bacilli;class;Bacillales;order;Paenibacillaceae 1;family;Paenibacillus;genus	98.12	0	649
2919	0	0	0	0	0	0	0	2	1	Firmicutes	Bacilli	Bacillales	Paenibacillaceae	Paenibacillus	S003616536 Paenibacillus pectinilyticus; BJC16-A32 Lineage=Root;rootrank;Bacteria;domain;Firmicutes;phylum;Bacilli;class;Bacillales;order;Paenibacillaceae 1;family;Paenibacillus;genus	100	0	682
1219	0	0	0	0	0	1	0	0	0	Firmicutes	Bacilli	Bacillales	Paenibacillaceae	Paenibacillus	S003617360 Paenibacillus illinoisensis; A1-37c-14 Lineage=Root;rootrank;Bacteria;domain;Firmicutes;phylum;Bacilli;class;Bacillales;order;Paenibacillaceae 1;family;Paenibacillus;genus	96.42	0	643
832	0	0	0	0	0	0	0	0	1	Firmicutes	Bacilli	Bacillales	Paenibacillaceae	Paenibacillus	S003718038 Paenibacillus montaniterrae; G1-3-20 Lineage=Root;rootrank;Bacteria;domain;Firmicutes;phylum;Bacilli;class;Bacillales;order;Paenibacillaceae 1;family;Paenibacillus;genus	98.46	0	684
1241	0	0	0	2	0	0	0	0	0	Firmicutes	Bacilli	Bacillales	Paenibacillaceae	Paenibacillus	S003723027 Paenibacillus xylanilyticus; AntCr13 Lineage=Root;rootrank;Bacteria;domain;Firmicutes;phylum;Bacilli;class;Bacillales;order;Paenibacillaceae 1;family;Paenibacillus;genus	94.88	3.00E-164	579
2680	0	1	0	0	0	0	1	0	0	Firmicutes	Bacilli	Bacillales	Paenibacillaceae	Paenibacillus	S003723027 Paenibacillus xylanilyticus; AntCr13 Lineage=Root;rootrank;Bacteria;domain;Firmicutes;phylum;Bacilli;class;Bacillales;order;Paenibacillaceae 1;family;Paenibacillus;genus	95.42	4.00E-178	625
2135	1	0	0	0	0	0	0	0	0	Firmicutes	Bacilli	Bacillales	Paenibacillaceae	Paenibacillus	S003745492 Paenibacillus sepulcri; CCNWQLS71 Lineage=Root;rootrank;Bacteria;domain;Firmicutes;phylum;Bacilli;class;Bacillales;order;Paenibacillaceae 1;family;Paenibacillus;genus	95.45	1.00E-168	593
966	2	1	1	0	2	13	2	6	0	Firmicutes	Bacilli	Bacillales	Paenibacillaceae	Paenibacillus	S003748917 Paenibacillus lautus; KAB_18; R Lineage=Root;rootrank;Bacteria;domain;Firmicutes;phylum;Bacilli;class;Bacillales;order;Paenibacillaceae 1;family;Paenibacillus;genus	100	0	686
1249	0	1	0	2	1	3	9	4	3	Firmicutes	Bacilli	Bacillales	Paenibacillaceae	Paenibacillus	S003752415 Paenibacillus popilliae; Sh-14 Lineage=Root;rootrank;Bacteria;domain;Firmicutes;phylum;Bacilli;class;Bacillales;order;Paenibacillaceae 1;family;Paenibacillus;genus	99.49	0	710
1110	0	0	0	0	1	0	0	0	0	Firmicutes	Bacilli	Bacillales	Paenibacillaceae	Paenibacillus	S003752415 Paenibacillus popilliae; Sh-14 Lineage=Root;rootrank;Bacteria;domain;Firmicutes;phylum;Bacilli;class;Bacillales;order;Paenibacillaceae 1;family;Paenibacillus;genus	95.69	0	634
420	18	3	12	2	11	5	3	0	1	Firmicutes	Bacilli	Bacillales	Paenibacillaceae	Thermobacillus	S003619922 Thermobacillus composti KWC4 Lineage=Root;rootrank;Bacteria;domain;Firmicutes;phylum;Bacilli;class;Bacillales;order;Paenibacillaceae 1;family;Thermobacillus;genus	100	0	688
2782	0	0	3	0	0	1	0	0	0	Firmicutes	Bacilli	Bacillales	Paenibacillaceae	Thermobacillus	S003619922 Thermobacillus composti KWC4 Lineage=Root;rootrank;Bacteria;domain;Firmicutes;phylum;Bacilli;class;Bacillales;order;Paenibacillaceae 1;family;Thermobacillus;genus	96.51	2.00E-175	616
1414	2	0	1	1	1	1	1	2	1	Firmicutes	Bacilli	Bacillales	Paenibacillaceae		S000496374 Paenibacillus hodogayensis (T); SG Lineage=Root;rootrank;Bacteria;domain;Firmicutes;phylum;Bacilli;class;Bacillales;order;Paenibacillaceae 1;family;Paenibacillus;genus	94.62	5.00E-172	604
1692	0	0	0	0	0	0	0	1	0	Firmicutes	Bacilli	Bacillales	Paenibacillaceae		S001096866 Paenibacillus aestuarii (T); CJ25 Lineage=Root;rootrank;Bacteria;domain;Firmicutes;phylum;Bacilli;class;Bacillales;order;Paenibacillaceae 1;family;Paenibacillus;genus	95.42	4.00E-168	592
2433	0	0	0	0	1	0	0	0	0	Firmicutes	Bacilli	Bacillales	Paenibacillaceae		S001292623 Paenibacillus pueri (T); b09i-3 Lineage=Root;rootrank;Bacteria;domain;Firmicutes;phylum;Bacilli;class;Bacillales;order;Paenibacillaceae 1;family;Paenibacillus;genus	92.8	6.00E-151	534
1859	1	0	0	0	6	0	0	0	1	Firmicutes	Bacilli	Bacillales	Paenibacillaceae		S001353124 Paenibacillus residui; MC-247 Lineage=Root;rootrank;Bacteria;domain;Firmicutes;phylum;Bacilli;class;Bacillales;order;Paenibacillaceae 1;family;Paenibacillus;genus	93.42	7.00E-166	584
920	0	0	0	0	0	0	1	0	0	Firmicutes	Bacilli	Bacillales	Paenibacillaceae		S001353124 Paenibacillus residui; MC-247 Lineage=Root;rootrank;Bacteria;domain;Firmicutes;phylum;Bacilli;class;Bacillales;order;Paenibacillaceae 1;family;Paenibacillus;genus	95.15	2.00E-175	616
657	2	2	1	1	3	2	1	1	0	Firmicutes	Bacilli	Bacillales	Paenibacillaceae		S001793416 Cohnella thermotolerans; CCGE2315 Lineage=Root;rootrank;Bacteria;domain;Firmicutes;phylum;Bacilli;class;Bacillales;order;Paenibacillaceae 1;family;Cohnella;genus	94.61	1.00E-162	573
3161	1	0	0	0	0	0	0	0	0	Firmicutes	Bacilli	Bacillales	Paenibacillaceae		S002223012 Paenibacillus agaridevorans; R-42742 Lineage=Root;rootrank;Bacteria;domain;Firmicutes;phylum;Bacilli;class;Bacillales;order;Paenibacillaceae 1;family;Paenibacillus;genus	93.67	1.00E-167	590
1663	1	0	0	0	0	1	0	0	0	Firmicutes	Bacilli	Bacillales	Paenibacillaceae		S002354952 Paenibacillus motobuensis; CCUG 60350 Lineage=Root;rootrank;Bacteria;domain;Firmicutes;phylum;Bacilli;class;Bacillales;order;Paenibacillaceae 1;family;Paenibacillus;genus	93.61	2.00E-165	582
364	0	1	2	1	1	13	15	2	5	Firmicutes	Bacilli	Bacillales	Paenibacillaceae		S003278233 Paenibacillus barcinonensis; E14 Lineage=Root;rootrank;Bacteria;domain;Firmicutes;phylum;Bacilli;class;Bacillales;order;Paenibacillaceae 1;family;Paenibacillus;genus	93.82	4.00E-158	558
1873	0	0	1	0	1	0	1	0	0	Firmicutes	Bacilli	Bacillales	Paenibacillaceae		S003753682 Paenibacillus elgii; MM-B22 Lineage=Root;rootrank;Bacteria;domain;Firmicutes;phylum;Bacilli;class;Bacillales;order;Paenibacillaceae 1;family;Paenibacillus;genus	95.7	0	634
1770	0	0	1	0	0	0	0	0	0	Firmicutes	Bacilli	Bacillales	Paenibacillaceae		S003753682 Paenibacillus elgii; MM-B22 Lineage=Root;rootrank;Bacteria;domain;Firmicutes;phylum;Bacilli;class;Bacillales;order;Paenibacillaceae 1;family;Paenibacillus;genus	94.64	1.00E-172	606
1557	0	0	0	1	0	0	0	0	0	Firmicutes	Bacilli	Bacillales	Paenibacillaceae		S003753682 Paenibacillus elgii; MM-B22 Lineage=Root;rootrank;Bacteria;domain;Firmicutes;phylum;Bacilli;class;Bacillales;order;Paenibacillaceae 1;family;Paenibacillus;genus	95.65	1.00E-178	627
967	0	3	0	33	3	16	22	9	28	Firmicutes	Bacilli	Bacillales	Planococcaceae	Marinibacillus	S002304537 Jeotgalibacillus marinus; R-42990 Lineage=Root;rootrank;Bacteria;domain;Firmicutes;phylum;Bacilli;class;Bacillales;order;Planococcaceae;family;Jeotgalibacillus;genus	100	0	688
2886	3	1	1	0	0	0	0	0	0	Firmicutes	Bacilli	Bacillales	Planococcaceae	Rummeliibacillus	S002166230 Lysinibacillus fusiformis; B116 Lineage=Root;rootrank;Bacteria;domain;Firmicutes;phylum;Bacilli;class;Bacillales;order;Planococcaceae;family;Rummeliibacillus;genus	98.72	0	693
2964	3	1	4	3	3	9	9	20	11	Firmicutes	Bacilli	Bacillales	Planococcaceae	Solibacillus	S000901777 Bacillus cecembensis (T) Lineage=Root;rootrank;Bacteria;domain;Firmicutes;phylum;Bacilli;class;Bacillales;order;Planococcaceae;family;unclassified_Planococcaceae;	99.23	0	706
1563	2	0	17	230	45	17	27	335	22	Firmicutes	Bacilli	Bacillales	Planococcaceae	Sporosarcina	S003752661 Sporosarcina pasteurii; WJ-5 Lineage=Root;rootrank;Bacteria;domain;Firmicutes;phylum;Bacilli;class;Bacillales;order;Planococcaceae;family;Sporosarcina;genus	98.66	0	660
1341	0	0	1	0	2	3	0	0	1	Firmicutes	Bacilli	Bacillales	Planococcaceae	Ureibacillus	S003747590 Ureibacillus suwonensis; FZWP-21 Lineage=Root;rootrank;Bacteria;domain;Firmicutes;phylum;Bacilli;class;Bacillales;order;Planococcaceae;family;Ureibacillus;genus	100	0	686
101	0	0	0	0	0	0	0	2	0	Firmicutes	Bacilli	Bacillales	Planococcaceae	Viridibacillus	S002912137 Bacillus niabensis; LFS80 Lineage=Root;rootrank;Bacteria;domain;Firmicutes;phylum;Bacilli;class;Bacillales;order;Bacillaceae 1;family;Bacillus;genus	92.62	3.00E-159	562
534	0	0	0	2	0	0	0	0	0	Firmicutes	Bacilli	Bacillales	Sporolactobacillaceae		S000652641 Tuberibacillus calidus (T); 607 Lineage=Root;rootrank;Bacteria;domain;Firmicutes;phylum;Bacilli;class;Bacillales;order;Bacillaceae 2;family;Tuberibacillus;genus	99.46	0	675
792	0	1	0	0	0	0	0	0	0	Firmicutes	Bacilli	Bacillales	Staphylococcaceae	Jeotgalicoccus	S002409170 Jeotgalicoccus coquinae; T-43 Lineage=Root;rootrank;Bacteria;domain;Firmicutes;phylum;Bacilli;class;Bacillales;order;Staphylococcaceae;family;Jeotgalicoccus;genus	99.46	0	676
2984	0	12	3	0	0	1	0	1	3	Firmicutes	Bacilli	Bacillales	Staphylococcaceae	Salinicoccus	S000776594 Salinicoccus albus (T); YIM-Y21 Lineage=Root;rootrank;Bacteria;domain;Firmicutes;phylum;Bacilli;class;Bacillales;order;Staphylococcaceae;family;Salinicoccus;genus	100	0	688
2376	0	5	0	2	1	17	4	3	12	Firmicutes	Bacilli	Bacillales	Staphylococcaceae	Staphylococcus	S003283551 Staphylococcus equorum; SCSAAB0024 Lineage=Root;rootrank;Bacteria;domain;Firmicutes;phylum;Bacilli;class;Bacillales;order;Staphylococcaceae;family;Staphylococcus;genus	99.23	0	702
462	0	0	1	0	0	0	0	0	0	Firmicutes	Bacilli	Bacillales	Staphylococcaceae		S000558938 Staphylococcus kloosii; Iv8 Lineage=Root;rootrank;Bacteria;domain;unclassified_Bacteria;	97.06	1.00E-178	627
1749	0	2	1	0	1	1	3	1	2	Firmicutes	Bacilli	Bacillales	Staphylococcaceae		S003755543 Staphylococcus sciuri; UVBAC12 Lineage=Root;rootrank;Bacteria;domain;Firmicutes;phylum;Bacilli;class;Bacillales;order;Staphylococcaceae;family;Staphylococcus;genus	99.73	0	680
2604	2	1	4	1	6	8	8	5	10	Firmicutes	Bacilli	Bacillales	Thermoactinomycetaceae		S000387931 Seinonella peptonophila (T); KCTC 9740 (type strain) Lineage=Root;rootrank;Bacteria;domain;Firmicutes;phylum;Bacilli;class;Bacillales;order;Thermoactinomycetaceae 2;family;Seinonella;genus	92.76	6.00E-151	534
2608	0	0	0	0	0	1	0	0	0	Firmicutes	Bacilli	Bacillales	Thermoactinomycetaceae		S000387931 Seinonella peptonophila (T); KCTC 9740 (type strain) Lineage=Root;rootrank;Bacteria;domain;Firmicutes;phylum;Bacilli;class;Bacillales;order;Thermoactinomycetaceae 2;family;Seinonella;genus	95.13	8.00E-175	614
1570	0	0	0	0	0	0	1	0	0	Firmicutes	Bacilli	Bacillales	Thermoactinomycetaceae		S000387931 Seinonella peptonophila (T); KCTC 9740 (type strain) Lineage=Root;rootrank;Bacteria;domain;Firmicutes;phylum;Bacilli;class;Bacillales;order;Thermoactinomycetaceae 2;family;Seinonella;genus	94.87	1.00E-173	610
3207	0	1	0	0	0	0	0	0	3	Firmicutes	Bacilli	Bacillales	Thermoactinomycetaceae	Mechercharimyces	S000022386 Thermoactinomyces sanguinis; CCUG 38657T Lineage=Root;rootrank;Bacteria;domain;Firmicutes;phylum;Bacilli;class;Bacillales;order;Thermoactinomycetaceae 1;family;unclassified_Thermoactinomycetaceae 1;	93.55	2.00E-156	553
860	0	5	3	0	3	3	5	2	3	Firmicutes	Bacilli	Bacillales	Thermoactinomycetaceae	Mechercharimyces	S003264045 Mechercharimyces mesophilus; NBRC 104191 Lineage=Root;rootrank;Bacteria;domain;Firmicutes;phylum;Bacilli;class;Bacillales;order;Thermoactinomycetaceae 2;family;Mechercharimyces;genus	97.84	0	641
1920	6	8	13	15	10	29	20	11	7	Firmicutes	Bacilli	Bacillales	Thermoactinomycetaceae	Planifilum	S000110722 Planifilum fimeticola (T); H0165 Lineage=Root;rootrank;Bacteria;domain;Firmicutes;phylum;Bacilli;class;Bacillales;order;Thermoactinomycetaceae 2;family;Planifilum;genus	100	0	682
2028	0	0	0	0	0	1	0	0	0	Firmicutes	Bacilli	Bacillales	Thermoactinomycetaceae	Planifilum	S000584753 Planifilum yunnanense (T); LA5 Lineage=Root;rootrank;Bacteria;domain;Firmicutes;phylum;Bacilli;class;Bacillales;order;Thermoactinomycetaceae 2;family;Planifilum;genus	94.87	4.00E-173	608
524	1	0	0	0	0	2	0	0	0	Firmicutes	Bacilli	Bacillales	Thermoactinomycetaceae	Shimazuella	S000090721 Shimazuella kribbensis (T); KCTC 9933; A9500 Lineage=Root;rootrank;Bacteria;domain;Firmicutes;phylum;Bacilli;class;Bacillales;order;Thermoactinomycetaceae 1;family;Shimazuella;genus	95.63	1.00E-177	623
648	0	0	0	0	0	0	1	1	0	Firmicutes	Bacilli	Bacillales	Thermoactinomycetaceae	Shimazuella	S000090721 Shimazuella kribbensis (T); KCTC 9933; A9500 Lineage=Root;rootrank;Bacteria;domain;Firmicutes;phylum;Bacilli;class;Bacillales;order;Thermoactinomycetaceae 1;family;Shimazuella;genus	99.23	0	702
3222	17	3	13	14	8	67	48	27	34	Firmicutes	Bacilli	Bacillales	Thermoactinomycetaceae	Thermoactinomyces	S003747591 Thermoactinomyces vulgaris; FZWP-24 Lineage=Root;rootrank;Bacteria;domain;Firmicutes;phylum;Bacilli;class;Bacillales;order;Thermoactinomycetaceae 1;family;Thermoactinomyces;genus	100	0	686
1934	0	0	0	0	1	0	0	1	0	Firmicutes	Bacilli	Bacillales	Thermoactinomycetaceae		S000022386 Thermoactinomyces sanguinis; CCUG 38657T Lineage=Root;rootrank;Bacteria;domain;Firmicutes;phylum;Bacilli;class;Bacillales;order;Thermoactinomycetaceae 1;family;unclassified_Thermoactinomycetaceae 1;	95.97	2.00E-171	603
1554	0	0	0	0	0	3	0	1	4	Firmicutes	Bacilli	Bacillales	Thermoactinomycetaceae		S000090721 Shimazuella kribbensis (T); KCTC 9933; A9500 Lineage=Root;rootrank;Bacteria;domain;Firmicutes;phylum;Bacilli;class;Bacillales;order;Thermoactinomycetaceae 1;family;Shimazuella;genus	91.79	4.00E-153	542
1096	0	0	0	1	1	0	1	0	0	Firmicutes	Bacilli	Bacillales	Thermoactinomycetaceae		S000090721 Shimazuella kribbensis (T); KCTC 9933; A9500 Lineage=Root;rootrank;Bacteria;domain;Firmicutes;phylum;Bacilli;class;Bacillales;order;Thermoactinomycetaceae 1;family;Shimazuella;genus	91.79	1.00E-152	540
3127	0	0	0	0	0	0	0	0	2	Firmicutes	Bacilli	Bacillales	Thermoactinomycetaceae		S000090721 Shimazuella kribbensis (T); KCTC 9933; A9500 Lineage=Root;rootrank;Bacteria;domain;Firmicutes;phylum;Bacilli;class;Bacillales;order;Thermoactinomycetaceae 1;family;Shimazuella;genus	94.87	4.00E-173	608
2241	0	0	0	0	0	1	0	0	0	Firmicutes	Bacilli	Bacillales	Thermoactinomycetaceae		S000090721 Shimazuella kribbensis (T); KCTC 9933; A9500 Lineage=Root;rootrank;Bacteria;domain;Firmicutes;phylum;Bacilli;class;Bacillales;order;Thermoactinomycetaceae 1;family;Shimazuella;genus	93.01	5.00E-152	538
492	0	0	0	0	0	0	1	0	0	Firmicutes	Bacilli	Bacillales	Thermoactinomycetaceae		S000090721 Shimazuella kribbensis (T); KCTC 9933; A9500 Lineage=Root;rootrank;Bacteria;domain;Firmicutes;phylum;Bacilli;class;Bacillales;order;Thermoactinomycetaceae 1;family;Shimazuella;genus	92.03	3.00E-154	545
2193	0	0	0	0	1	14	8	1	7	Firmicutes	Bacilli	Bacillales	Thermoactinomycetaceae		S000387930 Thermoactinomyces intermedius (T); KCTC 9646 (type strain) Lineage=Root;rootrank;Bacteria;domain;Firmicutes;phylum;Bacilli;class;Bacillales;order;Thermoactinomycetaceae 1;family;Thermoactinomyces;genus	96.23	4.00E-173	608
823	1	0	0	0	0	0	0	0	0	Firmicutes	Bacilli	Bacillales	Thermoactinomycetaceae		S000387930 Thermoactinomyces intermedius (T); KCTC 9646 (type strain) Lineage=Root;rootrank;Bacteria;domain;Firmicutes;phylum;Bacilli;class;Bacillales;order;Thermoactinomycetaceae 1;family;Thermoactinomyces;genus	96.68	0	649
2654	0	0	0	0	0	2	0	1	1	Firmicutes	Bacilli	Bacillales	Thermoactinomycetaceae		S000387931 Seinonella peptonophila (T); KCTC 9740 (type strain) Lineage=Root;rootrank;Bacteria;domain;Firmicutes;phylum;Bacilli;class;Bacillales;order;Thermoactinomycetaceae 2;family;Seinonella;genus	93.33	1.00E-163	577
1479	20	3	7	3	23	6	2	2	2	Firmicutes	Bacilli	Bacillales	Thermoactinomycetaceae		S000626956 Laceyella sacchari; K5 Lineage=Root;rootrank;Bacteria;domain;Firmicutes;phylum;Bacilli;class;Bacillales;order;Thermoactinomycetaceae 1;family;Thermoactinomyces;genus	93.82	1.00E-157	556
2700	0	4	2	2	0	2	3	0	0	Firmicutes	Bacilli	Bacillales	Thermoactinomycetaceae		S003264045 Mechercharimyces mesophilus; NBRC 104191 Lineage=Root;rootrank;Bacteria;domain;Firmicutes;phylum;Bacilli;class;Bacillales;order;Thermoactinomycetaceae 2;family;Mechercharimyces;genus	93.28	8.00E-155	547
2314	0	0	0	2	0	2	1	4	0	Firmicutes	Bacilli	Bacillales	Thermoactinomycetaceae		S003264045 Mechercharimyces mesophilus; NBRC 104191 Lineage=Root;rootrank;Bacteria;domain;Firmicutes;phylum;Bacilli;class;Bacillales;order;Thermoactinomycetaceae 2;family;Mechercharimyces;genus	93.01	4.00E-153	542
2469	1	0	0	0	0	0	0	0	0	Firmicutes	Bacilli	Bacillales	Thermoactinomycetaceae		S003264045 Mechercharimyces mesophilus; NBRC 104191 Lineage=Root;rootrank;Bacteria;domain;Firmicutes;phylum;Bacilli;class;Bacillales;order;Thermoactinomycetaceae 2;family;Mechercharimyces;genus	92.76	2.00E-151	536
1125	0	0	0	0	0	0	0	1	1	Firmicutes	Bacilli	Bacillales	Thermoactinomycetaceae		S003264045 Mechercharimyces mesophilus; NBRC 104191 Lineage=Root;rootrank;Bacteria;domain;Firmicutes;phylum;Bacilli;class;Bacillales;order;Thermoactinomycetaceae 2;family;Mechercharimyces;genus	93.08	2.00E-161	569
2404	0	0	0	0	1	0	0	0	0	Firmicutes	Bacilli	Bacillales	Thermoactinomycetaceae		S003264045 Mechercharimyces mesophilus; NBRC 104191 Lineage=Root;rootrank;Bacteria;domain;Firmicutes;phylum;Bacilli;class;Bacillales;order;Thermoactinomycetaceae 2;family;Mechercharimyces;genus	92.84	9.00E-160	564
676	0	0	0	0	0	0	1	0	0	Firmicutes	Bacilli	Bacillales	Thermoactinomycetaceae		S003264045 Mechercharimyces mesophilus; NBRC 104191 Lineage=Root;rootrank;Bacteria;domain;Firmicutes;phylum;Bacilli;class;Bacillales;order;Thermoactinomycetaceae 2;family;Mechercharimyces;genus	92.45	8.00E-150	531
2456	0	1	0	0	0	0	0	0	0	Firmicutes	Bacilli	Bacillales			S000021406 Oxalophagus oxalicus (T); DSM 5503T Lineage=Root;rootrank;Bacteria;domain;Firmicutes;phylum;Bacilli;class;Bacillales;order;Paenibacillaceae 2;family;Oxalophagus;genus	95.66	3.00E-179	628
2460	0	1	0	0	0	0	0	0	0	Firmicutes	Bacilli	Bacillales			S000021406 Oxalophagus oxalicus (T); DSM 5503T Lineage=Root;rootrank;Bacteria;domain;Firmicutes;phylum;Bacilli;class;Bacillales;order;Paenibacillaceae 2;family;Oxalophagus;genus	94.12	3.00E-169	595
1690	0	1	0	0	0	0	0	0	0	Firmicutes	Bacilli	Bacillales			S000021406 Oxalophagus oxalicus (T); DSM 5503T Lineage=Root;rootrank;Bacteria;domain;Firmicutes;phylum;Bacilli;class;Bacillales;order;Paenibacillaceae 2;family;Oxalophagus;genus	95.4	5.00E-177	621
1994	0	0	0	0	0	1	0	0	0	Firmicutes	Bacilli	Bacillales			S000021406 Oxalophagus oxalicus (T); DSM 5503T Lineage=Root;rootrank;Bacteria;domain;Firmicutes;phylum;Bacilli;class;Bacillales;order;Paenibacillaceae 2;family;Oxalophagus;genus	95.15	2.00E-166	586
785	0	0	1	0	0	0	0	0	0	Firmicutes	Bacilli	Bacillales			S000022386 Thermoactinomyces sanguinis; CCUG 38657T Lineage=Root;rootrank;Bacteria;domain;Firmicutes;phylum;Bacilli;class;Bacillales;order;Thermoactinomycetaceae 1;family;unclassified_Thermoactinomycetaceae 1;	91.82	1.00E-153	544
2013	0	0	0	0	1	0	0	0	0	Firmicutes	Bacilli	Bacillales			S000110722 Planifilum fimeticola (T); H0165 Lineage=Root;rootrank;Bacteria;domain;Firmicutes;phylum;Bacilli;class;Bacillales;order;Thermoactinomycetaceae 2;family;Planifilum;genus	91.67	1.00E-143	510
1734	0	1	0	0	0	0	0	0	0	Firmicutes	Bacilli	Bacillales			S000122260 Bacillus pseudofirmus; OF4 Lineage=Root;rootrank;Bacteria;domain;Firmicutes;phylum;Bacilli;class;Bacillales;order;Bacillaceae 1;family;Bacillus;genus	95.16	5.00E-167	588
1839	0	1	1	0	0	0	0	0	0	Firmicutes	Bacilli	Bacillales			S000130046 Bacillus nitritophilus; IB-256 Lineage=Root;rootrank;Bacteria;domain;Firmicutes;phylum;Bacilli;class;Bacillales;order;Bacillaceae 2;family;Virgibacillus;genus	92.39	4.00E-158	558
1480	0	0	2	5	0	19	29	7	5	Firmicutes	Bacilli	Bacillales			S000130888 Bacillus aminovorans; NCIMB 8292 Lineage=Root;rootrank;Bacteria;domain;Firmicutes;phylum;Bacilli;class;Bacillales;order;Bacillaceae 1;family;Bacillus;genus	99.19	0	675
3130	1	0	0	0	0	0	0	0	0	Firmicutes	Bacilli	Bacillales			S000262209 Geobacillus stearothermophilus Lineage=Root;rootrank;Bacteria;domain;Firmicutes;phylum;Bacilli;class;Bacillales;order;Bacillaceae 1;family;Geobacillus;genus	92.6	1.00E-158	560
214	0	0	0	0	0	1	0	0	0	Firmicutes	Bacilli	Bacillales			S000362993 Bacillus taeanensis (T); BH030017 Lineage=Root;rootrank;Bacteria;domain;Firmicutes;phylum;Bacilli;class;Bacillales;order;Bacillaceae 1;family;Bacillus;genus	94.13	1.00E-168	593
251	1	0	0	0	0	0	0	0	0	Firmicutes	Bacilli	Bacillales			S000406348 Paenibacillus polymyxa; BS04 Lineage=Root;rootrank;Bacteria;domain;Firmicutes;phylum;Bacilli;class;Bacillales;order;Paenibacillaceae 1;family;Paenibacillus;genus	93.4	7.00E-166	584
2235	1	0	0	0	0	0	0	0	0	Firmicutes	Bacilli	Bacillales			S000437567 anaerobic thermophile IC-BH Lineage=Root;rootrank;Bacteria;domain;Firmicutes;phylum;Bacilli;class;Bacillales;order;Bacillaceae 2;family;Tuberibacillus;genus	93.86	5.00E-167	588
1991	0	0	0	0	0	1	0	0	0	Firmicutes	Bacilli	Bacillales			S000437567 anaerobic thermophile IC-BH Lineage=Root;rootrank;Bacteria;domain;Firmicutes;phylum;Bacilli;class;Bacillales;order;Bacillaceae 2;family;Tuberibacillus;genus	92.78	5.00E-152	538
2199	0	0	0	0	0	1	0	0	0	Firmicutes	Bacilli	Bacillales			S000437567 anaerobic thermophile IC-BH Lineage=Root;rootrank;Bacteria;domain;Firmicutes;phylum;Bacilli;class;Bacillales;order;Bacillaceae 2;family;Tuberibacillus;genus	98.72	0	691
2173	0	0	1	0	0	0	0	0	0	Firmicutes	Bacilli	Bacillales			S000498988 Paenibacillus daejeonensis (T); AP-20 Lineage=Root;rootrank;Bacteria;domain;Firmicutes;phylum;Bacilli;class;Bacillales;order;Paenibacillaceae 1;family;Paenibacillus;genus	95.15	2.00E-175	616
1231	0	2	1	1	3	9	16	15	13	Firmicutes	Bacilli	Bacillales			S000544828 Vulcanibacillus modesticaldus (T); type strain: BR Lineage=Root;rootrank;Bacteria;domain;Firmicutes;phylum;Bacilli;class;Bacillales;order;Bacillaceae 2;family;Vulcanibacillus;genus	93.73	6.00E-156	551
2929	0	0	2	0	0	0	0	0	0	Firmicutes	Bacilli	Bacillales			S000586873 Sporolactobacillus inulinus; J1 Lineage=Root;rootrank;Bacteria;domain;Firmicutes;phylum;Bacilli;class;Bacillales;order;Sporolactobacillaceae;family;Sporolactobacillus;genus	99.23	0	706
1741	0	1	0	0	0	0	0	0	0	Firmicutes	Bacilli	Bacillales			S000652641 Tuberibacillus calidus (T); 607 Lineage=Root;rootrank;Bacteria;domain;Firmicutes;phylum;Bacilli;class;Bacillales;order;Bacillaceae 2;family;Tuberibacillus;genus	92.2	1.00E-147	523
2296	0	0	0	0	0	0	1	0	0	Firmicutes	Bacilli	Bacillales			S000652641 Tuberibacillus calidus (T); 607 Lineage=Root;rootrank;Bacteria;domain;Firmicutes;phylum;Bacilli;class;Bacillales;order;Bacillaceae 2;family;Tuberibacillus;genus	91.42	4.00E-143	508
373	0	0	0	1	0	0	0	0	1	Firmicutes	Bacilli	Bacillales			S000653177 Caldalkalibacillus uzonensis (T); JW/WZ-YB58 Lineage=Root;rootrank;Bacteria;domain;Firmicutes;phylum;Bacilli;class;Bacillales;order;Bacillales_incertae_sedis;family;Caldalkalibacillus;genus	91.76	2.00E-145	516
3061	0	5	0	0	0	1	0	1	0	Firmicutes	Bacilli	Bacillales			S000711958 Bacillus aidingensis (T); 17-5 Lineage=Root;rootrank;Bacteria;domain;Firmicutes;phylum;Bacilli;class;Bacillales;order;Bacillaceae 1;family;Bacillus;genus	92.62	1.00E-158	560
1659	2	0	0	0	0	0	0	0	0	Firmicutes	Bacilli	Bacillales			S000711958 Bacillus aidingensis (T); 17-5 Lineage=Root;rootrank;Bacteria;domain;Firmicutes;phylum;Bacilli;class;Bacillales;order;Bacillaceae 1;family;Bacillus;genus	92.84	2.00E-160	566
3261	0	0	1	0	0	0	0	0	0	Firmicutes	Bacilli	Bacillales			S000711958 Bacillus aidingensis (T); 17-5 Lineage=Root;rootrank;Bacteria;domain;Firmicutes;phylum;Bacilli;class;Bacillales;order;Bacillaceae 1;family;Bacillus;genus	94.89	8.00E-165	580
1622	0	0	0	0	0	0	1	0	0	Firmicutes	Bacilli	Bacillales			S000711958 Bacillus aidingensis (T); 17-5 Lineage=Root;rootrank;Bacteria;domain;Firmicutes;phylum;Bacilli;class;Bacillales;order;Bacillaceae 1;family;Bacillus;genus	92.74	6.00E-151	534
3185	0	0	0	0	0	0	0	0	1	Firmicutes	Bacilli	Bacillales			S000711958 Bacillus aidingensis (T); 17-5 Lineage=Root;rootrank;Bacteria;domain;Firmicutes;phylum;Bacilli;class;Bacillales;order;Bacillaceae 1;family;Bacillus;genus	92.84	2.00E-160	566
1430	0	0	0	0	0	0	1	0	0	Firmicutes	Bacilli	Bacillales			S000727797 Paenibacillus ginsengarvi (T); Gsoil 139 Lineage=Root;rootrank;Bacteria;domain;Firmicutes;phylum;Bacilli;class;Bacillales;order;Paenibacillaceae 1;family;Paenibacillus;genus	93.85	2.00E-166	586
1279	0	0	0	0	0	2	0	0	0	Firmicutes	Bacilli	Bacillales			S000806268 Cohnella yongneupensis (T); 5YN10-14 Lineage=Root;rootrank;Bacteria;domain;Firmicutes;phylum;Bacilli;class;Bacillales;order;Paenibacillaceae 1;family;Cohnella;genus	92	2.00E-146	520
2275	0	0	0	0	0	0	0	0	1	Firmicutes	Bacilli	Bacillales			S000824079 Bacillus polygoni (T); YN-1 Lineage=Root;rootrank;Bacteria;domain;Firmicutes;phylum;Bacilli;class;Bacillales;order;Sporolactobacillaceae;family;Sporolactobacillaceae_incertae_sedis;genus	94.9	3.00E-174	612
1866	0	0	1	0	0	0	0	0	0	Firmicutes	Bacilli	Bacillales			S000824199 Salsuginibacillus kocurii (T); type strain: CH9d Lineage=Root;rootrank;Bacteria;domain;Firmicutes;phylum;Bacilli;class;Bacillales;order;Bacillaceae 2;family;Salsuginibacillus;genus	92.74	2.00E-151	536
2292	0	0	3	0	1	0	1	0	0	Firmicutes	Bacilli	Bacillales			S000870713 Calditerricola yamamurae (T); YMO722 Lineage=Root;rootrank;Bacteria;domain;Firmicutes;phylum;Bacilli;class;Bacillales;order;Bacillales_incertae_sedis;family;Calditerricola;genus	92.84	2.00E-160	566
2488	30	1	1	7	4	8	4	3	0	Firmicutes	Bacilli	Bacillales			S000891239 Bacillus longiquaesitum; LMG 23783 Lineage=Root;rootrank;Bacteria;domain;Firmicutes;phylum;Bacilli;class;Bacillales;order;Bacillaceae 1;family;Bacillus;genus	98.99	0	708
1181	12	15	25	4	30	3	1	0	1	Firmicutes	Bacilli	Bacillales			S001199466 Cohnella laeviribosi; 4-7 Lineage=Root;rootrank;Bacteria;domain;Firmicutes;phylum;Bacilli;class;Bacillales;order;Paenibacillaceae 1;family;Cohnella;genus	94.05	1.00E-158	560
2724	0	1	0	0	0	2	0	0	1	Firmicutes	Bacilli	Bacillales			S001199466 Cohnella laeviribosi; 4-7 Lineage=Root;rootrank;Bacteria;domain;Firmicutes;phylum;Bacilli;class;Bacillales;order;Paenibacillaceae 1;family;Cohnella;genus	94.88	3.00E-164	579
33	0	0	0	0	0	0	1	0	0	Firmicutes	Bacilli	Bacillales			S001292623 Paenibacillus pueri (T); b09i-3 Lineage=Root;rootrank;Bacteria;domain;Firmicutes;phylum;Bacilli;class;Bacillales;order;Paenibacillaceae 1;family;Paenibacillus;genus	95.14	2.00E-175	616
2059	0	0	1	0	3	0	0	0	0	Firmicutes	Bacilli	Bacillales			S001551333 Bacillus clausii; DF4 Lineage=Root;rootrank;Bacteria;domain;Firmicutes;phylum;Bacilli;class;Bacillales;order;Bacillaceae 1;family;Geobacillus;genus	92.82	5.00E-152	538
3102	0	0	0	0	0	1	0	0	0	Firmicutes	Bacilli	Bacillales			S001551333 Bacillus clausii; DF4 Lineage=Root;rootrank;Bacteria;domain;Firmicutes;phylum;Bacilli;class;Bacillales;order;Bacillaceae 1;family;Geobacillus;genus	93.61	2.00E-165	582
687	1	0	0	0	0	0	0	0	0	Firmicutes	Bacilli	Bacillales			S001611491 Microaerobacter geothermalis (T); Nad S1 Lineage=Root;rootrank;Bacteria;domain;Firmicutes;phylum;Bacilli;class;Bacillales;order;Bacillales_incertae_sedis;family;Microaerobacter;genus	94.66	1.00E-172	606
1072	0	1	0	0	0	0	0	0	0	Firmicutes	Bacilli	Bacillales			S001611491 Microaerobacter geothermalis (T); Nad S1 Lineage=Root;rootrank;Bacteria;domain;Firmicutes;phylum;Bacilli;class;Bacillales;order;Bacillales_incertae_sedis;family;Microaerobacter;genus	90.64	4.00E-138	492
2889	0	0	0	0	1	0	0	0	0	Firmicutes	Bacilli	Bacillales			S001611491 Microaerobacter geothermalis (T); Nad S1 Lineage=Root;rootrank;Bacteria;domain;Firmicutes;phylum;Bacilli;class;Bacillales;order;Bacillales_incertae_sedis;family;Microaerobacter;genus	93.11	5.00E-162	571
3215	0	0	0	0	1	0	0	0	0	Firmicutes	Bacilli	Bacillales			S002223311 Bacillus hwajinpoensis; UDC350 Lineage=Root;rootrank;Bacteria;domain;Firmicutes;phylum;Bacilli;class;Bacillales;order;Bacillaceae 1;family;Bacillus;genus	93.35	4.00E-163	575
2696	0	0	0	0	0	0	0	1	0	Firmicutes	Bacilli	Bacillales			S002223311 Bacillus hwajinpoensis; UDC350 Lineage=Root;rootrank;Bacteria;domain;Firmicutes;phylum;Bacilli;class;Bacillales;order;Bacillaceae 1;family;Bacillus;genus	93.78	1.00E-157	556
326	0	1	0	0	0	0	0	0	0	Firmicutes	Bacilli	Bacillales			S002446601 Paenibacillus fonticola; 3424BBRRJ Lineage=Root;rootrank;Bacteria;domain;Firmicutes;phylum;Bacilli;class;Bacillales;order;Paenibacillaceae 1;family;Paenibacillus;genus	94.62	1.00E-162	573
2302	9	28	50	171	45	231	247	139	139	Firmicutes	Bacilli	Bacillales			S002912137 Bacillus niabensis; LFS80 Lineage=Root;rootrank;Bacteria;domain;Firmicutes;phylum;Bacilli;class;Bacillales;order;Bacillaceae 1;family;Bacillus;genus	99.23	0	706
2420	0	0	0	0	0	0	0	0	1	Firmicutes	Bacilli	Bacillales			S002959161 Paenibacillus lactis; SCTB119 Lineage=Root;rootrank;Bacteria;domain;Firmicutes;phylum;Bacilli;class;Bacillales;order;Paenibacillaceae 1;family;Paenibacillus;genus	93.35	4.00E-163	575
176	4	10	45	2	18	78	134	10	11	Firmicutes	Bacilli	Bacillales			S002967193 Scopulibacillus darangshiensis; DLS-08 Lineage=Root;rootrank;Bacteria;domain;Firmicutes;phylum;Bacilli;class;Bacillales;order;unclassified_Bacillales;	98.65	0	656
3	1	0	0	0	0	1	0	0	0	Firmicutes	Bacilli	Bacillales			S002967193 Scopulibacillus darangshiensis; DLS-08 Lineage=Root;rootrank;Bacteria;domain;Firmicutes;phylum;Bacilli;class;Bacillales;order;unclassified_Bacillales;	93.53	6.00E-156	551
1153	0	0	0	0	0	0	2	0	0	Firmicutes	Bacilli	Bacillales			S002967193 Scopulibacillus darangshiensis; DLS-08 Lineage=Root;rootrank;Bacteria;domain;Firmicutes;phylum;Bacilli;class;Bacillales;order;unclassified_Bacillales;	96.22	1.00E-172	606
2319	0	0	0	0	0	0	0	0	2	Firmicutes	Bacilli	Bacillales			S003278233 Paenibacillus barcinonensis; E14 Lineage=Root;rootrank;Bacteria;domain;Firmicutes;phylum;Bacilli;class;Bacillales;order;Paenibacillaceae 1;family;Paenibacillus;genus	94.62	4.00E-163	575
1347	0	0	0	0	0	1	0	0	0	Firmicutes	Bacilli	Bacillales			S003279971 Bacillus cellulosilyticus; AS1(1) Lineage=Root;rootrank;Bacteria;domain;Firmicutes;phylum;Bacilli;class;Bacillales;order;Sporolactobacillaceae;family;Sporolactobacillaceae_incertae_sedis;genus	94.46	4.00E-173	608
1531	0	0	0	4	0	0	0	3	1	Firmicutes	Bacilli	Bacillales			S003281482 Bacillus luciferensis; F30 Lineage=Root;rootrank;Bacteria;domain;Firmicutes;phylum;Bacilli;class;Bacillales;order;Bacillaceae 1;family;Bacillus;genus	99.49	0	712

2150	0	0	0	0	1	1	0	0	0	Firmicutes	Bacilli	Bacillales			S003300451 Bacillus vitellinus; A21 Lineage=Root;rootrank;Bacteria;domain;Firmicutes;phylum;Bacilli;class;Bacillales;order;Paenibacillaceae 1;family;Paenibacillus;genus	93.94	3.00E-169	595
1150	0	0	0	0	0	1	0	0	0	Firmicutes	Bacilli	Bacillales			S003304102 Paenibacillus azoreducens; DW-TSB-4 Lineage=Root;rootrank;Bacteria;domain;Firmicutes;phylum;Bacilli;class;Bacillales;order;Paenibacillaceae 1;family;Paenibacillus;genus	94.4	2.00E-170	599
2148	0	0	0	0	0	0	0	1	0	Firmicutes	Bacilli	Bacillales			S003312434 Paucisalibacillus globulus; TN23 Lineage=Root;rootrank;Bacteria;domain;Firmicutes;phylum;Bacilli;class;Bacillales;order;Bacillaceae 2;family;Ornithinibacillus;genus	92.88	2.00E-160	566
819	0	0	0	0	1	1	0	0	0	Firmicutes	Bacilli	Bacillales			S003616818 Paenibacillus xylanilyticus; REN52N Lineage=Root;rootrank;Bacteria;domain;Firmicutes;phylum;Bacilli;class;Bacillales;order;Paenibacillaceae 1;family;Paenibacillus;genus	95.9	2.00E-180	632
35	0	0	1	0	0	1	0	0	0	Firmicutes	Bacilli	Bacillales			S003617112 Bacillus algicola; M1E7a Lineage=Root;rootrank;Bacteria;domain;Firmicutes;phylum;Bacilli;class;Bacillales;order;Bacillaceae 1;family;Bacillus;genus	94.12	4.00E-168	592
1340	0	0	0	0	0	0	1	0	0	Firmicutes	Bacilli	Bacillales			S003716421 Paenibacillus agarexedens; KUDC1785 Lineage=Root;rootrank;Bacteria;domain;Firmicutes;phylum;Bacilli;class;Bacillales;order;Paenibacillaceae 1;family;Paenibacillus;genus	93.33	1.00E-163	577
1843	0	4	0	1	1	4	1	0	1	Firmicutes	Bacilli	Bacillales			S003718619 Bacillus clausii; APT32 Lineage=Root;rootrank;Bacteria;domain;Firmicutes;phylum;Bacilli;class;Bacillales;order;Bacillaceae 1;family;Bacillus;genus	99.49	0	712
2464	0	0	1	0	0	0	0	0	0	Firmicutes	Bacilli	Bacillales			S003746721 Geobacillus subterraneus; RL-2a Lineage=Root;rootrank;Bacteria;domain;Firmicutes;phylum;Bacilli;class;Bacillales;order;Bacillaceae 1;family;Geobacillus;genus	93.67	2.00E-166	586
1565	0	0	0	0	0	0	0	0	2	Firmicutes	Bacilli	Bacillales			S003747591 Thermoactinomyces vulgaris; FZWP-24 Lineage=Root;rootrank;Bacteria;domain;Firmicutes;phylum;Bacilli;class;Bacillales;order;Thermoactinomycetaceae 1;family;Thermoactinomyces;genus	92.56	4.00E-158	558
1419	0	0	0	0	0	1	0	0	0	Firmicutes	Bacilli	Bacillales			S003753682 Paenibacillus elgii; MM-B22 Lineage=Root;rootrank;Bacteria;domain;Firmicutes;phylum;Bacilli;class;Bacillales;order;Paenibacillaceae 1;family;Paenibacillus;genus	93.33	1.00E-163	577
1895	1	0	0	0	0	0	0	0	0	Firmicutes	Bacilli	Haloplasmatales	Haloplasmataceae		S001095420 Haloplasma contractile (T); SSD-17B Lineage=Root;rootrank;Bacteria;domain;"Tenericutes";phylum;Mollicutes;class;Haloplasmatales;order;Haloplasmataceae;family;Haloplasma;genus	88.11	2.00E-120	433
3262	0	0	1	0	0	0	0	0	0	Firmicutes	Bacilli	Haloplasmatales	Haloplasmataceae		S003617113 Bacillus solisalsi; V11SED5 Lineage=Root;rootrank;Bacteria;domain;Firmicutes;phylum;Bacilli;class;Bacillales;order;Bacillaceae 1;family;Bacillus;genus	88.68	2.00E-125	449
179	0	0	0	0	0	0	0	0	1	Firmicutes	Bacilli	Lactobacillales	Aerococcaceae	Facklamia	S000021549 Facklamia tabacinasalis (T); CCUG30090 Lineage=Root;rootrank;Bacteria;domain;Firmicutes;phylum;Bacilli;class;Lactobacillales;order;Aerococcaceae;family;Facklamia;genus	96.16	0	640
76	0	0	0	0	0	0	0	0	1	Firmicutes	Bacilli	Lactobacillales	Carnobacteriaceae	Desemzia	S003719574 Desemzia incerta; SB-B1 Lineage=Root;rootrank;Bacteria;domain;Firmicutes;phylum;Bacilli;class;Lactobacillales;order;Carnobacteriaceae;family;Desemzia;genus	99.49	0	712
28	0	2	1	0	4	1	0	0	1	Firmicutes	Bacilli	Lactobacillales	Carnobacteriaceae		S003712736 Marinilactibacillus psychrotolerans; DYS29 Lineage=Root;rootrank;Bacteria;domain;Firmicutes;phylum;Bacilli;class;Lactobacillales;order;Carnobacteriaceae;family;Marinilactibacillus;genus	97.97	0	686
1597	0	0	0	0	0	0	0	1	0	Firmicutes	Bacilli	Lactobacillales	Lactobacillaceae	Lactobacillus	S003749491 Lactobacillus fuchuensis; LMG 21669 Lineage=Root;rootrank;Bacteria;domain;Firmicutes;phylum;Bacilli;class;Lactobacillales;order;Lactobacillaceae;family;Lactobacillus;genus	99.49	0	712
2191	0	0	0	0	0	0	0	0	1	Firmicutes	Bacilli	Lactobacillales	Lactobacillaceae		S003262885 Lactobacillus malefermentans; NBRC 15905 Lineage=Root;rootrank;Bacteria;domain;Firmicutes;phylum;Bacilli;class;Lactobacillales;order;Lactobacillaceae;family;Lactobacillus;genus	99.23	0	708
2282	0	1	0	0	0	0	0	0	0	Firmicutes	Bacilli	Lactobacillales	Leuconostocaceae		S003258017 Fructobacillus tropaeoli; R-46389 Lineage=Root;rootrank;Bacteria;domain;Firmicutes;phylum;Bacilli;class;Lactobacillales;order;Leuconostocaceae;family;Fructobacillus;genus	91.96	5.00E-147	521
2705	0	2	0	1	0	1	0	0	0	Firmicutes	Bacilli	Lactobacillales	Streptococcaceae	Lactococcus	S003755218 Lactococcus lactis; PON10072 Lineage=Root;rootrank;Bacteria;domain;Firmicutes;phylum;Bacilli;class;Lactobacillales;order;Streptococcaceae;family;Lactococcus;genus	100	0	684
1425	0	0	0	1	1	0	3	0	1	Firmicutes	Bacilli	Turicibacterales	Turicibacteraceae		S002444921 Turicibacter sanguinis; 111732/2010 Lineage=Root;rootrank;Bacteria;domain;Firmicutes;phylum;Erysipelotrichia;class;Erysipelotrichales;order;Erysipelotrichaceae;family;Turicibacter;genus	96.12	2.00E-180	632
1429	0	0	0	0	0	0	0	0	1	Firmicutes	Bacilli				S000090721 Shimazuella kribbensis (T); KCTC 9933; A9500 Lineage=Root;rootrank;Bacteria;domain;Firmicutes;phylum;Bacilli;class;Bacillales;order;Thermoactinomycetaceae 1;family;Shimazuella;genus	90.54	9.00E-145	514
1400	0	1	0	0	0	0	0	0	0	Firmicutes	Bacilli				S000626956 Laceyella sacchari; K5 Lineage=Root;rootrank;Bacteria;domain;Firmicutes;phylum;Bacilli;class;Bacillales;order;Thermoactinomycetaceae 1;family;Thermoactinomyces;genus	90.84	1.00E-147	523
2055	2	0	1	0	0	1	1	0	1	Firmicutes	Bacilli				S000653177 Caldalkalibacillus uzonensis (T); JW/WZ-YB58 Lineage=Root;rootrank;Bacteria;domain;Firmicutes;phylum;Bacilli;class;Bacillales;order;Bacillales_incertae_sedis;family;Caldalkalibacillus;genus	92.86	7.00E-161	568
329	0	1	0	0	0	0	0	0	0	Firmicutes	Bacilli				S000824847 Nitriliruptor alkaliphilus (T); ANL-iso2 Lineage=Root;rootrank;Bacteria;domain;"Actinobacteria";phylum;Actinobacteria;class;Nitriliruptoridae;subclass;Nitriliruptorales;order;Nitriliruptoraceae;family;Nitriliruptor;genus	84.49	2.00E-100	366
2357	0	0	0	0	0	0	2	1	0	Firmicutes	Bacilli				S001093328 Bacillus aeolius; JAM-FM1601 Lineage=Root;rootrank;Bacteria;domain;Firmicutes;phylum;Bacilli;class;Bacillales;order;Bacillaceae 1;family;Bacillus;genus	93.35	1.00E-163	577
1655	1	5	5	4	3	8	6	4	5	Firmicutes	Clostridia	B07			S000016025 Bacillus thermocloacae (T); DSM 5250 Lineage=Root;rootrank;Bacteria;domain;Firmicutes;phylum;Bacilli;class;Bacillales;order;Bacillaceae 1;family;Bacillus;genus	89.07	1.00E-128	460
3113	0	0	0	0	1	1	0	0	0	Firmicutes	Clostridia	B07			S000016025 Bacillus thermocloacae (T); DSM 5250 Lineage=Root;rootrank;Bacteria;domain;Firmicutes;phylum;Bacilli;class;Bacillales;order;Bacillaceae 1;family;Bacillus;genus	88.74	2.00E-126	453
1864	0	0	0	1	0	0	0	0	0	Firmicutes	Clostridia	B07			S000016025 Bacillus thermocloacae (T); DSM 5250 Lineage=Root;rootrank;Bacteria;domain;Firmicutes;phylum;Bacilli;class;Bacillales;order;Bacillaceae 1;family;Bacillus;genus	88.47	2.00E-125	449
81	0	1	7	2	1	1	0	0	0	Firmicutes	Clostridia	B07			S000110722 Planifilum fimeticola (T); H0165 Lineage=Root;rootrank;Bacteria;domain;Firmicutes;phylum;Bacilli;class;Bacillales;order;Thermoactinomycetaceae 2;family;Planifilum;genus	90.19	4.00E-133	475
1718	0	0	0	0	0	0	0	1	0	Firmicutes	Clostridia	B07			S000110722 Planifilum fimeticola (T); H0165 Lineage=Root;rootrank;Bacteria;domain;Firmicutes;phylum;Bacilli;class;Bacillales;order;Thermoactinomycetaceae 2;family;Planifilum;genus	87.28	1.00E-123	444
2155	0	0	0	0	0	0	0	1	0	Firmicutes	Clostridia	B07			S000438509 Moorella glycerini (T); YS6 Lineage=Root;rootrank;Bacteria;domain;Firmicutes;phylum;Clostridia;class;Thermoanaerobacterales;order;Thermoanaerobacteraceae;family;Moorella;genus	88.52	2.00E-132	473
1356	0	1	5	1	1	0	0	0	0	Firmicutes	Clostridia	B07			S000584753 Planifilum yunnanense (T); LA5 Lineage=Root;rootrank;Bacteria;domain;Firmicutes;phylum;Bacilli;class;Bacillales;order;Thermoactinomycetaceae 2;family;Planifilum;genus	89.01	4.00E-128	459
3003	0	0	1	1	1	1	0	0	0	Firmicutes	Clostridia	B07			S000584753 Planifilum yunnanense (T); LA5 Lineage=Root;rootrank;Bacteria;domain;Firmicutes;phylum;Bacilli;class;Bacillales;order;Thermoactinomycetaceae 2;family;Planifilum;genus	89.03	3.00E-135	483
378	0	0	1	0	0	0	0	1	0	Firmicutes	Clostridia	B07			S000584753 Planifilum yunnanense (T); LA5 Lineage=Root;rootrank;Bacteria;domain;Firmicutes;phylum;Bacilli;class;Bacillales;order;Thermoactinomycetaceae 2;family;Planifilum;genus	88.01	3.00E-129	462
283	0	0	0	0	0	1	0	0	0	Firmicutes	Clostridia	B07			S000653177 Caldalkalibacillus uzonensis (T); JW/WZ-YB58 Lineage=Root;rootrank;Bacteria;domain;Firmicutes;phylum;Bacilli;class;Bacillales;order;Bacillales_incertae_sedis;family;Caldalkalibacillus;genus	88.86	1.00E-127	457
582	0	0	0	0	0	0	1	0	0	Firmicutes	Clostridia	B07			S000653177 Caldalkalibacillus uzonensis (T); JW/WZ-YB58 Lineage=Root;rootrank;Bacteria;domain;Firmicutes;phylum;Bacilli;class;Bacillales;order;Bacillales_incertae_sedis;family;Caldalkalibacillus;genus	88.04	3.00E-129	462
53	0	0	0	1	0	0	0	0	0	Firmicutes	Clostridia	B07			S001152889 Thermaerobacter subterraneus; ir-12 Lineage=Root;rootrank;Bacteria;domain;Firmicutes;phylum;Clostridia;class;Clostridiales;order;Clostridiales_Incertae Sedis XVII;family;Thermaerobacter;genus	87.37	1.00E-118	427
686	0	0	0	1	0	0	0	0	0	Firmicutes	Clostridia	B07			S001240925 Desulfotomaculum nigrificans; CINBIN-C2 Lineage=Root;rootrank;Bacteria;domain;Firmicutes;phylum;Clostridia;class;Clostridiales;order;Peptococcaceae 2;family;Desulfotomaculum;genus	86.61	1.00E-113	411
2385	0	0	0	0	1	0	0	0	0	Firmicutes	Clostridia	B07			S001611491 Microaerobacter geothermalis (T); Nad S1 Lineage=Root;rootrank;Bacteria;domain;Firmicutes;phylum;Bacilli;class;Bacillales;order;Bacillales_incertae_sedis;family;Microaerobacter;genus	88.1	3.00E-129	462
780	0	0	0	0	1	0	0	0	0	Firmicutes	Clostridia	B07			S002038681 Aneurinibacillus thermoaerophilus; BHK30 Lineage=Root;rootrank;Bacteria;domain;Firmicutes;phylum;Bacilli;class;Bacillales;order;Paenibacillaceae 2;family;Aneurinibacillus;genus	88.27	1.00E-123	444
403	1	0	5	1	9	7	4	1	2	Firmicutes	Clostridia	B07			S002933275 Desulfotomaculum carboxydivorans CO-1-SRB Lineage=Root;rootrank;Bacteria;domain;Firmicutes;phylum;Clostridia;class;Clostridiales;order;Peptococcaceae 2;family;Desulfotomaculum;genus	89.39	6.00E-131	468
620	0	0	1	0	0	0	0	0	0	Firmicutes	Clostridia	B07			S000110722 Planifilum fimeticola (T); H0165 Lineage=Root;rootrank;Bacteria;domain;Firmicutes;phylum;Bacilli;class;Bacillales;order;Thermoactinomycetaceae 2;family;Planifilum;genus	86.73	3.00E-120	433
1797	0	0	0	0	0	0	0	0	1	Firmicutes	Clostridia	B07			S000383946 Thermanaeromonas toyohensis (T); ToBE Lineage=Root;rootrank;Bacteria;domain;Firmicutes;phylum;Clostridia;class;Thermoanaerobacterales;order;Thermoanaerobacteraceae;family;Thermanaeromonas;genus	87.44	3.00E-124	446
557	0	1	0	0	0	0	0	1	0	Firmicutes	Clostridia	B07			S003286846 Moorella thermoacetica; Y73 Lineage=Root;rootrank;Bacteria;domain;Firmicutes;phylum;Clostridia;class;Thermoanaerobacterales;order;Thermoanaerobacteraceae;family;Moorella;genus	86.1	2.00E-110	399
2232	1	0	1	0	0	0	0	0	0	Firmicutes	Clostridia	BSA2B-08			S002288919 Halothermothrix orenii H 168; H 168; DSM 9562 Lineage=Root;rootrank;Bacteria;domain;Firmicutes;phylum;Clostridia;class;Halanaerobiales;order;Halanaerobiaceae;family;Halothermothrix;genus	89.03	9.00E-135	481
1992	0	0	0	0	0	1	0	0	0	Firmicutes	Clostridia	Clostridiales	Catabacteriaceae		S000260209 Clostridium putrificum (T); DSM 1734 Lineage=Root;rootrank;Bacteria;domain;Firmicutes;phylum;Clostridia;class;Clostridiales;order;Clostridiaceae 1;family;Clostridium sensu stricto;genus	91.89	8.00E-145	514
2587	1	0	1	0	0	0	0	0	0	Firmicutes	Clostridia	Clostridiales	Catabacteriaceae		S002444633 Clostridium botulinum; NAN152 Lineage=Root;rootrank;Bacteria;domain;Firmicutes;phylum;Clostridia;class;Clostridiales;order;Clostridiaceae 1;family;Clostridium sensu stricto;genus	93.46	3.00E-154	545
356	0	0	0	0	1	0	0	0	0	Firmicutes	Clostridia	Clostridiales	Catabacteriaceae		S003289060 Catabacter hongkongensis; 88678 Lineage=Root;rootrank;Bacteria;domain;Firmicutes;phylum;Clostridia;class;Clostridiales;order;unclassified_Clostridiales;	91.35	9.00E-150	531
187	0	0	0	0	0	0	1	0	0	Firmicutes	Clostridia	Clostridiales	Clostridiaceae		S003611802 Oxobacter pfennigii; type strain: DSM 3222; 36 Lineage=Root;rootrank;Bacteria;domain;Firmicutes;phylum;Clostridia;class;Clostridiales;order;Clostridiaceae 1;family;Oxobacter;genus	93.13	4.00E-163	575
2618	0	0	0	0	0	0	1	0	0	Firmicutes	Clostridia	Clostridiales	Clostridiaceae		S003611802 Oxobacter pfennigii; type strain: DSM 3222; 36 Lineage=Root;rootrank;Bacteria;domain;Firmicutes;phylum;Clostridia;class;Clostridiales;order;Clostridiaceae 1;family;Oxobacter;genus	95.4	1.00E-177	623
2627	1	0	0	0	0	0	0	0	0	Firmicutes	Clostridia	Clostridiales	Clostridiaceae	Alkaliphilus	S000018099 Alkaliphilus transvaalensis (T); SAGM1 Lineage=Root;rootrank;Bacteria;domain;Firmicutes;phylum;Clostridia;class;Clostridiales;order;Clostridiaceae 2;family;Alkaliphilus;genus	96.92	0	652
666	0	0	0	0	0	0	0	0	1	Firmicutes	Clostridia	Clostridiales	Clostridiaceae	Alkaliphilus	S000394644 Alkaliphilus crotonatoxidans (T); B11-2 Lineage=Root;rootrank;Bacteria;domain;Firmicutes;phylum;Clostridia;class;Clostridiales;order;Clostridiaceae 2;family;Alkaliphilus;genus	99.48	0	702
397	0	0	0	0	0	1	0	0	0	Firmicutes	Clostridia	Clostridiales	Clostridiaceae	Alkaliphilus	S000806420 Alkaliphilus peptidifermentans (T); Z-7036 Lineage=Root;rootrank;Bacteria;domain;Firmicutes;phylum;Clostridia;class;Clostridiales;order;Clostridiaceae 2;family;Alkaliphilus;genus	96.89	0	647
1805	1	0	1	0	1	3	2	0	1	Firmicutes	Clostridia	Clostridiales	Clostridiaceae	Alkaliphilus	S002290980 Alkaliphilus oremlandii OhILAs Lineage=Root;rootrank;Bacteria;domain;Firmicutes;phylum;Clostridia;class;Clostridiales;order;Clostridiaceae 2;family;Alkaliphilus;genus	98.37	0	645
1765	0	0	0	0	0	0	0	1	0	Firmicutes	Clostridia	Clostridiales	Clostridiaceae	Caloramator	S002228266 Caloramator australicus; BF3 Lineage=Root;rootrank;Bacteria;domain;Firmicutes;phylum;Clostridia;class;Clostridiales;order;Clostridiaceae 1;family;Caloramator;genus	98.64	0	654
3141	0	0	0	1	1	0	1	0	0	Firmicutes	Clostridia	Clostridiales	Clostridiaceae	Caloramator	S002228272 Caloramator australicus; DF4 Lineage=Root;rootrank;Bacteria;domain;Firmicutes;phylum;Clostridia;class;Clostridiales;order;Clostridiaceae 1;family;Caloramator;genus	94.86	4.00E-163	575
1736	0	0	0	1	0	0	0	0	0	Firmicutes	Clostridia	Clostridiales	Clostridiaceae	Caloramator	S002228272 Caloramator australicus; DF4 Lineage=Root;rootrank;Bacteria;domain;Firmicutes;phylum;Clostridia;class;Clostridiales;order;Clostridiaceae 1;family;Caloramator;genus	95.66	1.00E-168	593
439	2	0	0	1	1	6	0	0	1	Firmicutes	Clostridia	Clostridiales	Clostridiaceae	Caminicella	S000769549 Proteiniborus ethanoligenes (T); GW Lineage=Root;rootrank;Bacteria;domain;Firmicutes;phylum;Clostridia;class;Clostridiales;order;Clostridiales_incertae_sedis;family;Proteiniborus;genus	95.62	1.00E-177	623
736	0	0	0	0	0	1	1	0	1	Firmicutes	Clostridia	Clostridiales	Clostridiaceae	Clostridium	S000428722 Clostridium gasigenes (T); DSM 12272 Lineage=Root;rootrank;Bacteria;domain;Firmicutes;phylum;Clostridia;class;Clostridiales;order;Clostridiaceae 1;family;Clostridium sensu stricto;genus	97.59	0	636
2733	0	0	2	0	1	0	0	0	0	Firmicutes	Clostridia	Clostridiales	Clostridiaceae	Clostridium	S000943595 Clostridium caenicola (T); EBR596; EBR-02E-0596 Lineage=Root;rootrank;Bacteria;domain;Firmicutes;phylum;Clostridia;class;Clostridiales;order;Ruminococcaceae;family;Clostridium III;genus	99.73	0	682
764	0	0	0	2	0	0	0	0	0	Firmicutes	Clostridia	Clostridiales	Clostridiaceae	Clostridium	S001744855 Clostridium saccharobutylicum; BS2 Lineage=Root;rootrank;Bacteria;domain;Firmicutes;phylum;Clostridia;class;Clostridiales;order;Clostridiaceae 1;family;Clostridium sensu stricto;genus	99.73	0	680
959	0	0	0	0	0	0	1	0	0	Firmicutes	Clostridia	Clostridiales	Clostridiaceae	Clostridium	S001746047 Clostridium magnum; FM5 Lineage=Root;rootrank;Bacteria;domain;Firmicutes;phylum;Clostridia;class;Clostridiales;order;Clostridiaceae 1;family;Clostridium sensu stricto;genus	96.41	0	641
1094	0	0	0	0	0	1	0	4	0	Firmicutes	Clostridia	Clostridiales	Clostridiaceae	Clostridium	S002291022 Clostridium tetani E88; Massachusetts Lineage=Root;rootrank;Bacteria;domain;Firmicutes;phylum;Clostridia;class;Clostridiales;order;Clostridiaceae 1;family;Clostridium sensu stricto;genus	99.73	0	678
2161	9	6	13	0	9	2	0	0	1	Firmicutes	Clostridia	Clostridiales	Clostridiaceae	Clostridium	S003723521 Clostridium thermocellum; mbf-VZ-096; BC1 Lineage=Root;rootrank;Bacteria;domain;Firmicutes;phylum;Clostridia;class;Clostridiales;order;Ruminococcaceae;family;Clostridium III;genus	96.24	1.00E-173	610
1071	0	1	0	0	0	0	0	0	0	Firmicutes	Clostridia	Clostridiales	Clostridiaceae	Clostridium	S003754537 Clostridium tyrobutyricum; DEY4 Lineage=Root;rootrank;Bacteria;domain;Firmicutes;phylum;Clostridia;class;Clostridiales;order;Clostridiaceae 1;family;Clostridium sensu stricto;genus	97.86	0	643
1804	0	0	0	0	0	1	1	0	0	Firmicutes	Clostridia	Clostridiales	Clostridiaceae	Clostridium	S003755646 Clostridium sartagoforme; AAU1 Lineage=Root;rootrank;Bacteria;domain;Firmicutes;phylum;Clostridia;class;Clostridiales;order;Clostridiaceae 1;family;Clostridium sensu stricto;genus	97.57	0	636
2025	0	0	0	0	0	0	0	2	0	Firmicutes	Clostridia	Clostridiales	Clostridiaceae		S000012021 Sarcina maxima (T); DSM 316 Lineage=Root;rootrank;Bacteria;domain;Firmicutes;phylum;Clostridia;class;Clostridiales;order;Clostridiaceae 1;family;Sarcina;genus	97.3	1.00E-178	627
2711	0	0	0	0	0	0	0	1	0	Firmicutes	Clostridia	Clostridiales	Clostridiaceae		S001098524 Alkaliphilus halophilus (T); E2R Lineage=Root;rootrank;Bacteria;domain;Firmicutes;phylum;Clostridia;class;Clostridiales;order;Clostridiaceae 2;family;Alkaliphilus;genus	95.09	4.00E-173	608
2828	1	0	0	0	0	0	0	0	0	Firmicutes	Clostridia	Clostridiales	Clostridiaceae		S001549688 Clostridium colicanis; JPL_15 Lineage=Root;rootrank;Bacteria;domain;Firmicutes;phylum;Clostridia;class;Clostridiales;order;Clostridiaceae 1;family;Clostridium sensu stricto;genus	99.49	0	710
411	1	1	1	6	1	1	0	1	0	Firmicutes	Clostridia	Clostridiales	Clostridiaceae		S003611802 Oxobacter pfennigii; type strain: DSM 3222; 36 Lineage=Root;rootrank;Bacteria;domain;Firmicutes;phylum;Clostridia;class;Clostridiales;order;Clostridiaceae 1;family;Oxobacter;genus	96.24	1.00E-173	610
3217	0	0	1	0	0	0	0	0	0	Firmicutes	Clostridia	Clostridiales	Clostridiaceae		S003611802 Oxobacter pfennigii; type strain: DSM 3222; 36 Lineage=Root;rootrank;Bacteria;domain;Firmicutes;phylum;Clostridia;class;Clostridiales;order;Clostridiaceae 1;family;Oxobacter;genus	94.64	4.00E-173	608
181	12	3	0	1	4	1	0	5	0	Firmicutes	Clostridia	Clostridiales	Clostridiaceae		S003613897 Clostridium perfringens; E108 Lineage=Root;rootrank;Bacteria;domain;Firmicutes;phylum;Clostridia;class;Clostridiales;order;Clostridiaceae 1;family;Clostridium sensu stricto;genus	99.73	0	676
2876	0	0	0	0	1	0	0	0	0	Firmicutes	Clostridia	Clostridiales	ClostridialesFamilyXI.IncertaeSedis	Clostridium	S001744320 Clostridium ultunense; DSM 10521 Lineage=Root;rootrank;Bacteria;domain;Firmicutes;phylum;Clostridia;class;Clostridiales;order;Incertae Sedis XI;family;Clostridium XII;genus	97.01	6.00E-176	617
991	0	0	0	0	0	1	0	0	0	Firmicutes	Clostridia	Clostridiales	ClostridialesFamilyXI.IncertaeSedis	Clostridium	S001744320 Clostridium ultunense; DSM 10521 Lineage=Root;rootrank;Bacteria;domain;Firmicutes;phylum;Clostridia;class;Clostridiales;order;Incertae Sedis XI;family;Clostridium XII;genus	93.81	3.00E-164	579
2142	2	0	0	0	1	0	0	0	0	Firmicutes	Clostridia	Clostridiales	ClostridialesFamilyXI.IncertaeSedis	Sedimentibacter	S000001447 Sedimentibacter hydroxybenzoicus (T); JW/Z-1 Lineage=Root;rootrank;Bacteria;domain;Firmicutes;phylum;Clostridia;class;Clostridiales;order;Clostridiales_Incertae Sedis XI;family;Sedimentibacter;genus	95.86	1.00E-167	590
1154	1	1	0	0	0	0	0	0	0	Firmicutes	Clostridia	Clostridiales	ClostridialesFamilyXI.IncertaeSedis	Sedimentibacter	S000277167 Sedimentibacter saalensis (T); ZF2 Lineage=Root;rootrank;Bacteria;domain;Firmicutes;phylum;Clostridia;class;Clostridiales;order;Clostridiales_Incertae Sedis XI;family;Sedimentibacter;genus	95.87	4.00E-178	625
60	0	0	0	0	0	1	0	0	0	Firmicutes	Clostridia	Clostridiales	ClostridialesFamilyXI.IncertaeSedis	Sedimentibacter	S000548193 Sedimentibacter hongkongensis; KI Lineage=Root;rootrank;Bacteria;domain;Firmicutes;phylum;Clostridia;class;Clostridiales;order;Clostridiales_Incertae Sedis XI;family;Sedimentibacter;genus	99.18	0	658
1469	0	0	0	0	0	0	0	1	0	Firmicutes	Clostridia	Clostridiales	ClostridialesFamilyXI.IncertaeSedis	Sporanaerobacter	S000260573 Clostridium filamentosum; DSM 6645 Lineage=Root;rootrank;Bacteria;domain;Firmicutes;phylum;Clostridia;class;Clostridiales;order;Clostridiales_Incertae Sedis XI;family;unclassified_Clostridiales_Incertae Sedis XI;	98.06	2.00E-180	632
749	0	0	0	0	0	1	0	0	0	Firmicutes	Clostridia	Clostridiales	ClostridialesFamilyXI.IncertaeSedis	Tepidimicrobium	S003285530 Tepidimicrobium xylanilyticum; CS-3-1 Lineage=Root;rootrank;Bacteria;domain;Firmicutes;phylum;Clostridia;class;Clostridiales;order;Clostridiales_Incertae Sedis XI;family;Tepidimicrobium;genus	99.74	0	713
2625	1	0	0	0	0	0	0	0	0	Firmicutes	Clostridia	Clostridiales	ClostridialesFamilyXI.IncertaeSedis	Tissierella	S002350819 Tissierella creatinini; type strain: DSM 9508; 2 Lineage=Root;rootrank;Bacteria;domain;Firmicutes;phylum;Clostridia;class;Clostridiales;order;Clostridiales_Incertae Sedis XI;family;Tissierella;genus	97.95	0	676
488	2	1	0	0	2	0	6	1	0	Firmicutes	Clostridia	Clostridiales	ClostridialesFamilyXI.IncertaeSedis	Tissierella	S003613876 Tissierella praeacuta; E064 Lineage=Root;rootrank;Bacteria;domain;Firmicutes;phylum;Clostridia;class;Clostridiales;order;Clostridiales_Incertae Sedis XI;family;Tissierella;genus	97	2.00E-175	616
1472	0	0	0	0	0	0	1	0	0	Firmicutes	Clostridia	Clostridiales	ClostridialesFamilyXI.IncertaeSedis	Tissierella	S003613876 Tissierella praeacuta; E064 Lineage=Root;rootrank;Bacteria;domain;Firmicutes;phylum;Clostridia;class;Clostridiales;order;Clostridiales_Incertae Sedis XI;family;Tissierella;genus	96.38	0	636
1286	0	0	0	0	1	0	0	0	0	Firmicutes	Clostridia	Clostridiales	ClostridialesFamilyXIII.IncertaeSedis	Eubacterium	S000260540 Clostridium aminobutyricum; DSM 2634 Lineage=Root;rootrank;Bacteria;domain;Firmicutes;phylum;Clostridia;class;Clostridiales;order;Clostridiales_Incertae Sedis XIII;family;Anaerovorax;genus	97.56	2.00E-180	632
1457	0	0	0	2	0	0	0	0	0	Firmicutes	Clostridia	Clostridiales	D2		S000391487 Gelria glutamica (T); TGO Lineage=Root;rootrank;Bacteria;domain;Firmicutes;phylum;Clostridia;class;Thermoanaerobacterales;order;Thermoanaerobacteraceae;family;Gelria;genus	89.4	3.00E-129	462
204	6	1	4	0	3	0	1	0	0	Firmicutes	Clostridia	Clostridiales	Dehalobacteriaceae	Dehalobacterium	S000721549 Gracilibacter thermotolerans; JW/YJL-S1; 5 Lineage=Root;rootrank;Bacteria;domain;Firmicutes;phylum;Clostridia;class;Clostridiales;order;Gracilibacteraceae;family;Gracilibacter;genus	91.42	1.00E-143	510
2163	4	0	1	0	1	0	0	0	0	Firmicutes	Clostridia	Clostridiales	Dehalobacteriaceae	Dehalobacterium	S000721549 Gracilibacter thermotolerans; JW/YJL-S1; 5 Lineage=Root;rootrank;Bacteria;domain;Firmicutes;phylum;Clostridia;class;Clostridiales;order;Gracilibacteraceae;family;Gracilibacter;genus	92.23	3.00E-149	529
560	0	0	0	1	0	0	0	0	0	Firmicutes	Clostridia	Clostridiales	Lachnospiraceae	Blautia	S000436018 Ruminococcus lactaris (T); ATCC 29176 Lineage=Root;rootrank;Bacteria;domain;Firmicutes;phylum;Clostridia;class;Clostridiales;order;Ruminococcaceae;family;Ruminococcus;genus	95.9	2.00E-180	632
1477	0	0	0	0	1	0	0	0	0	Firmicutes	Clostridia	Clostridiales	Lachnospiraceae	Clostridium	S000001447 Sedimentibacter hydroxybenzoicus (T); JW/Z-1 Lineage=Root;rootrank;Bacteria;domain;Firmicutes;phylum;Clostridia;class;Clostridiales;order;Clostridiales_Incertae Sedis XI;family;Sedimentibacter;genus	89.33	1.00E-122	440
511	0	0	0	1	0	0	0	0	0	Firmicutes	Clostridia	Clostridiales	Lachnospiraceae	Coprococcus	S000436018 Ruminococcus lactaris (T); ATCC 29176 Lineage=Root;rootrank;Bacteria;domain;Firmicutes;phylum;Clostridia;class;Clostridiales;order;Ruminococcaceae;family;Ruminococcus;genus	91.73	6.00E-146	518
2922	0	1	0	0	0	0	0	7	1	Firmicutes	Clostridia	Clostridiales	Lachnospiraceae	Epulopiscium	S003289060 Catabacter hongkongensis; 88678 Lineage=Root;rootrank;Bacteria;domain;Firmicutes;phylum;Clostridia;class;Clostridiales;order;unclassified_Clostridiales;	88.97	9.00E-135	481
1196	1	0	0	0	0	0	0	0	0	Firmicutes	Clostridia	Clostridiales	Lachnospiraceae		S000436018 Ruminococcus lactaris (T); ATCC 29176 Lineage=Root;rootrank;Bacteria;domain;Firmicutes;phylum;Clostridia;class;Clostridiales;order;Ruminococcaceae;family;Ruminococcus;genus	91.4	1.00E-142	507
1169	0	0	0	0	0	1	0	0	0	Firmicutes	Clostridia	Clostridiales	Lachnospiraceae		S000436018 Ruminococcus lactaris (T); ATCC 29176 Lineage=Root;rootrank;Bacteria;domain;Firmicutes;phylum;Clostridia;class;Clostridiales;order;Ruminococcaceae;family;Ruminococcus;genus	91.84	3.00E-154	545
2795	0	0	0	0	2	0	0	0	0	Firmicutes	Clostridia	Clostridiales	Lachnospiraceae		S000583264 Candidatus Clostridium timonensis; 1 Lineage=Root;rootrank;Bacteria;domain;Firmicutes;phylum;Clostridia;class;Clostridiales;order;unclassified_Clostridiales;	92.39	1.00E-157	556
1632	0	0	0	0	0	1	0	0	0	Firmicutes	Clostridia	Clostridiales	Lachnospiraceae		S000583264 Candidatus Clostridium timonensis; 1 Lineage=Root;rootrank;Bacteria;domain;Firmicutes;phylum;Clostridia;class;Clostridiales;order;unclassified_Clostridiales;	92.53	2.00E-156	553
1779	7	3	11	5	15	1	0	2	0	Firmicutes	Clostridia	Clostridiales	Lachnospiraceae		S000859891 Ruminococcus gauvreauii (T); CCRI-16110 Lineage=Root;rootrank;Bacteria;domain;Firmicutes;phylum;Clostridia;class;Clostridiales;order;Ruminococcaceae;family;Ruminococcus;genus	91.14	7.00E-151	534
2122	5	3	8	3	7	0	3	0	3	Firmicutes	Clostridia	Clostridiales	Lachnospiraceae		S000859891 Ruminococcus gauvreauii (T); CCRI-16110 Lineage=Root;rootrank;Bacteria;domain;Firmicutes;phylum;Clostridia;class;Clostridiales;order;Ruminococcaceae;family;Ruminococcus;genus	91.69	8.00E-145	514
2175	0	1	0	1	1	0	0	0	0	Firmicutes	Clostridia	Clostridiales	Lachnospiraceae		S000859891 Ruminococcus gauvreauii (T); CCRI-16110 Lineage=Root;rootrank;Bacteria;domain;Firmicutes;phylum;Clostridia;class;Clostridiales;order;Ruminococcaceae;family;Ruminococcus;genus	91.62	3.00E-154	545
1638	0	0	0	0	0	0	1	1	0	Firmicutes	Clostridia	Clostridiales	Lachnospiraceae		S000859891 Ruminococcus gauvreauii (T); CCRI-16110 Lineage=Root;rootrank;Bacteria;domain;Firmicutes;phylum;Clostridia;class;Clostridiales;order;Ruminococcaceae;family;Ruminococcus;genus	92.09	2.00E-155	549
2892	0	0	0	0	1	0	0	0	0	Firmicutes	Clostridia	Clostridiales	Lachnospiraceae		S000859891 Ruminococcus gauvreauii (T); CCRI-16110 Lineage=Root;rootrank;Bacteria;domain;Firmicutes;phylum;Clostridia;class;Clostridiales;order;Ruminococcaceae;family;Ruminococcus;genus	93.82	4.00E-158	558
571	0	0	0	0	0	1	0	0	0	Firmicutes	Clostridia	Clostridiales	Lachnospiraceae		S000859891 Ruminococcus gauvreauii (T); CCRI-16110 Lineage=Root;rootrank;Bacteria;domain;Firmicutes;phylum;Clostridia;class;Clostridiales;order;Ruminococcaceae;family;Ruminococcus;genus	93.28	8.00E-155	547
853	0	0	0	0	0	0	0	0	1	Firmicutes	Clostridia	Clostridiales	Lachnospiraceae		S001587408 Christensenella minuta; YIT 12065 Lineage=Root;rootrank;Bacteria;domain;Firmicutes;phylum;Clostridia;class;Clostridiales;order;unclassified_Clostridiales;	89.29	5.00E-137	488
1067	0	0	0	3	4	0	0	0	0	Firmicutes	Clostridia	Clostridiales	Lachnospiraceae		S001933336 Candidatus Methylomirabilis oxyfera Lineage=Root;rootrank;Bacteria;domain;unclassified_Bacteria;	84.5	3.00E-104	379
1113	0	0	0	0	0	0	0	0	1	Firmicutes	Clostridia	Clostridiales	Peptococcaceae		S002291168 Syntrophomonas wolfei (T); Goettingen Lineage=Root;rootrank;Bacteria;domain;Firmicutes;phylum;Clostridia;class;Clostridiales;order;Syntrophomonadaceae;family;Syntrophomonas;genus	90.37	5.00E-137	488
1812	0	0	0	1	0	0	0	0	0	Firmicutes	Clostridia	Clostridiales	Peptococcaceae		S002441012 Clostridium cellobioparum; JCM 1422 Lineage=Root;rootrank;Bacteria;domain;Firmicutes;phylum;Clostridia;class;Clostridiales;order;Ruminococcaceae;family;Clostridium III;genus	91.73	2.00E-145	516
2254	1	0	1	0	0	0	0	0	0	Firmicutes	Clostridia	Clostridiales	Peptococcaceae	Dehalobacter	S000116229 chimeric sequence SJA-7 Lineage=Root;rootrank;Bacteria;domain;Firmicutes;phylum;Clostridia;class;Clostridiales;order;Peptococcaceae 1;family;Dehalobacter;genus	98.72	0	691
619	0	0	0	0	0	0	1	0	0	Firmicutes	Clostridia	Clostridiales	Peptococcaceae	Dehalobacter	S002355505 Syntrophobotulus glycolicus DSM 8271 (T) Lineage=Root;rootrank;Bacteria;domain;Firmicutes;phylum;Clostridia;class;Clostridiales;order;Peptococcaceae 1;family;Syntrophobotulus;genus	96.92	0	654
3124	0	0	0	2	2	0	0	0	0	Firmicutes	Clostridia	Clostridiales	Peptococcaceae	Desulfitobacterium	S003312533 Paenibacillus wynnii; Hgh02-S4 Lineage=Root;rootrank;Bacteria;domain;Firmicutes;phylum;Bacilli;class;Bacillales;order;Paenibacillaceae 1;family;Paenibacillus;genus	91.62	5.00E-142	505
49	0	0	0	2	1	0	1	0	0	Firmicutes	Clostridia	Clostridiales	Peptococcaceae	Desulfosporosinus	S002355505 Syntrophobotulus glycolicus DSM 8271 (T) Lineage=Root;rootrank;Bacteria;domain;Firmicutes;phylum;Clostridia;class;Clostridiales;order;Peptococcaceae 1;family;Syntrophobotulus;genus	93.82	4.00E-158	558
1504	0	0	0	1	0	0	0	0	0	Firmicutes	Clostridia	Clostridiales	Peptococcaceae	Desulfotomaculum	S000116588 Sporotomaculum syntrophicum (T); FB Lineage=Root;rootrank;Bacteria;domain;Firmicutes;phylum;Clostridia;class;Clostridiales;order;Peptococcaceae 2;family;Sporotomaculum;genus	95.7	8.00E-170	597
984	0	0	0	1	0	0	0	0	0	Firmicutes	Clostridia	Clostridiales	Peptococcaceae	Desulfotomaculum	S000126672 Sporotomaculum hydroxybenzoicum (T); BT, DSM 5475, type strain Lineage=Root;rootrank;Bacteria;domain;Firmicutes;phylum;Clostridia;class;Clostridiales;order;Peptococcaceae 2;family;Sporotomaculum;genus	96.77	5.00E-177	621
2176	0	0	0	0	0	0	0	1	0	Firmicutes	Clostridia	Clostridiales	Peptococcaceae	Desulfotomaculum	S000366775 Desulfotomaculum geothermicum; type strain: B2 Lineage=Root;rootrank;Bacteria;domain;Firmicutes;phylum;Clostridia;class;Clostridiales;order;Peptococcaceae 2;family;Desulfotomaculum;genus	95.43	4.00E-168	592
1912	0	0	0	0	0	0	0	1	0	Firmicutes	Clostridia	Clostridiales	Peptococcaceae	Desulfotomaculum	S000485862 Desulfotomaculum alcoholivorax (T); RE35E1 Lineage=Root;rootrank;Bacteria;domain;Firmicutes;phylum;Clostridia;class;Clostridiales;order;Peptococcaceae 2;family;Desulfotomaculum;genus	92.02	1.00E-147	523
82	0	0	0	1	0	0	0	0	0	Firmicutes	Clostridia	Clostridiales	Peptococcaceae	Desulfotomaculum	S001240925 Desulfotomaculum nigrificans; CINBIN-C2 Lineage=Root;rootrank;Bacteria;domain;Firmicutes;phylum;Clostridia;class;Clostridiales;order;Peptococcaceae 2;family;Desulfotomaculum;genus	98.12	0	649
3246	0	1	0	0	0	0	0	0	0	Firmicutes	Clostridia	Clostridiales	Peptococcaceae	Desulfotomaculum	S002291093 Desulfotomaculum reducens MI-1 Lineage=Root;rootrank;Bacteria;domain;Firmicutes;phylum;Clostridia;class;Clostridiales;order;Peptococcaceae 2;family;Desulfotomaculum;genus	97.71	0	673
2371	1	3	3	0	1	0	0	0	0	Firmicutes	Clostridia	Clostridiales	Peptococcaceae	Pelotomaculum	S000262080 Pelotomaculum propionicicum (T); MGP Lineage=Root;rootrank;Bacteria;domain;Firmicutes;phylum;Clostridia;class;Clostridiales;order;Peptococcaceae 2;family;Pelotomaculum;genus	95.98	5.00E-172	604
25	0	0	0	0	1	0	0	0	0	Firmicutes	Clostridia	Clostridiales	Peptococcaceae	Pelotomaculum	S000569921 Pelotomaculum isophthalicicum (T); JI Lineage=Root;rootrank;Bacteria;domain;Firmicutes;phylum;Clostridia;class;Clostridiales;order;Peptococcaceae 2;family;Pelotomaculum;genus	96.51	2.00E-175	616
576	0	0	0	1	0	0	0	0	0	Firmicutes	Clostridia	Clostridiales	Peptococcaceae	Thermincola	S000262080 Pelotomaculum propionicicum (T); MGP Lineage=Root;rootrank;Bacteria;domain;Firmicutes;phylum;Clostridia;class;Clostridiales;order;Peptococcaceae 2;family;Pelotomaculum;genus	90.32	5.00E-137	488
2463	0	1	0	0	0	0	1	1	1	Firmicutes	Clostridia	Clostridiales	Peptococcaceae	Thermincola	S002165556 Thermincola potens JR Lineage=Root;rootrank;Bacteria;domain;Firmicutes;phylum;Clostridia;class;Clostridiales;order;Peptococcaceae 1;family;Thermincola;genus	93.82	1.00E-158	560
50	0	0	0	1	0	0	0	0	0	Firmicutes	Clostridia	Clostridiales	Peptococcaceae	Thermincola	S002165556 Thermincola potens JR Lineage=Root;rootrank;Bacteria;domain;Firmicutes;phylum;Clostridia;class;Clostridiales;order;Peptococcaceae 1;family;Thermincola;genus	91.67	8.00E-145	514
2612	0	0	0	0	0	0	0	1	0	Firmicutes	Clostridia	Clostridiales	Peptococcaceae	Thermincola	S002165556 Thermincola potens JR Lineage=Root;rootrank;Bacteria;domain;Firmicutes;phylum;Clostridia;class;Clostridiales;order;Peptococcaceae 1;family;Thermincola;genus	91.4	1.00E-143	510
275	0	0	0	0	1	0	0	0	0	Firmicutes	Clostridia	Clostridiales	Peptococcaceae		S000544828 Vulcanibacillus modesticaldus (T); type strain: BR Lineage=Root;rootrank;Bacteria;domain;Firmicutes;phylum;Bacilli;class;Bacillales;order;Bacillaceae 2;family;Vulcanibacillus;genus	92.39	1.00E-157	556
2281	0	0	0	0	0	0	1	0	0	Firmicutes	Clostridia	Clostridiales	Peptococcaceae		S002291168 Syntrophomonas wolfei (T); Goettingen Lineage=Root;rootrank;Bacteria;domain;Firmicutes;phylum;Clostridia;class;Clostridiales;order;Syntrophomonadaceae;family;Syntrophomonas;genus	91.09	3.00E-149	529
2564	4	1	6	2	2	3	0	0	3	Firmicutes	Clostridia	Clostridiales	Peptococcaceae		S002355505 Syntrophobotulus glycolicus DSM 8271 (T) Lineage=Root;rootrank;Bacteria;domain;Firmicutes;phylum;Clostridia;class;Clostridiales;order;Peptococcaceae 1;family;Syntrophobotulus;genus	97.04	1.00E-177	623
894	1	0	1	0	0	0	0	0	0	Firmicutes	Clostridia	Clostridiales	Peptococcaceae		S002355505 Syntrophobotulus glycolicus DSM 8271 (T) Lineage=Root;rootrank;Bacteria;domain;Firmicutes;phylum;Clostridia;class;Clostridiales;order;Peptococcaceae 1;family;Syntrophobotulus;genus	97.44	0	667
634	0	0	1	0	0	0	0	0	0	Firmicutes	Clostridia	Clostridiales	Peptococcaceae		S002355505 Syntrophobotulus glycolicus DSM 8271 (T) Lineage=Root;rootrank;Bacteria;domain;Firmicutes;phylum;Clostridia;class;Clostridiales;order;Peptococcaceae 1;family;Syntrophobotulus;genus	94.92	3.00E-174	612

2360	0	0	0	0	1	0	0	0	0	Firmicutes	Clostridia	Clostridiales	Peptococcaceae		S002355505 Syntrophobotulus glycolicus DSM 8271 (T) Lineage=Root;rootrank;Bacteria;domain;Firmicutes;phylum;Clostridia;class;Clostridiales;order;Peptococcaceae 1;family;Syntrophobotulus;genus	93.11	5.00E-162	571
2339	3	1	0	2	2	0	0	0	0	Firmicutes	Clostridia	Clostridiales	Peptostreptococcaceae		S002953940 [Clostridium] ghonii; 2447_6 Lineage=Root;rootrank;Bacteria;domain;Firmicutes;phylum;Clostridia;class;Clostridiales;order;Peptostreptococcaceae;family;Clostridium XI;genus	100	0	682
2388	2	0	5	0	0	0	0	0	0	Firmicutes	Clostridia	Clostridiales	Ruminococcaceae		S000000835 Sporobacter termitidis (T); SYR Lineage=Root;rootrank;Bacteria;domain;Firmicutes;phylum;Clostridia;class;Clostridiales;order;Ruminococcaceae;family;Sporobacter;genus	96.77	5.00E-177	621
2965	0	0	0	0	1	0	0	0	0	Firmicutes	Clostridia	Clostridiales	Ruminococcaceae		S000000835 Sporobacter termitidis (T); SYR Lineage=Root;rootrank;Bacteria;domain;Firmicutes;phylum;Clostridia;class;Clostridiales;order;Ruminococcaceae;family;Sporobacter;genus	95.92	8.00E-180	630
2037	0	0	0	0	0	0	0	0	1	Firmicutes	Clostridia	Clostridiales	Ruminococcaceae		S000000835 Sporobacter termitidis (T); SYR Lineage=Root;rootrank;Bacteria;domain;Firmicutes;phylum;Clostridia;class;Clostridiales;order;Ruminococcaceae;family;Sporobacter;genus	92.47	2.00E-150	532
496	0	0	1	0	0	0	0	0	0	Firmicutes	Clostridia	Clostridiales	Ruminococcaceae		S000008119 Clostridium viride (T); T2-7 (DSM 6836) Lineage=Root;rootrank;Bacteria;domain;Firmicutes;phylum;Clostridia;class;Clostridiales;order;Ruminococcaceae;family;Clostridium IV;genus	93.3	2.00E-155	549
3064	1	0	0	0	0	0	0	0	0	Firmicutes	Clostridia	Clostridiales	Ruminococcaceae		S000388430 Papillibacter cinnamivorans (T); CIN1; DSM12816 Lineage=Root;rootrank;Bacteria;domain;Firmicutes;phylum;Clostridia;class;Clostridiales;order;Ruminococcaceae;family;Papillibacter;genus	94.36	7.00E-171	601
1173	0	0	0	0	0	1	0	0	0	Firmicutes	Clostridia	Clostridiales	Ruminococcaceae		S000388430 Papillibacter cinnamivorans (T); CIN1; DSM12816 Lineage=Root;rootrank;Bacteria;domain;Firmicutes;phylum;Clostridia;class;Clostridiales;order;Ruminococcaceae;family;Papillibacter;genus	92.84	2.00E-160	566
941	0	0	0	0	1	0	0	0	0	Firmicutes	Clostridia	Clostridiales	Ruminococcaceae		S000750004 Ruminococcus albus; 8 Lineage=Root;rootrank;Bacteria;domain;Firmicutes;phylum;Clostridia;class;Clostridiales;order;Ruminococcaceae;family;Ruminococcus;genus	92.82	2.00E-160	566
254	0	0	0	0	0	1	0	0	0	Firmicutes	Clostridia	Clostridiales	Ruminococcaceae		S003277052 Flavonifractor plautii; MT42 Lineage=Root;rootrank;Bacteria;domain;Firmicutes;phylum;Clostridia;class;Clostridiales;order;Ruminococcaceae;family;Flavonifractor;genus	93.88	7.00E-166	584
709	1	0	0	0	0	0	0	0	0	Firmicutes	Clostridia	Clostridiales	Ruminococcaceae		S003292132 Acetivibrio cellulolyticus; HPc Lineage=Root;rootrank;Bacteria;domain;Firmicutes;phylum;Clostridia;class;Clostridiales;order;Ruminococcaceae;family;Clostridium III;genus	88.55	5.00E-132	472
2441	1	0	0	1	1	0	0	0	0	Firmicutes	Clostridia	Clostridiales	Ruminococcaceae	Acetivibrio	S000001965 Clostridium thermosuccinogenes (T); DSM 5807 Lineage=Root;rootrank;Bacteria;domain;Firmicutes;phylum;Clostridia;class;Clostridiales;order;Ruminococcaceae;family;Clostridium III;genus	96.94	0	656
2073	0	0	0	0	2	0	0	0	0	Firmicutes	Clostridia	Clostridiales	Ruminococcaceae	Acetivibrio	S000001965 Clostridium thermosuccinogenes (T); DSM 5807 Lineage=Root;rootrank;Bacteria;domain;Firmicutes;phylum;Clostridia;class;Clostridiales;order;Ruminococcaceae;family;Clostridium III;genus	95.4	1.00E-177	623
2562	1	0	1	1	0	0	0	0	0	Firmicutes	Clostridia	Clostridiales	Ruminococcaceae	Acetivibrio	S000014604 Clostridium aldrichii (T); DSM 6159 Lineage=Root;rootrank;Bacteria;domain;Firmicutes;phylum;Clostridia;class;Clostridiales;order;Ruminococcaceae;family;Clostridium III;genus	94.64	1.00E-172	606
946	0	1	0	0	0	0	0	0	0	Firmicutes	Clostridia	Clostridiales	Ruminococcaceae	Acetivibrio	S000941547 Clostridium sufflavum (T); CDT-1 Lineage=Root;rootrank;Bacteria;domain;Firmicutes;phylum;Clostridia;class;Clostridiales;order;Ruminococcaceae;family;Clostridium III;genus	95.94	0	638
2602	0	0	0	0	0	0	0	1	0	Firmicutes	Clostridia	Clostridiales	Ruminococcaceae	Acetivibrio	S003261783 Clostridium josui; JCM 17888 Lineage=Root;rootrank;Bacteria;domain;Firmicutes;phylum;Clostridia;class;Clostridiales;order;Ruminococcaceae;family;Clostridium III;genus	96.68	0	651
982	23	2	3	2	4	2	0	3	2	Firmicutes	Clostridia	Clostridiales	Ruminococcaceae	Acetivibrio	S003292132 Acetivibrio cellulolyticus; HPc Lineage=Root;rootrank;Bacteria;domain;Firmicutes;phylum;Clostridia;class;Clostridiales;order;Ruminococcaceae;family;Clostridium III;genus	98.92	0	665
2408	1	0	0	0	0	0	0	0	0	Firmicutes	Clostridia	Clostridiales	Ruminococcaceae	Acetivibrio	S003292132 Acetivibrio cellulolyticus; HPc Lineage=Root;rootrank;Bacteria;domain;Firmicutes;phylum;Clostridia;class;Clostridiales;order;Ruminococcaceae;family;Clostridium III;genus	96.93	0	656
948	0	1	0	0	0	0	0	0	0	Firmicutes	Clostridia	Clostridiales	Ruminococcaceae	Acetivibrio	S003723521 Clostridium thermocellum; mbf-VZ-096; BC1 Lineage=Root;rootrank;Bacteria;domain;Firmicutes;phylum;Clostridia;class;Clostridiales;order;Ruminococcaceae;family;Clostridium III;genus	97.45	0	669
2678	0	1	0	1	0	0	0	0	0	Firmicutes	Clostridia	Clostridiales	Ruminococcaceae	Ethanoligenens	S001243254 Clostridium cellulosi; D3 Lineage=Root;rootrank;Bacteria;domain;Firmicutes;phylum;Clostridia;class;Clostridiales;order;Ruminococcaceae;family;Clostridium IV;genus	94.13	3.00E-169	595
732	1	1	0	0	0	0	0	0	0	Firmicutes	Clostridia	Clostridiales	Ruminococcaceae		S000388430 Papillibacter cinnamivorans (T); CIN1; DSM12816 Lineage=Root;rootrank;Bacteria;domain;Firmicutes;phylum;Clostridia;class;Clostridiales;order;Ruminococcaceae;family;Papillibacter;genus	92.58	9.00E-160	564
2827	0	0	1	0	0	1	0	0	0	Firmicutes	Clostridia	Clostridiales	Ruminococcaceae		S000414642 Bacteroides cellulosolvens Lineage=Root;rootrank;Bacteria;domain;Firmicutes;phylum;Clostridia;class;Clostridiales;order;Ruminococcaceae;family;Clostridium III;genus	96.16	0	641
698	0	0	0	1	0	0	0	0	0	Firmicutes	Clostridia	Clostridiales	Ruminococcaceae		S000439515 Clostridium straminisolvens (T); CSK1 Lineage=Root;rootrank;Bacteria;domain;Firmicutes;phylum;Clostridia;class;Clostridiales;order;Ruminococcaceae;family;Clostridium III;genus	95.14	7.00E-176	617
659	0	0	1	0	0	0	0	0	0	Firmicutes	Clostridia	Clostridiales	Ruminococcaceae		S000541382 Anaerotruncus colihominis; HKU19 Lineage=Root;rootrank;Bacteria;domain;Firmicutes;phylum;Clostridia;class;Clostridiales;order;Ruminococcaceae;family;Anaerotruncus;genus	95.42	5.00E-177	621
459	0	0	1	0	0	0	0	0	0	Firmicutes	Clostridia	Clostridiales	Ruminococcaceae		S000750004 Ruminococcus albus; 8 Lineage=Root;rootrank;Bacteria;domain;Firmicutes;phylum;Clostridia;class;Clostridiales;order;Ruminococcaceae;family;Ruminococcus;genus	93.53	2.00E-156	553
1510	1	0	0	0	0	0	0	0	0	Firmicutes	Clostridia	Clostridiales	Sulfobacillaceae		S001152076 Thermaerobacter composti (T); Ni80 Lineage=Root;rootrank;Bacteria;domain;Firmicutes;phylum;Clostridia;class;Clostridiales;order;Clostridiales_Incertae Sedis XVII;family;Thermaerobacter;genus	90.08	1.00E-142	507
726	0	0	0	0	2	0	0	0	0	Firmicutes	Clostridia	Clostridiales	Sulfobacillaceae		S001152076 Thermaerobacter composti (T); Ni80 Lineage=Root;rootrank;Bacteria;domain;Firmicutes;phylum;Clostridia;class;Clostridiales;order;Clostridiales_Incertae Sedis XVII;family;Thermaerobacter;genus	90.59	7.00E-146	518
2247	0	1	0	0	0	0	0	0	0	Firmicutes	Clostridia	Clostridiales	Sulfobacillaceae		S001152076 Thermaerobacter composti (T); Ni80 Lineage=Root;rootrank;Bacteria;domain;Firmicutes;phylum;Clostridia;class;Clostridiales;order;Clostridiales_Incertae Sedis XVII;family;Thermaerobacter;genus	89.25	8.00E-130	464
2505	0	0	0	0	0	0	1	0	0	Firmicutes	Clostridia	Clostridiales	Sulfobacillaceae		S001152076 Thermaerobacter composti (T); Ni80 Lineage=Root;rootrank;Bacteria;domain;Firmicutes;phylum;Clostridia;class;Clostridiales;order;Clostridiales_Incertae Sedis XVII;family;Thermaerobacter;genus	91.22	5.00E-142	505
2943	0	0	0	0	0	0	0	0	1	Firmicutes	Clostridia	Clostridiales	Sulfobacillaceae		S002350110 Thermaerobacter marianensis DSM 12885 Lineage=Root;rootrank;Bacteria;domain;Firmicutes;phylum;Clostridia;class;Clostridiales;order;Clostridiales_Incertae Sedis XVII;family;Thermaerobacter;genus	91.4	1.00E-143	510
2480	0	0	0	0	0	0	0	0	1	Firmicutes	Clostridia	Clostridiales	Sulfobacillaceae		S003286846 Moorella thermoacetica; Y73 Lineage=Root;rootrank;Bacteria;domain;Firmicutes;phylum;Clostridia;class;Thermoanaerobacterales;order;Thermoanaerobacteraceae;family;Moorella;genus	92.2	1.00E-148	527
1273	0	0	0	1	0	0	0	0	0	Firmicutes	Clostridia	Clostridiales	Sulfobacillaceae	Thermaerobacter	S002350110 Thermaerobacter marianensis DSM 12885 Lineage=Root;rootrank;Bacteria;domain;Firmicutes;phylum;Clostridia;class;Clostridiales;order;Clostridiales_Incertae Sedis XVII;family;Thermaerobacter;genus	99.46	0	676
272	0	0	0	0	0	1	0	0	0	Firmicutes	Clostridia	Clostridiales	Sulfobacillaceae	Thermaerobacter	S002350110 Thermaerobacter marianensis DSM 12885 Lineage=Root;rootrank;Bacteria;domain;Firmicutes;phylum;Clostridia;class;Clostridiales;order;Clostridiales_Incertae Sedis XVII;family;Thermaerobacter;genus	91.49	1.00E-143	510
1753	0	0	0	0	0	0	0	0	1	Firmicutes	Clostridia	Clostridiales	Sulfobacillaceae	Thermaerobacter	S002350110 Thermaerobacter marianensis DSM 12885 Lineage=Root;rootrank;Bacteria;domain;Firmicutes;phylum;Clostridia;class;Clostridiales;order;Clostridiales_Incertae Sedis XVII;family;Thermaerobacter;genus	92.07	7.00E-156	551
3000	1	3	0	0	0	0	1	0	0	Firmicutes	Clostridia	Clostridiales	Sulfobacillaceae		S000262080 Pelotomaculum propionicicum (T); MGP Lineage=Root;rootrank;Bacteria;domain;Firmicutes;phylum;Clostridia;class;Clostridiales;order;Peptococcaceae 2;family;Pelotomaculum;genus	89.82	2.00E-141	503
1028	0	0	0	0	0	0	0	0	1	Firmicutes	Clostridia	Clostridiales	Sulfobacillaceae		S000642543 Calditerricola satsumensis (T); YMO81 Lineage=Root;rootrank;Bacteria;domain;Firmicutes;phylum;Bacilli;class;Bacillales;order;Bacillales_incertae_sedis;family;Calditerricola;genus	85.5	6.00E-112	405
936	1	0	0	0	0	0	0	0	0	Firmicutes	Clostridia	Clostridiales	Sulfobacillaceae		S000752077 Moorella perchloratireducens; An10 Lineage=Root;rootrank;Bacteria;domain;Firmicutes;phylum;Clostridia;class;Thermoanaerobacterales;order;Thermoanaerobacteraceae;family;Moorella;genus	89.29	1.00E-137	490
3266	0	0	1	0	0	0	0	0	0	Firmicutes	Clostridia	Clostridiales	Sulfobacillaceae		S000752077 Moorella perchloratireducens; An10 Lineage=Root;rootrank;Bacteria;domain;Firmicutes;phylum;Clostridia;class;Thermoanaerobacterales;order;Thermoanaerobacteraceae;family;Moorella;genus	90.11	6.00E-136	484
2980	1	11	4	0	1	1	1	1	0	Firmicutes	Clostridia	Clostridiales	Sulfobacillaceae		S003286846 Moorella thermoacetica; Y73 Lineage=Root;rootrank;Bacteria;domain;Firmicutes;phylum;Clostridia;class;Thermoanaerobacterales;order;Thermoanaerobacteraceae;family;Moorella;genus	87.63	3.00E-119	429
58	0	0	1	0	0	2	0	0	0	Firmicutes	Clostridia	Clostridiales	Symbiobacteriaceae		S000002651 Desulfotomaculum sapomandens (T); DSM 3223 Lineage=Root;rootrank;Bacteria;domain;Firmicutes;phylum;Clostridia;class;Clostridiales;order;Peptococcaceae 2;family;Desulfotomaculum;genus	87.79	1.00E-128	460
3070	0	0	0	0	0	0	0	1	1	Firmicutes	Clostridia	Clostridiales	Symbiobacteriaceae		S000925530 Candidatus Helioclostridium ananthapuram; JHB3 Lineage=Root;rootrank;Bacteria;domain;Firmicutes;phylum;Clostridia;class;Clostridiales;order;Heliobacteriaceae;family;unclassified_Heliobacteriaceae;	86.77	2.00E-121	436
2029	0	0	0	0	0	0	0	1	0	Firmicutes	Clostridia	Clostridiales	Symbiobacteriaceae		S000925530 Candidatus Helioclostridium ananthapuram; JHB3 Lineage=Root;rootrank;Bacteria;domain;Firmicutes;phylum;Clostridia;class;Clostridiales;order;Heliobacteriaceae;family;unclassified_Heliobacteriaceae;	86.17	2.00E-111	403
1806	5	3	3	3	5	2	2	1	3	Firmicutes	Clostridia	Clostridiales	Symbiobacteriaceae	Symbiobacterium	S000870713 Calditerricola yamamurae (T); YMO722 Lineage=Root;rootrank;Bacteria;domain;Firmicutes;phylum;Bacilli;class;Bacillales;order;Bacillales_incertae_sedis;family;Calditerricola;genus	87.17	2.00E-116	420
2120	0	3	2	3	5	1	1	1	1	Firmicutes	Clostridia	Clostridiales	Symbiobacteriaceae		S000002651 Desulfotomaculum sapomandens (T); DSM 3223 Lineage=Root;rootrank;Bacteria;domain;Firmicutes;phylum;Clostridia;class;Clostridiales;order;Peptococcaceae 2;family;Desulfotomaculum;genus	87.76	4.00E-128	459
707	0	0	1	0	0	0	1	0	0	Firmicutes	Clostridia	Clostridiales	Symbiobacteriaceae		S000002651 Desulfotomaculum sapomandens (T); DSM 3223 Lineage=Root;rootrank;Bacteria;domain;Firmicutes;phylum;Clostridia;class;Clostridiales;order;Peptococcaceae 2;family;Desulfotomaculum;genus	87.73	7.00E-121	435
2841	0	0	0	0	0	0	2	0	0	Firmicutes	Clostridia	Clostridiales	Symbiobacteriaceae		S000002651 Desulfotomaculum sapomandens (T); DSM 3223 Lineage=Root;rootrank;Bacteria;domain;Firmicutes;phylum;Clostridia;class;Clostridiales;order;Peptococcaceae 2;family;Desulfotomaculum;genus	86.26	4.00E-118	425
705	0	0	0	0	0	0	0	1	0	Firmicutes	Clostridia	Clostridiales	Symbiobacteriaceae		S000002651 Desulfotomaculum sapomandens (T); DSM 3223 Lineage=Root;rootrank;Bacteria;domain;Firmicutes;phylum;Clostridia;class;Clostridiales;order;Peptococcaceae 2;family;Desulfotomaculum;genus	87.24	9.00E-125	448
1191	0	0	1	1	0	2	0	2	0	Firmicutes	Clostridia	Clostridiales	Symbiobacteriaceae		S000366775 Desulfotomaculum geothermicum; type strain: B2 Lineage=Root;rootrank;Bacteria;domain;Firmicutes;phylum;Clostridia;class;Clostridiales;order;Peptococcaceae 2;family;Desulfotomaculum;genus	87.77	9.00E-120	431
2476	0	0	2	0	1	0	0	0	1	Firmicutes	Clostridia	Clostridiales	Symbiobacteriaceae		S000391487 Gelria glutamica (T); TGO Lineage=Root;rootrank;Bacteria;domain;Firmicutes;phylum;Clostridia;class;Thermoanaerobacterales;order;Thermoanaerobacteraceae;family;Gelria;genus	87.4	1.00E-118	427
1031	0	0	0	0	0	1	2	0	0	Firmicutes	Clostridia	Clostridiales	Symbiobacteriaceae		S000569921 Pelotomaculum isophthalicicum (T); JI Lineage=Root;rootrank;Bacteria;domain;Firmicutes;phylum;Clostridia;class;Clostridiales;order;Peptococcaceae 2;family;Pelotomaculum;genus	87.76	5.00E-127	455
2885	0	0	0	1	1	0	0	0	0	Firmicutes	Clostridia	Clostridiales	Symbiobacteriaceae		S000569921 Pelotomaculum isophthalicicum (T); JI Lineage=Root;rootrank;Bacteria;domain;Firmicutes;phylum;Clostridia;class;Clostridiales;order;Peptococcaceae 2;family;Pelotomaculum;genus	87.72	2.00E-127	457
2489	0	0	0	0	0	0	0	1	1	Firmicutes	Clostridia	Clostridiales	Symbiobacteriaceae		S000569921 Pelotomaculum isophthalicicum (T); JI Lineage=Root;rootrank;Bacteria;domain;Firmicutes;phylum;Clostridia;class;Clostridiales;order;Peptococcaceae 2;family;Pelotomaculum;genus	86.45	3.00E-119	429
1318	0	0	0	0	0	0	0	0	2	Firmicutes	Clostridia	Clostridiales	Symbiobacteriaceae		S000569921 Pelotomaculum isophthalicicum (T); JI Lineage=Root;rootrank;Bacteria;domain;Firmicutes;phylum;Clostridia;class;Clostridiales;order;Peptococcaceae 2;family;Pelotomaculum;genus	87.4	1.00E-118	427
593	0	0	0	0	0	0	0	0	1	Firmicutes	Clostridia	Clostridiales	Symbiobacteriaceae		S000569921 Pelotomaculum isophthalicicum (T); JI Lineage=Root;rootrank;Bacteria;domain;Firmicutes;phylum;Clostridia;class;Clostridiales;order;Peptococcaceae 2;family;Pelotomaculum;genus	88.3	7.00E-131	468
1689	0	0	0	0	0	0	0	1	0	Firmicutes	Clostridia	Clostridiales	Symbiobacteriaceae		S000858632 Pelotomaculum thermopropionicum SI Lineage=Root;rootrank;Bacteria;domain;Firmicutes;phylum;Clostridia;class;Clostridiales;order;Peptococcaceae 2;family;Pelotomaculum;genus	86.13	2.00E-110	399
1908	0	0	0	1	0	0	0	0	0	Firmicutes	Clostridia	Clostridiales	Symbiobacteriaceae		S003257829 Heliophilum fasciatum; type strain: DSM 11170; 3 Lineage=Root;rootrank;Bacteria;domain;Firmicutes;phylum;Clostridia;class;Clostridiales;order;Heliobacteriaceae;family;Heliophilum;genus	86.51	3.00E-119	429
2569	0	2	0	0	1	0	0	0	1	Firmicutes	Clostridia	Clostridiales	Syntrophomonadaceae		S003259712 Syntrophothermus lipocalidus DSM 12680; TGB-C1 Lineage=Root;rootrank;Bacteria;domain;Firmicutes;phylum;Clostridia;class;Clostridiales;order;Syntrophomonadaceae;family;Syntrophothermus;genus	95.93	0	634
1517	0	1	0	0	0	0	0	0	0	Firmicutes	Clostridia	Clostridiales	Syntrophomonadaceae	Syntrophomonas	S000357627 Syntrophomonas curvata (T); GB8-1 Lineage=Root;rootrank;Bacteria;domain;Firmicutes;phylum;Clostridia;class;Clostridiales;order;Syntrophomonadaceae;family;Syntrophomonas;genus	97.59	0	638
1861	0	1	1	0	0	0	0	0	0	Firmicutes	Clostridia	Clostridiales	Syntrophomonadaceae	Syntrophomonas	S003277703 Syntrophomonas bryantii; type strain: DSM 3014 Lineage=Root;rootrank;Bacteria;domain;Firmicutes;phylum;Clostridia;class;Clostridiales;order;Syntrophomonadaceae;family;Syntrophomonas;genus	97.04	4.00E-178	625
627	0	0	0	0	0	0	0	0	1	Firmicutes	Clostridia	Clostridiales	Veillonellaceae	Desulfosporomusa	S000112718 Acetonema longum (T); DSM 6540(T) Lineage=Root;rootrank;Bacteria;domain;Firmicutes;phylum;Negativicutes;class;Selenomonadales;order;Veillonellaceae;family;Acetonema;genus	99.46	0	676
2576	1	0	0	1	0	0	0	1	0	Firmicutes	Clostridia	Clostridiales	Veillonellaceae	Desulfosporomusa	S002350802 Sporomusa paucivorans; type strain: DSM 3697; 1 Lineage=Root;rootrank;Bacteria;domain;Firmicutes;phylum;Negativicutes;class;Selenomonadales;order;Veillonellaceae;family;Sporomusa;genus	98.65	0	656
205	0	0	0	0	0	0	0	1	0	Firmicutes	Clostridia	Clostridiales	Veillonellaceae	Desulfosporomusa	S003288216 Sporomusa malonica; DSM 5090; K31 Lineage=Root;rootrank;Bacteria;domain;Firmicutes;phylum;Negativicutes;class;Selenomonadales;order;Veillonellaceae;family;Sporomusa;genus	98.39	0	654
2580	1	0	0	0	0	0	0	1	0	Firmicutes	Clostridia	Clostridiales	Veillonellaceae	Sporotalea	S002157014 Sporotalea propionica; TM1 Lineage=Root;rootrank;Bacteria;domain;Firmicutes;phylum;Negativicutes;class;Selenomonadales;order;Veillonellaceae;family;unclassified_Veillonellaceae;	99.46	0	675
1381	0	0	0	0	0	0	1	0	0	Firmicutes	Clostridia	Clostridiales	Veillonellaceae		S000769575 Thiohalomonas denitrificans; HLD 14 Lineage=Root;rootrank;Bacteria;domain;"Proteobacteria";phylum;Gammaproteobacteria;class;Gammaproteobacteria_incertae_sedis;order;Thiohalomonas;genus	87.1	2.00E-116	420
1935	0	0	0	0	0	0	0	1	0	Firmicutes	Clostridia	Clostridiales	Veillonellaceae		S000769575 Thiohalomonas denitrificans; HLD 14 Lineage=Root;rootrank;Bacteria;domain;"Proteobacteria";phylum;Gammaproteobacteria;class;Gammaproteobacteria_incertae_sedis;order;Thiohalomonas;genus	88.92	1.00E-127	457
2109	0	0	1	0	0	0	0	0	0	Firmicutes	Clostridia	Clostridiales	Veillonellaceae		S001198079 Thioprofundum lithotrophicum (T); 106 Lineage=Root;rootrank;Bacteria;domain;"Proteobacteria";phylum;Gammaproteobacteria;class;Gammaproteobacteria_incertae_sedis;order;Thioprofundum;genus	88.2	1.00E-123	444
924	1	0	0	0	0	0	0	0	0	Firmicutes	Clostridia	Clostridiales			S000131270 Clostridium uliginosum (T); CK55 Lineage=Root;rootrank;Bacteria;domain;Firmicutes;phylum;Clostridia;class;Clostridiales;order;Clostridiaceae 1;family;Clostridium sensu stricto;genus	87.28	1.00E-123	444
945	1	0	0	0	0	0	0	0	0	Firmicutes	Clostridia	Clostridiales			S000388299 Clostridium peptidivorans (T); TMC4; DSM 12505 Lineage=Root;rootrank;Bacteria;domain;Firmicutes;phylum;Clostridia;class;Clostridiales;order;Clostridiaceae 1;family;Clostridium sensu stricto;genus	89.25	1.00E-128	460
1420	0	0	0	0	2	0	0	0	0	Firmicutes	Clostridia	Clostridiales			S000409718 Clostridium bartlettii (T); WAL 16138 Lineage=Root;rootrank;Bacteria;domain;Firmicutes;phylum;Clostridia;class;Clostridiales;order;Peptostreptococcaceae;family;Clostridium XI;genus	97.19	0	662
1521	0	1	0	0	0	0	0	0	0	Firmicutes	Clostridia	Clostridiales			S000414352 Clostridium stercorarium Lineage=Root;rootrank;Bacteria;domain;Firmicutes;phylum;Clostridia;class;Clostridiales;order;Ruminococcaceae;family;Clostridium III;genus	96.51	2.00E-175	616
206	0	0	0	0	0	0	0	0	1	Firmicutes	Clostridia	Clostridiales			S000414352 Clostridium stercorarium Lineage=Root;rootrank;Bacteria;domain;Firmicutes;phylum;Clostridia;class;Clostridiales;order;Ruminococcaceae;family;Clostridium III;genus	94.63	1.00E-172	606
867	0	0	0	0	0	0	0	0	1	Firmicutes	Clostridia	Clostridiales			S000439515 Clostridium straminisolvens (T); CSK1 Lineage=Root;rootrank;Bacteria;domain;Firmicutes;phylum;Clostridia;class;Clostridiales;order;Ruminococcaceae;family;Clostridium III;genus	92.51	8.00E-150	531
401	14	11	19	6	18	1	1	1	0	Firmicutes	Clostridia	Clostridiales			S000721549 Gracilibacter thermotolerans; JW/YJL-S1; 5 Lineage=Root;rootrank;Bacteria;domain;Firmicutes;phylum;Clostridia;class;Clostridiales;order;Gracilibacteraceae;family;Gracilibacter;genus	99.73	0	680
971	0	1	1	3	3	1	3	0	4	Firmicutes	Clostridia	Clostridiales			S000721549 Gracilibacter thermotolerans; JW/YJL-S1; 5 Lineage=Root;rootrank;Bacteria;domain;Firmicutes;phylum;Clostridia;class;Clostridiales;order;Gracilibacteraceae;family;Gracilibacter;genus	93.55	5.00E-157	555
679	0	0	0	2	0	2	2	1	0	Firmicutes	Clostridia	Clostridiales			S000721549 Gracilibacter thermotolerans; JW/YJL-S1; 5 Lineage=Root;rootrank;Bacteria;domain;Firmicutes;phylum;Clostridia;class;Clostridiales;order;Gracilibacteraceae;family;Gracilibacter;genus	95.47	3.00E-169	595
2642	0	1	1	2	2	0	0	0	0	Firmicutes	Clostridia	Clostridiales			S000721549 Gracilibacter thermotolerans; JW/YJL-S1; 5 Lineage=Root;rootrank;Bacteria;domain;Firmicutes;phylum;Clostridia;class;Clostridiales;order;Gracilibacteraceae;family;Gracilibacter;genus	94.12	2.00E-160	566
844	0	2	0	0	0	0	0	0	0	Firmicutes	Clostridia	Clostridiales			S000721549 Gracilibacter thermotolerans; JW/YJL-S1; 5 Lineage=Root;rootrank;Bacteria;domain;Firmicutes;phylum;Clostridia;class;Clostridiales;order;Gracilibacteraceae;family;Gracilibacter;genus	96.17	0	638
1294	0	0	0	1	0	0	0	1	0	Firmicutes	Clostridia	Clostridiales			S000721549 Gracilibacter thermotolerans; JW/YJL-S1; 5 Lineage=Root;rootrank;Bacteria;domain;Firmicutes;phylum;Clostridia;class;Clostridiales;order;Gracilibacteraceae;family;Gracilibacter;genus	95.91	0	634
1733	0	0	1	0	0	0	0	0	0	Firmicutes	Clostridia	Clostridiales			S000721549 Gracilibacter thermotolerans; JW/YJL-S1; 5 Lineage=Root;rootrank;Bacteria;domain;Firmicutes;phylum;Clostridia;class;Clostridiales;order;Gracilibacteraceae;family;Gracilibacter;genus	92.66	2.00E-160	566
869	0	0	0	0	0	0	0	0	1	Firmicutes	Clostridia	Clostridiales			S000721549 Gracilibacter thermotolerans; JW/YJL-S1; 5 Lineage=Root;rootrank;Bacteria;domain;Firmicutes;phylum;Clostridia;class;Clostridiales;order;Gracilibacteraceae;family;Gracilibacter;genus	93.58	1.00E-157	556
2850	10	1	1	0	5	0	0	1	1	Firmicutes	Clostridia	Clostridiales			S000943595 Clostridium caenicola (T); EBR596; EBR-02E-0596 Lineage=Root;rootrank;Bacteria;domain;Firmicutes;phylum;Clostridia;class;Clostridiales;order;Ruminococcaceae;family;Clostridium III;genus	96.24	1.00E-173	610
470	0	0	1	0	0	0	0	0	0	Firmicutes	Clostridia	Clostridiales			S000943595 Clostridium caenicola (T); EBR596; EBR-02E-0596 Lineage=Root;rootrank;Bacteria;domain;Firmicutes;phylum;Clostridia;class;Clostridiales;order;Ruminococcaceae;family;Clostridium III;genus	95.15	2.00E-176	619
1845	0	0	1	0	0	0	0	0	0	Firmicutes	Clostridia	Clostridiales			S000943595 Clostridium caenicola (T); EBR596; EBR-02E-0596 Lineage=Root;rootrank;Bacteria;domain;Firmicutes;phylum;Clostridia;class;Clostridiales;order;Ruminococcaceae;family;Clostridium III;genus	96.2	0	643
262	1	0	0	0	0	0	0	1	0	Firmicutes	Clostridia	Clostridiales			S000970693 Clostridium glycolicum; C3_0702 Lineage=Root;rootrank;Bacteria;domain;Firmicutes;phylum;Clostridia;class;Clostridiales;order;Peptostreptococcaceae;family;Clostridium XI;genus	99.48	0	706
1987	2	0	0	6	0	0	0	4	1	Firmicutes	Clostridia	Clostridiales			S001168872 Clostridium maritimum; G12 Lineage=Root;rootrank;Bacteria;domain;Firmicutes;phylum;Clostridia;class;Clostridiales;order;Peptostreptococcaceae;family;Clostridium XI;genus	98.37	0	649
2536	3	4	2	3	3	4	6	0	1	Firmicutes	Clostridia	Clostridiales			S002350833 Clostridium hungatei; type strain: DSM 14427; 5 Lineage=Root;rootrank;Bacteria;domain;Firmicutes;phylum;Clostridia;class;Clostridiales;order;Ruminococcaceae;family;Clostridium III;genus	99.73	0	682
55	1	0	0	0	0	0	0	0	0	Firmicutes	Clostridia	Clostridiales			S002441012 Clostridium cellobioparum; JCM 1422 Lineage=Root;rootrank;Bacteria;domain;Firmicutes;phylum;Clostridia;class;Clostridiales;order;Ruminococcaceae;family;Clostridium III;genus	91.13	5.00E-142	505
2926	1	0	0	0	0	0	0	0	0	Firmicutes	Clostridia	Clostridiales			S002441012 Clostridium cellobioparum; JCM 1422 Lineage=Root;rootrank;Bacteria;domain;Firmicutes;phylum;Clostridia;class;Clostridiales;order;Ruminococcaceae;family;Clostridium III;genus	92.88	7.00E-161	568
2932	0	0	0	0	0	0	1	0	0	Firmicutes	Clostridia	Clostridiales			S003610510 Clostridium acidurici 9a Lineage=Root;rootrank;Bacteria;domain;Firmicutes;phylum;Clostridia;class;Clostridiales;order;Incertae Sedis XI;family;Clostridium XII;genus	99.22	0	697
1194	1	0	0	0	0	0	0	0	0	Firmicutes	Clostridia	Clostridiales			S003611802 Oxobacter pfennigii; type strain: DSM 3222; 36 Lineage=Root;rootrank;Bacteria;domain;Firmicutes;phylum;Clostridia;class;Clostridiales;order;Clostridiaceae 1;family;Oxobacter;genus	94.12	2.00E-160	566
1981	0	0	0	1	0	0	0	0	0	Firmicutes	Clostridia	Clostridiales			S003611802 Oxobacter pfennigii; type strain: DSM 3222; 36 Lineage=Root;rootrank;Bacteria;domain;Firmicutes;phylum;Clostridia;class;Clostridiales;order;Clostridiaceae 1;family;Oxobacter;genus	92.09	2.00E-155	549
2159	1	0	2	0	1	0	0	0	0	Firmicutes	Clostridia	Clostridiales			S003723521 Clostridium thermocellum; mbf-VZ-096; BC1 Lineage=Root;rootrank;Bacteria;domain;Firmicutes;phylum;Clostridia;class;Clostridiales;order;Ruminococcaceae;family;Clostridium III;genus	89.25	8.00E-130	464
1243	0	0	0	0	0	1	0	0	0	Firmicutes	Clostridia	Desulfitobacterales	Desulfitobacteraceae		S003286846 Moorella thermoacetica; Y73 Lineage=Root;rootrank;Bacteria;domain;Firmicutes;phylum;Clostridia;class;Thermoanaerobacterales;order;Thermoanaerobacteraceae;family;Moorella;genus	91.56	1.00E-152	540
2373	2	0	3	0	1	0	0	0	0	Firmicutes	Clostridia	Halanaerobiales	Halanaerobiaceae		S000001986 Halocella cellulosilytica (T); DSM 7362 Lineage=Root;rootrank;Bacteria;domain;Firmicutes;phylum;Clostridia;class;Halanaerobiales;order;Halanaerobiaceae;family;Halocella;genus	93.53	2.00E-156	553
3017	1	1	2	0	0	0	0	0	0	Firmicutes	Clostridia	Halanaerobiales	Halanaerobiaceae		S000001986 Halocella cellulosilytica (T); DSM 7362 Lineage=Root;rootrank;Bacteria;domain;Firmicutes;phylum;Clostridia;class;Halanaerobiales;order;Halanaerobiaceae;family;Halocella;genus	94.37	2.00E-170	599
3202	0	0	1	0	3	0	0	0	0	Firmicutes	Clostridia	OPB54			S000438509 Moorella glycerini (T); YS6 Lineage=Root;rootrank;Bacteria;domain;Firmicutes;phylum;Clostridia;class;Thermoanaerobacterales;order;Thermoanaerobacteraceae;family;Moorella;genus	91.82	3.00E-154	545
2162	0	0	0	1	0	0	1	0	0	Firmicutes	Clostridia	OPB54			S000438509 Moorella glycerini (T); YS6 Lineage=Root;rootrank;Bacteria;domain;Firmicutes;phylum;Clostridia;class;Thermoanaerobacterales;order;Thermoanaerobacteraceae;family;Moorella;genus	91.56	1.00E-152	540
540	6	24	40	20	63	2	1	2	4	Firmicutes	Clostridia	OPB54			S000721549 Gracilibacter thermotolerans; JW/YJL-S1; 5 Lineage=Root;rootrank;Bacteria;domain;Firmicutes;phylum;Clostridia;class;Clostridiales;order;Gracilibacteraceae;family;Gracilibacter;genus	90.91	2.00E-140	499
2823	2	0	1	0	0	0	0	0	0	Firmicutes	Clostridia	OPB54			S000721549 Gracilibacter thermotolerans; JW/YJL-S1; 5 Lineage=Root;rootrank;Bacteria;domain;Firmicutes;phylum;Clostridia;class;Clostridiales;order;Gracilibacteraceae;family;Gracilibacter;genus	90.35	5.00E-137	488
3257	0	0	3	0	1	0	0	0	0	Firmicutes	Clostridia	OPB54			S000721549 Gracilibacter thermotolerans; JW/YJL-S1; 5 Lineage=Root;rootrank;Bacteria;domain;Firmicutes;phylum;Clostridia;class;Clostridiales;order;Gracilibacteraceae;family;Gracilibacter;genus	90.32	5.00E-137	488
812	0	0	2	0	1	0	0	0	0	Firmicutes	Clostridia	OPB54			S000721549 Gracilibacter thermotolerans; JW/YJL-S1; 5 Lineage=Root;rootrank;Bacteria;domain;Firmicutes;phylum;Clostridia;class;Clostridiales;order;Gracilibacteraceae;family;Gracilibacter;genus	89.04	1.00E-128	460
1818	0	0	1	0	1	0	0	0	0	Firmicutes	Clostridia	OPB54			S000721549 Gracilibacter thermotolerans; JW/YJL-S1; 5 Lineage=Root;rootrank;Bacteria;domain;Firmicutes;phylum;Clostridia;class;Clostridiales;order;Gracilibacteraceae;family;Gracilibacter;genus	88.53	2.00E-125	449
460	0	0	1	0	0	0	0	0	0	Firmicutes	Clostridia	OPB54			S000721549 Gracilibacter thermotolerans; JW/YJL-S1; 5 Lineage=Root;rootrank;Bacteria;domain;Firmicutes;phylum;Clostridia;class;Clostridiales;order;Gracilibacteraceae;family;Gracilibacter;genus	90.62	1.00E-138	494
1142	0	0	1	0	0	0	0	0	0	Firmicutes	Clostridia	OPB54			S000721549 Gracilibacter thermotolerans; JW/YJL-S1; 5 Lineage=Root;rootrank;Bacteria;domain;Firmicutes;phylum;Clostridia;class;Clostridiales;order;Gracilibacteraceae;family;Gracilibacter;genus	90.61	2.00E-146	520
975	0	1	0	0	0	0	0	0	0	Firmicutes	Clostridia	OPB54			S000721549 Gracilibacter thermotolerans; JW/YJL-S1; 5 Lineage=Root;rootrank;Bacteria;domain;Firmicutes;phylum;Clostridia;class;Clostridiales;order;Gracilibacteraceae;family;Gracilibacter;genus	90.84	4.00E-148	525
2262	0	0	0	1	2	0	0	0	0	Firmicutes	Clostridia	OPB54			S000752077 Moorella perchloratireducens; An10 Lineage=Root;rootrank;Bacteria;domain;Firmicutes;phylum;Clostridia;class;Thermoanaerobacterales;order;Thermoanaerobacteraceae;family;Moorella;genus	90.82	1.00E-147	523
2057	0	0	1	0	0	0	0	0	0	Firmicutes	Clostridia	OPB54			S002165556 Thermincola potens JR Lineage=Root;rootrank;Bacteria;domain;Firmicutes;phylum;Clostridia;class;Clostridiales;order;Peptococcaceae 1;family;Thermincola;genus	89.42	4.00E-138	492
2813	0	0	1	0	0	0	0	0	0	Firmicutes	Clostridia	SHA-98			S000383946 Thermanaeromonas toyohensis (T); ToBE Lineage=Root;rootrank;Bacteria;domain;Firmicutes;phylum;Clostridia;class;Thermoanaerobacterales;order;Thermoanaerobacteraceae;family;Thermanaeromonas;genus	87.7	2.00E-120	433
549	0	1	0	0	0	0	0	0	0	Firmicutes	Clostridia	SHA-98			S000721549 Gracilibacter thermotolerans; JW/YJL-S1; 5 Lineage=Root;rootrank;Bacteria;domain;Firmicutes;phylum;Clostridia;class;Clostridiales;order;Gracilibacteraceae;family;Gracilibacter;genus	88.07	9.00E-130	464
1828	0	0	1	0	0	0	0	0	0	Firmicutes	Clostridia	SHA-98			S003257839 Zymophilus paucivorans; type strain: DSM 20756 Lineage=Root;rootrank;Bacteria;domain;Firmicutes;phylum;Negativicutes;class;Selenomonadales;order;Veillonellaceae;family;Zymophilus;genus	87.7	8.00E-120	431
3053	0	0	1	2	0	0	0	0	0	Firmicutes	Clostridia	Thermoanaerobacterales	Thermoanaerobacteraceae	Tepidanaerobacter	S003711252 Tepidanaerobacter acetatoxydans Re1 Lineage=Root;rootrank;Bacteria;domain;Firmicutes;phylum;Clostridia;class;Clostridiales;order;Clostridiales_Incertae Sedis III;family;Tepidanaerobacter;genus	94.4	7.00E-171	601
2307	4	1	3	1	1	0	0	0	0	Firmicutes	Clostridia	Thermoanaerobacterales	Thermoanaerobacteraceae	Thermacetogenium	S001015150 Syntrophaceticus schinkii (T); Sp3 Lineage=Root;rootrank;Bacteria;domain;Firmicutes;phylum;Clostridia;class;Thermoanaerobacterales;order;Thermoanaerobacteraceae;family;Syntrophaceticus;genus	96.24	3.00E-174	612
2409	0	1	0	0	0	0	0	0	0	Firmicutes	Clostridia	Thermoanaerobacterales	ThermoanaerobacteralesFamilyIII.IncertaeSedis	Thermovenabulum	S002289480 Thermosediminibacter oceani DSM 16646 Lineage=Root;rootrank;Bacteria;domain;Firmicutes;phylum;Clostridia;class;Clostridiales;order;Clostridiales_Incertae Sedis III;family;Thermosediminibacter;genus	97.7	0	675
628	0	0	0	0	0	0	1	0	0	Firmicutes	Clostridia	Thermoanaerobacterales			S000438509 Moorella glycerini (T); YS6 Lineage=Root;rootrank;Bacteria;domain;Firmicutes;phylum;Clostridia;class;Thermoanaerobacterales;order;Thermoanaerobacteraceae;family;Moorella;genus	89.84	1.00E-133	477
1537	0	0	0	0	1	0	0	0	0	Firmicutes	Clostridia				S000001965 Clostridium thermosuccinogenes (T); DSM 5807 Lineage=Root;rootrank;Bacteria;domain;Firmicutes;phylum;Clostridia;class;Clostridiales;order;Ruminococcaceae;family;Clostridium III;genus	87	2.00E-115	416
3055	0	0	0	0	1	0	0	0	0	Firmicutes	Clostridia				S000016025 Bacillus thermocloacae (T); DSM 5250 Lineage=Root;rootrank;Bacteria;domain;Firmicutes;phylum;Bacilli;class;Bacillales;order;Bacillaceae 1;family;Bacillus;genus	87.82	2.00E-127	457
2458	1	1	0	0	0	0	0	0	0	Firmicutes	Clostridia				S000375954 Pelotomaculum terephthalicicum (T); JT Lineage=Root;rootrank;Bacteria;domain;Firmicutes;phylum;Clostridia;class;Clostridiales;order;Peptococcaceae 2;family;Pelotomaculum;genus	87.79	2.00E-127	457
956	0	2	1	0	0	0	0	0	0	Firmicutes	Clostridia				S000391487 Gelria glutamica (T); TGO Lineage=Root;rootrank;Bacteria;domain;Firmicutes;phylum;Clostridia;class;Thermoanaerobacterales;order;Thermoanaerobacteraceae;family;Gelria;genus	89.22	1.00E-128	460
1977	0	1	0	0	0	0	0	0	0	Firmicutes	Clostridia				S000414352 Clostridium stercorarium Lineage=Root;rootrank;Bacteria;domain;Firmicutes;phylum;Clostridia;class;Clostridiales;order;Ruminococcaceae;family;Clostridium III;genus	90.54	7.00E-146	518
2440	0	0	1	0	1	0	0	0	0	Firmicutes	Clostridia				S000420543 Desulfitibacter alkalitolerans (T); sk.kt5 Lineage=Root;rootrank;Bacteria;domain;Firmicutes;phylum;Clostridia;class;Clostridiales;order;Peptococcaceae 1;family;Desulfitibacter;genus	90.62	1.00E-138	494
367	0	0	0	0	0	0	0	0	1	Firmicutes	Clostridia				S000426078 Desulfotomaculum nigrificans; CW-01 Lineage=Root;rootrank;Bacteria;domain;Firmicutes;phylum;Clostridia;class;Clostridiales;order;Peptococcaceae 2;family;Desulfotomaculum;genus	88.1	3.00E-129	462
950	1	0	0	0	0	0	0	0	0	Firmicutes	Clostridia				S000426636 Caldalkalibacillus thermarum (T); HA6 Lineage=Root;rootrank;Bacteria;domain;Firmicutes;phylum;Bacilli;class;Bacillales;order;Bacillales_incertae_sedis;family;Caldalkalibacillus;genus	87.53	3.00E-119	429
2833	1	0	0	0	0	0	0	0	0	Firmicutes	Clostridia				S000550678 Moorella thermoacetica; AMP Lineage=Root;rootrank;Bacteria;domain;Firmicutes;phylum;Clostridia;class;Thermoanaerobacterales;order;Thermoanaerobacteraceae;family;Moorella;genus	86.45	1.00E-118	427
944	2	0	2	0	2	0	0	0	0	Firmicutes	Clostridia				S000584753 Planifilum yunnanense (T); LA5 Lineage=Root;rootrank;Bacteria;domain;Firmicutes;phylum;Bacilli;class;Bacillales;order;Thermoactinomycetaceae 2;family;Planifilum;genus	86.74	4.00E-113	409
2939	0	1	3	0	2	1	0	0	1	Firmicutes	Clostridia				S000584753 Planifilum yunnanense (T); LA5 Lineage=Root;rootrank;Bacteria;domain;Firmicutes;phylum;Bacilli;class;Bacillales;order;Thermoactinomycetaceae 2;family;Planifilum;genus	87.4	4.00E-118	425

291	0	0	1	1	0	1	0	0	1	Firmicutes	Clostridia			S000584753 Planifilum yunnanense (T); LA5 Lineage=Root;rootrank;Bacteria;domain;Firmicutes;phylum;Bacilli;class;Bacillales;order;Thermoactinomycetaceae 2;family;Planifilum;genus	88.44	8.00E-125	448
1825	2	4	5	2	10	0	0	0	0	Firmicutes	Clostridia			S000721549 Gracilibacter thermotolerans; JW/YJL-S1; 5 Lineage=Root;rootrank;Bacteria;domain;Firmicutes;phylum;Clostridia;class;Clostridiales;order;Gracilibacteraceae;family;Gracilibacter;genus	90.37	2.00E-136	486
1128	1	0	1	0	0	0	0	1	0	Firmicutes	Clostridia			S000721549 Gracilibacter thermotolerans; JW/YJL-S1; 5 Lineage=Root;rootrank;Bacteria;domain;Firmicutes;phylum;Clostridia;class;Clostridiales;order;Gracilibacteraceae;family;Gracilibacter;genus	90.62	4.00E-138	492
3105	1	0	0	0	0	0	0	0	0	Firmicutes	Clostridia			S000721549 Gracilibacter thermotolerans; JW/YJL-S1; 5 Lineage=Root;rootrank;Bacteria;domain;Firmicutes;phylum;Clostridia;class;Clostridiales;order;Gracilibacteraceae;family;Gracilibacter;genus	94.9	1.00E-173	610
1894	1	0	0	0	0	0	0	0	0	Firmicutes	Clostridia			S000721549 Gracilibacter thermotolerans; JW/YJL-S1; 5 Lineage=Root;rootrank;Bacteria;domain;Firmicutes;phylum;Clostridia;class;Clostridiales;order;Gracilibacteraceae;family;Gracilibacter;genus	93.01	1.00E-153	544
3078	0	0	1	1	0	0	0	0	0	Firmicutes	Clostridia			S000721549 Gracilibacter thermotolerans; JW/YJL-S1; 5 Lineage=Root;rootrank;Bacteria;domain;Firmicutes;phylum;Clostridia;class;Clostridiales;order;Gracilibacteraceae;family;Gracilibacter;genus	92.23	1.00E-148	527
3031	0	0	1	0	0	0	0	0	0	Firmicutes	Clostridia			S000721549 Gracilibacter thermotolerans; JW/YJL-S1; 5 Lineage=Root;rootrank;Bacteria;domain;Firmicutes;phylum;Clostridia;class;Clostridiales;order;Gracilibacteraceae;family;Gracilibacter;genus	90.3	2.00E-135	483
43	1	0	0	0	1	1	0	0	0	Firmicutes	Clostridia			S000860311 Ammonifex thiophilus (T); SR Lineage=Root;rootrank;Bacteria;domain;Firmicutes;phylum;Clostridia;class;Thermoanaerobacterales;order;Thermoanaerobacteraceae;family;Ammonifex;genus	87.79	5.00E-127	455
3211	0	2	2	1	2	25	9	3	8	Firmicutes	Clostridia			S001152076 Thermaerobacter composti (T); Ni80 Lineage=Root;rootrank;Bacteria;domain;Firmicutes;phylum;Clostridia;class;Clostridiales;order;Clostridiales_Incertae Sedis XVII;family;Thermaerobacter;genus	87.94	2.00E-121	436
1065	0	0	1	0	0	0	0	0	0	Firmicutes	Clostridia			S001152076 Thermaerobacter composti (T); Ni80 Lineage=Root;rootrank;Bacteria;domain;Firmicutes;phylum;Clostridia;class;Clostridiales;order;Clostridiales_Incertae Sedis XVII;family;Thermaerobacter;genus	88.52	4.00E-133	475
1308	1	1	0	0	0	1	0	0	0	Firmicutes	Clostridia			S001572357 Caldicoprobacter oshimai (T); JW/HY-331 Lineage=Root;rootrank;Bacteria;domain;Firmicutes;phylum;Clostridia;class;Clostridiales;order;Incertae Sedis IV;family;Caldicoprobacter;genus	93.24	1.00E-153	544
1075	0	0	0	2	0	0	0	1	2	Firmicutes	Clostridia			S001572357 Caldicoprobacter oshimai (T); JW/HY-331 Lineage=Root;rootrank;Bacteria;domain;Firmicutes;phylum;Clostridia;class;Clostridiales;order;Incertae Sedis IV;family;Caldicoprobacter;genus	98.93	0	665
3106	0	0	0	1	0	0	0	0	0	Firmicutes	Clostridia			S002228266 Caloramator australicus; BF3 Lineage=Root;rootrank;Bacteria;domain;Firmicutes;phylum;Clostridia;class;Clostridiales;order;Clostridiaceae 1;family;Caloramator;genus	89.82	9.00E-140	497
200	0	0	0	1	0	0	0	0	0	Firmicutes	Clostridia			S002290426 Desulfotomaculum acetoxidans DSM 771 Lineage=Root;rootrank;Bacteria;domain;Firmicutes;phylum;Clostridia;class;Clostridiales;order;Peptococcaceae 2;family;Desulfotomaculum;genus	95.14	2.00E-175	616
296	0	0	0	1	0	0	0	0	0	Firmicutes	Clostridia			S002291311 Desulfotomaculum acetoxidans DSM 771 Lineage=Root;rootrank;Bacteria;domain;Firmicutes;phylum;Clostridia;class;Clostridiales;order;Peptococcaceae 2;family;Desulfotomaculum;genus	89.78	1.00E-133	477
1640	0	0	1	0	0	0	0	0	0	Firmicutes	Clostridia			S002350110 Thermaerobacter marianensis DSM 12885 Lineage=Root;rootrank;Bacteria;domain;Firmicutes;phylum;Clostridia;class;Clostridiales;order;Clostridiales_Incertae Sedis XVII;family;Thermaerobacter;genus	90.86	2.00E-140	499
1137	0	0	1	0	2	0	0	0	0	Firmicutes	Clostridia			S002355505 Syntrophobotulus glycolicus DSM 8271 (T) Lineage=Root;rootrank;Bacteria;domain;Firmicutes;phylum;Clostridia;class;Clostridiales;order;Peptococcaceae 1;family;Syntrophobotulus;genus	95.1	3.00E-164	579
2237	0	0	1	0	0	0	0	0	0	Firmicutes	Clostridia			S003277048 Clostridium clariflavum; CL-1 Lineage=Root;rootrank;Bacteria;domain;Firmicutes;phylum;Clostridia;class;Clostridiales;order;Ruminococcaceae;family;Clostridium III;genus	91.9	9.00E-155	547
929	0	0	1	0	0	0	0	0	0	Firmicutes	Clostridiales			S000399957 Garciella nitratireducens (T); Met 79; DSM 15102; CIP 107615 Lineage=Root;rootrank;Bacteria;domain;Firmicutes;phylum;Clostridia;class;Clostridiales;order;Eubacteriaceae;family;Garciella;genus	93.56	1.00E-163	577
660	0	0	0	0	0	1	0	0	0	Firmicutes				S000389038 Anaerobranca gottschalkii (T) Lineage=Root;rootrank;Bacteria;domain;Firmicutes;phylum;Clostridia;class;Clostridiales;order;Clostridiales_Incertae Sedis XIV;family;Anaerobranca;genus	88.49	2.00E-132	473
1170	0	0	1	4	0	0	0	0	0	Firmicutes				S000414351 Clostridium thermocellum Lineage=Root;rootrank;Bacteria;domain;Firmicutes;phylum;Clostridia;class;Clostridiales;order;Ruminococcaceae;family;Clostridium III;genus	86.13	2.00E-110	399
2836	0	1	0	3	0	1	0	0	0	Firmicutes				S000438735 Desulfotomaculum halophilum (T); SEBR 3139 Lineage=Root;rootrank;Bacteria;domain;Firmicutes;phylum;Clostridia;class;Clostridiales;order;Peptococcaceae 2;family;Desulfotomaculum;genus	92.76	5.00E-152	538
2937	0	0	0	0	1	0	0	0	0	Firmicutes				S000722658 Proteiniclasticum ruminis (T); D3RC-2 Lineage=Root;rootrank;Bacteria;domain;Firmicutes;phylum;Clostridia;class;Clostridiales;order;Clostridiaceae 1;family;Proteiniclasticum;genus	89.31	5.00E-137	488
2775	0	0	2	1	4	0	0	0	0	Firmicutes				S001152076 Thermaerobacter composti (T); Ni80 Lineage=Root;rootrank;Bacteria;domain;Firmicutes;phylum;Clostridia;class;Clostridiales;order;Clostridiales_Incertae Sedis XVII;family;Thermaerobacter;genus	92.49	2.00E-150	532
282	0	0	0	0	1	0	0	0	0	Firmicutes				S001199466 Cohnella laeviribosi; 4-7 Lineage=Root;rootrank;Bacteria;domain;Firmicutes;phylum;Bacilli;class;Bacillales;order;Paenibacillaceae 1;family;Cohnella;genus	91.42	4.00E-143	508
887	1	0	0	0	0	0	0	0	0	Firmicutes				S001353124 Paenibacillus residui; MC-247 Lineage=Root;rootrank;Bacteria;domain;Firmicutes;phylum;Bacilli;class;Bacillales;order;Paenibacillaceae 1;family;Paenibacillus;genus	93.18	3.00E-164	579
715	0	0	0	1	0	0	0	0	0	Firmicutes				S001933336 Candidatus Methylomirabilis oxyfera Lineage=Root;rootrank;Bacteria;domain;unclassified_Bacteria;	79.56	7.00E-66	252
970	1	0	0	0	0	0	0	0	0	Firmicutes				S002441012 Clostridium cellobioparum; JCM 1422 Lineage=Root;rootrank;Bacteria;domain;Firmicutes;phylum;Clostridia;class;Clostridiales;order;Ruminococcaceae;family;Clostridium III;genus	88.49	5.00E-132	472
1443	0	0	0	0	1	0	0	0	0	Firmicutes				S003298445 Desulfotomaculum halophilum; 17778_CA36 Lineage=Root;rootrank;Bacteria;domain;Firmicutes;phylum;unclassified_Firmicutes;	96.31	3.00E-164	579
1163	0	0	0	0	0	0	0	9	9	GAL5				S001328273 Fervidicola ferrireducens (T); Y170 Lineage=Root;rootrank;Bacteria;domain;Firmicutes;phylum;Clostridia;class;Clostridiales;order;Incertae Sedis III;family;Fervidicola;genus	85.6	2.00E-106	387
335	0	1	25	38	35	52	73	40	43	Gemmatimonadetes	Gemmatimonadetes			S000009676 Pelobacter acetylenicus (T); WoAcy1 DSM2348 Lineage=Root;rootrank;Bacteria;domain;"Proteobacteria";phylum;Deltaproteobacteria;class;Desulfuromonadales;order;Desulfuromonadaceae;family;Pelobacter;genus	85.56	7.00E-106	385
2417	0	0	0	13	9	4	4	4	8	Gemmatimonadetes	Gemmatimonadetes			S000009676 Pelobacter acetylenicus (T); WoAcy1 DSM2348 Lineage=Root;rootrank;Bacteria;domain;"Proteobacteria";phylum;Deltaproteobacteria;class;Desulfuromonadales;order;Desulfuromonadaceae;family;Pelobacter;genus	85.48	7.00E-106	385
914	0	0	7	2	3	1	2	0	2	Gemmatimonadetes	Gemmatimonadetes			S000009676 Pelobacter acetylenicus (T); WoAcy1 DSM2348 Lineage=Root;rootrank;Bacteria;domain;"Proteobacteria";phylum;Deltaproteobacteria;class;Desulfuromonadales;order;Desulfuromonadaceae;family;Pelobacter;genus	85.56	7.00E-106	385
1630	0	0	1	3	1	1	3	1	1	Gemmatimonadetes	Gemmatimonadetes			S000009676 Pelobacter acetylenicus (T); WoAcy1 DSM2348 Lineage=Root;rootrank;Bacteria;domain;"Proteobacteria";phylum;Deltaproteobacteria;class;Desulfuromonadales;order;Desulfuromonadaceae;family;Pelobacter;genus	85.75	1.00E-107	390
728	0	0	1	0	4	2	0	0	2	Gemmatimonadetes	Gemmatimonadetes			S000009676 Pelobacter acetylenicus (T); WoAcy1 DSM2348 Lineage=Root;rootrank;Bacteria;domain;"Proteobacteria";phylum;Deltaproteobacteria;class;Desulfuromonadales;order;Desulfuromonadaceae;family;Pelobacter;genus	84.72	7.00E-101	368
3209	0	0	0	0	2	0	0	0	1	Gemmatimonadetes	Gemmatimonadetes			S000009676 Pelobacter acetylenicus (T); WoAcy1 DSM2348 Lineage=Root;rootrank;Bacteria;domain;"Proteobacteria";phylum;Deltaproteobacteria;class;Desulfuromonadales;order;Desulfuromonadaceae;family;Pelobacter;genus	86.1	3.00E-109	396
2284	0	0	0	0	1	0	0	0	0	Gemmatimonadetes	Gemmatimonadetes			S000009676 Pelobacter acetylenicus (T); WoAcy1 DSM2348 Lineage=Root;rootrank;Bacteria;domain;"Proteobacteria";phylum;Deltaproteobacteria;class;Desulfuromonadales;order;Desulfuromonadaceae;family;Pelobacter;genus	86.22	7.00E-111	401
1303	0	0	0	1	0	0	0	0	0	Gemmatimonadetes	Gemmatimonadetes			S000009676 Pelobacter acetylenicus (T); WoAcy1 DSM2348 Lineage=Root;rootrank;Bacteria;domain;"Proteobacteria";phylum;Deltaproteobacteria;class;Desulfuromonadales;order;Desulfuromonadaceae;family;Pelobacter;genus	85.93	3.00E-114	412
2506	0	0	0	0	0	0	1	0	0	Gemmatimonadetes	Gemmatimonadetes			S000009676 Pelobacter acetylenicus (T); WoAcy1 DSM2348 Lineage=Root;rootrank;Bacteria;domain;"Proteobacteria";phylum;Deltaproteobacteria;class;Desulfuromonadales;order;Desulfuromonadaceae;family;Pelobacter;genus	85.07	1.00E-102	374
2182	0	0	0	0	0	0	0	1	0	Gemmatimonadetes	Gemmatimonadetes			S000009676 Pelobacter acetylenicus (T); WoAcy1 DSM2348 Lineage=Root;rootrank;Bacteria;domain;"Proteobacteria";phylum;Deltaproteobacteria;class;Desulfuromonadales;order;Desulfuromonadaceae;family;Pelobacter;genus	86.86	3.00E-114	412
2663	2	0	0	11	0	0	0	6	18	Gemmatimonadetes	Gemmatimonadetes			S000018219 Natronoanaerobium salstagnum; O-M12SP-2 Lineage=Root;rootrank;Bacteria;domain;Firmicutes;phylum;Clostridia;class;Natranaerobiales;order;Natranaerobiaceae;family;Natranaerobius;genus	87.17	7.00E-116	418
2897	0	0	0	0	0	1	0	0	0	Gemmatimonadetes	Gemmatimonadetes			S000019682 Gram-positive bacteria SOGA22 Lineage=Root;rootrank;Bacteria;domain;"Actinobacteria";phylum;Actinobacteria;class;Actinobacteridae;subclass;Actinomycetales;order;Frankineae;suborder;unclassified_Frankineae;	85.98	1.00E-108	394
1784	0	0	4	13	39	14	13	50	106	Gemmatimonadetes	Gemmatimonadetes			S000128481 Thermoleophilum album; type strain: ATCC 35266 Lineage=Root;rootrank;Bacteria;domain;"Actinobacteria";phylum;Actinobacteria;class;Rubrobacteridae;subclass;Thermoleophilales;order;Thermoleophilaceae;family;Thermoleophilum;genus	85.71	2.00E-106	387
1528	5	23	29	3	7	9	16	9	3	Gemmatimonadetes	Gemmatimonadetes			S000128481 Thermoleophilum album; type strain: ATCC 35266 Lineage=Root;rootrank;Bacteria;domain;"Actinobacteria";phylum;Actinobacteria;class;Rubrobacteridae;subclass;Thermoleophilales;order;Thermoleophilaceae;family;Thermoleophilum;genus	85.64	2.00E-106	387
907	0	0	0	3	2	1	0	0	1	Gemmatimonadetes	Gemmatimonadetes			S000128481 Thermoleophilum album; type strain: ATCC 35266 Lineage=Root;rootrank;Bacteria;domain;"Actinobacteria";phylum;Actinobacteria;class;Rubrobacteridae;subclass;Thermoleophilales;order;Thermoleophilaceae;family;Thermoleophilum;genus	85.95	4.00E-108	392
5	1	0	0	0	0	0	0	0	0	Gemmatimonadetes	Gemmatimonadetes			S000128481 Thermoleophilum album; type strain: ATCC 35266 Lineage=Root;rootrank;Bacteria;domain;"Actinobacteria";phylum;Actinobacteria;class;Rubrobacteridae;subclass;Thermoleophilales;order;Thermoleophilaceae;family;Thermoleophilum;genus	83.12	7.00E-91	335
1783	0	0	0	0	1	0	0	0	0	Gemmatimonadetes	Gemmatimonadetes			S000128481 Thermoleophilum album; type strain: ATCC 35266 Lineage=Root;rootrank;Bacteria;domain;"Actinobacteria";phylum;Actinobacteria;class;Rubrobacteridae;subclass;Thermoleophilales;order;Thermoleophilaceae;family;Thermoleophilum;genus	87.63	1.00E-118	427
2687	0	0	0	0	1	0	0	0	0	Gemmatimonadetes	Gemmatimonadetes			S000128481 Thermoleophilum album; type strain: ATCC 35266 Lineage=Root;rootrank;Bacteria;domain;"Actinobacteria";phylum;Actinobacteria;class;Rubrobacteridae;subclass;Thermoleophilales;order;Thermoleophilaceae;family;Thermoleophilum;genus	86.18	9.00E-110	398
3022	32	50	5	0	4	8	6	0	0	Gemmatimonadetes	Gemmatimonadetes			S000130885 Thermoleophilum album (T); type strain: ATCC 35263 Lineage=Root;rootrank;Bacteria;domain;"Actinobacteria";phylum;Actinobacteria;class;Rubrobacteridae;subclass;Thermoleophilales;order;Thermoleophilaceae;family;Thermoleophilum;genus	86.49	5.00E-112	405
514	0	3	16	4	9	3	3	3	2	Gemmatimonadetes	Gemmatimonadetes			S000130885 Thermoleophilum album (T); type strain: ATCC 35263 Lineage=Root;rootrank;Bacteria;domain;"Actinobacteria";phylum;Actinobacteria;class;Rubrobacteridae;subclass;Thermoleophilales;order;Thermoleophilaceae;family;Thermoleophilum;genus	88.07	1.00E-128	460
1391	4	2	4	5	4	4	6	0	6	Gemmatimonadetes	Gemmatimonadetes			S000130885 Thermoleophilum album (T); type strain: ATCC 35263 Lineage=Root;rootrank;Bacteria;domain;"Actinobacteria";phylum;Actinobacteria;class;Rubrobacteridae;subclass;Thermoleophilales;order;Thermoleophilaceae;family;Thermoleophilum;genus	88.92	5.00E-127	455
1919	0	8	9	0	7	1	0	1	0	Gemmatimonadetes	Gemmatimonadetes			S000130885 Thermoleophilum album (T); type strain: ATCC 35263 Lineage=Root;rootrank;Bacteria;domain;"Actinobacteria";phylum;Actinobacteria;class;Rubrobacteridae;subclass;Thermoleophilales;order;Thermoleophilaceae;family;Thermoleophilum;genus	87.3	5.00E-117	422
404	1	0	1	5	3	2	6	0	1	Gemmatimonadetes	Gemmatimonadetes			S000130885 Thermoleophilum album (T); type strain: ATCC 35263 Lineage=Root;rootrank;Bacteria;domain;"Actinobacteria";phylum;Actinobacteria;class;Rubrobacteridae;subclass;Thermoleophilales;order;Thermoleophilaceae;family;Thermoleophilum;genus	87.43	2.00E-115	416
2116	0	0	3	0	5	3	0	1	7	Gemmatimonadetes	Gemmatimonadetes			S000130885 Thermoleophilum album (T); type strain: ATCC 35263 Lineage=Root;rootrank;Bacteria;domain;"Actinobacteria";phylum;Actinobacteria;class;Rubrobacteridae;subclass;Thermoleophilales;order;Thermoleophilaceae;family;Thermoleophilum;genus	87.77	1.00E-117	424
3218	0	0	0	1	5	4	3	1	5	Gemmatimonadetes	Gemmatimonadetes			S000130885 Thermoleophilum album (T); type strain: ATCC 35263 Lineage=Root;rootrank;Bacteria;domain;"Actinobacteria";phylum;Actinobacteria;class;Rubrobacteridae;subclass;Thermoleophilales;order;Thermoleophilaceae;family;Thermoleophilum;genus	85.41	9.00E-105	381
906	1	4	0	0	0	2	7	0	0	Gemmatimonadetes	Gemmatimonadetes			S000130885 Thermoleophilum album (T); type strain: ATCC 35263 Lineage=Root;rootrank;Bacteria;domain;"Actinobacteria";phylum;Actinobacteria;class;Rubrobacteridae;subclass;Thermoleophilales;order;Thermoleophilaceae;family;Thermoleophilum;genus	85.68	2.00E-106	387
185	0	0	3	2	0	0	0	0	0	Gemmatimonadetes	Gemmatimonadetes			S000130885 Thermoleophilum album (T); type strain: ATCC 35263 Lineage=Root;rootrank;Bacteria;domain;"Actinobacteria";phylum;Actinobacteria;class;Rubrobacteridae;subclass;Thermoleophilales;order;Thermoleophilaceae;family;Thermoleophilum;genus	85.95	4.00E-108	392
1295	0	0	0	0	1	0	0	0	0	Gemmatimonadetes	Gemmatimonadetes			S000130885 Thermoleophilum album (T); type strain: ATCC 35263 Lineage=Root;rootrank;Bacteria;domain;"Actinobacteria";phylum;Actinobacteria;class;Rubrobacteridae;subclass;Thermoleophilales;order;Thermoleophilaceae;family;Thermoleophilum;genus	87.9	2.00E-120	433
837	0	0	0	1	0	0	0	0	0	Gemmatimonadetes	Gemmatimonadetes			S000130885 Thermoleophilum album (T); type strain: ATCC 35263 Lineage=Root;rootrank;Bacteria;domain;"Actinobacteria";phylum;Actinobacteria;class;Rubrobacteridae;subclass;Thermoleophilales;order;Thermoleophilaceae;family;Thermoleophilum;genus	85.96	3.00E-115	416
1703	0	0	1	3	20	20	17	3	13	Gemmatimonadetes	Gemmatimonadetes			S000392836 Desulfacinum subterraneum; 101 Lineage=Root;rootrank;Bacteria;domain;"Proteobacteria";phylum;Deltaproteobacteria;class;Syntrophobacterales;order;Syntrophobacteraceae;family;Desulfacinum;genus	84.41	3.00E-99	363
779	0	0	0	0	1	0	0	0	0	Gemmatimonadetes	Gemmatimonadetes			S000392836 Desulfacinum subterraneum; 101 Lineage=Root;rootrank;Bacteria;domain;"Proteobacteria";phylum;Deltaproteobacteria;class;Syntrophobacterales;order;Syntrophobacteraceae;family;Desulfacinum;genus	86.36	7.00E-111	401
847	15	24	44	12	52	22	27	24	16	Gemmatimonadetes	Gemmatimonadetes			S000614201 Desulfovirgula thermocuniculi (T); RL80JIV Lineage=Root;rootrank;Bacteria;domain;Firmicutes;phylum;Clostridia;class;Thermoanaerobacterales;order;Thermoanaerobacteraceae;family;Desulfovirgula;genus	84.18	5.00E-97	355
2719	0	0	0	0	10	71	71	19	60	Gemmatimonadetes	Gemmatimonadetes			S000642543 Calditerricola satsumensis (T); YMO81 Lineage=Root;rootrank;Bacteria;domain;Firmicutes;phylum;Bacilli;class;Bacillales;order;Bacillales_incertae_sedis;family;Calditerricola;genus	86.44	7.00E-111	401
1607	3	12	25	0	13	20	13	1	0	Gemmatimonadetes	Gemmatimonadetes			S000721674 Polyangium thaxteri; BICC 8959 Lineage=Root;rootrank;Bacteria;domain;"Proteobacteria";phylum;Deltaproteobacteria;class;Myxococcales;order;Sorangiineae;suborder;Polyangiaceae;family;Chondromyces;genus	85.79	1.00E-107	390
258	0	0	0	11	1	0	1	0	1	Gemmatimonadetes	Gemmatimonadetes			S000721674 Polyangium thaxteri; BICC 8959 Lineage=Root;rootrank;Bacteria;domain;"Proteobacteria";phylum;Deltaproteobacteria;class;Myxococcales;order;Sorangiineae;suborder;Polyangiaceae;family;Chondromyces;genus	86.02	1.00E-108	394
1737	0	1	0	0	0	1	3	0	0	Gemmatimonadetes	Gemmatimonadetes			S000721674 Polyangium thaxteri; BICC 8959 Lineage=Root;rootrank;Bacteria;domain;"Proteobacteria";phylum;Deltaproteobacteria;class;Myxococcales;order;Sorangiineae;suborder;Polyangiaceae;family;Chondromyces;genus	85.25	1.00E-103	377
3095	0	0	0	0	2	0	0	0	0	Gemmatimonadetes	Gemmatimonadetes			S000721674 Polyangium thaxteri; BICC 8959 Lineage=Root;rootrank;Bacteria;domain;"Proteobacteria";phylum;Deltaproteobacteria;class;Myxococcales;order;Sorangiineae;suborder;Polyangiaceae;family;Chondromyces;genus	85.44	2.00E-105	383
1000	0	0	0	0	0	6	5	0	0	Gemmatimonadetes	Gemmatimonadetes			S000858360 Thermosulfidibacter takaii (T); ABI70S6 (= JCM13301, = DSM 17441) Lineage=Root;rootrank;Bacteria;domain;"Aquificae";phylum;Aquificae;class;Aquificales;order;Aquificales_incertae_sedis;family;Thermosulfidibacter;genus	86.07	5.00E-107	388
1470	0	1	0	0	0	0	0	1	0	Gemmatimonadetes	Gemmatimonadetes			S000858360 Thermosulfidibacter takaii (T); ABI70S6 (= JCM13301, = DSM 17441) Lineage=Root;rootrank;Bacteria;domain;"Aquificae";phylum;Aquificae;class;Aquificales;order;Aquificales_incertae_sedis;family;Thermosulfidibacter;genus	85.75	3.00E-104	379
756	0	0	0	0	0	1	0	0	0	Gemmatimonadetes	Gemmatimonadetes			S000870713 Calditerricola yamamurae (T); YMO722 Lineage=Root;rootrank;Bacteria;domain;Firmicutes;phylum;Bacilli;class;Bacillales;order;Bacillales_incertae_sedis;family;Calditerricola;genus	87.28	2.00E-122	440
2928	0	0	0	4	2	0	1	0	0	Gemmatimonadetes	Gemmatimonadetes			S000978795 Streptomyces tenebrarius; NBRC 16177 Lineage=Root;rootrank;Bacteria;domain;"Actinobacteria";phylum;Actinobacteria;class;Actinobacteridae;subclass;Actinomycetales;order;Pseudonocardineae;suborder;Pseudonocardiaceae;family;Streptoalloteichus;genus	85.44	9.00E-105	381
2165	0	0	1	3	7	2	0	1	0	Gemmatimonadetes	Gemmatimonadetes			S001152076 Thermaerobacter composti (T); Ni80 Lineage=Root;rootrank;Bacteria;domain;Firmicutes;phylum;Clostridia;class;Clostridiales;order;Clostridiales_Incertae Sedis XVII;family;Thermaerobacter;genus	85.64	7.00E-106	385
1926	0	0	12	47	28	0	2	3	5	Gemmatimonadetes	Gemmatimonadetes			S001350322 Gemmatimonas aurantiaca T-27; T-27 (= NBRC 100505) Lineage=Root;rootrank;Bacteria;domain;"Gemmatimonadetes";phylum;Gemmatimonadetes;class;Gemmatimonadales;order;Gemmatimonadaceae;family;Gemmatimonas;genus	85.79	3.00E-114	412
1558	0	0	14	12	34	7	3	8	11	Gemmatimonadetes	Gemmatimonadetes			S001350322 Gemmatimonas aurantiaca T-27; T-27 (= NBRC 100505) Lineage=Root;rootrank;Bacteria;domain;"Gemmatimonadetes";phylum;Gemmatimonadetes;class;Gemmatimonadales;order;Gemmatimonadaceae;family;Gemmatimonas;genus	86.24	9.00E-110	398
1234	0	0	3	10	19	0	0	14	16	Gemmatimonadetes	Gemmatimonadetes			S001350322 Gemmatimonas aurantiaca T-27; T-27 (= NBRC 100505) Lineage=Root;rootrank;Bacteria;domain;"Gemmatimonadetes";phylum;Gemmatimonadetes;class;Gemmatimonadales;order;Gemmatimonadaceae;family;Gemmatimonas;genus	86.21	2.00E-110	399
2560	1	2	18	0	1	5	4	0	0	Gemmatimonadetes	Gemmatimonadetes			S001350322 Gemmatimonas aurantiaca T-27; T-27 (= NBRC 100505) Lineage=Root;rootrank;Bacteria;domain;"Gemmatimonadetes";phylum;Gemmatimonadetes;class;Gemmatimonadales;order;Gemmatimonadaceae;family;Gemmatimonas;genus	85.07	1.00E-103	377
3190	3	2	0	5	5	2	2	2	2	Gemmatimonadetes	Gemmatimonadetes			S001350322 Gemmatimonas aurantiaca T-27; T-27 (= NBRC 100505) Lineage=Root;rootrank;Bacteria;domain;"Gemmatimonadetes";phylum;Gemmatimonadetes;class;Gemmatimonadales;order;Gemmatimonadaceae;family;Gemmatimonas;genus	86.77	1.00E-113	411
1151	0	0	1	3	5	0	0	3	0	Gemmatimonadetes	Gemmatimonadetes			S001350322 Gemmatimonas aurantiaca T-27; T-27 (= NBRC 100505) Lineage=Root;rootrank;Bacteria;domain;"Gemmatimonadetes";phylum;Gemmatimonadetes;class;Gemmatimonadales;order;Gemmatimonadaceae;family;Gemmatimonas;genus	86.24	2.00E-110	399
3083	0	0	0	6	0	0	0	0	0	Gemmatimonadetes	Gemmatimonadetes			S001350322 Gemmatimonas aurantiaca T-27; T-27 (= NBRC 100505) Lineage=Root;rootrank;Bacteria;domain;"Gemmatimonadetes";phylum;Gemmatimonadetes;class;Gemmatimonadales;order;Gemmatimonadaceae;family;Gemmatimonas;genus	84.57	3.00E-99	363
829	0	0	0	0	1	0	0	2	1	Gemmatimonadetes	Gemmatimonadetes			S001350322 Gemmatimonas aurantiaca T-27; T-27 (= NBRC 100505) Lineage=Root;rootrank;Bacteria;domain;"Gemmatimonadetes";phylum;Gemmatimonadetes;class;Gemmatimonadales;order;Gemmatimonadaceae;family;Gemmatimonas;genus	86.17	2.00E-110	399
2548	0	0	0	0	1	0	0	0	0	Gemmatimonadetes	Gemmatimonadetes			S001350322 Gemmatimonas aurantiaca T-27; T-27 (= NBRC 100505) Lineage=Root;rootrank;Bacteria;domain;"Gemmatimonadetes";phylum;Gemmatimonadetes;class;Gemmatimonadales;order;Gemmatimonadaceae;family;Gemmatimonas;genus	84.3	1.00E-103	377
744	0	0	0	1	0	0	0	0	0	Gemmatimonadetes	Gemmatimonadetes			S001350322 Gemmatimonas aurantiaca T-27; T-27 (= NBRC 100505) Lineage=Root;rootrank;Bacteria;domain;"Gemmatimonadetes";phylum;Gemmatimonadetes;class;Gemmatimonadales;order;Gemmatimonadaceae;family;Gemmatimonas;genus	82.71	4.00E-88	326
3090	0	0	0	1	0	0	0	0	0	Gemmatimonadetes	Gemmatimonadetes			S001350322 Gemmatimonas aurantiaca T-27; T-27 (= NBRC 100505) Lineage=Root;rootrank;Bacteria;domain;"Gemmatimonadetes";phylum;Gemmatimonadetes;class;Gemmatimonadales;order;Gemmatimonadaceae;family;Gemmatimonas;genus	85.49	7.00E-106	385
2878	0	0	0	0	0	0	0	0	1	Gemmatimonadetes	Gemmatimonadetes			S001350322 Gemmatimonas aurantiaca T-27; T-27 (= NBRC 100505) Lineage=Root;rootrank;Bacteria;domain;"Gemmatimonadetes";phylum;Gemmatimonadetes;class;Gemmatimonadales;order;Gemmatimonadaceae;family;Gemmatimonas;genus	85.71	4.00E-113	409
935	3	2	9	0	0	4	0	0	0	Gemmatimonadetes	Gemmatimonadetes			S002304636 Dichotomicrobium thermohalophilum; type strain: DSM5002 Lineage=Root;rootrank;Bacteria;domain;"Proteobacteria";phylum;Alphaproteobacteria;class;Rhizobiales;order;unclassified_Rhizobiales;	85.75	7.00E-106	385
3117	0	0	0	0	2	0	0	0	3	Gemmatimonadetes	Gemmatimonadetes			S002445899 Streptomyces griseoaurantiacus; SZ1 Lineage=Root;rootrank;Bacteria;domain;"Actinobacteria";phylum;Actinobacteria;class;Actinobacteridae;subclass;Actinomycetales;order;Streptomycineae;suborder;Streptomycetaceae;family;Streptomyces;genus	85.37	3.00E-104	379
1213	0	0	0	6	4	4	1	0	2	Gemmatimonadetes	Gemmatimonadetes			S002949687 Peptococcus niger; JCM 6506 Lineage=Root;rootrank;Bacteria;domain;Firmicutes;phylum;Clostridia;class;Clostridiales;order;Peptococcaceae 1;family;Peptococcus;genus	85.29	3.00E-104	379
2011	0	0	0	0	0	0	0	2	5	Gemmatimonadetes	Gemmatimonadetes			S002949699 Collinsella tanakaei; JCM 16103 Lineage=Root;rootrank;Bacteria;domain;"Actinobacteria";phylum;Actinobacteria;class;Coriobacteridae;subclass;Coriobacteriales;order;"Coriobacterineae";suborder;Coriobacteriaceae;family;Collinsella;genus	85.4	7.00E-101	368
2093	6	2	15	5	12	40	26	23	4	Gemmatimonadetes	Gemmatimonadetes			S003257833 Desulfotomaculum luciae; type strain: DSM 12396; 4 Lineage=Root;rootrank;Bacteria;domain;Firmicutes;phylum;Clostridia;class;Clostridiales;order;Peptococcaceae 2;family;Desulfotomaculum;genus	80.98	6.00E-77	289
1986	0	0	2	2	0	0	0	0	1	Gemmatimonadetes	Gemmatimonadetes			S003259712 Syntrophothermus lipocalidus DSM 12680; TGB-C1 Lineage=Root;rootrank;Bacteria;domain;Firmicutes;phylum;Clostridia;class;Clostridiales;order;Syntrophomonadaceae;family;Syntrophothermus;genus	84.48	3.00E-105	383
1633	0	0	0	0	1	0	0	0	0	Gemmatimonadetes	Gemmatimonadetes			S003259712 Syntrophothermus lipocalidus DSM 12680; TGB-C1 Lineage=Root;rootrank;Bacteria;domain;Firmicutes;phylum;Clostridia;class;Clostridiales;order;Syntrophomonadaceae;family;Syntrophothermus;genus	84.73	2.00E-106	387
3043	1	1	16	13	11	3	12	1	6	Gemmatimonadetes	Gemmatimonadetes			S003610437 Pelobacter carbinolicus (T); DSM 2380 Lineage=Root;rootrank;Bacteria;domain;"Proteobacteria";phylum;Deltaproteobacteria;class;Desulfuromonadales;order;Desulfuromonadaceae;family;Pelobacter;genus	84.84	7.00E-101	368
1691	0	0	0	0	1	0	0	1	1	Gemmatimonadetes	Gemmatimonadetes	Gemmatimonadales		S000130885 Thermoleophilum album (T); type strain: ATCC 35263 Lineage=Root;rootrank;Bacteria;domain;"Actinobacteria";phylum;Actinobacteria;class;Rubrobacteridae;subclass;Thermoleophilales;order;Thermoleophilaceae;family;Thermoleophilum;genus	87.47	9.00E-125	448
3018	0	0	0	0	0	0	1	0	0	Gemmatimonadetes	Gemmatimonadetes	Gemmatimonadales		S000841843 Rubrobacter taiwanensis (T); LS-293 Lineage=Root;rootrank;Bacteria;domain;"Actinobacteria";phylum;Actinobacteria;class;Rubrobacteridae;subclass;Rubrobacterales;order;"Rubrobacterineae";suborder;Rubrobacteraceae;family;Rubrobacter;genus	87.21	4.00E-123	442
2131	0	0	6	54	24	17	15	14	13	Gemmatimonadetes	Gemmatimonadetes	Gemmatimonadales		S001350322 Gemmatimonas aurantiaca T-27; T-27 (= NBRC 100505) Lineage=Root;rootrank;Bacteria;domain;"Gemmatimonadetes";phylum;Gemmatimonadetes;class;Gemmatimonadales;order;Gemmatimonadaceae;family;Gemmatimonas;genus	88.52	2.00E-132	473
995	0	0	0	0	1	0	0	17	38	Gemmatimonadetes	Gemmatimonadetes	Gemmatimonadales		S001350322 Gemmatimonas aurantiaca T-27; T-27 (= NBRC 100505) Lineage=Root;rootrank;Bacteria;domain;"Gemmatimonadetes";phylum;Gemmatimonadetes;class;Gemmatimonadales;order;Gemmatimonadaceae;family;Gemmatimonas;genus	87	2.00E-115	416
2884	0	0	0	26	1	0	0	2	0	Gemmatimonadetes	Gemmatimonadetes	Gemmatimonadales		S001350322 Gemmatimonas aurantiaca T-27; T-27 (= NBRC 100505) Lineage=Root;rootrank;Bacteria;domain;"Gemmatimonadetes";phylum;Gemmatimonadetes;class;Gemmatimonadales;order;Gemmatimonadaceae;family;Gemmatimonas;genus	87.95	1.00E-128	460
2804	0	0	1	12	3	6	1	2	2	Gemmatimonadetes	Gemmatimonadetes	Gemmatimonadales		S001350322 Gemmatimonas aurantiaca T-27; T-27 (= NBRC 100505) Lineage=Root;rootrank;Bacteria;domain;"Gemmatimonadetes";phylum;Gemmatimonadetes;class;Gemmatimonadales;order;Gemmatimonadaceae;family;Gemmatimonas;genus	89.63	5.00E-132	472
2967	0	0	0	8	6	3	6	1	3	Gemmatimonadetes	Gemmatimonadetes	Gemmatimonadales		S001350322 Gemmatimonas aurantiaca T-27; T-27 (= NBRC 100505) Lineage=Root;rootrank;Bacteria;domain;"Gemmatimonadetes";phylum;Gemmatimonadetes;class;Gemmatimonadales;order;Gemmatimonadaceae;family;Gemmatimonas;genus	87.77	8.00E-120	431
3163	0	0	0	8	4	1	1	3	0	Gemmatimonadetes	Gemmatimonadetes	Gemmatimonadales		S001350322 Gemmatimonas aurantiaca T-27; T-27 (= NBRC 100505) Lineage=Root;rootrank;Bacteria;domain;"Gemmatimonadetes";phylum;Gemmatimonadetes;class;Gemmatimonadales;order;Gemmatimonadaceae;family;Gemmatimonas;genus	87.09	2.00E-122	440
1629	0	0	1	4	0	2	1	5	1	Gemmatimonadetes	Gemmatimonadetes	Gemmatimonadales		S001350322 Gemmatimonas aurantiaca T-27; T-27 (= NBRC 100505) Lineage=Root;rootrank;Bacteria;domain;"Gemmatimonadetes";phylum;Gemmatimonadetes;class;Gemmatimonadales;order;Gemmatimonadaceae;family;Gemmatimonas;genus	88.44	8.00E-125	448
2317	0	0	0	4	3	0	0	1	7	Gemmatimonadetes	Gemmatimonadetes	Gemmatimonadales		S001350322 Gemmatimonas aurantiaca T-27; T-27 (= NBRC 100505) Lineage=Root;rootrank;Bacteria;domain;"Gemmatimonadetes";phylum;Gemmatimonadetes;class;Gemmatimonadales;order;Gemmatimonadaceae;family;Gemmatimonas;genus	88	2.00E-121	436
760	0	0	0	9	2	0	0	0	0	Gemmatimonadetes	Gemmatimonadetes	Gemmatimonadales		S001350322 Gemmatimonas aurantiaca T-27; T-27 (= NBRC 100505) Lineage=Root;rootrank;Bacteria;domain;"Gemmatimonadetes";phylum;Gemmatimonadetes;class;Gemmatimonadales;order;Gemmatimonadaceae;family;Gemmatimonas;genus	86.86	9.00E-115	414
1225	0	0	0	1	3	2	0	0	4	Gemmatimonadetes	Gemmatimonadetes	Gemmatimonadales		S001350322 Gemmatimonas aurantiaca T-27; T-27 (= NBRC 100505) Lineage=Root;rootrank;Bacteria;domain;"Gemmatimonadetes";phylum;Gemmatimonadetes;class;Gemmatimonadales;order;Gemmatimonadaceae;family;Gemmatimonas;genus	87.73	2.00E-120	433
217	0	0	1	2	1	0	0	0	0	Gemmatimonadetes	Gemmatimonadetes	Gemmatimonadales		S001350322 Gemmatimonas aurantiaca T-27; T-27 (= NBRC 100505) Lineage=Root;rootrank;Bacteria;domain;"Gemmatimonadetes";phylum;Gemmatimonadetes;class;Gemmatimonadales;order;Gemmatimonadaceae;family;Gemmatimonas;genus	89.78	4.00E-133	475
978	0	0	0	3	0	1	0	0	0	Gemmatimonadetes	Gemmatimonadetes	Gemmatimonadales		S001350322 Gemmatimonas aurantiaca T-27; T-27 (= NBRC 100505) Lineage=Root;rootrank;Bacteria;domain;"Gemmatimonadetes";phylum;Gemmatimonadetes;class;Gemmatimonadales;order;Gemmatimonadaceae;family;Gemmatimonas;genus	87.94	2.00E-121	436
399	0	0	0	0	1	0	0	0	0	Gemmatimonadetes	Gemmatimonadetes	Gemmatimonadales		S001350322 Gemmatimonas aurantiaca T-27; T-27 (= NBRC 100505) Lineage=Root;rootrank;Bacteria;domain;"Gemmatimonadetes";phylum;Gemmatimonadetes;class;Gemmatimonadales;order;Gemmatimonadaceae;family;Gemmatimonas;genus	89.19	5.00E-127	455
772	0	0	0	0	1	0	0	0	0	Gemmatimonadetes	Gemmatimonadetes	Gemmatimonadales		S001350322 Gemmatimonas aurantiaca T-27; T-27 (= NBRC 100505) Lineage=Root;rootrank;Bacteria;domain;"Gemmatimonadetes";phylum;Gemmatimonadetes;class;Gemmatimonadales;order;Gemmatimonadaceae;family;Gemmatimonas;genus	87.73	2.00E-120	433
2958	0	0	0	2	2	0	0	1	0	Gemmatimonadetes	Gemmatimonadetes	Gemmatimonadales		S003709934 Calderihabitans maritimus; KKC1 Lineage=Root;rootrank;Bacteria;domain;Firmicutes;phylum;Clostridia;class;Thermoanaerobacterales;order;Thermoanaerobacteraceae;family;unclassified_Thermoanaerobacteraceae;	87.21	4.00E-123	442
3158	0	0	0	0	1	0	0	0	0	Gemmatimonadetes	Gemmatimonadetes	Gemmatimonadales		S003709934 Calderihabitans maritimus; KKC1 Lineage=Root;rootrank;Bacteria;domain;Firmicutes;phylum;Clostridia;class;Thermoanaerobacterales;order;Thermoanaerobacteraceae;family;unclassified_Thermoanaerobacteraceae;	87.47	1.00E-117	424
149	0	0	16	64	49	2	0	0	9	Gemmatimonadetes	Gemmatimonadetes	Gemmatimonadales	Gemmatimonadaceae	S001350322 Gemmatimonas aurantiaca T-27; T-27 (= NBRC 100505) Lineage=Root;rootrank;Bacteria;domain;"Gemmatimonadetes";phylum;Gemmatimonadetes;class;Gemmatimonadales;order;Gemmatimonadaceae;family;Gemmatimonas;genus	94.92	2.00E-165	582
2089	0	0	5	24	28	27	35	7	7	Gemmatimonadetes	Gemmatimonadetes	Gemmatimonadales	Gemmatimonadaceae	S001350322 Gemmatimonas aurantiaca T-27; T-27 (= NBRC 100505) Lineage=Root;rootrank;Bacteria;domain;"Gemmatimonadetes";phylum;Gemmatimonadetes;class;Gemmatimonadales;order;Gemmatimonadaceae;family;Gemmatimonas;genus	91.98	5.00E-147	521
449	0	0	0	21	4	5	2	0	2	Gemmatimonadetes	Gemmatimonadetes	Gemmatimonadales	Gemmatimonadaceae	S001350322 Gemmatimonas aurantiaca T-27; T-27 (= NBRC 100505) Lineage=Root;rootrank;Bacteria;domain;"Gemmatimonadetes";phylum;Gemmatimonadetes;class;Gemmatimonadales;order;Gemmatimonadaceae;family;Gemmatimonas;genus	92.45	1.00E-148	527
1578	0	0	0	7	7	1	0	5	1	Gemmatimonadetes	Gemmatimonadetes	Gemmatimonadales	Gemmatimonadaceae	S001350322 Gemmatimonas aurantiaca T-27; T-27 (= NBRC 100505) Lineage=Root;rootrank;Bacteria;domain;"Gemmatimonadetes";phylum;Gemmatimonadetes;class;Gemmatimonadales;order;Gemmatimonadaceae;family;Gemmatimonas;genus	90.96	2.00E-140	499
1829	0	0	0	0	1	9	7	0	0	Gemmatimonadetes	Gemmatimonadetes	Gemmatimonadales	Gemmatimonadaceae	S001350322 Gemmatimonas aurantiaca T-27; T-27 (= NBRC 100505) Lineage=Root;rootrank;Bacteria;domain;"Gemmatimonadetes";phylum;Gemmatimonadetes;class;Gemmatimonadales;order;Gemmatimonadaceae;family;Gemmatimonas;genus	91.33	7.00E-151	534
1657	0	0	1	11	0	0	0	3	0	Gemmatimonadetes	Gemmatimonadetes	Gemmatimonadales	Gemmatimonadaceae	S001350322 Gemmatimonas aurantiaca T-27; T-27 (= NBRC 100505) Lineage=Root;rootrank;Bacteria;domain;"Gemmatimonadetes";phylum;Gemmatimonadetes;class;Gemmatimonadales;order;Gemmatimonadaceae;family;Gemmatimonas;genus	93.64	7.00E-166	584
1426	0	0	0	6	4	2	0	0	1	Gemmatimonadetes	Gemmatimonadetes	Gemmatimonadales	Gemmatimonadaceae	S001350322 Gemmatimonas aurantiaca T-27; T-27 (= NBRC 100505) Lineage=Root;rootrank;Bacteria;domain;"Gemmatimonadetes";phylum;Gemmatimonadetes;class;Gemmatimonadales;order;Gemmatimonadaceae;family;Gemmatimonas;genus	86.33	2.00E-111	403
1541	0	0	0	1	3	5	1	0	0	Gemmatimonadetes	Gemmatimonadetes	Gemmatimonadales	Gemmatimonadaceae	S001350322 Gemmatimonas aurantiaca T-27; T-27 (= NBRC 100505) Lineage=Root;rootrank;Bacteria;domain;"Gemmatimonadetes";phylum;Gemmatimonadetes;class;Gemmatimonadales;order;Gemmatimonadaceae;family;Gemmatimonas;genus	92.18	4.00E-148	525
29	0	0	0	7	1	0	0	0	0	Gemmatimonadetes	Gemmatimonadetes	Gemmatimonadales	Gemmatimonadaceae	S001350322 Gemmatimonas aurantiaca T-27; T-27 (= NBRC 100505) Lineage=Root;rootrank;Bacteria;domain;"Gemmatimonadetes";phylum;Gemmatimonadetes;class;Gemmatimonadales;order;Gemmatimonadaceae;family;Gemmatimonas;genus	85.71	4.00E-108	392
1967	0	0	0	2	4	0	0	0	0	Gemmatimonadetes	Gemmatimonadetes	Gemmatimonadales	Gemmatimonadaceae	S001350322 Gemmatimonas aurantiaca T-27; T-27 (= NBRC 100505) Lineage=Root;rootrank;Bacteria;domain;"Gemmatimonadetes";phylum;Gemmatimonadetes;class;Gemmatimonadales;order;Gemmatimonadaceae;family;Gemmatimonas;genus	93.05	1.00E-153	544
219	0	0	0	0	2	1	0	0	0	Gemmatimonadetes	Gemmatimonadetes	Gemmatimonadales	Gemmatimonadaceae	S001350322 Gemmatimonas aurantiaca T-27; T-27 (= NBRC 100505) Lineage=Root;rootrank;Bacteria;domain;"Gemmatimonadetes";phylum;Gemmatimonadetes;class;Gemmatimonadales;order;Gemmatimonadaceae;family;Gemmatimonas;genus	86.92	6.00E-122	438
1454	0	0	0	2	0	0	0	0	0	Gemmatimonadetes	Gemmatimonadetes	Gemmatimonadales	Gemmatimonadaceae	S001350322 Gemmatimonas aurantiaca T-27; T-27 (= NBRC 100505) Lineage=Root;rootrank;Bacteria;domain;"Gemmatimonadetes";phylum;Gemmatimonadetes;class;Gemmatimonadales;order;Gemmatimonadaceae;family;Gemmatimonas;genus	92.27	1.00E-148	527
547	0	0	0	1	0	1	0	0	0	Gemmatimonadetes	Gemmatimonadetes	Gemmatimonadales	Gemmatimonadaceae	S001350322 Gemmatimonas aurantiaca T-27; T-27 (= NBRC 100505) Lineage=Root;rootrank;Bacteria;domain;"Gemmatimonadetes";phylum;Gemmatimonadetes;class;Gemmatimonadales;order;Gemmatimonadaceae;family;Gemmatimonas;genus	90.21	2.00E-135	483
878	0	0	0	0	1	0	0	0	0	Gemmatimonadetes	Gemmatimonadetes	Gemmatimonadales	Gemmatimonadaceae	S001350322 Gemmatimonas aurantiaca T-27; T-27 (= NBRC 100505) Lineage=Root;rootrank;Bacteria;domain;"Gemmatimonadetes";phylum;Gemmatimonadetes;class;Gemmatimonadales;order;Gemmatimonadaceae;family;Gemmatimonas;genus	85.96	3.00E-115	416
1559	0	0	0	1	0	0	0	0	0	Gemmatimonadetes	Gemmatimonadetes	Gemmatimonadales	Gemmatimonadaceae	S001350322 Gemmatimonas aurantiaca T-27; T-27 (= NBRC 100505) Lineage=Root;rootrank;Bacteria;domain;"Gemmatimonadetes";phylum;Gemmatimonadetes;class;Gemmatimonadales;order;Gemmatimonadaceae;family;Gemmatimonas;genus	86.27	2.00E-117	424

856	0	0	2	12	2	0	0	3	0	Gemmatimonadetes	Gemmatimonadetes	Gemmatimonadales	Gemmatimonadaceae	Gemmatimonas	S001350322 Gemmatimonas aurantiaca T-27; T-27 (= NBRC 100505) Lineage=Root;rootrank;Bacteria;domain;"Gemmatimonadetes";phylum;Gemmatimonadetes;class;Gemmatimonadales;order;Gemmatimonadaceae;family;Gemmatimonas;genus	91.03	1.00E-148	527
119	0	0	0	1	2	0	0	3	0	Gemmatimonadetes	Gemmatimonadetes	Gemmatimonadales	Gemmatimonadaceae	Gemmatimonas	S001350322 Gemmatimonas aurantiaca T-27; T-27 (= NBRC 100505) Lineage=Root;rootrank;Bacteria;domain;"Gemmatimonadetes";phylum;Gemmatimonadetes;class;Gemmatimonadales;order;Gemmatimonadaceae;family;Gemmatimonas;genus	90.35	5.00E-137	488
2938	0	0	0	1	4	0	0	0	0	Gemmatimonadetes	Gemmatimonadetes	Gemmatimonadales	Gemmatimonadaceae	Gemmatimonas	S001350322 Gemmatimonas aurantiaca T-27; T-27 (= NBRC 100505) Lineage=Root;rootrank;Bacteria;domain;"Gemmatimonadetes";phylum;Gemmatimonadetes;class;Gemmatimonadales;order;Gemmatimonadaceae;family;Gemmatimonas;genus	91.28	2.00E-150	532
2390	0	0	0	1	3	1	0	0	0	Gemmatimonadetes	Gemmatimonadetes	Gemmatimonadales	Gemmatimonadaceae	Gemmatimonas	S001350322 Gemmatimonas aurantiaca T-27; T-27 (= NBRC 100505) Lineage=Root;rootrank;Bacteria;domain;"Gemmatimonadetes";phylum;Gemmatimonadetes;class;Gemmatimonadales;order;Gemmatimonadaceae;family;Gemmatimonas;genus	91.79	1.00E-153	544
2170	0	0	0	2	1	0	0	0	0	Gemmatimonadetes	Gemmatimonadetes	Gemmatimonadales	Gemmatimonadaceae	Gemmatimonas	S001350322 Gemmatimonas aurantiaca T-27; T-27 (= NBRC 100505) Lineage=Root;rootrank;Bacteria;domain;"Gemmatimonadetes";phylum;Gemmatimonadetes;class;Gemmatimonadales;order;Gemmatimonadaceae;family;Gemmatimonas;genus	90.54	5.00E-137	488
1030	0	0	0	0	0	0	0	0	1	Gemmatimonadetes	Gemmatimonadetes	Gemmatimonadales	Gemmatimonadaceae		S001350322 Gemmatimonas aurantiaca T-27; T-27 (= NBRC 100505) Lineage=Root;rootrank;Bacteria;domain;"Gemmatimonadetes";phylum;Gemmatimonadetes;class;Gemmatimonadales;order;Gemmatimonadaceae;family;Gemmatimonas;genus	90.48	7.00E-146	518
1983	0	0	0	0	1	0	0	0	0	Gemmatimonadetes	Gemmatimonadetes	Gemmatimonadales			S001350322 Gemmatimonas aurantiaca T-27; T-27 (= NBRC 100505) Lineage=Root;rootrank;Bacteria;domain;"Gemmatimonadetes";phylum;Gemmatimonadetes;class;Gemmatimonadales;order;Gemmatimonadaceae;family;Gemmatimonas;genus	86.1	3.00E-109	396
2526	5	50	4	1	0	6	12	0	0	Gemmatimonadetes	Gemmatimonadetes				S000128481 Thermoleophilum album; type strain: ATCC 35266 Lineage=Root;rootrank;Bacteria;domain;"Actinobacteria";phylum;Actinobacteria;class;Rubrobacteridae;subclass;Thermoleophilales;order;Thermoleophilaceae;family;Thermoleophilum;genus	84.05	2.00E-96	353
2168	4	4	7	5	3	5	4	1	0	Gemmatimonadetes	Gemmatimonadetes				S000128481 Thermoleophilum album; type strain: ATCC 35266 Lineage=Root;rootrank;Bacteria;domain;"Actinobacteria";phylum;Actinobacteria;class;Rubrobacteridae;subclass;Thermoleophilales;order;Thermoleophilaceae;family;Thermoleophilum;genus	81.59	7.00E-81	302
1182	4	1	0	0	0	0	0	0	0	Gemmatimonadetes	Gemmatimonadetes				S000128481 Thermoleophilum album; type strain: ATCC 35266 Lineage=Root;rootrank;Bacteria;domain;"Actinobacteria";phylum;Actinobacteria;class;Rubrobacteridae;subclass;Thermoleophilales;order;Thermoleophilaceae;family;Thermoleophilum;genus	84.66	1.00E-97	357
1446	0	0	0	0	1	0	0	0	0	Gemmatimonadetes	Gemmatimonadetes				S000128481 Thermoleophilum album; type strain: ATCC 35266 Lineage=Root;rootrank;Bacteria;domain;"Actinobacteria";phylum;Actinobacteria;class;Rubrobacteridae;subclass;Thermoleophilales;order;Thermoleophilaceae;family;Thermoleophilum;genus	86.01	3.00E-115	416
1624	182	488	110	4	22	55	50	3	0	Gemmatimonadetes	Gemmatimonadetes				S000130885 Thermoleophilum album (T); type strain: ATCC 35263 Lineage=Root;rootrank;Bacteria;domain;"Actinobacteria";phylum;Actinobacteria;class;Rubrobacteridae;subclass;Thermoleophilales;order;Thermoleophilaceae;family;Thermoleophilum;genus	85.37	9.00E-105	381
193	17	54	27	5	10	6	8	0	0	Gemmatimonadetes	Gemmatimonadetes				S000130885 Thermoleophilum album (T); type strain: ATCC 35263 Lineage=Root;rootrank;Bacteria;domain;"Actinobacteria";phylum;Actinobacteria;class;Rubrobacteridae;subclass;Thermoleophilales;order;Thermoleophilaceae;family;Thermoleophilum;genus	85.09	4.00E-103	375
2000	0	9	5	0	2	1	1	0	0	Gemmatimonadetes	Gemmatimonadetes				S000130885 Thermoleophilum album (T); type strain: ATCC 35263 Lineage=Root;rootrank;Bacteria;domain;"Actinobacteria";phylum;Actinobacteria;class;Rubrobacteridae;subclass;Thermoleophilales;order;Thermoleophilaceae;family;Thermoleophilum;genus	85.37	9.00E-105	381
2801	0	1	5	1	0	0	0	0	1	Gemmatimonadetes	Gemmatimonadetes				S000130885 Thermoleophilum album (T); type strain: ATCC 35263 Lineage=Root;rootrank;Bacteria;domain;"Actinobacteria";phylum;Actinobacteria;class;Rubrobacteridae;subclass;Thermoleophilales;order;Thermoleophilaceae;family;Thermoleophilum;genus	82.05	1.00E-83	311
793	0	1	2	0	0	0	0	0	0	Gemmatimonadetes	Gemmatimonadetes				S000130885 Thermoleophilum album (T); type strain: ATCC 35263 Lineage=Root;rootrank;Bacteria;domain;"Actinobacteria";phylum;Actinobacteria;class;Rubrobacteridae;subclass;Thermoleophilales;order;Thermoleophilaceae;family;Thermoleophilum;genus	82.49	2.00E-86	320
2924	0	0	0	0	0	0	0	0	1	Gemmatimonadetes	Gemmatimonadetes				S000130885 Thermoleophilum album (T); type strain: ATCC 35263 Lineage=Root;rootrank;Bacteria;domain;"Actinobacteria";phylum;Actinobacteria;class;Rubrobacteridae;subclass;Thermoleophilales;order;Thermoleophilaceae;family;Thermoleophilum;genus	85.68	2.00E-112	407
2567	0	0	1	2	0	0	0	0	0	Gemmatimonadetes	Gemmatimonadetes				S000390061 Heliorestis baculata (T); OS H1 (T) Lineage=Root;rootrank;Bacteria;domain;Firmicutes;phylum;Clostridia;class;Clostridiales;order;Heliobacteriaceae;family;Heliorestis;genus	85.18	2.00E-105	383
625	0	0	5	2	2	1	2	0	0	Gemmatimonadetes	Gemmatimonadetes				S000420543 Desulfitibacter alkalitolerans (T); sk.kt5 Lineage=Root;rootrank;Bacteria;domain;Firmicutes;phylum;Clostridia;class;Clostridiales;order;Peptococcaceae 1;family;Desulfitibacter;genus	85.87	2.00E-106	387
942	0	1	1	0	0	0	0	0	0	Gemmatimonadetes	Gemmatimonadetes				S000434346 Desulfotomaculum thermobenzoicum (T); TPO Lineage=Root;rootrank;Bacteria;domain;Firmicutes;phylum;Clostridia;class;Clostridiales;order;Peptococcaceae 2;family;Desulfotomaculum;genus	80.56	1.00E-73	278
2386	0	0	1	0	0	0	0	0	0	Gemmatimonadetes	Gemmatimonadetes				S000434346 Desulfotomaculum thermobenzoicum (T); TPO Lineage=Root;rootrank;Bacteria;domain;Firmicutes;phylum;Clostridia;class;Clostridiales;order;Peptococcaceae 2;family;Desulfotomaculum;genus	79.95	1.00E-69	265
2732	1	1	21	4	9	16	25	30	35	Gemmatimonadetes	Gemmatimonadetes				S000469501 Heliobacillus mobilis (T); DSM 6151 Lineage=Root;rootrank;Bacteria;domain;Firmicutes;phylum;Clostridia;class;Clostridiales;order;Heliobacteriaceae;family;Heliobacillus;genus	87.06	7.00E-116	418
2259	0	1	174	973	552	244	231	715	525	Gemmatimonadetes	Gemmatimonadetes				S000569921 Pelotomaculum isophthalicicum (T); JI Lineage=Root;rootrank;Bacteria;domain;Firmicutes;phylum;Clostridia;class;Clostridiales;order;Peptococcaceae 2;family;Pelotomaculum;genus	85.14	1.00E-103	377
1881	0	0	18	94	66	7	21	9	17	Gemmatimonadetes	Gemmatimonadetes				S000569921 Pelotomaculum isophthalicicum (T); JI Lineage=Root;rootrank;Bacteria;domain;Firmicutes;phylum;Clostridia;class;Clostridiales;order;Peptococcaceae 2;family;Pelotomaculum;genus	85.41	7.00E-106	385
2951	0	0	0	0	0	7	16	78	126	Gemmatimonadetes	Gemmatimonadetes				S000569921 Pelotomaculum isophthalicicum (T); JI Lineage=Root;rootrank;Bacteria;domain;Firmicutes;phylum;Clostridia;class;Clostridiales;order;Peptococcaceae 2;family;Pelotomaculum;genus	86.29	7.00E-111	401
882	0	0	1	1	0	0	0	0	0	Gemmatimonadetes	Gemmatimonadetes				S001014825 Geoalkalibacter subterraneus (T); Red1 Lineage=Root;rootrank;Bacteria;domain;"Proteobacteria";phylum;Deltaproteobacteria;class;Desulfuromonadales;order;Geobacteraceae;family;Geoalkalibacter;genus	86.01	3.00E-115	416
607	0	0	0	5	5	0	0	0	0	Gemmatimonadetes	Gemmatimonadetes				S001350322 Gemmatimonas aurantiaca T-27; T-27 (= NBRC 100505) Lineage=Root;rootrank;Bacteria;domain;"Gemmatimonadetes";phylum;Gemmatimonadetes;class;Gemmatimonadales;order;Gemmatimonadaceae;family;Gemmatimonas;genus	87.97	2.00E-121	436
2401	0	0	0	0	1	0	0	0	0	Gemmatimonadetes	Gemmatimonadetes				S001350322 Gemmatimonas aurantiaca T-27; T-27 (= NBRC 100505) Lineage=Root;rootrank;Bacteria;domain;"Gemmatimonadetes";phylum;Gemmatimonadetes;class;Gemmatimonadales;order;Gemmatimonadaceae;family;Gemmatimonas;genus	87.56	3.00E-125	449
615	0	0	0	0	0	0	1	0	0	Gemmatimonadetes	Gemmatimonadetes				S002290453 Nitrosococcus watsoni C-113 Lineage=Root;rootrank;Bacteria;domain;"Proteobacteria";phylum;Gammaproteobacteria;class;Chromatiales;order;Chromatiaceae;family;Nitrosococcus;genus	85.09	4.00E-103	375
2217	0	0	0	3	0	3	2	0	0	Gemmatimonadetes	Gemmatimonadetes				S003257833 Desulfotomaculum luciae; type strain: DSM 12396; 4 Lineage=Root;rootrank;Bacteria;domain;Firmicutes;phylum;Clostridia;class;Clostridiales;order;Peptococcaceae 2;family;Desulfotomaculum;genus	81.44	3.00E-80	300
2	1	0	0	0	0	0	0	0	0	Gemmatimonadetes	Gemmatimonadetes				S003257833 Desulfotomaculum luciae; type strain: DSM 12396; 4 Lineage=Root;rootrank;Bacteria;domain;Firmicutes;phylum;Clostridia;class;Clostridiales;order;Peptococcaceae 2;family;Desulfotomaculum;genus	81.68	7.00E-81	302
796	0	0	0	0	0	1	0	0	0	Gemmatimonadetes	Gemmatimonadetes				S003257833 Desulfotomaculum luciae; type strain: DSM 12396; 4 Lineage=Root;rootrank;Bacteria;domain;Firmicutes;phylum;Clostridia;class;Clostridiales;order;Peptococcaceae 2;family;Desulfotomaculum;genus	83.76	3.00E-95	350
1532	0	0	0	0	0	0	0	0	1	Gemmatimonadetes	Gemmatimonadetes				S003258802 Naumannella halotolerans; WS4624 Lineage=Root;rootrank;Bacteria;domain;"Actinobacteria";phylum;Actinobacteria;class;Actinobacteridae;subclass;Actinomycetales;order;Propionibacterineae;suborder;Propionibacteriaceae;family;Microlunatus;genus	85.41	3.00E-104	379
791	0	0	2	2	3	0	0	0	1	Gemmatimonadetes	Gemmatimonadetes				S003610437 Pelobacter carbinolicus (T); DSM 2380 Lineage=Root;rootrank;Bacteria;domain;"Proteobacteria";phylum;Deltaproteobacteria;class;Desulfuromonadales;order;Desulfuromonadaceae;family;Pelobacter;genus	86.17	9.00E-110	398
2616	0	0	0	6	0	0	0	0	0	Gemmatimonadetes	Gemmatimonadetes				S003710612 Geminicoccus roseus; SU27 Lineage=Root;rootrank;Bacteria;domain;"Proteobacteria";phylum;Alphaproteobacteria;class;Alphaproteobacteria_incertae_sedis;order;Geminicoccus;genus	86.41	2.00E-110	399
1080	0	0	0	1	0	0	0	0	0	GN04	GN5				S000428878 Desulfofrigus fragile (T); LSv21 Lineage=Root;rootrank;Bacteria;domain;"Proteobacteria";phylum;Deltaproteobacteria;class;Desulfobacterales;order;Desulfobacteraceae;family;Desulfofrigus;genus	84.77	6.00E-107	388
2260	0	0	0	2	1	0	0	1	2	GN04					S000438509 Moorella glycerini (T); YS6 Lineage=Root;rootrank;Bacteria;domain;Firmicutes;phylum;Clostridia;class;Thermoanaerobacterales;order;Thermoanaerobacteraceae;family;Moorella;genus	87.43	7.00E-116	418
2764	0	0	0	2	14	66	79	13	5	Niterbed cluster					S000841843 Rubrobacter taiwanensis (T); LS-293 Lineage=Root;rootrank;Bacteria;domain;"Actinobacteria";phylum;Actinobacteria;class;Rubrobacteridae;subclass;Rubrobacterales;order;"Rubrobacterineae";suborder;Rubrobacteraceae;family;Rubrobacter;genus	84.76	5.00E-102	372
2520	0	0	0	24	2	0	0	0	2	Niterbed cluster					S000841843 Rubrobacter taiwanensis (T); LS-293 Lineage=Root;rootrank;Bacteria;domain;"Actinobacteria";phylum;Actinobacteria;class;Rubrobacteridae;subclass;Rubrobacterales;order;"Rubrobacterineae";suborder;Rubrobacteraceae;family;Rubrobacter;genus	83.29	2.00E-91	337
1081	0	0	0	8	0	0	0	0	0	Niterbed cluster					S000841843 Rubrobacter taiwanensis (T); LS-293 Lineage=Root;rootrank;Bacteria;domain;"Actinobacteria";phylum;Actinobacteria;class;Rubrobacteridae;subclass;Rubrobacterales;order;"Rubrobacterineae";suborder;Rubrobacteraceae;family;Rubrobacter;genus	82.93	2.00E-90	333
1015	0	0	0	0	0	0	0	1	0	Niterbed cluster					S000841843 Rubrobacter taiwanensis (T); LS-293 Lineage=Root;rootrank;Bacteria;domain;"Actinobacteria";phylum;Actinobacteria;class;Rubrobacteridae;subclass;Rubrobacterales;order;"Rubrobacterineae";suborder;Rubrobacteraceae;family;Rubrobacter;genus	85.71	5.00E-107	388
12	0	0	1	2	4	0	0	0	0	Niterbed cluster					S001577231 Thermosipho affectus (T); ik275mar Lineage=Root;rootrank;Bacteria;domain;"Thermotogae";phylum;Thermotogae;class;Thermotogales;order;Thermotogaceae;family;Thermosipho;genus	83.65	5.00E-92	339
352	0	0	0	0	0	1	0	0	1	Niterbed cluster					S002288086 Caldanaerobacter subterraneus (T); MB4 Lineage=Root;rootrank;Bacteria;domain;Firmicutes;phylum;Clostridia;class;Thermoanaerobacterales;order;Thermoanaerobacteraceae;family;Caldanaerobacter;genus	85.71	5.00E-107	388
788	0	0	0	4	0	0	0	0	0	Niterbed cluster					S002289364 Thermoanaerobacter italicus Ab9 Lineage=Root;rootrank;Bacteria;domain;Firmicutes;phylum;Clostridia;class;Thermoanaerobacterales;order;Thermoanaerobacteraceae;family;Thermoanaerobacter;genus	84.32	5.00E-97	355
2681	0	0	0	1	0	0	0	0	0	Niterbed cluster					S003287741 Rubrobacter bracarensis; CECT 7926 = VFA70612_S5 Lineage=Root;rootrank;Bacteria;domain;"Actinobacteria";phylum;Actinobacteria;class;Rubrobacteridae;subclass;Rubrobacterales;order;"Rubrobacterineae";suborder;Rubrobacteraceae;family;Rubrobacter;genus	84.22	4.00E-98	359
2309	0	0	0	7	1	7	1	0	0	Nitrospirae	Nitrospira	Nitrospirales	Nitrospiraceae	Nitrospira	S001328527 Desulfosoma caldarium (T); USBA-053 Lineage=Root;rootrank;Bacteria;domain;"Proteobacteria";phylum;Deltaproteobacteria;class;Syntrophobacterales;order;Syntrophobacteraceae;family;Desulfosoma;genus	85.6	3.00E-104	379
1670	0	0	0	6	2	4	3	0	1	Nitrospirae	Nitrospira	Nitrospirales	Nitrospiraceae	Nitrospira	S001328527 Desulfosoma caldarium (T); USBA-053 Lineage=Root;rootrank;Bacteria;domain;"Proteobacteria";phylum;Deltaproteobacteria;class;Syntrophobacterales;order;Syntrophobacteraceae;family;Desulfosoma;genus	83.51	7.00E-91	335
2818	0	0	0	8	8	0	0	0	1	Nitrospirae	Nitrospira	Nitrospirales	Nitrospiraceae	Nitrospira	S001612170 Acidithiobacillus caldus; SMK Lineage=Root;rootrank;Bacteria;domain;"Proteobacteria";phylum;Gammaproteobacteria;class;Acidithiobacillales;order;Acidithiobacillaceae;family;Acidithiobacillus;genus	83.47	2.00E-90	333
854	0	0	0	2	2	0	0	0	0	Nitrospirae	Nitrospira	Nitrospirales	Nitrospiraceae	Nitrospira	S002290453 Nitrosococcus watsoni C-113 Lineage=Root;rootrank;Bacteria;domain;"Proteobacteria";phylum;Gammaproteobacteria;class;Chromatiales;order;Chromatiaceae;family;Nitrosococcus;genus	84.22	7.00E-101	368
568	0	0	0	8	8	0	0	0	0	Nitrospirae	Nitrospira	Nitrospirales	Nitrospiraceae	Nitrospira	S002967174 Enterococcus faecalis; mf2 Lineage=Root;rootrank;Bacteria;domain;Firmicutes;phylum;Bacilli;class;Lactobacillales;order;Enterococcaceae;family;Enterococcus;genus	82.26	3.00E-90	333
1145	0	0	0	14	32	55	31	45	67	Nitrospirae	Nitrospira	Nitrospirales	Nitrospiraceae	Nitrospira	S003610437 Pelobacter carbinolicus (T); DSM 2380 Lineage=Root;rootrank;Bacteria;domain;"Proteobacteria";phylum;Deltaproteobacteria;class;Desulfuromonadales;order;Desulfuromonadaceae;family;Pelobacter;genus	83.65	2.00E-91	337
2921	0	0	0	1	2	0	0	1	0	Nitrospirae	Nitrospira	Nitrospirales			S000550678 Moorella thermoacetica; AMP Lineage=Root;rootrank;Bacteria;domain;Firmicutes;phylum;Clostridia;class;Thermoanaerobacterales;order;Thermoanaerobacteraceae;family;Moorella;genus	87.06	7.00E-116	418
1476	0	0	0	0	1	0	0	0	0	Nitrospirae	Nitrospira	Nitrospirales			S000721549 Gracilibacter thermotolerans; JW/YJL-S1; 5 Lineage=Root;rootrank;Bacteria;domain;Firmicutes;phylum;Clostridia;class;Clostridiales;order;Gracilibacteraceae;family;Gracilibacter;genus	86.49	1.00E-112	407
2096	0	0	7	3	1	1	0	0	0	NKB9					S000390565 Desulfomonile limimaris; DCB-F Lineage=Root;rootrank;Bacteria;domain;"Proteobacteria";phylum;Deltaproteobacteria;class;Syntrophobacterales;order;Syntrophaceae;family;Desulfomonile;genus	88.83	7.00E-126	451
183	0	0	0	1	0	0	0	0	0	NKB9					S000390565 Desulfomonile limimaris; DCB-F Lineage=Root;rootrank;Bacteria;domain;"Proteobacteria";phylum;Deltaproteobacteria;class;Syntrophobacterales;order;Syntrophaceae;family;Desulfomonile;genus	84.48	2.00E-106	387
3241	0	0	0	0	1	0	0	0	1	NKB9					S000390567 Thermolithobacter ferrireducens (T); KA2a Lineage=Root;rootrank;Bacteria;domain;Firmicutes;phylum;Thermolithobacteria;class;Thermolithobacterales;order;Thermolithobacteraceae;family;Thermolithobacter;genus	84.68	3.00E-105	383
1132	0	1	2	1	4	0	2	7	2	NKB9					S001152076 Thermaerobacter composti (T); Ni80 Lineage=Root;rootrank;Bacteria;domain;Firmicutes;phylum;Clostridia;class;Clostridiales;order;Clostridiales_Incertae Sedis XVII;family;Thermaerobacter;genus	85.67	9.00E-105	381
2024	0	0	0	0	0	0	0	1	0	NKB9					S001933336 Candidatus Methylomirabilis oxyfera Lineage=Root;rootrank;Bacteria;domain;unclassified_Bacteria;	85.79	1.00E-103	377
2425	0	1	0	0	0	0	0	0	0	OD					S000126206 Lineage=Root;rootrank;Bacteria;domain;unclassified_Bacteria;	81.17	3.00E-85	316
1292	0	2	0	0	0	0	0	0	0	OD					S000592754 Rubrivivax gelatinosus; 20 0D12 Lineage=Root;rootrank;Bacteria;domain;"Proteobacteria";phylum;Betaproteobacteria;class;Burkholderiales;order;unclassified_Burkholderiales;	79.52	4.00E-69	263
1083	0	0	0	1	0	0	0	0	0	OD					S001020446 Lobularia maritima (T) Lineage=Root;rootrank;Bacteria;domain;Cyanobacteria/Chloroplast;phylum;Chloroplast;class;Chloroplast;family;Streptophyta;genus	78.12	8.00E-61	235
3263	0	0	1	0	0	0	0	0	0	OP					S000083867 Desulfovibrio vietnamensis (T); type strain: G3 100; ID95-371 Lineage=Root;rootrank;Bacteria;domain;"Proteobacteria";phylum;Deltaproteobacteria;class;Desulfovibrionales;order;Desulfovibrionaceae;family;Desulfovibrio;genus	79.78	4.00E-68	259
207	0	0	0	1	1	0	0	0	0	OP					S000346778 Lineage=Root;rootrank;Bacteria;domain;unclassified_Bacteria;	81.28	2.00E-42	174
972	0	0	0	0	1	0	0	0	4	OP					S000995322 Desulfovibrio tunisiensis (T); RB22 Lineage=Root;rootrank;Bacteria;domain;"Proteobacteria";phylum;Deltaproteobacteria;class;Desulfovibrionales;order;Desulfovibrionaceae;family;Desulfovibrio;genus	82.11	6.00E-52	206
1525	0	0	0	0	0	0	0	0	2	OP					S001153278 Methylogaea oryzae JCM 16910; E10 Lineage=Root;rootrank;Bacteria;domain;"Proteobacteria";phylum;Gammaproteobacteria;class;Methylococcales;order;Methylococcaceae;family;Methylococcus;genus	78.99	6.00E-62	239
1349	0	0	0	0	0	0	0	0	4	OP					S001577989 Nocardia jejuensis Lineage=Root;rootrank;Bacteria;domain;"Actinobacteria";phylum;Actinobacteria;class;Actinobacteridae;subclass;Actinomycetales;order;Corynebacterineae;suborder;Nocardiaceae;family;Nocardia;genus	76	2.00E-46	187
1060	0	0	0	0	1	0	0	0	0	OP	OP-3				S000346037 Lineage=Root;rootrank;Bacteria;domain;unclassified_Bacteria;	76.41	2.00E-47	191
2742	0	0	0	1	0	0	0	0	0	OP	OP-4	KNA6-NB4			S000428040 candidate division OP11; from Lake Griffy freshwater sediment, Bloomington, IN; LGd6 Lineage=Root;rootrank;Bacteria;domain;unclassified_Bacteria;	80.93	4.00E-48	193
655	0	0	0	0	1	0	0	0	0	OP	OP-4	KNA6-NB4			S000428041 candidate division OP11; from Lake Griffy freshwater sediment, Bloomington, IN; LGd10 Lineage=Root;rootrank;Bacteria;domain;unclassified_Bacteria;	76.38	5.00E-48	193
827	0	0	0	0	0	0	0	2	0	OP	WCHB-64	d53			S000346037 Lineage=Root;rootrank;Bacteria;domain;unclassified_Bacteria;	84.1	2.00E-95	350
2304	0	0	0	0	0	0	0	0	1	OP					S001198587 thiotrophic endosymbiont of Adipicola crypta Lineage=Root;rootrank;Bacteria;domain;"Proteobacteria";phylum;Gammaproteobacteria;class;unclassified_Gammaproteobacteria;	78.7	5.00E-63	243
2412	0	0	0	0	1	0	0	0	0	OP3	koll				S002156824 Olsenella profusa; CCUG 45372 Lineage=Root;rootrank;Bacteria;domain;"Actinobacteria";phylum;Actinobacteria;class;Coriobacteridae;subclass;Coriobacteriales;order;"Coriobacterineae";suborder;Coriobacteriaceae;family;Olsenella;genus	85.61	6.00E-77	289
1343	0	0	1	0	0	0	0	0	0	OP3	koll				S002989305 Granulicella sapmiensis; S6CTX5A Lineage=Root;rootrank;Bacteria;domain;"Acidobacteria";phylum;Acidobacteria_Gp1;class;Granulicella;genus	81.33	2.00E-76	287
1569	0	0	0	0	1	0	0	0	0	OP3	koll	GIF0	kpj58rc		S002953735 Bathymodiolus heckerae gill symbiont; GoM_Chap_16S_1.1_cycloclasticus_symbiont Lineage=Root;rootrank;Bacteria;domain;"Proteobacteria";phylum;Gammaproteobacteria;class;Thiotrichales;order;Piscirickettsiaceae;family;Cycloclasticus;genus	78.83	8.00E-66	252
1501	0	0	0	1	0	0	0	0	0	OP3	koll				S001240928 Desulfovibrio oryzae; CINBIN-C5 Lineage=Root;rootrank;Bacteria;domain;"Proteobacteria";phylum;Deltaproteobacteria;class;Desulfovibrionales;order;Desulfovibrionaceae;family;Desulfovibrio;genus	81.12	5.00E-77	289
2242	0	0	0	0	0	1	0	0	0	OP3	koll				S001328494 Desulfonatronum thiosulfatophilum; ASO4-2 Lineage=Root;rootrank;Bacteria;domain;"Proteobacteria";phylum;Deltaproteobacteria;class;Desulfovibrionales;order;Desulfonatronaceae;family;Desulfonatronum;genus	82.35	7.00E-86	318
1947	0	0	0	2	1	0	0	0	0	OP3	PBS-25				S002483207 Maorithyas hadalis gill thioautotrophic symbiont II; SB1-1 Lineage=Root;rootrank;Bacteria;domain;"Proteobacteria";phylum;Gammaproteobacteria;class;Methylococcales;order;Methylococcaceae;family;unclassified_Methylococcaceae;	81.82	6.00E-87	322
1024	0	0	0	0	1	0	0	0	0	OP3	PBS-25				S001933336 Candidatus Methylomirabilis oxyfera Lineage=Root;rootrank;Bacteria;domain;unclassified_Bacteria;	81.99	4.00E-83	309
2753	0	0	0	0	1	0	0	0	0	OP3	PBS-25				S001933336 Candidatus Methylomirabilis oxyfera Lineage=Root;rootrank;Bacteria;domain;unclassified_Bacteria;	82.19	1.00E-89	331
2502	0	0	0	1	0	0	0	0	0	OP3	PBS-25				S002351528 Terriglobus saanensis; SP1PR5 Lineage=Root;rootrank;Bacteria;domain;"Acidobacteria";phylum;Acidobacteria_Gp1;class;Terriglobus;genus	82.69	2.00E-92	340
3004	0	0	0	1	0	0	0	0	0	OP3					S003264050 Frateuria terrea; NBRC 104236 Lineage=Root;rootrank;Bacteria;domain;"Proteobacteria";phylum;Gammaproteobacteria;class;Xanthomonadales;order;Xanthomonadaceae;family;Frateuria;genus	80.56	2.00E-76	287
103	0	1	0	0	0	0	0	0	0	OP9	JS	SB-45			S003259712 Syntrophothermus lipocalidus DSM 12680; TGB-C1 Lineage=Root;rootrank;Bacteria;domain;Firmicutes;phylum;Clostridia;class;Clostridiales;order;Syntrophomonadaceae;family;Syntrophothermus;genus	86.03	2.00E-106	387
865	0	0	0	0	0	0	0	0	1	PAUC34f					S000457292 Desulfuromonas svalbardensis; 60 Lineage=Root;rootrank;Bacteria;domain;"Proteobacteria";phylum;Deltaproteobacteria;class;Desulfuromonadales;order;Desulfuromonadaceae;family;Desulfuromonas;genus	84.91	7.00E-101	368
2798	0	0	0	0	3	5	2	0	1	Planctomycetes	agg27	CL500-5			S000389818 Gemmata-like str. CJuql4 Lineage=Root;rootrank;Bacteria;domain;"Planctomycetes";phylum;"Planctomycetacia";class;Planctomycetales;order;Planctomycetaceae;family;Gemmata;genus	84.89	4.00E-98	359
1686	0	0	0	1	1	0	0	0	0	Planctomycetes	agg27	CL500-5			S000389818 Gemmata-like str. CJuql4 Lineage=Root;rootrank;Bacteria;domain;"Planctomycetes";phylum;"Planctomycetacia";class;Planctomycetales;order;Planctomycetaceae;family;Gemmata;genus	85.31	4.00E-108	392
2853	0	0	0	1	1	0	0	0	0	Planctomycetes	agg27	CL500-5			S000871748 planctomycete Zi62 Lineage=Root;rootrank;Bacteria;domain;"Planctomycetes";phylum;"Planctomycetacia";class;Planctomycetales;order;Planctomycetaceae;family;Blastopirellula;genus	84.97	2.00E-100	366
290	0	0	0	0	0	1	1	0	0	Planctomycetes	agg27	CL500-5			S002952176 planctomycete MST324 Lineage=Root;rootrank;Bacteria;domain;"Planctomycetes";phylum;"Planctomycetacia";class;Planctomycetales;order;Planctomycetaceae;family;Blastopirellula;genus	84.02	4.00E-93	342
1285	0	0	0	2	0	1	0	0	0	Planctomycetes	agg27	CL500-5			S002952177 planctomycete MSF146 Lineage=Root;rootrank;Bacteria;domain;"Planctomycetes";phylum;"Planctomycetacia";class;Planctomycetales;order;Planctomycetaceae;family;Blastopirellula;genus	84.47	5.00E-97	355
2557	0	0	1	3	4	5	3	9	4	Planctomycetes	agg27	CL500-5			S002952191 planctomycete DDSe3021 Lineage=Root;rootrank;Bacteria;domain;"Planctomycetes";phylum;"Planctomycetacia";class;Planctomycetales;order;Planctomycetaceae;family;unclassified_Planctomycetaceae;	83.01	5.00E-87	322
2960	0	0	0	0	0	0	0	1	1	Planctomycetes	agg27	CL500-5			S002952201 planctomycete MSD101 Lineage=Root;rootrank;Bacteria;domain;"Planctomycetes";phylum;"Planctomycetacia";class;Planctomycetales;order;Planctomycetaceae;family;Rhodopirellula;genus	83.76	1.00E-98	361
1284	0	0	0	2	0	0	0	0	0	Planctomycetes	agg27	OM90			S000013736 anaerobic ammonium-oxidizing planctomycete; 14 Lineage=Root;rootrank;Bacteria;domain;unclassified_Bacteria;	84.07	1.00E-93	344
2855	0	0	0	1	0	0	0	0	0	Planctomycetes	agg27	OM90			S000383909 planctomycete KSU-1 Lineage=Root;rootrank;Bacteria;domain;unclassified_Bacteria;	83.15	7.00E-91	335
1740	0	0	0	1	1	1	0	0	0	Planctomycetes	agg27	OM90			S002447728 Candidatus Brocadia caroliniensis; NRRL_B-50286 Lineage=Root;rootrank;Bacteria;domain;unclassified_Bacteria;	82.45	1.00E-87	324
1872	0	0	0	1	0	0	0	0	0	Planctomycetes	agg27	OM90			S002950110 Desulfovibrio alaskensis G20 Lineage=Root;rootrank;Bacteria;domain;"Proteobacteria";phylum;Deltaproteobacteria;class;Desulfovibrionales;order;Desulfovibrionaceae;family;Desulfovibrio;genus	84.91	9.00E-100	364
1533	0	0	0	1	0	0	0	0	0	Planctomycetes	agg27	OM90			S002952179 planctomycete MS3332 Lineage=Root;rootrank;Bacteria;domain;"Planctomycetes";phylum;"Planctomycetacia";class;Planctomycetales;order;Planctomycetaceae;family;Blastopirellula;genus	82.64	9.00E-85	315
1589	0	0	2	7	2	1	0	0	0	Planctomycetes	agg27	OM90			S002952183 planctomycete DDSe3011 Lineage=Root;rootrank;Bacteria;domain;"Planctomycetes";phylum;"Planctomycetacia";class;Planctomycetales;order;Planctomycetaceae;family;Blastopirellula;genus	83.52	2.00E-90	333
3186	0	0	0	1	0	0	0	0	0	Planctomycetes	agg27	OM90			S002952188 planctomycete DDSW3022 Lineage=Root;rootrank;Bacteria;domain;"Planctomycetes";phylum;"Planctomycetacia";class;Planctomycetales;order;Planctomycetaceae;family;unclassified_Planctomycetaceae;	84.11	7.00E-101	368
523	0	0	0	5	2	0	1	4	4	Planctomycetes	C6				S000571483 planctomycete A-2 Lineage=Root;rootrank;Bacteria;domain;"Planctomycetes";phylum;"Planctomycetacia";class;Planctomycetales;order;Planctomycetaceae;family;unclassified_Planctomycetaceae;	89.54	3.00E-139	496
357	0	0	0	0	0	0	0	0	2	Planctomycetes	C6				S002952184 planctomycete DDSe3012 Lineage=Root;rootrank;Bacteria;domain;"Planctomycetes";phylum;"Planctomycetacia";class;Planctomycetales;order;Planctomycetaceae;family;unclassified_Planctomycetaceae;	83.01	3.00E-89	329
1953	0	0	0	1	0	0	0	0	0	Planctomycetes	FFCH393				S000653977 Candidatus Brocadia fulgida Lineage=Root;rootrank;Bacteria;domain;unclassified_Bacteria;	83.01	4.00E-88	326
2650	0	0	0	0	1	0	0	0	0	Planctomycetes	FFCH393				S000657470 Candidatus Kuenenia stuttgartiensis Lineage=Root;rootrank;Bacteria;domain;unclassified_Bacteria;	84.85	9.00E-100	364
2335	0	0	0	1	0	0	0	1	1	Planctomycetes	Kueneniae				S002447728 Candidatus Brocadia caroliniensis; NRRL_B-50286 Lineage=Root;rootrank;Bacteria;domain;unclassified_Bacteria;	86.77	7.00E-121	435
1271	0	0	0	3	0	0	0	0	3	Planctomycetes	Phycisphaerae				S000009676 Pelobacter acetylenicus (T); WoAcy1 DSM2348 Lineage=Root;rootrank;Bacteria;domain;"Proteobacteria";phylum;Deltaproteobacteria;class;Desulfuromonadales;order;Desulfuromonadaceae;family;Pelobacter;genus	81.7	5.00E-82	305
1023	0	0	0	1	0	0	0	0	0	Planctomycetes	Phycisphaerae				S000009676 Pelobacter acetylenicus (T); WoAcy1 DSM2348 Lineage=Root;rootrank;Bacteria;domain;"Proteobacteria";phylum;Deltaproteobacteria;class;Desulfuromonadales;order;Desulfuromonadaceae;family;Pelobacter;genus	81.02	1.00E-78	294
3153	0	0	0	18	2	0	0	0	0	Planctomycetes	Phycisphaerae				S000341318 Lineage=Root;rootrank;Bacteria;domain;unclassified_Bacteria;	82.81	5.00E-67	255
168	0	0	1	0	0	0	0	0	0	Planctomycetes	Phycisphaerae				S000389818 Gemmata-like str. CJuql4 Lineage=Root;rootrank;Bacteria;domain;"Planctomycetes";phylum;"Planctomycetacia";class;Planctomycetales;order;Planctomycetaceae;family;Gemmata;genus	81.07	1.00E-78	294
1326	0	0	0	1	0	0	0	0	0	Planctomycetes	Phycisphaerae				S000389818 Gemmata-like str. CJuql4 Lineage=Root;rootrank;Bacteria;domain;"Planctomycetes";phylum;"Planctomycetacia";class;Planctomycetales;order;Planctomycetaceae;family;Gemmata;genus	82.71	4.00E-88	326
1847	0	0	0	0	0	0	0	1	1	Planctomycetes	Phycisphaerae				S000389820 Gemmata-like str. JW10-3f1 Lineage=Root;rootrank;Bacteria;domain;"Planctomycetes";phylum;"Planctomycetacia";class;Planctomycetales;order;Planctomycetaceae;family;Zavarzinella;genus	78.71	1.00E-58	228
231	0	0	0	5	3	1	0	1	0	Planctomycetes	Phycisphaerae				S000571483 planctomycete A-2 Lineage=Root;rootrank;Bacteria;domain;"Planctomycetes";phylum;"Planctomycetacia";class;Planctomycetales;order;Planctomycetaceae;family;unclassified_Planctomycetaceae;	81.99	5.00E-82	305
702	0	0	0	6	2	0	0	0	0	Planctomycetes	Phycisphaerae				S000571483 planctomycete A-2 Lineage=Root;rootrank;Bacteria;domain;"Planctomycetes";phylum;"Planctomycetacia";class;Planctomycetales;order;Planctomycetaceae;family;unclassified_Planctomycetaceae;	83.08	3.00E-94	346
2475	0	1	3	0	0	0	0	0	0	Planctomycetes	Phycisphaerae				S000871748 planctomycete Zi62 Lineage=Root;rootrank;Bacteria;domain;"Planctomycetes";phylum;"Planctomycetacia";class;Planctomycetales;order;Planctomycetaceae;family;Blastopirellula;genus	81.34	2.00E-77	291
1445	0	0	0	2	0	0	0	0	0	Planctomycetes	Phycisphaerae				S002290918 Sorangium cellulosum 'So ce 56' Lineage=Root;rootrank;Bacteria;domain;"Proteobacteria";phylum;Deltaproteobacteria;class;Myxococcales;order;Sorangiineae;suborder;Polyangiaceae;family;Sorangium;genus	78.45	4.00E-64	246
645	0	0	0	1	1	0	0	0	2	Planctomycetes	Phycisphaerae				S002440613 Conexibacter arvalis; KV-962 Lineage=Root;rootrank;Bacteria;domain;"Actinobacteria";phylum;Actinobacteria;class;Rubrobacteridae;subclass;Solirubrobacterales;order;Conexibacteraceae;family;Conexibacter;genus	82.99	7.00E-96	351
2916	0	0	0	2	1	0	0	0	0	Planctomycetes	Phycisphaerae				S002440614 Conexibacter arvalis; KV-963 Lineage=Root;rootrank;Bacteria;domain;"Actinobacteria";phylum;Actinobacteria;class;Rubrobacteridae;subclass;Solirubrobacterales;order;Conexibacteraceae;family;Conexibacter;genus	80.22	9.00E-70	265
2285	0	4	6	1	1	2	1	3	0	Planctomycetes	Phycisphaerae				S002952177 planctomycete MSF146 Lineage=Root;rootrank;Bacteria;domain;"Planctomycetes";phylum;"Planctomycetacia";class;Planctomycetales;order;Planctomycetaceae;family;Blastopirellula;genus	79.4	5.00E-67	255
528	0	0	0	2	0	0	4	0	0	Planctomycetes	Phycisphaerae				S002952177 planctomycete MSF146 Lineage=Root;rootrank;Bacteria;domain;"Planctomycetes";phylum;"Planctomycetacia";class;Planctomycetales;order;Planctomycetaceae;family;Blastopirellula;genus	79.61	7.00E-66	252
1201	0	0	1	1	1	0	0	0	0	Planctomycetes	Phycisphaerae				S002952177 planctomycete MSF146 Lineage=Root;rootrank;Bacteria;domain;"Planctomycetes";phylum;"Planctomycetacia";class;Planctomycetales;order;Planctomycetaceae;family;Blastopirellula;genus	79.12	5.00E-68	259
491	0	0	1	0	0	0	0	0	0	Planctomycetes	Phycisphaerae				S002952177 planctomycete MSF146 Lineage=Root;rootrank;Bacteria;domain;"Planctomycetes";phylum;"Planctomycetacia";class;Planctomycetales;order;Planctomycetaceae;family;Blastopirellula;genus	80.61	2.00E-72	274
680	0	0	0	3	1	3	6	4	6	Planctomycetes	Phycisphaerae				S002952179 planctomycete MS3332 Lineage=Root;rootrank;Bacteria;domain;"Planctomycetes";phylum;"Planctomycetacia";class;Planctomycetales;order;Planctomycetaceae;family;Blastopirellula;genus	80	3.00E-69	263
465	9	33	3	2	1	2	1	0	1	Planctomycetes	Phycisphaerae				S002952188 planctomycete DDSW3022 Lineage=Root;rootrank;Bacteria;domain;"Planctomycetes";phylum;"Planctomycetacia";class;Planctomycetales;order;Planctomycetaceae;family;unclassified_Planctomycetaceae;	81.82	3.00E-79	296
223	0	0	0	0	0	0	0	5	8	Planctomycetes	Phycisphaerae				S002952222 planctomycete MS3039 Lineage=Root;rootrank;Bacteria;domain;"Planctomycetes";phylum;"Planctomycetacia";class;Planctomycetales;order;Planctomycetaceae;family;unclassified_Planctomycetaceae;	80.33	3.00E-70	267
3092	0	0	0	1	0	0	0	0	0	Planctomycetes	Phycisphaerae				S003259712 Syntrophothermus lipocalidus DSM 12680; TGB-C1 Lineage=Root;rootrank;Bacteria;domain;Firmicutes;phylum;Clostridia;class;Clostridiales;order;Syntrophomonadaceae;family;Syntrophothermus;genus	81.33	1.00E-78	294
809	0	0	0	1	0	0	0	0	0	Planctomycetes	Phycisphaerae	Phycisphaerales			S000015698 Desulfovibrio indonesiensis (T) Lineage=Root;rootrank;Bacteria;domain;"Proteobacteria";phylum;Deltaproteobacteria;class;Desulfovibrionales;order;Desulfovibrionaceae;family;Desulfovibrio;genus	79.65	2.00E-47	191
2826	1	0	2	1	0	0	1	0	1	Planctomycetes	Phycisphaerae	Phycisphaerales			S000016660 Blastopirellula marina (T); IFAM 1313 Lineage=Root;rootrank;Bacteria;domain;"Planctomycetes";phylum;"Planctomycetacia";class;Planctomycetales;order;Planctomycetaceae;family;Blastopirellula;genus	82.38	7.00E-81	302
2052	0	0	1	1	1	0	0	3	0	Planctomycetes	Phycisphaerae	Phycisphaerales			S000571483 planctomycete A-2 Lineage=Root;rootrank;Bacteria;domain;"Planctomycetes";phylum;"Planctomycetacia";class;Planctomycetales;order;Planctomycetaceae;family;unclassified_Planctomycetaceae;	81.64	1.00E-78	294

2894	0	0	0	0	3	0	0	0	0	Planctomycetes	Phycisphaerae	Phycisphaerales		S000571483 planctomycete A-2 Lineage=Root;rootrank;Bacteria;domain;"Planctomycetes";phylum;"Planctomycetacia";class;Planctomycetales;order;Planctomycetaceae;family;unclassified_Planctomycetaceae;	82.42	1.00E-83	311
2713	0	0	2	0	0	0	0	0	0	Planctomycetes	Phycisphaerae	Phycisphaerales		S002289094 Pirellula staleyi DSM 6068 Lineage=Root;rootrank;Bacteria;domain;Firmicutes;phylum;Bacilli;class;Bacillales;order;Pasteuriaceae;family;Pasteuria;genus	80.66	5.00E-72	272
469	0	0	0	0	1	0	0	0	0	Planctomycetes	Phycisphaerae	Phycisphaerales		S002289094 Pirellula staleyi DSM 6068 Lineage=Root;rootrank;Bacteria;domain;Firmicutes;phylum;Bacilli;class;Bacillales;order;Pasteuriaceae;family;Pasteuria;genus	79.25	9.00E-65	248
154	0	0	0	0	0	0	1	0	0	Planctomycetes	Phycisphaerae	Phycisphaerales		S002289094 Pirellula staleyi DSM 6068 Lineage=Root;rootrank;Bacteria;domain;Firmicutes;phylum;Bacilli;class;Bacillales;order;Pasteuriaceae;family;Pasteuria;genus	80	3.00E-70	267
2512	0	0	0	0	2	0	0	0	0	Planctomycetes	Phycisphaerae	Phycisphaerales		S002917589 planctomycete FF4 Lineage=Root;rootrank;Bacteria;domain;"Planctomycetes";phylum;"Planctomycetacia";class;Planctomycetales;order;Planctomycetaceae;family;Rhodopirellula;genus	80.78	6.00E-77	289
1240	0	0	0	3	0	0	0	0	1	Planctomycetes	Phycisphaerae	Phycisphaerales		S002952176 planctomycete MST324 Lineage=Root;rootrank;Bacteria;domain;"Planctomycetes";phylum;"Planctomycetacia";class;Planctomycetales;order;Planctomycetaceae;family;Blastopirellula;genus	80.28	1.00E-68	261
1348	11	22	0	0	0	1	0	0	0	Planctomycetes	Phycisphaerae	Phycisphaerales		S002952177 planctomycete MSF146 Lineage=Root;rootrank;Bacteria;domain;"Planctomycetes";phylum;"Planctomycetacia";class;Planctomycetales;order;Planctomycetaceae;family;Blastopirellula;genus	81.35	5.00E-77	289
1814	0	0	9	6	4	2	0	0	0	Planctomycetes	Phycisphaerae	Phycisphaerales		S002952177 planctomycete MSF146 Lineage=Root;rootrank;Bacteria;domain;"Planctomycetes";phylum;"Planctomycetacia";class;Planctomycetales;order;Planctomycetaceae;family;Blastopirellula;genus	80.71	1.00E-73	278
1598	0	0	1	9	2	0	0	4	1	Planctomycetes	Phycisphaerae	Phycisphaerales		S002952177 planctomycete MSF146 Lineage=Root;rootrank;Bacteria;domain;"Planctomycetes";phylum;"Planctomycetacia";class;Planctomycetales;order;Planctomycetaceae;family;Blastopirellula;genus	81.84	3.00E-80	300
1235	0	0	0	14	2	0	0	0	0	Planctomycetes	Phycisphaerae	Phycisphaerales		S002952177 planctomycete MSF146 Lineage=Root;rootrank;Bacteria;domain;"Planctomycetes";phylum;"Planctomycetacia";class;Planctomycetales;order;Planctomycetaceae;family;Blastopirellula;genus	80.81	1.00E-73	278
881	0	0	0	0	0	0	1	1	0	Planctomycetes	Phycisphaerae	Phycisphaerales		S002952177 planctomycete MSF146 Lineage=Root;rootrank;Bacteria;domain;"Planctomycetes";phylum;"Planctomycetacia";class;Planctomycetales;order;Planctomycetaceae;family;Blastopirellula;genus	81.44	5.00E-83	309
2574	0	0	2	20	21	0	2	1	2	Planctomycetes	Phycisphaerae	Phycisphaerales		S002952179 planctomycete MS3332 Lineage=Root;rootrank;Bacteria;domain;"Planctomycetes";phylum;"Planctomycetacia";class;Planctomycetales;order;Planctomycetaceae;family;Blastopirellula;genus	81.2	5.00E-77	289
2909	0	0	0	0	3	0	0	1	0	Planctomycetes	Phycisphaerae	Phycisphaerales		S002952179 planctomycete MS3332 Lineage=Root;rootrank;Bacteria;domain;"Planctomycetes";phylum;"Planctomycetacia";class;Planctomycetales;order;Planctomycetaceae;family;Blastopirellula;genus	79.62	5.00E-67	255
165	0	0	3	9	18	0	0	0	0	Planctomycetes	Phycisphaerae	Phycisphaerales		S002952195 planctomycete DDSe3017 Lineage=Root;rootrank;Bacteria;domain;unclassified_Bacteria;	79.28	3.00E-64	246
2484	2	15	0	1	0	0	1	0	0	Planctomycetes	Phycisphaerae	Phycisphaerales		S002952195 planctomycete DDSe3017 Lineage=Root;rootrank;Bacteria;domain;unclassified_Bacteria;	80.44	7.00E-71	268
409	0	0	0	1	0	0	1	0	0	Planctomycetes	Phycisphaerae	Phycisphaerales		S002952195 planctomycete DDSe3017 Lineage=Root;rootrank;Bacteria;domain;unclassified_Bacteria;	82.8	1.00E-88	327
2541	0	0	0	0	0	0	0	0	2	Planctomycetes	Phycisphaerae	Phycisphaerales		S002952195 planctomycete DDSe3017 Lineage=Root;rootrank;Bacteria;domain;unclassified_Bacteria;	81.56	3.00E-75	283
8	0	0	0	0	1	0	0	0	0	Planctomycetes	Phycisphaerae	Phycisphaerales		S002952195 planctomycete DDSe3017 Lineage=Root;rootrank;Bacteria;domain;unclassified_Bacteria;	82.08	7.00E-86	318
1799	0	0	0	1	0	0	0	0	0	Planctomycetes	Phycisphaerae	Phycisphaerales		S002952195 planctomycete DDSe3017 Lineage=Root;rootrank;Bacteria;domain;unclassified_Bacteria;	81.61	2.00E-82	307
475	0	0	0	1	0	0	0	0	0	Planctomycetes	Phycisphaerae	Phycisphaerales		S002952196 planctomycete DDSe3004 Lineage=Root;rootrank;Bacteria;domain;"Planctomycetes";phylum;"Planctomycetacia";class;Planctomycetales;order;Planctomycetaceae;family;Rhodopirellula;genus	82.01	1.00E-83	311
1809	0	0	0	1	0	0	0	0	0	Planctomycetes	Phycisphaerae	Phycisphaerales		S002952196 planctomycete DDSe3004 Lineage=Root;rootrank;Bacteria;domain;"Planctomycetes";phylum;"Planctomycetacia";class;Planctomycetales;order;Planctomycetaceae;family;Rhodopirellula;genus	80.21	5.00E-73	276
1114	0	0	0	0	0	0	0	1	0	Planctomycetes	Phycisphaerae	Phycisphaerales		S002952197 planctomycete DDSW3008 Lineage=Root;rootrank;Bacteria;domain;"Planctomycetes";phylum;"Planctomycetacia";class;Planctomycetales;order;Planctomycetaceae;family;Rhodopirellula;genus	81.14	1.00E-79	298
647	0	0	0	1	1	0	0	0	0	Planctomycetes	Phycisphaerae	Phycisphaerales		S002952199 planctomycete MSL156 Lineage=Root;rootrank;Bacteria;domain;"Planctomycetes";phylum;"Planctomycetacia";class;Planctomycetales;order;Planctomycetaceae;family;Rhodopirellula;genus	79.79	2.00E-72	274
1377	0	0	0	0	0	0	0	1	0	Planctomycetes	Phycisphaerae	Phycisphaerales		S003288169 Blastopirellula marina; type strain: DSM 3645 Lineage=Root;rootrank;Bacteria;domain;"Planctomycetes";phylum;"Planctomycetacia";class;Planctomycetales;order;Planctomycetaceae;family;Blastopirellula;genus	81.79	9.00E-80	298
897	0	0	1	0	0	0	0	1	0	Planctomycetes	Phycisphaerae	Phycisphaerales	Phycisphaeraceae	S000017510 planctomycete str. 610 Lineage=Root;rootrank;Bacteria;domain;"Planctomycetes";phylum;"Planctomycetacia";class;Planctomycetales;order;Planctomycetaceae;family;Rhodopirellula;genus	80.2	1.00E-74	281
1618	2	7	1	0	0	4	8	1	0	Planctomycetes	Phycisphaerae	Phycisphaerales	Phycisphaeraceae	S000654143 Thiohalorhabdus denitrificans (T); HL 19 Lineage=Root;rootrank;Bacteria;domain;"Proteobacteria";phylum;Gammaproteobacteria;class;Gammaproteobacteria_incertae_sedis;order;Thiohalorhabdus;genus	78.99	3.00E-64	246
2558	0	0	0	0	0	0	0	3	10	Planctomycetes	Phycisphaerae	Phycisphaerales	Phycisphaeraceae	S000995807 Thiohalorhabdus denitrificans; HLD 10 Lineage=Root;rootrank;Bacteria;domain;"Proteobacteria";phylum;Gammaproteobacteria;class;Gammaproteobacteria_incertae_sedis;order;Thiohalorhabdus;genus	79.27	2.00E-62	241
1834	25	50	9	2	1	4	4	2	2	Planctomycetes	Phycisphaerae	Phycisphaerales	Phycisphaeraceae	S001152076 Thermaerobacter composti (T); Ni80 Lineage=Root;rootrank;Bacteria;domain;Firmicutes;phylum;Clostridia;class;Clostridiales;order;Clostridiales_Incertae Sedis XVII;family;Thermaerobacter;genus	77.84	6.00E-57	222
1566	0	0	0	0	0	9	16	5	1	Planctomycetes	Phycisphaerae	Phycisphaerales	Phycisphaeraceae	S001152076 Thermaerobacter composti (T); Ni80 Lineage=Root;rootrank;Bacteria;domain;Firmicutes;phylum;Clostridia;class;Clostridiales;order;Clostridiales_Incertae Sedis XVII;family;Thermaerobacter;genus	78.82	3.00E-65	250
559	2	1	0	0	0	0	0	0	0	Planctomycetes	Phycisphaerae	Phycisphaerales	Phycisphaeraceae	S001152076 Thermaerobacter composti (T); Ni80 Lineage=Root;rootrank;Bacteria;domain;Firmicutes;phylum;Clostridia;class;Clostridiales;order;Clostridiales_Incertae Sedis XVII;family;Thermaerobacter;genus	79.5	5.00E-67	255
564	0	1	0	0	0	0	0	0	0	Planctomycetes	Phycisphaerae	Phycisphaerales	Phycisphaeraceae	S001612658 Nevskia terrae (T); KIS13-15 Lineage=Root;rootrank;Bacteria;domain;"Proteobacteria";phylum;Gammaproteobacteria;class;Xanthomonadales;order;Sinobacteraceae;family;Nevskia;genus	79.89	8.00E-71	268
376	1	14	7	0	0	3	10	0	0	Planctomycetes	Phycisphaerae	Phycisphaerales	Phycisphaeraceae	S002290651 Coraliomargarita akajimensis DSM 45221 (T) Lineage=Root;rootrank;Bacteria;domain;"Verrucomicrobia";phylum;Opitutae;class;Puniceicoccales;order;Puniceicoccaceae;family;Coraliomargarita;genus	78.96	4.00E-63	243
481	2	1	8	0	5	1	8	0	0	Planctomycetes	Phycisphaerae	Phycisphaerales	Phycisphaeraceae	S002952176 planctomycete MST324 Lineage=Root;rootrank;Bacteria;domain;"Planctomycetes";phylum;"Planctomycetacia";class;Planctomycetales;order;Planctomycetaceae;family;Blastopirellula;genus	78.9	4.00E-63	243
2872	0	0	0	1	0	0	0	0	0	Planctomycetes	Phycisphaerae	Phycisphaerales	Phycisphaeraceae	S002952176 planctomycete MST324 Lineage=Root;rootrank;Bacteria;domain;"Planctomycetes";phylum;"Planctomycetacia";class;Planctomycetales;order;Planctomycetaceae;family;Blastopirellula;genus	79.23	3.00E-65	250
312	0	0	0	0	0	0	0	1	0	Planctomycetes	Phycisphaerae	Phycisphaerales	Phycisphaeraceae	S002952176 planctomycete MST324 Lineage=Root;rootrank;Bacteria;domain;"Planctomycetes";phylum;"Planctomycetacia";class;Planctomycetales;order;Planctomycetaceae;family;Blastopirellula;genus	80.61	2.00E-72	274
575	2	12	1	0	1	0	0	0	0	Planctomycetes	Phycisphaerae	Phycisphaerales	Phycisphaeraceae	S002952177 planctomycete MSF146 Lineage=Root;rootrank;Bacteria;domain;"Planctomycetes";phylum;"Planctomycetacia";class;Planctomycetales;order;Planctomycetaceae;family;Blastopirellula;genus	81.32	1.00E-78	294
802	0	0	2	1	1	0	1	1	4	Planctomycetes	Phycisphaerae	Phycisphaerales	Phycisphaeraceae	S002952177 planctomycete MSF146 Lineage=Root;rootrank;Bacteria;domain;"Planctomycetes";phylum;"Planctomycetacia";class;Planctomycetales;order;Planctomycetaceae;family;Blastopirellula;genus	78.98	6.00E-62	239
3231	175	165	46	4	11	31	60	14	4	Planctomycetes	Phycisphaerae	Phycisphaerales	Phycisphaeraceae	S002952181 planctomycete DSM1A9 Lineage=Root;rootrank;Bacteria;domain;"Planctomycetes";phylum;"Planctomycetacia";class;Planctomycetales;order;Planctomycetaceae;family;Blastopirellula;genus	79.61	3.00E-64	246
1320	5	15	5	4	6	1	2	8	4	Planctomycetes	Phycisphaerae	Phycisphaerales	Phycisphaeraceae	S002952181 planctomycete DSM1A9 Lineage=Root;rootrank;Bacteria;domain;"Planctomycetes";phylum;"Planctomycetacia";class;Planctomycetales;order;Planctomycetaceae;family;Blastopirellula;genus	79.02	4.00E-63	243
3047	5	4	4	0	0	0	2	0	0	Planctomycetes	Phycisphaerae	Phycisphaerales	Phycisphaeraceae	S002952195 planctomycete DDSe3017 Lineage=Root;rootrank;Bacteria;domain;unclassified_Bacteria;	78.79	7.00E-61	235
3042	3	6	2	0	0	1	0	1	0	Planctomycetes	Phycisphaerae	Phycisphaerales	Phycisphaeraceae	S002952195 planctomycete DDSe3017 Lineage=Root;rootrank;Bacteria;domain;unclassified_Bacteria;	85.26	8.00E-66	252
359	0	0	0	0	0	4	1	0	0	Planctomycetes	Phycisphaerae	Phycisphaerales	Phycisphaeraceae	S002952195 planctomycete DDSe3017 Lineage=Root;rootrank;Bacteria;domain;unclassified_Bacteria;	77.75	2.00E-52	207
482	2	9	8	0	4	1	10	0	0	Planctomycetes	Phycisphaerae	Phycisphaerales	Phycisphaeraceae	S002952196 planctomycete DDSe3004 Lineage=Root;rootrank;Bacteria;domain;"Planctomycetes";phylum;"Planctomycetacia";class;Planctomycetales;order;Planctomycetaceae;family;Rhodopirellula;genus	79.72	2.00E-67	257
1757	0	0	0	1	0	0	1	1	1	Planctomycetes	Phycisphaerae	Phycisphaerales	Phycisphaeraceae	S002952196 planctomycete DDSe3004 Lineage=Root;rootrank;Bacteria;domain;"Planctomycetes";phylum;"Planctomycetacia";class;Planctomycetales;order;Planctomycetaceae;family;Rhodopirellula;genus	80.73	3.00E-70	267
1074	0	1	1	0	0	0	0	0	0	Planctomycetes	Phycisphaerae	Phycisphaerales	Phycisphaeraceae	S002952196 planctomycete DDSe3004 Lineage=Root;rootrank;Bacteria;domain;"Planctomycetes";phylum;"Planctomycetacia";class;Planctomycetales;order;Planctomycetaceae;family;Rhodopirellula;genus	79.13	1.00E-63	244
834	0	4	0	0	0	0	0	0	0	Planctomycetes	Phycisphaerae	Phycisphaerales	Phycisphaeraceae	S002952201 planctomycete MSD101 Lineage=Root;rootrank;Bacteria;domain;"Planctomycetes";phylum;"Planctomycetacia";class;Planctomycetales;order;Planctomycetaceae;family;Rhodopirellula;genus	79.51	5.00E-67	255
1735	0	1	0	0	0	0	0	0	0	Planctomycetes	Phycisphaerae	Phycisphaerales	Phycisphaeraceae	S002952202 planctomycete MS3057 Lineage=Root;rootrank;Bacteria;domain;"Planctomycetes";phylum;"Planctomycetacia";class;Planctomycetales;order;Planctomycetaceae;family;Rhodopirellula;genus	80	5.00E-67	255
1932	0	0	0	0	0	0	0	2	1	Planctomycetes	Phycisphaerae	Phycisphaerales	Phycisphaeraceae	S002952219 planctomycete DDSW1318 Lineage=Root;rootrank;Bacteria;domain;"Planctomycetes";phylum;"Planctomycetacia";class;Planctomycetales;order;Planctomycetaceae;family;Planctomyces;genus	78.33	3.00E-55	217
1768	0	0	0	0	2	0	0	2	4	Planctomycetes	Phycisphaerae	Phycisphaerales	Phycisphaeraceae	S003288169 Blastopirellula marina; type strain: DSM 3645 Lineage=Root;rootrank;Bacteria;domain;"Planctomycetes";phylum;"Planctomycetacia";class;Planctomycetales;order;Planctomycetaceae;family;Blastopirellula;genus	79.84	6.00E-72	272
1133	0	1	0	0	0	0	0	0	0	Planctomycetes	Phycisphaerae	Phycisphaerales	Phycisphaeraceae	S003288169 Blastopirellula marina; type strain: DSM 3645 Lineage=Root;rootrank;Bacteria;domain;"Planctomycetes";phylum;"Planctomycetacia";class;Planctomycetales;order;Planctomycetaceae;family;Blastopirellula;genus	79.89	4.00E-68	259
108	0	0	0	0	0	0	0	1	0	Planctomycetes	Phycisphaerae	Phycisphaerales		S000022803 planctomycete str. 292 Lineage=Root;rootrank;Bacteria;domain;"Planctomycetes";phylum;"Planctomycetacia";class;Planctomycetales;order;Planctomycetaceae;family;Blastopirellula;genus	80.41	3.00E-75	283
1105	1	5	2	4	2	0	4	1	1	Planctomycetes	Phycisphaerae	Phycisphaerales		S000356845 Petrobacter succinatimandens (T); BON4 Lineage=Root;rootrank;Bacteria;domain;"Proteobacteria";phylum;Betaproteobacteria;class;Hydrogenophilales;order;Hydrogenophilaceae;family;Petrobacter;genus	84.25	4.00E-73	276
518	0	0	12	0	4	3	5	0	2	Planctomycetes	Phycisphaerae	Phycisphaerales		S000570461 Marinobacter maritimus (T); type strain: CK47 Lineage=Root;rootrank;Bacteria;domain;"Proteobacteria";phylum;Gammaproteobacteria;class;Alteromonadales;order;Alteromonadaceae;family;Marinobacter;genus	78.15	2.00E-61	237
366	120	190	4	1	0	14	4	1	1	Planctomycetes	Phycisphaerae	Phycisphaerales		S000571483 planctomycete A-2 Lineage=Root;rootrank;Bacteria;domain;"Planctomycetes";phylum;"Planctomycetacia";class;Planctomycetales;order;Planctomycetaceae;family;unclassified_Planctomycetaceae;	80.28	1.00E-68	261
857	0	8	7	0	7	27	67	2	0	Planctomycetes	Phycisphaerae	Phycisphaerales		S000571483 planctomycete A-2 Lineage=Root;rootrank;Bacteria;domain;"Planctomycetes";phylum;"Planctomycetacia";class;Planctomycetales;order;Planctomycetaceae;family;unclassified_Planctomycetaceae;	80.17	9.00E-70	265
2172	0	9	6	0	0	4	2	1	0	Planctomycetes	Phycisphaerae	Phycisphaerales		S000571483 planctomycete A-2 Lineage=Root;rootrank;Bacteria;domain;"Planctomycetes";phylum;"Planctomycetacia";class;Planctomycetales;order;Planctomycetaceae;family;unclassified_Planctomycetaceae;	80.28	1.00E-68	261
1167	0	0	0	2	0	0	1	0	0	Planctomycetes	Phycisphaerae	Phycisphaerales		S000571483 planctomycete A-2 Lineage=Root;rootrank;Bacteria;domain;"Planctomycetes";phylum;"Planctomycetacia";class;Planctomycetales;order;Planctomycetaceae;family;unclassified_Planctomycetaceae;	82.69	2.00E-91	337
3025	0	1	1	0	0	0	0	0	0	Planctomycetes	Phycisphaerae	Phycisphaerales		S000571483 planctomycete A-2 Lineage=Root;rootrank;Bacteria;domain;"Planctomycetes";phylum;"Planctomycetacia";class;Planctomycetales;order;Planctomycetaceae;family;unclassified_Planctomycetaceae;	81.41	3.00E-75	283
122	0	0	0	0	0	0	0	2	0	Planctomycetes	Phycisphaerae	Phycisphaerales		S000571483 planctomycete A-2 Lineage=Root;rootrank;Bacteria;domain;"Planctomycetes";phylum;"Planctomycetacia";class;Planctomycetales;order;Planctomycetaceae;family;unclassified_Planctomycetaceae;	82.91	9.00E-65	248
1964	0	0	0	1	0	0	0	0	0	Planctomycetes	Phycisphaerae	Phycisphaerales		S000571483 planctomycete A-2 Lineage=Root;rootrank;Bacteria;domain;"Planctomycetes";phylum;"Planctomycetacia";class;Planctomycetales;order;Planctomycetaceae;family;unclassified_Planctomycetaceae;	82.24	4.00E-83	309
454	0	0	0	1	0	0	0	0	0	Planctomycetes	Phycisphaerae	Phycisphaerales		S000571483 planctomycete A-2 Lineage=Root;rootrank;Bacteria;domain;"Planctomycetes";phylum;"Planctomycetacia";class;Planctomycetales;order;Planctomycetaceae;family;unclassified_Planctomycetaceae;	80.27	3.00E-70	267
2664	0	0	0	1	0	0	0	0	0	Planctomycetes	Phycisphaerae	Phycisphaerales		S000571483 planctomycete A-2 Lineage=Root;rootrank;Bacteria;domain;"Planctomycetes";phylum;"Planctomycetacia";class;Planctomycetales;order;Planctomycetaceae;family;unclassified_Planctomycetaceae;	80.82	9.00E-75	281
2822	0	0	0	1	0	0	0	0	0	Planctomycetes	Phycisphaerae	Phycisphaerales		S000571483 planctomycete A-2 Lineage=Root;rootrank;Bacteria;domain;"Planctomycetes";phylum;"Planctomycetacia";class;Planctomycetales;order;Planctomycetaceae;family;unclassified_Planctomycetaceae;	82.47	3.00E-84	313
2035	0	0	0	0	0	0	0	0	1	Planctomycetes	Phycisphaerae	Phycisphaerales		S000571483 planctomycete A-2 Lineage=Root;rootrank;Bacteria;domain;"Planctomycetes";phylum;"Planctomycetacia";class;Planctomycetales;order;Planctomycetaceae;family;unclassified_Planctomycetaceae;	80.33	3.00E-70	267
1899	0	0	0	0	0	0	1	0	0	Planctomycetes	Phycisphaerae	Phycisphaerales		S000654143 Thiohalorhabdus denitrificans (T); HL 19 Lineage=Root;rootrank;Bacteria;domain;"Proteobacteria";phylum;Gammaproteobacteria;class;Gammaproteobacteria_incertae_sedis;order;Thiohalorhabdus;genus	81.4	6.00E-82	305
1605	1	0	0	0	0	0	0	0	0	Planctomycetes	Phycisphaerae	Phycisphaerales		S000769899 Lentimonas marisflavi; IMCC2112 Lineage=Root;rootrank;Bacteria;domain;"Verrucomicrobia";phylum;Opitutae;class;Puniceicoccales;order;Puniceicoccaceae;family;Coraliomargarita;genus	81.4	2.00E-81	303
1440	0	0	0	3	0	0	1	0	0	Planctomycetes	Phycisphaerae	Phycisphaerales		S000871748 planctomycete Zi62 Lineage=Root;rootrank;Bacteria;domain;"Planctomycetes";phylum;"Planctomycetacia";class;Planctomycetales;order;Planctomycetaceae;family;Blastopirellula;genus	82.51	1.00E-88	327
2761	33	222	138	2	14	35	38	33	19	Planctomycetes	Phycisphaerae	Phycisphaerales		S001155619 Halothiobacillus neapolitanus; S-A Lineage=Root;rootrank;Bacteria;domain;"Proteobacteria";phylum;Gammaproteobacteria;class;Chromatiales;order;Halothiobacillaceae;family;unclassified_Halothiobacillaceae;	79.73	1.00E-68	261
1933	0	1	0	0	0	0	0	0	0	Planctomycetes	Phycisphaerae	Phycisphaerales		S001155619 Halothiobacillus neapolitanus; S-A Lineage=Root;rootrank;Bacteria;domain;"Proteobacteria";phylum;Gammaproteobacteria;class;Chromatiales;order;Halothiobacillaceae;family;unclassified_Halothiobacillaceae;	79.48	3.00E-70	267
1850	0	1	1	2	1	0	2	3	1	Planctomycetes	Phycisphaerae	Phycisphaerales		S001612658 Nevskia terrae (T); KIS13-15 Lineage=Root;rootrank;Bacteria;domain;"Proteobacteria";phylum;Gammaproteobacteria;class;Xanthomonadales;order;Sinobacteraceae;family;Nevskia;genus	78.93	3.00E-65	250
589	0	0	0	0	0	0	0	4	2	Planctomycetes	Phycisphaerae	Phycisphaerales		S001612980 Gilvimarinus agarilyticus; M5c Lineage=Root;rootrank;Bacteria;domain;"Proteobacteria";phylum;Gammaproteobacteria;class;Gammaproteobacteria_incertae_sedis;order;Gilvimarinus;genus	79.39	2.00E-62	241
2189	0	0	0	1	0	0	0	0	0	Planctomycetes	Phycisphaerae	Phycisphaerales		S002288919 Halothermothrix orenii H 168; H 168; DSM 9562 Lineage=Root;rootrank;Bacteria;domain;Firmicutes;phylum;Clostridia;class;Halanaerobiales;order;Halanaerobiaceae;family;Halothermothrix;genus	78.32	1.00E-64	248
594	0	3	0	0	0	0	0	0	0	Planctomycetes	Phycisphaerae	Phycisphaerales		S002290518 Desulfovibrio salexigens (T); DSM 2638 Lineage=Root;rootrank;Bacteria;domain;"Proteobacteria";phylum;Deltaproteobacteria;class;Desulfovibrionales;order;Desulfovibrionaceae;family;Desulfovibrio;genus	79.67	2.00E-66	254
981	0	0	0	0	1	0	0	0	0	Planctomycetes	Phycisphaerae	Phycisphaerales		S002440613 Conexibacter arvalis; KV-962 Lineage=Root;rootrank;Bacteria;domain;"Actinobacteria";phylum;Actinobacteria;class;Rubrobacteridae;subclass;Solirubrobacterales;order;Conexibacteraceae;family;Conexibacter;genus	82.14	3.00E-89	329
2880	0	2	0	0	0	1	1	0	0	Planctomycetes	Phycisphaerae	Phycisphaerales		S002952177 planctomycete MSF146 Lineage=Root;rootrank;Bacteria;domain;"Planctomycetes";phylum;"Planctomycetacia";class;Planctomycetales;order;Planctomycetaceae;family;Blastopirellula;genus	79.64	5.00E-73	276
1228	0	0	0	0	1	0	0	0	0	Planctomycetes	Phycisphaerae	Phycisphaerales		S002952177 planctomycete MSF146 Lineage=Root;rootrank;Bacteria;domain;"Planctomycetes";phylum;"Planctomycetacia";class;Planctomycetales;order;Planctomycetaceae;family;Blastopirellula;genus	80.37	5.00E-73	276
17	0	0	0	0	0	1	0	0	0	Planctomycetes	Phycisphaerae	Phycisphaerales		S002952177 planctomycete MSF146 Lineage=Root;rootrank;Bacteria;domain;"Planctomycetes";phylum;"Planctomycetacia";class;Planctomycetales;order;Planctomycetaceae;family;Blastopirellula;genus	81.4	5.00E-83	309
1459	7	34	4	1	1	4	4	1	1	Planctomycetes	Phycisphaerae	Phycisphaerales		S002952179 planctomycete MS3332 Lineage=Root;rootrank;Bacteria;domain;"Planctomycetes";phylum;"Planctomycetacia";class;Planctomycetales;order;Planctomycetaceae;family;Blastopirellula;genus	79.95	1.00E-68	261
243	0	4	1	0	2	4	0	3	0	Planctomycetes	Phycisphaerae	Phycisphaerales		S002952195 planctomycete DDSe3017 Lineage=Root;rootrank;Bacteria;domain;unclassified_Bacteria;	77.87	2.00E-56	220
2327	3	0	2	2	0	1	3	1	0	Planctomycetes	Phycisphaerae	Phycisphaerales		S002952202 planctomycete MS3057 Lineage=Root;rootrank;Bacteria;domain;"Planctomycetes";phylum;"Planctomycetacia";class;Planctomycetales;order;Planctomycetaceae;family;Rhodopirellula;genus	78.47	1.00E-58	228
558	0	1	0	0	0	0	0	0	0	Planctomycetes	Phycisphaerae	Phycisphaerales		S002952218 planctomycete MS3054 Lineage=Root;rootrank;Bacteria;domain;"Planctomycetes";phylum;"Planctomycetacia";class;Planctomycetales;order;Planctomycetaceae;family;Planctomyces;genus	77.89	1.00E-59	231
1109	0	0	0	1	0	0	0	0	0	Planctomycetes	Phycisphaerae	Phycisphaerales		S002989144 Stenotrophomonas maltophilia; SO57 Lineage=Root;rootrank;Bacteria;domain;"Proteobacteria";phylum;Gammaproteobacteria;class;Xanthomonadales;order;Xanthomonadaceae;family;Stenotrophomonas;genus	81.03	2.00E-76	287
642	0	0	2	2	0	16	44	29	41	Planctomycetes	Phycisphaerae	Phycisphaerales		S003264308 Nevskia soli; NBRC 106401 Lineage=Root;rootrank;Bacteria;domain;"Proteobacteria";phylum;Gammaproteobacteria;class;Xanthomonadales;order;Sinobacteraceae;family;Nevskia;genus	80.32	5.00E-72	272
116	0	0	1	0	0	0	0	0	0	Planctomycetes	Phycisphaerae	Phycisphaerales		S003288169 Blastopirellula marina; type strain: DSM 3645 Lineage=Root;rootrank;Bacteria;domain;"Planctomycetes";phylum;"Planctomycetacia";class;Planctomycetales;order;Planctomycetaceae;family;Blastopirellula;genus	79.64	5.00E-73	276
2130	0	0	0	0	1	0	0	0	0	Planctomycetes	Phycisphaerae	Phycisphaerales		S003288169 Blastopirellula marina; type strain: DSM 3645 Lineage=Root;rootrank;Bacteria;domain;"Planctomycetes";phylum;"Planctomycetacia";class;Planctomycetales;order;Planctomycetaceae;family;Blastopirellula;genus	81.51	5.00E-83	309
522	0	0	0	1	0	0	0	0	0	Planctomycetes	Phycisphaerae			S000356845 Petrobacter succinatimandens (T); BON4 Lineage=Root;rootrank;Bacteria;domain;"Proteobacteria";phylum;Betaproteobacteria;class;Hydrogenophilales;order;Hydrogenophilaceae;family;Petrobacter;genus	80.36	1.00E-74	281
2535	0	1	18	0	9	18	18	44	3	Planctomycetes	Phycisphaerae			S000571483 planctomycete A-2 Lineage=Root;rootrank;Bacteria;domain;"Planctomycetes";phylum;"Planctomycetacia";class;Planctomycetales;order;Planctomycetaceae;family;unclassified_Planctomycetaceae;	82.42	1.00E-83	311
1867	7	12	0	7	1	0	0	0	1	Planctomycetes	Phycisphaerae			S000871748 planctomycete Zi62 Lineage=Root;rootrank;Bacteria;domain;"Planctomycetes";phylum;"Planctomycetacia";class;Planctomycetales;order;Planctomycetaceae;family;Blastopirellula;genus	82.03	2.00E-86	320
128	0	0	0	0	0	0	1	0	0	Planctomycetes	Phycisphaerae			S002952193 planctomycete MS1316 Lineage=Root;rootrank;Bacteria;domain;"Planctomycetes";phylum;"Planctomycetacia";class;Planctomycetales;order;Planctomycetaceae;family;unclassified_Planctomycetaceae;	79.37	2.00E-66	254
2157	0	0	1	1	9	0	0	0	3	Planctomycetes	Planctomycea	Gemmatales	Gemmataceae	S000128801 Gemmata obscuriglobus; ACM 2246 Lineage=Root;rootrank;Bacteria;domain;"Planctomycetes";phylum;"Planctomycetacia";class;Planctomycetales;order;Planctomycetaceae;family;Gemmata;genus	88.74	2.00E-126	453
3023	0	0	0	0	0	0	0	5	9	Planctomycetes	Planctomycea	Gemmatales	Gemmataceae	S000128801 Gemmata obscuriglobus; ACM 2246 Lineage=Root;rootrank;Bacteria;domain;"Planctomycetes";phylum;"Planctomycetacia";class;Planctomycetales;order;Planctomycetaceae;family;Gemmata;genus	83.24	2.00E-91	337
602	0	0	0	0	1	2	2	0	0	Planctomycetes	Planctomycea	Gemmatales	Gemmataceae	S000128801 Gemmata obscuriglobus; ACM 2246 Lineage=Root;rootrank;Bacteria;domain;"Planctomycetes";phylum;"Planctomycetacia";class;Planctomycetales;order;Planctomycetaceae;family;Gemmata;genus	88.44	8.00E-125	448
1998	0	0	0	0	1	1	0	0	3	Planctomycetes	Planctomycea	Gemmatales	Gemmataceae	S000128801 Gemmata obscuriglobus; ACM 2246 Lineage=Root;rootrank;Bacteria;domain;"Planctomycetes";phylum;"Planctomycetacia";class;Planctomycetales;order;Planctomycetaceae;family;Gemmata;genus	88.95	4.00E-128	459
52	0	0	0	1	1	0	0	0	0	Planctomycetes	Planctomycea	Gemmatales	Gemmataceae	S000128801 Gemmata obscuriglobus; ACM 2246 Lineage=Root;rootrank;Bacteria;domain;"Planctomycetes";phylum;"Planctomycetacia";class;Planctomycetales;order;Planctomycetaceae;family;Gemmata;genus	90.05	8.00E-135	481
2992	0	0	0	0	0	1	0	0	0	Planctomycetes	Planctomycea	Gemmatales	Gemmataceae	S000128801 Gemmata obscuriglobus; ACM 2246 Lineage=Root;rootrank;Bacteria;domain;"Planctomycetes";phylum;"Planctomycetacia";class;Planctomycetales;order;Planctomycetaceae;family;Gemmata;genus	88.74	6.00E-126	451
1591	0	0	0	0	0	0	0	0	1	Planctomycetes	Planctomycea	Gemmatales	Gemmataceae	S000128801 Gemmata obscuriglobus; ACM 2246 Lineage=Root;rootrank;Bacteria;domain;"Planctomycetes";phylum;"Planctomycetacia";class;Planctomycetales;order;Planctomycetaceae;family;Gemmata;genus	86.67	1.00E-112	407
3027	0	0	0	18	1	0	0	0	0	Planctomycetes	Planctomycea	Gemmatales	Gemmataceae	S000389818 Gemmata-like str. CJuql4 Lineage=Root;rootrank;Bacteria;domain;"Planctomycetes";phylum;"Planctomycetacia";class;Planctomycetales;order;Planctomycetaceae;family;Gemmata;genus	86.83	9.00E-115	414
2773	0	0	0	2	0	1	2	2	3	Planctomycetes	Planctomycea	Gemmatales	Gemmataceae	S000389818 Gemmata-like str. CJuql4 Lineage=Root;rootrank;Bacteria;domain;"Planctomycetes";phylum;"Planctomycetacia";class;Planctomycetales;order;Planctomycetaceae;family;Gemmata;genus	86.25	7.00E-111	401
2010	0	0	0	0	2	3	1	0	0	Planctomycetes	Planctomycea	Gemmatales	Gemmataceae	S000389818 Gemmata-like str. CJuql4 Lineage=Root;rootrank;Bacteria;domain;"Planctomycetes";phylum;"Planctomycetacia";class;Planctomycetales;order;Planctomycetaceae;family;Gemmata;genus	89.25	8.00E-130	464
2411	0	0	0	2	0	0	0	0	0	Planctomycetes	Planctomycea	Gemmatales	Gemmataceae	S000389818 Gemmata-like str. CJuql4 Lineage=Root;rootrank;Bacteria;domain;"Planctomycetes";phylum;"Planctomycetacia";class;Planctomycetales;order;Planctomycetaceae;family;Gemmata;genus	87.97	2.00E-121	436
2215	0	0	0	0	1	0	0	0	0	Planctomycetes	Planctomycea	Gemmatales	Gemmataceae	S000389818 Gemmata-like str. CJuql4 Lineage=Root;rootrank;Bacteria;domain;"Planctomycetes";phylum;"Planctomycetacia";class;Planctomycetales;order;Planctomycetaceae;family;Gemmata;genus	85.79	4.00E-108	392
2882	0	0	0	0	1	0	0	0	0	Planctomycetes	Planctomycea	Gemmatales	Gemmataceae	S000389818 Gemmata-like str. CJuql4 Lineage=Root;rootrank;Bacteria;domain;"Planctomycetes";phylum;"Planctomycetacia";class;Planctomycetales;order;Planctomycetaceae;family;Gemmata;genus	88.38	1.00E-123	444
383	0	0	0	1	0	0	0	0	0	Planctomycetes	Planctomycea	Gemmatales	Gemmataceae	S000389818 Gemmata-like str. CJuql4 Lineage=Root;rootrank;Bacteria;domain;"Planctomycetes";phylum;"Planctomycetacia";class;Planctomycetales;order;Planctomycetaceae;family;Gemmata;genus	88.44	8.00E-125	448
2900	0	0	0	1	0	0	0	0	0	Planctomycetes	Planctomycea	Gemmatales	Gemmataceae	S000389818 Gemmata-like str. CJuql4 Lineage=Root;rootrank;Bacteria;domain;"Planctomycetes";phylum;"Planctomycetacia";class;Planctomycetales;order;Planctomycetaceae;family;Gemmata;genus	91.13	2.00E-141	503
2905	0	0	0	1	0	0	0	0	0	Planctomycetes	Planctomycea	Gemmatales	Gemmataceae	S000389818 Gemmata-like str. CJuql4 Lineage=Root;rootrank;Bacteria;domain;"Planctomycetes";phylum;"Planctomycetacia";class;Planctomycetales;order;Planctomycetaceae;family;Gemmata;genus	88.68	6.00E-126	451
47	0	0	5	4	0	1	1	6	3	Planctomycetes	Planctomycea	Gemmatales	Gemmataceae	S000389819 Gemmata-like str. JW3-8s0 Lineage=Root;rootrank;Bacteria;domain;"Planctomycetes";phylum;"Planctomycetacia";class;Planctomycetales;order;Planctomycetaceae;family;Gemmata;genus	87.06	7.00E-116	418
1660	0	0	0	3	1	0	0	0	0	Planctomycetes	Planctomycea	Gemmatales	Gemmataceae	S000389819 Gemmata-like str. JW3-8s0 Lineage=Root;rootrank;Bacteria;domain;"Planctomycetes";phylum;"Planctomycetacia";class;Planctomycetales;order;Planctomycetaceae;family;Gemmata;genus	88.17	1.00E-122	440
1826	0	0	2	0	0	0	0	0	0	Planctomycetes	Planctomycea	Gemmatales	Gemmataceae	S000389819 Gemmata-like str. JW3-8s0 Lineage=Root;rootrank;Bacteria;domain;"Planctomycetes";phylum;"Planctomycetacia";class;Planctomycetales;order;Planctomycetaceae;family;Gemmata;genus	87.37	1.00E-117	424
1059	0	0	0	0	1	0	0	0	1	Planctomycetes	Planctomycea	Gemmatales	Gemmataceae	S000389819 Gemmata-like str. JW3-8s0 Lineage=Root;rootrank;Bacteria;domain;"Planctomycetes";phylum;"Planctomycetacia";class;Planctomycetales;order;Planctomycetaceae;family;Gemmata;genus	84.95	4.00E-103	375
2178	0	0	0	0	0	0	0	1	0	Planctomycetes	Planctomycea	Gemmatales	Gemmataceae	S000389819 Gemmata-like str. JW3-8s0 Lineage=Root;rootrank;Bacteria;domain;"Planctomycetes";phylum;"Planctomycetacia";class;Planctomycetales;order;Planctomycetaceae;family;Gemmata;genus	88.47	8.00E-125	448
1048	0	0	0	0	0	2	1	0	0	Planctomycetes	Planctomycea	Gemmatales	Gemmataceae	S000389820 Gemmata-like str. JW10-3f1 Lineage=Root;rootrank;Bacteria;domain;"Planctomycetes";phylum;"Planctomycetacia";class;Planctomycetales;order;Planctomycetaceae;family;Zavarzinella;genus	87.17	2.00E-116	420
2751	0	0	0	1	0	0	0	0	0	Planctomycetes	Planctomycea	Gemmatales	Gemmataceae	S000389820 Gemmata-like str. JW10-3f1 Lineage=Root;rootrank;Bacteria;domain;"Planctomycetes";phylum;"Planctomycetacia";class;Planctomycetales;order;Planctomycetaceae;family;Zavarzinella;genus	90.37	2.00E-136	486
1968	0	0	0	0	0	0	0	1	0	Planctomycetes	Planctomycea	Gemmatales	Gemmataceae	S000389820 Gemmata-like str. JW10-3f1 Lineage=Root;rootrank;Bacteria;domain;"Planctomycetes";phylum;"Planctomycetacia";class;Planctomycetales;order;Planctomycetaceae;family;Zavarzinella;genus	87.17	2.00E-116	420
3028	0	0	1	0	1	0	0	0	0	Planctomycetes	Planctomycea	Gemmatales	Gemmataceae	S000389821 Gemmata-like str. JW11-2f5 Lineage=Root;rootrank;Bacteria;domain;"Planctomycetes";phylum;"Planctomycetacia";class;Planctomycetales;order;Planctomycetaceae;family;Gemmata;genus	86.9	1.00E-113	411
2438	0	0	0	0	1	0	0	0	0	Planctomycetes	Planctomycea	Gemmatales	Gemmataceae	S000389821 Gemmata-like str. JW11-2f5 Lineage=Root;rootrank;Bacteria;domain;"Planctomycetes";phylum;"Planctomycetacia";class;Planctomycetales;order;Planctomycetaceae;family;Gemmata;genus	83.42	1.00E-92	340
755	0	0	0	0	2	10	12	1	0	Planctomycetes	Planctomycea	Gemmatales	Gemmataceae	S000650674 Zavarzinella formosa (T); type strain: A10 Lineage=Root;rootrank;Bacteria;domain;"Planctomycetes";phylum;"Planctomycetacia";class;Planctomycetales;order;Planctomycetaceae;family;Zavarzinella;genus	89.25	8.00E-130	464
2400	0	0	3	5	5	1	1	1	2	Planctomycetes	Planctomycea	Gemmatales	Gemmataceae	S000650674 Zavarzinella formosa (T); type strain: A10 Lineage=Root;rootrank;Bacteria;domain;"Planctomycetes";phylum;"Planctomycetacia";class;Planctomycetales;order;Planctomycetaceae;family;Zavarzinella;genus	88.44	8.00E-125	448
1819	0	0	0	10	1	0	0	1	1	Planctomycetes	Planctomycea	Gemmatales	Gemmataceae	S000650674 Zavarzinella formosa (T); type strain: A10 Lineage=Root;rootrank;Bacteria;domain;"Planctomycetes";phylum;"Planctomycetacia";class;Planctomycetales;order;Planctomycetaceae;family;Zavarzinella;genus	87.37	1.00E-117	424
3012	0	0	0	0	0	0	4	5	5	Planctomycetes	Planctomycea	Gemmatales	Gemmataceae	S000650674 Zavarzinella formosa (T); type strain: A10 Lineage=Root;rootrank;Bacteria;domain;"Planctomycetes";phylum;"Planctomycetacia";class;Planctomycetales;order;Planctomycetaceae;family;Zavarzinella;genus	87.67	8.00E-120	431
3250	0	0	0	0	1	3	2	0	1	Planctomycetes	Planctomycea	Gemmatales	Gemmataceae	S000650674 Zavarzinella formosa (T); type strain: A10 Lineage=Root;rootrank;Bacteria;domain;"Planctomycetes";phylum;"Planctomycetacia";class;Planctomycetales;order;Planctomycetaceae;family;Zavarzinella;genus	88.95	1.00E-127	457
1092	0	0	0	3	1	0	0	1	0	Planctomycetes	Planctomycea	Gemmatales	Gemmataceae	S000650674 Zavarzinella formosa (T); type strain: A10 Lineage=Root;rootrank;Bacteria;domain;"Planctomycetes";phylum;"Planctomycetacia";class;Planctomycetales;order;Planctomycetaceae;family;Zavarzinella;genus	87.33	4.00E-118	425
871	0	0	1	3	0	0	0	0	1	Planctomycetes	Planctomycea	Gemmatales	Gemmataceae	S000650674 Zavarzinella formosa (T); type strain: A10 Lineage=Root;rootrank;Bacteria;domain;"Planctomycetes";phylum;"Planctomycetacia";class;Planctomycetales;order;Planctomycetaceae;family;Zavarzinella;genus	87.8	8.00E-120	431
348	0	0	0	1	1	0	0	0	2	Planctomycetes	Planctomycea	Gemmatales	Gemmataceae	S000650674 Zavarzinella formosa (T); type strain: A10 Lineage=Root;rootrank;Bacteria;domain;"Planctomycetes";phylum;"Planctomycetacia";class;Planctomycetales;order;Planctomycetaceae;family;Zavarzinella;genus	87.43	4.00E-118	425
1224	0	0	0	0	3	0	0	0	0	Planctomycetes	Planctomycea	Gemmatales	Gemmataceae	S000650674 Zavarzinella formosa (T); type strain: A10 Lineage=Root;rootrank;Bacteria;domain;"Planctomycetes";phylum;"Planctomycetacia";class;Planctomycetales;order;Planctomycetaceae;family;Zavarzinella;genus	87.67	2.00E-120	433
2277	0	0	0	1	0	0	0	1	2	Planctomycetes	Planctomycea	Gemmatales	Gemmataceae	S000650674 Zavarzinella formosa (T); type strain: A10 Lineage=Root;rootrank;Bacteria;domain;"Planctomycetes";phylum;"Planctomycetacia";class;Planctomycetales;order;Planctomycetaceae;family;Zavarzinella;genus	87.6	3.00E-119	429

745	0	0	0	1	2	0	0	0	0	Planctomycetes	Planctomycea	Gemmatales	Gemmataceae		S000650674 Zavarzinella formosa (T); type strain: A10 Lineage=Root;rootrank;Bacteria;domain;"Planctomycetes";phylum;"Planctomycetacia";class;Planctomycetales;order;Planctomycetaceae;family;Zavarzinella;genus	85.9	4.00E-108	392
2398	0	0	0	1	0	1	1	0	0	Planctomycetes	Planctomycea	Gemmatales	Gemmataceae		S000650674 Zavarzinella formosa (T); type strain: A10 Lineage=Root;rootrank;Bacteria;domain;"Planctomycetes";phylum;"Planctomycetacia";class;Planctomycetales;order;Planctomycetaceae;family;Zavarzinella;genus	88.5	8.00E-125	448
305	0	0	0	2	0	0	0	1	0	Planctomycetes	Planctomycea	Gemmatales	Gemmataceae		S000650674 Zavarzinella formosa (T); type strain: A10 Lineage=Root;rootrank;Bacteria;domain;"Planctomycetes";phylum;"Planctomycetacia";class;Planctomycetales;order;Planctomycetaceae;family;Zavarzinella;genus	88.44	3.00E-124	446
2543	0	0	0	0	0	0	0	0	2	Planctomycetes	Planctomycea	Gemmatales	Gemmataceae		S000650674 Zavarzinella formosa (T); type strain: A10 Lineage=Root;rootrank;Bacteria;domain;"Planctomycetes";phylum;"Planctomycetacia";class;Planctomycetales;order;Planctomycetaceae;family;Zavarzinella;genus	87.13	2.00E-116	420
123	0	0	0	0	1	0	0	0	0	Planctomycetes	Planctomycea	Gemmatales	Gemmataceae		S000650674 Zavarzinella formosa (T); type strain: A10 Lineage=Root;rootrank;Bacteria;domain;"Planctomycetes";phylum;"Planctomycetacia";class;Planctomycetales;order;Planctomycetaceae;family;Zavarzinella;genus	85.56	2.00E-106	387
287	0	0	0	0	1	0	0	0	0	Planctomycetes	Planctomycea	Gemmatales	Gemmataceae		S000650674 Zavarzinella formosa (T); type strain: A10 Lineage=Root;rootrank;Bacteria;domain;"Planctomycetes";phylum;"Planctomycetacia";class;Planctomycetales;order;Planctomycetaceae;family;Zavarzinella;genus	89.36	3.00E-129	462
2714	0	0	0	1	0	0	0	0	0	Planctomycetes	Planctomycea	Gemmatales	Gemmataceae		S000650674 Zavarzinella formosa (T); type strain: A10 Lineage=Root;rootrank;Bacteria;domain;"Planctomycetes";phylum;"Planctomycetacia";class;Planctomycetales;order;Planctomycetaceae;family;Zavarzinella;genus	89.49	2.00E-131	470
3159	0	0	0	0	0	0	1	0	0	Planctomycetes	Planctomycea	Gemmatales	Gemmataceae		S000650674 Zavarzinella formosa (T); type strain: A10 Lineage=Root;rootrank;Bacteria;domain;"Planctomycetes";phylum;"Planctomycetacia";class;Planctomycetales;order;Planctomycetaceae;family;Zavarzinella;genus	87.7	8.00E-120	431
1208	0	0	0	0	0	0	0	1	0	Planctomycetes	Planctomycea	Gemmatales	Gemmataceae		S000650674 Zavarzinella formosa (T); type strain: A10 Lineage=Root;rootrank;Bacteria;domain;"Planctomycetes";phylum;"Planctomycetacia";class;Planctomycetales;order;Planctomycetaceae;family;Zavarzinella;genus	89.01	4.00E-128	459
1255	0	0	0	0	0	0	0	1	0	Planctomycetes	Planctomycea	Gemmatales	Gemmataceae		S000650674 Zavarzinella formosa (T); type strain: A10 Lineage=Root;rootrank;Bacteria;domain;"Planctomycetes";phylum;"Planctomycetacia";class;Planctomycetales;order;Planctomycetaceae;family;Zavarzinella;genus	86.74	4.00E-113	409
3063	0	0	0	2	6	0	0	0	0	Planctomycetes	Planctomycea	Gemmatales	Gemmataceae	Gemmata	S000389818 Gemmata-like str. CJuql4 Lineage=Root;rootrank;Bacteria;domain;"Planctomycetes";phylum;"Planctomycetacia";class;Planctomycetales;order;Planctomycetaceae;family;Gemmata;genus	89.49	2.00E-131	470
2027	0	0	0	1	0	0	0	1	0	Planctomycetes	Planctomycea	Gemmatales	Gemmataceae	Gemmata	S000389818 Gemmata-like str. CJuql4 Lineage=Root;rootrank;Bacteria;domain;"Planctomycetes";phylum;"Planctomycetacia";class;Planctomycetales;order;Planctomycetaceae;family;Gemmata;genus	95.71	8.00E-170	597
1448	0	0	1	0	0	0	0	0	0	Planctomycetes	Planctomycea	Gemmatales	Gemmataceae	Gemmata	S000389818 Gemmata-like str. CJuql4 Lineage=Root;rootrank;Bacteria;domain;"Planctomycetes";phylum;"Planctomycetacia";class;Planctomycetales;order;Planctomycetaceae;family;Gemmata;genus	90.86	8.00E-140	497
577	0	0	0	1	0	0	0	0	0	Planctomycetes	Planctomycea	Gemmatales	Gemmataceae	Gemmata	S000389818 Gemmata-like str. CJuql4 Lineage=Root;rootrank;Bacteria;domain;"Planctomycetes";phylum;"Planctomycetacia";class;Planctomycetales;order;Planctomycetaceae;family;Gemmata;genus	95.42	1.00E-167	590
2192	0	0	0	8	0	0	0	0	0	Planctomycetes	Planctomycea	Gemmatales	Gemmataceae	Gemmata	S000389819 Gemmata-like str. JW3-8s0 Lineage=Root;rootrank;Bacteria;domain;"Planctomycetes";phylum;"Planctomycetacia";class;Planctomycetales;order;Planctomycetaceae;family;Gemmata;genus	92.45	8.00E-150	531
2431	0	0	2	0	1	0	1	0	1	Planctomycetes	Planctomycea	Gemmatales	Gemmataceae	Gemmata	S000389819 Gemmata-like str. JW3-8s0 Lineage=Root;rootrank;Bacteria;domain;"Planctomycetes";phylum;"Planctomycetacia";class;Planctomycetales;order;Planctomycetaceae;family;Gemmata;genus	87.6	3.00E-119	429
1951	0	0	0	1	2	0	0	0	0	Planctomycetes	Planctomycea	Gemmatales	Gemmataceae	Gemmata	S000650674 Zavarzinella formosa (T); type strain: A10 Lineage=Root;rootrank;Bacteria;domain;"Planctomycetes";phylum;"Planctomycetacia";class;Planctomycetales;order;Planctomycetaceae;family;Zavarzinella;genus	91.96	2.00E-146	520
1424	0	0	0	0	0	0	1	1	0	Planctomycetes	Planctomycea	Gemmatales	Gemmataceae	Gemmata	S000650674 Zavarzinella formosa (T); type strain: A10 Lineage=Root;rootrank;Bacteria;domain;"Planctomycetes";phylum;"Planctomycetacia";class;Planctomycetales;order;Planctomycetaceae;family;Zavarzinella;genus	92.45	8.00E-150	531
1763	0	0	0	0	0	0	1	1	0	Planctomycetes	Planctomycea	Gemmatales	Gemmataceae	Gemmata	S000650674 Zavarzinella formosa (T); type strain: A10 Lineage=Root;rootrank;Bacteria;domain;"Planctomycetes";phylum;"Planctomycetacia";class;Planctomycetales;order;Planctomycetaceae;family;Zavarzinella;genus	94.62	4.00E-163	575
213	0	0	0	9	3	1	4	17	2	Planctomycetes	Planctomycea	Gemmatales	Gemmataceae		S000389819 Gemmata-like str. JW3-8s0 Lineage=Root;rootrank;Bacteria;domain;"Planctomycetes";phylum;"Planctomycetacia";class;Planctomycetales;order;Planctomycetaceae;family;Gemmata;genus	87.6	8.00E-120	431
536	0	0	0	3	0	0	0	0	0	Planctomycetes	Planctomycea	Gemmatales	Gemmataceae		S000650674 Zavarzinella formosa (T); type strain: A10 Lineage=Root;rootrank;Bacteria;domain;"Planctomycetes";phylum;"Planctomycetacia";class;Planctomycetales;order;Planctomycetaceae;family;Zavarzinella;genus	84.31	4.00E-98	359
3229	0	1	0	2	0	1	3	2	1	Planctomycetes	Planctomycea	Gemmatales	Isosphaeraceae		S000018536 planctomycete str. 532 Lineage=Root;rootrank;Bacteria;domain;"Planctomycetes";phylum;"Planctomycetacia";class;Planctomycetales;order;Planctomycetaceae;family;unclassified_Planctomycetaceae;	91.2	1.00E-142	507
2383	0	0	2	0	1	0	1	2	2	Planctomycetes	Planctomycea	Gemmatales	Isosphaeraceae		S000018536 planctomycete str. 532 Lineage=Root;rootrank;Bacteria;domain;"Planctomycetes";phylum;"Planctomycetacia";class;Planctomycetales;order;Planctomycetaceae;family;unclassified_Planctomycetaceae;	93.07	3.00E-154	545
1865	0	0	1	0	0	0	0	0	0	Planctomycetes	Planctomycea	Gemmatales	Isosphaeraceae		S000018536 planctomycete str. 532 Lineage=Root;rootrank;Bacteria;domain;"Planctomycetes";phylum;"Planctomycetacia";class;Planctomycetales;order;Planctomycetaceae;family;unclassified_Planctomycetaceae;	90.67	1.00E-138	494
2652	0	0	0	0	1	0	0	0	0	Planctomycetes	Planctomycea	Gemmatales	Isosphaeraceae		S000018536 planctomycete str. 532 Lineage=Root;rootrank;Bacteria;domain;"Planctomycetes";phylum;"Planctomycetacia";class;Planctomycetales;order;Planctomycetaceae;family;unclassified_Planctomycetaceae;	91.47	3.00E-144	512
2649	0	0	0	0	1	0	0	0	0	Planctomycetes	Planctomycea	Gemmatales			S000018536 planctomycete str. 532 Lineage=Root;rootrank;Bacteria;domain;"Planctomycetes";phylum;"Planctomycetacia";class;Planctomycetales;order;Planctomycetaceae;family;unclassified_Planctomycetaceae;	84.72	9.00E-100	364
1332	0	0	1	0	0	0	0	2	1	Planctomycetes	Planctomycea	Pirellulales			S002952183 planctomycete DDSe3011 Lineage=Root;rootrank;Bacteria;domain;"Planctomycetes";phylum;"Planctomycetacia";class;Planctomycetales;order;Planctomycetaceae;family;Blastopirellula;genus	95.7	2.00E-170	599
2313	0	0	0	0	1	0	0	0	0	Planctomycetes	Planctomycea	Pirellulales			S002952183 planctomycete DDSe3011 Lineage=Root;rootrank;Bacteria;domain;"Planctomycetes";phylum;"Planctomycetacia";class;Planctomycetales;order;Planctomycetaceae;family;Blastopirellula;genus	91.94	5.00E-147	521
110	0	0	0	0	0	0	0	1	0	Planctomycetes	Planctomycea	Pirellulales			S002952183 planctomycete DDSe3011 Lineage=Root;rootrank;Bacteria;domain;"Planctomycetes";phylum;"Planctomycetacia";class;Planctomycetales;order;Planctomycetaceae;family;Blastopirellula;genus	91.67	2.00E-145	516
1684	9	55	220	3	55	73	142	199	96	Planctomycetes	Planctomycea	Pirellulales			S002952186 planctomycete MS1399 Lineage=Root;rootrank;Bacteria;domain;"Planctomycetes";phylum;"Planctomycetacia";class;Planctomycetales;order;Planctomycetaceae;family;unclassified_Planctomycetaceae;	94.35	2.00E-161	569
3139	0	0	0	0	0	0	1	0	0	Planctomycetes	Planctomycea	Pirellulales			S002952186 planctomycete MS1399 Lineage=Root;rootrank;Bacteria;domain;"Planctomycetes";phylum;"Planctomycetacia";class;Planctomycetales;order;Planctomycetaceae;family;unclassified_Planctomycetaceae;	92.86	7.00E-161	568
989	0	0	0	1	2	0	0	0	0	Planctomycetes	Planctomycea	Pirellulales			S002952187 planctomycete DSC3C2 Lineage=Root;rootrank;Bacteria;domain;"Planctomycetes";phylum;"Planctomycetacia";class;Planctomycetales;order;Planctomycetaceae;family;unclassified_Planctomycetaceae;	90.22	1.00E-132	473
2291	3	3	57	41	63	29	52	36	33	Planctomycetes	Planctomycea	Pirellulales			S002952188 planctomycete DDSW3022 Lineage=Root;rootrank;Bacteria;domain;"Planctomycetes";phylum;"Planctomycetacia";class;Planctomycetales;order;Planctomycetaceae;family;unclassified_Planctomycetaceae;	93.85	1.00E-158	560
2221	3	0	10	10	14	5	5	4	3	Planctomycetes	Planctomycea	Pirellulales			S002952188 planctomycete DDSW3022 Lineage=Root;rootrank;Bacteria;domain;"Planctomycetes";phylum;"Planctomycetacia";class;Planctomycetales;order;Planctomycetaceae;family;unclassified_Planctomycetaceae;	90.68	2.00E-135	483
1007	0	0	0	3	0	0	0	0	1	Planctomycetes	Planctomycea	Pirellulales			S002952188 planctomycete DDSW3022 Lineage=Root;rootrank;Bacteria;domain;"Planctomycetes";phylum;"Planctomycetacia";class;Planctomycetales;order;Planctomycetaceae;family;unclassified_Planctomycetaceae;	95.16	5.00E-167	588
2078	0	0	0	0	2	0	0	0	0	Planctomycetes	Planctomycea	Pirellulales			S002952188 planctomycete DDSW3022 Lineage=Root;rootrank;Bacteria;domain;"Planctomycetes";phylum;"Planctomycetacia";class;Planctomycetales;order;Planctomycetaceae;family;unclassified_Planctomycetaceae;	94.39	7.00E-171	601
235	0	0	0	2	0	0	0	0	0	Planctomycetes	Planctomycea	Pirellulales			S002952188 planctomycete DDSW3022 Lineage=Root;rootrank;Bacteria;domain;"Planctomycetes";phylum;"Planctomycetacia";class;Planctomycetales;order;Planctomycetaceae;family;unclassified_Planctomycetaceae;	92.21	2.00E-156	553
2786	0	0	0	0	1	0	0	1	0	Planctomycetes	Planctomycea	Pirellulales			S002952188 planctomycete DDSW3022 Lineage=Root;rootrank;Bacteria;domain;"Planctomycetes";phylum;"Planctomycetacia";class;Planctomycetales;order;Planctomycetaceae;family;unclassified_Planctomycetaceae;	92.86	2.00E-160	566
1020	0	0	0	0	1	0	0	0	0	Planctomycetes	Planctomycea	Pirellulales			S002952188 planctomycete DDSW3022 Lineage=Root;rootrank;Bacteria;domain;"Planctomycetes";phylum;"Planctomycetacia";class;Planctomycetales;order;Planctomycetaceae;family;unclassified_Planctomycetaceae;	93.83	1.00E-158	560
1199	0	0	0	0	1	0	0	0	0	Planctomycetes	Planctomycea	Pirellulales			S002952188 planctomycete DDSW3022 Lineage=Root;rootrank;Bacteria;domain;"Planctomycetes";phylum;"Planctomycetacia";class;Planctomycetales;order;Planctomycetaceae;family;unclassified_Planctomycetaceae;	92.39	1.00E-157	556
654	0	0	0	1	0	0	0	0	0	Planctomycetes	Planctomycea	Pirellulales			S002952188 planctomycete DDSW3022 Lineage=Root;rootrank;Bacteria;domain;"Planctomycetes";phylum;"Planctomycetacia";class;Planctomycetales;order;Planctomycetaceae;family;unclassified_Planctomycetaceae;	91.58	2.00E-140	499
440	1	1	7	0	2	0	1	0	0	Planctomycetes	Planctomycea	Pirellulales			S002952189 planctomycete DDSe3016 Lineage=Root;rootrank;Bacteria;domain;"Planctomycetes";phylum;"Planctomycetacia";class;Planctomycetales;order;Planctomycetaceae;family;unclassified_Planctomycetaceae;	93.01	1.00E-153	544
30	0	0	0	1	1	1	0	0	0	Planctomycetes	Planctomycea	Pirellulales			S002952189 planctomycete DDSe3016 Lineage=Root;rootrank;Bacteria;domain;"Planctomycetes";phylum;"Planctomycetacia";class;Planctomycetales;order;Planctomycetaceae;family;unclassified_Planctomycetaceae;	90.59	1.00E-138	494
1841	0	0	3	11	8	8	6	21	7	Planctomycetes	Planctomycea	Pirellulales			S002952190 planctomycete DDSe1305 Lineage=Root;rootrank;Bacteria;domain;"Planctomycetes";phylum;"Planctomycetacia";class;Planctomycetales;order;Planctomycetaceae;family;Blastopirellula;genus	93.35	1.00E-163	577
2859	0	0	3	3	4	0	0	14	2	Planctomycetes	Planctomycea	Pirellulales			S002952190 planctomycete DDSe1305 Lineage=Root;rootrank;Bacteria;domain;"Planctomycetes";phylum;"Planctomycetacia";class;Planctomycetales;order;Planctomycetaceae;family;Blastopirellula;genus	92.47	2.00E-150	532
862	1	0	0	0	1	1	0	0	1	Planctomycetes	Planctomycea	Pirellulales			S002952190 planctomycete DDSe1305 Lineage=Root;rootrank;Bacteria;domain;"Planctomycetes";phylum;"Planctomycetacia";class;Planctomycetales;order;Planctomycetaceae;family;Blastopirellula;genus	94.64	1.00E-163	577
2022	0	0	2	0	1	1	0	1	0	Planctomycetes	Planctomycea	Pirellulales			S002952190 planctomycete DDSe1305 Lineage=Root;rootrank;Bacteria;domain;"Planctomycetes";phylum;"Planctomycetacia";class;Planctomycetales;order;Planctomycetaceae;family;Blastopirellula;genus	94.62	1.00E-163	577
1596	0	0	0	0	1	0	0	0	0	Planctomycetes	Planctomycea	Pirellulales			S002952190 planctomycete DDSe1305 Lineage=Root;rootrank;Bacteria;domain;"Planctomycetes";phylum;"Planctomycetacia";class;Planctomycetales;order;Planctomycetaceae;family;Blastopirellula;genus	92.23	1.00E-148	527
1390	0	1	3	10	11	30	35	29	18	Planctomycetes	Planctomycea	Pirellulales			S002952191 planctomycete DDSe3021 Lineage=Root;rootrank;Bacteria;domain;"Planctomycetes";phylum;"Planctomycetacia";class;Planctomycetales;order;Planctomycetaceae;family;unclassified_Planctomycetaceae;	93.09	5.00E-162	571
917	0	0	22	11	15	9	14	7	3	Planctomycetes	Planctomycea	Pirellulales			S002952191 planctomycete DDSe3021 Lineage=Root;rootrank;Bacteria;domain;"Planctomycetes";phylum;"Planctomycetacia";class;Planctomycetales;order;Planctomycetaceae;family;unclassified_Planctomycetaceae;	94.9	3.00E-174	612
3168	0	0	5	13	4	0	1	2	4	Planctomycetes	Planctomycea	Pirellulales			S002952191 planctomycete DDSe3021 Lineage=Root;rootrank;Bacteria;domain;"Planctomycetes";phylum;"Planctomycetacia";class;Planctomycetales;order;Planctomycetaceae;family;unclassified_Planctomycetaceae;	94.59	1.00E-162	573
892	3	0	1	1	4	2	1	6	9	Planctomycetes	Planctomycea	Pirellulales			S002952191 planctomycete DDSe3021 Lineage=Root;rootrank;Bacteria;domain;"Planctomycetes";phylum;"Planctomycetacia";class;Planctomycetales;order;Planctomycetaceae;family;unclassified_Planctomycetaceae;	97.85	0	641
541	0	0	0	0	2	0	0	0	0	Planctomycetes	Planctomycea	Pirellulales			S002952191 planctomycete DDSe3021 Lineage=Root;rootrank;Bacteria;domain;"Planctomycetes";phylum;"Planctomycetacia";class;Planctomycetales;order;Planctomycetaceae;family;unclassified_Planctomycetaceae;	87.47	1.00E-118	427
2501	0	0	0	2	0	0	0	0	0	Planctomycetes	Planctomycea	Pirellulales			S002952191 planctomycete DDSe3021 Lineage=Root;rootrank;Bacteria;domain;"Planctomycetes";phylum;"Planctomycetacia";class;Planctomycetales;order;Planctomycetaceae;family;unclassified_Planctomycetaceae;	91	3.00E-149	529
1644	0	1	0	0	0	0	0	0	0	Planctomycetes	Planctomycea	Pirellulales			S002952191 planctomycete DDSe3021 Lineage=Root;rootrank;Bacteria;domain;"Planctomycetes";phylum;"Planctomycetacia";class;Planctomycetales;order;Planctomycetaceae;family;unclassified_Planctomycetaceae;	93.4	9.00E-165	580
1011	0	0	0	0	0	1	0	0	0	Planctomycetes	Planctomycea	Pirellulales			S002952191 planctomycete DDSe3021 Lineage=Root;rootrank;Bacteria;domain;"Planctomycetes";phylum;"Planctomycetacia";class;Planctomycetales;order;Planctomycetaceae;family;unclassified_Planctomycetaceae;	95.14	7.00E-176	617
3155	0	0	0	0	0	0	0	1	0	Planctomycetes	Planctomycea	Pirellulales			S002952191 planctomycete DDSe3021 Lineage=Root;rootrank;Bacteria;domain;"Planctomycetes";phylum;"Planctomycetacia";class;Planctomycetales;order;Planctomycetaceae;family;unclassified_Planctomycetaceae;	93.55	2.00E-156	553
2043	0	0	0	0	0	0	0	0	1	Planctomycetes	Planctomycea	Pirellulales			S002952191 planctomycete DDSe3021 Lineage=Root;rootrank;Bacteria;domain;"Planctomycetes";phylum;"Planctomycetacia";class;Planctomycetales;order;Planctomycetaceae;family;unclassified_Planctomycetaceae;	93.85	3.00E-159	562
695	1	0	2	2	1	5	13	2	2	Planctomycetes	Planctomycea	Pirellulales			S002952192 planctomycete MS1331 Lineage=Root;rootrank;Bacteria;domain;"Planctomycetes";phylum;"Planctomycetacia";class;Planctomycetales;order;Planctomycetaceae;family;unclassified_Planctomycetaceae;	93.61	7.00E-166	584
681	1	35	3	0	0	0	4	0	1	Planctomycetes	Planctomycea	Pirellulales			S002952195 planctomycete DDSe3017 Lineage=Root;rootrank;Bacteria;domain;unclassified_Bacteria;	92.58	1.00E-147	523
2987	1	0	2	2	0	0	1	0	0	Planctomycetes	Planctomycea	Pirellulales			S002952195 planctomycete DDSe3017 Lineage=Root;rootrank;Bacteria;domain;unclassified_Bacteria;	94.2	2.00E-156	553
3221	3	1	1	5	1	0	2	8	3	Planctomycetes	Planctomycea	Pirellulales			S003288169 Blastopirellula marina; type strain: DSM 3645 Lineage=Root;rootrank;Bacteria;domain;"Planctomycetes";phylum;"Planctomycetacia";class;Planctomycetales;order;Planctomycetaceae;family;Blastopirellula;genus	91.42	4.00E-143	508
700	0	0	1	1	2	0	2	0	2	Planctomycetes	Planctomycea	Pirellulales			S003288169 Blastopirellula marina; type strain: DSM 3645 Lineage=Root;rootrank;Bacteria;domain;"Planctomycetes";phylum;"Planctomycetacia";class;Planctomycetales;order;Planctomycetaceae;family;Blastopirellula;genus	92.58	1.00E-147	523
1960	0	0	0	1	1	1	0	0	0	Planctomycetes	Planctomycea	Pirellulales			S003288169 Blastopirellula marina; type strain: DSM 3645 Lineage=Root;rootrank;Bacteria;domain;"Planctomycetes";phylum;"Planctomycetacia";class;Planctomycetales;order;Planctomycetaceae;family;Blastopirellula;genus	92.74	5.00E-152	538
1311	0	1	0	0	0	0	0	0	1	Planctomycetes	Planctomycea	Pirellulales			S003288169 Blastopirellula marina; type strain: DSM 3645 Lineage=Root;rootrank;Bacteria;domain;"Planctomycetes";phylum;"Planctomycetacia";class;Planctomycetales;order;Planctomycetaceae;family;Blastopirellula;genus	88.8	5.00E-127	455
1742	0	0	0	1	0	0	0	0	0	Planctomycetes	Planctomycea	Pirellulales			S003288169 Blastopirellula marina; type strain: DSM 3645 Lineage=Root;rootrank;Bacteria;domain;"Planctomycetes";phylum;"Planctomycetacia";class;Planctomycetales;order;Planctomycetaceae;family;Blastopirellula;genus	89.22	3.00E-129	462
2670	0	0	0	0	0	1	0	0	0	Planctomycetes	Planctomycea	Pirellulales			S003288169 Blastopirellula marina; type strain: DSM 3645 Lineage=Root;rootrank;Bacteria;domain;"Planctomycetes";phylum;"Planctomycetacia";class;Planctomycetales;order;Planctomycetaceae;family;Blastopirellula;genus	91.74	2.00E-141	503
1893	0	0	0	0	1	0	0	0	0	Planctomycetes	Planctomycea	Pirellulales		Rhodopirellula	S002952193 planctomycete MS1316 Lineage=Root;rootrank;Bacteria;domain;"Planctomycetes";phylum;"Planctomycetacia";class;Planctomycetales;order;Planctomycetaceae;family;unclassified_Planctomycetaceae;	91.05	3.00E-149	529
2328	0	0	0	8	6	0	0	1	0	Planctomycetes	Planctomycea	Pirellulales		Rhodopirellula	S002952197 planctomycete DDSW3008 Lineage=Root;rootrank;Bacteria;domain;"Planctomycetes";phylum;"Planctomycetacia";class;Planctomycetales;order;Planctomycetaceae;family;Rhodopirellula;genus	94.35	2.00E-161	569
1772	0	0	0	0	0	0	0	0	1	Planctomycetes	Planctomycea	Pirellulales		Rhodopirellula	S002952201 planctomycete MSD101 Lineage=Root;rootrank;Bacteria;domain;"Planctomycetes";phylum;"Planctomycetacia";class;Planctomycetales;order;Planctomycetaceae;family;Rhodopirellula;genus	94.09	2.00E-160	566
2911	1	0	0	0	0	0	0	2	1	Planctomycetes	Planctomycea	Pirellulales			S002952177 planctomycete MSF146 Lineage=Root;rootrank;Bacteria;domain;"Planctomycetes";phylum;"Planctomycetacia";class;Planctomycetales;order;Planctomycetaceae;family;Blastopirellula;genus	92.47	8.00E-150	531
1641	0	0	1	2	3	1	1	1	2	Planctomycetes	Planctomycea	Pirellulales			S002952197 planctomycete DDSW3008 Lineage=Root;rootrank;Bacteria;domain;"Planctomycetes";phylum;"Planctomycetacia";class;Planctomycetales;order;Planctomycetaceae;family;Rhodopirellula;genus	90.13	8.00E-135	481
1423	0	0	0	11	6	4	2	6	11	Planctomycetes	Planctomycea	Pirellulales	Pirellulaceae		S002952201 planctomycete MSD101 Lineage=Root;rootrank;Bacteria;domain;"Planctomycetes";phylum;"Planctomycetacia";class;Planctomycetales;order;Planctomycetaceae;family;Rhodopirellula;genus	88.17	1.00E-123	444
4	1	0	0	0	0	0	0	0	1	Planctomycetes	Planctomycea	Pirellulales	Pirellulaceae	A7	S002952188 planctomycete DDSW3022 Lineage=Root;rootrank;Bacteria;domain;"Planctomycetes";phylum;"Planctomycetacia";class;Planctomycetales;order;Planctomycetaceae;family;unclassified_Planctomycetaceae;	89.5	4.00E-128	459
2081	0	0	1	0	0	0	0	0	0	Planctomycetes	Planctomycea	Pirellulales	Pirellulaceae	A7	S002952190 planctomycete DDSe1305 Lineage=Root;rootrank;Bacteria;domain;"Planctomycetes";phylum;"Planctomycetacia";class;Planctomycetales;order;Planctomycetaceae;family;Blastopirellula;genus	88.46	1.00E-122	440
588	10	3	9	8	5	1	1	1	2	Planctomycetes	Planctomycea	Pirellulales	Pirellulaceae	A7	S002952191 planctomycete DDSe3021 Lineage=Root;rootrank;Bacteria;domain;"Planctomycetes";phylum;"Planctomycetacia";class;Planctomycetales;order;Planctomycetaceae;family;unclassified_Planctomycetaceae;	90.35	5.00E-137	488
1227	0	0	3	2	12	23	9	0	3	Planctomycetes	Planctomycea	Pirellulales	Pirellulaceae	A7	S002952191 planctomycete DDSe3021 Lineage=Root;rootrank;Bacteria;domain;"Planctomycetes";phylum;"Planctomycetacia";class;Planctomycetales;order;Planctomycetaceae;family;unclassified_Planctomycetaceae;	88.49	5.00E-122	438
389	0	0	5	48	65	2	2	4	24	Planctomycetes	Planctomycea	Pirellulales	Pirellulaceae	Pirellula	S002289094 Pirellula staleyi DSM 6068 Lineage=Root;rootrank;Bacteria;domain;Firmicutes;phylum;Bacilli;class;Bacillales;order;Pasteuriaceae;family;Pasteuria;genus	91.89	6.00E-146	518
1973	0	0	1	9	3	15	14	0	0	Planctomycetes	Planctomycea	Pirellulales	Pirellulaceae	Pirellula	S002289094 Pirellula staleyi DSM 6068 Lineage=Root;rootrank;Bacteria;domain;Firmicutes;phylum;Bacilli;class;Bacillales;order;Pasteuriaceae;family;Pasteuria;genus	91.77	4.00E-153	542
442	0	0	1	3	11	3	3	0	1	Planctomycetes	Planctomycea	Pirellulales	Pirellulaceae	Pirellula	S002289094 Pirellula staleyi DSM 6068 Lineage=Root;rootrank;Bacteria;domain;Firmicutes;phylum;Bacilli;class;Bacillales;order;Pasteuriaceae;family;Pasteuria;genus	91.35	5.00E-142	505
386	0	0	0	0	4	0	0	0	0	Planctomycetes	Planctomycea	Pirellulales	Pirellulaceae	Pirellula	S002289094 Pirellula staleyi DSM 6068 Lineage=Root;rootrank;Bacteria;domain;Firmicutes;phylum;Bacilli;class;Bacillales;order;Pasteuriaceae;family;Pasteuria;genus	90.26	4.00E-143	508
1236	0	0	0	4	0	0	0	0	0	Planctomycetes	Planctomycea	Pirellulales	Pirellulaceae	Pirellula	S002289094 Pirellula staleyi DSM 6068 Lineage=Root;rootrank;Bacteria;domain;Firmicutes;phylum;Bacilli;class;Bacillales;order;Pasteuriaceae;family;Pasteuria;genus	91.94	6.00E-146	518
39	0	0	0	1	1	0	0	0	0	Planctomycetes	Planctomycea	Pirellulales	Pirellulaceae	Pirellula	S002289094 Pirellula staleyi DSM 6068 Lineage=Root;rootrank;Bacteria;domain;Firmicutes;phylum;Bacilli;class;Bacillales;order;Pasteuriaceae;family;Pasteuria;genus	92.23	1.00E-147	523
324	0	0	1	0	0	0	0	0	0	Planctomycetes	Planctomycea	Pirellulales	Pirellulaceae	Pirellula	S002289094 Pirellula staleyi DSM 6068 Lineage=Root;rootrank;Bacteria;domain;Firmicutes;phylum;Bacilli;class;Bacillales;order;Pasteuriaceae;family;Pasteuria;genus	89.82	2.00E-141	503
173	0	0	0	0	1	0	0	0	0	Planctomycetes	Planctomycea	Pirellulales	Pirellulaceae	Pirellula	S002289094 Pirellula staleyi DSM 6068 Lineage=Root;rootrank;Bacteria;domain;Firmicutes;phylum;Bacilli;class;Bacillales;order;Pasteuriaceae;family;Pasteuria;genus	95.89	8.00E-180	630
3030	0	0	1	0	0	0	0	0	0	Planctomycetes	Planctomycea	Pirellulales	Pirellulaceae		S002952188 planctomycete DDSW3022 Lineage=Root;rootrank;Bacteria;domain;"Planctomycetes";phylum;"Planctomycetacia";class;Planctomycetales;order;Planctomycetaceae;family;unclassified_Planctomycetaceae;	89.57	5.00E-132	472
320	0	0	0	0	0	0	6	0	3	Planctomycetes	Planctomycea	Pirellulales	Pirellulaceae		S002952190 planctomycete DDSe1305 Lineage=Root;rootrank;Bacteria;domain;"Planctomycetes";phylum;"Planctomycetacia";class;Planctomycetales;order;Planctomycetaceae;family;Blastopirellula;genus	88.24	9.00E-130	464
639	0	0	0	2	0	0	0	0	0	Planctomycetes	Planctomycea	Pirellulales	Pirellulaceae		S002952191 planctomycete DDSe3021 Lineage=Root;rootrank;Bacteria;domain;"Planctomycetes";phylum;"Planctomycetacia";class;Planctomycetales;order;Planctomycetaceae;family;unclassified_Planctomycetaceae;	88.5	2.00E-125	449
1944	0	0	0	4	0	9	6	1	3	Planctomycetes	Planctomycea	Pirellulales			S000013122 Planctomycetaceae; Schlesner 670 Lineage=Root;rootrank;Bacteria;domain;unclassified_Bacteria;	87.2	5.00E-117	422
56	0	0	0	0	0	1	0	0	0	Planctomycetes	Planctomycea	Pirellulales			S000013122 Planctomycetaceae; Schlesner 670 Lineage=Root;rootrank;Bacteria;domain;unclassified_Bacteria;	86.13	2.00E-110	399
2126	0	0	0	0	1	0	0	0	0	Planctomycetes	Planctomycea	Pirellulales			S000019081 planctomycete str. 116 Lineage=Root;rootrank;Bacteria;domain;"Planctomycetes";phylum;"Planctomycetacia";class;Planctomycetales;order;Planctomycetaceae;family;Blastopirellula;genus	91.14	3.00E-149	529
79	0	0	0	1	0	0	0	0	0	Planctomycetes	Planctomycea	Pirellulales			S000871748 planctomycete Zi62 Lineage=Root;rootrank;Bacteria;domain;"Planctomycetes";phylum;"Planctomycetacia";class;Planctomycetales;order;Planctomycetaceae;family;Blastopirellula;genus	91.33	2.00E-140	499
2364	0	0	0	0	4	1	0	0	2	Planctomycetes	Planctomycea	Pirellulales			S002289094 Pirellula staleyi DSM 6068 Lineage=Root;rootrank;Bacteria;domain;Firmicutes;phylum;Bacilli;class;Bacillales;order;Pasteuriaceae;family;Pasteuria;genus	90.1	1.00E-138	494
578	0	0	1	2	2	0	0	0	0	Planctomycetes	Planctomycea	Pirellulales			S002289094 Pirellula staleyi DSM 6068 Lineage=Root;rootrank;Bacteria;domain;Firmicutes;phylum;Bacilli;class;Bacillales;order;Pasteuriaceae;family;Pasteuria;genus	90.44	1.00E-133	477
137	0	0	0	0	0	0	0	0	1	Planctomycetes	Planctomycea	Pirellulales			S002289094 Pirellula staleyi DSM 6068 Lineage=Root;rootrank;Bacteria;domain;Firmicutes;phylum;Bacilli;class;Bacillales;order;Pasteuriaceae;family;Pasteuria;genus	90.13	1.00E-138	494
2325	1	0	0	0	0	0	0	0	0	Planctomycetes	Planctomycea	Pirellulales			S002952183 planctomycete DDSe3011 Lineage=Root;rootrank;Bacteria;domain;"Planctomycetes";phylum;"Planctomycetacia";class;Planctomycetales;order;Planctomycetaceae;family;Blastopirellula;genus	90.79	2.00E-136	486
671	2	0	16	10	7	4	10	0	7	Planctomycetes	Planctomycea	Pirellulales			S002952185 planctomycete DDSe3013 Lineage=Root;rootrank;Bacteria;domain;"Planctomycetes";phylum;"Planctomycetacia";class;Planctomycetales;order;Planctomycetaceae;family;unclassified_Planctomycetaceae;	90.05	3.00E-134	479
1645	0	0	0	4	5	1	1	1	1	Planctomycetes	Planctomycea	Pirellulales			S002952188 planctomycete DDSW3022 Lineage=Root;rootrank;Bacteria;domain;"Planctomycetes";phylum;"Planctomycetacia";class;Planctomycetales;order;Planctomycetaceae;family;unclassified_Planctomycetaceae;	92.25	1.00E-148	527
444	0	0	0	1	1	0	0	0	0	Planctomycetes	Planctomycea	Pirellulales			S002952188 planctomycete DDSW3022 Lineage=Root;rootrank;Bacteria;domain;"Planctomycetes";phylum;"Planctomycetacia";class;Planctomycetales;order;Planctomycetaceae;family;unclassified_Planctomycetaceae;	90.32	6.00E-136	484
196	0	0	0	0	1	0	0	0	0	Planctomycetes	Planctomycea	Pirellulales			S002952188 planctomycete DDSW3022 Lineage=Root;rootrank;Bacteria;domain;"Planctomycetes";phylum;"Planctomycetacia";class;Planctomycetales;order;Planctomycetaceae;family;unclassified_Planctomycetaceae;	90.08	2.00E-135	483
579	0	0	0	1	0	0	0	0	0	Planctomycetes	Planctomycea	Pirellulales			S002952189 planctomycete DDSe3016 Lineage=Root;rootrank;Bacteria;domain;"Planctomycetes";phylum;"Planctomycetacia";class;Planctomycetales;order;Planctomycetaceae;family;unclassified_Planctomycetaceae;	90.05	8.00E-135	481
1721	0	0	0	1	0	0	0	0	0	Planctomycetes	Planctomycea	Pirellulales			S002952189 planctomycete DDSe3016 Lineage=Root;rootrank;Bacteria;domain;"Planctomycetes";phylum;"Planctomycetacia";class;Planctomycetales;order;Planctomycetaceae;family;unclassified_Planctomycetaceae;	89.52	5.00E-132	472
1254	0	0	0	0	0	0	0	1	0	Planctomycetes	Planctomycea	Pirellulales			S002952189 planctomycete DDSe3016 Lineage=Root;rootrank;Bacteria;domain;"Planctomycetes";phylum;"Planctomycetacia";class;Planctomycetales;order;Planctomycetaceae;family;unclassified_Planctomycetaceae;	89.25	2.00E-130	466
2206	1	0	9	15	17	3	9	7	15	Planctomycetes	Planctomycea	Pirellulales			S002952201 planctomycete MSD101 Lineage=Root;rootrank;Bacteria;domain;"Planctomycetes";phylum;"Planctomycetacia";class;Planctomycetales;order;Planctomycetaceae;family;Rhodopirellula;genus	86.56	4.00E-113	409
1353	0	0	0	0	0	0	0	0	2	Planctomycetes	Planctomycea	Pirellulales			S002952201 planctomycete MSD101 Lineage=Root;rootrank;Bacteria;domain;"Planctomycetes";phylum;"Planctomycetacia";class;Planctomycetales;order;Planctomycetaceae;family;Rhodopirellula;genus	88.44	2.00E-125	449
3198	0	0	0	0	0	0	1	0	0	Planctomycetes	Planctomycea	Pirellulales			S002952201 planctomycete MSD101 Lineage=Root;rootrank;Bacteria;domain;"Planctomycetes";phylum;"Planctomycetacia";class;Planctomycetales;order;Planctomycetaceae;family;Rhodopirellula;genus	87.67	8.00E-120	431
237	2	0	18	7	9	5	4	0	1	Planctomycetes	Planctomycea	Planctomycetales	Planctomycetaceae	Planctomyces	S000023044 Planctomyces maris; ATCC 29209T Lineage=Root;rootrank;Bacteria;domain;"Planctomycetes";phylum;"Planctomycetacia";class;Planctomycetales;order;Planctomycetaceae;family;Planctomyces;genus	88.11	1.00E-122	440
999	0	0	0	5	1	0	0	0	0	Planctomycetes	Planctomycea	Planctomycetales	Planctomycetaceae	Planctomyces	S000804520 Schlesneria paludicola (T); type strain: MPL7 Lineage=Root;rootrank;Bacteria;domain;"Planctomycetes";phylum;"Planctomycetacia";class;Planctomycetales;order;Planctomycetaceae;family;Schlesneria;genus	90.81	3.00E-139	496
414	0	0	0	1	0	0	0	0	0	Planctomycetes	Planctomycea	Planctomycetales	Planctomycetaceae	Planctomyces	S000804520 Schlesneria paludicola (T); type strain: MPL7 Lineage=Root;rootrank;Bacteria;domain;"Planctomycetes";phylum;"Planctomycetacia";class;Planctomycetales;order;Planctomycetaceae;family;Schlesneria;genus	89.97	2.00E-141	503
1745	3	8	39	2	11	1	5	2	3	Planctomycetes	Planctomycea	Planctomycetales	Planctomycetaceae	Planctomyces	S002289008 Planctomyces limnophilus DSM 3776 Lineage=Root;rootrank;Bacteria;domain;"Planctomycetes";phylum;"Planctomycetacia";class;Planctomycetales;order;Planctomycetaceae;family;Planctomyces;genus	87.6	4.00E-118	425
2443	4	0	2	4	0	0	0	2	0	Planctomycetes	Planctomycea	Planctomycetales	Planctomycetaceae	Planctomyces	S002289008 Planctomyces limnophilus DSM 3776 Lineage=Root;rootrank;Bacteria;domain;"Planctomycetes";phylum;"Planctomycetacia";class;Planctomycetales;order;Planctomycetaceae;family;Planctomyces;genus	85.83	1.00E-107	390
2174	0	0	0	1	0	1	0	1	0	Planctomycetes	Planctomycea	Planctomycetales	Planctomycetaceae	Planctomyces	S002289008 Planctomyces limnophilus DSM 3776 Lineage=Root;rootrank;Bacteria;domain;"Planctomycetes";phylum;"Planctomycetacia";class;Planctomycetales;order;Planctomycetaceae;family;Planctomyces;genus	90.56	9.00E-145	514
1324	0	0	0	1	0	0	0	0	0	Planctomycetes	Planctomycea	Planctomycetales	Planctomycetaceae	Planctomyces	S002289008 Planctomyces limnophilus DSM 3776 Lineage=Root;rootrank;Bacteria;domain;"Planctomycetes";phylum;"Planctomycetacia";class;Planctomycetales;order;Planctomycetaceae;family;Planctomyces;genus	88.97	1.00E-133	477
2983	0	0	0	0	0	0	0	0	1	Planctomycetes	Planctomycea	Planctomycetales	Planctomycetaceae	Planctomyces	S002289008 Planctomyces limnophilus DSM 3776 Lineage=Root;rootrank;Bacteria;domain;"Planctomycetes";phylum;"Planctomycetacia";class;Planctomycetales;order;Planctomycetaceae;family;Planctomyces;genus	90.31	4.00E-143	508
895	0	0	3	2	8	14	17	0	3	Planctomycetes	Planctomycea	Planctomycetales	Planctomycetaceae	Planctomyces	S002410761 Planctomyces brasiliensis; Gr7 Lineage=Root;rootrank;Bacteria;domain;"Planctomycetes";phylum;"Planctomycetacia";class;Planctomycetales;order;Planctomycetaceae;family;Planctomyces;genus	87.43	1.00E-117	424
1767	0	0	0	0	0	4	1	0	0	Planctomycetes	Planctomycea	Planctomycetales	Planctomycetaceae	Planctomyces	S002410761 Planctomyces brasiliensis; Gr7 Lineage=Root;rootrank;Bacteria;domain;"Planctomycetes";phylum;"Planctomycetacia";class;Planctomycetales;order;Planctomycetaceae;family;Planctomyces;genus	87.94	7.00E-121	435
311	0	0	0	4	3	0	0	1	0	Planctomycetes	Planctomycea	Planctomycetales	Planctomycetaceae	Planctomyces	S002952193 planctomycete MS1316 Lineage=Root;rootrank;Bacteria;domain;"Planctomycetes";phylum;"Planctomycetacia";class;Planctomycetales;order;Planctomycetaceae;family;unclassified_Planctomycetaceae;	85.86	1.00E-113	411
388	0	0	1	0	2	3	8	1	1	Planctomycetes	Planctomycea	Planctomycetales	Planctomycetaceae	Planctomyces	S002952218 planctomycete MS3054 Lineage=Root;rootrank;Bacteria;domain;"Planctomycetes";phylum;"Planctomycetacia";class;Planctomycetales;order;Planctomycetaceae;family;Planctomyces;genus	89.97	1.00E-132	473
1413	0	0	0	0	1	0	0	0	0	Planctomycetes	Planctomycea	Planctomycetales	Planctomycetaceae	Planctomyces	S002952218 planctomycete MS3054 Lineage=Root;rootrank;Bacteria;domain;"Planctomycetes";phylum;"Planctomycetacia";class;Planctomycetales;order;Planctomycetaceae;family;Planctomyces;genus	89.69	4.00E-138	492
3247	0	0	0	0	1	0	0	0	0	Planctomycetes	Planctomycea	Planctomycetales	Planctomycetaceae	Planctomyces	S002952218 planctomycete MS3054 Lineage=Root;rootrank;Bacteria;domain;"Planctomycetes";phylum;"Planctomycetacia";class;Planctomycetales;order;Planctomycetaceae;family;Planctomyces;genus	89.18	2.00E-135	483
2643	0	0	0	1	0	0	0	0	0	Planctomycetes	Planctomycea	Planctomycetales	Planctomycetaceae	Planctomyces	S002952218 planctomycete MS3054 Lineage=Root;rootrank;Bacteria;domain;"Planctomycetes";phylum;"Planctomycetacia";class;Planctomycetales;order;Planctomycetaceae;family;Planctomyces;genus	88.04	2.00E-121	436
3220	0	0	0	0	0	0	0	1	0	Planctomycetes	Planctomycea	Planctomycetales	Planctomycetaceae	Planctomyces	S002952218 planctomycete MS3054 Lineage=Root;rootrank;Bacteria;domain;"Planctomycetes";phylum;"Planctomycetacia";class;Planctomycetales;order;Planctomycetaceae;family;Planctomyces;genus	89.85	9.00E-140	497
1976	0	0	4	22	10	54	41	3	10	Planctomycetes	Planctomycea	Planctomycetales	Planctomycetaceae	Planctomyces	S002952219 planctomycete DDSW1318 Lineage=Root;rootrank;Bacteria;domain;"Planctomycetes";phylum;"Planctomycetacia";class;Planctomycetales;order;Planctomycetaceae;family;Planctomyces;genus	88.62	8.00E-125	448
1978	0	0	12	0	1	2	6	33	18	Planctomycetes	Planctomycea	Planctomycetales	Planctomycetaceae	Planctomyces	S002952219 planctomycete DDSW1318 Lineage=Root;rootrank;Bacteria;domain;"Planctomycetes";phylum;"Planctomycetacia";class;Planctomycetales;order;Planctomycetaceae;family;Planctomyces;genus	88.95	1.00E-127	457
998	0	0	1	10	3	3	2	13	9	Planctomycetes	Planctomycea	Planctomycetales	Planctomycetaceae	Planctomyces	S002952219 planctomycete DDSW1318 Lineage=Root;rootrank;Bacteria;domain;"Planctomycetes";phylum;"Planctomycetacia";class;Planctomycetales;order;Planctomycetaceae;family;Planctomyces;genus	91.4	1.00E-142	507

879	0	0	3	4	0	9	8	5	5	Planctomycetes	Planctomycea	Planctomycetales	Planctomycetaceae	Planctomyces	S002952219 planctomycete DDSW1318 Lineage=Root;rootrank;Bacteria;domain;"Planctomycetes";phylum;"Planctomycetacia";class;Planctomycetales;order;Planctomycetaceae;family;Planctomyces;genus	90.3	6.00E-136	484
2088	3	0	4	1	2	2	1	2	0	Planctomycetes	Planctomycea	Planctomycetales	Planctomycetaceae	Planctomyces	S002952219 planctomycete DDSW1318 Lineage=Root;rootrank;Bacteria;domain;"Planctomycetes";phylum;"Planctomycetacia";class;Planctomycetales;order;Planctomycetaceae;family;Planctomyces;genus	89.97	4.00E-133	475
3235	0	0	7	4	3	0	0	0	0	Planctomycetes	Planctomycea	Planctomycetales	Planctomycetaceae	Planctomyces	S002952219 planctomycete DDSW1318 Lineage=Root;rootrank;Bacteria;domain;"Planctomycetes";phylum;"Planctomycetacia";class;Planctomycetales;order;Planctomycetaceae;family;Planctomyces;genus	91.4	1.00E-142	507
964	0	0	2	4	3	2	0	0	1	Planctomycetes	Planctomycea	Planctomycetales	Planctomycetaceae	Planctomyces	S002952219 planctomycete DDSW1318 Lineage=Root;rootrank;Bacteria;domain;"Planctomycetes";phylum;"Planctomycetacia";class;Planctomycetales;order;Planctomycetaceae;family;Planctomyces;genus	88.2	4.00E-123	442
1676	1	2	1	0	1	1	2	0	0	Planctomycetes	Planctomycea	Planctomycetales	Planctomycetaceae	Planctomyces	S002952219 planctomycete DDSW1318 Lineage=Root;rootrank;Bacteria;domain;"Planctomycetes";phylum;"Planctomycetacia";class;Planctomycetales;order;Planctomycetaceae;family;Planctomyces;genus	89.43	8.00E-130	464
2785	0	0	3	1	0	0	0	0	0	Planctomycetes	Planctomycea	Planctomycetales	Planctomycetaceae	Planctomyces	S002952219 planctomycete DDSW1318 Lineage=Root;rootrank;Bacteria;domain;"Planctomycetes";phylum;"Planctomycetacia";class;Planctomycetales;order;Planctomycetaceae;family;Planctomyces;genus	92.2	4.00E-148	525
224	1	0	0	0	0	0	0	0	1	Planctomycetes	Planctomycea	Planctomycetales	Planctomycetaceae	Planctomyces	S002952219 planctomycete DDSW1318 Lineage=Root;rootrank;Bacteria;domain;"Planctomycetes";phylum;"Planctomycetacia";class;Planctomycetales;order;Planctomycetaceae;family;Planctomyces;genus	88.75	4.00E-133	475
3076	0	0	0	1	1	0	0	0	0	Planctomycetes	Planctomycea	Planctomycetales	Planctomycetaceae	Planctomyces	S002952219 planctomycete DDSW1318 Lineage=Root;rootrank;Bacteria;domain;"Planctomycetes";phylum;"Planctomycetacia";class;Planctomycetales;order;Planctomycetaceae;family;Planctomyces;genus	92.99	5.00E-152	538
803	0	0	0	2	0	0	0	0	0	Planctomycetes	Planctomycea	Planctomycetales	Planctomycetaceae	Planctomyces	S002952219 planctomycete DDSW1318 Lineage=Root;rootrank;Bacteria;domain;"Planctomycetes";phylum;"Planctomycetacia";class;Planctomycetales;order;Planctomycetaceae;family;Planctomyces;genus	91.37	2.00E-141	503
1475	0	0	0	0	1	0	0	0	0	Planctomycetes	Planctomycea	Planctomycetales	Planctomycetaceae	Planctomyces	S002952219 planctomycete DDSW1318 Lineage=Root;rootrank;Bacteria;domain;"Planctomycetes";phylum;"Planctomycetacia";class;Planctomycetales;order;Planctomycetaceae;family;Planctomyces;genus	91.67	4.00E-143	508
1985	0	0	0	0	1	0	0	0	0	Planctomycetes	Planctomycea	Planctomycetales	Planctomycetaceae	Planctomyces	S002952219 planctomycete DDSW1318 Lineage=Root;rootrank;Bacteria;domain;"Planctomycetes";phylum;"Planctomycetacia";class;Planctomycetales;order;Planctomycetaceae;family;Planctomyces;genus	88.17	4.00E-123	442
2289	0	0	0	0	1	0	0	0	0	Planctomycetes	Planctomycea	Planctomycetales	Planctomycetaceae	Planctomyces	S002952219 planctomycete DDSW1318 Lineage=Root;rootrank;Bacteria;domain;"Planctomycetes";phylum;"Planctomycetacia";class;Planctomycetales;order;Planctomycetaceae;family;Planctomyces;genus	89.74	3.00E-139	496
1499	0	0	0	1	0	0	0	0	0	Planctomycetes	Planctomycea	Planctomycetales	Planctomycetaceae	Planctomyces	S002952219 planctomycete DDSW1318 Lineage=Root;rootrank;Bacteria;domain;"Planctomycetes";phylum;"Planctomycetacia";class;Planctomycetales;order;Planctomycetaceae;family;Planctomyces;genus	89.19	4.00E-128	459
2856	0	0	0	1	0	0	0	0	0	Planctomycetes	Planctomycea	Planctomycetales	Planctomycetaceae	Planctomyces	S002952219 planctomycete DDSW1318 Lineage=Root;rootrank;Bacteria;domain;"Planctomycetes";phylum;"Planctomycetacia";class;Planctomycetales;order;Planctomycetaceae;family;Planctomyces;genus	92.18	1.00E-147	523
2341	0	0	0	0	0	1	0	0	0	Planctomycetes	Planctomycea	Planctomycetales	Planctomycetaceae	Planctomyces	S002952219 planctomycete DDSW1318 Lineage=Root;rootrank;Bacteria;domain;"Planctomycetes";phylum;"Planctomycetacia";class;Planctomycetales;order;Planctomycetaceae;family;Planctomyces;genus	90.59	1.00E-137	490
561	0	0	0	0	0	0	0	1	0	Planctomycetes	Planctomycea	Planctomycetales	Planctomycetaceae	Planctomyces	S002952219 planctomycete DDSW1318 Lineage=Root;rootrank;Bacteria;domain;"Planctomycetes";phylum;"Planctomycetacia";class;Planctomycetales;order;Planctomycetaceae;family;Planctomyces;genus	90.54	2.00E-136	486
1395	0	0	0	0	0	0	0	1	0	Planctomycetes	Planctomycea	Planctomycetales	Planctomycetaceae	Planctomyces	S002952219 planctomycete DDSW1318 Lineage=Root;rootrank;Bacteria;domain;"Planctomycetes";phylum;"Planctomycetacia";class;Planctomycetales;order;Planctomycetaceae;family;Planctomyces;genus	90.75	2.00E-145	516
2918	0	0	0	0	0	0	0	1	0	Planctomycetes	Planctomycea	Planctomycetales	Planctomycetaceae	Planctomyces	S002952219 planctomycete DDSW1318 Lineage=Root;rootrank;Bacteria;domain;"Planctomycetes";phylum;"Planctomycetacia";class;Planctomycetales;order;Planctomycetaceae;family;Planctomyces;genus	90.05	3.00E-134	479
1990	0	0	0	0	0	0	0	0	1	Planctomycetes	Planctomycea	Planctomycetales	Planctomycetaceae	Planctomyces	S002952219 planctomycete DDSW1318 Lineage=Root;rootrank;Bacteria;domain;"Planctomycetes";phylum;"Planctomycetacia";class;Planctomycetales;order;Planctomycetaceae;family;Planctomyces;genus	87.63	9.00E-125	448
3212	0	0	0	0	0	0	0	0	1	Planctomycetes	Planctomycea	Planctomycetales	Planctomycetaceae	Planctomyces	S002952219 planctomycete DDSW1318 Lineage=Root;rootrank;Bacteria;domain;"Planctomycetes";phylum;"Planctomycetacia";class;Planctomycetales;order;Planctomycetaceae;family;Planctomyces;genus	89.26	2.00E-136	486
520	1	0	26	42	76	17	32	19	5	Planctomycetes	Planctomycea	Planctomycetales	Planctomycetaceae	Planctomyces	S002952220 planctomycete DDD3G8 Lineage=Root;rootrank;Bacteria;domain;"Planctomycetes";phylum;"Planctomycetacia";class;Planctomycetales;order;Planctomycetaceae;family;Planctomyces;genus	89.52	6.00E-131	468
192	2	0	14	14	29	14	28	5	18	Planctomycetes	Planctomycea	Planctomycetales	Planctomycetaceae	Planctomyces	S002952220 planctomycete DDD3G8 Lineage=Root;rootrank;Bacteria;domain;"Planctomycetes";phylum;"Planctomycetacia";class;Planctomycetales;order;Planctomycetaceae;family;Planctomyces;genus	88.65	6.00E-126	451
3007	1	17	8	0	1	12	8	2	0	Planctomycetes	Planctomycea	Planctomycetales	Planctomycetaceae	Planctomyces	S002952220 planctomycete DDD3G8 Lineage=Root;rootrank;Bacteria;domain;"Planctomycetes";phylum;"Planctomycetacia";class;Planctomycetales;order;Planctomycetaceae;family;Planctomyces;genus	94.86	3.00E-164	579
1546	0	0	0	0	0	0	0	19	14	Planctomycetes	Planctomycea	Planctomycetales	Planctomycetaceae	Planctomyces	S002952220 planctomycete DDD3G8 Lineage=Root;rootrank;Bacteria;domain;"Planctomycetes";phylum;"Planctomycetacia";class;Planctomycetales;order;Planctomycetaceae;family;Planctomyces;genus	88.2	1.00E-122	440
2930	2	0	1	6	1	1	1	5	3	Planctomycetes	Planctomycea	Planctomycetales	Planctomycetaceae	Planctomyces	S002952220 planctomycete DDD3G8 Lineage=Root;rootrank;Bacteria;domain;"Planctomycetes";phylum;"Planctomycetacia";class;Planctomycetales;order;Planctomycetaceae;family;Planctomyces;genus	89.51	4.00E-138	492
242	0	0	2	6	5	0	0	3	0	Planctomycetes	Planctomycea	Planctomycetales	Planctomycetaceae	Planctomyces	S002952220 planctomycete DDD3G8 Lineage=Root;rootrank;Bacteria;domain;"Planctomycetes";phylum;"Planctomycetacia";class;Planctomycetales;order;Planctomycetaceae;family;Planctomyces;genus	88.71	6.00E-126	451
2969	0	0	0	5	6	0	0	0	0	Planctomycetes	Planctomycea	Planctomycetales	Planctomycetaceae	Planctomyces	S002952220 planctomycete DDD3G8 Lineage=Root;rootrank;Bacteria;domain;"Planctomycetes";phylum;"Planctomycetacia";class;Planctomycetales;order;Planctomycetaceae;family;Planctomyces;genus	87.63	3.00E-125	449
1556	0	0	3	4	3	0	0	0	0	Planctomycetes	Planctomycea	Planctomycetales	Planctomycetaceae	Planctomyces	S002952220 planctomycete DDD3G8 Lineage=Root;rootrank;Bacteria;domain;"Planctomycetes";phylum;"Planctomycetacia";class;Planctomycetales;order;Planctomycetaceae;family;Planctomyces;genus	88.37	3.00E-130	466
3085	0	0	0	0	0	3	5	0	0	Planctomycetes	Planctomycea	Planctomycetales	Planctomycetaceae	Planctomyces	S002952220 planctomycete DDD3G8 Lineage=Root;rootrank;Bacteria;domain;"Planctomycetes";phylum;"Planctomycetacia";class;Planctomycetales;order;Planctomycetaceae;family;Planctomyces;genus	87.94	2.00E-121	436
1923	1	0	0	0	0	1	0	0	0	Planctomycetes	Planctomycea	Planctomycetales	Planctomycetaceae	Planctomyces	S002952220 planctomycete DDD3G8 Lineage=Root;rootrank;Bacteria;domain;"Planctomycetes";phylum;"Planctomycetacia";class;Planctomycetales;order;Planctomycetaceae;family;Planctomyces;genus	87.76	2.00E-126	453
1162	0	1	0	0	2	0	0	0	0	Planctomycetes	Planctomycea	Planctomycetales	Planctomycetaceae	Planctomyces	S002952220 planctomycete DDD3G8 Lineage=Root;rootrank;Bacteria;domain;"Planctomycetes";phylum;"Planctomycetacia";class;Planctomycetales;order;Planctomycetaceae;family;Planctomyces;genus	87.37	1.00E-123	444
2352	0	0	2	0	0	0	1	0	0	Planctomycetes	Planctomycea	Planctomycetales	Planctomycetaceae	Planctomyces	S002952220 planctomycete DDD3G8 Lineage=Root;rootrank;Bacteria;domain;"Planctomycetes";phylum;"Planctomycetacia";class;Planctomycetales;order;Planctomycetaceae;family;Planctomyces;genus	89.7	2.00E-131	470
2834	0	0	0	2	1	0	0	0	0	Planctomycetes	Planctomycea	Planctomycetales	Planctomycetaceae	Planctomyces	S002952220 planctomycete DDD3G8 Lineage=Root;rootrank;Bacteria;domain;"Planctomycetes";phylum;"Planctomycetacia";class;Planctomycetales;order;Planctomycetaceae;family;Planctomyces;genus	88.4	3.00E-130	466
1468	0	0	0	2	0	0	0	1	0	Planctomycetes	Planctomycea	Planctomycetales	Planctomycetaceae	Planctomyces	S002952220 planctomycete DDD3G8 Lineage=Root;rootrank;Bacteria;domain;"Planctomycetes";phylum;"Planctomycetacia";class;Planctomycetales;order;Planctomycetaceae;family;Planctomyces;genus	88.04	2.00E-121	436
71	0	0	0	1	0	0	0	0	0	Planctomycetes	Planctomycea	Planctomycetales	Planctomycetaceae	Planctomyces	S002952220 planctomycete DDD3G8 Lineage=Root;rootrank;Bacteria;domain;"Planctomycetes";phylum;"Planctomycetacia";class;Planctomycetales;order;Planctomycetaceae;family;Planctomyces;genus	87.57	1.00E-118	427
156	0	0	0	0	0	0	1	0	0	Planctomycetes	Planctomycea	Planctomycetales	Planctomycetaceae	Planctomyces	S002952220 planctomycete DDD3G8 Lineage=Root;rootrank;Bacteria;domain;"Planctomycetes";phylum;"Planctomycetacia";class;Planctomycetales;order;Planctomycetaceae;family;Planctomyces;genus	88.08	7.00E-121	435
1466	0	0	0	0	0	0	0	1	0	Planctomycetes	Planctomycea	Planctomycetales	Planctomycetaceae	Planctomyces	S002952220 planctomycete DDD3G8 Lineage=Root;rootrank;Bacteria;domain;"Planctomycetes";phylum;"Planctomycetacia";class;Planctomycetales;order;Planctomycetaceae;family;Planctomyces;genus	88.62	8.00E-125	448
436	52	222	49	0	1	21	55	20	10	Planctomycetes	Planctomycea	Planctomycetales	Planctomycetaceae	Planctomyces	S002952222 planctomycete MS3039 Lineage=Root;rootrank;Bacteria;domain;"Planctomycetes";phylum;"Planctomycetacia";class;Planctomycetales;order;Planctomycetaceae;family;unclassified_Planctomycetaceae;	86.77	9.00E-120	431
1687	32	44	48	10	12	17	59	53	11	Planctomycetes	Planctomycea	Planctomycetales	Planctomycetaceae	Planctomyces	S002952222 planctomycete MS3039 Lineage=Root;rootrank;Bacteria;domain;"Planctomycetes";phylum;"Planctomycetacia";class;Planctomycetales;order;Planctomycetaceae;family;unclassified_Planctomycetaceae;	85.97	3.00E-114	412
663	1	4	6	0	0	5	13	8	0	Planctomycetes	Planctomycea	Planctomycetales	Planctomycetaceae	Planctomyces	S002952222 planctomycete MS3039 Lineage=Root;rootrank;Bacteria;domain;"Planctomycetes";phylum;"Planctomycetacia";class;Planctomycetales;order;Planctomycetaceae;family;unclassified_Planctomycetaceae;	86.73	3.00E-119	429
722	0	0	0	1	0	0	0	0	0	Planctomycetes	Planctomycea	Planctomycetales	Planctomycetaceae	Planctomyces	S002952222 planctomycete MS3039 Lineage=Root;rootrank;Bacteria;domain;"Planctomycetes";phylum;"Planctomycetacia";class;Planctomycetales;order;Planctomycetaceae;family;unclassified_Planctomycetaceae;	86.86	1.00E-113	411
1474	0	0	0	0	0	0	1	0	0	Planctomycetes	Planctomycea	Planctomycetales	Planctomycetaceae	Planctomyces	S002952222 planctomycete MS3039 Lineage=Root;rootrank;Bacteria;domain;"Planctomycetes";phylum;"Planctomycetacia";class;Planctomycetales;order;Planctomycetaceae;family;unclassified_Planctomycetaceae;	86.9	1.00E-113	411
314	0	0	0	0	0	0	0	1	0	Planctomycetes	Planctomycea	Planctomycetales	Planctomycetaceae	Planctomyces	S002952222 planctomycete MS3039 Lineage=Root;rootrank;Bacteria;domain;"Planctomycetes";phylum;"Planctomycetacia";class;Planctomycetales;order;Planctomycetaceae;family;unclassified_Planctomycetaceae;	81.17	2.00E-77	291
3121	0	0	0	0	0	1	2	0	2	Planctomycetes	Planctomycea				S000389821 Gemmata-like str. JW11-2f5 Lineage=Root;rootrank;Bacteria;domain;"Planctomycetes";phylum;"Planctomycetacia";class;Planctomycetales;order;Planctomycetaceae;family;Gemmata;genus	84.18	5.00E-97	355
3084	5	0	42	23	26	23	18	4	13	Planctomycetes	Planctomycea				S002410761 Planctomyces brasiliensis; Gr7 Lineage=Root;rootrank;Bacteria;domain;"Planctomycetes";phylum;"Planctomycetacia";class;Planctomycetales;order;Planctomycetaceae;family;Planctomyces;genus	88.2	1.00E-122	440
2537	0	3	13	6	4	1	0	0	1	Planctomycetes	Planctomycea				S002952220 planctomycete DDD3G8 Lineage=Root;rootrank;Bacteria;domain;"Planctomycetes";phylum;"Planctomycetacia";class;Planctomycetales;order;Planctomycetaceae;family;Planctomyces;genus	93.78	1.00E-157	556
2002	0	0	0	1	0	1	1	3	2	Planctomycetes	Planctomycea				S002952220 planctomycete DDD3G8 Lineage=Root;rootrank;Bacteria;domain;"Planctomycetes";phylum;"Planctomycetacia";class;Planctomycetales;order;Planctomycetaceae;family;Planctomyces;genus	92.54	2.00E-146	520
2673	0	1	3	0	0	0	0	0	1	Planctomycetes	Planctomycea				S002952220 planctomycete DDD3G8 Lineage=Root;rootrank;Bacteria;domain;"Planctomycetes";phylum;"Planctomycetacia";class;Planctomycetales;order;Planctomycetaceae;family;Planctomyces;genus	89.28	8.00E-130	464
1188	0	0	1	0	0	0	0	0	0	Planctomycetes	Planctomycea				S002952220 planctomycete DDD3G8 Lineage=Root;rootrank;Bacteria;domain;"Planctomycetes";phylum;"Planctomycetacia";class;Planctomycetales;order;Planctomycetaceae;family;Planctomyces;genus	88.95	1.00E-123	444
379	2	3	21	0	1	16	16	0	0	Planctomycetes	Planctomycea				S002952222 planctomycete MS3039 Lineage=Root;rootrank;Bacteria;domain;"Planctomycetes";phylum;"Planctomycetacia";class;Planctomycetales;order;Planctomycetaceae;family;unclassified_Planctomycetaceae;	87.79	2.00E-126	453
1399	0	0	0	0	0	1	0	0	0	Planctomycetes	Planctomycea				S002952222 planctomycete MS3039 Lineage=Root;rootrank;Bacteria;domain;"Planctomycetes";phylum;"Planctomycetacia";class;Planctomycetales;order;Planctomycetaceae;family;unclassified_Planctomycetaceae;	86.11	3.00E-115	416
1552	0	0	0	0	0	0	1	0	0	Planctomycetes	Planctomycea				S002952222 planctomycete MS3039 Lineage=Root;rootrank;Bacteria;domain;"Planctomycetes";phylum;"Planctomycetacia";class;Planctomycetales;order;Planctomycetaceae;family;unclassified_Planctomycetaceae;	86.11	9.00E-115	414
490	4	0	24	9	12	8	22	39	57	Planctomycetes	Planctomycea				S003614355 Planctomyces brasiliensis; LU4-23 Lineage=Root;rootrank;Bacteria;domain;"Planctomycetes";phylum;"Planctomycetacia";class;Planctomycetales;order;Planctomycetaceae;family;Planctomyces;genus	86.97	4.00E-113	409
2747	0	0	0	0	0	0	1	0	0	Planctomycetes	PW285				S000013736 anaerobic ammonium-oxidizing planctomycete; 14 Lineage=Root;rootrank;Bacteria;domain;unclassified_Bacteria;	82.89	4.00E-88	326
2278	0	0	0	0	1	0	0	3	0	Planctomycetes	PW285				S000626300 Candidatus Jettenia asiatica; AS-1 Lineage=Root;rootrank;Bacteria;domain;unclassified_Bacteria;	83.97	7.00E-96	351
392	0	0	1	0	0	0	0	1	0	Planctomycetes	PW285				S000653977 Candidatus Brocadia fulgida Lineage=Root;rootrank;Bacteria;domain;unclassified_Bacteria;	84.01	7.00E-96	351
2238	0	0	0	1	0	0	0	0	0	Planctomycetes	PW285				S000749609 anaerobic ammonium-oxidizing planctomycete JMK-2 Lineage=Root;rootrank;Bacteria;domain;unclassified_Bacteria;	83.2	7.00E-91	335
1957	0	0	0	1	0	0	0	0	0	Planctomycetes	PW285				S002447728 Candidatus Brocadia caroliniensis; NRRL_B-50286 Lineage=Root;rootrank;Bacteria;domain;unclassified_Bacteria;	83.51	4.00E-93	342
208	0	0	0	2	2	0	0	0	0	Planctomycetes	vadinHA49				S000383909 planctomycete KSU-1 Lineage=Root;rootrank;Bacteria;domain;unclassified_Bacteria;	82.29	1.00E-83	311
1802	0	0	0	2	0	11	5	4	3	Planctomycetes	vadinHA49				S000653977 Candidatus Brocadia fulgida Lineage=Root;rootrank;Bacteria;domain;unclassified_Bacteria;	86.3	1.00E-108	394
1041	0	0	0	1	0	0	0	0	0	Planctomycetes	vadinHA49				S000657470 Candidatus Kuenenia stuttgartiensis Lineage=Root;rootrank;Bacteria;domain;unclassified_Bacteria;	87.57	9.00E-115	414
2017	0	0	0	0	3	0	0	0	0	Planctomycetes	vadinHA49				S000776302 Filomicrobium insigne (T); SLG5B-19 Lineage=Root;rootrank;Bacteria;domain;"Proteobacteria";phylum;Alphaproteobacteria;class;Rhizobiales;order;Hyphomicrobiaceae;family;Filomicrobium;genus	80	1.00E-68	261
1868	0	0	0	0	0	0	0	1	0	Planctomycetes	vadinHA49				S001044362 Candidatus Scalindua marina; 12C Lineage=Root;rootrank;Bacteria;domain;unclassified_Bacteria;	86.23	1.00E-107	390
2378	0	0	0	0	1	5	1	0	1	Planctomycetes	vadinHA49				S002447728 Candidatus Brocadia caroliniensis; NRRL_B-50286 Lineage=Root;rootrank;Bacteria;domain;unclassified_Bacteria;	85.87	2.00E-105	383
2632	0	0	0	1	2	0	0	0	0	Planctomycetes					S000013736 anaerobic ammonium-oxidizing planctomycete; 14 Lineage=Root;rootrank;Bacteria;domain;unclassified_Bacteria;	84.64	2.00E-100	366
1939	0	0	0	7	1	0	0	0	0	Planctomycetes					S000383909 planctomycete KSU-1 Lineage=Root;rootrank;Bacteria;domain;unclassified_Bacteria;	84.91	1.00E-108	394
273	0	0	0	2	0	0	0	0	1	Planctomycetes					S000404727 Candidatus Scalindua brodae; EN 8 Lineage=Root;rootrank;Bacteria;domain;unclassified_Bacteria;	83.08	3.00E-95	350
248	0	0	0	0	1	0	0	0	0	Planctomycetes					S000571483 planctomycete A-2 Lineage=Root;rootrank;Bacteria;domain;"Planctomycetes";phylum;"Planctomycetacia";class;Planctomycetales;order;Planctomycetaceae;family;unclassified_Planctomycetaceae;	82.78	3.00E-94	346
3107	0	0	0	0	0	1	0	0	0	Planctomycetes					S000653227 Candidatus Anammoxoglobus propionicus Lineage=Root;rootrank;Bacteria;domain;unclassified_Bacteria;	83.47	5.00E-92	339
64	0	0	0	1	0	0	0	1	0	Planctomycetes					S000653977 Candidatus Brocadia fulgida Lineage=Root;rootrank;Bacteria;domain;unclassified_Bacteria;	84.51	6.00E-102	372
877	0	0	2	9	20	26	52	10	7	Planctomycetes					S000657470 Candidatus Kuenenia stuttgartiensis Lineage=Root;rootrank;Bacteria;domain;unclassified_Bacteria;	86.23	4.00E-108	392
125	0	0	0	0	0	0	0	0	1	Planctomycetes					S000657470 Candidatus Kuenenia stuttgartiensis Lineage=Root;rootrank;Bacteria;domain;unclassified_Bacteria;	80.81	3.00E-74	279
1823	0	0	0	0	0	0	0	0	1	Planctomycetes					S002447728 Candidatus Brocadia caroliniensis; NRRL_B-50286 Lineage=Root;rootrank;Bacteria;domain;unclassified_Bacteria;	86.49	2.00E-116	420
2634	0	0	0	0	0	5	11	3	4	Planctomycetes					S002952220 planctomycete DDD3G8 Lineage=Root;rootrank;Bacteria;domain;"Planctomycetes";phylum;"Planctomycetacia";class;Planctomycetales;order;Planctomycetaceae;family;Planctomyces;genus	92.7	6.00E-151	534
1035	0	0	0	0	0	1	0	0	0	pMC2A5					S003659734 Haloplanus natans; AS96 Lineage=Root;rootrank;Archaea;domain;"Euryarchaeota";phylum;Halobacteria;class;Halobacteriales;order;Halobacteriaceae;family;Haloplanus;genus	73.53	1.00E-29	132
1103	0	0	1	8	7	9	2	3	7	Proteobacteria	Alphaproteobacteria				S000925329 Rhizomicrobium palustre (T); A48 Lineage=Root;rootrank;Bacteria;domain;"Proteobacteria";phylum;Alphaproteobacteria;class;Alphaproteobacteria_incertae_sedis;order;Rhizomicrobium;genus	90.57	1.00E-137	490
962	0	0	2	5	11	3	1	3	3	Proteobacteria	Alphaproteobacteria				S000925329 Rhizomicrobium palustre (T); A48 Lineage=Root;rootrank;Bacteria;domain;"Proteobacteria";phylum;Alphaproteobacteria;class;Alphaproteobacteria_incertae_sedis;order;Rhizomicrobium;genus	92.2	1.00E-147	523
2811	0	0	0	2	5	1	3	1	2	Proteobacteria	Alphaproteobacteria				S000925329 Rhizomicrobium palustre (T); A48 Lineage=Root;rootrank;Bacteria;domain;"Proteobacteria";phylum;Alphaproteobacteria;class;Alphaproteobacteria_incertae_sedis;order;Rhizomicrobium;genus	92.43	3.00E-149	529
2809	0	0	0	2	1	1	0	6	5	Proteobacteria	Alphaproteobacteria				S003745514 Hyphomicrobium aestuarii; ATCC 27483 Lineage=Root;rootrank;Bacteria;domain;"Proteobacteria";phylum;Alphaproteobacteria;class;Rhizobiales;order;Hyphomicrobiaceae;family;Hyphomicrobium;genus	88.68	7.00E-126	451
313	0	0	0	3	0	0	0	0	0	Proteobacteria	Alphaproteobacteria	Caulobacterales	Caulobacteraceae		S000112395 Caulobacter mirabilis (T); type strain: FWC 38 Lineage=Root;rootrank;Bacteria;domain;"Proteobacteria";phylum;Alphaproteobacteria;class;Caulobacterales;order;Caulobacteraceae;family;Caulobacter;genus	96.92	0	652
1197	0	0	0	0	0	0	0	1	2	Proteobacteria	Alphaproteobacteria	Caulobacterales	Caulobacteraceae		S000112395 Caulobacter mirabilis (T); type strain: FWC 38 Lineage=Root;rootrank;Bacteria;domain;"Proteobacteria";phylum;Alphaproteobacteria;class;Caulobacterales;order;Caulobacteraceae;family;Caulobacter;genus	95.64	4.00E-178	625
1993	0	0	0	4	5	2	0	0	0	Proteobacteria	Alphaproteobacteria	Caulobacterales	Caulobacteraceae	Brevundimonas	S000516043 Brevundimonas kwangchunensis (T); KSL-102 Lineage=Root;rootrank;Bacteria;domain;"Proteobacteria";phylum;Alphaproteobacteria;class;Caulobacterales;order;Caulobacteraceae;family;Brevundimonas;genus	100	0	684
295	0	0	0	2	0	0	0	0	1	Proteobacteria	Alphaproteobacteria	Caulobacterales	Caulobacteraceae	Phenylobacterium	S002447466 Caulobacter fusiformis; DWJ1 Lineage=Root;rootrank;Bacteria;domain;"Proteobacteria";phylum;Alphaproteobacteria;class;Caulobacterales;order;Caulobacteraceae;family;Caulobacter;genus	96.76	6.00E-176	617
2064	1	0	4	2	2	1	1	1	3	Proteobacteria	Alphaproteobacteria	Rhizobiales			S000858544 Rhodobium orientis; JA208 Lineage=Root;rootrank;Bacteria;domain;"Proteobacteria";phylum;Alphaproteobacteria;class;Rhizobiales;order;Rhodobiaceae;family;Rhodobium;genus	95.12	2.00E-165	582
1744	0	0	0	1	0	0	0	1	0	Proteobacteria	Alphaproteobacteria	Rhizobiales			S001099356 Labrenzia aggregata; D7 Lineage=Root;rootrank;Bacteria;domain;"Proteobacteria";phylum;Alphaproteobacteria;class;Rhodobacterales;order;Rhodobacteraceae;family;Labrenzia;genus	93.07	1.00E-153	544
1210	0	0	0	16	8	2	1	8	6	Proteobacteria	Alphaproteobacteria	Rhizobiales			S001328552 Bauldia consociata (T); 11 Lineage=Root;rootrank;Bacteria;domain;"Proteobacteria";phylum;Alphaproteobacteria;class;Rhizobiales;order;Rhizobiales_incertae_sedis;family;Bauldia;genus	95.96	6.00E-171	601
1101	1	1	0	4	5	2	4	3	15	Proteobacteria	Alphaproteobacteria	Rhizobiales			S001328552 Bauldia consociata (T); 11 Lineage=Root;rootrank;Bacteria;domain;"Proteobacteria";phylum;Alphaproteobacteria;class;Rhizobiales;order;Rhizobiales_incertae_sedis;family;Bauldia;genus	97.04	1.00E-177	623
308	0	0	0	0	0	0	0	3	0	Proteobacteria	Alphaproteobacteria	Rhizobiales			S001328552 Bauldia consociata (T); 11 Lineage=Root;rootrank;Bacteria;domain;"Proteobacteria";phylum;Alphaproteobacteria;class;Rhizobiales;order;Rhizobiales_incertae_sedis;family;Bauldia;genus	95.42	1.00E-167	590
2931	1	4	14	26	19	22	23	2	7	Proteobacteria	Alphaproteobacteria	Rhizobiales			S001550319 Amorphus orientalis (T); YIM D10 Lineage=Root;rootrank;Bacteria;domain;"Proteobacteria";phylum;Alphaproteobacteria;class;Rhizobiales;order;Rhizobiales_incertae_sedis;family;Amorphus;genus	95.13	2.00E-175	616
787	0	0	0	1	3	0	0	0	0	Proteobacteria	Alphaproteobacteria	Rhizobiales			S000711714 Amorphus coralli (T); RS.Sph.026 Lineage=Root;rootrank;Bacteria;domain;"Proteobacteria";phylum;Alphaproteobacteria;class;Rhizobiales;order;Rhizobiales_incertae_sedis;family;Amorphus;genus	94.88	4.00E-163	575
292	0	0	0	1	2	0	0	0	0	Proteobacteria	Alphaproteobacteria	Rhizobiales	Aurantimonadaceae		S003298351 Aureimonas ferruginea; CC-CFT023 Lineage=Root;rootrank;Bacteria;domain;"Proteobacteria";phylum;Alphaproteobacteria;class;Rhizobiales;order;"Aurantimonadaceae";family;Aurantimonas;genus	97.84	0	640
770	0	0	2	0	0	0	0	1	0	Proteobacteria	Alphaproteobacteria	Rhizobiales	Aurantimonadaceae		S003290745 Aureimonas jatrophae; L7-484 Lineage=Root;rootrank;Bacteria;domain;"Proteobacteria";phylum;Alphaproteobacteria;class;Rhizobiales;order;"Aurantimonadaceae";family;Aurantimonas;genus	95.14	2.00E-165	582
1309	0	1	0	0	0	0	0	0	0	Proteobacteria	Alphaproteobacteria	Rhizobiales	Beijerinckiaceae	Chelatococcus	S000979341 Chelatococcus daeguensis; S22-38 Lineage=Root;rootrank;Bacteria;domain;"Proteobacteria";phylum;Alphaproteobacteria;class;Rhizobiales;order;Beijerinckiaceae;family;Chelatococcus;genus	99.73	0	678
1127	0	0	1	2	2	0	0	0	0	Proteobacteria	Alphaproteobacteria	Rhizobiales	Bradyrhizobiaceae		S003616328 Bradyrhizobium jicamae; BJC15-C39 Lineage=Root;rootrank;Bacteria;domain;"Proteobacteria";phylum;Alphaproteobacteria;class;Rhizobiales;order;Bradyrhizobiaceae;family;Bradyrhizobium;genus	99.46	0	673
1002	0	0	0	1	0	0	0	1	0	Proteobacteria	Alphaproteobacteria	Rhizobiales	Brucellaceae	Ochrobactrum	S003751671 Ochrobactrum cytisi; FLN6 Lineage=Root;rootrank;Bacteria;domain;"Proteobacteria";phylum;Alphaproteobacteria;class;Rhizobiales;order;Brucellaceae;family;Ochrobactrum;genus	99.49	0	708
2957	0	0	0	1	0	0	0	0	0	Proteobacteria	Alphaproteobacteria	Rhizobiales	Hyphomicrobiaceae		S001097925 Kaistia adipata; NF1 Lineage=Root;rootrank;Bacteria;domain;"Proteobacteria";phylum;Alphaproteobacteria;class;Rhizobiales;order;Rhizobiaceae;family;Kaistia;genus	95.38	2.00E-176	619
2824	0	0	0	5	1	0	0	0	0	Proteobacteria	Alphaproteobacteria	Rhizobiales	Hyphomicrobiaceae	Devosia	S000440546 Devosia limi (T); type strain: R-21940 = LMG 22951 Lineage=Root;rootrank;Bacteria;domain;"Proteobacteria";phylum;Alphaproteobacteria;class;Rhizobiales;order;Hyphomicrobiaceae;family;Devosia;genus	98.46	0	686
3111	0	0	0	0	0	0	0	0	1	Proteobacteria	Alphaproteobacteria	Rhizobiales	Hyphomicrobiaceae	Devosia	S000440546 Devosia limi (T); type strain: R-21940 = LMG 22951 Lineage=Root;rootrank;Bacteria;domain;"Proteobacteria";phylum;Alphaproteobacteria;class;Rhizobiales;order;Hyphomicrobiaceae;family;Devosia;genus	94.87	4.00E-173	608
1846	0	2	4	11	13	0	2	20	50	Proteobacteria	Alphaproteobacteria	Rhizobiales	Hyphomicrobiaceae	Devosia	S000825085 Devosia chinhatensis (T); IPL18 Lineage=Root;rootrank;Bacteria;domain;"Proteobacteria";phylum;Alphaproteobacteria;class;Rhizobiales;order;Hyphomicrobiaceae;family;Devosia;genus	100	0	684
2799	0	0	1	8	5	1	0	0	1	Proteobacteria	Alphaproteobacteria	Rhizobiales	Hyphomicrobiaceae	Devosia	S002230137 Devosia glacialis; Cr4-44 Lineage=Root;rootrank;Bacteria;domain;"Proteobacteria";phylum;Alphaproteobacteria;class;Rhizobiales;order;Hyphomicrobiaceae;family;Devosia;genus	97.03	1.00E-177	623
1206	0	0	0	6	23	5	8	3	6	Proteobacteria	Alphaproteobacteria	Rhizobiales	Hyphomicrobiaceae	Devosia	S003263656 Vasilyevaea enhydra; NBRC 102409 Lineage=Root;rootrank;Bacteria;domain;"Proteobacteria";phylum;Alphaproteobacteria;class;Rhizobiales;order;Rhizobiales_incertae_sedis;family;Vasilyevaea;genus	96.22	1.00E-172	606
3136	0	0	1	7	0	0	1	11	74	Proteobacteria	Alphaproteobacteria	Rhizobiales	Hyphomicrobiaceae	Devosia	S003264329 Devosia soli; NBRC 106426 Lineage=Root;rootrank;Bacteria;domain;"Proteobacteria";phylum;Alphaproteobacteria;class;Rhizobiales;order;Hyphomicrobiaceae;family;Devosia;genus	95.41	1.00E-167	590
2303	0	0	0	0	1	0	0	0	0	Proteobacteria	Alphaproteobacteria	Rhizobiales	Hyphomicrobiaceae	Devosia	S003618578 Devosia neptuniae; CB-281530 Lineage=Root;rootrank;Bacteria;domain;"Proteobacteria";phylum;Alphaproteobacteria;class;Rhizobiales;order;Hyphomicrobiaceae;family;Devosia;genus	96.49	1.00E-173	610
2337	0	0	1	0	2	0	0	0	0	Proteobacteria	Alphaproteobacteria	Rhizobiales	Hyphomicrobiaceae	Devosia	S003720403 Devosia ginsengisoli; SBR7 Lineage=Root;rootrank;Bacteria;domain;"Proteobacteria";phylum;Alphaproteobacteria;class;Rhizobiales;order;Hyphomicrobiaceae;family;Devosia;genus	97.05	4.00E-178	625
396	0	0	0	1	0	0	0	0	0	Proteobacteria	Alphaproteobacteria	Rhizobiales	Hyphomicrobiaceae	Devosia	S003748951 Devosia glacialis; CH1-30 Lineage=Root;rootrank;Bacteria;domain;"Proteobacteria";phylum;Alphaproteobacteria;class;Rhizobiales;order;Hyphomicrobiaceae;family;Devosia;genus	97.69	0	669
2389	0	0	0	0	0	0	0	0	2	Proteobacteria	Alphaproteobacteria	Rhizobiales	Hyphomicrobiaceae	Rhodomicrobium	S002910555 Rhodomicrobium vannielii; K-1 Lineage=Root;rootrank;Bacteria;domain;"Proteobacteria";phylum;Alphaproteobacteria;class;Rhizobiales;order;Hyphomicrobiaceae;family;Rhodomicrobium;genus	99.23	0	704
2344	0	0	0	17	7	0	7	4	3	Proteobacteria	Alphaproteobacteria	Rhizobiales	Hyphomicrobiaceae	Rhodoplanes	S000537534 Pseudolabrys taiwanensis (T); CC-BB4 Lineage=Root;rootrank;Bacteria;domain;"Proteobacteria";phylum;Alphaproteobacteria;class;Rhizobiales;order;Xanthobacteraceae;family;Pseudolabrys;genus	96.76	6.00E-176	617
3216	0	0	0	2	4	0	1	1	0	Proteobacteria	Alphaproteobacteria	Rhizobiales	Hyphomicrobiaceae	Rhodoplanes	S000537534 Pseudolabrys taiwanensis (T); CC-BB4 Lineage=Root;rootrank;Bacteria;domain;"Proteobacteria";phylum;Alphaproteobacteria;class;Rhizobiales;order;Xanthobacteraceae;family;Pseudolabrys;genus	96.14	0	636
450	1	1	4	93	33	15	13	7	21	Proteobacteria	Alphaproteobacteria	Rhizobiales	Hyphomicrobiaceae	Rhodoplanes	S001169708 Pseudolabrys taiwanensis; KIS20-7 Lineage=Root;rootrank;Bacteria;domain;"Proteobacteria";phylum;Alphaproteobacteria;class;Rhizobiales;order;Xanthobacteraceae;family;Pseudolabrys;genus	95.63	4.00E-178	625
2076	0	0	0	8	0	1	1	0	1	Proteobacteria	Alphaproteobacteria	Rhizobiales	Hyphomicrobiaceae		S000016421 Hyphomicrobium hollandicum (T); IFAM KB-677 Lineage=Root;rootrank;Bacteria;domain;"Proteobacteria";phylum;Alphaproteobacteria;class;Rhizobiales;order;Hyphomicrobiaceae;family;Hyphomicrobium;genus	95.37	5.00E-177	621
3150	4	12	8	30	47	35	42	20	47	Proteobacteria	Alphaproteobacteria	Rhizobiales	Hyphomicrobiaceae		S000016449 Filomicrobium fusiforme (T); DSM 5304 Lineage=Root;rootrank;Bacteria;domain;"Proteobacteria";phylum;Alphaproteobacteria;class;Rhizobiales;order;Hyphomicrobiaceae;family;Filomicrobium;genus	98.11	0	645
59	0	0	0	0	0	1	0	0	0	Proteobacteria	Alphaproteobacteria	Rhizobiales	Hyphomicrobiaceae		S002910555 Rhodomicrobium vannielii; K-1 Lineage=Root;rootrank;Bacteria;domain;"Proteobacteria";phylum;Alphaproteobacteria;class;Rhizobiales;order;Hyphomicrobiaceae;family;Rhodomicrobium;genus	94.96	2.00E-166	586
1965	0	0	1	3	0	0	0	0	0	Proteobacteria	Alphaproteobacteria	Rhizobiales	Hyphomicrobiaceae		S003262470 Hyphomicrobium methylovorum; NBRC 14180 Lineage=Root;rootrank;Bacteria;domain;"Proteobacteria";phylum;Alphaproteobacteria;class;Rhizobiales;order;Hyphomicrobiaceae;family;Hyphomicrobium;genus	99.19	0	667
2971	0	0	0	1	0	0	0	0	0	Proteobacteria	Alphaproteobacteria	Rhizobiales	Hyphomicrobiaceae		S003745514 Hyphomicrobium aestuarii; ATCC 27483 Lineage=Root;rootrank;Bacteria;domain;"Proteobacteria";phylum;Alphaproteobacteria;class;Rhizobiales;order;Hyphomicrobiaceae;family;Hyphomicrobium;genus	95.64	4.00E-178	625
304	0	0	0	1	0	0	0	0	0	Proteobacteria	Alphaproteobacteria	Rhizobiales	Hyphomicrobiaceae		S003745514 Hyphomicrobium aestuarii; ATCC 27483 Lineage=Root;rootrank;Bacteria;domain;"Proteobacteria";phylum;Alphaproteobacteria;class;Rhizobiales;order;Hyphomicrobiaceae;family;Hyphomicrobium;genus	96.01	5.00E-172	604
1647	0	0	0	0	0	0	0	1	0	Proteobacteria	Alphaproteobacteria	Rhizobiales	Methylobacteriaceae		S000979341 Chelatococcus daeguensis; S22-38 Lineage=Root;rootrank;Bacteria;domain;"Proteobacteria";phylum;Alphaproteobacteria;class;Rhizobiales;order;Beijerinckiaceae;family;Chelatococcus;genus	95.12	8.00E-175	614
2026	0	0	0	0	0	0	0	1	0	Proteobacteria	Alphaproteobacteria	Rhizobiales	Methylobacteriaceae	Methylobacterium	S003745401 Bosea massiliensis; Tibetlhz-50 Lineage=Root;rootrank;Bacteria;domain;"Proteobacteria";phylum;Alphaproteobacteria;class;Rhizobiales;order;Bradyrhizobiaceae;family;Bosea;genus	94.05	3.00E-159	562
265	0	0	1	6	1	0	0	0	1	Proteobacteria	Alphaproteobacteria	Rhizobiales	Methylocystaceae		S000014117 Methylocystis echinoides (T); IMET 10491 Lineage=Root;rootrank;Bacteria;domain;"Proteobacteria";phylum;Alphaproteobacteria;class;Rhizobiales;order;Methylocystaceae;family;Methylocystis;genus	94.32	6.00E-161	568
930	10	5	5	12	14	2	5	7	6	Proteobacteria	Alphaproteobacteria	Rhizobiales	Phyllobacteriaceae		S000338803 Methylocystis parvus; pAMC269 Lineage=Root;rootrank;Bacteria;domain;"Proteobacteria";phylum;Alphaproteobacteria;class;Rhizobiales;order;Methylocystaceae;family;Methylocystis;genus	94.29	8.00E-160	564
1811	0	0	0	2	0	0	0	0	2	Proteobacteria	Alphaproteobacteria	Rhizobiales	Phyllobacteriaceae		S000539216 Nesiotobacter exalbescens (T); LA33B Lineage=Root;rootrank;Bacteria;domain;"Proteobacteria";phylum;Alphaproteobacteria;class;Rhodobacterales;order;Rhodobacteraceae;family;Nesiotobacter;genus	92.12	2.00E-146	520
2741	1	5	16	53	40	72	71	23	35	Proteobacteria	Alphaproteobacteria	Rhizobiales	Phyllobacteriaceae		S000858544 Rhodobium orientis; JA208 Lineage=Root;rootrank;Bacteria;domain;"Proteobacteria";phylum;Alphaproteobacteria;class;Rhizobiales;order;Rhodobiaceae;family;Rhodobium;genus	94.57	2.00E-161	569
3132	84	76	161	153	191	287	313	161	196	Proteobacteria	Alphaproteobacteria	Rhizobiales	Phyllobacteriaceae		S001550319 Amorphus orientalis (T); YIM D10 Lineage=Root;rootrank;Bacteria;domain;"Proteobacteria";phylum;Alphaproteobacteria;class;Rhizobiales;order;Rhizobiales_incertae_sedis;family;Amorphus;genus	93.48	8.00E-155	547
2419	0	0	0	0	0	2	1	0	0	Proteobacteria	Alphaproteobacteria	Rhizobiales	Phyllobacteriaceae		S001550319 Amorphus orientalis (T); YIM D10 Lineage=Root;rootrank;Bacteria;domain;"Proteobacteria";phylum;Alphaproteobacteria;class;Rhizobiales;order;Rhizobiales_incertae_sedis;family;Amorphus;genus	93.99	5.00E-157	555
1290	0	0	0	0	0	0	1	0	0	Proteobacteria	Alphaproteobacteria	Rhizobiales	Phyllobacteriaceae		S001550319 Amorphus orientalis (T); YIM D10 Lineage=Root;rootrank;Bacteria;domain;"Proteobacteria";phylum;Alphaproteobacteria;class;Rhizobiales;order;Rhizobiales_incertae_sedis;family;Amorphus;genus	95.98	5.00E-172	604
1785	6	6	23	32	27	38	53	55	97	Proteobacteria	Alphaproteobacteria	Rhizobiales	Phyllobacteriaceae		S002304636 Dichotomicrobium thermohalophilum; type strain: DSM5002 Lineage=Root;rootrank;Bacteria;domain;"Proteobacteria";phylum;Alphaproteobacteria;class;Rhizobiales;order;unclassified_Rhizobiales;	93.7	1.00E-153	544

2045	0	1	1	78	10	8	14	72	158	Proteobacteria	Alphaproteobacteria	Rhizobiales	Phyllobacteriaceae		S002907986 Kaistia granuli; N106 Lineage=Root;rootrank;Bacteria;domain;"Proteobacteria";phylum;Alphaproteobacteria;class;Rhizobiales;order;Rhizobiaceae;family;Kaistia;genus	94.31	8.00E-160	564
1261	0	0	0	2	2	0	0	0	1	Proteobacteria	Alphaproteobacteria	Rhizobiales	Phyllobacteriaceae		S002907986 Kaistia granuli; N106 Lineage=Root;rootrank;Bacteria;domain;"Proteobacteria";phylum;Alphaproteobacteria;class;Rhizobiales;order;Rhizobiaceae;family;Kaistia;genus	95.11	8.00E-165	580
1674	30	7	2	1	4	2	2	36	9	Proteobacteria	Alphaproteobacteria	Rhizobiales	Phyllobacteriaceae	Chelativorans	S002033886 Tepidamorphus gemmatus (T); CB-27A Lineage=Root;rootrank;Bacteria;domain;"Proteobacteria";phylum;Alphaproteobacteria;class;Rhizobiales;order;Rhodobiaceae;family;Tepidamorphus;genus	95.65	4.00E-168	592
2183	2	0	12	147	74	6	4	11	17	Proteobacteria	Alphaproteobacteria	Rhizobiales	Phyllobacteriaceae	Mesorhizobium	S003619942 Mesorhizobium australicum WSM2073 Lineage=Root;rootrank;Bacteria;domain;"Proteobacteria";phylum;Alphaproteobacteria;class;Rhizobiales;order;Phyllobacteriaceae;family;Mesorhizobium;genus	98.38	0	651
1870	0	0	0	0	6	0	0	1	0	Proteobacteria	Alphaproteobacteria	Rhizobiales	Phyllobacteriaceae	Mesorhizobium	S003747001 Mesorhizobium robiniae; BSCA9 Lineage=Root;rootrank;Bacteria;domain;"Proteobacteria";phylum;Alphaproteobacteria;class;Rhizobiales;order;Phyllobacteriaceae;family;Mesorhizobium;genus	98.92	0	662
453	0	0	3	0	0	2	0	8	14	Proteobacteria	Alphaproteobacteria	Rhizobiales	Phyllobacteriaceae	Nitratireductor	S002034288 Nitratireductor indicus (T); C115 Lineage=Root;rootrank;Bacteria;domain;"Proteobacteria";phylum;Alphaproteobacteria;class;Rhizobiales;order;Phyllobacteriaceae;family;Nitratireductor;genus	97.3	1.00E-178	627
1619	0	0	0	0	0	0	0	0	1	Proteobacteria	Alphaproteobacteria	Rhizobiales	Phyllobacteriaceae	Parvibaculum	S002287949 Parvibaculum lavamentivorans DS-1 Lineage=Root;rootrank;Bacteria;domain;"Proteobacteria";phylum;Alphaproteobacteria;class;Rhizobiales;order;Rhodobiaceae;family;Parvibaculum;genus	97.67	0	665
1202	0	1	9	34	47	0	0	7	19	Proteobacteria	Alphaproteobacteria	Rhizobiales	Phyllobacteriaceae		S001152544 Aliihoeflea aestuarii; N8 Lineage=Root;rootrank;Bacteria;domain;"Proteobacteria";phylum;Alphaproteobacteria;class;Rhizobiales;order;Phyllobacteriaceae;family;unclassified_Phyllobacteriaceae;	98.92	0	662
1458	1	0	0	15	4	2	1	2	14	Proteobacteria	Alphaproteobacteria	Rhizobiales	Phyllobacteriaceae		S003298524 Nitratireductor kimnyeongensis; OW16 Lineage=Root;rootrank;Bacteria;domain;"Proteobacteria";phylum;Alphaproteobacteria;class;Rhizobiales;order;Phyllobacteriaceae;family;Nitratireductor;genus	97.03	5.00E-177	621
1649	0	2	9	14	14	3	5	22	32	Proteobacteria	Alphaproteobacteria	Rhizobiales	Phyllobacteriaceae		S003620311 Mesorhizobium thiogangeticum; BSN58 Lineage=Root;rootrank;Bacteria;domain;"Proteobacteria";phylum;Alphaproteobacteria;class;Rhizobiales;order;Phyllobacteriaceae;family;Mesorhizobium;genus	98.2	0	680
3180	0	0	0	0	0	2	0	0	0	Proteobacteria	Alphaproteobacteria	Rhizobiales	Rhizobiaceae	Shinella	S003286699 Shinella zoogloeoides; 81g Lineage=Root;rootrank;Bacteria;domain;"Proteobacteria";phylum;Betaproteobacteria;class;Rhodocyclales;order;Rhodocyclaceae;family;Shinella;genus	99.49	0	708
3026	0	0	1	8	1	0	0	1	1	Proteobacteria	Alphaproteobacteria	Rhizobiales	Rhizobiaceae		S003750859 Ensifer adhaerens; DR242 Lineage=Root;rootrank;Bacteria;domain;"Proteobacteria";phylum;Alphaproteobacteria;class;Rhizobiales;order;Rhizobiaceae;family;Ensifer;genus	100	0	684
1588	0	0	0	0	0	0	1	0	0	Proteobacteria	Alphaproteobacteria	Rhizobiales	Rhodobiaceae		S000019650 Rhodovulum robiginosum (T); N2 Lineage=Root;rootrank;Bacteria;domain;"Proteobacteria";phylum;Alphaproteobacteria;class;Rhodobacterales;order;Rhodobacteraceae;family;Rhodovulum;genus	93.02	2.00E-160	566
3144	0	0	1	4	6	0	0	0	1	Proteobacteria	Alphaproteobacteria	Rhizobiales	Rhodobiaceae		S000542978 Pleomorphomonas oryzae; F-4 Lineage=Root;rootrank;Bacteria;domain;"Proteobacteria";phylum;Alphaproteobacteria;class;Rhizobiales;order;Methylocystaceae;family;Pleomorphomonas;genus	93.61	9.00E-165	580
3268	0	0	5	27	17	2	0	1	10	Proteobacteria	Alphaproteobacteria	Rhizobiales	Rhodobiaceae		S000711714 Amorphus coralli (T); RS.Sph.026 Lineage=Root;rootrank;Bacteria;domain;"Proteobacteria";phylum;Alphaproteobacteria;class;Rhizobiales;order;Rhizobiales_incertae_sedis;family;Amorphus;genus	94.32	6.00E-161	568
1593	0	0	2	15	14	0	0	0	0	Proteobacteria	Alphaproteobacteria	Rhizobiales	Rhodobiaceae		S002304674 Afifella pfennigii; type strain: DSM17143 Lineage=Root;rootrank;Bacteria;domain;"Proteobacteria";phylum;Alphaproteobacteria;class;Rhizobiales;order;Rhodobiaceae;family;Afifella;genus	92.43	3.00E-149	529
471	82	50	154	58	108	178	197	8	22	Proteobacteria	Alphaproteobacteria	Rhizobiales			S000016421 Hyphomicrobium hollandicum (T); IFAM KB-677 Lineage=Root;rootrank;Bacteria;domain;"Proteobacteria";phylum;Alphaproteobacteria;class;Rhizobiales;order;Hyphomicrobiaceae;family;Hyphomicrobium;genus	94.31	2.00E-160	566
821	0	0	0	0	0	1	0	0	0	Proteobacteria	Alphaproteobacteria	Rhizobiales			S000016449 Filomicrobium fusiforme (T); DSM 5304 Lineage=Root;rootrank;Bacteria;domain;"Proteobacteria";phylum;Alphaproteobacteria;class;Rhizobiales;order;Hyphomicrobiaceae;family;Filomicrobium;genus	94.95	2.00E-165	582
2355	1	4	0	0	2	1	0	2	4	Proteobacteria	Alphaproteobacteria	Rhizobiales			S000388225 Elbe River snow isolate Iso39; Iso39_948 Lineage=Root;rootrank;Bacteria;domain;"Proteobacteria";phylum;Alphaproteobacteria;class;Rhizobiales;order;Phyllobacteriaceae;family;Hoeflea;genus	96.74	8.00E-175	614
358	0	0	0	0	0	0	0	3	1	Proteobacteria	Alphaproteobacteria	Rhizobiales			S000388225 Elbe River snow isolate Iso39; Iso39_948 Lineage=Root;rootrank;Bacteria;domain;"Proteobacteria";phylum;Alphaproteobacteria;class;Rhizobiales;order;Phyllobacteriaceae;family;Hoeflea;genus	96.17	0	634
2996	7	0	2	12	15	2	5	8	4	Proteobacteria	Alphaproteobacteria	Rhizobiales			S000413605 Rhodoplanes roseus (T); 941 Lineage=Root;rootrank;Bacteria;domain;"Proteobacteria";phylum;Alphaproteobacteria;class;Rhizobiales;order;Hyphomicrobiaceae;family;Rhodoplanes;genus	96.77	2.00E-176	619
2513	0	0	0	0	0	0	0	0	1	Proteobacteria	Alphaproteobacteria	Rhizobiales			S000752409 Xanthobacter agilis (T); SA35 Lineage=Root;rootrank;Bacteria;domain;"Proteobacteria";phylum;Alphaproteobacteria;class;Rhizobiales;order;Xanthobacteraceae;family;Xanthobacter;genus	94.86	4.00E-173	608
1117	0	0	8	0	2	1	0	1	0	Proteobacteria	Alphaproteobacteria	Rhizobiales			S000824449 Mesorhizobium plurifarium; ORS 3324 Lineage=Root;rootrank;Bacteria;domain;"Proteobacteria";phylum;Alphaproteobacteria;class;Rhizobiales;order;Phyllobacteriaceae;family;Mesorhizobium;genus	97.44	0	665
2105	0	3	0	0	0	0	0	12	9	Proteobacteria	Alphaproteobacteria	Rhizobiales			S000843244 Pseudoxanthobacter soli (T); CC4 Lineage=Root;rootrank;Bacteria;domain;"Proteobacteria";phylum;Alphaproteobacteria;class;Rhizobiales;order;Xanthobacteraceae;family;Pseudoxanthobacter;genus	95.16	6.00E-166	584
3147	4	7	27	101	16	79	94	38	25	Proteobacteria	Alphaproteobacteria	Rhizobiales			S000858544 Rhodobium orientis; JA208 Lineage=Root;rootrank;Bacteria;domain;"Proteobacteria";phylum;Alphaproteobacteria;class;Rhizobiales;order;Rhodobiaceae;family;Rhodobium;genus	95.11	8.00E-165	580
2424	0	0	0	5	0	0	1	0	0	Proteobacteria	Alphaproteobacteria	Rhizobiales			S000858544 Rhodobium orientis; JA208 Lineage=Root;rootrank;Bacteria;domain;"Proteobacteria";phylum;Alphaproteobacteria;class;Rhizobiales;order;Rhodobiaceae;family;Rhodobium;genus	95.68	1.00E-168	593
1006	0	0	0	0	2	0	0	0	0	Proteobacteria	Alphaproteobacteria	Rhizobiales			S000858544 Rhodobium orientis; JA208 Lineage=Root;rootrank;Bacteria;domain;"Proteobacteria";phylum;Alphaproteobacteria;class;Rhizobiales;order;Rhodobiaceae;family;Rhodobium;genus	94.05	3.00E-159	562
2090	7	40	16	12	11	12	19	13	17	Proteobacteria	Alphaproteobacteria	Rhizobiales			S001097925 Kaistia adipata; NF1 Lineage=Root;rootrank;Bacteria;domain;"Proteobacteria";phylum;Alphaproteobacteria;class;Rhizobiales;order;Rhizobiaceae;family;Kaistia;genus	94.31	8.00E-160	564
19	0	0	0	3	4	0	0	3	0	Proteobacteria	Alphaproteobacteria	Rhizobiales			S001097925 Kaistia adipata; NF1 Lineage=Root;rootrank;Bacteria;domain;"Proteobacteria";phylum;Alphaproteobacteria;class;Rhizobiales;order;Rhizobiaceae;family;Kaistia;genus	88.97	3.00E-134	479
2563	0	0	0	0	0	0	0	0	1	Proteobacteria	Alphaproteobacteria	Rhizobiales			S001097925 Kaistia adipata; NF1 Lineage=Root;rootrank;Bacteria;domain;"Proteobacteria";phylum;Alphaproteobacteria;class;Rhizobiales;order;Rhizobiaceae;family;Kaistia;genus	93.78	2.00E-156	553
1056	0	18	16	0	6	0	2	33	51	Proteobacteria	Alphaproteobacteria	Rhizobiales			S001168838 Rhizobium oryzae (T); Alt 505 Lineage=Root;rootrank;Bacteria;domain;"Proteobacteria";phylum;Alphaproteobacteria;class;Rhizobiales;order;Rhizobiaceae;family;Rhizobium;genus	97.3	3.00E-179	628
1621	0	3	22	16	41	2	4	35	86	Proteobacteria	Alphaproteobacteria	Rhizobiales			S001169458 Mesorhizobium thiogangeticum; CK-I3-13-1 Lineage=Root;rootrank;Bacteria;domain;"Proteobacteria";phylum;Alphaproteobacteria;class;Rhizobiales;order;Phyllobacteriaceae;family;unclassified_Phyllobacteriaceae;	95.11	8.00E-165	580
2793	0	0	8	16	16	0	0	7	6	Proteobacteria	Alphaproteobacteria	Rhizobiales			S001328552 Bauldia consociata (T); 11 Lineage=Root;rootrank;Bacteria;domain;"Proteobacteria";phylum;Alphaproteobacteria;class;Rhizobiales;order;Rhizobiales_incertae_sedis;family;Bauldia;genus	94.04	4.00E-158	558
216	0	0	0	0	1	0	0	0	0	Proteobacteria	Alphaproteobacteria	Rhizobiales			S001328552 Bauldia consociata (T); 11 Lineage=Root;rootrank;Bacteria;domain;"Proteobacteria";phylum;Alphaproteobacteria;class;Rhizobiales;order;Rhizobiales_incertae_sedis;family;Bauldia;genus	95.19	5.00E-167	588
600	0	0	1	19	0	0	0	2	0	Proteobacteria	Alphaproteobacteria	Rhizobiales			S001550319 Amorphus orientalis (T); YIM D10 Lineage=Root;rootrank;Bacteria;domain;"Proteobacteria";phylum;Alphaproteobacteria;class;Rhizobiales;order;Rhizobiales_incertae_sedis;family;Amorphus;genus	94.06	5.00E-167	588
2353	0	0	0	0	0	0	1	0	0	Proteobacteria	Alphaproteobacteria	Rhizobiales			S001550319 Amorphus orientalis (T); YIM D10 Lineage=Root;rootrank;Bacteria;domain;"Proteobacteria";phylum;Alphaproteobacteria;class;Rhizobiales;order;Rhizobiales_incertae_sedis;family;Amorphus;genus	93.32	4.00E-163	575
2699	0	0	0	0	0	0	0	0	1	Proteobacteria	Alphaproteobacteria	Rhizobiales			S001576671 Breoghania corrubedonensis (T); UBF-P1 Lineage=Root;rootrank;Bacteria;domain;"Proteobacteria";phylum;Alphaproteobacteria;class;Alphaproteobacteria_incertae_sedis;order;Breoghania;genus	95.41	2.00E-176	619
1671	0	0	5	11	9	11	23	4	0	Proteobacteria	Alphaproteobacteria	Rhizobiales			S002033886 Tepidamorphus gemmatus (T); CB-27A Lineage=Root;rootrank;Bacteria;domain;"Proteobacteria";phylum;Alphaproteobacteria;class;Rhizobiales;order;Rhodobiaceae;family;Tepidamorphus;genus	93.77	5.00E-157	555
1438	1	0	0	3	1	0	2	0	1	Proteobacteria	Alphaproteobacteria	Rhizobiales			S002033886 Tepidamorphus gemmatus (T); CB-27A Lineage=Root;rootrank;Bacteria;domain;"Proteobacteria";phylum;Alphaproteobacteria;class;Rhizobiales;order;Rhodobiaceae;family;Tepidamorphus;genus	95.35	2.00E-175	616
2365	1	0	0	0	0	0	0	0	0	Proteobacteria	Alphaproteobacteria	Rhizobiales			S002233491 Blastochloris viridis; DSM 133 Lineage=Root;rootrank;Bacteria;domain;"Proteobacteria";phylum;Alphaproteobacteria;class;Rhizobiales;order;Hyphomicrobiaceae;family;Blastochloris;genus	96.15	0	636
69	0	1	0	0	0	0	0	0	0	Proteobacteria	Alphaproteobacteria	Rhizobiales			S002304636 Dichotomicrobium thermohalophilum; type strain: DSM5002 Lineage=Root;rootrank;Bacteria;domain;"Proteobacteria";phylum;Alphaproteobacteria;class;Rhizobiales;order;unclassified_Rhizobiales;	92.84	4.00E-148	525
1613	0	0	0	0	0	1	0	0	0	Proteobacteria	Alphaproteobacteria	Rhizobiales			S002355142 Rhodoligotrophos appendicifer; ATCC BAA-2115 Lineage=Root;rootrank;Bacteria;domain;"Proteobacteria";phylum;Alphaproteobacteria;class;Rhizobiales;order;unclassified_Rhizobiales;	94.37	9.00E-170	597
3213	0	0	0	0	5	2	0	0	5	Proteobacteria	Alphaproteobacteria	Rhizobiales			S003315125 Oceanicella actignis; PRQ-68 Lineage=Root;rootrank;Bacteria;domain;"Proteobacteria";phylum;Alphaproteobacteria;class;Rhodobacterales;order;Rhodobacteraceae;family;unclassified_Rhodobacteraceae;	93.24	3.00E-154	545
2976	0	0	0	0	1	0	0	0	0	Proteobacteria	Alphaproteobacteria	Rhizobiales			S003716626 Candidatus Nitrosoarchaeum limnia BG20; bBG20-78 Lineage=Root;rootrank;Bacteria;domain;"Proteobacteria";phylum;Alphaproteobacteria;class;Rhizobiales;order;Xanthobacteraceae;family;Pseudolabrys;genus	94.51	4.00E-173	608
2599	0	0	1	4	12	3	5	8	5	Proteobacteria	Alphaproteobacteria	Rhizobiales			S003745514 Hyphomicrobium aestuarii; ATCC 27483 Lineage=Root;rootrank;Bacteria;domain;"Proteobacteria";phylum;Alphaproteobacteria;class;Rhizobiales;order;Hyphomicrobiaceae;family;Hyphomicrobium;genus	96.12	2.00E-180	632
3081	0	0	12	25	19	0	1	39	80	Proteobacteria	Alphaproteobacteria	Rhizobiales			S003745528 Sinorhizobium fredii; WW9 Lineage=Root;rootrank;Bacteria;domain;"Proteobacteria";phylum;Alphaproteobacteria;class;Rhizobiales;order;Rhizobiaceae;family;Ensifer;genus	97.57	0	634
2776	0	0	1	1	0	1	0	5	10	Proteobacteria	Alphaproteobacteria	Rhizobiales			S003748950 Devosia chinhatensis; CH1-29 Lineage=Root;rootrank;Bacteria;domain;"Proteobacteria";phylum;Alphaproteobacteria;class;Rhizobiales;order;Hyphomicrobiaceae;family;Devosia;genus	95.41	1.00E-167	590
1124	0	0	1	4	1	0	0	0	0	Proteobacteria	Alphaproteobacteria	Rhodobacterales			S000981180 Parvularcula lutaonensis (T); CC-MMS-1 Lineage=Root;rootrank;Bacteria;domain;"Proteobacteria";phylum;Alphaproteobacteria;class;"Parvularculales";order;"Parvularculaceae";family;Parvularcula;genus	91.91	6.00E-146	518
2322	0	0	0	0	0	0	0	0	2	Proteobacteria	Alphaproteobacteria	Rhodobacterales			S000981180 Parvularcula lutaonensis (T); CC-MMS-1 Lineage=Root;rootrank;Bacteria;domain;"Proteobacteria";phylum;Alphaproteobacteria;class;"Parvularculales";order;"Parvularculaceae";family;Parvularcula;genus	93.78	1.00E-157	556
884	0	0	1	0	2	0	0	0	0	Proteobacteria	Alphaproteobacteria	Rhodobacterales			S002289490 Parvularcula bermudensis HTCC2503 Lineage=Root;rootrank;Bacteria;domain;"Proteobacteria";phylum;Alphaproteobacteria;class;"Parvularculales";order;"Parvularculaceae";family;Parvularcula;genus	91.26	9.00E-150	531
1098	0	0	0	0	0	0	2	0	0	Proteobacteria	Alphaproteobacteria	Rhodobacterales	Hyphomonadaceae		S002967179 Woodsholea maritima; OTSz_A_272 Lineage=Root;rootrank;Bacteria;domain;"Proteobacteria";phylum;Alphaproteobacteria;class;unclassified_Alphaproteobacteria;	97.17	0	658
3170	0	0	0	0	0	0	0	0	1	Proteobacteria	Alphaproteobacteria	Rhodobacterales	Hyphomonadaceae	Maricaulis	S003263714 Maricaulis maris; NBRC 102484 Lineage=Root;rootrank;Bacteria;domain;"Proteobacteria";phylum;Alphaproteobacteria;class;Caulobacterales;order;Hyphomonadaceae;family;Maricaulis;genus	92.74	2.00E-151	536
2455	0	0	3	15	9	0	1	5	1	Proteobacteria	Alphaproteobacteria	Rhodobacterales	Rhodobacteraceae	Amaricoccus	S000438701 Amaricoccus macauensis (T); Ben104 Lineage=Root;rootrank;Bacteria;domain;"Proteobacteria";phylum;Alphaproteobacteria;class;Rhodobacterales;order;Rhodobacteraceae;family;Amaricoccus;genus	96.4	0	641
63	0	0	0	1	0	0	0	0	0	Proteobacteria	Alphaproteobacteria	Rhodobacterales	Rhodobacteraceae	Amaricoccus	S000438701 Amaricoccus macauensis (T); Ben104 Lineage=Root;rootrank;Bacteria;domain;"Proteobacteria";phylum;Alphaproteobacteria;class;Rhodobacterales;order;Rhodobacteraceae;family;Amaricoccus;genus	96.41	0	641
2728	0	0	0	0	6	0	0	0	1	Proteobacteria	Alphaproteobacteria	Rhodobacterales	Rhodobacteraceae	Amaricoccus	S000902653 Antarcticicola litoralis; IMCC3295 Lineage=Root;rootrank;Bacteria;domain;"Proteobacteria";phylum;Alphaproteobacteria;class;Rhodobacterales;order;Rhodobacteraceae;family;unclassified_Rhodobacteraceae;	95.12	8.00E-175	614
717	0	0	0	1	0	0	0	0	0	Proteobacteria	Alphaproteobacteria	Rhodobacterales	Rhodobacteraceae	Paracoccus	S000539079 Clostridium sardiniense; DSM 600 Lineage=Root;rootrank;Bacteria;domain;Firmicutes;phylum;Clostridia;class;Clostridiales;order;Clostridiaceae 1;family;Clostridium sensu stricto;genus	81.15	1.00E-77	291
1115	0	0	0	0	0	0	0	0	1	Proteobacteria	Alphaproteobacteria	Rhodobacterales	Rhodobacteraceae	Rubellimicrobium	S003264335 Rubellimicrobium aerolatum; NBRC 106433 Lineage=Root;rootrank;Bacteria;domain;"Proteobacteria";phylum;Alphaproteobacteria;class;Rhodobacterales;order;Rhodobacteraceae;family;Rubellimicrobium;genus	96.47	1.00E-172	606
2479	0	0	0	0	0	0	0	0	1	Proteobacteria	Alphaproteobacteria	Rhodobacterales	Rhodobacteraceae		S000438700 Amaricoccus kaplicensis (T); Ben101 Lineage=Root;rootrank;Bacteria;domain;"Proteobacteria";phylum;Alphaproteobacteria;class;Rhodobacterales;order;Rhodobacteraceae;family;Amaricoccus;genus	94.85	1.00E-163	577
2115	0	1	2	2	0	5	1	3	2	Proteobacteria	Alphaproteobacteria	Rhodobacterales	Rhodobacteraceae		S000651283 Paracoccus alcaliphilus; mku-E2 Lineage=Root;rootrank;Bacteria;domain;"Proteobacteria";phylum;Alphaproteobacteria;class;Rhodobacterales;order;Rhodobacteraceae;family;Paracoccus;genus	98.38	0	649
762	4	7	4	1	1	3	9	1	1	Proteobacteria	Alphaproteobacteria	Rhodobacterales	Rhodobacteraceae		S000980064 Rhodovulum thermophilus (T); CC-MHSW-1 Lineage=Root;rootrank;Bacteria;domain;"Proteobacteria";phylum;Alphaproteobacteria;class;Rhodobacterales;order;Rhodobacteraceae;family;Jhaorihella;genus	97.18	0	660
2348	0	4	0	0	0	0	0	0	0	Proteobacteria	Alphaproteobacteria	Rhodobacterales	Rhodobacteraceae		S000980064 Rhodovulum thermophilus (T); CC-MHSW-1 Lineage=Root;rootrank;Bacteria;domain;"Proteobacteria";phylum;Alphaproteobacteria;class;Rhodobacterales;order;Rhodobacteraceae;family;Jhaorihella;genus	95.9	2.00E-180	632
2233	0	5	0	0	0	0	0	9	8	Proteobacteria	Alphaproteobacteria	Rhodobacterales	Rhodobacteraceae		S001198080 Piezobacter thermophilus; 108 Lineage=Root;rootrank;Bacteria;domain;"Proteobacteria";phylum;Alphaproteobacteria;class;Rhodobacterales;order;Rhodobacteraceae;family;Jhaorihella;genus	97.17	0	658
1078	0	0	0	1	0	0	0	1	0	Proteobacteria	Alphaproteobacteria	Rhodobacterales	Rhodobacteraceae		S001588132 Roseovarius pelophilus; HK7 Lineage=Root;rootrank;Bacteria;domain;"Proteobacteria";phylum;Alphaproteobacteria;class;Rhodobacterales;order;Rhodobacteraceae;family;Roseovarius;genus	97.17	0	658
1966	0	0	0	0	0	0	0	1	0	Proteobacteria	Alphaproteobacteria	Rhodobacterales	Rhodobacteraceae		S002449719 Paracoccus alcaliphilus; 36A Lineage=Root;rootrank;Bacteria;domain;"Proteobacteria";phylum;Alphaproteobacteria;class;Rhodobacterales;order;Rhodobacteraceae;family;Paracoccus;genus	99.73	0	676
408	0	0	0	0	0	0	0	1	0	Proteobacteria	Alphaproteobacteria	Rhodobacterales	Rhodobacteraceae		S002950179 Ketogulonigenium vulgarum WSH-001 Lineage=Root;rootrank;Bacteria;domain;"Proteobacteria";phylum;Alphaproteobacteria;class;Rhodobacterales;order;Rhodobacteraceae;family;Ketogulonicigenium;genus	98.21	0	680
1551	0	0	0	0	0	0	0	0	1	Proteobacteria	Alphaproteobacteria	Rhodospirillales	Acetobacteraceae		S002909692 Candidatus Riegeria galatelae; C08086 Lineage=Root;rootrank;Bacteria;domain;"Proteobacteria";phylum;Alphaproteobacteria;class;unclassified_Alphaproteobacteria;	84.95	2.00E-101	370
2324	0	0	0	0	0	0	0	0	3	Proteobacteria	Alphaproteobacteria	Rhodospirillales	Acetobacteraceae	Roseococcus	S001094905 Elioraea tepidiphila (T); TU-7 Lineage=Root;rootrank;Bacteria;domain;"Proteobacteria";phylum;Alphaproteobacteria;class;Alphaproteobacteria_incertae_sedis;order;Elioraea;genus	89.15	4.00E-128	459
872	0	0	0	0	0	0	0	0	1	Proteobacteria	Alphaproteobacteria	Rhodospirillales	Acetobacteraceae	Roseomonas	S001094905 Elioraea tepidiphila (T); TU-7 Lineage=Root;rootrank;Bacteria;domain;"Proteobacteria";phylum;Alphaproteobacteria;class;Alphaproteobacteria_incertae_sedis;order;Elioraea;genus	87.76	5.00E-127	455
1397	0	0	0	0	0	0	0	0	1	Proteobacteria	Alphaproteobacteria	Rhodospirillales	Acetobacteraceae	Roseomonas	S001094905 Elioraea tepidiphila (T); TU-7 Lineage=Root;rootrank;Bacteria;domain;"Proteobacteria";phylum;Alphaproteobacteria;class;Alphaproteobacteria_incertae_sedis;order;Elioraea;genus	90.05	5.00E-142	505
1257	0	0	0	1	1	0	0	0	0	Proteobacteria	Alphaproteobacteria	Rhodospirillales	Rhodospirillaceae		S000011209 Hyphomicrobium zavarzinii (T); ZV-622 Lineage=Root;rootrank;Bacteria;domain;"Proteobacteria";phylum;Alphaproteobacteria;class;Rhizobiales;order;Hyphomicrobiaceae;family;Hyphomicrobium;genus	90.79	4.00E-138	492
2860	0	0	0	2	0	0	0	0	0	Proteobacteria	Alphaproteobacteria	Rhodospirillales	Rhodospirillaceae		S000011209 Hyphomicrobium zavarzinii (T); ZV-622 Lineage=Root;rootrank;Bacteria;domain;"Proteobacteria";phylum;Alphaproteobacteria;class;Rhizobiales;order;Hyphomicrobiaceae;family;Hyphomicrobium;genus	90.32	8.00E-135	481
621	4	0	22	31	18	28	28	8	17	Proteobacteria	Alphaproteobacteria	Rhodospirillales	Rhodospirillaceae		S000019513 Olavius loisae endosymbiont 2 Lineage=Root;rootrank;Bacteria;domain;"Proteobacteria";phylum;Alphaproteobacteria;class;unclassified_Alphaproteobacteria;	90.57	1.00E-137	490
1037	1	0	1	0	0	1	1	0	0	Proteobacteria	Alphaproteobacteria	Rhodospirillales	Rhodospirillaceae		S000019513 Olavius loisae endosymbiont 2 Lineage=Root;rootrank;Bacteria;domain;"Proteobacteria";phylum;Alphaproteobacteria;class;unclassified_Alphaproteobacteria;	93.55	2.00E-156	553
859	0	0	1	0	0	0	0	0	0	Proteobacteria	Alphaproteobacteria	Rhodospirillales	Rhodospirillaceae		S000019513 Olavius loisae endosymbiont 2 Lineage=Root;rootrank;Bacteria;domain;"Proteobacteria";phylum;Alphaproteobacteria;class;unclassified_Alphaproteobacteria;	91.28	2.00E-150	532
148	11	4	40	36	32	107	100	33	73	Proteobacteria	Alphaproteobacteria	Rhodospirillales	Rhodospirillaceae		S000022613 Olavius loisae endosymbiont 3 Lineage=Root;rootrank;Bacteria;domain;"Proteobacteria";phylum;Alphaproteobacteria;class;unclassified_Alphaproteobacteria;	91.69	3.00E-144	512
2622	9	21	16	14	12	29	22	5	10	Proteobacteria	Alphaproteobacteria	Rhodospirillales	Rhodospirillaceae		S000022613 Olavius loisae endosymbiont 3 Lineage=Root;rootrank;Bacteria;domain;"Proteobacteria";phylum;Alphaproteobacteria;class;unclassified_Alphaproteobacteria;	91.03	4.00E-148	525
1699	0	0	4	15	7	11	7	14	12	Proteobacteria	Alphaproteobacteria	Rhodospirillales	Rhodospirillaceae		S000022613 Olavius loisae endosymbiont 3 Lineage=Root;rootrank;Bacteria;domain;"Proteobacteria";phylum;Alphaproteobacteria;class;unclassified_Alphaproteobacteria;	90.3	8.00E-135	481
266	2	4	4	1	7	8	15	8	2	Proteobacteria	Alphaproteobacteria	Rhodospirillales	Rhodospirillaceae		S000022613 Olavius loisae endosymbiont 3 Lineage=Root;rootrank;Bacteria;domain;"Proteobacteria";phylum;Alphaproteobacteria;class;unclassified_Alphaproteobacteria;	90.62	1.00E-137	490
1177	4	13	4	3	7	4	6	1	0	Proteobacteria	Alphaproteobacteria	Rhodospirillales	Rhodospirillaceae		S000022613 Olavius loisae endosymbiont 3 Lineage=Root;rootrank;Bacteria;domain;"Proteobacteria";phylum;Alphaproteobacteria;class;unclassified_Alphaproteobacteria;	89.52	6.00E-131	468
644	0	1	10	6	13	2	3	5	2	Proteobacteria	Alphaproteobacteria	Rhodospirillales	Rhodospirillaceae		S000022613 Olavius loisae endosymbiont 3 Lineage=Root;rootrank;Bacteria;domain;"Proteobacteria";phylum;Alphaproteobacteria;class;unclassified_Alphaproteobacteria;	89.52	6.00E-131	468
3097	2	2	0	0	0	1	2	0	0	Proteobacteria	Alphaproteobacteria	Rhodospirillales	Rhodospirillaceae		S000022613 Olavius loisae endosymbiont 3 Lineage=Root;rootrank;Bacteria;domain;"Proteobacteria";phylum;Alphaproteobacteria;class;unclassified_Alphaproteobacteria;	91.28	9.00E-150	531
2763	1	2	1	0	0	2	2	0	0	Proteobacteria	Alphaproteobacteria	Rhodospirillales	Rhodospirillaceae		S000022613 Olavius loisae endosymbiont 3 Lineage=Root;rootrank;Bacteria;domain;"Proteobacteria";phylum;Alphaproteobacteria;class;unclassified_Alphaproteobacteria;	89.46	1.00E-137	490
1824	0	1	0	0	1	0	0	3	4	Proteobacteria	Alphaproteobacteria	Rhodospirillales	Rhodospirillaceae		S000022613 Olavius loisae endosymbiont 3 Lineage=Root;rootrank;Bacteria;domain;"Proteobacteria";phylum;Alphaproteobacteria;class;unclassified_Alphaproteobacteria;	90.81	1.00E-138	494
215	0	1	4	0	0	0	0	0	0	Proteobacteria	Alphaproteobacteria	Rhodospirillales	Rhodospirillaceae		S000022613 Olavius loisae endosymbiont 3 Lineage=Root;rootrank;Bacteria;domain;"Proteobacteria";phylum;Alphaproteobacteria;class;unclassified_Alphaproteobacteria;	90.05	1.00E-133	477
2133	0	0	0	2	0	0	2	0	1	Proteobacteria	Alphaproteobacteria	Rhodospirillales	Rhodospirillaceae		S000022613 Olavius loisae endosymbiont 3 Lineage=Root;rootrank;Bacteria;domain;"Proteobacteria";phylum;Alphaproteobacteria;class;unclassified_Alphaproteobacteria;	91.03	4.00E-148	525
499	0	0	0	1	3	0	0	0	0	Proteobacteria	Alphaproteobacteria	Rhodospirillales	Rhodospirillaceae		S000022613 Olavius loisae endosymbiont 3 Lineage=Root;rootrank;Bacteria;domain;"Proteobacteria";phylum;Alphaproteobacteria;class;unclassified_Alphaproteobacteria;	90.03	1.00E-133	477
2615	0	1	1	0	0	0	1	0	0	Proteobacteria	Alphaproteobacteria	Rhodospirillales	Rhodospirillaceae		S000022613 Olavius loisae endosymbiont 3 Lineage=Root;rootrank;Bacteria;domain;"Proteobacteria";phylum;Alphaproteobacteria;class;unclassified_Alphaproteobacteria;	92.05	9.00E-155	547
2071	0	0	0	1	0	0	0	0	3	Proteobacteria	Alphaproteobacteria	Rhodospirillales	Rhodospirillaceae		S000022613 Olavius loisae endosymbiont 3 Lineage=Root;rootrank;Bacteria;domain;"Proteobacteria";phylum;Alphaproteobacteria;class;unclassified_Alphaproteobacteria;	90	1.00E-133	477
413	0	0	0	0	2	0	0	0	0	Proteobacteria	Alphaproteobacteria	Rhodospirillales	Rhodospirillaceae		S000022613 Olavius loisae endosymbiont 3 Lineage=Root;rootrank;Bacteria;domain;"Proteobacteria";phylum;Alphaproteobacteria;class;unclassified_Alphaproteobacteria;	88.27	3.00E-130	466
836	0	0	0	1	1	0	0	0	0	Proteobacteria	Alphaproteobacteria	Rhodospirillales	Rhodospirillaceae		S000022613 Olavius loisae endosymbiont 3 Lineage=Root;rootrank;Bacteria;domain;"Proteobacteria";phylum;Alphaproteobacteria;class;unclassified_Alphaproteobacteria;	91.54	2.00E-150	532
2166	0	0	0	0	1	0	0	1	0	Proteobacteria	Alphaproteobacteria	Rhodospirillales	Rhodospirillaceae		S000022613 Olavius loisae endosymbiont 3 Lineage=Root;rootrank;Bacteria;domain;"Proteobacteria";phylum;Alphaproteobacteria;class;unclassified_Alphaproteobacteria;	91.28	3.00E-149	529
1608	0	0	0	0	0	0	2	0	0	Proteobacteria	Alphaproteobacteria	Rhodospirillales	Rhodospirillaceae		S000022613 Olavius loisae endosymbiont 3 Lineage=Root;rootrank;Bacteria;domain;"Proteobacteria";phylum;Alphaproteobacteria;class;unclassified_Alphaproteobacteria;	92.18	1.00E-147	523
820	0	0	0	0	1	0	0	0	0	Proteobacteria	Alphaproteobacteria	Rhodospirillales	Rhodospirillaceae		S000022613 Olavius loisae endosymbiont 3 Lineage=Root;rootrank;Bacteria;domain;"Proteobacteria";phylum;Alphaproteobacteria;class;unclassified_Alphaproteobacteria;	90.88	4.00E-138	492
2427	0	0	0	1	0	0	0	0	0	Proteobacteria	Alphaproteobacteria	Rhodospirillales	Rhodospirillaceae		S000022613 Olavius loisae endosymbiont 3 Lineage=Root;rootrank;Bacteria;domain;"Proteobacteria";phylum;Alphaproteobacteria;class;unclassified_Alphaproteobacteria;	93.24	1.00E-153	544
2684	0	0	0	1	0	0	0	0	0	Proteobacteria	Alphaproteobacteria	Rhodospirillales	Rhodospirillaceae		S000022613 Olavius loisae endosymbiont 3 Lineage=Root;rootrank;Bacteria;domain;"Proteobacteria";phylum;Alphaproteobacteria;class;unclassified_Alphaproteobacteria;	89.01	1.00E-127	457
1997	0	0	0	0	0	1	0	0	0	Proteobacteria	Alphaproteobacteria	Rhodospirillales	Rhodospirillaceae		S000022613 Olavius loisae endosymbiont 3 Lineage=Root;rootrank;Bacteria;domain;"Proteobacteria";phylum;Alphaproteobacteria;class;unclassified_Alphaproteobacteria;	89.19	4.00E-128	459
342	0	0	0	0	0	0	1	0	0	Proteobacteria	Alphaproteobacteria	Rhodospirillales	Rhodospirillaceae		S000022613 Olavius loisae endosymbiont 3 Lineage=Root;rootrank;Bacteria;domain;"Proteobacteria";phylum;Alphaproteobacteria;class;unclassified_Alphaproteobacteria;	92.05	9.00E-155	547
2902	0	0	0	0	0	0	1	0	0	Proteobacteria	Alphaproteobacteria	Rhodospirillales	Rhodospirillaceae		S000022613 Olavius loisae endosymbiont 3 Lineage=Root;rootrank;Bacteria;domain;"Proteobacteria";phylum;Alphaproteobacteria;class;unclassified_Alphaproteobacteria;	90	7.00E-141	501
1800	0	0	0	0	0	0	0	0	1	Proteobacteria	Alphaproteobacteria	Rhodospirillales	Rhodospirillaceae		S000022613 Olavius loisae endosymbiont 3 Lineage=Root;rootrank;Bacteria;domain;"Proteobacteria";phylum;Alphaproteobacteria;class;unclassified_Alphaproteobacteria;	90.03	2.00E-141	503
960	0	0	1	0	1	0	0	0	0	Proteobacteria	Alphaproteobacteria	Rhodospirillales	Rhodospirillaceae		S000112626 Brevundimonas poindexterae (T); FWC40 Lineage=Root;rootrank;Bacteria;domain;"Proteobacteria";phylum;Alphaproteobacteria;class;Caulobacterales;order;Caulobacteraceae;family;Brevundimonas;genus	89.51	4.00E-138	492
2606	1	0	0	0	0	2	0	0	0	Proteobacteria	Alphaproteobacteria	Rhodospirillales	Rhodospirillaceae		S000133119 Blastochloris sulfoviridis; RN1 Lineage=Root;rootrank;Bacteria;domain;"Proteobacteria";phylum;Alphaproteobacteria;class;unclassified_Alphaproteobacteria;	87.6	1.00E-117	424
1082	0	4	1	0	2	0	0	0	0	Proteobacteria	Alphaproteobacteria	Rhodospirillales	Rhodospirillaceae		S000438701 Amaricoccus macauensis (T); Ben104 Lineage=Root;rootrank;Bacteria;domain;"Proteobacteria";phylum;Alphaproteobacteria;class;Rhodobacterales;order;Rhodobacteraceae;family;Amaricoccus;genus	89.19	3.00E-129	462
3205	1	0	0	1	0	6	2	107	89	Proteobacteria	Alphaproteobacteria	Rhodospirillales	Rhodospirillaceae		S000460547 endosymbiont of Chlamys farreri Lineage=Root;rootrank;Bacteria;domain;"Proteobacteria";phylum;Alphaproteobacteria;class;Kiloniellales;order;Kiloniellaceae;family;Kiloniella;genus	88.47	3.00E-124	446
3174	0	0	0	0	0	0	0	0	1	Proteobacteria	Alphaproteobacteria	Rhodospirillales	Rhodospirillaceae		S000871089 Cucumibacter marinus (T); CL-GR60 Lineage=Root;rootrank;Bacteria;domain;"Proteobacteria";phylum;Alphaproteobacteria;class;Rhizobiales;order;Hyphomicrobiaceae;family;Cucumibacter;genus	90.91	3.00E-139	496
3227	1	0	1	0	2	2	1	12	3	Proteobacteria	Alphaproteobacteria	Rhodospirillales	Rhodospirillaceae		S000902653 Antarcticicola litoralis; IMCC3295 Lineage=Root;rootrank;Bacteria;domain;"Proteobacteria";phylum;Alphaproteobacteria;class;Rhodobacterales;order;Rhodobacteraceae;family;unclassified_Rhodobacteraceae;	89.25	3.00E-129	462
2972	0	0	0	2	1	0	0	0	2	Proteobacteria	Alphaproteobacteria	Rhodospirillales	Rhodospirillaceae		S000902653 Antarcticicola litoralis; IMCC3295 Lineage=Root;rootrank;Bacteria;domain;"Proteobacteria";phylum;Alphaproteobacteria;class;Rhodobacterales;order;Rhodobacteraceae;family;unclassified_Rhodobacteraceae;	91.67	3.00E-144	512
2113	0	0	0	1	0	1	0	16	5	Proteobacteria	Alphaproteobacteria	Rhodospirillales	Rhodospirillaceae		S000980064 Rhodovulum thermophilus (T); CC-MHSW-1 Lineage=Root;rootrank;Bacteria;domain;"Proteobacteria";phylum;Alphaproteobacteria;class;Rhodobacterales;order;Rhodobacteraceae;family;Jhaorihella;genus	91.62	3.00E-144	512
2250	0	0	0	0	0	0	0	1	0	Proteobacteria	Alphaproteobacteria	Rhodospirillales	Rhodospirillaceae		S000995281 Reyranella massiliensis; 521 Lineage=Root;rootrank;Bacteria;domain;"Proteobacteria";phylum;Alphaproteobacteria;class;unclassified_Alphaproteobacteria;	97.3	3.00E-179	628
1168	0	0	0	20	8	0	0	0	7	Proteobacteria	Alphaproteobacteria	Rhodospirillales	Rhodospirillaceae		S001094905 Elioraea tepidiphila (T); TU-7 Lineage=Root;rootrank;Bacteria;domain;"Proteobacteria";phylum;Alphaproteobacteria;class;Alphaproteobacteria_incertae_sedis;order;Elioraea;genus	89.76	5.00E-132	472
2819	0	0	0	1	1	0	0	0	0	Proteobacteria	Alphaproteobacteria	Rhodospirillales	Rhodospirillaceae		S001094905 Elioraea tepidiphila (T); TU-7 Lineage=Root;rootrank;Bacteria;domain;"Proteobacteria";phylum;Alphaproteobacteria;class;Alphaproteobacteria_incertae_sedis;order;Elioraea;genus	88.8	1.00E-133	477
2230	0	0	30	24	36	17	27	44	39	Proteobacteria	Alphaproteobacteria	Rhodospirillales	Rhodospirillaceae		S001188390 Parvibaculum indicum (T); P31 Lineage=Root;rootrank;Bacteria;domain;"Proteobacteria";phylum;Alphaproteobacteria;class;Rhizobiales;order;Rhodobiaceae;family;Parvibaculum;genus	88.47	1.00E-123	444
1251	0	0	0	0	0	0	0	1	1	Proteobacteria	Alphaproteobacteria	Rhodospirillales	Rhodospirillaceae		S001188390 Parvibaculum indicum (T); P31 Lineage=Root;rootrank;Bacteria;domain;"Proteobacteria";phylum;Alphaproteobacteria;class;Rhizobiales;order;Rhodobiaceae;family;Parvibaculum;genus	89.52	6.00E-131	468
1898	0	0	0	3	1	0	0	0	3	Proteobacteria	Alphaproteobacteria	Rhodospirillales	Rhodospirillaceae		S001568551 endosymbiont of Ridgeia piscesae; P1s-297 Lineage=Root;rootrank;Bacteria;domain;"Proteobacteria";phylum;unclassified_"Proteobacteria";	89.23	7.00E-136	484
226	0	0	0	5	0	0	0	0	0	Proteobacteria	Alphaproteobacteria	Rhodospirillales	Rhodospirillaceae		S001568551 endosymbiont of Ridgeia piscesae; P1s-297 Lineage=Root;rootrank;Bacteria;domain;"Proteobacteria";phylum;unclassified_"Proteobacteria";	89.26	7.00E-136	484
622	0	0	0	1	0	0	0	0	0	Proteobacteria	Alphaproteobacteria	Rhodospirillales	Rhodospirillaceae		S001575277 Loktanella hongkongensis; AMV3; AMV18 Lineage=Root;rootrank;Bacteria;domain;"Proteobacteria";phylum;Alphaproteobacteria;class;Rhodobacterales;order;Rhodobacteraceae;family;Loktanella;genus	89.78	1.00E-132	473
670	0	0	37	96	64	119	182	6	14	Proteobacteria	Alphaproteobacteria	Rhodospirillales	Rhodospirillaceae		S001577943 Ferrovibrio denitrificans; Sp-1 Lineage=Root;rootrank;Bacteria;domain;"Proteobacteria";phylum;Alphaproteobacteria;class;unclassified_Alphaproteobacteria;	88.44	1.00E-123	444
570	0	0	0	2	0	2	1	0	0	Proteobacteria	Alphaproteobacteria	Rhodospirillales	Rhodospirillaceae		S002152203 Reyranella massiliensis; 3B26 Lineage=Root;rootrank;Bacteria;domain;"Proteobacteria";phylum;Alphaproteobacteria;class;unclassified_Alphaproteobacteria;	97.84	0	640
683	0	0	1	10	5	0	0	0	0	Proteobacteria	Alphaproteobacteria	Rhodospirillales	Rhodospirillaceae		S002909692 Candidatus Riegeria galatelae; C08086 Lineage=Root;rootrank;Bacteria;domain;"Proteobacteria";phylum;Alphaproteobacteria;class;unclassified_Alphaproteobacteria;	89.76	1.00E-132	473
2545	0	0	1	5	2	0	1	0	0	Proteobacteria	Alphaproteobacteria	Rhodospirillales	Rhodospirillaceae		S002909701 Candidatus Riegeria galatelae; C07001 Lineage=Root;rootrank;Bacteria;domain;"Proteobacteria";phylum;Alphaproteobacteria;class;unclassified_Alphaproteobacteria;	90	3.00E-134	479
3098	1	1	0	0	1	0	0	0	0	Proteobacteria	Alphaproteobacteria	Rhodospirillales	Rhodospirillaceae		S002909701 Candidatus Riegeria galatelae; C07001 Lineage=Root;rootrank;Bacteria;domain;"Proteobacteria";phylum;Alphaproteobacteria;class;unclassified_Alphaproteobacteria;	89.76	1.00E-132	473
178	17	26	17	5	17	9	15	17	15	Proteobacteria	Alphaproteobacteria	Rhodospirillales	Rhodospirillaceae		S002909704 Candidatus Riegeria galatelae; D09093 Lineage=Root;rootrank;Bacteria;domain;"Proteobacteria";phylum;Alphaproteobacteria;class;unclassified_Alphaproteobacteria;	89.28	4.00E-128	459
1833	0	0	0	1	1	0	0	5	6	Proteobacteria	Alphaproteobacteria	Rhodospirillales	Rhodospirillaceae		S002909704 Candidatus Riegeria galatelae; D09093 Lineage=Root;rootrank;Bacteria;domain;"Proteobacteria";phylum;Alphaproteobacteria;class;unclassified_Alphaproteobacteria;	89.22	4.00E-128	459
407	1	14	16	2	3	8	13	19	19	Proteobacteria	Alphaproteobacteria	Rhodospirillales	Rhodospirillaceae		S002909712 Candidatus Riegeria galatelae; D08001 Lineage=Root;rootrank;Bacteria;domain;"Proteobacteria";phylum;Alphaproteobacteria;class;unclassified_Alphaproteobacteria;	89.22	1.00E-128	460
199	0	0	3	19	9	9	5	5	12	Proteobacteria	Alphaproteobacteria	Rhodospirillales	Rhodospirillaceae		S002909712 Candidatus Riegeria galatelae; D08001 Lineage=Root;rootrank;Bacteria;domain;"Proteobacteria";phylum;Alphaproteobacteria;class;unclassified_Alphaproteobacteria;	89.49	6.00E-131	468
2989	0	0	0	6	5	5	6	8	4	Proteobacteria	Alphaproteobacteria	Rhodospirillales	Rhodospirillaceae		S002909712 Candidatus Riegeria galatelae; D08001 Lineage=Root;rootrank;Bacteria;domain;"Proteobacteria";phylum;Alphaproteobacteria;class;unclassified_Alphaproteobacteria;	89.01	4.00E-128	459

1245	0	0	0	7	6	0	0	1	0	Proteobacteria	Alphaproteobacteria	Rhodospirillales	Rhodospirillaceae		S002909712 Candidatus Riegeria galatelae; D08001 Lineage=Root;rootrank;Bacteria;domain;"Proteobacteria";phylum;Alphaproteobacteria;class;unclassified_Alphaproteobacteria;	88.68	7.00E-126	451
2381	0	0	0	4	1	0	0	0	0	Proteobacteria	Alphaproteobacteria	Rhodospirillales	Rhodospirillaceae		S002909712 Candidatus Riegeria galatelae; D08001 Lineage=Root;rootrank;Bacteria;domain;"Proteobacteria";phylum;Alphaproteobacteria;class;unclassified_Alphaproteobacteria;	89.22	1.00E-128	460
1112	0	0	0	0	0	0	0	0	1	Proteobacteria	Alphaproteobacteria	Rhodospirillales	Rhodospirillaceae		S002909712 Candidatus Riegeria galatelae; D08001 Lineage=Root;rootrank;Bacteria;domain;"Proteobacteria";phylum;Alphaproteobacteria;class;unclassified_Alphaproteobacteria;	91.6	1.00E-143	510
1014	0	0	0	0	0	1	0	0	0	Proteobacteria	Alphaproteobacteria	Rhodospirillales	Rhodospirillaceae		S002909713 Candidatus Riegeria galatelae; E10001 Lineage=Root;rootrank;Bacteria;domain;"Proteobacteria";phylum;Alphaproteobacteria;class;unclassified_Alphaproteobacteria;	87.6	1.00E-117	424
170	0	0	4	10	15	0	2	2	0	Proteobacteria	Alphaproteobacteria	Rhodospirillales	Rhodospirillaceae		S003260254 Thermovum composti; Nis3 Lineage=Root;rootrank;Bacteria;domain;"Proteobacteria";phylum;Alphaproteobacteria;class;Rhizobiales;order;Phyllobacteriaceae;family;unclassified_Phyllobacteriaceae;	88.95	9.00E-135	481
2736	0	0	0	2	1	0	0	0	0	Proteobacteria	Alphaproteobacteria	Rhodospirillales	Rhodospirillaceae		S003260254 Thermovum composti; Nis3 Lineage=Root;rootrank;Bacteria;domain;"Proteobacteria";phylum;Alphaproteobacteria;class;Rhizobiales;order;Phyllobacteriaceae;family;unclassified_Phyllobacteriaceae;	88.07	4.00E-128	459
1116	0	0	0	0	0	0	0	0	1	Proteobacteria	Alphaproteobacteria	Rhodospirillales	Rhodospirillaceae		S003260254 Thermovum composti; Nis3 Lineage=Root;rootrank;Bacteria;domain;"Proteobacteria";phylum;Alphaproteobacteria;class;Rhizobiales;order;Phyllobacteriaceae;family;unclassified_Phyllobacteriaceae;	88.65	8.00E-125	448
2779	0	0	0	1	0	0	0	3	12	Proteobacteria	Alphaproteobacteria	Rhodospirillales	Rhodospirillaceae		S003263888 Sneathiella glossodoripedis; NBRC 103391 Lineage=Root;rootrank;Bacteria;domain;"Proteobacteria";phylum;Alphaproteobacteria;class;Sneathiellales;order;Sneathiellaceae;family;Sneathiella;genus	97.04	1.00E-177	623
380	0	0	0	1	1	0	0	1	2	Proteobacteria	Alphaproteobacteria	Rhodospirillales	Rhodospirillaceae		S003263888 Sneathiella glossodoripedis; NBRC 103391 Lineage=Root;rootrank;Bacteria;domain;"Proteobacteria";phylum;Alphaproteobacteria;class;Sneathiellales;order;Sneathiellaceae;family;Sneathiella;genus	96.76	2.00E-175	616
2030	0	0	0	0	0	0	0	1	0	Proteobacteria	Alphaproteobacteria	Rhodospirillales	Rhodospirillaceae		S003263891 Sneathiella chinensis; NBRC 103408 Lineage=Root;rootrank;Bacteria;domain;"Proteobacteria";phylum;Alphaproteobacteria;class;Sneathiellales;order;Sneathiellaceae;family;Sneathiella;genus	90.54	1.00E-137	490
1669	2	36	4	4	1	4	4	3	2	Proteobacteria	Alphaproteobacteria	Rhodospirillales	Rhodospirillaceae		S003315125 Oceanicella actignis; PRQ-68 Lineage=Root;rootrank;Bacteria;domain;"Proteobacteria";phylum;Alphaproteobacteria;class;Rhodobacterales;order;Rhodobacteraceae;family;unclassified_Rhodobacteraceae;	90.62	1.00E-137	490
2842	0	0	6	14	11	6	9	6	11	Proteobacteria	Alphaproteobacteria	Rhodospirillales	Rhodospirillaceae		S003315125 Oceanicella actignis; PRQ-68 Lineage=Root;rootrank;Bacteria;domain;"Proteobacteria";phylum;Alphaproteobacteria;class;Rhodobacterales;order;Rhodobacteraceae;family;unclassified_Rhodobacteraceae;	89.4	8.00E-130	464
2879	0	0	0	0	2	0	0	1	0	Proteobacteria	Alphaproteobacteria	Rhodospirillales	Rhodospirillaceae		S003315125 Oceanicella actignis; PRQ-68 Lineage=Root;rootrank;Bacteria;domain;"Proteobacteria";phylum;Alphaproteobacteria;class;Rhodobacterales;order;Rhodobacteraceae;family;unclassified_Rhodobacteraceae;	90	3.00E-134	479
2351	0	0	0	0	0	0	1	0	0	Proteobacteria	Alphaproteobacteria	Rhodospirillales	Rhodospirillaceae		S003610740 Rhodothalassium salexigens; type strain: 260 Lineage=Root;rootrank;Bacteria;domain;"Proteobacteria";phylum;Alphaproteobacteria;class;Rhodobacterales;order;Rhodobacteraceae;family;Rhodothalassium;genus	88.68	6.00E-126	451
2845	10	4	291	457	700	1412	1164	960	1239	Proteobacteria	Alphaproteobacteria	Rhodospirillales	Rhodospirillaceae		S003710612 Geminicoccus roseus; SU27 Lineage=Root;rootrank;Bacteria;domain;"Proteobacteria";phylum;Alphaproteobacteria;class;Alphaproteobacteria_incertae_sedis;order;Geminicoccus;genus	90	1.00E-133	477
1093	111	459	312	212	289	439	418	137	146	Proteobacteria	Alphaproteobacteria	Rhodospirillales	Rhodospirillaceae		S003710612 Geminicoccus roseus; SU27 Lineage=Root;rootrank;Bacteria;domain;"Proteobacteria";phylum;Alphaproteobacteria;class;Alphaproteobacteria_incertae_sedis;order;Geminicoccus;genus	90.24	2.00E-135	483
2491	69	53	372	362	309	286	253	95	183	Proteobacteria	Alphaproteobacteria	Rhodospirillales	Rhodospirillaceae		S003710612 Geminicoccus roseus; SU27 Lineage=Root;rootrank;Bacteria;domain;"Proteobacteria";phylum;Alphaproteobacteria;class;Alphaproteobacteria_incertae_sedis;order;Geminicoccus;genus	91.11	2.00E-140	499
517	3	1	26	114	42	67	78	28	75	Proteobacteria	Alphaproteobacteria	Rhodospirillales	Rhodospirillaceae		S003710612 Geminicoccus roseus; SU27 Lineage=Root;rootrank;Bacteria;domain;"Proteobacteria";phylum;Alphaproteobacteria;class;Alphaproteobacteria_incertae_sedis;order;Geminicoccus;genus	88.65	2.00E-125	449
402	0	0	3	0	2	9	3	3	3	Proteobacteria	Alphaproteobacteria	Rhodospirillales	Rhodospirillaceae		S003710612 Geminicoccus roseus; SU27 Lineage=Root;rootrank;Bacteria;domain;"Proteobacteria";phylum;Alphaproteobacteria;class;Alphaproteobacteria_incertae_sedis;order;Geminicoccus;genus	90.03	1.00E-133	477
2803	2	1	4	2	2	0	0	3	2	Proteobacteria	Alphaproteobacteria	Rhodospirillales	Rhodospirillaceae		S003710612 Geminicoccus roseus; SU27 Lineage=Root;rootrank;Bacteria;domain;"Proteobacteria";phylum;Alphaproteobacteria;class;Alphaproteobacteria_incertae_sedis;order;Geminicoccus;genus	90.27	2.00E-135	483
2725	0	0	0	7	1	0	3	7	0	Proteobacteria	Alphaproteobacteria	Rhodospirillales	Rhodospirillaceae		S003710612 Geminicoccus roseus; SU27 Lineage=Root;rootrank;Bacteria;domain;"Proteobacteria";phylum;Alphaproteobacteria;class;Alphaproteobacteria_incertae_sedis;order;Geminicoccus;genus	88.95	1.00E-127	457
968	0	0	1	9	1	0	0	0	3	Proteobacteria	Alphaproteobacteria	Rhodospirillales	Rhodospirillaceae		S003710612 Geminicoccus roseus; SU27 Lineage=Root;rootrank;Bacteria;domain;"Proteobacteria";phylum;Alphaproteobacteria;class;Alphaproteobacteria_incertae_sedis;order;Geminicoccus;genus	87.84	8.00E-120	431
1359	0	0	0	1	2	2	0	3	4	Proteobacteria	Alphaproteobacteria	Rhodospirillales	Rhodospirillaceae		S003710612 Geminicoccus roseus; SU27 Lineage=Root;rootrank;Bacteria;domain;"Proteobacteria";phylum;Alphaproteobacteria;class;Alphaproteobacteria_incertae_sedis;order;Geminicoccus;genus	89.16	1.00E-128	460
1835	0	0	0	4	1	0	0	3	3	Proteobacteria	Alphaproteobacteria	Rhodospirillales	Rhodospirillaceae		S003710612 Geminicoccus roseus; SU27 Lineage=Root;rootrank;Bacteria;domain;"Proteobacteria";phylum;Alphaproteobacteria;class;Alphaproteobacteria_incertae_sedis;order;Geminicoccus;genus	88.38	4.00E-123	442
2510	0	2	0	0	0	2	0	0	0	Proteobacteria	Alphaproteobacteria	Rhodospirillales	Rhodospirillaceae		S003710612 Geminicoccus roseus; SU27 Lineage=Root;rootrank;Bacteria;domain;"Proteobacteria";phylum;Alphaproteobacteria;class;Alphaproteobacteria_incertae_sedis;order;Geminicoccus;genus	90.24	2.00E-135	483
757	0	0	12	79	75	2	6	19	12	Proteobacteria	Alphaproteobacteria	Rhodospirillales	Rhodospirillaceae		S003716514 Candidatus Nitrosoarchaeum limnia SFB1; bSFB1-77 Lineage=Root;rootrank;Bacteria;domain;"Proteobacteria";phylum;Alphaproteobacteria;class;unclassified_Alphaproteobacteria;	89.52	6.00E-131	468
1422	16	8	0	2	0	3	1	7	3	Proteobacteria	Alphaproteobacteria	Rhodospirillales	Rhodospirillaceae		S003716514 Candidatus Nitrosoarchaeum limnia SFB1; bSFB1-77 Lineage=Root;rootrank;Bacteria;domain;"Proteobacteria";phylum;Alphaproteobacteria;class;unclassified_Alphaproteobacteria;	88	2.00E-121	436
2586	0	1	0	5	1	6	10	3	0	Proteobacteria	Alphaproteobacteria	Rhodospirillales	Rhodospirillaceae		S003716514 Candidatus Nitrosoarchaeum limnia SFB1; bSFB1-77 Lineage=Root;rootrank;Bacteria;domain;"Proteobacteria";phylum;Alphaproteobacteria;class;unclassified_Alphaproteobacteria;	88.77	2.00E-126	453
285	0	0	0	7	2	0	0	0	0	Proteobacteria	Alphaproteobacteria	Rhodospirillales	Rhodospirillaceae		S003716514 Candidatus Nitrosoarchaeum limnia SFB1; bSFB1-77 Lineage=Root;rootrank;Bacteria;domain;"Proteobacteria";phylum;Alphaproteobacteria;class;unclassified_Alphaproteobacteria;	88.62	3.00E-124	446
3164	0	1	5	1	3	2	2	1	3	Proteobacteria	Alphaproteobacteria	Rhodospirillales	Rhodospirillaceae		S003716576 Candidatus Nitrosoarchaeum limnia BG20; bBG20-19 Lineage=Root;rootrank;Bacteria;domain;"Proteobacteria";phylum;Alphaproteobacteria;class;unclassified_Alphaproteobacteria;	90.05	1.00E-133	477
2849	0	1	0	0	3	0	1	1	4	Proteobacteria	Alphaproteobacteria	Rhodospirillales	Rhodospirillaceae		S003744139 Candidatus Liberibacter asiaticus Lineage=Root;rootrank;Bacteria;domain;"Proteobacteria";phylum;Alphaproteobacteria;class;unclassified_Alphaproteobacteria;	87.77	4.00E-118	425
1854	0	0	0	1	0	0	0	0	0	Proteobacteria	Alphaproteobacteria	Rhodospirillales	Rhodospirillaceae	Inquilinus	S000133119 Blastochloris sulfoviridis; RN1 Lineage=Root;rootrank;Bacteria;domain;"Proteobacteria";phylum;Alphaproteobacteria;class;unclassified_Alphaproteobacteria;	89.23	3.00E-135	483
778	0	0	0	1	2	1	0	1	0	Proteobacteria	Alphaproteobacteria	Rhodospirillales	Rhodospirillaceae	Inquilinus	S002033886 Tepidamorphus gemmatus (T); CB-27A Lineage=Root;rootrank;Bacteria;domain;"Proteobacteria";phylum;Alphaproteobacteria;class;Rhizobiales;order;Rhodobiaceae;family;Tepidamorphus;genus	88.8	4.00E-133	475
580	0	0	0	1	0	0	0	0	0	Proteobacteria	Alphaproteobacteria	Rhodospirillales	Rhodospirillaceae	Phaeospirillum	S001577943 Ferrovibrio denitrificans; Sp-1 Lineage=Root;rootrank;Bacteria;domain;"Proteobacteria";phylum;Alphaproteobacteria;class;unclassified_Alphaproteobacteria;	91.62	3.00E-144	512
2726	0	0	0	10	0	0	0	0	2	Proteobacteria	Alphaproteobacteria	Rhodospirillales	Rhodospirillaceae		S000019513 Olavius loisae endosymbiont 2 Lineage=Root;rootrank;Bacteria;domain;"Proteobacteria";phylum;Alphaproteobacteria;class;unclassified_Alphaproteobacteria;	88.43	2.00E-131	470
338	0	1	0	3	3	6	5	1	7	Proteobacteria	Alphaproteobacteria	Rhodospirillales	Rhodospirillaceae		S000022613 Olavius loisae endosymbiont 3 Lineage=Root;rootrank;Bacteria;domain;"Proteobacteria";phylum;Alphaproteobacteria;class;unclassified_Alphaproteobacteria;	90.05	2.00E-141	503
1567	0	0	1	0	0	2	0	0	0	Proteobacteria	Alphaproteobacteria	Rhodospirillales	Rhodospirillaceae		S000022613 Olavius loisae endosymbiont 3 Lineage=Root;rootrank;Bacteria;domain;"Proteobacteria";phylum;Alphaproteobacteria;class;unclassified_Alphaproteobacteria;	89.26	2.00E-136	486
1880	0	0	0	2	0	0	0	0	0	Proteobacteria	Alphaproteobacteria	Rhodospirillales	Rhodospirillaceae		S000022613 Olavius loisae endosymbiont 3 Lineage=Root;rootrank;Bacteria;domain;"Proteobacteria";phylum;Alphaproteobacteria;class;unclassified_Alphaproteobacteria;	89.2	9.00E-135	481
2098	0	0	0	2	0	0	0	0	0	Proteobacteria	Alphaproteobacteria	Rhodospirillales	Rhodospirillaceae		S000022613 Olavius loisae endosymbiont 3 Lineage=Root;rootrank;Bacteria;domain;"Proteobacteria";phylum;Alphaproteobacteria;class;unclassified_Alphaproteobacteria;	90.23	1.00E-142	507
2101	0	0	0	0	0	1	0	1	0	Proteobacteria	Alphaproteobacteria	Rhodospirillales	Rhodospirillaceae		S000022613 Olavius loisae endosymbiont 3 Lineage=Root;rootrank;Bacteria;domain;"Proteobacteria";phylum;Alphaproteobacteria;class;unclassified_Alphaproteobacteria;	87.98	2.00E-127	457
637	0	0	0	1	0	0	0	0	0	Proteobacteria	Alphaproteobacteria	Rhodospirillales	Rhodospirillaceae		S000022613 Olavius loisae endosymbiont 3 Lineage=Root;rootrank;Bacteria;domain;"Proteobacteria";phylum;Alphaproteobacteria;class;unclassified_Alphaproteobacteria;	88.78	4.00E-133	475
551	0	0	0	1	4	0	0	0	0	Proteobacteria	Alphaproteobacteria	Rhodospirillales	Rhodospirillaceae		S000133119 Blastochloris sulfoviridis; RN1 Lineage=Root;rootrank;Bacteria;domain;"Proteobacteria";phylum;Alphaproteobacteria;class;unclassified_Alphaproteobacteria;	90.03	3.00E-134	479
1462	4	46	9	2	1	6	12	3	10	Proteobacteria	Alphaproteobacteria	Rhodospirillales	Rhodospirillaceae		S000438700 Amaricoccus kaplicensis (T); Ben101 Lineage=Root;rootrank;Bacteria;domain;"Proteobacteria";phylum;Alphaproteobacteria;class;Rhodobacterales;order;Rhodobacteraceae;family;Amaricoccus;genus	89.6	6.00E-131	468
1437	0	0	3	16	16	2	4	13	9	Proteobacteria	Alphaproteobacteria	Rhodospirillales	Rhodospirillaceae		S000438701 Amaricoccus macauensis (T); Ben104 Lineage=Root;rootrank;Bacteria;domain;"Proteobacteria";phylum;Alphaproteobacteria;class;Rhodobacterales;order;Rhodobacteraceae;family;Amaricoccus;genus	88.8	4.00E-133	475
1380	0	0	0	0	0	0	1	0	0	Proteobacteria	Alphaproteobacteria	Rhodospirillales	Rhodospirillaceae		S000438701 Amaricoccus macauensis (T); Ben104 Lineage=Root;rootrank;Bacteria;domain;"Proteobacteria";phylum;Alphaproteobacteria;class;Rhodobacterales;order;Rhodobacteraceae;family;Amaricoccus;genus	90.62	5.00E-137	488
994	6	2	15	34	22	14	13	37	25	Proteobacteria	Alphaproteobacteria	Rhodospirillales	Rhodospirillaceae		S000460547 endosymbiont of Chlamys farreri Lineage=Root;rootrank;Bacteria;domain;"Proteobacteria";phylum;Alphaproteobacteria;class;Kiloniellales;order;Kiloniellaceae;family;Kiloniella;genus	90.24	8.00E-135	481
3036	6	4	9	3	0	3	2	2	3	Proteobacteria	Alphaproteobacteria	Rhodospirillales	Rhodospirillaceae		S000460547 endosymbiont of Chlamys farreri Lineage=Root;rootrank;Bacteria;domain;"Proteobacteria";phylum;Alphaproteobacteria;class;Kiloniellales;order;Kiloniellaceae;family;Kiloniella;genus	88.97	9.00E-135	481
3264	0	0	1	0	0	0	0	0	0	Proteobacteria	Alphaproteobacteria	Rhodospirillales	Rhodospirillaceae		S001094905 Elioraea tepidiphila (T); TU-7 Lineage=Root;rootrank;Bacteria;domain;"Proteobacteria";phylum;Alphaproteobacteria;class;Alphaproteobacteria_incertae_sedis;order;Elioraea;genus	89.73	6.00E-131	468
980	0	0	0	5	2	0	2	1	10	Proteobacteria	Alphaproteobacteria	Rhodospirillales	Rhodospirillaceae		S001550319 Amorphus orientalis (T); YIM D10 Lineage=Root;rootrank;Bacteria;domain;"Proteobacteria";phylum;Alphaproteobacteria;class;Rhizobiales;order;Rhizobiales_incertae_sedis;family;Amorphus;genus	89.19	4.00E-128	459
1869	0	0	0	1	4	0	0	1	0	Proteobacteria	Alphaproteobacteria	Rhodospirillales	Rhodospirillaceae		S001577943 Ferrovibrio denitrificans; Sp-1 Lineage=Root;rootrank;Bacteria;domain;"Proteobacteria";phylum;Alphaproteobacteria;class;unclassified_Alphaproteobacteria;	87.7	3.00E-119	429
345	0	0	0	0	1	0	0	0	0	Proteobacteria	Alphaproteobacteria	Rhodospirillales	Rhodospirillaceae		S001577943 Ferrovibrio denitrificans; Sp-1 Lineage=Root;rootrank;Bacteria;domain;"Proteobacteria";phylum;Alphaproteobacteria;class;unclassified_Alphaproteobacteria;	88.5	8.00E-125	448
2689	0	0	0	0	1	0	0	0	0	Proteobacteria	Alphaproteobacteria	Rhodospirillales	Rhodospirillaceae		S002304636 Dichotomicrobium thermohalophilum; type strain: DSM5002 Lineage=Root;rootrank;Bacteria;domain;"Proteobacteria";phylum;Alphaproteobacteria;class;Rhizobiales;order;unclassified_Rhizobiales;	90.93	1.00E-137	490
3242	0	0	0	1	0	0	0	0	0	Proteobacteria	Alphaproteobacteria	Rhodospirillales	Rhodospirillaceae		S002304636 Dichotomicrobium thermohalophilum; type strain: DSM5002 Lineage=Root;rootrank;Bacteria;domain;"Proteobacteria";phylum;Alphaproteobacteria;class;Rhizobiales;order;unclassified_Rhizobiales;	90	2.00E-140	499
2432	0	0	0	0	0	0	1	2	0	Proteobacteria	Alphaproteobacteria	Rhodospirillales	Rhodospirillaceae		S002909701 Candidatus Riegeria galatelae; C07001 Lineage=Root;rootrank;Bacteria;domain;"Proteobacteria";phylum;Alphaproteobacteria;class;unclassified_Alphaproteobacteria;	88.43	9.00E-130	464
2267	0	0	0	7	1	0	0	0	2	Proteobacteria	Alphaproteobacteria	Rhodospirillales	Rhodospirillaceae		S002909704 Candidatus Riegeria galatelae; D09093 Lineage=Root;rootrank;Bacteria;domain;"Proteobacteria";phylum;Alphaproteobacteria;class;unclassified_Alphaproteobacteria;	87.97	5.00E-122	438
1543	0	0	0	3	1	0	0	1	0	Proteobacteria	Alphaproteobacteria	Rhodospirillales	Rhodospirillaceae		S002909704 Candidatus Riegeria galatelae; D09093 Lineage=Root;rootrank;Bacteria;domain;"Proteobacteria";phylum;Alphaproteobacteria;class;unclassified_Alphaproteobacteria;	87.97	2.00E-121	436
365	0	0	0	1	0	0	0	0	0	Proteobacteria	Alphaproteobacteria	Rhodospirillales	Rhodospirillaceae		S002909712 Candidatus Riegeria galatelae; D08001 Lineage=Root;rootrank;Bacteria;domain;"Proteobacteria";phylum;Alphaproteobacteria;class;unclassified_Alphaproteobacteria;	88.01	1.00E-128	460
3201	0	0	0	0	0	0	1	0	0	Proteobacteria	Alphaproteobacteria	Rhodospirillales	Rhodospirillaceae		S002909712 Candidatus Riegeria galatelae; D08001 Lineage=Root;rootrank;Bacteria;domain;"Proteobacteria";phylum;Alphaproteobacteria;class;unclassified_Alphaproteobacteria;	89.52	6.00E-131	468
2768	0	5	15	4	7	5	5	13	1	Proteobacteria	Alphaproteobacteria	Rhodospirillales	Rhodospirillaceae		S002909713 Candidatus Riegeria galatelae; E10001 Lineage=Root;rootrank;Bacteria;domain;"Proteobacteria";phylum;Alphaproteobacteria;class;unclassified_Alphaproteobacteria;	88.35	1.00E-122	440
99	4	203	3	5	6	14	1	16	10	Proteobacteria	Alphaproteobacteria	Rhodospirillales	Rhodospirillaceae		S003710612 Geminicoccus roseus; SU27 Lineage=Root;rootrank;Bacteria;domain;"Proteobacteria";phylum;Alphaproteobacteria;class;Alphaproteobacteria_incertae_sedis;order;Geminicoccus;genus	89.43	8.00E-130	464
1832	0	2	8	1	0	0	0	2	3	Proteobacteria	Alphaproteobacteria	Rhodospirillales	Rhodospirillaceae		S003710612 Geminicoccus roseus; SU27 Lineage=Root;rootrank;Bacteria;domain;"Proteobacteria";phylum;Alphaproteobacteria;class;Alphaproteobacteria_incertae_sedis;order;Geminicoccus;genus	89.16	4.00E-128	459
130	0	0	0	0	0	0	0	0	1	Proteobacteria	Alphaproteobacteria	Rhodospirillales	Rhodospirillaceae		S003710612 Geminicoccus roseus; SU27 Lineage=Root;rootrank;Bacteria;domain;"Proteobacteria";phylum;Alphaproteobacteria;class;Alphaproteobacteria_incertae_sedis;order;Geminicoccus;genus	89.04	1.00E-127	457
2329	1	1	25	143	122	38	40	80	42	Proteobacteria	Alphaproteobacteria	Rhodospirillales	Rhodospirillaceae		S003716514 Candidatus Nitrosoarchaeum limnia SFB1; bSFB1-77 Lineage=Root;rootrank;Bacteria;domain;"Proteobacteria";phylum;Alphaproteobacteria;class;unclassified_Alphaproteobacteria;	91.13	6.00E-141	501
839	0	0	0	3	0	0	0	0	0	Proteobacteria	Alphaproteobacteria	Rhodospirillales			S000460547 endosymbiont of Chlamys farreri Lineage=Root;rootrank;Bacteria;domain;"Proteobacteria";phylum;Alphaproteobacteria;class;Kiloniellales;order;Kiloniellaceae;family;Kiloniella;genus	88.44	7.00E-131	468
294	0	0	0	1	0	0	0	0	0	Proteobacteria	Alphaproteobacteria	Rhodospirillales			S002164967 Aciditerrimonas ferrireducens (T); IC-180 Lineage=Root;rootrank;Bacteria;domain;"Actinobacteria";phylum;Actinobacteria;class;Acidimicrobidae;subclass;Acidimicrobiales;order;"Acidimicrobineae";suborder;"Acidimicrobineae"_incertae_sedis;family;Aciditerrimonas;genus	85.87	1.00E-108	394
665	0	0	0	0	1	0	0	0	0	Proteobacteria	Alphaproteobacteria	Rhodospirillales			S003710612 Geminicoccus roseus; SU27 Lineage=Root;rootrank;Bacteria;domain;"Proteobacteria";phylum;Alphaproteobacteria;class;Alphaproteobacteria_incertae_sedis;order;Geminicoccus;genus	87.06	2.00E-115	416
500	0	2	21	4	25	9	26	18	5	Proteobacteria	Alphaproteobacteria	Rhodospirillales			S003716514 Candidatus Nitrosoarchaeum limnia SFB1; bSFB1-77 Lineage=Root;rootrank;Bacteria;domain;"Proteobacteria";phylum;Alphaproteobacteria;class;unclassified_Alphaproteobacteria;	88.69	2.00E-131	470
2478	0	0	1	0	0	0	0	0	0	Proteobacteria	Alphaproteobacteria	Rhodospirillales			S003716514 Candidatus Nitrosoarchaeum limnia SFB1; bSFB1-77 Lineage=Root;rootrank;Bacteria;domain;"Proteobacteria";phylum;Alphaproteobacteria;class;unclassified_Alphaproteobacteria;	87.02	3.00E-120	433
2877	0	0	0	0	1	0	0	0	0	Proteobacteria	Alphaproteobacteria	Rickettsiales		CandidatusOdyssella	S000387797 endosymbiont of Acanthamoeba polyphaga Lineage=Root;rootrank;Bacteria;domain;"Proteobacteria";phylum;Gammaproteobacteria;class;Thiotrichales;order;Thiotrichales_incertae_sedis;family;Caedibacter;genus	88.44	1.00E-123	444
618	0	0	0	0	0	0	0	1	4	Proteobacteria	Alphaproteobacteria	Rickettsiales			S000387797 endosymbiont of Acanthamoeba polyphaga Lineage=Root;rootrank;Bacteria;domain;"Proteobacteria";phylum;Gammaproteobacteria;class;Thiotrichales;order;Thiotrichales_incertae_sedis;family;Caedibacter;genus	91.51	6.00E-141	501
833	0	2	0	0	0	1	0	0	0	Proteobacteria	Alphaproteobacteria	Rickettsiales			S000387797 endosymbiont of Acanthamoeba polyphaga Lineage=Root;rootrank;Bacteria;domain;"Proteobacteria";phylum;Gammaproteobacteria;class;Thiotrichales;order;Thiotrichales_incertae_sedis;family;Caedibacter;genus	92.1	2.00E-145	516
2012	0	0	0	0	1	0	0	1	0	Proteobacteria	Alphaproteobacteria	Rickettsiales			S000768480 Caedibacter macronucleorum; cm_31 Lineage=Root;rootrank;Bacteria;domain;"Proteobacteria";phylum;Gammaproteobacteria;class;Thiotrichales;order;Thiotrichales_incertae_sedis;family;Caedibacter;genus	88.89	2.00E-125	449
412	0	0	0	0	0	0	0	0	1	Proteobacteria	Alphaproteobacteria	Rickettsiales			S000387019 Klebsiella granulomatis Lineage=Root;rootrank;Bacteria;domain;"Proteobacteria";phylum;Gammaproteobacteria;class;"Enterobacteriales";order;Enterobacteriaceae;family;Klebsiella;genus	91.04	6.00E-17	89.8
1712	0	0	0	1	0	0	0	1	1	Proteobacteria	Alphaproteobacteria	Rickettsiales			S000498991 Malawimonas jakobiformis; ATCC 50310 Lineage=Root;rootrank;Bacteria;domain;"Proteobacteria";phylum;unclassified_"Proteobacteria";	86.56	1.00E-112	407
493	0	0	1	0	0	0	0	0	0	Proteobacteria	Alphaproteobacteria	Rickettsiales			S000539475 Methylocaldum szegediense; KTM-1 Lineage=Root;rootrank;Bacteria;domain;"Proteobacteria";phylum;Gammaproteobacteria;class;Methylococcales;order;Methylococcaceae;family;Methylocaldum;genus	84.99	1.00E-102	374
3110	0	0	0	0	2	0	0	0	0	Proteobacteria	Alphaproteobacteria	Rickettsiales			S000824445 Wolbachia endosymbiont of Nasutitermes nigriceps Lineage=Root;rootrank;Bacteria;domain;"Proteobacteria";phylum;Alphaproteobacteria;class;Rickettsiales;order;Anaplasmataceae;family;unclassified_Anaplasmataceae;	80.11	3.00E-69	263
1810	0	0	1	0	2	0	0	0	1	Proteobacteria	Alphaproteobacteria	Rickettsiales			S001239142 Pediococcus argentinicus (T) Lineage=Root;rootrank;Bacteria;domain;Firmicutes;phylum;Bacilli;class;Lactobacillales;order;Lactobacillaceae;family;Pediococcus;genus	77.44	9.00E-35	148
3034	1	0	0	0	0	0	0	0	0	Proteobacteria	Alphaproteobacteria	Rickettsiales			S002225583 Lactobacillus rhamnosus; E3 Lineage=Root;rootrank;Bacteria;domain;Firmicutes;phylum;Bacilli;class;Lactobacillales;order;Lactobacillaceae;family;Lactobacillus;genus	79.33	3.00E-20	100
40	0	1	0	0	0	0	0	0	0	Proteobacteria	Alphaproteobacteria	Rickettsiales			S002441910 Candidatus Thiopilula aggregata; COS016 Lineage=Root;rootrank;Bacteria;domain;"Proteobacteria";phylum;Gammaproteobacteria;class;unclassified_Gammaproteobacteria;	86.55	3.00E-119	429
2666	0	0	0	0	0	2	1	0	0	Proteobacteria	Alphaproteobacteria	Rickettsiales			S003722169 Bacillus cereus; BD18C2-R20 Lineage=Root;rootrank;Bacteria;domain;Firmicutes;phylum;Bacilli;class;Bacillales;order;Bacillaceae 1;family;Bacillus;genus	86.67	3.00E-15	84.2
1232	2	0	8	36	24	2	3	17	50	Proteobacteria	Alphaproteobacteria	Sphingomonadales			S000394590 Erythromicrobium ramosum (T); DSM 8510 Lineage=Root;rootrank;Bacteria;domain;"Proteobacteria";phylum;Alphaproteobacteria;class;Sphingomonadales;order;Erythrobacteraceae;family;unclassified_Erythrobacteraceae;	99.46	0	675
257	0	0	0	1	1	0	0	1	20	Proteobacteria	Alphaproteobacteria	Sphingomonadales	Sphingomonadaceae		S003610727 Sphingopyxis litoris (T); FR1093 Lineage=Root;rootrank;Bacteria;domain;"Proteobacteria";phylum;Alphaproteobacteria;class;Sphingomonadales;order;Sphingomonadaceae;family;Sphingopyxis;genus	99.46	0	675
2620	0	0	0	1	0	0	0	0	0	Proteobacteria	Alphaproteobacteria	Sphingomonadales	Sphingomonadaceae	Kaistobacter	S000701146 Kaistobacter terrae; KCTC12630 Lineage=Root;rootrank;Bacteria;domain;"Proteobacteria";phylum;Alphaproteobacteria;class;Sphingomonadales;order;Sphingomonadaceae;family;Sphingomonas;genus	98.92	0	664
369	0	0	0	1	0	0	0	0	0	Proteobacteria	Alphaproteobacteria	Sphingomonadales	Sphingomonadaceae	Sphingobium	S003752104 Sphingobium yanoikuyae; Cl-21 Lineage=Root;rootrank;Bacteria;domain;"Proteobacteria";phylum;Alphaproteobacteria;class;Sphingomonadales;order;Sphingomonadaceae;family;Sphingobium;genus	97.69	0	671
3013	0	0	0	0	0	0	0	0	1	Proteobacteria	Alphaproteobacteria	Sphingomonadales	Sphingomonadaceae	Sphingomonas	S002447667 Sphingomonas dokdonensis; T2CP8 Lineage=Root;rootrank;Bacteria;domain;"Proteobacteria";phylum;Alphaproteobacteria;class;Sphingomonadales;order;Sphingomonadaceae;family;Sphingomonas;genus	96.75	2.00E-175	616
2675	0	0	2	11	23	0	0	1	5	Proteobacteria	Alphaproteobacteria	Sphingomonadales	Sphingomonadaceae	Sphingopyxis	S003620307 Sphingopyxis ginsengisoli; BSN51 Lineage=Root;rootrank;Bacteria;domain;"Proteobacteria";phylum;Alphaproteobacteria;class;Sphingomonadales;order;Sphingomonadaceae;family;Sphingopyxis;genus	99.23	0	704
3032	0	0	1	0	0	0	0	0	0	Proteobacteria	Alphaproteobacteria	Sphingomonadales	Sphingomonadaceae		S000558533 Sphingosinicella soli (T); KSL-125 Lineage=Root;rootrank;Bacteria;domain;"Proteobacteria";phylum;Alphaproteobacteria;class;Sphingomonadales;order;Sphingomonadaceae;family;Sphingosinicella;genus	97.57	0	636
2852	0	0	0	4	2	0	0	0	0	Proteobacteria	Alphaproteobacteria	Sphingomonadales	Sphingomonadaceae		S003613241 Sphingomonas sanxanigenens; ERG5 Lineage=Root;rootrank;Bacteria;domain;"Proteobacteria";phylum;Alphaproteobacteria;class;Sphingomonadales;order;Sphingomonadaceae;family;Sphingomonas;genus	96.77	2.00E-176	619
2387	0	0	6	51	48	0	0	0	4	Proteobacteria	Alphaproteobacteria	Sphingomonadales	Sphingomonadaceae		S003721163 Sphingobium fuliginis; HC3 Lineage=Root;rootrank;Bacteria;domain;"Proteobacteria";phylum;Alphaproteobacteria;class;Sphingomonadales;order;Sphingomonadaceae;family;Sphingobium;genus	95.97	2.00E-171	603
2331	0	0	0	0	1	0	0	5	10	Proteobacteria	Alphaproteobacteria	Sphingomonadales			S000128764 Sphingobium xenophagum (T); BN6 Lineage=Root;rootrank;Bacteria;domain;"Proteobacteria";phylum;Alphaproteobacteria;class;Sphingomonadales;order;Sphingomonadaceae;family;Sphingobium;genus	96.51	8.00E-175	614
891	0	0	6	33	22	1	1	1	2	Proteobacteria	Alphaproteobacteria	Sphingomonadales			S001577009 Altererythrobacter epoxidivorans; THWCSN31 Lineage=Root;rootrank;Bacteria;domain;"Proteobacteria";phylum;Alphaproteobacteria;class;Sphingomonadales;order;Erythrobacteraceae;family;Altererythrobacter;genus	98.11	0	647
515	0	0	0	0	0	1	1	0	2	Proteobacteria	Alphaproteobacteria				S000019513 Olavius loisae endosymbiont 2 Lineage=Root;rootrank;Bacteria;domain;"Proteobacteria";phylum;Alphaproteobacteria;class;unclassified_Alphaproteobacteria;	89.2	2.00E-135	483
2539	0	0	0	0	0	0	0	0	1	Proteobacteria	Alphaproteobacteria				S000022613 Olavius loisae endosymbiont 3 Lineage=Root;rootrank;Bacteria;domain;"Proteobacteria";phylum;Alphaproteobacteria;class;unclassified_Alphaproteobacteria;	88.28	7.00E-121	435
2047	0	0	0	0	1	0	0	2	1	Proteobacteria	Alphaproteobacteria				S000387797 endosymbiont of Acanthamoeba polyphaga Lineage=Root;rootrank;Bacteria;domain;"Proteobacteria";phylum;Gammaproteobacteria;class;Thiotrichales;order;Thiotrichales_incertae_sedis;family;Caedibacter;genus	90	1.00E-133	477
2903	0	0	0	1	0	0	0	0	0	Proteobacteria	Alphaproteobacteria				S000396204 Blastochloris sulfoviridis; Wai3G1e Lineage=Root;rootrank;Bacteria;domain;"Proteobacteria";phylum;Alphaproteobacteria;class;Rhizobiales;order;Hyphomicrobiaceae;family;Blastochloris;genus	88.95	2.00E-126	453
382	46	225	40	19	24	41	89	67	58	Proteobacteria	Alphaproteobacteria				S000438701 Amaricoccus macauensis (T); Ben104 Lineage=Root;rootrank;Bacteria;domain;"Proteobacteria";phylum;Alphaproteobacteria;class;Rhodobacterales;order;Rhodobacteraceae;family;Amaricoccus;genus	90.59	5.00E-137	488
1404	0	0	0	1	2	0	0	143	72	Proteobacteria	Alphaproteobacteria				S000702049 Wenxinia marina (T); HY34 Lineage=Root;rootrank;Bacteria;domain;"Proteobacteria";phylum;Alphaproteobacteria;class;Rhodobacterales;order;Rhodobacteraceae;family;Wenxinia;genus	91.62	1.00E-143	510
406	1	0	0	0	0	0	0	0	0	Proteobacteria	Alphaproteobacteria				S000858544 Rhodobium orientis; JA208 Lineage=Root;rootrank;Bacteria;domain;"Proteobacteria";phylum;Alphaproteobacteria;class;Rhizobiales;order;Rhodobiaceae;family;Rhodobium;genus	90.11	2.00E-131	470
974	0	1	0	0	0	0	0	0	0	Proteobacteria	Alphaproteobacteria				S000902653 Antarcticicola litoralis; IMCC3295 Lineage=Root;rootrank;Bacteria;domain;"Proteobacteria";phylum;Alphaproteobacteria;class;Rhodobacterales;order;Rhodobacteraceae;family;unclassified_Rhodobacteraceae;	89.57	1.00E-138	494
2787	0	0	0	1	0	0	0	0	0	Proteobacteria	Alphaproteobacteria				S000902653 Antarcticicola litoralis; IMCC3295 Lineage=Root;rootrank;Bacteria;domain;"Proteobacteria";phylum;Alphaproteobacteria;class;Rhodobacterales;order;Rhodobacteraceae;family;unclassified_Rhodobacteraceae;	86.89	3.00E-120	433
2040	0	0	0	0	0	0	0	0	1	Proteobacteria	Alphaproteobacteria				S000925329 Rhizomicrobium palustre (T); A48 Lineage=Root;rootrank;Bacteria;domain;"Proteobacteria";phylum;Alphaproteobacteria;class;Alphaproteobacteria_incertae_sedis;order;Rhizomicrobium;genus	89.67	6.00E-131	468
3187	0	0	0	0	0	0	1	0	0	Proteobacteria	Alphaproteobacteria				S001152972 Sinorhizobium chiapanecum; ITTG S70 Lineage=Root;rootrank;Bacteria;domain;"Proteobacteria";phylum;Alphaproteobacteria;class;Rhizobiales;order;Rhizobiaceae;family;Ensifer;genus	91.24	4.00E-148	525
2647	0	1	3	0	0	0	0	0	0	Proteobacteria	Alphaproteobacteria				S001331897 Rhodothalassium salexigens; type strain: DSM 2132 Lineage=Root;rootrank;Bacteria;domain;"Proteobacteria";phylum;Alphaproteobacteria;class;Rhodobacterales;order;Rhodobacteraceae;family;Rhodothalassium;genus	91.35	2.00E-150	532
150	0	0	0	1	0	0	0	1	0	Proteobacteria	Alphaproteobacteria				S001331897 Rhodothalassium salexigens; type strain: DSM 2132 Lineage=Root;rootrank;Bacteria;domain;"Proteobacteria";phylum;Alphaproteobacteria;class;Rhodobacterales;order;Rhodobacteraceae;family;Rhodothalassium;genus	89.77	2.00E-140	499
1221	0	0	0	0	0	0	1	0	1	Proteobacteria	Alphaproteobacteria				S001331897 Rhodothalassium salexigens; type strain: DSM 2132 Lineage=Root;rootrank;Bacteria;domain;"Proteobacteria";phylum;Alphaproteobacteria;class;Rhodobacterales;order;Rhodobacteraceae;family;Rhodothalassium;genus	91.56	5.00E-152	538
21	0	0	0	2	1	0	0	0	0	Proteobacteria	Alphaproteobacteria				S001550319 Amorphus orientalis (T); YIM D10 Lineage=Root;rootrank;Bacteria;domain;"Proteobacteria";phylum;Alphaproteobacteria;class;Rhizobiales;order;Rhizobiales_incertae_sedis;family;Amorphus;genus	88.75	5.00E-132	472
1436	0	0	0	2	1	0	1	0	1	Proteobacteria	Alphaproteobacteria				S001574078 Kordiimonas gwangyangensis; S20-13 Lineage=Root;rootrank;Bacteria;domain;"Proteobacteria";phylum;Alphaproteobacteria;class;Kordiimonadales;order;"Kordiimonadaceae";family;Kordiimonas;genus	89.19	4.00E-128	459
2956	0	0	0	1	0	0	0	0	0	Proteobacteria	Alphaproteobacteria				S001575982 Prosthecomicrobium pneumaticum; 3a Lineage=Root;rootrank;Bacteria;domain;"Proteobacteria";phylum;Alphaproteobacteria;class;Rhizobiales;order;Hyphomicrobiaceae;family;Prosthecomicrobium;genus	87.69	7.00E-126	451
2867	0	0	0	1	0	0	0	0	0	Proteobacteria	Alphaproteobacteria				S002156318 Rhodovulum sulfidophilum; P721(3) Lineage=Root;rootrank;Bacteria;domain;"Proteobacteria";phylum;Alphaproteobacteria;class;Rhodobacterales;order;Rhodobacteraceae;family;unclassified_Rhodobacteraceae;	89.95	4.00E-133	475
2759	0	1	0	0	0	0	0	1	0	Proteobacteria	Alphaproteobacteria				S002287949 Parvibaculum lavamentivorans DS-1 Lineage=Root;rootrank;Bacteria;domain;"Proteobacteria";phylum;Alphaproteobacteria;class;Rhizobiales;order;Rhodobiaceae;family;Parvibaculum;genus	88.72	2.00E-132	473
1226	0	0	0	11	8	1	0	4	4	Proteobacteria	Alphaproteobacteria				S002909842 Albidovulum xiamenense; YBY-7 Lineage=Root;rootrank;Bacteria;domain;"Proteobacteria";phylum;Alphaproteobacteria;class;Rhodobacterales;order;Rhodobacteraceae;family;unclassified_Rhodobacteraceae;	92.05	9.00E-155	547
2514	0	0	0	1	0	0	0	0	0	Proteobacteria	Alphaproteobacteria				S002914216 Rhizobium leguminosarum; 430 Lineage=Root;rootrank;Bacteria;domain;"Proteobacteria";phylum;Alphaproteobacteria;class;Rhizobiales;order;Rhizobiaceae;family;Rhizobium;genus	89.51	4.00E-138	492
2531	2	0	0	0	0	4	1	0	0	Proteobacteria	Alphaproteobacteria				S003263714 Maricaulis maris; NBRC 102484 Lineage=Root;rootrank;Bacteria;domain;"Proteobacteria";phylum;Alphaproteobacteria;class;Caulobacterales;order;Hyphomonadaceae;family;Maricaulis;genus	91.28	9.00E-150	531
2887	1	0	0	0	0	0	0	0	0	Proteobacteria	Alphaproteobacteria				S003263714 Maricaulis maris; NBRC 102484 Lineage=Root;rootrank;Bacteria;domain;"Proteobacteria";phylum;Alphaproteobacteria;class;Caulobacterales;order;Hyphomonadaceae;family;Maricaulis;genus	91.79	4.00E-153	542
731	0	0	0	4	4	0	0	0	2	Proteobacteria	Alphaproteobacteria				S003263888 Sneathiella glossodoripedis; NBRC 103391 Lineage=Root;rootrank;Bacteria;domain;"Proteobacteria";phylum;Alphaproteobacteria;class;Sneathiellales;order;Sneathiellaceae;family;Sneathiella;genus	91.33	2.00E-141	503
405	17	3	85	15	36	60	59	4	9	Proteobacteria	Alphaproteobacteria				S003315125 Oceanicella actignis; PRQ-68 Lineage=Root;rootrank;Bacteria;domain;"Proteobacteria";phylum;Alphaproteobacteria;class;Rhodobacterales;order;Rhodobacteraceae;family;unclassified_Rhodobacteraceae;	91.58	5.00E-142	505
752	0	0	4	3	15	1	3	3	8	Proteobacteria	Alphaproteobacteria				S003315125 Oceanicella actignis; PRQ-68 Lineage=Root;rootrank;Bacteria;domain;"Proteobacteria";phylum;Alphaproteobacteria;class;Rhodobacterales;order;Rhodobacteraceae;family;unclassified_Rhodobacteraceae;	91.3	3.00E-149	529
1793	0	0	0	0	0	3	0	0	0	Proteobacteria	Alphaproteobacteria				S003315125 Oceanicella actignis; PRQ-68 Lineage=Root;rootrank;Bacteria;domain;"Proteobacteria";phylum;Alphaproteobacteria;class;Rhodobacterales;order;Rhodobacteraceae;family;unclassified_Rhodobacteraceae;	91.87	8.00E-145	514
1012	0	0	2	0	4	0	0	0	0	Proteobacteria	Alphaproteobacteria				S003710675 Micavibrio aeruginosavorus EPB Lineage=Root;rootrank;Bacteria;domain;"Proteobacteria";phylum;Alphaproteobacteria;class;unclassified_Alphaproteobacteria;	89.52	6.00E-131	468
3219	0	0	0	0	1	0	0	0	0	Proteobacteria	Alphaproteobacteria				S003710675 Micavibrio aeruginosavorus EPB Lineage=Root;rootrank;Bacteria;domain;"Proteobacteria";phylum;Alphaproteobacteria;class;unclassified_Alphaproteobacteria;	86.62	7.00E-121	435
375	0	0	0	1	0	0	0	0	0	Proteobacteria	Alphaproteobacteria				S003710675 Micavibrio aeruginosavorus EPB Lineage=Root;rootrank;Bacteria;domain;"Proteobacteria";phylum;Alphaproteobacteria;class;unclassified_Alphaproteobacteria;	87.5	3.00E-125	449
41	0	0	0	0	0	0	1	0	0	Proteobacteria	Alphaproteobacteria				S003718970 Hyphomonas johnsonii; SSW131 Lineage=Root;rootrank;Bacteria;domain;"Proteobacteria";phylum;Alphaproteobacteria;class;Caulobacterales;order;Hyphomonadaceae;family;Hyphomonas;genus	88.98	1.00E-127	457
1076	0	0	0	0	0	0	0	0	1	Proteobacteria	Alphaproteobacteria				S003745528 Sinorhizobium fredii; WW9 Lineage=Root;rootrank;Bacteria;domain;"Proteobacteria";phylum;Alphaproteobacteria;class;Rhizobiales;order;Rhizobiaceae;family;Ensifer;genus	86.92	1.00E-113	411
1396	0	0	0	18	3	0	0	0	0	Proteobacteria	Betaproteobacteria				S000711212 Tepidimonas thermarum; AA2 Lineage=Root;rootrank;Bacteria;domain;"Proteobacteria";phylum;Betaproteobacteria;class;Burkholderiales;order;Burkholderiales_incertae_sedis;family;Tepidimonas;genus	91.89	8.00E-145	514
3181	0	0	0	3	0	0	0	0	0	Proteobacteria	Betaproteobacteria				S002956166 Gulbenkiania mobilis; IARI-MB-18 Lineage=Root;rootrank;Bacteria;domain;"Proteobacteria";phylum;Betaproteobacteria;class;Neisseriales;order;Neisseriaceae;family;Gulbenkiania;genus	90.79	2.00E-146	520

2428	0	0	0	0	1	0	0	0	0	Proteobacteria	Betaproteobacteria				S003286830 Chitinimonas taiwanensis; OK-1 Lineage=Root;rootrank;Bacteria;domain;"Proteobacteria";phylum;Betaproteobacteria;class;Burkholderiales;order;Burkholderiaceae;family;Chitinimonas;genus	90.86	3.00E-139	496
804	0	0	0	7	4	0	0	0	0	Proteobacteria	Betaproteobacteria	Burkholderiales		Methylibium	S002290658 Lineage=Root;rootrank;Bacteria;domain;"Proteobacteria";phylum;Betaproteobacteria;class;Burkholderiales;order;Burkholderiales_incertae_sedis;family;Methylibium;genus	99.73	0	678
3233	0	0	0	0	0	0	1	0	0	Proteobacteria	Betaproteobacteria	Burkholderiales			S003284586 Pelomonas puraquae; S3-119 Lineage=Root;rootrank;Bacteria;domain;"Proteobacteria";phylum;Betaproteobacteria;class;Burkholderiales;order;Comamonadaceae;family;Pelomonas;genus	99.49	0	708
3265	0	0	1	0	0	0	0	0	0	Proteobacteria	Betaproteobacteria	Burkholderiales	Alcaligenaceae		S000516377 Denitromonas indolicum; MPKc Lineage=Root;rootrank;Bacteria;domain;"Proteobacteria";phylum;Betaproteobacteria;class;Rhodocyclales;order;Rhodocyclaceae;family;Azoarcus;genus	93.6	2.00E-156	553
718	2	0	0	25	6	1	0	0	0	Proteobacteria	Betaproteobacteria	Burkholderiales	Alcaligenaceae		S000824403 Pusillimonas noertemannii; T7-7 Lineage=Root;rootrank;Bacteria;domain;"Proteobacteria";phylum;Betaproteobacteria;class;Burkholderiales;order;Alcaligenaceae;family;Pusillimonas;genus	99.46	0	673
2004	0	0	0	1	0	0	0	1	3	Proteobacteria	Betaproteobacteria	Burkholderiales	Alcaligenaceae	Alcaligenes	S000541021 Pusillimonas noertemannii (T); BN9 Lineage=Root;rootrank;Bacteria;domain;"Proteobacteria";phylum;Betaproteobacteria;class;Burkholderiales;order;Alcaligenaceae;family;Pusillimonas;genus	96.49	1.00E-173	610
1287	0	0	0	0	1	0	0	0	0	Proteobacteria	Betaproteobacteria	Burkholderiales	Alcaligenaceae	Alcaligenes	S003755643 Alcaligenes faecalis; AP18 Lineage=Root;rootrank;Bacteria;domain;"Proteobacteria";phylum;Betaproteobacteria;class;Burkholderiales;order;Alcaligenaceae;family;Alcaligenes;genus	98.98	0	702
1325	0	0	0	1	0	0	0	0	0	Proteobacteria	Betaproteobacteria	Burkholderiales	Alcaligenaceae	Azohydromonas	S002411533 Aquincola tertiaricarbonis; C93 Lineage=Root;rootrank;Bacteria;domain;"Proteobacteria";phylum;Betaproteobacteria;class;Burkholderiales;order;Burkholderiales_incertae_sedis;family;unclassified_Burkholderiales_incertae_sedis;	97.18	0	658
464	0	0	0	0	1	0	0	0	0	Proteobacteria	Betaproteobacteria	Burkholderiales	Burkholderiaceae		S003722571 Lautropia mirabilis; SSI AB 2188 Lineage=Root;rootrank;Bacteria;domain;"Proteobacteria";phylum;Betaproteobacteria;class;Burkholderiales;order;unclassified_Burkholderiales;	97.57	0	634
591	0	0	0	0	0	0	0	0	1	Proteobacteria	Betaproteobacteria	Burkholderiales	Comamonadaceae	Polaromonas	S000627891 Polaromonas ginsengisoli; Gsoil 115 Lineage=Root;rootrank;Bacteria;domain;"Proteobacteria";phylum;Betaproteobacteria;class;Burkholderiales;order;Comamonadaceae;family;Polaromonas;genus	99.23	0	701
1665	0	0	0	3	0	0	0	1	0	Proteobacteria	Betaproteobacteria	Burkholderiales	Comamonadaceae		S000278331 Aquamonas fontana; AQ11 Lineage=Root;rootrank;Bacteria;domain;"Proteobacteria";phylum;Betaproteobacteria;class;Burkholderiales;order;Comamonadaceae;family;Curvibacter;genus	99.49	0	708
1143	0	0	0	0	1	0	0	0	0	Proteobacteria	Betaproteobacteria	Burkholderiales	Comamonadaceae		S003659868 Xylophilus ampelinus; RR3-1 Lineage=Root;rootrank;Bacteria;domain;"Proteobacteria";phylum;Betaproteobacteria;class;Burkholderiales;order;Burkholderiales_incertae_sedis;family;Xylophilus;genus	97.5	0	676
965	0	0	0	0	0	1	0	1	0	Proteobacteria	Betaproteobacteria	Burkholderiales	Oxalobacteraceae		S001572700 Herminiimonas fonticola; da Costa S-94 (=CIP 108398, =LMG 22527) Lineage=Root;rootrank;Bacteria;domain;"Proteobacteria";phylum;Betaproteobacteria;class;Burkholderiales;order;Oxalobacteraceae;family;Herminiimonas;genus	97.43	0	664
3112	0	0	0	1	1	0	0	0	0	Proteobacteria	Betaproteobacteria	Burkholderiales	Oxalobacteraceae		S003616548 Collimonas pratensis; BJC16-B7 Lineage=Root;rootrank;Bacteria;domain;"Proteobacteria";phylum;Betaproteobacteria;class;Burkholderiales;order;Oxalobacteraceae;family;Collimonas;genus	99.19	0	667
1097	0	0	0	0	1	0	0	0	0	Proteobacteria	Betaproteobacteria	Burkholderiales			S000627891 Polaromonas ginsengisoli; Gsoil 115 Lineage=Root;rootrank;Bacteria;domain;"Proteobacteria";phylum;Betaproteobacteria;class;Burkholderiales;order;Comamonadaceae;family;Polaromonas;genus	98.22	0	688
2530	0	0	0	12	1	0	0	0	0	Proteobacteria	Betaproteobacteria	Gallionellales	Gallionellaceae		S000873552 Candidatus Nitrotoga arctica; 6680 Lineage=Root;rootrank;Bacteria;domain;"Proteobacteria";phylum;Betaproteobacteria;class;unclassified_Betaproteobacteria;	99.46	0	676
2682	0	0	0	1	0	0	0	0	0	Proteobacteria	Betaproteobacteria	Methylophilales	Methylophilaceae		S002304658 Methylobacillus glycogenes; type strain: DSM5685 Lineage=Root;rootrank;Bacteria;domain;"Proteobacteria";phylum;Betaproteobacteria;class;Methylophilales;order;Methylophilaceae;family;Methylobacillus;genus	97.58	0	638
2080	0	0	1	1	0	0	0	0	0	Proteobacteria	Betaproteobacteria	Nitrosomonadales	Nitrosomonadaceae	Nitrosomonas	S000340522 Nitrosomonas aestuarii Lineage=Root;rootrank;Bacteria;domain;"Proteobacteria";phylum;Betaproteobacteria;class;Nitrosomonadales;order;Nitrosomonadaceae;family;unclassified_Nitrosomonadaceae;	97.57	2.00E-180	632
2384	0	0	0	0	0	0	0	0	1	Proteobacteria	Betaproteobacteria	Procabacteriales	Procabacteriaceae	Procabacter	S000388686 Candidatus Procabacter acanthamoebae Lineage=Root;rootrank;Bacteria;domain;"Proteobacteria";phylum;Betaproteobacteria;class;unclassified_Betaproteobacteria;	98.12	0	647
1611	0	0	0	1	0	3	2	5	1	Proteobacteria	Betaproteobacteria	Rhodocyclales			S000008376 Azoarcus buckelii (T); type strain: U120 = DSM 14744 Lineage=Root;rootrank;Bacteria;domain;"Proteobacteria";phylum;Betaproteobacteria;class;Rhodocyclales;order;Rhodocyclaceae;family;Azoarcus;genus	92.25	1.00E-148	527
2272	0	0	0	0	0	0	0	0	1	Proteobacteria	Betaproteobacteria	Rhodocyclales			S000008376 Azoarcus buckelii (T); type strain: U120 = DSM 14744 Lineage=Root;rootrank;Bacteria;domain;"Proteobacteria";phylum;Betaproteobacteria;class;Rhodocyclales;order;Rhodocyclaceae;family;Azoarcus;genus	94.4	1.00E-162	573
256	0	0	0	40	17	10	2	3	18	Proteobacteria	Betaproteobacteria	Rhodocyclales			S000469824 Thiobacter subterraneus (T); C55 Lineage=Root;rootrank;Bacteria;domain;"Proteobacteria";phylum;Betaproteobacteria;class;Burkholderiales;order;Burkholderiales_incertae_sedis;family;Thiobacter;genus	90.86	3.00E-139	496
1263	0	0	0	1	3	0	0	0	0	Proteobacteria	Betaproteobacteria	Rhodocyclales			S000860635 Duganella nigrescens; YIM H16 Lineage=Root;rootrank;Bacteria;domain;"Proteobacteria";phylum;Betaproteobacteria;class;Burkholderiales;order;Oxalobacteraceae;family;unclassified_Oxalobacteraceae;	93.01	4.00E-153	542
3015	0	0	0	13	4	0	0	0	0	Proteobacteria	Betaproteobacteria	Rhodocyclales			S001015527 Thauera phenylacetica; b208 Lineage=Root;rootrank;Bacteria;domain;"Proteobacteria";phylum;Betaproteobacteria;class;Rhodocyclales;order;Rhodocyclaceae;family;Thauera;genus	93.35	3.00E-154	545
825	0	0	0	0	2	0	0	0	0	Proteobacteria	Betaproteobacteria	Rhodocyclales			S001872154 Sulfuricella denitrificans (T); NBRC 105220 Lineage=Root;rootrank;Bacteria;domain;"Proteobacteria";phylum;Betaproteobacteria;class;Hydrogenophilales;order;Hydrogenophilaceae;family;Sulfuricella;genus	92.53	6.00E-151	534
2474	0	0	0	8	3	0	0	0	0	Proteobacteria	Betaproteobacteria	Rhodocyclales			S002150824 Polynucleobacter cosmopolitanus; MWH-MoK3 Lineage=Root;rootrank;Bacteria;domain;"Proteobacteria";phylum;Betaproteobacteria;class;Burkholderiales;order;Burkholderiaceae;family;Polynucleobacter;genus	90.62	4.00E-138	492
1581	0	0	2	31	42	3	4	4	59	Proteobacteria	Betaproteobacteria	Rhodocyclales			S002289911 Methylobacillus flagellatus KT Lineage=Root;rootrank;Bacteria;domain;"Proteobacteria";phylum;Betaproteobacteria;class;Methylophilales;order;Methylophilaceae;family;Methylobacillus;genus	89.81	4.00E-133	475
986	0	0	3	22	18	4	4	3	13	Proteobacteria	Betaproteobacteria	Rhodocyclales			S002289911 Methylobacillus flagellatus KT Lineage=Root;rootrank;Bacteria;domain;"Proteobacteria";phylum;Betaproteobacteria;class;Methylophilales;order;Methylophilaceae;family;Methylobacillus;genus	90.05	1.00E-142	507
1988	0	0	0	0	1	0	0	0	0	Proteobacteria	Betaproteobacteria	Rhodocyclales			S003261645 Burkholderia symbiont of Cavelerius saccharivorus; MDT50 Lineage=Root;rootrank;Bacteria;domain;"Proteobacteria";phylum;Betaproteobacteria;class;Burkholderiales;order;Burkholderiaceae;family;Burkholderia;genus	92.74	2.00E-151	536
1609	0	0	4	52	35	20	8	43	51	Proteobacteria	Betaproteobacteria	Rhodocyclales			S003264328 Chitinimonas koreensis; NBRC 106425 Lineage=Root;rootrank;Bacteria;domain;"Proteobacteria";phylum;Betaproteobacteria;class;Burkholderiales;order;Burkholderiaceae;family;Chitinimonas;genus	93.53	6.00E-156	551
2565	0	0	0	0	0	0	1	0	0	Proteobacteria	Betaproteobacteria	Rhodocyclales			S003264328 Chitinimonas koreensis; NBRC 106425 Lineage=Root;rootrank;Bacteria;domain;"Proteobacteria";phylum;Betaproteobacteria;class;Burkholderiales;order;Burkholderiaceae;family;Chitinimonas;genus	91.58	5.00E-152	538
2619	0	0	1	2	5	0	0	0	0	Proteobacteria	Betaproteobacteria	Rhodocyclales			S003288319 Thiobacillus aquaesulis; type strain: DSM 4255 Lineage=Root;rootrank;Bacteria;domain;"Proteobacteria";phylum;Betaproteobacteria;class;Hydrogenophilales;order;Hydrogenophilaceae;family;Thiobacillus;genus	93.57	6.00E-156	551
2585	0	0	0	3	0	0	0	1	3	Proteobacteria	Betaproteobacteria	Rhodocyclales			S003288319 Thiobacillus aquaesulis; type strain: DSM 4255 Lineage=Root;rootrank;Bacteria;domain;"Proteobacteria";phylum;Betaproteobacteria;class;Hydrogenophilales;order;Hydrogenophilaceae;family;Thiobacillus;genus	90.62	1.00E-138	494
1384	0	0	5	73	109	196	134	85	255	Proteobacteria	Betaproteobacteria	Rhodocyclales			S003290114 Burkholderia phenazinium; TA6 Lineage=Root;rootrank;Bacteria;domain;"Proteobacteria";phylum;Betaproteobacteria;class;Burkholderiales;order;Burkholderiaceae;family;Burkholderia;genus	92.74	6.00E-151	534
2838	0	0	3	22	9	7	5	2	3	Proteobacteria	Betaproteobacteria	Rhodocyclales			S003718976 Limnobacter thiooxidans; SSW137 Lineage=Root;rootrank;Bacteria;domain;"Proteobacteria";phylum;Betaproteobacteria;class;Burkholderiales;order;Burkholderiaceae;family;Limnobacter;genus	93.09	2.00E-161	569
650	0	0	1	7	6	0	0	0	0	Proteobacteria	Betaproteobacteria	Rhodocyclales			S003749475 Burkholderia andropogonis; LMG 2129 Lineage=Root;rootrank;Bacteria;domain;"Proteobacteria";phylum;Betaproteobacteria;class;Burkholderiales;order;Burkholderiaceae;family;Burkholderia;genus	94.05	3.00E-159	562
1949	0	0	0	8	7	0	0	0	0	Proteobacteria	Betaproteobacteria	Rhodocyclales			S002235982 Sulfuritalea hydrogenivorans (T); DSM 22779 Lineage=Root;rootrank;Bacteria;domain;"Proteobacteria";phylum;Betaproteobacteria;class;Rhodocyclales;order;Rhodocyclaceae;family;Sulfuritalea;genus	94.89	2.00E-165	582
673	0	0	2	151	45	2	2	81	3	Proteobacteria	Betaproteobacteria				S000016637 Nitrosococcus mobilis; Nc2 Lineage=Root;rootrank;Bacteria;domain;"Proteobacteria";phylum;Betaproteobacteria;class;Nitrosomonadales;order;Nitrosomonadaceae;family;unclassified_Nitrosomonadaceae;	98.65	0	656
2532	0	0	0	0	1	0	0	0	0	Proteobacteria	Betaproteobacteria				S002351616 Burkholderia endofungorum; G4101 Lineage=Root;rootrank;Bacteria;domain;"Proteobacteria";phylum;Betaproteobacteria;class;Burkholderiales;order;Burkholderiaceae;family;Burkholderia;genus	91.94	2.00E-156	553
284	0	0	0	0	1	0	0	0	0	Proteobacteria	Deltaproteobacteria	Syntrophaceae			S000388299 Clostridium peptidivorans (T); TMC4; DSM 12505 Lineage=Root;rootrank;Bacteria;domain;Firmicutes;phylum;Clostridia;class;Clostridiales;order;Clostridiaceae 1;family;Clostridium sensu stricto;genus	88.44	1.00E-123	444
3093	0	1	0	0	0	0	0	0	0	Proteobacteria	Deltaproteobacteria	Syntrophaceae			S000391487 Gelria glutamica (T); TGO Lineage=Root;rootrank;Bacteria;domain;Firmicutes;phylum;Clostridia;class;Thermoanaerobacterales;order;Thermoanaerobacteraceae;family;Gelria;genus	87.31	2.00E-122	440
350	0	0	0	0	0	0	0	0	1	Proteobacteria	Deltaproteobacteria	Syntrophaceae			S000391487 Gelria glutamica (T); TGO Lineage=Root;rootrank;Bacteria;domain;Firmicutes;phylum;Clostridia;class;Thermoanaerobacterales;order;Thermoanaerobacteraceae;family;Gelria;genus	81.96	1.00E-82	307
2769	1	0	0	0	0	0	0	0	0	Proteobacteria	Deltaproteobacteria	Syntrophaceae			S000396664 endosymbiont of Lamellibrachia barhami; host individual 11 Lineage=Root;rootrank;Bacteria;domain;"Proteobacteria";phylum;Gammaproteobacteria;class;Gammaproteobacteria_incertae_sedis;order;unclassified_Gammaproteobacteria_incertae_sedis;	87.47	3.00E-125	449
1362	0	0	0	1	0	0	0	0	0	Proteobacteria	Deltaproteobacteria	Syntrophaceae			S000436485 Sporohalobacter lortetii (T) Lineage=Root;rootrank;Bacteria;domain;Firmicutes;phylum;Clostridia;class;Halanaerobiales;order;Halobacteroidaceae;family;Sporohalobacter;genus	84.87	8.00E-36	152
2156	0	0	0	1	0	0	0	0	0	Proteobacteria	Deltaproteobacteria	Syntrophaceae			S000752077 Moorella perchloratireducens; An10 Lineage=Root;rootrank;Bacteria;domain;Firmicutes;phylum;Clostridia;class;Thermoanaerobacterales;order;Thermoanaerobacteraceae;family;Moorella;genus	87.5	3.00E-125	449
1016	0	0	0	0	0	0	0	1	0	Proteobacteria	Deltaproteobacteria	Syntrophaceae			S000841843 Rubrobacter taiwanensis (T); LS-293 Lineage=Root;rootrank;Bacteria;domain;"Actinobacteria";phylum;Actinobacteria;class;Rubrobacteridae;subclass;Rubrobacterales;order;"Rubrobacterineae";suborder;Rubrobacteraceae;family;Rubrobacter;genus	86.52	2.00E-111	403
1382	0	0	0	0	0	0	1	0	0	Proteobacteria	Deltaproteobacteria	Syntrophaceae			S000842814 Dethiobacter alkaliphilus (T); AHT 1 Lineage=Root;rootrank;Bacteria;domain;Firmicutes;phylum;Clostridia;class;Natranaerobiales;order;Natranaerobiaceae;family;Dethiobacter;genus	88.32	5.00E-122	438
1315	0	0	0	0	0	0	0	0	1	Proteobacteria	Deltaproteobacteria	Syntrophaceae			S001168707 Olavius ilvae Delta 3 endosymbiont; O. ilvae Individual 5; OilvEIII33-4 Lineage=Root;rootrank;Bacteria;domain;"Proteobacteria";phylum;Deltaproteobacteria;class;Desulfobacterales;order;Desulfobacteraceae;family;Desulfonema;genus	85.07	1.00E-102	374
2110	0	0	1	0	0	0	0	0	0	Proteobacteria	Deltaproteobacteria	Syntrophaceae			S003279968 Pseudomonas syringae pv. theae; KIWI-ZZ Lineage=Root;rootrank;Bacteria;domain;"Proteobacteria";phylum;Gammaproteobacteria;class;Pseudomonadales;order;Pseudomonadaceae;family;Pseudomonas;genus	81.78	7.00E-46	185
2982	0	0	2	4	2	10	3	3	1	Proteobacteria	Deltaproteobacteria				S000022588 Haliangium tepidum (T); SMP-10 Lineage=Root;rootrank;Bacteria;domain;"Proteobacteria";phylum;Deltaproteobacteria;class;Myxococcales;order;Nannocystineae;suborder;"Haliangiaceae";family;Haliangium;genus	84.82	2.00E-100	366
516	0	0	0	0	2	0	0	0	0	Proteobacteria	Deltaproteobacteria				S000437417 Desulfuromonas palmitatis (T); SDBY1 Lineage=Root;rootrank;Bacteria;domain;"Proteobacteria";phylum;Deltaproteobacteria;class;Desulfuromonadales;order;Desulfuromonadaceae;family;Desulfuromonas;genus	89	2.00E-136	486
3072	0	0	0	1	0	0	0	0	0	Proteobacteria	Deltaproteobacteria				S000437417 Desulfuromonas palmitatis (T); SDBY1 Lineage=Root;rootrank;Bacteria;domain;"Proteobacteria";phylum;Deltaproteobacteria;class;Desulfuromonadales;order;Desulfuromonadaceae;family;Desulfuromonas;genus	86.18	2.00E-110	399
1682	0	1	0	0	0	0	0	0	0	Proteobacteria	Deltaproteobacteria				S000769397 Haliangium ochraceum; GS1 Lineage=Root;rootrank;Bacteria;domain;"Proteobacteria";phylum;Deltaproteobacteria;class;Myxococcales;order;Nannocystineae;suborder;"Haliangiaceae";family;Haliangium;genus	87.85	2.00E-127	457
112	0	1	0	0	0	0	0	0	0	Proteobacteria	Deltaproteobacteria				S002289057 Haliangium ochraceum DSM 14365 Lineage=Root;rootrank;Bacteria;domain;"Proteobacteria";phylum;Deltaproteobacteria;class;Myxococcales;order;Nannocystineae;suborder;"Haliangiaceae";family;Haliangium;genus	87.28	9.00E-125	448
1180	0	2	0	0	1	0	0	1	3	Proteobacteria	Deltaproteobacteria				S003263888 Sneathiella glossodoripedis; NBRC 103391 Lineage=Root;rootrank;Bacteria;domain;"Proteobacteria";phylum;Alphaproteobacteria;class;Sneathiellales;order;Sneathiellaceae;family;Sneathiella;genus	87.26	2.00E-115	416
2644	0	0	0	1	0	0	0	0	0	Proteobacteria	Deltaproteobacteria				S003263888 Sneathiella glossodoripedis; NBRC 103391 Lineage=Root;rootrank;Bacteria;domain;"Proteobacteria";phylum;Alphaproteobacteria;class;Sneathiellales;order;Sneathiellaceae;family;Sneathiella;genus	88.55	9.00E-120	431
3077	0	0	0	1	0	0	0	0	1	Proteobacteria	Deltaproteobacteria				S003263891 Sneathiella chinensis; NBRC 103408 Lineage=Root;rootrank;Bacteria;domain;"Proteobacteria";phylum;Alphaproteobacteria;class;Sneathiellales;order;Sneathiellaceae;family;Sneathiella;genus	87.23	2.00E-115	416
1901	0	2	0	0	0	0	1	1	0	Proteobacteria	Deltaproteobacteria				S003611761 Kofleria flava; DSM:14620; K2 Lineage=Root;rootrank;Bacteria;domain;"Proteobacteria";phylum;Deltaproteobacteria;class;Myxococcales;order;Nannocystineae;suborder;unclassified_Nannocystineae;	86.62	9.00E-120	431
963	0	0	0	0	1	0	0	0	0	Proteobacteria	Deltaproteobacteria	Bdellovibrionales	Bacteriovoracaceae	Bacteriovorax	S000540435 Bacteriovorax litoralis; JS5 Lineage=Root;rootrank;Bacteria;domain;"Proteobacteria";phylum;Deltaproteobacteria;class;Bdellovibrionales;order;Bacteriovoracaceae;family;Bacteriovorax;genus	91.05	1.00E-148	527
3046	0	0	0	1	0	0	0	0	0	Proteobacteria	Deltaproteobacteria	Bdellovibrionales	Bacteriovoracaceae	Bacteriovorax	S000540435 Bacteriovorax litoralis; JS5 Lineage=Root;rootrank;Bacteria;domain;"Proteobacteria";phylum;Deltaproteobacteria;class;Bdellovibrionales;order;Bacteriovoracaceae;family;Bacteriovorax;genus	92.88	7.00E-161	568
992	0	0	0	1	0	0	0	0	0	Proteobacteria	Deltaproteobacteria	Bdellovibrionales	Bdellovibrionaceae	Bdellovibrio	S003717708 Bdellovibrio bacteriovorus; DSAR-3 Lineage=Root;rootrank;Bacteria;domain;"Proteobacteria";phylum;Deltaproteobacteria;class;Bdellovibrionales;order;Bdellovibrionaceae;family;Bdellovibrio;genus	87.4	1.00E-118	427
873	0	0	0	0	0	0	0	1	0	Proteobacteria	Deltaproteobacteria	CTD005-82B-02			S000387799 Desulfomicrobium hypogeium; CN-A Lineage=Root;rootrank;Bacteria;domain;"Proteobacteria";phylum;Deltaproteobacteria;class;Desulfovibrionales;order;Desulfomicrobiaceae;family;Desulfomicrobium;genus	86.22	9.00E-110	398
2231	0	0	0	0	1	0	0	0	0	Proteobacteria	Deltaproteobacteria	CTD005-82B-02			S001014826 Spirobacillus cienkowskii Lineage=Root;rootrank;Bacteria;domain;"Proteobacteria";phylum;unclassified_"Proteobacteria";	84.99	4.00E-103	375
2990	1	0	0	0	0	0	0	0	0	Proteobacteria	Deltaproteobacteria	CTD005-82B-02			S002224490 Chondromyces pediculatus; Cm p51 Lineage=Root;rootrank;Bacteria;domain;"Proteobacteria";phylum;Deltaproteobacteria;class;Myxococcales;order;Sorangiineae;suborder;Polyangiaceae;family;Chondromyces;genus	86.99	6.00E-122	438
1523	0	1	0	0	0	0	0	0	0	Proteobacteria	Deltaproteobacteria	CTD005-82B-02			S002224578 Jahnella thaxteri; SBSr007 Lineage=Root;rootrank;Bacteria;domain;"Proteobacteria";phylum;Deltaproteobacteria;class;Myxococcales;order;Sorangiineae;suborder;Polyangiaceae;family;Chondromyces;genus	86.36	2.00E-111	403
1322	0	0	0	2	1	0	0	0	0	Proteobacteria	Deltaproteobacteria	CTD005-82B-02			S002228848 Sphingomonas dokdonensis; BAC2037 Lineage=Root;rootrank;Bacteria;domain;"Proteobacteria";phylum;Alphaproteobacteria;class;Sphingomonadales;order;Sphingomonadaceae;family;Sphingomonas;genus	86.36	2.00E-117	424
502	0	0	0	0	0	0	0	2	0	Proteobacteria	Deltaproteobacteria	CTD005-82B-02			S002950110 Desulfovibrio alaskensis G20 Lineage=Root;rootrank;Bacteria;domain;"Proteobacteria";phylum;Deltaproteobacteria;class;Desulfovibrionales;order;Desulfovibrionaceae;family;Desulfovibrio;genus	86.55	4.00E-118	425
302	0	0	0	1	0	0	0	2	0	Proteobacteria	Deltaproteobacteria	CTD005-82B-02			S003222495 Desulfomicrobium orale; OB018 Lineage=Root;rootrank;Bacteria;domain;"Proteobacteria";phylum;Deltaproteobacteria;class;Desulfovibrionales;order;Desulfomicrobiaceae;family;Desulfomicrobium;genus	85.98	5.00E-107	388
66	0	0	0	0	1	0	0	0	0	Proteobacteria	Deltaproteobacteria	CTD005-82B-02			S003222495 Desulfomicrobium orale; OB018 Lineage=Root;rootrank;Bacteria;domain;"Proteobacteria";phylum;Deltaproteobacteria;class;Desulfovibrionales;order;Desulfomicrobiaceae;family;Desulfomicrobium;genus	85.6	2.00E-105	383
332	0	0	2	21	29	38	40	29	24	Proteobacteria	Deltaproteobacteria	Entotheonellales	Entotheonellaceae	CandidatusEntotheonella	S000024027 Candidatus Entotheonella palauensis Lineage=Root;rootrank;Bacteria;domain;unclassified_Bacteria;	92.53	8.00E-150	531
1568	0	0	0	0	0	42	42	1	0	Proteobacteria	Deltaproteobacteria	Entotheonellales	Entotheonellaceae	CandidatusEntotheonella	S000024027 Candidatus Entotheonella palauensis Lineage=Root;rootrank;Bacteria;domain;unclassified_Bacteria;	91.96	2.00E-146	520
1401	0	2	4	2	0	32	14	1	0	Proteobacteria	Deltaproteobacteria	Entotheonellales	Entotheonellaceae	CandidatusEntotheonella	S000024027 Candidatus Entotheonella palauensis Lineage=Root;rootrank;Bacteria;domain;unclassified_Bacteria;	91.15	2.00E-141	503
1989	0	0	0	1	1	0	0	1	25	Proteobacteria	Deltaproteobacteria	Entotheonellales	Entotheonellaceae	CandidatusEntotheonella	S000024027 Candidatus Entotheonella palauensis Lineage=Root;rootrank;Bacteria;domain;unclassified_Bacteria;	92.09	2.00E-155	549
2837	0	0	0	1	0	2	0	0	9	Proteobacteria	Deltaproteobacteria	Entotheonellales	Entotheonellaceae	CandidatusEntotheonella	S000024027 Candidatus Entotheonella palauensis Lineage=Root;rootrank;Bacteria;domain;unclassified_Bacteria;	92.86	2.00E-160	566
2774	0	0	2	0	5	0	0	1	0	Proteobacteria	Deltaproteobacteria	Entotheonellales	Entotheonellaceae	CandidatusEntotheonella	S000024027 Candidatus Entotheonella palauensis Lineage=Root;rootrank;Bacteria;domain;unclassified_Bacteria;	94.89	3.00E-164	579
431	0	0	0	0	3	0	0	0	1	Proteobacteria	Deltaproteobacteria	Entotheonellales	Entotheonellaceae	CandidatusEntotheonella	S000024027 Candidatus Entotheonella palauensis Lineage=Root;rootrank;Bacteria;domain;unclassified_Bacteria;	90.28	1.00E-143	510
2690	0	0	0	1	0	0	0	0	0	Proteobacteria	Deltaproteobacteria	Entotheonellales	Entotheonellaceae	CandidatusEntotheonella	S000024027 Candidatus Entotheonella palauensis Lineage=Root;rootrank;Bacteria;domain;unclassified_Bacteria;	91.37	9.00E-150	531
723	0	0	0	1	0	0	0	0	0	Proteobacteria	Deltaproteobacteria	FAC87			S000570998 Desulfobulbus mediterraneus; NA62 Lineage=Root;rootrank;Bacteria;domain;"Proteobacteria";phylum;Deltaproteobacteria;class;Desulfobacterales;order;Desulfobulbaceae;family;Desulfobulbus;genus	85.06	3.00E-109	396
1716	0	0	0	1	0	0	0	0	0	Proteobacteria	Deltaproteobacteria	MIZ46			S000437296 Desulfuromonas acetexigens (T) Lineage=Root;rootrank;Bacteria;domain;"Proteobacteria";phylum;Deltaproteobacteria;class;Desulfuromonadales;order;Desulfuromonadaceae;family;Desulfuromonas;genus	84	5.00E-97	355
2709	0	0	0	1	0	0	0	0	0	Proteobacteria	Deltaproteobacteria	MIZ46			S000437296 Desulfuromonas acetexigens (T) Lineage=Root;rootrank;Bacteria;domain;"Proteobacteria";phylum;Deltaproteobacteria;class;Desulfuromonadales;order;Desulfuromonadaceae;family;Desulfuromonas;genus	85.24	1.00E-108	394
3135	0	0	0	0	0	0	0	1	0	Proteobacteria	Deltaproteobacteria	MIZ46			S000437296 Desulfuromonas acetexigens (T) Lineage=Root;rootrank;Bacteria;domain;"Proteobacteria";phylum;Deltaproteobacteria;class;Desulfuromonadales;order;Desulfuromonadaceae;family;Desulfuromonas;genus	85.82	1.00E-113	411
139	0	0	0	0	0	0	0	0	1	Proteobacteria	Deltaproteobacteria	MIZ46			S000437296 Desulfuromonas acetexigens (T) Lineage=Root;rootrank;Bacteria;domain;"Proteobacteria";phylum;Deltaproteobacteria;class;Desulfuromonadales;order;Desulfuromonadaceae;family;Desulfuromonas;genus	84.66	7.00E-101	368
527	0	0	0	0	1	0	0	1	0	Proteobacteria	Deltaproteobacteria	MIZ46			S000438916 Geobacter pelophilus (T); Dfr2 Lineage=Root;rootrank;Bacteria;domain;"Proteobacteria";phylum;Deltaproteobacteria;class;Desulfuromonadales;order;Geobacteraceae;family;Geobacter;genus	82.71	1.00E-88	327
1363	0	0	0	0	0	0	1	0	1	Proteobacteria	Deltaproteobacteria	MIZ46			S000438916 Geobacter pelophilus (T); Dfr2 Lineage=Root;rootrank;Bacteria;domain;"Proteobacteria";phylum;Deltaproteobacteria;class;Desulfuromonadales;order;Geobacteraceae;family;Geobacter;genus	83.86	2.00E-95	350
344	0	0	0	0	1	0	0	0	0	Proteobacteria	Deltaproteobacteria	MIZ46			S000438916 Geobacter pelophilus (T); Dfr2 Lineage=Root;rootrank;Bacteria;domain;"Proteobacteria";phylum;Deltaproteobacteria;class;Desulfuromonadales;order;Geobacteraceae;family;Geobacter;genus	84.49	9.00E-100	364
1527	0	0	0	0	0	0	0	0	1	Proteobacteria	Deltaproteobacteria	MIZ46			S000438916 Geobacter pelophilus (T); Dfr2 Lineage=Root;rootrank;Bacteria;domain;"Proteobacteria";phylum;Deltaproteobacteria;class;Desulfuromonadales;order;Geobacteraceae;family;Geobacter;genus	85.87	1.00E-108	394
601	0	1	0	0	0	0	0	0	0	Proteobacteria	Deltaproteobacteria	MIZ46			S000574366 Geobacter psychrophilus (T); P35 Lineage=Root;rootrank;Bacteria;domain;"Proteobacteria";phylum;Deltaproteobacteria;class;Desulfuromonadales;order;Geobacteraceae;family;Geobacter;genus	83.63	1.00E-98	361
1136	0	0	0	0	0	0	0	1	0	Proteobacteria	Deltaproteobacteria	MIZ46			S000574366 Geobacter psychrophilus (T); P35 Lineage=Root;rootrank;Bacteria;domain;"Proteobacteria";phylum;Deltaproteobacteria;class;Desulfuromonadales;order;Geobacteraceae;family;Geobacter;genus	83.38	2.00E-96	353
2944	0	0	0	0	0	0	0	0	1	Proteobacteria	Deltaproteobacteria	MIZ46			S000650984 Desulfoglaeba alkanexedens (T); ALDC Lineage=Root;rootrank;Bacteria;domain;"Proteobacteria";phylum;Deltaproteobacteria;class;Syntrophobacterales;order;Syntrophobacteraceae;family;Desulfoglaeba;genus	84.14	1.00E-98	361
976	0	0	0	1	0	0	0	0	0	Proteobacteria	Deltaproteobacteria	MIZ46			S002288161 Geobacter bemidjiensis Bem Lineage=Root;rootrank;Bacteria;domain;"Proteobacteria";phylum;Deltaproteobacteria;class;Desulfuromonadales;order;Geobacteraceae;family;Geobacter;genus	83.82	2.00E-95	350
2522	0	0	0	1	0	0	0	0	0	Proteobacteria	Deltaproteobacteria	MIZ46			S002289057 Haliangium ochraceum DSM 14365 Lineage=Root;rootrank;Bacteria;domain;"Proteobacteria";phylum;Deltaproteobacteria;class;Myxococcales;order;Nannocystineae;suborder;"Haliangiaceae";family;Haliangium;genus	84.99	3.00E-109	396
2601	0	0	0	1	1	0	0	1	0	Proteobacteria	Deltaproteobacteria	MIZ46			S002290737 Pelobacter propionicus (T); DSM 2379 Lineage=Root;rootrank;Bacteria;domain;"Proteobacteria";phylum;Deltaproteobacteria;class;Desulfuromonadales;order;Desulfuromonadaceae;family;Pelobacter;genus	83.91	7.00E-96	351
1698	0	0	0	0	1	0	0	0	0	Proteobacteria	Deltaproteobacteria	MIZ46			S002290737 Pelobacter propionicus (T); DSM 2379 Lineage=Root;rootrank;Bacteria;domain;"Proteobacteria";phylum;Deltaproteobacteria;class;Desulfuromonadales;order;Desulfuromonadaceae;family;Pelobacter;genus	85.07	1.00E-102	374
610	0	0	0	0	0	1	0	0	0	Proteobacteria	Deltaproteobacteria	MIZ46			S002290737 Pelobacter propionicus (T); DSM 2379 Lineage=Root;rootrank;Bacteria;domain;"Proteobacteria";phylum;Deltaproteobacteria;class;Desulfuromonadales;order;Desulfuromonadaceae;family;Pelobacter;genus	83.87	2.00E-95	350
1378	0	0	0	0	0	0	1	0	0	Proteobacteria	Deltaproteobacteria	MIZ46			S002290737 Pelobacter propionicus (T); DSM 2379 Lineage=Root;rootrank;Bacteria;domain;"Proteobacteria";phylum;Deltaproteobacteria;class;Desulfuromonadales;order;Desulfuromonadaceae;family;Pelobacter;genus	85.22	1.00E-103	377
2406	0	0	0	0	0	0	0	1	0	Proteobacteria	Deltaproteobacteria	MIZ46			S002290737 Pelobacter propionicus (T); DSM 2379 Lineage=Root;rootrank;Bacteria;domain;"Proteobacteria";phylum;Deltaproteobacteria;class;Desulfuromonadales;order;Desulfuromonadaceae;family;Pelobacter;genus	84.99	5.00E-102	372
1120	0	0	0	0	0	0	0	0	1	Proteobacteria	Deltaproteobacteria	MIZ46			S002290737 Pelobacter propionicus (T); DSM 2379 Lineage=Root;rootrank;Bacteria;domain;"Proteobacteria";phylum;Deltaproteobacteria;class;Desulfuromonadales;order;Desulfuromonadaceae;family;Pelobacter;genus	83.76	9.00E-100	364
1756	0	0	0	1	1	0	0	0	2	Proteobacteria	Deltaproteobacteria	MIZ46			S002349656 Desulfofrigus oceanense Lineage=Root;rootrank;Bacteria;domain;"Proteobacteria";phylum;unclassified_"Proteobacteria";	94.65	3.00E-164	579
883	0	0	0	0	1	0	0	0	0	Proteobacteria	Deltaproteobacteria	MIZ46			S003610437 Pelobacter carbinolicus (T); DSM 2380 Lineage=Root;rootrank;Bacteria;domain;"Proteobacteria";phylum;Deltaproteobacteria;class;Desulfuromonadales;order;Desulfuromonadaceae;family;Pelobacter;genus	85.5	2.00E-112	407
990	0	0	0	0	0	1	0	0	0	Proteobacteria	Deltaproteobacteria	MIZ46			S003611761 Kofleria flava; DSM:14620; K2 Lineage=Root;rootrank;Bacteria;domain;"Proteobacteria";phylum;Deltaproteobacteria;class;Myxococcales;order;Nannocystineae;suborder;unclassified_Nannocystineae;	85.5	2.00E-112	407
1471	0	0	0	0	0	0	0	1	0	Proteobacteria	Deltaproteobacteria	MIZ46			S003611761 Kofleria flava; DSM:14620; K2 Lineage=Root;rootrank;Bacteria;domain;"Proteobacteria";phylum;Deltaproteobacteria;class;Myxococcales;order;Nannocystineae;suborder;unclassified_Nannocystineae;	85.25	2.00E-105	383
85	0	0	0	0	0	1	1	0	0	Proteobacteria	Deltaproteobacteria	MIZ46			S003614644 Pyxidicoccus fallax; S127 Lineage=Root;rootrank;Bacteria;domain;"Proteobacteria";phylum;Deltaproteobacteria;class;Myxococcales;order;Cystobacterineae;suborder;Myxococcaceae;family;unclassified_Myxococcaceae;	84.31	4.00E-98	359
826	0	0	0	1	0	0	0	0	0	Proteobacteria	Deltaproteobacteria	MIZ46			S003614644 Pyxidicoccus fallax; S127 Lineage=Root;rootrank;Bacteria;domain;"Proteobacteria";phylum;Deltaproteobacteria;class;Myxococcales;order;Cystobacterineae;suborder;Myxococcaceae;family;unclassified_Myxococcaceae;	84.62	3.00E-105	383
315	0	0	0	0	0	1	0	1	0	Proteobacteria	Deltaproteobacteria	MIZ46			S000022286 magnetic coccus MP17; TB24 Lineage=Root;rootrank;Bacteria;domain;"Proteobacteria";phylum;unclassified_"Proteobacteria";	84	5.00E-97	355
1373	0	0	0	0	1	0	0	0	0	Proteobacteria	Deltaproteobacteria	MIZ46			S000022588 Haliangium tepidum (T); SMP-10 Lineage=Root;rootrank;Bacteria;domain;"Proteobacteria";phylum;Deltaproteobacteria;class;Myxococcales;order;Nannocystineae;suborder;"Haliangiaceae";family;Haliangium;genus	83.78	2.00E-95	350
2448	0	0	0	0	0	1	0	0	0	Proteobacteria	Deltaproteobacteria	MIZ46			S001153278 Methylogaea oryzae JCM 16910; E10 Lineage=Root;rootrank;Bacteria;domain;"Proteobacteria";phylum;Gammaproteobacteria;class;Methylococcales;order;Methylococcaceae;family;Methylococcus;genus	83.64	3.00E-94	346
3096	0	0	0	0	1	0	0	2	0	Proteobacteria	Deltaproteobacteria	MIZ46			S003610437 Pelobacter carbinolicus (T); DSM 2380 Lineage=Root;rootrank;Bacteria;domain;"Proteobacteria";phylum;Deltaproteobacteria;class;Desulfuromonadales;order;Desulfuromonadaceae;family;Pelobacter;genus	85.2	7.00E-111	401
346	0	0	0	0	1	0	0	0	0	Proteobacteria	Deltaproteobacteria	Myxococcales			S000417333 Byssovorax cruenta (T); type strain: By c2 = DSM 14553 Lineage=Root;rootrank;Bacteria;domain;"Proteobacteria";phylum;Deltaproteobacteria;class;Myxococcales;order;Sorangiineae;suborder;Polyangiaceae;family;Byssovorax;genus	90.32	5.00E-137	488
3253	0	0	0	0	1	0	0	0	0	Proteobacteria	Deltaproteobacteria	Myxococcales			S000417333 Byssovorax cruenta (T); type strain: By c2 = DSM 14553 Lineage=Root;rootrank;Bacteria;domain;"Proteobacteria";phylum;Deltaproteobacteria;class;Myxococcales;order;Sorangiineae;suborder;Polyangiaceae;family;Byssovorax;genus	92.07	7.00E-156	551
1509	0	0	0	1	0	0	0	0	0	Proteobacteria	Deltaproteobacteria	Myxococcales			S000721671 Chondromyces apiculatus; BICC 8620 Lineage=Root;rootrank;Bacteria;domain;"Proteobacteria";phylum;Deltaproteobacteria;class;Myxococcales;order;Sorangiineae;suborder;Polyangiaceae;family;Chondromyces;genus	92.74	5.00E-152	538
209	0	0	0	1	0	0	0	0	0	Proteobacteria	Deltaproteobacteria	Myxococcales			S000721674 Polyangium thaxteri; BICC 8959 Lineage=Root;rootrank;Bacteria;domain;"Proteobacteria";phylum;Deltaproteobacteria;class;Myxococcales;order;Sorangiineae;suborder;Polyangiaceae;family;Chondromyces;genus	90.51	6.00E-136	484
3051	0	0	0	1	2	0	0	1	0	Proteobacteria	Deltaproteobacteria	Myxococcales			S000722014 Cystobacter miniatus (T); DSM 14712 Lineage=Root;rootrank;Bacteria;domain;"Proteobacteria";phylum;Deltaproteobacteria;class;Myxococcales;order;Cystobacterineae;suborder;Cystobacteraceae;family;Cystobacter;genus	91.13	5.00E-142	505
3203	0	0	0	4	0	1	1	0	0	Proteobacteria	Deltaproteobacteria	Myxococcales			S000769498 Stigmatella koreensis; KYC-1019 Lineage=Root;rootrank;Bacteria;domain;"Proteobacteria";phylum;Deltaproteobacteria;class;Myxococcales;order;Cystobacterineae;suborder;unclassified_Cystobacterineae;	95.97	5.00E-172	604
1975	0	0	0	1	0	3	0	0	0	Proteobacteria	Deltaproteobacteria	Myxococcales			S001096520 Phaselicystis flava (T); SBKo001 Lineage=Root;rootrank;Bacteria;domain;"Proteobacteria";phylum;Deltaproteobacteria;class;Myxococcales;order;Sorangiineae;suborder;Phaselicystidaceae;family;Phaselicystis;genus	92.47	2.00E-150	532
2293	0	0	0	2	0	0	1	0	0	Proteobacteria	Deltaproteobacteria	Myxococcales			S001096520 Phaselicystis flava (T); SBKo001 Lineage=Root;rootrank;Bacteria;domain;"Proteobacteria";phylum;Deltaproteobacteria;class;Myxococcales;order;Sorangiineae;suborder;Phaselicystidaceae;family;Phaselicystis;genus	92.47	8.00E-150	531
2111	0	0	1	0	0	0	0	0	0	Proteobacteria	Deltaproteobacteria	Myxococcales			S001096520 Phaselicystis flava (T); SBKo001 Lineage=Root;rootrank;Bacteria;domain;"Proteobacteria";phylum;Deltaproteobacteria;class;Myxococcales;order;Sorangiineae;suborder;Phaselicystidaceae;family;Phaselicystis;genus	92.2	1.00E-148	527
808	0	0	0	1	0	0	0	0	0	Proteobacteria	Deltaproteobacteria	Myxococcales			S001096520 Phaselicystis flava (T); SBKo001 Lineage=Root;rootrank;Bacteria;domain;"Proteobacteria";phylum;Deltaproteobacteria;class;Myxococcales;order;Sorangiineae;suborder;Phaselicystidaceae;family;Phaselicystis;genus	92.2	1.00E-148	527
1562	0	0	0	3	3	4	3	3	0	Proteobacteria	Deltaproteobacteria	Myxococcales			S001188426 Anaeromyxobacter dehalogenans; FRC-D5 Lineage=Root;rootrank;Bacteria;domain;"Proteobacteria";phylum;Deltaproteobacteria;class;Myxococcales;order;Cystobacterineae;suborder;Cystobacteraceae;family;Anaeromyxobacter;genus	89.26	1.00E-137	490
2503	0	0	0	0	0	0	1	0	0	Proteobacteria	Deltaproteobacteria	Myxococcales			S001188426 Anaeromyxobacter dehalogenans; FRC-D5 Lineage=Root;rootrank;Bacteria;domain;"Proteobacteria";phylum;Deltaproteobacteria;class;Myxococcales;order;Cystobacterineae;suborder;Cystobacteraceae;family;Anaeromyxobacter;genus	91.94	5.00E-147	521
849	0	0	0	0	1	0	0	0	0	Proteobacteria	Deltaproteobacteria	Myxococcales			S001612665 Sorangium cellulosum; M5 Lineage=Root;rootrank;Bacteria;domain;"Proteobacteria";phylum;Deltaproteobacteria;class;Myxococcales;order;Sorangiineae;suborder;Polyangiaceae;family;Sorangium;genus	88.98	1.00E-128	460
430	0	0	0	1	0	0	0	0	0	Proteobacteria	Deltaproteobacteria	Myxococcales			S002224495 Polyangium sorediatum; Pl s12 Lineage=Root;rootrank;Bacteria;domain;"Proteobacteria";phylum;Deltaproteobacteria;class;Myxococcales;order;Sorangiineae;suborder;Polyangiaceae;family;unclassified_Polyangiaceae;	92.76	5.00E-152	538
2320	0	0	0	0	0	0	0	0	1	Proteobacteria	Deltaproteobacteria	Myxococcales			S002224495 Polyangium sorediatum; Pl s12 Lineage=Root;rootrank;Bacteria;domain;"Proteobacteria";phylum;Deltaproteobacteria;class;Myxococcales;order;Sorangiineae;suborder;Polyangiaceae;family;unclassified_Polyangiaceae;	94.12	2.00E-160	566
563	0	0	0	1	0	1	0	0	0	Proteobacteria	Deltaproteobacteria	Myxococcales			S002224574 Aetherobacter rufus; SBSr003 Lineage=Root;rootrank;Bacteria;domain;"Proteobacteria";phylum;Deltaproteobacteria;class;Myxococcales;order;Sorangiineae;suborder;Polyangiaceae;family;Byssovorax;genus	93.85	4.00E-158	558
2857	0	0	0	1	0	0	0	0	0	Proteobacteria	Deltaproteobacteria	Myxococcales			S002290737 Pelobacter propionicus (T); DSM 2379 Lineage=Root;rootrank;Bacteria;domain;"Proteobacteria";phylum;Deltaproteobacteria;class;Desulfuromonadales;order;Desulfuromonadaceae;family;Pelobacter;genus	90.51	5.00E-137	488
2814	0	0	1	1	1	0	0	0	0	Proteobacteria	Deltaproteobacteria	Myxococcales			S002290918 Sorangium cellulosum 'So ce 56' Lineage=Root;rootrank;Bacteria;domain;"Proteobacteria";phylum;Deltaproteobacteria;class;Myxococcales;order;Sorangiineae;suborder;Polyangiaceae;family;Sorangium;genus	93.58	5.00E-157	555

1836	0	6	0	0	0	0	2	1	0	Proteobacteria	Deltaproteobacteria	Myxococcales			S002409687 Sandaracinus amylolyticus; NOSO 4 Lineage=Root;rootrank;Bacteria;domain;"Proteobacteria";phylum;Deltaproteobacteria;class;Myxococcales;order;Sorangiineae;suborder;unclassified_Sorangiineae;	90.67	1.00E-138	494
3024	0	0	0	1	0	5	0	1	0	Proteobacteria	Deltaproteobacteria	Myxococcales			S002409687 Sandaracinus amylolyticus; NOSO 4 Lineage=Root;rootrank;Bacteria;domain;"Proteobacteria";phylum;Deltaproteobacteria;class;Myxococcales;order;Sorangiineae;suborder;unclassified_Sorangiineae;	92.25	1.00E-148	527
2461	1	0	2	0	0	0	0	0	0	Proteobacteria	Deltaproteobacteria	Myxococcales			S002409687 Sandaracinus amylolyticus; NOSO 4 Lineage=Root;rootrank;Bacteria;domain;"Proteobacteria";phylum;Deltaproteobacteria;class;Myxococcales;order;Sorangiineae;suborder;unclassified_Sorangiineae;	91.18	5.00E-142	505
1334	0	0	0	1	0	0	0	1	2	Proteobacteria	Deltaproteobacteria	Myxococcales			S002409687 Sandaracinus amylolyticus; NOSO 4 Lineage=Root;rootrank;Bacteria;domain;"Proteobacteria";phylum;Deltaproteobacteria;class;Myxococcales;order;Sorangiineae;suborder;unclassified_Sorangiineae;	92.02	5.00E-147	521
3200	0	0	0	0	0	0	1	0	0	Proteobacteria	Deltaproteobacteria	Myxococcales			S002409687 Sandaracinus amylolyticus; NOSO 4 Lineage=Root;rootrank;Bacteria;domain;"Proteobacteria";phylum;Deltaproteobacteria;class;Myxococcales;order;Sorangiineae;suborder;unclassified_Sorangiineae;	99.19	0	671
1913	0	0	1	1	6	0	1	0	1	Proteobacteria	Deltaproteobacteria	Myxococcales			S003287266 Desulfomonile tiedjei DSM 6799 Lineage=Root;rootrank;Bacteria;domain;"Proteobacteria";phylum;Deltaproteobacteria;class;Syntrophobacterales;order;Syntrophaceae;family;Desulfomonile;genus	90.19	1.00E-133	477
2104	0	0	0	0	0	0	0	1	0	Proteobacteria	Deltaproteobacteria	Myxococcales			S003611760 Kofleria flava; DSM:14659; 16 Lineage=Root;rootrank;Bacteria;domain;"Proteobacteria";phylum;Deltaproteobacteria;class;Myxococcales;order;Nannocystineae;suborder;Kofleriaceae;family;Kofleria;genus	90.62	4.00E-138	492
473	0	3	4	0	0	1	1	0	0	Proteobacteria	Deltaproteobacteria	Myxococcales	Haliangiaceae		S000769397 Haliangium ochraceum; GS1 Lineage=Root;rootrank;Bacteria;domain;"Proteobacteria";phylum;Deltaproteobacteria;class;Myxococcales;order;Nannocystineae;suborder;"Haliangiaceae";family;Haliangium;genus	94.68	4.00E-173	608
2677	0	0	0	2	1	0	0	0	0	Proteobacteria	Deltaproteobacteria	Myxococcales	Haliangiaceae		S000769397 Haliangium ochraceum; GS1 Lineage=Root;rootrank;Bacteria;domain;"Proteobacteria";phylum;Deltaproteobacteria;class;Myxococcales;order;Nannocystineae;suborder;"Haliangiaceae";family;Haliangium;genus	89.76	5.00E-132	472
96	0	0	0	0	0	0	0	0	1	Proteobacteria	Deltaproteobacteria	Myxococcales	Haliangiaceae		S000769397 Haliangium ochraceum; GS1 Lineage=Root;rootrank;Bacteria;domain;"Proteobacteria";phylum;Deltaproteobacteria;class;Myxococcales;order;Nannocystineae;suborder;"Haliangiaceae";family;Haliangium;genus	94.13	3.00E-169	595
544	0	0	0	1	0	0	0	0	0	Proteobacteria	Deltaproteobacteria	Myxococcales	Haliangiaceae		S001188426 Anaeromyxobacter dehalogenans; FRC-D5 Lineage=Root;rootrank;Bacteria;domain;"Proteobacteria";phylum;Deltaproteobacteria;class;Myxococcales;order;Cystobacterineae;suborder;Cystobacteraceae;family;Anaeromyxobacter;genus	92.18	4.00E-148	525
2679	0	0	0	0	0	1	0	1	2	Proteobacteria	Deltaproteobacteria	Myxococcales	Haliangiaceae		S002289057 Haliangium ochraceum DSM 14365 Lineage=Root;rootrank;Bacteria;domain;"Proteobacteria";phylum;Deltaproteobacteria;class;Myxococcales;order;Nannocystineae;suborder;"Haliangiaceae";family;Haliangium;genus	91.35	9.00E-150	531
2179	0	0	0	0	0	1	0	1	0	Proteobacteria	Deltaproteobacteria	Myxococcales	Haliangiaceae		S002289057 Haliangium ochraceum DSM 14365 Lineage=Root;rootrank;Bacteria;domain;"Proteobacteria";phylum;Deltaproteobacteria;class;Myxococcales;order;Nannocystineae;suborder;"Haliangiaceae";family;Haliangium;genus	92.49	2.00E-150	532
2499	0	0	0	1	0	0	0	0	0	Proteobacteria	Deltaproteobacteria	Myxococcales	Haliangiaceae		S002289057 Haliangium ochraceum DSM 14365 Lineage=Root;rootrank;Bacteria;domain;"Proteobacteria";phylum;Deltaproteobacteria;class;Myxococcales;order;Nannocystineae;suborder;"Haliangiaceae";family;Haliangium;genus	91.05	3.00E-149	529
269	0	0	4	1	2	1	6	0	1	Proteobacteria	Deltaproteobacteria	Myxococcales	Haliangiaceae		S003611760 Kofleria flava; DSM:14659; 16 Lineage=Root;rootrank;Bacteria;domain;"Proteobacteria";phylum;Deltaproteobacteria;class;Myxococcales;order;Nannocystineae;suborder;Kofleriaceae;family;Kofleria;genus	95.16	5.00E-167	588
2881	0	0	2	1	1	2	0	0	3	Proteobacteria	Deltaproteobacteria	Myxococcales	Haliangiaceae		S003611760 Kofleria flava; DSM:14659; 16 Lineage=Root;rootrank;Bacteria;domain;"Proteobacteria";phylum;Deltaproteobacteria;class;Myxococcales;order;Nannocystineae;suborder;Kofleriaceae;family;Kofleria;genus	91.67	3.00E-144	512
2710	0	0	0	4	0	0	0	0	0	Proteobacteria	Deltaproteobacteria	Myxococcales	Haliangiaceae		S003611760 Kofleria flava; DSM:14659; 16 Lineage=Root;rootrank;Bacteria;domain;"Proteobacteria";phylum;Deltaproteobacteria;class;Myxococcales;order;Nannocystineae;suborder;Kofleriaceae;family;Kofleria;genus	91.64	3.00E-144	512
840	0	0	0	2	0	0	0	0	1	Proteobacteria	Deltaproteobacteria	Myxococcales	Haliangiaceae		S003611760 Kofleria flava; DSM:14659; 16 Lineage=Root;rootrank;Bacteria;domain;"Proteobacteria";phylum;Deltaproteobacteria;class;Myxococcales;order;Nannocystineae;suborder;Kofleriaceae;family;Kofleria;genus	93.61	7.00E-166	584
2843	0	0	0	0	0	2	0	0	0	Proteobacteria	Deltaproteobacteria	Myxococcales	Haliangiaceae		S003611760 Kofleria flava; DSM:14659; 16 Lineage=Root;rootrank;Bacteria;domain;"Proteobacteria";phylum;Deltaproteobacteria;class;Myxococcales;order;Nannocystineae;suborder;Kofleriaceae;family;Kofleria;genus	94.88	3.00E-174	612
2014	5	2	4	2	3	9	2	3	1	Proteobacteria	Deltaproteobacteria	Myxococcales	Haliangiaceae	Haliangium	S000769397 Haliangium ochraceum; GS1 Lineage=Root;rootrank;Bacteria;domain;"Proteobacteria";phylum;Deltaproteobacteria;class;Myxococcales;order;Nannocystineae;suborder;"Haliangiaceae";family;Haliangium;genus	97.19	0	662
1087	4	1	0	0	0	0	0	1	0	Proteobacteria	Deltaproteobacteria	Myxococcales	Haliangiaceae	Haliangium	S002289057 Haliangium ochraceum DSM 14365 Lineage=Root;rootrank;Bacteria;domain;"Proteobacteria";phylum;Deltaproteobacteria;class;Myxococcales;order;Nannocystineae;suborder;"Haliangiaceae";family;Haliangium;genus	98.92	0	665
2402	0	0	0	0	1	0	0	0	0	Proteobacteria	Deltaproteobacteria	Myxococcales	Haliangiaceae	Haliangium	S003611760 Kofleria flava; DSM:14659; 16 Lineage=Root;rootrank;Bacteria;domain;"Proteobacteria";phylum;Deltaproteobacteria;class;Myxococcales;order;Nannocystineae;suborder;Kofleriaceae;family;Kofleria;genus	95.95	0	638
2288	0	0	0	0	1	0	0	0	0	Proteobacteria	Deltaproteobacteria	Myxococcales	Haliangiaceae		S000022588 Haliangium tepidum (T); SMP-10 Lineage=Root;rootrank;Bacteria;domain;"Proteobacteria";phylum;Deltaproteobacteria;class;Myxococcales;order;Nannocystineae;suborder;"Haliangiaceae";family;Haliangium;genus	90.72	2.00E-145	516
1792	13	4	0	6	2	0	2	1	0	Proteobacteria	Deltaproteobacteria	Myxococcales	Haliangiaceae		S000769397 Haliangium ochraceum; GS1 Lineage=Root;rootrank;Bacteria;domain;"Proteobacteria";phylum;Deltaproteobacteria;class;Myxococcales;order;Nannocystineae;suborder;"Haliangiaceae";family;Haliangium;genus	94.4	7.00E-171	601
1724	0	2	0	0	0	1	1	0	1	Proteobacteria	Deltaproteobacteria	Myxococcales	Haliangiaceae		S000769397 Haliangium ochraceum; GS1 Lineage=Root;rootrank;Bacteria;domain;"Proteobacteria";phylum;Deltaproteobacteria;class;Myxococcales;order;Nannocystineae;suborder;"Haliangiaceae";family;Haliangium;genus	96.17	0	641
1514	0	0	1	0	0	0	0	0	0	Proteobacteria	Deltaproteobacteria	Myxococcales	Haliangiaceae		S002289057 Haliangium ochraceum DSM 14365 Lineage=Root;rootrank;Bacteria;domain;"Proteobacteria";phylum;Deltaproteobacteria;class;Myxococcales;order;Nannocystineae;suborder;"Haliangiaceae";family;Haliangium;genus	94.4	2.00E-171	603
2369	4	1	2	1	1	2	4	0	0	Proteobacteria	Deltaproteobacteria	Myxococcales	Haliangiaceae		S003611760 Kofleria flava; DSM:14659; 16 Lineage=Root;rootrank;Bacteria;domain;"Proteobacteria";phylum;Deltaproteobacteria;class;Myxococcales;order;Nannocystineae;suborder;Kofleriaceae;family;Kofleria;genus	97.31	2.00E-180	632
1815	0	1	0	0	0	1	0	0	0	Proteobacteria	Deltaproteobacteria	Myxococcales	Haliangiaceae		S003611761 Kofleria flava; DSM:14620; K2 Lineage=Root;rootrank;Bacteria;domain;"Proteobacteria";phylum;Deltaproteobacteria;class;Myxococcales;order;Nannocystineae;suborder;unclassified_Nannocystineae;	96.16	0	640
2593	0	1	0	0	0	0	0	0	0	Proteobacteria	Deltaproteobacteria	Myxococcales	Haliangiaceae		S003611761 Kofleria flava; DSM:14620; K2 Lineage=Root;rootrank;Bacteria;domain;"Proteobacteria";phylum;Deltaproteobacteria;class;Myxococcales;order;Nannocystineae;suborder;unclassified_Nannocystineae;	94.64	4.00E-173	608
1602	0	0	0	0	0	0	0	0	1	Proteobacteria	Deltaproteobacteria	Myxococcales	Myxococcaceae	Anaeromyxobacter	S001188426 Anaeromyxobacter dehalogenans; FRC-D5 Lineage=Root;rootrank;Bacteria;domain;"Proteobacteria";phylum;Deltaproteobacteria;class;Myxococcales;order;Cystobacterineae;suborder;Cystobacteraceae;family;Anaeromyxobacter;genus	96.42	0	645
1711	0	0	0	1	0	0	0	0	0	Proteobacteria	Deltaproteobacteria	Myxococcales	Nannocystaceae	Nannocystis	S000384941 Nannocystis exedens (T); DSM71 Lineage=Root;rootrank;Bacteria;domain;"Proteobacteria";phylum;Deltaproteobacteria;class;Myxococcales;order;Nannocystineae;suborder;Nannocystaceae;family;Nannocystis;genus	94.4	5.00E-162	571
1929	0	1	0	0	0	0	0	0	0	Proteobacteria	Deltaproteobacteria	Myxococcales	Nannocystaceae	Nannocystis	S000539322 Nannocystis aggregans; Na a1 Lineage=Root;rootrank;Bacteria;domain;"Proteobacteria";phylum;Deltaproteobacteria;class;Myxococcales;order;Nannocystineae;suborder;Nannocystaceae;family;Nannocystis;genus	98.47	0	691
692	0	0	3	0	1	1	0	0	0	Proteobacteria	Deltaproteobacteria	Myxococcales	Nannocystaceae	Nannocystis	S002350770 Nannocystis pusilla; type strain: DSM 14622 Lineage=Root;rootrank;Bacteria;domain;"Proteobacteria";phylum;Deltaproteobacteria;class;Myxococcales;order;Nannocystineae;suborder;Nannocystaceae;family;Nannocystis;genus	99.49	0	713
1761	0	0	0	1	0	1	0	1	1	Proteobacteria	Deltaproteobacteria	Myxococcales	Nannocystaceae	Plesiocystis	S002232797 Enhygromyxa salina; SWB004 Lineage=Root;rootrank;Bacteria;domain;"Proteobacteria";phylum;Deltaproteobacteria;class;Myxococcales;order;Nannocystineae;suborder;Nannocystaceae;family;Enhygromyxa;genus	91.18	5.00E-142	505
2610	0	0	0	0	0	0	1	3	0	Proteobacteria	Deltaproteobacteria	Myxococcales	Nannocystaceae	Plesiocystis	S002232797 Enhygromyxa salina; SWB004 Lineage=Root;rootrank;Bacteria;domain;"Proteobacteria";phylum;Deltaproteobacteria;class;Myxococcales;order;Nannocystineae;suborder;Nannocystaceae;family;Enhygromyxa;genus	96.25	4.00E-173	608
322	0	0	1	3	0	0	0	0	1	Proteobacteria	Deltaproteobacteria	Myxococcales	Nannocystaceae	Plesiocystis	S002232798 Enhygromyxa salina; SWB005 Lineage=Root;rootrank;Bacteria;domain;"Proteobacteria";phylum;Deltaproteobacteria;class;Myxococcales;order;Nannocystineae;suborder;Nannocystaceae;family;Enhygromyxa;genus	91.6	1.00E-152	540
543	0	0	0	1	0	0	2	0	0	Proteobacteria	Deltaproteobacteria	Myxococcales	Nannocystaceae	Plesiocystis	S002232798 Enhygromyxa salina; SWB005 Lineage=Root;rootrank;Bacteria;domain;"Proteobacteria";phylum;Deltaproteobacteria;class;Myxococcales;order;Nannocystineae;suborder;Nannocystaceae;family;Enhygromyxa;genus	91.42	4.00E-143	508
1891	0	1	0	0	0	0	1	0	0	Proteobacteria	Deltaproteobacteria	Myxococcales	Nannocystaceae	Plesiocystis	S002232798 Enhygromyxa salina; SWB005 Lineage=Root;rootrank;Bacteria;domain;"Proteobacteria";phylum;Deltaproteobacteria;class;Myxococcales;order;Nannocystineae;suborder;Nannocystaceae;family;Enhygromyxa;genus	98.46	0	688
2568	0	0	0	0	1	0	0	0	0	Proteobacteria	Deltaproteobacteria	Myxococcales	Nannocystaceae		S002232797 Enhygromyxa salina; SWB004 Lineage=Root;rootrank;Bacteria;domain;"Proteobacteria";phylum;Deltaproteobacteria;class;Myxococcales;order;Nannocystineae;suborder;Nannocystaceae;family;Enhygromyxa;genus	91.54	5.00E-152	538
74	0	0	0	0	2	0	0	0	0	Proteobacteria	Deltaproteobacteria	Myxococcales	Nannocystaceae		S002232799 Enhygromyxa salina; SWB006 Lineage=Root;rootrank;Bacteria;domain;"Proteobacteria";phylum;Deltaproteobacteria;class;Myxococcales;order;Nannocystineae;suborder;Nannocystaceae;family;Enhygromyxa;genus	89.84	4.00E-133	475
120	0	0	0	0	1	1	0	3	1	Proteobacteria	Deltaproteobacteria	Myxococcales	Polyangiaceae		S000417333 Byssovorax cruenta (T); type strain: By c2 = DSM 14553 Lineage=Root;rootrank;Bacteria;domain;"Proteobacteria";phylum;Deltaproteobacteria;class;Myxococcales;order;Sorangiineae;suborder;Polyangiaceae;family;Byssovorax;genus	98.39	0	654
381	0	0	0	0	0	0	0	4	1	Proteobacteria	Deltaproteobacteria	Myxococcales	Polyangiaceae		S000417333 Byssovorax cruenta (T); type strain: By c2 = DSM 14553 Lineage=Root;rootrank;Bacteria;domain;"Proteobacteria";phylum;Deltaproteobacteria;class;Myxococcales;order;Sorangiineae;suborder;Polyangiaceae;family;Byssovorax;genus	96.24	1.00E-173	610
2393	0	0	1	0	0	0	0	0	0	Proteobacteria	Deltaproteobacteria	Myxococcales	Polyangiaceae	Sorangium	S001873957 Sorangium cellulosum; KYC3234 Lineage=Root;rootrank;Bacteria;domain;"Proteobacteria";phylum;Deltaproteobacteria;class;Myxococcales;order;Sorangiineae;suborder;Polyangiaceae;family;Sorangium;genus	99.19	0	671
2869	0	0	0	1	0	0	0	0	0	Proteobacteria	Deltaproteobacteria	Myxococcales			S000022588 Haliangium tepidum (T); SMP-10 Lineage=Root;rootrank;Bacteria;domain;"Proteobacteria";phylum;Deltaproteobacteria;class;Myxococcales;order;Nannocystineae;suborder;"Haliangiaceae";family;Haliangium;genus	91.71	8.00E-145	514
159	0	0	0	1	0	0	0	0	0	Proteobacteria	Deltaproteobacteria	Myxococcales			S000392255 Desulfuromonas michiganensis (T); BB1 Lineage=Root;rootrank;Bacteria;domain;"Proteobacteria";phylum;Deltaproteobacteria;class;Desulfuromonadales;order;Desulfuromonadaceae;family;Desulfuromonas;genus	88.72	1.00E-133	477
239	0	0	0	1	0	0	0	0	0	Proteobacteria	Deltaproteobacteria	Myxococcales			S000539289 Archangium gephyra; Ar g1 Lineage=Root;rootrank;Bacteria;domain;"Proteobacteria";phylum;Deltaproteobacteria;class;Myxococcales;order;Cystobacterineae;suborder;Cystobacteraceae;family;unclassified_Cystobacteraceae;	96.16	0	640
2153	0	1	0	21	11	7	5	7	7	Proteobacteria	Deltaproteobacteria	Myxococcales			S000722027 Hyalangium minutum (T); DSM 14724 Lineage=Root;rootrank;Bacteria;domain;"Proteobacteria";phylum;Deltaproteobacteria;class;Myxococcales;order;Cystobacterineae;suborder;Cystobacteraceae;family;Hyalangium;genus	99.73	0	682
2128	0	0	0	1	0	0	0	0	0	Proteobacteria	Deltaproteobacteria	Myxococcales			S000769397 Haliangium ochraceum; GS1 Lineage=Root;rootrank;Bacteria;domain;"Proteobacteria";phylum;Deltaproteobacteria;class;Myxococcales;order;Nannocystineae;suborder;"Haliangiaceae";family;Haliangium;genus	89	3.00E-134	479
2648	0	1	1	1	0	0	0	0	0	Proteobacteria	Deltaproteobacteria	Myxococcales			S000769498 Stigmatella koreensis; KYC-1019 Lineage=Root;rootrank;Bacteria;domain;"Proteobacteria";phylum;Deltaproteobacteria;class;Myxococcales;order;Cystobacterineae;suborder;unclassified_Cystobacterineae;	93.88	4.00E-168	592
1685	0	0	0	0	1	0	1	1	0	Proteobacteria	Deltaproteobacteria	Myxococcales			S000980702 Anaeromyxobacter dehalogenans; R Lineage=Root;rootrank;Bacteria;domain;"Proteobacteria";phylum;Deltaproteobacteria;class;Myxococcales;order;Cystobacterineae;suborder;Cystobacteraceae;family;Anaeromyxobacter;genus	92.41	9.00E-160	564
495	0	0	2	1	5	0	0	0	1	Proteobacteria	Deltaproteobacteria	Myxococcales			S001188426 Anaeromyxobacter dehalogenans; FRC-D5 Lineage=Root;rootrank;Bacteria;domain;"Proteobacteria";phylum;Deltaproteobacteria;class;Myxococcales;order;Cystobacterineae;suborder;Cystobacteraceae;family;Anaeromyxobacter;genus	90.59	4.00E-138	492
1547	0	0	0	0	0	0	2	1	1	Proteobacteria	Deltaproteobacteria	Myxococcales			S001188426 Anaeromyxobacter dehalogenans; FRC-D5 Lineage=Root;rootrank;Bacteria;domain;"Proteobacteria";phylum;Deltaproteobacteria;class;Myxococcales;order;Cystobacterineae;suborder;Cystobacteraceae;family;Anaeromyxobacter;genus	88.27	3.00E-130	466
2952	1	0	0	0	0	0	0	0	1	Proteobacteria	Deltaproteobacteria	Myxococcales			S001188426 Anaeromyxobacter dehalogenans; FRC-D5 Lineage=Root;rootrank;Bacteria;domain;"Proteobacteria";phylum;Deltaproteobacteria;class;Myxococcales;order;Cystobacterineae;suborder;Cystobacteraceae;family;Anaeromyxobacter;genus	88.3	3.00E-130	466
1107	0	0	0	1	1	0	0	0	0	Proteobacteria	Deltaproteobacteria	Myxococcales			S001188435 Anaeromyxobacter dehalogenans; FRC-R8 Lineage=Root;rootrank;Bacteria;domain;"Proteobacteria";phylum;Deltaproteobacteria;class;Myxococcales;order;Cystobacterineae;suborder;Cystobacteraceae;family;Anaeromyxobacter;genus	91.05	3.00E-149	529
428	0	0	0	0	1	1	0	0	0	Proteobacteria	Deltaproteobacteria	Myxococcales			S001188435 Anaeromyxobacter dehalogenans; FRC-R8 Lineage=Root;rootrank;Bacteria;domain;"Proteobacteria";phylum;Deltaproteobacteria;class;Myxococcales;order;Cystobacterineae;suborder;Cystobacteraceae;family;Anaeromyxobacter;genus	94.35	2.00E-161	569
298	0	0	0	1	0	0	0	1	0	Proteobacteria	Deltaproteobacteria	Myxococcales			S002288637 Anaeromyxobacter dehalogenans 2CP-C Lineage=Root;rootrank;Bacteria;domain;"Proteobacteria";phylum;Deltaproteobacteria;class;Myxococcales;order;Cystobacterineae;suborder;Cystobacteraceae;family;Anaeromyxobacter;genus	91.26	8.00E-140	497
398	0	0	1	0	0	0	0	0	0	Proteobacteria	Deltaproteobacteria	Myxococcales			S002288637 Anaeromyxobacter dehalogenans 2CP-C Lineage=Root;rootrank;Bacteria;domain;"Proteobacteria";phylum;Deltaproteobacteria;class;Myxococcales;order;Cystobacterineae;suborder;Cystobacteraceae;family;Anaeromyxobacter;genus	93.62	2.00E-166	586
2915	0	1	0	0	0	0	0	0	0	Proteobacteria	Deltaproteobacteria	Myxococcales			S002288637 Anaeromyxobacter dehalogenans 2CP-C Lineage=Root;rootrank;Bacteria;domain;"Proteobacteria";phylum;Deltaproteobacteria;class;Myxococcales;order;Cystobacterineae;suborder;Cystobacteraceae;family;Anaeromyxobacter;genus	93.37	9.00E-165	580
437	0	0	0	0	0	1	0	0	0	Proteobacteria	Deltaproteobacteria	Myxococcales			S003288466 Angiococcus disciformis; x82 Lineage=Root;rootrank;Bacteria;domain;"Proteobacteria";phylum;Deltaproteobacteria;class;Myxococcales;order;Cystobacterineae;suborder;Myxococcaceae;family;unclassified_Myxococcaceae;	95.4	1.00E-177	623
574	0	2	0	0	0	0	2	0	0	Proteobacteria	Deltaproteobacteria	Myxococcales			S003614644 Pyxidicoccus fallax; S127 Lineage=Root;rootrank;Bacteria;domain;"Proteobacteria";phylum;Deltaproteobacteria;class;Myxococcales;order;Cystobacterineae;suborder;Myxococcaceae;family;unclassified_Myxococcaceae;	92.27	1.00E-148	527
1200	0	0	1	0	0	0	0	0	0	Proteobacteria	Deltaproteobacteria	Myxococcales			S003614644 Pyxidicoccus fallax; S127 Lineage=Root;rootrank;Bacteria;domain;"Proteobacteria";phylum;Deltaproteobacteria;class;Myxococcales;order;Cystobacterineae;suborder;Myxococcaceae;family;unclassified_Myxococcaceae;	92.64	9.00E-160	564
1848	0	0	0	0	0	0	0	0	1	Proteobacteria	Deltaproteobacteria	Myxococcales			S003614644 Pyxidicoccus fallax; S127 Lineage=Root;rootrank;Bacteria;domain;"Proteobacteria";phylum;Deltaproteobacteria;class;Myxococcales;order;Cystobacterineae;suborder;Myxococcaceae;family;unclassified_Myxococcaceae;	93.61	7.00E-166	584
927	0	0	0	0	0	0	2	0	0	Proteobacteria	Deltaproteobacteria	NB-j			S001096520 Phaselicystis flava (T); SBKo001 Lineage=Root;rootrank;Bacteria;domain;"Proteobacteria";phylum;Deltaproteobacteria;class;Myxococcales;order;Sorangiineae;suborder;Phaselicystidaceae;family;Phaselicystis;genus	90.26	1.00E-143	510
1040	0	0	0	1	0	0	0	0	0	Proteobacteria	Deltaproteobacteria	NB-j			S002224489 Chondromyces crocatus; Cm c5 Lineage=Root;rootrank;Bacteria;domain;"Proteobacteria";phylum;Deltaproteobacteria;class;Myxococcales;order;Sorangiineae;suborder;Polyangiaceae;family;Chondromyces;genus	90.84	8.00E-140	497
1386	1	1	1	2	1	2	1	2	1	Proteobacteria	Deltaproteobacteria	NB-j			S002224495 Polyangium sorediatum; Pl s12 Lineage=Root;rootrank;Bacteria;domain;"Proteobacteria";phylum;Deltaproteobacteria;class;Myxococcales;order;Sorangiineae;suborder;Polyangiaceae;family;unclassified_Polyangiaceae;	89.76	1.00E-132	473
2835	0	0	0	2	0	0	0	0	0	Proteobacteria	Deltaproteobacteria	NB-j			S002224495 Polyangium sorediatum; Pl s12 Lineage=Root;rootrank;Bacteria;domain;"Proteobacteria";phylum;Deltaproteobacteria;class;Myxococcales;order;Sorangiineae;suborder;Polyangiaceae;family;unclassified_Polyangiaceae;	89.51	1.00E-138	494
626	0	0	0	2	0	13	10	4	0	Proteobacteria	Deltaproteobacteria	NB-j			S002224578 Jahnella thaxteri; SBSr007 Lineage=Root;rootrank;Bacteria;domain;"Proteobacteria";phylum;Deltaproteobacteria;class;Myxococcales;order;Sorangiineae;suborder;Polyangiaceae;family;Chondromyces;genus	90.84	8.00E-140	497
95	0	0	0	1	0	0	0	0	0	Proteobacteria	Deltaproteobacteria	NB-j			S003288459 Sorangium cellulosum; x3t8 Lineage=Root;rootrank;Bacteria;domain;"Proteobacteria";phylum;Deltaproteobacteria;class;Myxococcales;order;Sorangiineae;suborder;unclassified_Sorangiineae;	89.26	4.00E-138	492
525	0	0	0	1	0	0	0	0	0	Proteobacteria	Deltaproteobacteria	NB-j	MND4		S000626776 Geoalkalibacter ferrihydriticus (T); Z-0531 Lineage=Root;rootrank;Bacteria;domain;"Proteobacteria";phylum;Deltaproteobacteria;class;Desulfuromonadales;order;Geobacteraceae;family;Geoalkalibacter;genus	88.92	3.00E-134	479
761	0	0	0	2	0	0	0	0	0	Proteobacteria	Deltaproteobacteria	NB-j	MND4		S003288459 Sorangium cellulosum; x3t8 Lineage=Root;rootrank;Bacteria;domain;"Proteobacteria";phylum;Deltaproteobacteria;class;Myxococcales;order;Sorangiineae;suborder;unclassified_Sorangiineae;	88.68	1.00E-127	457
1061	0	0	0	0	1	0	0	0	0	Proteobacteria	Deltaproteobacteria	NB-j	MND4		S003288459 Sorangium cellulosum; x3t8 Lineage=Root;rootrank;Bacteria;domain;"Proteobacteria";phylum;Deltaproteobacteria;class;Myxococcales;order;Sorangiineae;suborder;unclassified_Sorangiineae;	87.87	1.00E-122	440
1216	0	0	0	1	0	4	4	1	6	Proteobacteria	Deltaproteobacteria	NB-j	MND4		S003610437 Pelobacter carbinolicus (T); DSM 2380 Lineage=Root;rootrank;Bacteria;domain;"Proteobacteria";phylum;Deltaproteobacteria;class;Desulfuromonadales;order;Desulfuromonadaceae;family;Pelobacter;genus	88.47	2.00E-125	449
1526	0	0	4	10	17	66	56	3	4	Proteobacteria	Deltaproteobacteria	NB-j	NB-i		S002224495 Polyangium sorediatum; Pl s12 Lineage=Root;rootrank;Bacteria;domain;"Proteobacteria";phylum;Deltaproteobacteria;class;Myxococcales;order;Sorangiineae;suborder;Polyangiaceae;family;unclassified_Polyangiaceae;	92.18	4.00E-148	525
1367	0	0	0	2	0	0	1	0	2	Proteobacteria	Deltaproteobacteria	NB-j			S002224495 Polyangium sorediatum; Pl s12 Lineage=Root;rootrank;Bacteria;domain;"Proteobacteria";phylum;Deltaproteobacteria;class;Myxococcales;order;Sorangiineae;suborder;Polyangiaceae;family;unclassified_Polyangiaceae;	90.76	1.00E-137	490
933	0	0	0	1	1	0	0	0	0	Proteobacteria	Deltaproteobacteria	Spirobacillales			S002236156 Desulfomicrobium orale; JCM 17150 Lineage=Root;rootrank;Bacteria;domain;"Proteobacteria";phylum;Deltaproteobacteria;class;Desulfovibrionales;order;Desulfomicrobiaceae;family;Desulfomicrobium;genus	86.29	6.00E-117	422
977	0	0	2	12	4	0	0	0	1	Proteobacteria	Deltaproteobacteria	Syntrophobacterales	Syntrophaceae		S000390568 Thermolithobacter ferrireducens; JW/KA-2; KA2b Lineage=Root;rootrank;Bacteria;domain;Firmicutes;phylum;Thermolithobacteria;class;Thermolithobacterales;order;Thermolithobacteraceae;family;Thermolithobacter;genus	83.24	2.00E-91	337
1970	0	0	0	0	2	3	3	0	0	Proteobacteria	Deltaproteobacteria	Syntrophobacterales	Syntrophaceae		S000900272 Verticillium dahliae; 4S2_F08 Lineage=Root;rootrank;Bacteria;domain;"Acidobacteria";phylum;Acidobacteria_Gp6;class;Gp6;genus	91.92	2.00E-115	416
1620	1	1	42	212	237	575	559	85	142	Proteobacteria	Deltaproteobacteria	Syntrophobacterales	Syntrophobacteraceae		S000009676 Pelobacter acetylenicus (T); WoAcy1 DSM2348 Lineage=Root;rootrank;Bacteria;domain;"Proteobacteria";phylum;Deltaproteobacteria;class;Desulfuromonadales;order;Desulfuromonadaceae;family;Pelobacter;genus	87.84	4.00E-118	425
1204	0	0	0	16	9	28	16	12	17	Proteobacteria	Deltaproteobacteria	Syntrophobacterales	Syntrophobacteraceae		S000009676 Pelobacter acetylenicus (T); WoAcy1 DSM2348 Lineage=Root;rootrank;Bacteria;domain;"Proteobacteria";phylum;Deltaproteobacteria;class;Desulfuromonadales;order;Desulfuromonadaceae;family;Pelobacter;genus	89.97	1.00E-132	473
742	0	0	1	4	12	38	25	6	1	Proteobacteria	Deltaproteobacteria	Syntrophobacterales	Syntrophobacteraceae		S000009676 Pelobacter acetylenicus (T); WoAcy1 DSM2348 Lineage=Root;rootrank;Bacteria;domain;"Proteobacteria";phylum;Deltaproteobacteria;class;Desulfuromonadales;order;Desulfuromonadaceae;family;Pelobacter;genus	88.03	5.00E-122	438
3101	0	0	0	3	1	16	13	2	2	Proteobacteria	Deltaproteobacteria	Syntrophobacterales	Syntrophobacteraceae		S000009676 Pelobacter acetylenicus (T); WoAcy1 DSM2348 Lineage=Root;rootrank;Bacteria;domain;"Proteobacteria";phylum;Deltaproteobacteria;class;Desulfuromonadales;order;Desulfuromonadaceae;family;Pelobacter;genus	90.3	3.00E-134	479
1327	0	0	0	0	2	6	1	0	2	Proteobacteria	Deltaproteobacteria	Syntrophobacterales	Syntrophobacteraceae		S000009676 Pelobacter acetylenicus (T); WoAcy1 DSM2348 Lineage=Root;rootrank;Bacteria;domain;"Proteobacteria";phylum;Deltaproteobacteria;class;Desulfuromonadales;order;Desulfuromonadaceae;family;Pelobacter;genus	89.78	1.00E-133	477
2019	0	0	0	0	0	1	1	0	0	Proteobacteria	Deltaproteobacteria	Syntrophobacterales	Syntrophobacteraceae		S000009676 Pelobacter acetylenicus (T); WoAcy1 DSM2348 Lineage=Root;rootrank;Bacteria;domain;"Proteobacteria";phylum;Deltaproteobacteria;class;Desulfuromonadales;order;Desulfuromonadaceae;family;Pelobacter;genus	91.22	2.00E-141	503
1019	0	0	0	0	0	0	0	2	0	Proteobacteria	Deltaproteobacteria	Syntrophobacterales	Syntrophobacteraceae		S000009676 Pelobacter acetylenicus (T); WoAcy1 DSM2348 Lineage=Root;rootrank;Bacteria;domain;"Proteobacteria";phylum;Deltaproteobacteria;class;Desulfuromonadales;order;Desulfuromonadaceae;family;Pelobacter;genus	87.17	5.00E-117	422
260	0	0	0	1	0	0	0	0	0	Proteobacteria	Deltaproteobacteria	Syntrophobacterales	Syntrophobacteraceae		S000009676 Pelobacter acetylenicus (T); WoAcy1 DSM2348 Lineage=Root;rootrank;Bacteria;domain;"Proteobacteria";phylum;Deltaproteobacteria;class;Desulfuromonadales;order;Desulfuromonadaceae;family;Pelobacter;genus	91.18	5.00E-142	505
509	0	0	0	0	0	3	7	2	30	Proteobacteria	Deltaproteobacteria	Syntrophobacterales	Syntrophobacteraceae		S000392255 Desulfuromonas michiganensis (T); BB1 Lineage=Root;rootrank;Bacteria;domain;"Proteobacteria";phylum;Deltaproteobacteria;class;Desulfuromonadales;order;Desulfuromonadaceae;family;Desulfuromonas;genus	88.3	4.00E-123	442
2995	0	0	0	0	1	3	2	3	8	Proteobacteria	Deltaproteobacteria	Syntrophobacterales	Syntrophobacteraceae		S000392836 Desulfacinum subterraneum; 101 Lineage=Root;rootrank;Bacteria;domain;"Proteobacteria";phylum;Deltaproteobacteria;class;Syntrophobacterales;order;Syntrophobacteraceae;family;Desulfacinum;genus	90.35	2.00E-136	486
822	0	0	0	0	1	0	0	0	0	Proteobacteria	Deltaproteobacteria	Syntrophobacterales	Syntrophobacteraceae		S000392836 Desulfacinum subterraneum; 101 Lineage=Root;rootrank;Bacteria;domain;"Proteobacteria";phylum;Deltaproteobacteria;class;Syntrophobacterales;order;Syntrophobacteraceae;family;Desulfacinum;genus	92.25	3.00E-149	529
815	0	0	0	0	0	0	0	1	0	Proteobacteria	Deltaproteobacteria	Syntrophobacterales	Syntrophobacteraceae		S000592818 Desulfomonile tiedjei (T); DSM 6799 Lineage=Root;rootrank;Bacteria;domain;"Proteobacteria";phylum;Deltaproteobacteria;class;Syntrophobacterales;order;Syntrophaceae;family;Desulfomonile;genus	91.33	7.00E-151	534
2121	0	0	0	2	3	4	3	0	7	Proteobacteria	Deltaproteobacteria	Syntrophobacterales	Syntrophobacteraceae		S000650984 Desulfoglaeba alkanexedens (T); ALDC Lineage=Root;rootrank;Bacteria;domain;"Proteobacteria";phylum;Deltaproteobacteria;class;Syntrophobacterales;order;Syntrophobacteraceae;family;Desulfoglaeba;genus	87.84	9.00E-120	431
1166	0	0	0	1	0	0	0	0	0	Proteobacteria	Deltaproteobacteria	Syntrophobacterales	Syntrophobacteraceae		S002350132 Desulfobulbus propionicus DSM 2032 Lineage=Root;rootrank;Bacteria;domain;"Proteobacteria";phylum;Deltaproteobacteria;class;Desulfobacterales;order;Desulfobulbaceae;family;Desulfobulbus;genus	86.67	3.00E-114	412
2223	0	0	0	0	1	0	0	0	0	Proteobacteria	Deltaproteobacteria	Syntrophobacterales	Syntrophobacteraceae		S002953916 Clostridium sardiniense; 235 Lineage=Root;rootrank;Bacteria;domain;Firmicutes;phylum;Clostridia;class;Clostridiales;order;Clostridiaceae 1;family;Clostridium sensu stricto;genus	86.52	1.00E-112	407
2465	0	0	0	0	0	1	0	1	1	Proteobacteria	Deltaproteobacteria	Syntrophobacterales	Syntrophobacteraceae		S003287266 Desulfomonile tiedjei DSM 6799 Lineage=Root;rootrank;Bacteria;domain;"Proteobacteria";phylum;Deltaproteobacteria;class;Syntrophobacterales;order;Syntrophaceae;family;Desulfomonile;genus	91.71	2.00E-145	516
2332	0	0	0	14	0	6	0	2	0	Proteobacteria	Deltaproteobacteria	Syntrophobacterales	Syntrophobacteraceae		S003610437 Pelobacter carbinolicus (T); DSM 2380 Lineage=Root;rootrank;Bacteria;domain;"Proteobacteria";phylum;Deltaproteobacteria;class;Desulfuromonadales;order;Desulfuromonadaceae;family;Pelobacter;genus	89.49	3.00E-129	462
2137	0	0	1	4	4	5	6	0	2	Proteobacteria	Deltaproteobacteria	Syntrophobacterales	Syntrophobacteraceae		S003610437 Pelobacter carbinolicus (T); DSM 2380 Lineage=Root;rootrank;Bacteria;domain;"Proteobacteria";phylum;Deltaproteobacteria;class;Desulfuromonadales;order;Desulfuromonadaceae;family;Pelobacter;genus	91.18	5.00E-142	505
1043	0	0	0	3	1	0	0	0	1	Proteobacteria	Deltaproteobacteria	Syntrophobacterales	Syntrophobacteraceae		S003610437 Pelobacter carbinolicus (T); DSM 2380 Lineage=Root;rootrank;Bacteria;domain;"Proteobacteria";phylum;Deltaproteobacteria;class;Desulfuromonadales;order;Desulfuromonadaceae;family;Pelobacter;genus	90.59	4.00E-138	492
2906	0	0	0	3	0	0	0	0	0	Proteobacteria	Deltaproteobacteria	Syntrophobacterales	Syntrophobacteraceae		S003610437 Pelobacter carbinolicus (T); DSM 2380 Lineage=Root;rootrank;Bacteria;domain;"Proteobacteria";phylum;Deltaproteobacteria;class;Desulfuromonadales;order;Desulfuromonadaceae;family;Pelobacter;genus	90.61	7.00E-146	518
2208	0	0	0	0	1	1	0	0	1	Proteobacteria	Deltaproteobacteria	Syntrophobacterales	Syntrophobacteraceae		S003610437 Pelobacter carbinolicus (T); DSM 2380 Lineage=Root;rootrank;Bacteria;domain;"Proteobacteria";phylum;Deltaproteobacteria;class;Desulfuromonadales;order;Desulfuromonadaceae;family;Pelobacter;genus	88.98	4.00E-128	459
713	0	1	0	0	2	1	0	0	0	Proteobacteria	Deltaproteobacteria	Syntrophobacterales			S000387293 Smithella propionica (T); LYP Lineage=Root;rootrank;Bacteria;domain;"Proteobacteria";phylum;Deltaproteobacteria;class;Syntrophobacterales;order;Syntrophaceae;family;Smithella;genus	93.55	5.00E-157	555
446	0	0	0	0	0	0	0	0	1	Proteobacteria	Deltaproteobacteria	Syntrophobacterales			S000460057 Sphaerobacter thermophilus; 185-3 Lineage=Root;rootrank;Bacteria;domain;unclassified_Bacteria;	91.64	1.00E-128	460
612	0	0	0	0	0	0	0	0	1	Proteobacteria	Deltaproteobacteria				S000005623 Polyangium cellulosum; So ce26 Lineage=Root;rootrank;Bacteria;domain;"Proteobacteria";phylum;Deltaproteobacteria;class;Myxococcales;order;Sorangiineae;suborder;Polyangiaceae;family;Sorangium;genus	84.99	1.00E-108	394
134	0	0	1	2	2	0	0	0	1	Proteobacteria	Deltaproteobacteria				S000009676 Pelobacter acetylenicus (T); WoAcy1 DSM2348 Lineage=Root;rootrank;Bacteria;domain;"Proteobacteria";phylum;Deltaproteobacteria;class;Desulfuromonadales;order;Desulfuromonadaceae;family;Pelobacter;genus	90.33	3.00E-144	512
2910	0	0	0	1	0	0	0	0	0	Proteobacteria	Deltaproteobacteria				S000009676 Pelobacter acetylenicus (T); WoAcy1 DSM2348 Lineage=Root;rootrank;Bacteria;domain;"Proteobacteria";phylum;Deltaproteobacteria;class;Desulfuromonadales;order;Desulfuromonadaceae;family;Pelobacter;genus	89.52	2.00E-131	470
2831	0	0	0	0	0	3	0	0	0	Proteobacteria	Deltaproteobacteria				S000022588 Haliangium tepidum (T); SMP-10 Lineage=Root;rootrank;Bacteria;domain;"Proteobacteria";phylum;Deltaproteobacteria;class;Myxococcales;order;Nannocystineae;suborder;"Haliangiaceae";family;Haliangium;genus	85.86	3.00E-114	412
1278	0	0	0	1	0	0	0	0	0	Proteobacteria	Deltaproteobacteria				S000425634 Geobacter hephaestius; VeEG4 Lineage=Root;rootrank;Bacteria;domain;"Proteobacteria";phylum;Deltaproteobacteria;class;Desulfuromonadales;order;Geobacteraceae;family;Geobacter;genus	86.17	7.00E-111	401
1270	0	0	0	0	1	2	0	0	1	Proteobacteria	Deltaproteobacteria				S000434673 Thermovenabulum ferriorganovorum (T); Z-9801 Lineage=Root;rootrank;Bacteria;domain;Firmicutes;phylum;Clostridia;class;Clostridiales;order;Clostridiales_Incertae Sedis III;family;Thermovenabulum;genus	86.45	3.00E-120	433
1855	0	0	0	1	0	2	1	4	0	Proteobacteria	Deltaproteobacteria				S000437417 Desulfuromonas palmitatis (T); SDBY1 Lineage=Root;rootrank;Bacteria;domain;"Proteobacteria";phylum;Deltaproteobacteria;class;Desulfuromonadales;order;Desulfuromonadaceae;family;Desulfuromonas;genus	92.18	1.00E-148	527
23	0	0	0	2	0	2	0	0	0	Proteobacteria	Deltaproteobacteria				S000437417 Desulfuromonas palmitatis (T); SDBY1 Lineage=Root;rootrank;Bacteria;domain;"Proteobacteria";phylum;Deltaproteobacteria;class;Desulfuromonadales;order;Desulfuromonadaceae;family;Desulfuromonas;genus	89.23	5.00E-137	488
2084	0	0	1	0	0	0	0	0	0	Proteobacteria	Deltaproteobacteria				S000437417 Desulfuromonas palmitatis (T); SDBY1 Lineage=Root;rootrank;Bacteria;domain;"Proteobacteria";phylum;Deltaproteobacteria;class;Desulfuromonadales;order;Desulfuromonadaceae;family;Desulfuromonas;genus	90.03	8.00E-135	481
174	0	0	0	0	1	0	0	0	1	Proteobacteria	Deltaproteobacteria				S000437601 Geothrix fermentans (T); H5 Lineage=Root;rootrank;Bacteria;domain;"Acidobacteria";phylum;Holophagae;class;Holophagales;order;Holophagaceae;family;Geothrix;genus	84.65	6.00E-107	388
236	0	3	0	0	1	0	0	7	2	Proteobacteria	Deltaproteobacteria				S000556968 Desulfosalsimonas propionicica (T); PropA Lineage=Root;rootrank;Bacteria;domain;"Proteobacteria";phylum;Deltaproteobacteria;class;Desulfobacterales;order;Desulfobacteraceae;family;Desulfosalsimonas;genus	84.45	9.00E-100	364
72	0	0	0	2	0	0	0	0	0	Proteobacteria	Deltaproteobacteria				S000626776 Geoalkalibacter ferrihydriticus (T); Z-0531 Lineage=Root;rootrank;Bacteria;domain;"Proteobacteria";phylum;Deltaproteobacteria;class;Desulfuromonadales;order;Geobacteraceae;family;Geoalkalibacter;genus	91.64	8.00E-145	514
238	0	0	0	1	0	0	0	0	0	Proteobacteria	Deltaproteobacteria				S000769397 Haliangium ochraceum; GS1 Lineage=Root;rootrank;Bacteria;domain;"Proteobacteria";phylum;Deltaproteobacteria;class;Myxococcales;order;Nannocystineae;suborder;"Haliangiaceae";family;Haliangium;genus	88.74	5.00E-127	455
222	1	4	0	0	0	0	0	0	0	Proteobacteria	Deltaproteobacteria				S000871061 Pelobacter seleniigenes (T); KM Lineage=Root;rootrank;Bacteria;domain;"Proteobacteria";phylum;Deltaproteobacteria;class;Desulfuromonadales;order;Desulfuromonadaceae;family;Pelobacter;genus	85.03	1.00E-103	377
2050	1	0	0	2	1	0	0	1	0	Proteobacteria	Deltaproteobacteria				S001188426 Anaeromyxobacter dehalogenans; FRC-D5 Lineage=Root;rootrank;Bacteria;domain;"Proteobacteria";phylum;Deltaproteobacteria;class;Myxococcales;order;Cystobacterineae;suborder;Cystobacteraceae;family;Anaeromyxobacter;genus	92.74	5.00E-152	538
2416	0	0	2	7	8	1	1	4	1	Proteobacteria	Deltaproteobacteria				S001188435 Anaeromyxobacter dehalogenans; FRC-R8 Lineage=Root;rootrank;Bacteria;domain;"Proteobacteria";phylum;Deltaproteobacteria;class;Myxococcales;order;Cystobacterineae;suborder;Cystobacteraceae;family;Anaeromyxobacter;genus	90.28	3.00E-144	512
2791	0	0	0	1	0	0	0	0	0	Proteobacteria	Deltaproteobacteria				S001188435 Anaeromyxobacter dehalogenans; FRC-R8 Lineage=Root;rootrank;Bacteria;domain;"Proteobacteria";phylum;Deltaproteobacteria;class;Myxococcales;order;Cystobacterineae;suborder;Cystobacteraceae;family;Anaeromyxobacter;genus	89.81	4.00E-133	475
1519	0	1	0	0	0	0	0	0	0	Proteobacteria	Deltaproteobacteria				S001241100 Chondromyces lanuginosus; KYC2904 Lineage=Root;rootrank;Bacteria;domain;"Proteobacteria";phylum;Deltaproteobacteria;class;Myxococcales;order;Sorangiineae;suborder;Polyangiaceae;family;Chondromyces;genus	89.52	5.00E-132	472
1418	2	0	0	0	0	0	1	0	0	Proteobacteria	Deltaproteobacteria				S002288029 Syntrophus aciditrophicus (T); SB Lineage=Root;rootrank;Bacteria;domain;"Proteobacteria";phylum;Deltaproteobacteria;class;Syntrophobacterales;order;Syntrophaceae;family;Syntrophus;genus	84.64	2.00E-100	366
603	0	0	0	0	0	4	2	0	1	Proteobacteria	Deltaproteobacteria				S002288637 Anaeromyxobacter dehalogenans 2CP-C Lineage=Root;rootrank;Bacteria;domain;"Proteobacteria";phylum;Deltaproteobacteria;class;Myxococcales;order;Cystobacterineae;suborder;Cystobacteraceae;family;Anaeromyxobacter;genus	92.74	5.00E-152	538
1722	0	0	0	2	0	0	0	1	0	Proteobacteria	Deltaproteobacteria				S002288637 Anaeromyxobacter dehalogenans 2CP-C Lineage=Root;rootrank;Bacteria;domain;"Proteobacteria";phylum;Deltaproteobacteria;class;Myxococcales;order;Cystobacterineae;suborder;Cystobacteraceae;family;Anaeromyxobacter;genus	90.08	6.00E-136	484
1345	0	0	0	0	0	0	0	0	1	Proteobacteria	Deltaproteobacteria				S002290737 Pelobacter propionicus (T); DSM 2379 Lineage=Root;rootrank;Bacteria;domain;"Proteobacteria";phylum;Deltaproteobacteria;class;Desulfuromonadales;order;Desulfuromonadaceae;family;Pelobacter;genus	91.08	2.00E-140	499
807	0	0	0	1	0	0	0	0	0	Proteobacteria	Deltaproteobacteria				S002964296 Geobacter lovleyi; Geo7.3C Lineage=Root;rootrank;Bacteria;domain;"Proteobacteria";phylum;Deltaproteobacteria;class;Desulfuromonadales;order;Geobacteraceae;family;Geobacter;genus	91.13	6.00E-141	501
1250	0	0	0	1	0	0	0	0	0	Proteobacteria	Deltaproteobacteria				S003263888 Sneathiella glossodoripedis; NBRC 103391 Lineage=Root;rootrank;Bacteria;domain;"Proteobacteria";phylum;Alphaproteobacteria;class;Sneathiellales;order;Sneathiellaceae;family;Sneathiella;genus	85.75	1.00E-113	411
2039	0	0	0	0	0	0	0	0	1	Proteobacteria	Deltaproteobacteria				S003263891 Sneathiella chinensis; NBRC 103408 Lineage=Root;rootrank;Bacteria;domain;"Proteobacteria";phylum;Alphaproteobacteria;class;Sneathiellales;order;Sneathiellaceae;family;Sneathiella;genus	88.5	8.00E-125	448

767	0	0	0	2	0	0	0	0	0	Proteobacteria	Deltaproteobacteria				S003610437 Pelobacter carbinolicus (T); DSM 2380 Lineage=Root;rootrank;Bacteria;domain;"Proteobacteria";phylum;Deltaproteobacteria;class;Desulfuromonadales;order;Desulfuromonadaceae;family;Pelobacter;genus	93.28	2.00E-155	549
1365	0	0	0	0	0	0	1	0	0	Proteobacteria	Deltaproteobacteria				S003610438 Geobacter metallireducens GS-15 Lineage=Root;rootrank;Bacteria;domain;"Proteobacteria";phylum;Deltaproteobacteria;class;Desulfuromonadales;order;Geobacteraceae;family;Geobacter;genus	90.54	5.00E-137	488
1705	0	0	0	0	0	0	0	38	67	Proteobacteria	Gammaproteobacteria			Kangiella	S000654145 Thiohalophilus thiocyanatoxydans (T); HRhD 2 Lineage=Root;rootrank;Bacteria;domain;"Proteobacteria";phylum;Gammaproteobacteria;class;Gammaproteobacteria_incertae_sedis;order;Thiohalophilus;genus	91.18	2.00E-141	503
1747	0	0	0	0	0	0	0	2	0	Proteobacteria	Gammaproteobacteria			Kangiella	S000722236 Gilvimarinus chinensis (T); QM42 Lineage=Root;rootrank;Bacteria;domain;"Proteobacteria";phylum;Gammaproteobacteria;class;Gammaproteobacteria_incertae_sedis;order;Gilvimarinus;genus	90.61	7.00E-146	518
3238	0	0	0	0	0	0	0	2	12	Proteobacteria	Gammaproteobacteria	Alteromonadales	Idiomarinaceae	Idiomarina	S003714431 Idiomarina loihiensis; LAMA 691 Lineage=Root;rootrank;Bacteria;domain;"Proteobacteria";phylum;Gammaproteobacteria;class;Alteromonadales;order;Idiomarinaceae;family;Idiomarina;genus	100	0	686
2255	0	0	0	0	0	0	0	1	1	Proteobacteria	Gammaproteobacteria	Alteromonadales	Idiomarinaceae	Pseudidiomarina	S000843599 Pseudidiomarina salinarum (T); ISL-52 Lineage=Root;rootrank;Bacteria;domain;"Proteobacteria";phylum;Gammaproteobacteria;class;Alteromonadales;order;Idiomarinaceae;family;Pseudidiomarina;genus	98.21	0	684
922	0	0	0	0	0	1	0	0	0	Proteobacteria	Gammaproteobacteria	Chromatiales			S000654145 Thiohalophilus thiocyanatoxydans (T); HRhD 2 Lineage=Root;rootrank;Bacteria;domain;"Proteobacteria";phylum;Gammaproteobacteria;class;Gammaproteobacteria_incertae_sedis;order;Thiohalophilus;genus	93.53	2.00E-155	549
2533	0	0	34	980	823	84	50	1	2	Proteobacteria	Gammaproteobacteria	Chromatiales			S001198079 Thioprofundum lithotrophicum (T); 106 Lineage=Root;rootrank;Bacteria;domain;"Proteobacteria";phylum;Gammaproteobacteria;class;Gammaproteobacteria_incertae_sedis;order;Thioprofundum;genus	94.13	2.00E-160	566
360	0	0	2	14	7	2	1	2	7	Proteobacteria	Gammaproteobacteria	Chromatiales			S001198079 Thioprofundum lithotrophicum (T); 106 Lineage=Root;rootrank;Bacteria;domain;"Proteobacteria";phylum;Gammaproteobacteria;class;Gammaproteobacteria_incertae_sedis;order;Thioprofundum;genus	93.3	2.00E-155	549
2589	0	0	0	3	0	3	9	2	13	Proteobacteria	Gammaproteobacteria	Chromatiales			S001793637 Thiohalobacter thiocyanaticus (T); HRh1 Lineage=Root;rootrank;Bacteria;domain;"Proteobacteria";phylum;Gammaproteobacteria;class;Gammaproteobacteria_incertae_sedis;order;Thiohalobacter;genus	90.81	1.00E-138	494
27	0	0	0	0	0	0	0	40	1	Proteobacteria	Gammaproteobacteria	Chromatiales			S002032707 Loripes lacteus gill symbiont; 2C Lineage=Root;rootrank;Bacteria;domain;"Proteobacteria";phylum;Gammaproteobacteria;class;Gammaproteobacteria_incertae_sedis;order;Sedimenticola;genus	94.1	2.00E-160	566
3154	0	0	0	0	0	0	0	0	1	Proteobacteria	Gammaproteobacteria	Chromatiales			S002916742 Methylococcus capsulatus; CIc Lineage=Root;rootrank;Bacteria;domain;"Proteobacteria";phylum;Gammaproteobacteria;class;Methylococcales;order;Methylococcaceae;family;Methylococcus;genus	93.82	4.00E-158	558
9	0	0	0	3	0	0	0	0	0	Proteobacteria	Gammaproteobacteria	Chromatiales			S002973546 Acidiferrobacter thiooxydans; type strain: DSM 2392 Lineage=Root;rootrank;Bacteria;domain;"Proteobacteria";phylum;Gammaproteobacteria;class;unclassified_Gammaproteobacteria;	90.32	2.00E-136	486
1583	3	79	2	4	3	3	2	11	18	Proteobacteria	Gammaproteobacteria	Chromatiales			S000891953 Methylohalomonas lacus (T); HMT 1 Lineage=Root;rootrank;Bacteria;domain;"Proteobacteria";phylum;Gammaproteobacteria;class;Gammaproteobacteria_incertae_sedis;order;Methylohalomonas;genus	96.51	6.00E-176	617
2075	0	0	0	0	1	0	0	0	0	Proteobacteria	Gammaproteobacteria	Chromatiales			S000891953 Methylohalomonas lacus (T); HMT 1 Lineage=Root;rootrank;Bacteria;domain;"Proteobacteria";phylum;Gammaproteobacteria;class;Gammaproteobacteria_incertae_sedis;order;Methylohalomonas;genus	93.89	4.00E-168	592
2435	1	18	8	0	1	13	14	14	0	Proteobacteria	Gammaproteobacteria	Chromatiales	Chromatiaceae	Nitrosococcus	S002290401 Nitrosococcus halophilus Nc4 Lineage=Root;rootrank;Bacteria;domain;"Proteobacteria";phylum;Gammaproteobacteria;class;Chromatiales;order;Chromatiaceae;family;Nitrosococcus;genus	94.35	2.00E-161	569
2701	1	4	0	0	0	0	0	0	0	Proteobacteria	Gammaproteobacteria	Chromatiales	Chromatiaceae	Nitrosococcus	S002290453 Nitrosococcus watsoni C-113 Lineage=Root;rootrank;Bacteria;domain;"Proteobacteria";phylum;Gammaproteobacteria;class;Chromatiales;order;Chromatiaceae;family;Nitrosococcus;genus	94.07	8.00E-160	564
1267	0	0	0	1	0	2	1	0	0	Proteobacteria	Gammaproteobacteria	Chromatiales	Chromatiaceae	Nitrosococcus	S002290453 Nitrosococcus watsoni C-113 Lineage=Root;rootrank;Bacteria;domain;"Proteobacteria";phylum;Gammaproteobacteria;class;Chromatiales;order;Chromatiaceae;family;Nitrosococcus;genus	95.42	4.00E-168	592
2338	0	0	0	0	0	1	0	0	0	Proteobacteria	Gammaproteobacteria	Chromatiales	Ectothiorhodospiraceae		S000543472 Alviniconcha hessleri gill endosymbiont Lineage=Root;rootrank;Bacteria;domain;"Proteobacteria";phylum;Gammaproteobacteria;class;Gammaproteobacteria_incertae_sedis;order;Thioprofundum;genus	93.28	2.00E-155	549
1185	0	0	1	0	0	0	0	0	1	Proteobacteria	Gammaproteobacteria	Chromatiales	Ectothiorhodospiraceae		S000893775 Methylonatrum kenyense; AMT 3 Lineage=Root;rootrank;Bacteria;domain;"Proteobacteria";phylum;Gammaproteobacteria;class;Gammaproteobacteria_incertae_sedis;order;Methylonatrum;genus	94.62	1.00E-163	577
2740	65	372	24	0	2	26	55	13	10	Proteobacteria	Gammaproteobacteria	Chromatiales	Ectothiorhodospiraceae		S000942429 sulfur-oxidizing symbiont of Inandrilus exumae Lineage=Root;rootrank;Bacteria;domain;"Proteobacteria";phylum;Gammaproteobacteria;class;unclassified_Gammaproteobacteria;	90.59	1.00E-137	490
1536	1	2	0	1	1	2	0	227	186	Proteobacteria	Gammaproteobacteria	Chromatiales	Ectothiorhodospiraceae		S000942429 sulfur-oxidizing symbiont of Inandrilus exumae Lineage=Root;rootrank;Bacteria;domain;"Proteobacteria";phylum;Gammaproteobacteria;class;unclassified_Gammaproteobacteria;	90.86	3.00E-139	496
2864	22	152	10	0	0	27	43	2	0	Proteobacteria	Gammaproteobacteria	Chromatiales	Ectothiorhodospiraceae		S000942429 sulfur-oxidizing symbiont of Inandrilus exumae Lineage=Root;rootrank;Bacteria;domain;"Proteobacteria";phylum;Gammaproteobacteria;class;unclassified_Gammaproteobacteria;	90.32	6.00E-136	484
2392	3	0	0	0	0	0	0	0	0	Proteobacteria	Gammaproteobacteria	Chromatiales	Ectothiorhodospiraceae		S000942429 sulfur-oxidizing symbiont of Inandrilus exumae Lineage=Root;rootrank;Bacteria;domain;"Proteobacteria";phylum;Gammaproteobacteria;class;unclassified_Gammaproteobacteria;	90.35	2.00E-136	486
229	5	8	6	6	0	8	9	9	2	Proteobacteria	Gammaproteobacteria	Chromatiales	Ectothiorhodospiraceae		S001793637 Thiohalobacter thiocyanaticus (T); HRh1 Lineage=Root;rootrank;Bacteria;domain;"Proteobacteria";phylum;Gammaproteobacteria;class;Gammaproteobacteria_incertae_sedis;order;Thiohalobacter;genus	94.09	2.00E-160	566
1555	0	0	0	1	0	0	0	8	0	Proteobacteria	Gammaproteobacteria	Chromatiales	Ectothiorhodospiraceae		S000893775 Methylonatrum kenyense; AMT 3 Lineage=Root;rootrank;Bacteria;domain;"Proteobacteria";phylum;Gammaproteobacteria;class;Gammaproteobacteria_incertae_sedis;order;Methylonatrum;genus	92.37	1.00E-157	556
1369	0	0	1	4	6	4	5	1	3	Proteobacteria	Gammaproteobacteria	Chromatiales	Sinobacteraceae		S000843124 Thiohalomonas denitrificans; HLD 15 Lineage=Root;rootrank;Bacteria;domain;"Proteobacteria";phylum;Gammaproteobacteria;class;Gammaproteobacteria_incertae_sedis;order;Thiohalomonas;genus	91.08	2.00E-140	499
1673	0	0	9	50	54	19	17	32	27	Proteobacteria	Gammaproteobacteria	Chromatiales	Sinobacteraceae		S000995323 Steroidobacter denitrificans (T); FS Lineage=Root;rootrank;Bacteria;domain;"Proteobacteria";phylum;Gammaproteobacteria;class;Xanthomonadales;order;Sinobacteraceae;family;Steroidobacter;genus	89.52	2.00E-131	470
750	0	0	0	32	7	32	17	5	1	Proteobacteria	Gammaproteobacteria	Chromatiales	Sinobacteraceae		S000995323 Steroidobacter denitrificans (T); FS Lineage=Root;rootrank;Bacteria;domain;"Proteobacteria";phylum;Gammaproteobacteria;class;Xanthomonadales;order;Sinobacteraceae;family;Steroidobacter;genus	90.11	8.00E-135	481
1306	0	0	6	6	5	10	5	1	1	Proteobacteria	Gammaproteobacteria	Chromatiales	Sinobacteraceae		S000995323 Steroidobacter denitrificans (T); FS Lineage=Root;rootrank;Bacteria;domain;"Proteobacteria";phylum;Gammaproteobacteria;class;Xanthomonadales;order;Sinobacteraceae;family;Steroidobacter;genus	91.37	5.00E-152	538
566	0	0	0	9	9	2	1	0	0	Proteobacteria	Gammaproteobacteria	Chromatiales	Sinobacteraceae		S000995323 Steroidobacter denitrificans (T); FS Lineage=Root;rootrank;Bacteria;domain;"Proteobacteria";phylum;Gammaproteobacteria;class;Xanthomonadales;order;Sinobacteraceae;family;Steroidobacter;genus	91.05	1.00E-148	527
940	0	0	0	0	1	0	1	1	0	Proteobacteria	Gammaproteobacteria	Chromatiales	Sinobacteraceae		S000995323 Steroidobacter denitrificans (T); FS Lineage=Root;rootrank;Bacteria;domain;"Proteobacteria";phylum;Gammaproteobacteria;class;Xanthomonadales;order;Sinobacteraceae;family;Steroidobacter;genus	89.49	1.00E-137	490
1844	0	0	1	1	0	0	0	0	0	Proteobacteria	Gammaproteobacteria	Chromatiales	Sinobacteraceae		S000995323 Steroidobacter denitrificans (T); FS Lineage=Root;rootrank;Bacteria;domain;"Proteobacteria";phylum;Gammaproteobacteria;class;Xanthomonadales;order;Sinobacteraceae;family;Steroidobacter;genus	89.51	3.00E-139	496
2635	0	0	0	1	0	0	0	0	0	Proteobacteria	Gammaproteobacteria	Chromatiales	Sinobacteraceae		S000995323 Steroidobacter denitrificans (T); FS Lineage=Root;rootrank;Bacteria;domain;"Proteobacteria";phylum;Gammaproteobacteria;class;Xanthomonadales;order;Sinobacteraceae;family;Steroidobacter;genus	92.47	8.00E-150	531
1728	0	0	0	0	0	0	0	1	0	Proteobacteria	Gammaproteobacteria	Chromatiales	Sinobacteraceae		S000995323 Steroidobacter denitrificans (T); FS Lineage=Root;rootrank;Bacteria;domain;"Proteobacteria";phylum;Gammaproteobacteria;class;Xanthomonadales;order;Sinobacteraceae;family;Steroidobacter;genus	95.17	5.00E-167	588
1055	37	38	5	1	5	16	12	1	3	Proteobacteria	Gammaproteobacteria	Chromatiales	Sinobacteraceae		S001153278 Methylogaea oryzae JCM 16910; E10 Lineage=Root;rootrank;Bacteria;domain;"Proteobacteria";phylum;Gammaproteobacteria;class;Methylococcales;order;Methylococcaceae;family;Methylococcus;genus	91.08	8.00E-140	497
550	387	1136	690	623	907	568	764	813	581	Proteobacteria	Gammaproteobacteria	Chromatiales	Sinobacteraceae		S001198079 Thioprofundum lithotrophicum (T); 106 Lineage=Root;rootrank;Bacteria;domain;"Proteobacteria";phylum;Gammaproteobacteria;class;Gammaproteobacteria_incertae_sedis;order;Thioprofundum;genus	92.25	5.00E-147	521
1755	0	0	0	0	1	0	0	0	0	Proteobacteria	Gammaproteobacteria	Chromatiales	Sinobacteraceae		S002290453 Nitrosococcus watsoni C-113 Lineage=Root;rootrank;Bacteria;domain;"Proteobacteria";phylum;Gammaproteobacteria;class;Chromatiales;order;Chromatiaceae;family;Nitrosococcus;genus	92.18	1.00E-147	523
2899	0	0	0	0	1	0	0	0	0	Proteobacteria	Gammaproteobacteria	Chromatiales	Sinobacteraceae		S002290453 Nitrosococcus watsoni C-113 Lineage=Root;rootrank;Bacteria;domain;"Proteobacteria";phylum;Gammaproteobacteria;class;Chromatiales;order;Chromatiaceae;family;Nitrosococcus;genus	90.57	1.00E-137	490
249	0	0	0	1	0	0	1	0	0	Proteobacteria	Gammaproteobacteria	Chromatiales	Sinobacteraceae		S002441980 Candidatus Thiomargarita joergensenii; NAM034 Lineage=Root;rootrank;Bacteria;domain;"Proteobacteria";phylum;Gammaproteobacteria;class;unclassified_Gammaproteobacteria;	89.54	1.00E-138	494
1266	0	0	0	10	13	3	2	2	0	Proteobacteria	Gammaproteobacteria	Chromatiales	Sinobacteraceae	Steroidobacter	S000995323 Steroidobacter denitrificans (T); FS Lineage=Root;rootrank;Bacteria;domain;"Proteobacteria";phylum;Gammaproteobacteria;class;Xanthomonadales;order;Sinobacteraceae;family;Steroidobacter;genus	98.92	0	664
2252	0	0	0	0	3	0	0	0	1	Proteobacteria	Gammaproteobacteria	Chromatiales	Sinobacteraceae		S000995323 Steroidobacter denitrificans (T); FS Lineage=Root;rootrank;Bacteria;domain;"Proteobacteria";phylum;Gammaproteobacteria;class;Xanthomonadales;order;Sinobacteraceae;family;Steroidobacter;genus	95.44	3.00E-179	628
2668	0	0	0	0	0	1	0	0	0	Proteobacteria	Gammaproteobacteria	Chromatiales	Sinobacteraceae		S000995323 Steroidobacter denitrificans (T); FS Lineage=Root;rootrank;Bacteria;domain;"Proteobacteria";phylum;Gammaproteobacteria;class;Xanthomonadales;order;Sinobacteraceae;family;Steroidobacter;genus	94.62	4.00E-163	575
2212	0	0	0	0	1	0	0	0	0	Proteobacteria	Gammaproteobacteria	Chromatiales	Sinobacteraceae		S002290453 Nitrosococcus watsoni C-113 Lineage=Root;rootrank;Bacteria;domain;"Proteobacteria";phylum;Gammaproteobacteria;class;Chromatiales;order;Chromatiaceae;family;Nitrosococcus;genus	85.14	5.00E-102	372
1890	0	2	3	3	5	8	17	4	15	Proteobacteria	Gammaproteobacteria	Chromatiales			S000015324 Codakia orbicularis gill symbiont Lineage=Root;rootrank;Bacteria;domain;"Proteobacteria";phylum;Gammaproteobacteria;class;Gammaproteobacteria_incertae_sedis;order;unclassified_Gammaproteobacteria_incertae_sedis;	92.49	2.00E-150	532
2306	0	0	0	3	0	9	5	5	5	Proteobacteria	Gammaproteobacteria	Chromatiales			S000414253 Thyasira flexuosa gill symbiont Lineage=Root;rootrank;Bacteria;domain;"Proteobacteria";phylum;Gammaproteobacteria;class;Gammaproteobacteria_incertae_sedis;order;Sedimenticola;genus	91.6	1.00E-142	507
2033	0	0	0	0	0	0	0	0	2	Proteobacteria	Gammaproteobacteria	Chromatiales			S000654145 Thiohalophilus thiocyanatoxydans (T); HRhD 2 Lineage=Root;rootrank;Bacteria;domain;"Proteobacteria";phylum;Gammaproteobacteria;class;Gammaproteobacteria_incertae_sedis;order;Thiohalophilus;genus	90.32	8.00E-135	481
160	0	2	8	0	4	9	16	2	8	Proteobacteria	Gammaproteobacteria	Chromatiales			S000769575 Thiohalomonas denitrificans; HLD 14 Lineage=Root;rootrank;Bacteria;domain;"Proteobacteria";phylum;Gammaproteobacteria;class;Gammaproteobacteria_incertae_sedis;order;Thiohalomonas;genus	90.82	1.00E-147	523
416	0	0	0	0	0	0	0	4	6	Proteobacteria	Gammaproteobacteria	Chromatiales			S000769575 Thiohalomonas denitrificans; HLD 14 Lineage=Root;rootrank;Bacteria;domain;"Proteobacteria";phylum;Gammaproteobacteria;class;Gammaproteobacteria_incertae_sedis;order;Thiohalomonas;genus	94.4	7.00E-171	601
331	0	3	0	0	0	0	0	0	0	Proteobacteria	Gammaproteobacteria	Chromatiales			S000769575 Thiohalomonas denitrificans; HLD 14 Lineage=Root;rootrank;Bacteria;domain;"Proteobacteria";phylum;Gammaproteobacteria;class;Gammaproteobacteria_incertae_sedis;order;Thiohalomonas;genus	89.46	2.00E-130	466
1370	0	0	1	6	2	1	2	1	2	Proteobacteria	Gammaproteobacteria	Chromatiales			S000843124 Thiohalomonas denitrificans; HLD 15 Lineage=Root;rootrank;Bacteria;domain;"Proteobacteria";phylum;Gammaproteobacteria;class;Gammaproteobacteria_incertae_sedis;order;Thiohalomonas;genus	90.88	1.00E-138	494
3123	0	0	0	1	0	0	0	0	0	Proteobacteria	Gammaproteobacteria	Chromatiales			S000891930 Methylonatrum kenyense (T); AMT 1 Lineage=Root;rootrank;Bacteria;domain;"Proteobacteria";phylum;Gammaproteobacteria;class;Gammaproteobacteria_incertae_sedis;order;Methylonatrum;genus	92.99	5.00E-152	538
1473	0	0	0	0	0	0	0	1	0	Proteobacteria	Gammaproteobacteria	Chromatiales			S000891953 Methylohalomonas lacus (T); HMT 1 Lineage=Root;rootrank;Bacteria;domain;"Proteobacteria";phylum;Gammaproteobacteria;class;Gammaproteobacteria_incertae_sedis;order;Methylohalomonas;genus	88.2	5.00E-122	438
211	0	7	0	0	1	0	1	3	3	Proteobacteria	Gammaproteobacteria	Chromatiales			S000893775 Methylonatrum kenyense; AMT 3 Lineage=Root;rootrank;Bacteria;domain;"Proteobacteria";phylum;Gammaproteobacteria;class;Gammaproteobacteria_incertae_sedis;order;Methylonatrum;genus	93.58	2.00E-156	553
1158	0	1	0	0	0	0	0	4	4	Proteobacteria	Gammaproteobacteria	Chromatiales			S000893775 Methylonatrum kenyense; AMT 3 Lineage=Root;rootrank;Bacteria;domain;"Proteobacteria";phylum;Gammaproteobacteria;class;Gammaproteobacteria_incertae_sedis;order;Methylonatrum;genus	94.1	2.00E-160	566
1774	0	0	0	0	3	0	0	110	77	Proteobacteria	Gammaproteobacteria	Chromatiales			S000942429 sulfur-oxidizing symbiont of Inandrilus exumae Lineage=Root;rootrank;Bacteria;domain;"Proteobacteria";phylum;Gammaproteobacteria;class;unclassified_Gammaproteobacteria;	92.2	1.00E-148	527
2729	0	0	0	0	0	0	0	0	1	Proteobacteria	Gammaproteobacteria	Chromatiales			S000942429 sulfur-oxidizing symbiont of Inandrilus exumae Lineage=Root;rootrank;Bacteria;domain;"Proteobacteria";phylum;Gammaproteobacteria;class;unclassified_Gammaproteobacteria;	86.23	4.00E-108	392
2623	0	1	0	0	0	0	0	0	0	Proteobacteria	Gammaproteobacteria	Chromatiales			S001198079 Thioprofundum lithotrophicum (T); 106 Lineage=Root;rootrank;Bacteria;domain;"Proteobacteria";phylum;Gammaproteobacteria;class;Gammaproteobacteria_incertae_sedis;order;Thioprofundum;genus	93.64	2.00E-166	586
701	0	0	0	0	0	0	0	1	0	Proteobacteria	Gammaproteobacteria	Chromatiales			S001198079 Thioprofundum lithotrophicum (T); 106 Lineage=Root;rootrank;Bacteria;domain;"Proteobacteria";phylum;Gammaproteobacteria;class;Gammaproteobacteria_incertae_sedis;order;Thioprofundum;genus	92.39	5.00E-157	555
1586	2	3	3	1	3	2	0	41	73	Proteobacteria	Gammaproteobacteria	Chromatiales			S001793637 Thiohalobacter thiocyanaticus (T); HRh1 Lineage=Root;rootrank;Bacteria;domain;"Proteobacteria";phylum;Gammaproteobacteria;class;Gammaproteobacteria_incertae_sedis;order;Thiohalobacter;genus	92.74	2.00E-151	536
2603	16	1	4	26	20	6	4	0	0	Proteobacteria	Gammaproteobacteria	Chromatiales			S002290453 Nitrosococcus watsoni C-113 Lineage=Root;rootrank;Bacteria;domain;"Proteobacteria";phylum;Gammaproteobacteria;class;Chromatiales;order;Chromatiaceae;family;Nitrosococcus;genus	91.56	5.00E-152	538
2106	0	0	1	0	0	0	0	0	0	Proteobacteria	Gammaproteobacteria	Chromatiales			S002290453 Nitrosococcus watsoni C-113 Lineage=Root;rootrank;Bacteria;domain;"Proteobacteria";phylum;Gammaproteobacteria;class;Chromatiales;order;Chromatiaceae;family;Nitrosococcus;genus	85.48	2.00E-106	387
372	0	0	0	0	0	0	0	0	1	Proteobacteria	Gammaproteobacteria	Chromatiales			S002290453 Nitrosococcus watsoni C-113 Lineage=Root;rootrank;Bacteria;domain;"Proteobacteria";phylum;Gammaproteobacteria;class;Chromatiales;order;Chromatiaceae;family;Nitrosococcus;genus	93.01	4.00E-153	542
1237	0	3	0	13	0	6	3	2	7	Proteobacteria	Gammaproteobacteria	Chromatiales			S003258418 endosymbiont of Sclerolinum contortum; 1099 Lineage=Root;rootrank;Bacteria;domain;"Proteobacteria";phylum;Gammaproteobacteria;class;Gammaproteobacteria_incertae_sedis;order;Sedimenticola;genus	92.16	5.00E-147	521
497	3	2	1	2	0	0	0	0	0	Proteobacteria	Gammaproteobacteria	Enterobacteriales	Enterobacteriaceae	Escherichia	S003303227 Escherichia coli; CUSMBN2 Lineage=Root;rootrank;Bacteria;domain;"Proteobacteria";phylum;Gammaproteobacteria;class;"Enterobacteriales";order;Enterobacteriaceae;family;Escherichia/Shigella;genus	99.73	0	682
42	0	1	0	1	0	0	0	1	0	Proteobacteria	Gammaproteobacteria	Enterobacteriales	Enterobacteriaceae		S003751236 Escherichia hermannii; h-10 Lineage=Root;rootrank;Bacteria;domain;"Proteobacteria";phylum;Gammaproteobacteria;class;"Enterobacteriales";order;Enterobacteriaceae;family;unclassified_Enterobacteriaceae;	99.49	0	710
3254	0	0	0	4	0	0	0	0	0	Proteobacteria	Gammaproteobacteria	Legionellales	Coxiellaceae		S000008928 Pseudomonas oleovorans (T); IAM 1508 Lineage=Root;rootrank;Bacteria;domain;"Proteobacteria";phylum;Gammaproteobacteria;class;Pseudomonadales;order;Pseudomonadaceae;family;Pseudomonas;genus	90.72	7.00E-146	518
34	0	0	1	0	0	0	0	0	0	Proteobacteria	Gammaproteobacteria	Legionellales	Coxiellaceae		S000323019 symbiont of Haemaphysalis concinnae Lineage=Root;rootrank;Bacteria;domain;"Proteobacteria";phylum;Gammaproteobacteria;class;Legionellales;order;Coxiellaceae;family;Coxiella;genus	89.49	6.00E-131	468
65	0	0	0	1	0	0	0	0	0	Proteobacteria	Gammaproteobacteria	Legionellales	Coxiellaceae		S000425966 Legionella-like amoebal pathogen HT99 Lineage=Root;rootrank;Bacteria;domain;"Proteobacteria";phylum;Gammaproteobacteria;class;unclassified_Gammaproteobacteria;	89.81	4.00E-133	475
1274	0	0	0	1	0	0	0	0	0	Proteobacteria	Gammaproteobacteria	Legionellales	Coxiellaceae		S000425966 Legionella-like amoebal pathogen HT99 Lineage=Root;rootrank;Bacteria;domain;"Proteobacteria";phylum;Gammaproteobacteria;class;unclassified_Gammaproteobacteria;	87.4	4.00E-118	425
751	0	0	0	1	0	0	1	0	1	Proteobacteria	Gammaproteobacteria	Legionellales	Coxiellaceae		S000824755 Coxiella cheraxi; TO-98 Lineage=Root;rootrank;Bacteria;domain;"Proteobacteria";phylum;Gammaproteobacteria;class;Legionellales;order;Coxiellaceae;family;Coxiella;genus	91.15	2.00E-141	503
2034	0	0	0	0	0	1	0	0	1	Proteobacteria	Gammaproteobacteria	Legionellales	Coxiellaceae		S000824755 Coxiella cheraxi; TO-98 Lineage=Root;rootrank;Bacteria;domain;"Proteobacteria";phylum;Gammaproteobacteria;class;Legionellales;order;Coxiellaceae;family;Coxiella;genus	92.35	2.00E-146	520
2391	0	0	0	1	0	0	0	0	0	Proteobacteria	Gammaproteobacteria	Legionellales	Coxiellaceae		S000824755 Coxiella cheraxi; TO-98 Lineage=Root;rootrank;Bacteria;domain;"Proteobacteria";phylum;Gammaproteobacteria;class;Legionellales;order;Coxiellaceae;family;Coxiella;genus	90.98	4.00E-138	492
3054	0	0	0	0	0	0	0	1	0	Proteobacteria	Gammaproteobacteria	Legionellales	Coxiellaceae		S000891236 Proteinimicrobium ihbtica; type strain: HMPB3 Lineage=Root;rootrank;Bacteria;domain;"Proteobacteria";phylum;Betaproteobacteria;class;unclassified_Betaproteobacteria;	91	4.00E-148	525
1091	0	0	0	3	1	1	0	2	0	Proteobacteria	Gammaproteobacteria	Legionellales	Coxiellaceae		S002291134 Coxiella burnetii RSA 331 Lineage=Root;rootrank;Bacteria;domain;"Proteobacteria";phylum;Gammaproteobacteria;class;Legionellales;order;Coxiellaceae;family;Coxiella;genus	91.64	3.00E-144	512
1585	0	0	1	1	2	0	2	11	3	Proteobacteria	Gammaproteobacteria	Legionellales	Coxiellaceae	Aquicella	S000408383 Aquicella siphonis (T); SGT-108 Lineage=Root;rootrank;Bacteria;domain;"Proteobacteria";phylum;Gammaproteobacteria;class;Legionellales;order;Coxiellaceae;family;Aquicella;genus	97.03	1.00E-177	623
2342	0	0	0	0	0	2	3	6	7	Proteobacteria	Gammaproteobacteria	Legionellales	Coxiellaceae	Aquicella	S000408383 Aquicella siphonis (T); SGT-108 Lineage=Root;rootrank;Bacteria;domain;"Proteobacteria";phylum;Gammaproteobacteria;class;Legionellales;order;Coxiellaceae;family;Aquicella;genus	94.59	1.00E-162	573
2645	0	0	0	1	4	0	0	0	0	Proteobacteria	Gammaproteobacteria	Legionellales	Coxiellaceae	Aquicella	S000408383 Aquicella siphonis (T); SGT-108 Lineage=Root;rootrank;Bacteria;domain;"Proteobacteria";phylum;Gammaproteobacteria;class;Legionellales;order;Coxiellaceae;family;Aquicella;genus	95.43	4.00E-168	592
271	0	0	0	0	1	1	0	0	0	Proteobacteria	Gammaproteobacteria	Legionellales	Coxiellaceae	Aquicella	S000408383 Aquicella siphonis (T); SGT-108 Lineage=Root;rootrank;Bacteria;domain;"Proteobacteria";phylum;Gammaproteobacteria;class;Legionellales;order;Coxiellaceae;family;Aquicella;genus	93.09	2.00E-161	569
1693	0	0	1	0	0	0	0	1	0	Proteobacteria	Gammaproteobacteria	Legionellales	Coxiellaceae	Aquicella	S000408383 Aquicella siphonis (T); SGT-108 Lineage=Root;rootrank;Bacteria;domain;"Proteobacteria";phylum;Gammaproteobacteria;class;Legionellales;order;Coxiellaceae;family;Aquicella;genus	95.42	1.00E-167	590
2299	0	0	0	0	0	0	0	0	1	Proteobacteria	Gammaproteobacteria	Legionellales	Coxiellaceae	Coxiella	S000560316 Haemaphysalis longicornis symbiont A Lineage=Root;rootrank;Bacteria;domain;"Proteobacteria";phylum;Gammaproteobacteria;class;Legionellales;order;Coxiellaceae;family;Coxiella;genus	94.32	4.00E-168	592
201	0	0	0	1	0	0	0	0	0	Proteobacteria	Gammaproteobacteria	Legionellales	Coxiellaceae	Coxiella	S002291134 Coxiella burnetii RSA 331 Lineage=Root;rootrank;Bacteria;domain;"Proteobacteria";phylum;Gammaproteobacteria;class;Legionellales;order;Coxiellaceae;family;Coxiella;genus	93.83	7.00E-166	584
289	0	1	0	0	0	0	0	0	0	Proteobacteria	Gammaproteobacteria	Legionellales	Coxiellaceae	Rickettsiella	S003612063 Rickettsiella costelytrae; JKI ANZ2008-1 Lineage=Root;rootrank;Bacteria;domain;"Proteobacteria";phylum;Gammaproteobacteria;class;Legionellales;order;Coxiellaceae;family;Coxiella;genus	99.48	0	691
876	0	0	1	0	0	0	0	0	0	Proteobacteria	Gammaproteobacteria	Legionellales	Coxiellaceae		S000824755 Coxiella cheraxi; TO-98 Lineage=Root;rootrank;Bacteria;domain;"Proteobacteria";phylum;Gammaproteobacteria;class;Legionellales;order;Coxiellaceae;family;Coxiella;genus	93.85	5.00E-167	588
947	1	1	0	0	0	1	1	0	0	Proteobacteria	Gammaproteobacteria	Legionellales	Coxiellaceae		S000859363 Legionella-like amoebal pathogen CC99 Lineage=Root;rootrank;Bacteria;domain;"Proteobacteria";phylum;Gammaproteobacteria;class;unclassified_Gammaproteobacteria;	85.68	5.00E-107	388
1529	0	0	0	0	0	0	0	0	1	Proteobacteria	Gammaproteobacteria	Legionellales	Coxiellaceae		S000942792 Methylomicrobium agile; ATCC 35068 Lineage=Root;rootrank;Bacteria;domain;"Proteobacteria";phylum;Gammaproteobacteria;class;Methylococcales;order;Methylococcaceae;family;Methylosarcina;genus	86.6	1.00E-112	407
915	0	0	1	0	0	0	0	1	0	Proteobacteria	Gammaproteobacteria	Legionellales	Coxiellaceae		S002290340 Coxiella burnetii CbuK_Q154 Lineage=Root;rootrank;Bacteria;domain;"Proteobacteria";phylum;Gammaproteobacteria;class;Legionellales;order;Coxiellaceae;family;Coxiella;genus	93.61	3.00E-164	579
2363	0	0	0	0	1	0	1	0	0	Proteobacteria	Gammaproteobacteria	Legionellales	Coxiellaceae		S002290401 Nitrosococcus halophilus Nc4 Lineage=Root;rootrank;Bacteria;domain;"Proteobacteria";phylum;Gammaproteobacteria;class;Chromatiales;order;Chromatiaceae;family;Nitrosococcus;genus	86.79	4.00E-113	409
2800	2	1	3	1	0	0	0	0	1	Proteobacteria	Gammaproteobacteria	Legionellales	Coxiellaceae		S002291134 Coxiella burnetii RSA 331 Lineage=Root;rootrank;Bacteria;domain;"Proteobacteria";phylum;Gammaproteobacteria;class;Legionellales;order;Coxiellaceae;family;Coxiella;genus	95.42	1.00E-167	590
1516	1	0	1	0	0	0	0	0	0	Proteobacteria	Gammaproteobacteria	Legionellales	Coxiellaceae		S002291134 Coxiella burnetii RSA 331 Lineage=Root;rootrank;Bacteria;domain;"Proteobacteria";phylum;Gammaproteobacteria;class;Legionellales;order;Coxiellaceae;family;Coxiella;genus	92.56	1.00E-157	556
3088	0	0	0	1	1	0	0	0	0	Proteobacteria	Gammaproteobacteria	Legionellales	Legionellaceae	Legionella	S001152221 Candidatus Legionella jeonii Lineage=Root;rootrank;Bacteria;domain;"Proteobacteria";phylum;Gammaproteobacteria;class;Legionellales;order;Legionellaceae;family;Legionella;genus	94.62	4.00E-163	575
633	0	0	0	0	0	0	0	0	1	Proteobacteria	Gammaproteobacteria	Legionellales	Legionellaceae	Legionella	S001152221 Candidatus Legionella jeonii Lineage=Root;rootrank;Bacteria;domain;"Proteobacteria";phylum;Gammaproteobacteria;class;Legionellales;order;Legionellaceae;family;Legionella;genus	96.77	2.00E-176	619
1406	0	0	1	0	0	0	0	0	0	Proteobacteria	Gammaproteobacteria	Legionellales	Legionellaceae	Tatlockia	S000841839 Legionella gresilensis (T); Greoux 11 D13; ATCC 700509 Lineage=Root;rootrank;Bacteria;domain;"Proteobacteria";phylum;Gammaproteobacteria;class;Legionellales;order;Legionellaceae;family;Legionella;genus	94.62	1.00E-163	577
2667	0	0	0	0	0	1	0	0	0	Proteobacteria	Gammaproteobacteria	Legionellales	Legionellaceae	Tatlockia	S003745347 Legionella oakridgensis; 4232 Lineage=Root;rootrank;Bacteria;domain;"Proteobacteria";phylum;Gammaproteobacteria;class;Legionellales;order;Legionellaceae;family;Legionella;genus	96.79	5.00E-177	621
741	0	0	0	0	0	0	0	0	2	Proteobacteria	Gammaproteobacteria	Legionellales	Legionellaceae		S000591193 Legionella impletisoli (T); OA1-1 Lineage=Root;rootrank;Bacteria;domain;"Proteobacteria";phylum;Gammaproteobacteria;class;Legionellales;order;Legionellaceae;family;Legionella;genus	93.62	7.00E-166	584
2031	0	0	0	0	0	0	0	2	1	Proteobacteria	Gammaproteobacteria	Legionellales	Legionellaceae		S002442301 Legionella londiniensis; 36_HC3_2008 Lineage=Root;rootrank;Bacteria;domain;"Proteobacteria";phylum;Gammaproteobacteria;class;Legionellales;order;Legionellaceae;family;Legionella;genus	97.85	0	643
2945	0	0	0	0	0	0	0	0	1	Proteobacteria	Gammaproteobacteria	Legionellales			S000591196 Legionella yabuuchiae; OA1-4 Lineage=Root;rootrank;Bacteria;domain;"Proteobacteria";phylum;Gammaproteobacteria;class;Legionellales;order;Legionellaceae;family;Legionella;genus	95.43	1.00E-168	593
1310	0	1	0	0	0	0	0	0	0	Proteobacteria	Gammaproteobacteria	Methylococcales	Methylococcaceae	Methylocaldum	S000438737 Methylocaldum gracile (T); VKM-14L Lineage=Root;rootrank;Bacteria;domain;"Proteobacteria";phylum;Gammaproteobacteria;class;Methylococcales;order;Methylococcaceae;family;Methylocaldum;genus	99.46	0	676
2253	0	0	0	1	0	0	2	0	0	Proteobacteria	Gammaproteobacteria	Methylococcales			S000007118 Methylobacter luteus (T); NCIMB 11914 Lineage=Root;rootrank;Bacteria;domain;"Proteobacteria";phylum;Gammaproteobacteria;class;Methylococcales;order;Methylococcaceae;family;Methylobacter;genus	98.92	0	665
2665	0	0	0	1	0	1	0	0	0	Proteobacteria	Gammaproteobacteria	Oceanospirillales	25ds0		S001743992 Haliea mediterranea (T); type strain: 7SM29 = CECT 7447 Lineage=Root;rootrank;Bacteria;domain;"Proteobacteria";phylum;Gammaproteobacteria;class;Alteromonadales;order;Alteromonadaceae;family;Haliea;genus	92.2	1.00E-147	523
2683	0	0	0	1	0	0	0	0	0	Proteobacteria	Gammaproteobacteria	Oceanospirillales	2ds20		S002355070 Halioglobus pacificus; S1-72 Lineage=Root;rootrank;Bacteria;domain;"Proteobacteria";phylum;Gammaproteobacteria;class;Alteromonadales;order;Alteromonadaceae;family;Haliea;genus	94.09	8.00E-160	564
3115	0	1	0	1	0	0	0	2	1	Proteobacteria	Gammaproteobacteria	Oceanospirillales	Alcanivoracaceae	Alcanivorax	S000624183 endosymbiont of Bugula pacifica; WB Lineage=Root;rootrank;Bacteria;domain;"Proteobacteria";phylum;Gammaproteobacteria;class;Gammaproteobacteria_incertae_sedis;order;Eionea;genus	93.33	2.00E-161	569
1857	0	1	0	1	0	0	0	1	10	Proteobacteria	Gammaproteobacteria	Oceanospirillales	Alteromonadaceae	Cellvibrio	S002291281 Cellvibrio japonicus Ueda107 Lineage=Root;rootrank;Bacteria;domain;"Proteobacteria";phylum;Gammaproteobacteria;class;Pseudomonadales;order;Pseudomonadaceae;family;Cellvibrio;genus	98.11	0	647
2914	0	0	0	0	1	0	0	53	76	Proteobacteria	Gammaproteobacteria	Oceanospirillales	Alteromonadaceae	Marinimicrobium	S001155661 Marinimicrobium locisalis (T); ISL-43 Lineage=Root;rootrank;Bacteria;domain;"Proteobacteria";phylum;Gammaproteobacteria;class;Alteromonadales;order;Alteromonadaceae;family;Marinimicrobium;genus	100	0	686
1187	0	0	0	0	0	0	0	6	9	Proteobacteria	Gammaproteobacteria	Oceanospirillales	Alteromonadaceae	Marinobacter	S000843598 Marinobacter salicampi (T); ISL-40 Lineage=Root;rootrank;Bacteria;domain;"Proteobacteria";phylum;Gammaproteobacteria;class;Alteromonadales;order;Alteromonadaceae;family;Marinobacter;genus	99.19	0	669
339	0	0	1	1	0	1	1	163	270	Proteobacteria	Gammaproteobacteria	Oceanospirillales	Alteromonadaceae	Marinobacter	S003716714 Marinobacter guineae; S3-3-1 Lineage=Root;rootrank;Bacteria;domain;"Proteobacteria";phylum;Gammaproteobacteria;class;Alteromonadales;order;Alteromonadaceae;family;Marinobacter;genus	100	0	686
2812	0	0	1	0	0	0	0	0	0	Proteobacteria	Gammaproteobacteria	Oceanospirillales	Alteromonadaceae	Microbulbifer	S001551220 Microbulbifer agarilyticus; BLCJ 1 Lineage=Root;rootrank;Bacteria;domain;"Proteobacteria";phylum;Gammaproteobacteria;class;Alteromonadales;order;Alteromonadaceae;family;Microbulbifer;genus	97.57	0	636
91	0	0	0	0	0	0	1	3	0	Proteobacteria	Gammaproteobacteria	Oceanospirillales	Alteromonadaceae	Microbulbifer	S001743155 Microbulbifer chitinilyticus; ABABA 212 Lineage=Root;rootrank;Bacteria;domain;"Proteobacteria";phylum;Gammaproteobacteria;class;Alteromonadales;order;Alteromonadaceae;family;Microbulbifer;genus	98.21	0	682
2473	3	0	0	0	0	0	0	0	0	Proteobacteria	Gammaproteobacteria	Oceanospirillales	Alteromonadaceae	ND37	S001613516 Marinimicrobium haloxylanilyticum; SX15 Lineage=Root;rootrank;Bacteria;domain;"Proteobacteria";phylum;Gammaproteobacteria;class;unclassified_Gammaproteobacteria;	98.38	0	652
2888	0	15	3	1	0	5	5	21	51	Proteobacteria	Gammaproteobacteria	Oceanospirillales	Alteromonadaceae	Teredinibacter	S000017407 unclassified Pseudomonadales Lineage=Root;rootrank;Bacteria;domain;"Proteobacteria";phylum;Gammaproteobacteria;class;Alteromonadales;order;Alteromonadales_incertae_sedis;family;Teredinibacter;genus	95.42	4.00E-168	592
419	0	8	2	0	0	4	4	3	0	Proteobacteria	Gammaproteobacteria	Oceanospirillales	Alteromonadaceae	Teredinibacter	S001098252 Teredinibacter turnerae; T0609 Lineage=Root;rootrank;Bacteria;domain;"Proteobacteria";phylum;Gammaproteobacteria;class;Alteromonadales;order;Alteromonadales_incertae_sedis;family;Teredinibacter;genus	94.61	4.00E-163	575
672	1	0	2	4	3	0	6	28	132	Proteobacteria	Gammaproteobacteria	Oceanospirillales	Alteromonadaceae	Teredinibacter	S002411224 Marinimicrobium koreense; LS-A18 Lineage=Root;rootrank;Bacteria;domain;"Proteobacteria";phylum;Gammaproteobacteria;class;Alteromonadales;order;Alteromonadaceae;family;Marinimicrobium;genus	95.42	4.00E-168	592
754	0	0	0	0	0	0	0	7	34	Proteobacteria	Gammaproteobacteria	Oceanospirillales	Alteromonadaceae	Teredinibacter	S002411224 Marinimicrobium koreense; LS-A18 Lineage=Root;rootrank;Bacteria;domain;"Proteobacteria";phylum;Gammaproteobacteria;class;Alteromonadales;order;Alteromonadaceae;family;Marinimicrobium;genus	94.1	8.00E-160	564
93	0	0	4	0	0	0	0	28	48	Proteobacteria	Gammaproteobacteria	Oceanospirillales	Alteromonadaceae	Teredinibacter	S003313680 Saccharophagus degradans; HME8281 Lineage=Root;rootrank;Bacteria;domain;"Proteobacteria";phylum;Gammaproteobacteria;class;Alteromonadales;order;Alteromonadaceae;family;Saccharophagus;genus	95.15	2.00E-166	586
1337	0	0	0	0	0	0	0	84	316	Proteobacteria	Gammaproteobacteria	Oceanospirillales	Alteromonadaceae		S001612980 Gilvimarinus agarilyticus; M5c Lineage=Root;rootrank;Bacteria;domain;"Proteobacteria";phylum;Gammaproteobacteria;class;Gammaproteobacteria_incertae_sedis;order;Gilvimarinus;genus	97.04	4.00E-178	625
1637	0	0	0	0	0	0	0	0	1	Proteobacteria	Gammaproteobacteria	Oceanospirillales	Alteromonadaceae		S002916001 Porticoccus hydrocarbonoclasticus; MCTG13d Lineage=Root;rootrank;Bacteria;domain;"Proteobacteria";phylum;Gammaproteobacteria;class;Gammaproteobacteria_incertae_sedis;order;Porticoccus;genus	96.25	4.00E-173	608
1494	0	0	0	1	0	0	0	1	0	Proteobacteria	Gammaproteobacteria	Oceanospirillales	Halomonadaceae	Candidatus Portiera	S000570234 Lactovum miscens (T); anNAG3 Lineage=Root;rootrank;Bacteria;domain;Firmicutes;phylum;Bacilli;class;Lactobacillales;order;Streptococcaceae;family;Lactovum;genus	80.86	3.00E-49	196
2527	1	9	50	89	42	10	16	576	712	Proteobacteria	Gammaproteobacteria	Oceanospirillales	Halomonadaceae	Halomonas	S000903111 Pseudomonas duriflava (T); HR2 Lineage=Root;rootrank;Bacteria;domain;"Proteobacteria";phylum;Gammaproteobacteria;class;Pseudomonadales;order;Pseudomonadaceae;family;Pseudomonas;genus	91.62	3.00E-144	512
32	25	74	10	0	6	17	35	4	0	Proteobacteria	Gammaproteobacteria	Oceanospirillales	HTCC2089		S000427629 obligately oligotrophic bacteria KI89C Lineage=Root;rootrank;Bacteria;domain;"Proteobacteria";phylum;Gammaproteobacteria;class;unclassified_Gammaproteobacteria;	92.76	2.00E-151	536
3056	9	12	5	12	7	18	10	13	15	Proteobacteria	Gammaproteobacteria	Oceanospirillales	HTCC2089		S000427629 obligately oligotrophic bacteria KI89C Lineage=Root;rootrank;Bacteria;domain;"Proteobacteria";phylum;Gammaproteobacteria;class;unclassified_Gammaproteobacteria;	95.38	2.00E-176	619
1432	3	10	0	0	0	1	3	0	0	Proteobacteria	Gammaproteobacteria	Oceanospirillales	HTCC2089		S000427629 obligately oligotrophic bacteria KI89C Lineage=Root;rootrank;Bacteria;domain;"Proteobacteria";phylum;Gammaproteobacteria;class;unclassified_Gammaproteobacteria;	93.57	5.00E-157	555
1374	0	0	1	1	6	14	4	0	1	Proteobacteria	Gammaproteobacteria	Oceanospirillales	HTCC2089		S000624182 endosymbiont of Bugula turbinata; DP Lineage=Root;rootrank;Bacteria;domain;"Proteobacteria";phylum;Gammaproteobacteria;class;Gammaproteobacteria_incertae_sedis;order;Eionea;genus	92.18	4.00E-148	525
699	2	5	2	0	0	2	2	1	0	Proteobacteria	Gammaproteobacteria	Oceanospirillales	HTCC2089		S003284834 Acinetobacter radioresistens; g111 Lineage=Root;rootrank;Bacteria;domain;"Proteobacteria";phylum;Gammaproteobacteria;class;unclassified_Gammaproteobacteria;	95.91	0	634
1878	0	0	0	1	0	0	0	0	0	Proteobacteria	Gammaproteobacteria	Oceanospirillales	HTCC2089		S003284834 Acinetobacter radioresistens; g111 Lineage=Root;rootrank;Bacteria;domain;"Proteobacteria";phylum;Gammaproteobacteria;class;unclassified_Gammaproteobacteria;	92.45	8.00E-150	531
1482	0	0	0	0	1	0	0	0	1	Proteobacteria	Gammaproteobacteria	Oceanospirillales	HTCC288		S002355067 Halioglobus japonicus; S1-36 Lineage=Root;rootrank;Bacteria;domain;"Proteobacteria";phylum;Gammaproteobacteria;class;Alteromonadales;order;Alteromonadaceae;family;Haliea;genus	91.13	2.00E-141	503

2758	0	0	0	0	0	0	0	0	2	Proteobacteria	Gammaproteobacteria	Oceanospirillales	HTCC288	HTCC	S001743992 Haliea mediterranea (T); type strain: 7SM29 = CECT 7447 Lineage=Root;rootrank;Bacteria;domain;"Proteobacteria";phylum;Gammaproteobacteria;class;Alteromonadales;order;Alteromonadaceae;family;Haliea;genus	90.64	1.00E-138	494
1159	0	0	0	2	3	0	0	0	0	Proteobacteria	Gammaproteobacteria	Oceanospirillales	HTCC288		S000805352 Pseudomonas putida; PM1 Lineage=Root;rootrank;Bacteria;domain;"Proteobacteria";phylum;Gammaproteobacteria;class;Pseudomonadales;order;Pseudomonadaceae;family;unclassified_Pseudomonadaceae;	91.64	3.00E-144	512
2748	0	0	0	1	1	0	0	5	35	Proteobacteria	Gammaproteobacteria	Oceanospirillales	HTCC288		S002355070 Halioglobus pacificus; S1-72 Lineage=Root;rootrank;Bacteria;domain;"Proteobacteria";phylum;Gammaproteobacteria;class;Alteromonadales;order;Alteromonadaceae;family;Haliea;genus	91.69	3.00E-144	512
438	0	0	0	0	0	0	0	37	183	Proteobacteria	Gammaproteobacteria	Oceanospirillales	Saccharospirillaceae	Saccharospirillum	S002916541 Microbulbifer variabilis; HNS025 Lineage=Root;rootrank;Bacteria;domain;"Proteobacteria";phylum;Gammaproteobacteria;class;Alteromonadales;order;Alteromonadaceae;family;Microbulbifer;genus	91.96	2.00E-146	520
2382	9	4	0	0	0	0	0	0	0	Proteobacteria	Gammaproteobacteria	Oceanospirillales			S000531708 Alkanindiges hongkongensis; HKU9 Lineage=Root;rootrank;Bacteria;domain;"Proteobacteria";phylum;Gammaproteobacteria;class;Pseudomonadales;order;Moraxellaceae;family;Alkanindiges;genus	90.88	3.00E-139	496
623	2	7	5	2	3	4	2	2	9	Proteobacteria	Gammaproteobacteria	Oceanospirillales			S002234810 Pseudomonas xinjiangensis; PT-39(1) Lineage=Root;rootrank;Bacteria;domain;"Proteobacteria";phylum;Gammaproteobacteria;class;Pseudomonadales;order;Pseudomonadaceae;family;Pseudomonas;genus	91.98	2.00E-145	516
44	0	0	0	0	0	0	0	5	15	Proteobacteria	Gammaproteobacteria	Oceanospirillales			S002355067 Halioglobus japonicus; S1-36 Lineage=Root;rootrank;Bacteria;domain;"Proteobacteria";phylum;Gammaproteobacteria;class;Alteromonadales;order;Alteromonadaceae;family;Haliea;genus	91.58	5.00E-152	538
1435	0	2	0	0	0	0	0	0	0	Proteobacteria	Gammaproteobacteria	Oceanospirillales			S002355070 Halioglobus pacificus; S1-72 Lineage=Root;rootrank;Bacteria;domain;"Proteobacteria";phylum;Gammaproteobacteria;class;Alteromonadales;order;Alteromonadaceae;family;Haliea;genus	91.82	3.00E-154	545
1229	0	4	0	0	1	0	0	5	14	Proteobacteria	Gammaproteobacteria	Oceanospirillales			S002916001 Porticoccus hydrocarbonoclasticus; MCTG13d Lineage=Root;rootrank;Bacteria;domain;"Proteobacteria";phylum;Gammaproteobacteria;class;Gammaproteobacteria_incertae_sedis;order;Porticoccus;genus	94.62	5.00E-172	604
1651	0	2	9	0	0	6	0	0	0	Proteobacteria	Gammaproteobacteria	Oceanospirillales			S003280090 Pseudomonas luteola; CBN-1 Lineage=Root;rootrank;Bacteria;domain;"Proteobacteria";phylum;Gammaproteobacteria;class;Pseudomonadales;order;Pseudomonadaceae;family;Pseudomonas;genus	91.33	2.00E-150	532
2771	1	0	0	0	1	0	1	6	6	Proteobacteria	Gammaproteobacteria	Oceanospirillales			S003721066 Pseudomonas azotifigens; MA-69 Lineage=Root;rootrank;Bacteria;domain;"Proteobacteria";phylum;Gammaproteobacteria;class;Pseudomonadales;order;Pseudomonadaceae;family;Pseudomonas;genus	92.74	6.00E-151	534
1085	0	0	1	0	0	0	0	0	0	Proteobacteria	Gammaproteobacteria	Pseudomonadales	Moraxellaceae	Acinetobacter	S003750183 Acinetobacter radioresistens; MMC5 Lineage=Root;rootrank;Bacteria;domain;"Proteobacteria";phylum;Gammaproteobacteria;class;Pseudomonadales;order;Moraxellaceae;family;Acinetobacter;genus	100	0	723
2738	0	0	0	13	2	0	0	1	0	Proteobacteria	Gammaproteobacteria	Pseudomonadales	Pseudomonadaceae	Pseudomonas	S000141133 Pseudomonas filiscindens; ATCC BAA-697 Lineage=Root;rootrank;Bacteria;domain;"Proteobacteria";phylum;Gammaproteobacteria;class;Pseudomonadales;order;Pseudomonadaceae;family;unclassified_Pseudomonadaceae;	99.23	0	704
2613	0	0	0	0	1	0	0	0	0	Proteobacteria	Gammaproteobacteria	Pseudomonadales	Pseudomonadaceae	Pseudomonas	S002444400 Serpens flexibilis; PM-37 Lineage=Root;rootrank;Bacteria;domain;"Proteobacteria";phylum;Gammaproteobacteria;class;Pseudomonadales;order;Pseudomonadaceae;family;Serpens;genus	97.44	0	664
737	0	1	0	7	3	1	0	8	16	Proteobacteria	Gammaproteobacteria	Pseudomonadales	Pseudomonadaceae	Pseudomonas	S002958986 Pseudomonas cuatrocienegasensis; LEH6_4A Lineage=Root;rootrank;Bacteria;domain;"Proteobacteria";phylum;Gammaproteobacteria;class;Pseudomonadales;order;Pseudomonadaceae;family;Pseudomonas;genus	98.97	0	699
746	0	0	0	0	0	0	1	0	0	Proteobacteria	Gammaproteobacteria	Pseudomonadales	Pseudomonadaceae	Pseudomonas	S003753610 Pseudomonas stutzeri; CFY1 Lineage=Root;rootrank;Bacteria;domain;"Proteobacteria";phylum;Gammaproteobacteria;class;Pseudomonadales;order;Pseudomonadaceae;family;Pseudomonas;genus	100	0	686
758	0	0	0	1	0	0	0	31	10	Proteobacteria	Gammaproteobacteria	Salinisphaerales			S002032707 Loripes lacteus gill symbiont; 2C Lineage=Root;rootrank;Bacteria;domain;"Proteobacteria";phylum;Gammaproteobacteria;class;Gammaproteobacteria_incertae_sedis;order;Sedimenticola;genus	89.22	1.00E-127	457
2955	11	80	16	0	1	0	0	0	0	Proteobacteria	Gammaproteobacteria	Salinisphaerales	Salinisphaeraceae		S000927654 Thioalkalibacter halophilus (T); ALCO1 Lineage=Root;rootrank;Bacteria;domain;"Proteobacteria";phylum;Gammaproteobacteria;class;Chromatiales;order;Halothiobacillaceae;family;Thioalkalibacter;genus	88.3	4.00E-123	442
2752	5	21	1	0	0	3	9	1	0	Proteobacteria	Gammaproteobacteria	Salinisphaerales	Salinisphaeraceae	Salinisphaera	S000019893 Alkanibacter difficilis (T); MN 154.3 Lineage=Root;rootrank;Bacteria;domain;"Proteobacteria";phylum;Gammaproteobacteria;class;Xanthomonadales;order;Sinobacteraceae;family;Alkanibacter;genus	88.32	1.00E-122	440
2301	6	94	125	11	17	123	230	362	104	Proteobacteria	Gammaproteobacteria	Salinisphaerales	Salinisphaeraceae	Salinisphaera	S000622959 endosymbiont of Ifremeria nautilei Lineage=Root;rootrank;Bacteria;domain;"Proteobacteria";phylum;Gammaproteobacteria;class;Gammaproteobacteria_incertae_sedis;order;Thioprofundum;genus	87.19	2.00E-111	403
1862	6	0	0	0	0	0	0	0	0	Proteobacteria	Gammaproteobacteria	Salinisphaerales	Salinisphaeraceae	Salinisphaera	S000871452 Dokdonella koreensis; NML 01-0233 Lineage=Root;rootrank;Bacteria;domain;"Proteobacteria";phylum;Gammaproteobacteria;class;Xanthomonadales;order;Xanthomonadaceae;family;Dokdonella;genus	87.06	3.00E-114	412
2994	3	26	7	0	0	10	9	4	0	Proteobacteria	Gammaproteobacteria	Salinisphaerales	Salinisphaeraceae	Salinisphaera	S001198079 Thioprofundum lithotrophicum (T); 106 Lineage=Root;rootrank;Bacteria;domain;"Proteobacteria";phylum;Gammaproteobacteria;class;Gammaproteobacteria_incertae_sedis;order;Thioprofundum;genus	88	7.00E-121	435
2617	0	0	0	0	0	1	1	0	0	Proteobacteria	Gammaproteobacteria	Salinisphaerales	Salinisphaeraceae	Salinisphaera	S001198079 Thioprofundum lithotrophicum (T); 106 Lineage=Root;rootrank;Bacteria;domain;"Proteobacteria";phylum;Gammaproteobacteria;class;Gammaproteobacteria_incertae_sedis;order;Thioprofundum;genus	86.8	3.00E-119	429
188	0	0	3	0	0	0	3	0	0	Proteobacteria	Gammaproteobacteria	Salinisphaerales	Salinisphaeraceae	Salinisphaera	S003264052 Hydrocarboniphaga daqingensis; NBRC 104238 Lineage=Root;rootrank;Bacteria;domain;"Proteobacteria";phylum;Gammaproteobacteria;class;Xanthomonadales;order;Sinobacteraceae;family;Hydrocarboniphaga;genus	88.04	3.00E-119	429
1487	24	23	4	1	0	10	25	1	2	Proteobacteria	Gammaproteobacteria	Salinisphaerales	Salinisphaeraceae	Salinisphaera	S003316326 Nevskia ramosa; NA3 Lineage=Root;rootrank;Bacteria;domain;"Proteobacteria";phylum;Gammaproteobacteria;class;Xanthomonadales;order;Sinobacteraceae;family;Nevskia;genus	88.22	7.00E-121	435
2249	0	33	0	0	0	0	0	0	0	Proteobacteria	Gammaproteobacteria	Salinisphaerales	Salinisphaeraceae		S000654142 Thiohalomonas denitrificans; HLD 16 Lineage=Root;rootrank;Bacteria;domain;"Proteobacteria";phylum;Gammaproteobacteria;class;Gammaproteobacteria_incertae_sedis;order;Thiohalomonas;genus	88.74	2.00E-126	453
606	0	4	0	0	0	0	1	0	0	Proteobacteria	Gammaproteobacteria	Salinisphaerales	Salinisphaeraceae		S001872192 Bathymodiolus septemdierum thioautotrophic gill symbiont Lineage=Root;rootrank;Bacteria;domain;"Proteobacteria";phylum;Gammaproteobacteria;class;unclassified_Gammaproteobacteria;	88.17	5.00E-122	438
2099	2	2	1	1	0	3	6	5	2	Proteobacteria	Gammaproteobacteria	Salinisphaerales			S000427629 obligately oligotrophic bacteria KI89C Lineage=Root;rootrank;Bacteria;domain;"Proteobacteria";phylum;Gammaproteobacteria;class;unclassified_Gammaproteobacteria;	88.44	1.00E-123	444
2495	0	1	0	0	0	0	0	0	0	Proteobacteria	Gammaproteobacteria	Salinisphaerales			S000531708 Alkanindiges hongkongensis; HKU9 Lineage=Root;rootrank;Bacteria;domain;"Proteobacteria";phylum;Gammaproteobacteria;class;Pseudomonadales;order;Moraxellaceae;family;Alkanindiges;genus	88.78	1.00E-133	477
1238	4	46	10	1	2	5	6	37	34	Proteobacteria	Gammaproteobacteria	Salinisphaerales			S002290453 Nitrosococcus watsoni C-113 Lineage=Root;rootrank;Bacteria;domain;"Proteobacteria";phylum;Gammaproteobacteria;class;Chromatiales;order;Chromatiaceae;family;Nitrosococcus;genus	88.47	8.00E-125	448
2697	0	0	0	0	0	0	0	1	1	Proteobacteria	Gammaproteobacteria	Thiotrichales			S000425966 Legionella-like amoebal pathogen HT99 Lineage=Root;rootrank;Bacteria;domain;"Proteobacteria";phylum;Gammaproteobacteria;class;unclassified_Gammaproteobacteria;	85.52	2.00E-106	387
3210	0	0	1	0	0	0	0	0	0	Proteobacteria	Gammaproteobacteria	Thiotrichales			S000425966 Legionella-like amoebal pathogen HT99 Lineage=Root;rootrank;Bacteria;domain;"Proteobacteria";phylum;Gammaproteobacteria;class;unclassified_Gammaproteobacteria;	92.86	7.00E-161	568
2582	2	1	0	0	0	3	1	0	0	Proteobacteria	Gammaproteobacteria	Thiotrichales			S000859363 Legionella-like amoebal pathogen CC99 Lineage=Root;rootrank;Bacteria;domain;"Proteobacteria";phylum;Gammaproteobacteria;class;unclassified_Gammaproteobacteria;	97.05	1.00E-178	627
1681	0	0	0	3	8	0	2	1	5	Proteobacteria	Gammaproteobacteria	Thiotrichales	Piscirickettsiaceae	Methylophaga	S000392835 Methylophaga alcalica (T); M39 Lineage=Root;rootrank;Bacteria;domain;"Proteobacteria";phylum;Gammaproteobacteria;class;Thiotrichales;order;Piscirickettsiaceae;family;Methylophaga;genus	99.73	0	682
135	0	0	0	0	0	0	0	0	1	Proteobacteria	Gammaproteobacteria	Thiotrichales			S000859363 Legionella-like amoebal pathogen CC99 Lineage=Root;rootrank;Bacteria;domain;"Proteobacteria";phylum;Gammaproteobacteria;class;unclassified_Gammaproteobacteria;	87.53	7.00E-126	451
1762	0	0	0	0	0	0	0	1	0	Proteobacteria	Gammaproteobacteria	Vibrionales	Vibrionaceae	Vibrio	S003719073 Vibrio rumoiensis; VSD904 Lineage=Root;rootrank;Bacteria;domain;"Proteobacteria";phylum;Gammaproteobacteria;class;"Vibrionales";order;Vibrionaceae;family;Vibrio;genus	100	0	686
1777	0	0	0	1	0	0	0	0	1	Proteobacteria	Gammaproteobacteria	Xanthomonadales			S000547277 Hydrocarboniphaga effusa; AP102 Lineage=Root;rootrank;Bacteria;domain;"Proteobacteria";phylum;Gammaproteobacteria;class;Xanthomonadales;order;Sinobacteraceae;family;Hydrocarboniphaga;genus	91.91	5.00E-147	521
2528	0	0	0	0	0	0	0	0	3	Proteobacteria	Gammaproteobacteria	Xanthomonadales			S000769243 Solimonas soli; DCY15 Lineage=Root;rootrank;Bacteria;domain;"Proteobacteria";phylum;Gammaproteobacteria;class;Gammaproteobacteria_incertae_sedis;order;Solimonas;genus	91.58	5.00E-152	538
3089	0	0	0	1	0	0	0	0	0	Proteobacteria	Gammaproteobacteria	Xanthomonadales			S000769243 Solimonas soli; DCY15 Lineage=Root;rootrank;Bacteria;domain;"Proteobacteria";phylum;Gammaproteobacteria;class;Gammaproteobacteria_incertae_sedis;order;Solimonas;genus	92.23	4.00E-148	525
1070	0	0	0	0	6	0	0	0	1	Proteobacteria	Gammaproteobacteria	Xanthomonadales			S002355520 Polycyclovorans algicola; TG408 Lineage=Root;rootrank;Bacteria;domain;"Proteobacteria";phylum;Gammaproteobacteria;class;Xanthomonadales;order;Sinobacteraceae;family;Singularimonas;genus	98.98	0	701
167	0	0	6	105	23	0	0	0	0	Proteobacteria	Gammaproteobacteria	Xanthomonadales	Xanthomonadaceae		S000571431 Dokdonella fugitiva (T); type strain: A3 Lineage=Root;rootrank;Bacteria;domain;"Proteobacteria";phylum;Gammaproteobacteria;class;Xanthomonadales;order;Xanthomonadaceae;family;Dokdonella;genus	94.89	2.00E-165	582
753	0	0	0	3	2	0	0	0	1	Proteobacteria	Gammaproteobacteria	Xanthomonadales	Xanthomonadaceae		S000727896 Dyella koreensis; 1-A-4 Lineage=Root;rootrank;Bacteria;domain;"Proteobacteria";phylum;Gammaproteobacteria;class;Xanthomonadales;order;Xanthomonadaceae;family;Dyella;genus	98.39	0	654
1398	0	0	40	82	80	0	1	58	158	Proteobacteria	Gammaproteobacteria	Xanthomonadales	Xanthomonadaceae		S000968192 Lysobacter pocheonensis; Gsoil 193 Lineage=Root;rootrank;Bacteria;domain;"Proteobacteria";phylum;Gammaproteobacteria;class;Xanthomonadales;order;Xanthomonadaceae;family;Lysobacter;genus	95.97	5.00E-172	604
1230	0	0	0	5	0	0	0	0	0	Proteobacteria	Gammaproteobacteria	Xanthomonadales	Xanthomonadaceae		S002223474 Arenimonas oryziterrae; YC6267 Lineage=Root;rootrank;Bacteria;domain;"Proteobacteria";phylum;Gammaproteobacteria;class;Xanthomonadales;order;Xanthomonadaceae;family;Arenimonas;genus	96.94	0	658
669	0	0	7	183	15	17	11	11	12	Proteobacteria	Gammaproteobacteria	Xanthomonadales	Xanthomonadaceae		S002408791 Lysobacter panaciterrae; MTH26 Lineage=Root;rootrank;Bacteria;domain;"Proteobacteria";phylum;Gammaproteobacteria;class;Xanthomonadales;order;Xanthomonadaceae;family;unclassified_Xanthomonadaceae;	98.66	0	660
2946	0	0	0	8	0	0	0	0	0	Proteobacteria	Gammaproteobacteria	Xanthomonadales	Xanthomonadaceae		S003264293 Arenimonas donghaensis; NBRC 106386 Lineage=Root;rootrank;Bacteria;domain;"Proteobacteria";phylum;Gammaproteobacteria;class;Xanthomonadales;order;Xanthomonadaceae;family;Arenimonas;genus	95.7	2.00E-170	599
1407	0	0	0	2	1	0	0	2	2	Proteobacteria	Gammaproteobacteria	Xanthomonadales	Xanthomonadaceae	Arenimonas	S000619973 Arenimonas malthae (T); CC-JY-1 Lineage=Root;rootrank;Bacteria;domain;"Proteobacteria";phylum;Gammaproteobacteria;class;Xanthomonadales;order;Xanthomonadaceae;family;Arenimonas;genus	99.19	0	671
1486	1	0	0	1	0	0	0	0	0	Proteobacteria	Gammaproteobacteria	Xanthomonadales	Xanthomonadaceae	Luteibacter	S002441004 Luteibacter rhizovicinus; EC-V30-gmG1 Lineage=Root;rootrank;Bacteria;domain;"Proteobacteria";phylum;Gammaproteobacteria;class;Xanthomonadales;order;Xanthomonadaceae;family;Luteibacter;genus	97.31	2.00E-180	632
2579	2	0	0	0	0	0	0	0	0	Proteobacteria	Gammaproteobacteria	Xanthomonadales	Xanthomonadaceae	Luteibacter	S003751039 Dyella ginsengisoli; ED50 Lineage=Root;rootrank;Bacteria;domain;"Proteobacteria";phylum;Gammaproteobacteria;class;Xanthomonadales;order;Xanthomonadaceae;family;Dyella;genus	92.95	2.00E-151	536
1050	0	0	138	198	136	10	12	26	354	Proteobacteria	Gammaproteobacteria	Xanthomonadales	Xanthomonadaceae	Lysobacter	S000542979 Lysobacter concretionis (T); Ko07 Lineage=Root;rootrank;Bacteria;domain;"Proteobacteria";phylum;Gammaproteobacteria;class;Xanthomonadales;order;Xanthomonadaceae;family;Lysobacter;genus	98.92	0	665
78	0	0	0	1	0	0	0	0	0	Proteobacteria	Gammaproteobacteria	Xanthomonadales	Xanthomonadaceae	Pseudoxanthomonas	S000375272 Pseudoxanthomonas kalamensis (T); JA40 Lineage=Root;rootrank;Bacteria;domain;"Proteobacteria";phylum;Gammaproteobacteria;class;Xanthomonadales;order;Xanthomonadaceae;family;Pseudoxanthomonas;genus	97.58	0	638
87	0	0	0	0	0	0	0	0	2	Proteobacteria	Gammaproteobacteria	Xanthomonadales	Xanthomonadaceae		S000140772 Thermomonas hydrothermalis (T); SGM-6 Lineage=Root;rootrank;Bacteria;domain;"Proteobacteria";phylum;Gammaproteobacteria;class;Xanthomonadales;order;Xanthomonadaceae;family;Thermomonas;genus	94.64	1.00E-172	606
1242	0	0	0	1	0	0	1	0	0	Proteobacteria	Gammaproteobacteria	Xanthomonadales	Xanthomonadaceae		S000571431 Dokdonella fugitiva (T); type strain: A3 Lineage=Root;rootrank;Bacteria;domain;"Proteobacteria";phylum;Gammaproteobacteria;class;Xanthomonadales;order;Xanthomonadaceae;family;Dokdonella;genus	94.66	4.00E-173	608
954	0	0	0	12	1	0	2	1	0	Proteobacteria	Gammaproteobacteria	Xanthomonadales	Xanthomonadaceae		S002224467 Luteimonas lutimaris; G3 Lineage=Root;rootrank;Bacteria;domain;"Proteobacteria";phylum;Gammaproteobacteria;class;Xanthomonadales;order;Xanthomonadaceae;family;Luteimonas;genus	99.46	0	676
2974	0	0	0	0	0	0	0	0	4	Proteobacteria	Gammaproteobacteria	Xanthomonadales	Xanthomonadaceae		S002225292 Lysobacter capsici; M63031 Lineage=Root;rootrank;Bacteria;domain;"Proteobacteria";phylum;Gammaproteobacteria;class;Xanthomonadales;order;Xanthomonadaceae;family;Lysobacter;genus	96.25	1.00E-173	610
2866	0	0	0	1	0	0	0	0	0	Proteobacteria	Gammaproteobacteria	Xanthomonadales			S000547277 Hydrocarboniphaga effusa; AP102 Lineage=Root;rootrank;Bacteria;domain;"Proteobacteria";phylum;Gammaproteobacteria;class;Xanthomonadales;order;Sinobacteraceae;family;Hydrocarboniphaga;genus	90.84	2.00E-140	499
3086	0	0	0	1	0	0	0	0	0	Proteobacteria	Gammaproteobacteria				S000003124 Legionella quinlivanii (T); sreogroup 2, nctc 12433 Lineage=Root;rootrank;Bacteria;domain;"Proteobacteria";phylum;Gammaproteobacteria;class;Legionellales;order;Legionellaceae;family;Legionella;genus	91.73	2.00E-145	516
477	10	94	141	7	9	55	117	254	292	Proteobacteria	Gammaproteobacteria				S000007569 Lucina floridana gill symbiont Lineage=Root;rootrank;Bacteria;domain;"Proteobacteria";phylum;Gammaproteobacteria;class;unclassified_Gammaproteobacteria;	90.27	2.00E-135	483
931	0	0	1	0	1	7	13	1	0	Proteobacteria	Gammaproteobacteria				S000010442 Rhabdochromatium marinum (T) Lineage=Root;rootrank;Bacteria;domain;"Proteobacteria";phylum;Gammaproteobacteria;class;Chromatiales;order;Chromatiaceae;family;Rhabdochromatium;genus	85.64	4.00E-113	409
1771	0	0	0	1	0	0	0	0	1	Proteobacteria	Gammaproteobacteria				S000011218 Lucina nassula gill symbiont Lineage=Root;rootrank;Bacteria;domain;"Proteobacteria";phylum;Gammaproteobacteria;class;Gammaproteobacteria_incertae_sedis;order;unclassified_Gammaproteobacteria_incertae_sedis;	91.87	2.00E-145	516
225	0	0	0	1	0	0	0	0	0	Proteobacteria	Gammaproteobacteria				S000335574 Fangia hongkongensis (T); UST040201-002; CML28 Lineage=Root;rootrank;Bacteria;domain;"Proteobacteria";phylum;Gammaproteobacteria;class;Thiotrichales;order;Thiotrichales_incertae_sedis;family;Fangia;genus	86.06	9.00E-110	398
2592	12	7	0	0	0	0	0	0	1	Proteobacteria	Gammaproteobacteria				S000388423 vestimentiferan endosymbiont 'Shinkai #6500' Lineage=Root;rootrank;Bacteria;domain;"Proteobacteria";phylum;Gammaproteobacteria;class;Gammaproteobacteria_incertae_sedis;order;Sedimenticola;genus	90.13	3.00E-134	479
3140	26	12	17	0	3	21	19	18	20	Proteobacteria	Gammaproteobacteria				S000396664 endosymbiont of Lamellibrachia barhami; host individual 11 Lineage=Root;rootrank;Bacteria;domain;"Proteobacteria";phylum;Gammaproteobacteria;class;Gammaproteobacteria_incertae_sedis;order;unclassified_Gammaproteobacteria_incertae_sedis;	90.86	8.00E-140	497
658	0	1	0	0	0	0	0	0	1	Proteobacteria	Gammaproteobacteria				S000425966 Legionella-like amoebal pathogen HT99 Lineage=Root;rootrank;Bacteria;domain;"Proteobacteria";phylum;Gammaproteobacteria;class;unclassified_Gammaproteobacteria;	87.53	7.00E-126	451
1906	0	0	0	0	0	0	0	1	0	Proteobacteria	Gammaproteobacteria				S000425966 Legionella-like amoebal pathogen HT99 Lineage=Root;rootrank;Bacteria;domain;"Proteobacteria";phylum;Gammaproteobacteria;class;unclassified_Gammaproteobacteria;	89.82	2.00E-140	499
1039	0	0	0	7	1	0	0	0	0	Proteobacteria	Gammaproteobacteria				S000438737 Methylocaldum gracile (T); VKM-14L Lineage=Root;rootrank;Bacteria;domain;"Proteobacteria";phylum;Gammaproteobacteria;class;Methylococcales;order;Methylococcaceae;family;Methylocaldum;genus	92.58	3.00E-159	562
703	0	0	0	16	6	0	0	0	0	Proteobacteria	Gammaproteobacteria				S000547277 Hydrocarboniphaga effusa; AP102 Lineage=Root;rootrank;Bacteria;domain;"Proteobacteria";phylum;Gammaproteobacteria;class;Xanthomonadales;order;Sinobacteraceae;family;Hydrocarboniphaga;genus	88.53	2.00E-125	449
2436	0	0	0	0	0	0	0	0	1	Proteobacteria	Gammaproteobacteria				S000654142 Thiohalomonas denitrificans; HLD 16 Lineage=Root;rootrank;Bacteria;domain;"Proteobacteria";phylum;Gammaproteobacteria;class;Gammaproteobacteria_incertae_sedis;order;Thiohalomonas;genus	91.07	1.00E-148	527
1927	0	0	3	0	0	1	2	0	0	Proteobacteria	Gammaproteobacteria				S000751826 Legionella jordanis; NML 060502 Lineage=Root;rootrank;Bacteria;domain;"Proteobacteria";phylum;Gammaproteobacteria;class;Legionellales;order;Legionellaceae;family;Legionella;genus	89.62	3.00E-139	496
649	55	132	134	23	47	90	116	10	24	Proteobacteria	Gammaproteobacteria				S000769575 Thiohalomonas denitrificans; HLD 14 Lineage=Root;rootrank;Bacteria;domain;"Proteobacteria";phylum;Gammaproteobacteria;class;Gammaproteobacteria_incertae_sedis;order;Thiohalomonas;genus	90.84	4.00E-148	525
18	0	0	0	0	0	1	0	0	0	Proteobacteria	Gammaproteobacteria				S000769575 Thiohalomonas denitrificans; HLD 14 Lineage=Root;rootrank;Bacteria;domain;"Proteobacteria";phylum;Gammaproteobacteria;class;Gammaproteobacteria_incertae_sedis;order;Thiohalomonas;genus	90.56	2.00E-145	516
1215	0	0	3	3	3	0	0	16	59	Proteobacteria	Gammaproteobacteria				S000806276 Marinobacter taiwanensis; GI-1701 Lineage=Root;rootrank;Bacteria;domain;"Proteobacteria";phylum;Gammaproteobacteria;class;Alteromonadales;order;Alteromonadaceae;family;Marinobacter;genus	94.07	8.00E-160	564
1317	0	0	5	6	7	1	3	1	2	Proteobacteria	Gammaproteobacteria				S000824755 Coxiella cheraxi; TO-98 Lineage=Root;rootrank;Bacteria;domain;"Proteobacteria";phylum;Gammaproteobacteria;class;Legionellales;order;Coxiellaceae;family;Coxiella;genus	91.91	6.00E-146	518
1447	0	0	0	0	0	1	0	0	0	Proteobacteria	Gammaproteobacteria				S000824755 Coxiella cheraxi; TO-98 Lineage=Root;rootrank;Bacteria;domain;"Proteobacteria";phylum;Gammaproteobacteria;class;Legionellales;order;Coxiellaceae;family;Coxiella;genus	91.03	4.00E-148	525
2006	0	0	0	0	0	0	0	0	1	Proteobacteria	Gammaproteobacteria				S000824755 Coxiella cheraxi; TO-98 Lineage=Root;rootrank;Bacteria;domain;"Proteobacteria";phylum;Gammaproteobacteria;class;Legionellales;order;Coxiellaceae;family;Coxiella;genus	87.5	3.00E-125	449
230	0	0	1	0	0	0	0	3	9	Proteobacteria	Gammaproteobacteria				S000843124 Thiohalomonas denitrificans; HLD 15 Lineage=Root;rootrank;Bacteria;domain;"Proteobacteria";phylum;Gammaproteobacteria;class;Gammaproteobacteria_incertae_sedis;order;Thiohalomonas;genus	90.82	5.00E-147	521
111	1	0	0	0	0	0	0	0	0	Proteobacteria	Gammaproteobacteria				S000843124 Thiohalomonas denitrificans; HLD 15 Lineage=Root;rootrank;Bacteria;domain;"Proteobacteria";phylum;Gammaproteobacteria;class;Gammaproteobacteria_incertae_sedis;order;Thiohalomonas;genus	89.8	7.00E-141	501
1405	0	0	0	0	0	1	1	0	0	Proteobacteria	Gammaproteobacteria				S000843124 Thiohalomonas denitrificans; HLD 15 Lineage=Root;rootrank;Bacteria;domain;"Proteobacteria";phylum;Gammaproteobacteria;class;Gammaproteobacteria_incertae_sedis;order;Thiohalomonas;genus	90.84	5.00E-147	521
3006	0	0	1	1	0	0	0	7	2	Proteobacteria	Gammaproteobacteria				S000859363 Legionella-like amoebal pathogen CC99 Lineage=Root;rootrank;Bacteria;domain;"Proteobacteria";phylum;Gammaproteobacteria;class;unclassified_Gammaproteobacteria;	89.12	1.00E-128	460
794	0	0	0	1	0	0	0	0	0	Proteobacteria	Gammaproteobacteria				S000859363 Legionella-like amoebal pathogen CC99 Lineage=Root;rootrank;Bacteria;domain;"Proteobacteria";phylum;Gammaproteobacteria;class;unclassified_Gammaproteobacteria;	86.97	9.00E-115	414
36	0	0	0	0	0	0	0	1	0	Proteobacteria	Gammaproteobacteria				S000859363 Legionella-like amoebal pathogen CC99 Lineage=Root;rootrank;Bacteria;domain;"Proteobacteria";phylum;Gammaproteobacteria;class;unclassified_Gammaproteobacteria;	84.04	1.00E-97	357
2656	0	0	0	0	0	0	0	1	0	Proteobacteria	Gammaproteobacteria				S000859363 Legionella-like amoebal pathogen CC99 Lineage=Root;rootrank;Bacteria;domain;"Proteobacteria";phylum;Gammaproteobacteria;class;unclassified_Gammaproteobacteria;	88.1	3.00E-129	462
1375	0	0	0	1	0	0	0	0	0	Proteobacteria	Gammaproteobacteria				S000891236 Proteinimicrobium ihbtica; type strain: HMPB3 Lineage=Root;rootrank;Bacteria;domain;"Proteobacteria";phylum;Betaproteobacteria;class;unclassified_Betaproteobacteria;	87.53	9.00E-125	448
2194	0	0	0	1	0	0	0	0	0	Proteobacteria	Gammaproteobacteria				S000891236 Proteinimicrobium ihbtica; type strain: HMPB3 Lineage=Root;rootrank;Bacteria;domain;"Proteobacteria";phylum;Betaproteobacteria;class;unclassified_Betaproteobacteria;	87.66	2.00E-126	453
786	0	0	1	0	0	0	1	0	0	Proteobacteria	Gammaproteobacteria				S000891953 Methylohalomonas lacus (T); HMT 1 Lineage=Root;rootrank;Bacteria;domain;"Proteobacteria";phylum;Gammaproteobacteria;class;Gammaproteobacteria_incertae_sedis;order;Methylohalomonas;genus	90.33	9.00E-145	514
1498	0	0	0	1	0	0	0	0	0	Proteobacteria	Gammaproteobacteria				S000902254 Candidatus Allobeggiatoa halophila Lineage=Root;rootrank;Bacteria;domain;"Proteobacteria";phylum;Gammaproteobacteria;class;Thiotrichales;order;Thiotrichaceae;family;Beggiatoa;genus	90.86	8.00E-140	497
415	0	0	1	0	2	1	1	0	0	Proteobacteria	Gammaproteobacteria				S000942429 sulfur-oxidizing symbiont of Inandrilus exumae Lineage=Root;rootrank;Bacteria;domain;"Proteobacteria";phylum;Gammaproteobacteria;class;unclassified_Gammaproteobacteria;	87.1	7.00E-116	418
2641	0	0	0	0	0	0	0	1	0	Proteobacteria	Gammaproteobacteria				S000942429 sulfur-oxidizing symbiont of Inandrilus exumae Lineage=Root;rootrank;Bacteria;domain;"Proteobacteria";phylum;Gammaproteobacteria;class;unclassified_Gammaproteobacteria;	91.44	4.00E-143	508
1572	0	0	0	0	0	0	0	0	1	Proteobacteria	Gammaproteobacteria				S000995323 Steroidobacter denitrificans (T); FS Lineage=Root;rootrank;Bacteria;domain;"Proteobacteria";phylum;Gammaproteobacteria;class;Xanthomonadales;order;Sinobacteraceae;family;Steroidobacter;genus	87.19	4.00E-123	442
507	494	1156	2371	201	622	1953	3014	4968	4881	Proteobacteria	Gammaproteobacteria				S001153278 Methylogaea oryzae JCM 16910; E10 Lineage=Root;rootrank;Bacteria;domain;"Proteobacteria";phylum;Gammaproteobacteria;class;Methylococcales;order;Methylococcaceae;family;Methylococcus;genus	91.62	1.00E-143	510
1009	0	0	0	0	0	0	0	1	0	Proteobacteria	Gammaproteobacteria				S001153278 Methylogaea oryzae JCM 16910; E10 Lineage=Root;rootrank;Bacteria;domain;"Proteobacteria";phylum;Gammaproteobacteria;class;Methylococcales;order;Methylococcaceae;family;Methylococcus;genus	89.87	2.00E-141	503
1758	0	0	0	0	0	0	0	1	0	Proteobacteria	Gammaproteobacteria				S001153278 Methylogaea oryzae JCM 16910; E10 Lineage=Root;rootrank;Bacteria;domain;"Proteobacteria";phylum;Gammaproteobacteria;class;Methylococcales;order;Methylococcaceae;family;Methylococcus;genus	89.22	1.00E-128	460
604	0	0	0	0	0	0	0	1	0	Proteobacteria	Gammaproteobacteria				S001153278 Methylogaea oryzae JCM 16910; E10 Lineage=Root;rootrank;Bacteria;domain;"Proteobacteria";phylum;Gammaproteobacteria;class;Methylococcales;order;Methylococcaceae;family;Methylococcus;genus	89.34	1.00E-137	490
1193	3	1	0	0	0	0	0	0	0	Proteobacteria	Gammaproteobacteria				S001198079 Thioprofundum lithotrophicum (T); 106 Lineage=Root;rootrank;Bacteria;domain;"Proteobacteria";phylum;Gammaproteobacteria;class;Gammaproteobacteria_incertae_sedis;order;Thioprofundum;genus	91.42	1.00E-143	510
529	1	0	0	0	0	0	0	1	0	Proteobacteria	Gammaproteobacteria				S001198079 Thioprofundum lithotrophicum (T); 106 Lineage=Root;rootrank;Bacteria;domain;"Proteobacteria";phylum;Gammaproteobacteria;class;Gammaproteobacteria_incertae_sedis;order;Thioprofundum;genus	93.42	3.00E-164	579
452	0	1	0	0	0	0	0	0	0	Proteobacteria	Gammaproteobacteria				S001198079 Thioprofundum lithotrophicum (T); 106 Lineage=Root;rootrank;Bacteria;domain;"Proteobacteria";phylum;Gammaproteobacteria;class;Gammaproteobacteria_incertae_sedis;order;Thioprofundum;genus	87.34	9.00E-125	448
572	0	0	0	0	0	1	0	0	0	Proteobacteria	Gammaproteobacteria				S001198079 Thioprofundum lithotrophicum (T); 106 Lineage=Root;rootrank;Bacteria;domain;"Proteobacteria";phylum;Gammaproteobacteria;class;Gammaproteobacteria_incertae_sedis;order;Thioprofundum;genus	88.47	3.00E-124	446
555	0	0	0	0	0	0	0	1	0	Proteobacteria	Gammaproteobacteria				S001198079 Thioprofundum lithotrophicum (T); 106 Lineage=Root;rootrank;Bacteria;domain;"Proteobacteria";phylum;Gammaproteobacteria;class;Gammaproteobacteria_incertae_sedis;order;Thioprofundum;genus	88.8	1.00E-127	457
1694	0	0	0	0	0	0	0	1	0	Proteobacteria	Gammaproteobacteria				S001198079 Thioprofundum lithotrophicum (T); 106 Lineage=Root;rootrank;Bacteria;domain;"Proteobacteria";phylum;Gammaproteobacteria;class;Gammaproteobacteria_incertae_sedis;order;Thioprofundum;genus	92.2	4.00E-148	525
1781	0	0	2	0	0	0	0	0	0	Proteobacteria	Gammaproteobacteria				S001793637 Thiohalobacter thiocyanaticus (T); HRh1 Lineage=Root;rootrank;Bacteria;domain;"Proteobacteria";phylum;Gammaproteobacteria;class;Gammaproteobacteria_incertae_sedis;order;Thiohalobacter;genus	92.07	7.00E-156	551
738	0	0	0	0	0	0	0	0	1	Proteobacteria	Gammaproteobacteria				S001793637 Thiohalobacter thiocyanaticus (T); HRh1 Lineage=Root;rootrank;Bacteria;domain;"Proteobacteria";phylum;Gammaproteobacteria;class;Gammaproteobacteria_incertae_sedis;order;Thiohalobacter;genus	88.83	1.00E-127	457
716	0	0	0	0	1	0	0	0	0	Proteobacteria	Gammaproteobacteria				S001794555 Pseudomonas koreensis; PBRs7 Lineage=Root;rootrank;Bacteria;domain;"Proteobacteria";phylum;Gammaproteobacteria;class;Pseudomonadales;order;Pseudomonadaceae;family;Pseudomonas;genus	88.04	3.00E-129	462
2125	0	0	0	1	1	0	0	0	0	Proteobacteria	Gammaproteobacteria				S002224467 Luteimonas lutimaris; G3 Lineage=Root;rootrank;Bacteria;domain;"Proteobacteria";phylum;Gammaproteobacteria;class;Xanthomonadales;order;Xanthomonadaceae;family;Luteimonas;genus	87.3	7.00E-116	418
1642	0	0	1	2	2	0	0	5	2	Proteobacteria	Gammaproteobacteria				S002233493 Marinobacter pelagius; CT43-2 Lineage=Root;rootrank;Bacteria;domain;"Proteobacteria";phylum;Gammaproteobacteria;class;Alteromonadales;order;Alteromonadaceae;family;Marinobacter;genus	93.26	8.00E-155	547
870	10	79	39	1	13	48	104	18	11	Proteobacteria	Gammaproteobacteria				S002290453 Nitrosococcus watsoni C-113 Lineage=Root;rootrank;Bacteria;domain;"Proteobacteria";phylum;Gammaproteobacteria;class;Chromatiales;order;Chromatiaceae;family;Nitrosococcus;genus	88.38	4.00E-123	442
2141	6	0	0	0	0	0	4	0	0	Proteobacteria	Gammaproteobacteria				S002290453 Nitrosococcus watsoni C-113 Lineage=Root;rootrank;Bacteria;domain;"Proteobacteria";phylum;Gammaproteobacteria;class;Chromatiales;order;Chromatiaceae;family;Nitrosococcus;genus	90.57	4.00E-138	492
1038	0	0	0	0	1	0	1	0	2	Proteobacteria	Gammaproteobacteria				S002291134 Coxiella burnetii RSA 331 Lineage=Root;rootrank;Bacteria;domain;"Proteobacteria";phylum;Gammaproteobacteria;class;Legionellales;order;Coxiellaceae;family;Coxiella;genus	91.05	4.00E-148	525
893	0	0	0	0	1	0	0	1	0	Proteobacteria	Gammaproteobacteria				S002291134 Coxiella burnetii RSA 331 Lineage=Root;rootrank;Bacteria;domain;"Proteobacteria";phylum;Gammaproteobacteria;class;Legionellales;order;Coxiellaceae;family;Coxiella;genus	91.28	9.00E-150	531
3228	0	0	0	0	0	0	0	0	1	Proteobacteria	Gammaproteobacteria				S002291134 Coxiella burnetii RSA 331 Lineage=Root;rootrank;Bacteria;domain;"Proteobacteria";phylum;Gammaproteobacteria;class;Legionellales;order;Coxiellaceae;family;Coxiella;genus	88.49	2.00E-131	470
1344	0	0	0	1	0	0	0	0	0	Proteobacteria	Gammaproteobacteria				S002305822 Thalassomonas agarivorans; M23b Lineage=Root;rootrank;Bacteria;domain;"Proteobacteria";phylum;Gammaproteobacteria;class;Alteromonadales;order;Colwelliaceae;family;Thalassomonas;genus	86.48	4.00E-118	425
107	0	0	1	0	0	0	0	0	1	Proteobacteria	Gammaproteobacteria				S002407953 Thioploca ingrica; DEN002 Lineage=Root;rootrank;Bacteria;domain;"Proteobacteria";phylum;Gammaproteobacteria;class;unclassified_Gammaproteobacteria;	93.1	4.00E-92	339
2661	0	0	0	0	0	0	0	0	1	Proteobacteria	Gammaproteobacteria				S002916742 Methylococcus capsulatus; Clc Lineage=Root;rootrank;Bacteria;domain;"Proteobacteria";phylum;Gammaproteobacteria;class;Methylococcales;order;Methylococcaceae;family;Methylococcus;genus	92.6	3.00E-159	562
1146	44	123	114	40	61	49	47	19	25	Proteobacteria	Gammaproteobacteria				S003258418 endosymbiont of Sclerolinum contortum; 1099 Lineage=Root;rootrank;Bacteria;domain;"Proteobacteria";phylum;Gammaproteobacteria;class;Gammaproteobacteria_incertae_sedis;order;Sedimenticola;genus	91.08	2.00E-140	499
2629	0	3	0	0	1	4	1	11	3	Proteobacteria	Gammaproteobacteria				S003263792 Porticoccus litoralis; NBRC 102686 Lineage=Root;rootrank;Bacteria;domain;"Proteobacteria";phylum;Gammaproteobacteria;class;Gammaproteobacteria_incertae_sedis;order;Porticoccus;genus	91.35	2.00E-150	532
2704	0	0	0	0	0	0	0	0	1	Proteobacteria	Gammaproteobacteria				S003264294 Luteibacter yeojuensis; NBRC 106387 Lineage=Root;rootrank;Bacteria;domain;"Proteobacteria";phylum;Gammaproteobacteria;class;Xanthomonadales;order;Xanthomonadaceae;family;Luteibacter;genus	91.09	4.00E-113	409
689	0	2	0	0	0	0	3	0	0	Proteobacteria	Gammaproteobacteria				S003617958 endosymbiont of Ridgeia piscesae; LF8CM1b18 Lineage=Root;rootrank;Bacteria;domain;"Proteobacteria";phylum;Gammaproteobacteria;class;Gammaproteobacteria_incertae_sedis;order;Sedimenticola;genus	88.95	3.00E-134	479
1626	1	0	0	0	0	0	1	0	0	Proteobacteria	Gammaproteobacteria				S003617966 endosymbiont of Ridgeia piscesae; LF8CM1b5 Lineage=Root;rootrank;Bacteria;domain;"Proteobacteria";phylum;Gammaproteobacteria;class;unclassified_Gammaproteobacteria;	87.23	7.00E-116	418
2600	0	0	0	0	0	0	0	1	21	Proteobacteria	Gammaproteobacteria				S003719002 Spongiibacter tropicus; VSG724b Lineage=Root;rootrank;Bacteria;domain;"Proteobacteria";phylum;Gammaproteobacteria;class;Gammaproteobacteria_incertae_sedis;order;Spongiibacter;genus	93.86	5.00E-167	588
169	0	0	0	1	1	0	1	0	1	Proteobacteria					S000022588 Haliangium tepidum (T); SMP-10 Lineage=Root;rootrank;Bacteria;domain;"Proteobacteria";phylum;Deltaproteobacteria;class;Myxococcales;order;Nannocystineae;suborder;"Haliangiaceae";family;Haliangium;genus	86.22	2.00E-117	424
151	0	0	0	2	0	0	0	0	0	Proteobacteria					S000022588 Haliangium tepidum (T); SMP-10 Lineage=Root;rootrank;Bacteria;domain;"Proteobacteria";phylum;Deltaproteobacteria;class;Myxococcales;order;Nannocystineae;suborder;"Haliangiaceae";family;Haliangium;genus	86.48	1.00E-118	427
1089	0	0	0	1	0	0	0	0	0	Proteobacteria					S000022588 Haliangium tepidum (T); SMP-10 Lineage=Root;rootrank;Bacteria;domain;"Proteobacteria";phylum;Deltaproteobacteria;class;Myxococcales;order;Nannocystineae;suborder;"Haliangiaceae";family;Haliangium;genus	85.97	3.00E-115	416
1335	2	0	2	0	0	0	0	1	0	Proteobacteria					S000392255 Desulfuromonas michiganensis (T); BB1 Lineage=Root;rootrank;Bacteria;domain;"Proteobacteria";phylum;Deltaproteobacteria;class;Desulfuromonadales;order;Desulfuromonadaceae;family;Desulfuromonas;genus	91.33	5.00E-142	505
3166	0	0	0	0	0	0	0	1	1	Proteobacteria					S000392255 Desulfuromonas michiganensis (T); BB1 Lineage=Root;rootrank;Bacteria;domain;"Proteobacteria";phylum;Deltaproteobacteria;class;Desulfuromonadales;order;Desulfuromonadaceae;family;Desulfuromonas;genus	85.2	7.00E-111	401
1246	0	0	0	1	0	0	0	0	0	Proteobacteria					S000428823 Desulfotomaculum alkaliphilum (T); S1 Lineage=Root;rootrank;Bacteria;domain;Firmicutes;phylum;Clostridia;class;Clostridiales;order;Peptococcaceae 2;family;Desulfotomaculum;genus	82.78	7.00E-96	351
2127	0	0	0	0	0	0	1	0	0	Proteobacteria					S000841866 Subaequorebacter tamlense; type strain: HST3-12 Lineage=Root;rootrank;Bacteria;domain;"Proteobacteria";phylum;Alphaproteobacteria;class;Alphaproteobacteria_incertae_sedis;order;Geminicoccus;genus	88.97	3.00E-134	479

202	2	2	5	0	0	2	3	0	0	Proteobacteria						S000843124 Thiohalomonas denitrificans; HLD 15 Lineage=Root;rootrank;Bacteria;domain;"Proteobacteria";phylum;Gammaproteobacteria;class;Gammaproteobacteria_incertae_sedis;order;Thiohalomonas;genus	87.63	3.00E-119	429
693	0	0	0	0	0	0	0	1	1	Proteobacteria						S000902254 Candidatus Allobeggiatoa halophila Lineage=Root;rootrank;Bacteria;domain;"Proteobacteria";phylum;Gammaproteobacteria;class;Thiotrichales;order;Thiotrichaceae;family;Beggiatoa;genus	85.5	2.00E-112	407
1218	0	0	1	0	0	0	3	0	0	Proteobacteria						S001188435 Anaeromyxobacter dehalogenans; FRC-R8 Lineage=Root;rootrank;Bacteria;domain;"Proteobacteria";phylum;Deltaproteobacteria;class;Myxococcales;order;Cystobacterineae;suborder;Cystobacteraceae;family;Anaeromyxobacter;genus	88.52	5.00E-132	472
2258	0	1	0	0	0	0	0	0	0	Proteobacteria						S002224491 Jahnella thaxteri; Pl t4 Lineage=Root;rootrank;Bacteria;domain;"Proteobacteria";phylum;Deltaproteobacteria;class;Myxococcales;order;Sorangiineae;suborder;Polyangiaceae;family;unclassified_Polyangiaceae;	85.56	7.00E-106	385
1886	0	0	0	0	0	0	0	1	0	Proteobacteria						S002236156 Desulfomicrobium orale; JCM 17150 Lineage=Root;rootrank;Bacteria;domain;"Proteobacteria";phylum;Deltaproteobacteria;class;Desulfovibrionales;order;Desulfomicrobiaceae;family;Desulfomicrobium;genus	85.5	2.00E-111	403
1984	0	0	0	0	1	0	0	0	0	Proteobacteria						S002288029 Syntrophus aciditrophicus (T); SB Lineage=Root;rootrank;Bacteria;domain;"Proteobacteria";phylum;Deltaproteobacteria;class;Syntrophobacterales;order;Syntrophaceae;family;Syntrophus;genus	85.41	2.00E-105	383
68	0	0	0	2	0	0	0	0	0	Proteobacteria						S002290453 Nitrosococcus watsoni C-113 Lineage=Root;rootrank;Bacteria;domain;"Proteobacteria";phylum;Gammaproteobacteria;class;Chromatiales;order;Chromatiaceae;family;Nitrosococcus;genus	86.83	9.00E-115	414
2844	0	0	0	0	0	0	0	1	0	Proteobacteria						S002290453 Nitrosococcus watsoni C-113 Lineage=Root;rootrank;Bacteria;domain;"Proteobacteria";phylum;Gammaproteobacteria;class;Chromatiales;order;Chromatiaceae;family;Nitrosococcus;genus	85.38	7.00E-111	401
15	0	0	0	0	0	0	0	0	1	Proteobacteria						S002290737 Pelobacter propionicus (T); DSM 2379 Lineage=Root;rootrank;Bacteria;domain;"Proteobacteria";phylum;Deltaproteobacteria;class;Desulfuromonadales;order;Desulfuromonadaceae;family;Pelobacter;genus	90.51	9.00E-145	514
3177	0	0	1	0	0	0	0	0	1	Proteobacteria						S002349656 Desulfofrigus oceanense Lineage=Root;rootrank;Bacteria;domain;"Proteobacteria";phylum;unclassified_"Proteobacteria";	94.32	2.00E-160	566
765	0	0	0	0	2	0	0	0	0	Proteobacteria						S002441585 Desulfobacca acetoxidans DSM 11109 Lineage=Root;rootrank;Bacteria;domain;"Proteobacteria";phylum;Deltaproteobacteria;class;Syntrophobacterales;order;Syntrophaceae;family;Desulfobacca;genus	85.98	9.00E-110	398
261	0	1	0	0	0	0	0	0	0	Proteobacteria						S003263891 Sneathiella chinensis; NBRC 103408 Lineage=Root;rootrank;Bacteria;domain;"Proteobacteria";phylum;Alphaproteobacteria;class;Sneathiellales;order;Sneathiellaceae;family;Sneathiella;genus	86.77	7.00E-121	435
3001	0	0	0	1	0	0	0	0	0	Proteobacteria						S003288459 Sorangium cellulosum; x3t8 Lineage=Root;rootrank;Bacteria;domain;"Proteobacteria";phylum;Deltaproteobacteria;class;Myxococcales;order;Sorangiineae;suborder;unclassified_Sorangiineae;	89.36	1.00E-132	473
1560	0	0	0	3	0	0	0	0	0	Proteobacteria						S003610437 Pelobacter carbinolicus (T); DSM 2380 Lineage=Root;rootrank;Bacteria;domain;"Proteobacteria";phylum;Deltaproteobacteria;class;Desulfuromonadales;order;Desulfuromonadaceae;family;Pelobacter;genus	88.58	2.00E-132	473
2745	0	0	1	0	0	0	0	14	9	SBR093						S000996514 Thermodesulfatator atlanticus (T); AT1325 Lineage=Root;rootrank;Bacteria;domain;"Thermodesulfobacteria";phylum;Thermodesulfobacteria;class;Thermodesulfobacteriales;order;Thermodesulfobacteriaceae;family;Thermodesulfatator;genus	86.25	9.00E-110	398
2865	0	0	0	1	0	0	0	0	0	SBR093	VHS-B5-50					S000130733 Caldicellulosiruptor kristjanssonii (T); I77R1B Lineage=Root;rootrank;Bacteria;domain;Firmicutes;phylum;Clostridia;class;Clostridiales;order;Clostridiales_Incertae Sedis III;family;Caldicellulosiruptor;genus	84.45	1.00E-98	361
472	0	0	0	2	0	3	0	1	1	SBR093	VHS-B5-50					S000414361 Thermoanaerobacter cellulolyticus; NA 10B Lineage=Root;rootrank;Bacteria;domain;Firmicutes;phylum;Clostridia;class;Clostridiales;order;Clostridiales_Incertae Sedis III;family;Caldicellulosiruptor;genus	85.05	4.00E-108	392
1467	0	0	0	0	0	1	0	0	0	SBR093						S000965423 Natranaerobius thermophilus; JW/WN-NM-LF2 Lineage=Root;rootrank;Bacteria;domain;Firmicutes;phylum;Clostridia;class;Natranaerobiales;order;Natranaerobiaceae;family;Natranaerobius;genus	85.22	1.00E-103	377
1323	0	0	0	26	1	2	1	29	38	SPAM	039-6G9					S000428939 Ruminococcus flavefaciens; AR72 Lineage=Root;rootrank;Bacteria;domain;Firmicutes;phylum;Clostridia;class;Clostridiales;order;Ruminococcaceae;family;Ruminococcus;genus	84.05	7.00E-96	351
2152	0	0	1	18	5	53	46	46	67	SPAM	039-6G9					S001152076 Thermaerobacter composti (T); Ni80 Lineage=Root;rootrank;Bacteria;domain;Firmicutes;phylum;Clostridia;class;Clostridiales;order;Clostridiales_Incertae Sedis XVII;family;Thermaerobacter;genus	85.07	1.00E-102	374
2500	0	0	0	1	0	1	0	0	0	SPAM	039-6G9					S001152076 Thermaerobacter composti (T); Ni80 Lineage=Root;rootrank;Bacteria;domain;Firmicutes;phylum;Clostridia;class;Clostridiales;order;Clostridiales_Incertae Sedis XVII;family;Thermaerobacter;genus	85.71	2.00E-112	407
1416	0	1	11	48	63	213	134	77	92	SPAM	039-6G9					S002289633 Elusimicrobium minutum Pei191 Lineage=Root;rootrank;Bacteria;domain;"Elusimicrobia";phylum;Elusimicrobia;class;Elusimicrobiales;order;Elusimicrobiaceae;family;Elusimicrobium;genus	83.74	9.00E-95	348
97	0	0	1	2	2	0	0	9	1	SPAM	FFCH6980					S000130885 Thermoleophilum album (T); type strain: ATCC 35263 Lineage=Root;rootrank;Bacteria;domain;"Actinobacteria";phylum;Actinobacteria;class;Rubrobacteridae;subclass;Thermoleophilales;order;Thermoleophilaceae;family;Thermoleophilum;genus	86.99	2.00E-121	436
1372	0	0	1	2	0	1	0	0	1	SPAM	FFCH6980					S002224490 Chondromyces pediculatus; Cm p51 Lineage=Root;rootrank;Bacteria;domain;"Proteobacteria";phylum;Deltaproteobacteria;class;Myxococcales;order;Sorangiineae;suborder;Polyangiaceae;family;Chondromyces;genus	89.62	3.00E-129	462
233	0	0	1	29	9	0	0	1	1	SPAM	FFCH6980					S002950314 Thermodesulfatator indicus DSM 15286 Lineage=Root;rootrank;Bacteria;domain;"Thermodesulfobacteria";phylum;Thermodesulfobacteria;class;Thermodesulfobacteriales;order;Thermodesulfobacteriaceae;family;Thermodesulfatator;genus	87.37	1.00E-117	424
1574	0	0	0	0	0	0	0	0	2	SPAM						S000539475 Methylocaldum szegediense; KTM-1 Lineage=Root;rootrank;Bacteria;domain;"Proteobacteria";phylum;Gammaproteobacteria;class;Methylococcales;order;Methylococcaceae;family;Methylocaldum;genus	86.63	1.00E-113	411
1550	0	0	1	0	0	0	0	0	0	Tenericutes	ML65J-28					S001095420 Haloplasma contractile (T); SSD-17B Lineage=Root;rootrank;Bacteria;domain;"Tenericutes";phylum;Mollicutes;class;Haloplasmatales;order;Haloplasmataceae;family;Haloplasma;genus	88.08	1.00E-127	457
1639	0	0	0	0	1	0	0	0	0	Tenericutes	ML65J-28					S001095420 Haloplasma contractile (T); SSD-17B Lineage=Root;rootrank;Bacteria;domain;"Tenericutes";phylum;Mollicutes;class;Haloplasmatales;order;Haloplasmataceae;family;Haloplasma;genus	88.17	3.00E-129	462
3016	0	1	0	0	0	0	0	0	0	Tenericutes	ML65J-28					S002444921 Turicibacter sanguinis; 111732/2010 Lineage=Root;rootrank;Bacteria;domain;Firmicutes;phylum;Erysipelotrichia;class;Erysipelotrichales;order;Erysipelotrichaceae;family;Turicibacter;genus	88.63	2.00E-131	470
2227	0	1	1	0	1	0	0	0	0	TG3	TG3-	GN09				S002441480 Hippea maritima DSM 10411 Lineage=Root;rootrank;Bacteria;domain;"Proteobacteria";phylum;Deltaproteobacteria;class;Desulfurellales;order;Desulfurellaceae;family;Hippea;genus	82.62	7.00E-86	318
476	0	0	0	0	1	0	0	0	0	TG3						S000414642 Bacteroides cellulosolvens Lineage=Root;rootrank;Bacteria;domain;Firmicutes;phylum;Clostridia;class;Clostridiales;order;Ruminococcaceae;family;Clostridium III;genus	84.36	3.00E-104	379
3252	123	700	125	3	94	74	87	22	6	Thermi	Deinococci	Deinococcales	Deinococcaceae		Deinococcus	S002291110 Truepera radiovictrix DSM 17093 Lineage=Root;rootrank;Bacteria;domain;"Deinococcus-Thermus";phylum;Deinococci;class;Deinococcales;order;Trueperaceae;family;Truepera;genus	91.89	8.00E-145	514
1174	43	69	141	60	149	47	57	237	202	Thermi	Deinococci	Deinococcales	Deinococcaceae		Deinococcus	S002291110 Truepera radiovictrix DSM 17093 Lineage=Root;rootrank;Bacteria;domain;"Deinococcus-Thermus";phylum;Deinococci;class;Deinococcales;order;Trueperaceae;family;Truepera;genus	91.03	3.00E-139	496
3005	73	109	52	11	30	17	49	74	46	Thermi	Deinococci	Deinococcales	Deinococcaceae		Deinococcus	S002291110 Truepera radiovictrix DSM 17093 Lineage=Root;rootrank;Bacteria;domain;"Deinococcus-Thermus";phylum;Deinococci;class;Deinococcales;order;Trueperaceae;family;Truepera;genus	91.06	8.00E-140	497
1247	1	0	3	40	10	2	0	41	38	Thermi	Deinococci	Deinococcales	Deinococcaceae		Deinococcus	S002291110 Truepera radiovictrix DSM 17093 Lineage=Root;rootrank;Bacteria;domain;"Deinococcus-Thermus";phylum;Deinococci;class;Deinococcales;order;Trueperaceae;family;Truepera;genus	94.02	4.00E-158	558
136	0	0	0	5	0	0	0	0	0	Thermi	Deinococci	Deinococcales	Deinococcaceae		Deinococcus	S002291110 Truepera radiovictrix DSM 17093 Lineage=Root;rootrank;Bacteria;domain;"Deinococcus-Thermus";phylum;Deinococci;class;Deinococcales;order;Trueperaceae;family;Truepera;genus	91.26	1.00E-148	527
2195	0	0	0	0	0	1	2	0	0	Thermi	Deinococci	Deinococcales	Deinococcaceae		Deinococcus	S002291110 Truepera radiovictrix DSM 17093 Lineage=Root;rootrank;Bacteria;domain;"Deinococcus-Thermus";phylum;Deinococci;class;Deinococcales;order;Trueperaceae;family;Truepera;genus	89.43	8.00E-130	464
86	0	0	0	0	0	0	0	2	1	Thermi	Deinococci	Deinococcales	Deinococcaceae		Deinococcus	S002291110 Truepera radiovictrix DSM 17093 Lineage=Root;rootrank;Bacteria;domain;"Deinococcus-Thermus";phylum;Deinococci;class;Deinococcales;order;Trueperaceae;family;Truepera;genus	91.89	8.00E-145	514
2542	0	0	0	0	0	0	0	0	2	Thermi	Deinococci	Deinococcales	Deinococcaceae		Deinococcus	S002291110 Truepera radiovictrix DSM 17093 Lineage=Root;rootrank;Bacteria;domain;"Deinococcus-Thermus";phylum;Deinococci;class;Deinococcales;order;Trueperaceae;family;Truepera;genus	91.58	4.00E-143	508
1450	0	1	0	0	0	0	0	0	0	Thermi	Deinococci	Deinococcales	Deinococcaceae		Deinococcus	S002291110 Truepera radiovictrix DSM 17093 Lineage=Root;rootrank;Bacteria;domain;"Deinococcus-Thermus";phylum;Deinococci;class;Deinococcales;order;Trueperaceae;family;Truepera;genus	82.62	2.00E-86	320
51	0	0	0	0	0	0	1	0	0	Thermi	Deinococci	Deinococcales	Deinococcaceae		Deinococcus	S002291110 Truepera radiovictrix DSM 17093 Lineage=Root;rootrank;Bacteria;domain;"Deinococcus-Thermus";phylum;Deinococci;class;Deinococcales;order;Trueperaceae;family;Truepera;genus	90.96	2.00E-146	520
1564	0	0	0	0	0	0	0	0	1	Thermi	Deinococci	Deinococcales	Deinococcaceae		Deinococcus	S002291110 Truepera radiovictrix DSM 17093 Lineage=Root;rootrank;Bacteria;domain;"Deinococcus-Thermus";phylum;Deinococci;class;Deinococcales;order;Trueperaceae;family;Truepera;genus	85.79	2.00E-112	407
1272	0	0	0	1	0	0	0	0	0	TM6	SBRH58					S000769893 Sinobacter flavus (T); CW-KD 4 Lineage=Root;rootrank;Bacteria;domain;"Proteobacteria";phylum;Gammaproteobacteria;class;Xanthomonadales;order;Sinobacteraceae;family;Sinobacter;genus	81.57	9.00E-80	298
3039	4	0	0	0	0	0	0	0	0	TM6	SJA-4					S000023633 Vesicomya chordata gill symbiont Lineage=Root;rootrank;Bacteria;domain;"Proteobacteria";phylum;Gammaproteobacteria;class;unclassified_Gammaproteobacteria;	82.53	5.00E-87	322
1922	0	0	0	0	1	0	0	0	0	TM6	SJA-4					S000131201 Nitrosococcus mobilis; Nc2 Lineage=Root;rootrank;Bacteria;domain;"Proteobacteria";phylum;Betaproteobacteria;class;Nitrosomonadales;order;Nitrosomonadaceae;family;Nitrosomonas;genus	82.41	6.00E-92	339
115	0	0	0	0	1	0	0	1	1	TM6	SJA-4					S000425966 Legionella-like amoebal pathogen HT99 Lineage=Root;rootrank;Bacteria;domain;"Proteobacteria";phylum;Gammaproteobacteria;class;unclassified_Gammaproteobacteria;	82.23	2.00E-85	316
1909	0	0	0	0	0	0	0	1	0	TM6	SJA-4					S000446585 Fulvimonas soli (T); type strain: LMG 19981 Lineage=Root;rootrank;Bacteria;domain;"Proteobacteria";phylum;Gammaproteobacteria;class;Xanthomonadales;order;Xanthomonadaceae;family;Fulvimonas;genus	81.42	1.00E-83	311
3134	0	0	0	0	0	0	1	2	0	TM6	SJA-4					S000547278 Hydrocarboniphaga effusa (T); AP103; ATCC BAA-332 Lineage=Root;rootrank;Bacteria;domain;"Proteobacteria";phylum;Gammaproteobacteria;class;Xanthomonadales;order;Sinobacteraceae;family;Hydrocarboniphaga;genus	81.4	1.00E-78	294
2988	2	0	5	0	1	2	0	0	0	TM6	SJA-4					S000548164 Aquimonas voraii (T); GPTSA 20 Lineage=Root;rootrank;Bacteria;domain;"Proteobacteria";phylum;Gammaproteobacteria;class;Xanthomonadales;order;Xanthomonadaceae;family;Aquimonas;genus	82.43	9.00E-85	315
1675	1	0	0	0	0	4	3	0	0	TM6	SJA-4					S000548164 Aquimonas voraii (T); GPTSA 20 Lineage=Root;rootrank;Bacteria;domain;"Proteobacteria";phylum;Gammaproteobacteria;class;Xanthomonadales;order;Xanthomonadaceae;family;Aquimonas;genus	81.69	7.00E-81	302
2350	0	0	0	0	0	0	0	1	0	TM6	SJA-4					S000548164 Aquimonas voraii (T); GPTSA 20 Lineage=Root;rootrank;Bacteria;domain;"Proteobacteria";phylum;Gammaproteobacteria;class;Xanthomonadales;order;Xanthomonadaceae;family;Aquimonas;genus	81.61	8.00E-86	318
1297	0	0	0	0	1	0	0	0	0	TM6	SJA-4					S000622960 endosymbiont of Ifremeria nautilei Lineage=Root;rootrank;Bacteria;domain;"Proteobacteria";phylum;Gammaproteobacteria;class;Gammaproteobacteria_incertae_sedis;order;Thioprofundum;genus	79.52	2.00E-67	257
2470	0	9	6	2	3	20	19	2	9	TM6	SJA-4					S000768423 Rhodanobacter thiooxydans (T); LCS2 Lineage=Root;rootrank;Bacteria;domain;"Proteobacteria";phylum;Gammaproteobacteria;class;Xanthomonadales;order;Xanthomonadaceae;family;Rhodanobacter;genus	81.45	2.00E-77	291
455	19	9	14	0	4	2	8	0	1	TM6	SJA-4					S000769243 Solimonas soli; DCY15 Lineage=Root;rootrank;Bacteria;domain;"Proteobacteria";phylum;Gammaproteobacteria;class;Gammaproteobacteria_incertae_sedis;order;Solimonas;genus	83.42	7.00E-91	335
1282	0	0	0	0	0	1	0	0	0	TM6	SJA-4					S000842462 Moraxella ovis; ATCC 33078 Lineage=Root;rootrank;Bacteria;domain;"Proteobacteria";phylum;Gammaproteobacteria;class;Pseudomonadales;order;Moraxellaceae;family;Moraxella;genus	82.45	2.00E-85	316
1358	0	0	0	0	0	0	0	0	2	TM6	SJA-4					S000842867 Xylella fastidiosa; Cafe20-11 Lineage=Root;rootrank;Bacteria;domain;"Proteobacteria";phylum;Gammaproteobacteria;class;Xanthomonadales;order;Xanthomonadaceae;family;Xylella;genus	82.14	3.00E-89	329
1248	0	0	1	0	0	0	0	0	2	TM6	SJA-4					S000859363 Legionella-like amoebal pathogen CC99 Lineage=Root;rootrank;Bacteria;domain;"Proteobacteria";phylum;Gammaproteobacteria;class;unclassified_Gammaproteobacteria;	83.25	6.00E-97	355
596	0	0	0	0	0	0	0	0	2	TM6	SJA-4					S000859363 Legionella-like amoebal pathogen CC99 Lineage=Root;rootrank;Bacteria;domain;"Proteobacteria";phylum;Gammaproteobacteria;class;unclassified_Gammaproteobacteria;	81.73	7.00E-86	318
1739	0	0	0	1	0	0	0	0	0	TM6	SJA-4					S000859363 Legionella-like amoebal pathogen CC99 Lineage=Root;rootrank;Bacteria;domain;"Proteobacteria";phylum;Gammaproteobacteria;class;unclassified_Gammaproteobacteria;	80.38	2.00E-72	274
1732	0	0	0	0	0	1	0	0	0	TM6	SJA-4					S000859363 Legionella-like amoebal pathogen CC99 Lineage=Root;rootrank;Bacteria;domain;"Proteobacteria";phylum;Gammaproteobacteria;class;unclassified_Gammaproteobacteria;	81.28	3.00E-84	313
651	0	0	0	2	1	0	3	2	19	TM6	SJA-4					S000871452 Dokdonella koreensis; NML 01-0233 Lineage=Root;rootrank;Bacteria;domain;"Proteobacteria";phylum;Gammaproteobacteria;class;Xanthomonadales;order;Xanthomonadaceae;family;Dokdonella;genus	82.43	3.00E-84	313
1627	0	1	0	5	0	3	2	0	2	TM6	SJA-4					S000871452 Dokdonella koreensis; NML 01-0233 Lineage=Root;rootrank;Bacteria;domain;"Proteobacteria";phylum;Gammaproteobacteria;class;Xanthomonadales;order;Xanthomonadaceae;family;Dokdonella;genus	83.7	1.00E-92	340
16	2	9	0	0	0	0	1	0	0	TM6	SJA-4					S000891930 Methylonatrum kenyense (T); AMT 1 Lineage=Root;rootrank;Bacteria;domain;"Proteobacteria";phylum;Gammaproteobacteria;class;Gammaproteobacteria_incertae_sedis;order;Methylonatrum;genus	82.4	2.00E-92	340
552	0	0	0	0	0	0	1	4	0	TM6	SJA-4					S000891930 Methylonatrum kenyense (T); AMT 1 Lineage=Root;rootrank;Bacteria;domain;"Proteobacteria";phylum;Gammaproteobacteria;class;Gammaproteobacteria_incertae_sedis;order;Methylonatrum;genus	81.5	5.00E-82	305
864	0	0	0	0	0	0	0	1	2	TM6	SJA-4					S000891930 Methylonatrum kenyense (T); AMT 1 Lineage=Root;rootrank;Bacteria;domain;"Proteobacteria";phylum;Gammaproteobacteria;class;Gammaproteobacteria_incertae_sedis;order;Methylonatrum;genus	82.79	1.00E-87	324
89	0	0	0	0	0	0	0	0	2	TM6	SJA-4					S000891930 Methylonatrum kenyense (T); AMT 1 Lineage=Root;rootrank;Bacteria;domain;"Proteobacteria";phylum;Gammaproteobacteria;class;Gammaproteobacteria_incertae_sedis;order;Methylonatrum;genus	82.61	4.00E-93	342
2056	0	0	0	1	0	0	0	0	0	TM6	SJA-4					S000891930 Methylonatrum kenyense (T); AMT 1 Lineage=Root;rootrank;Bacteria;domain;"Proteobacteria";phylum;Gammaproteobacteria;class;Gammaproteobacteria_incertae_sedis;order;Methylonatrum;genus	81.63	2.00E-87	324
2815	0	0	0	0	0	12	22	0	0	TM6	SJA-4					S001046429 Rudaea cellulosilytica (T); KIS3-4 Lineage=Root;rootrank;Bacteria;domain;"Proteobacteria";phylum;Gammaproteobacteria;class;Xanthomonadales;order;Xanthomonadaceae;family;Rudaea;genus	81.89	1.00E-82	307
2103	4	8	1	0	0	2	6	4	1	TM6	SJA-4					S001152221 Candidatus Legionella jeonii Lineage=Root;rootrank;Bacteria;domain;"Proteobacteria";phylum;Gammaproteobacteria;class;Legionellales;order;Legionellaceae;family;Legionella;genus	81.17	5.00E-77	289
2434	0	1	0	0	0	0	0	0	0	TM6	SJA-4					S001154924 Moraxellaceae Bacterium MAG Lineage=Root;rootrank;Bacteria;domain;"Proteobacteria";phylum;Gammaproteobacteria;class;Pseudomonadales;order;Moraxellaceae;family;Acinetobacter;genus	82.16	4.00E-88	326
2486	0	0	0	1	0	0	0	0	0	TM6	SJA-4					S001264367 Dyella yeojuensis; rif200829 Lineage=Root;rootrank;Bacteria;domain;"Proteobacteria";phylum;Gammaproteobacteria;class;Xanthomonadales;order;Xanthomonadaceae;family;Luteibacter;genus	81.27	5.00E-83	309
309	0	0	0	0	0	0	0	1	0	TM6	SJA-4					S001331314 Desulfobotulus alkaliphilus (T); ASO4-4; DSM 22078 Lineage=Root;rootrank;Bacteria;domain;"Proteobacteria";phylum;Deltaproteobacteria;class;Desulfobacterales;order;Desulfobacteraceae;family;Desulfobotulus;genus	81.7	2.00E-82	307
1502	0	0	0	1	0	0	0	0	0	TM6	SJA-4					S001417995 Acidithiobacillus ferrooxidans; SY1 Lineage=Root;rootrank;Bacteria;domain;"Proteobacteria";phylum;Gammaproteobacteria;class;Acidithiobacillales;order;Acidithiobacillaceae;family;Acidithiobacillus;genus	80.38	1.00E-73	278
567	0	0	0	1	0	0	0	0	0	TM6	SJA-4					S001587468 Nevskia ramosa; MAFF 211643 Lineage=Root;rootrank;Bacteria;domain;"Proteobacteria";phylum;Gammaproteobacteria;class;Xanthomonadales;order;Sinobacteraceae;family;Nevskia;genus	81.35	1.00E-78	294
1433	0	0	0	0	0	0	1	0	0	TM6	SJA-4					S001587468 Nevskia ramosa; MAFF 211643 Lineage=Root;rootrank;Bacteria;domain;"Proteobacteria";phylum;Gammaproteobacteria;class;Xanthomonadales;order;Sinobacteraceae;family;Nevskia;genus	80.49	1.00E-73	278
774	0	0	0	0	0	0	0	1	0	TM6	SJA-4					S001612658 Nevskia terrae (T); KIS13-15 Lineage=Root;rootrank;Bacteria;domain;"Proteobacteria";phylum;Gammaproteobacteria;class;Xanthomonadales;order;Sinobacteraceae;family;Nevskia;genus	82.29	3.00E-84	313
385	0	0	0	0	0	0	0	0	1	TM6	SJA-4					S001873794 Thiothrix fructosivorans (T); Q Lineage=Root;rootrank;Bacteria;domain;"Proteobacteria";phylum;Gammaproteobacteria;class;Thiotrichales;order;Thiotrichaceae;family;Thiothrix;genus	80.2	1.00E-74	281
2953	8	322	44	0	7	2	8	5	7	TM6	SJA-4					S002166186 Helicobacter marmotae; MIT 04-8589 Lineage=Root;rootrank;Bacteria;domain;"Proteobacteria";phylum;Epsilonproteobacteria;class;Campylobacterales;order;Helicobacteraceae;family;Helicobacter;genus	80.26	3.00E-70	267
688	0	0	1	0	0	0	0	0	0	TM6	SJA-4					S002355520 Polycyclovorans algicola; TG408 Lineage=Root;rootrank;Bacteria;domain;"Proteobacteria";phylum;Gammaproteobacteria;class;Xanthomonadales;order;Sinobacteraceae;family;Singularimonas;genus	82.21	1.00E-83	311
2515	0	0	0	0	0	0	0	0	1	TM6	SJA-4					S002449003 Luteibacter anthropi; NSBm.5(AN7) Lineage=Root;rootrank;Bacteria;domain;"Proteobacteria";phylum;Gammaproteobacteria;class;Xanthomonadales;order;Xanthomonadaceae;family;Luteibacter;genus	82.23	3.00E-89	329
1330	0	0	0	1	0	0	0	0	0	TM6	SJA-4					S003262299 Frateuria aurantia; NBRC 13330 Lineage=Root;rootrank;Bacteria;domain;"Proteobacteria";phylum;Gammaproteobacteria;class;Xanthomonadales;order;Xanthomonadaceae;family;Frateuria;genus	79.46	2.00E-66	254
377	0	0	1	0	0	0	1	1	0	TM6	SJA-4					S003262529 Moraxella atlantae; NBRC 14588 Lineage=Root;rootrank;Bacteria;domain;"Proteobacteria";phylum;Gammaproteobacteria;class;Pseudomonadales;order;Moraxellaceae;family;Moraxella;genus	82.78	4.00E-93	342
2730	0	0	1	0	0	0	0	0	0	TM6	SJA-4					S003264052 Hydrocarboniphaga daqingensis; NBRC 104238 Lineage=Root;rootrank;Bacteria;domain;"Proteobacteria";phylum;Gammaproteobacteria;class;Xanthomonadales;order;Sinobacteraceae;family;Hydrocarboniphaga;genus	81.31	5.00E-83	309
141	0	0	0	0	0	0	1	0	0	TM6	SJA-4					S003264052 Hydrocarboniphaga daqingensis; NBRC 104238 Lineage=Root;rootrank;Bacteria;domain;"Proteobacteria";phylum;Gammaproteobacteria;class;Xanthomonadales;order;Sinobacteraceae;family;Hydrocarboniphaga;genus	82.65	6.00E-92	339
632	0	0	0	0	0	0	0	0	1	TM6	SJA-4					S003264308 Nevskia soli; NBRC 106401 Lineage=Root;rootrank;Bacteria;domain;"Proteobacteria";phylum;Gammaproteobacteria;class;Xanthomonadales;order;Sinobacteraceae;family;Nevskia;genus	81.25	4.00E-78	292
2202	0	0	1	1	0	0	0	0	0	TM6	SJA-4					S003287785 Psychrobacter fozii; Kongs-24 Lineage=Root;rootrank;Bacteria;domain;"Proteobacteria";phylum;Gammaproteobacteria;class;Pseudomonadales;order;Moraxellaceae;family;Psychrobacter;genus	80.75	4.00E-79	296
2816	0	0	0	0	1	0	0	4	4	TM6	SJA-4					S003288146 Nitrosomonas communis; Nm2 Lineage=Root;rootrank;Bacteria;domain;"Proteobacteria";phylum;Betaproteobacteria;class;Nitrosomonadales;order;Nitrosomonadaceae;family;Nitrosomonas;genus	79.68	6.00E-67	255
545	0	0	0	0	0	0	0	0	1	TM6	SJA-4					S003291672 Acinetobacter nectaris; SAP 971.1 Lineage=Root;rootrank;Bacteria;domain;"Proteobacteria";phylum;Gammaproteobacteria;class;Pseudomonadales;order;Moraxellaceae;family;Acinetobacter;genus	83.96	3.00E-99	363
1587	4	1	1	1	2	9	3	2	3	TM6	SJA-4					S003615834 Acidithiobacillus ferrooxidans; 9/11 Lineage=Root;rootrank;Bacteria;domain;"Proteobacteria";phylum;Gammaproteobacteria;class;Acidithiobacillales;order;Acidithiobacillaceae;family;Acidithiobacillus;genus	81.11	2.00E-81	303
1782	0	11	2	0	0	0	0	0	0	TM6	SJA-4					S003617106 Psychrobacter celer; MN5SW1 Lineage=Root;rootrank;Bacteria;domain;"Proteobacteria";phylum;Gammaproteobacteria;class;Pseudomonadales;order;Moraxellaceae;family;Psychrobacter;genus	80.8	3.00E-75	283
2797	1	0	2	4	2	1	0	0	0	TM6	SJA-4					S003713756 Dyella marensis; CS8G3-6 Lineage=Root;rootrank;Bacteria;domain;"Proteobacteria";phylum;Gammaproteobacteria;class;Xanthomonadales;order;Xanthomonadaceae;family;Dyella;genus	82.18	4.00E-83	309
171	0	0	0	0	0	0	0	0	1	TM6	SJA-4					S003746948 Acinetobacter radioresistens; Dst04 Lineage=Root;rootrank;Bacteria;domain;"Proteobacteria";phylum;Gammaproteobacteria;class;Pseudomonadales;order;Moraxellaceae;family;Acinetobacter;genus	81.11	6.00E-82	305
2723	0	0	0	0	0	0	0	0	1	TM6	SJA-4					S003748944 Stenotrophomonas chelatiphaga; CH1-19 Lineage=Root;rootrank;Bacteria;domain;"Proteobacteria";phylum;Gammaproteobacteria;class;Xanthomonadales;order;Xanthomonadaceae;family;Stenotrophomonas;genus	82.65	6.00E-92	339
2871	0	0	2	0	0	0	1	0	0	TM6	SJA-4					S003751039 Dyella ginsengisoli; ED50 Lineage=Root;rootrank;Bacteria;domain;"Proteobacteria";phylum;Gammaproteobacteria;class;Xanthomonadales;order;Xanthomonadaceae;family;Dyella;genus	81.3	3.00E-79	296
1010	0	0	0	1	0	1	0	0	0	TM6	SJA-4					S003753637 Acidithiobacillus ferrooxidans; MCF59 Lineage=Root;rootrank;Bacteria;domain;"Proteobacteria";phylum;Gammaproteobacteria;class;Acidithiobacillales;order;Acidithiobacillaceae;family;Acidithiobacillus;genus	82.65	2.00E-92	340
2490	0	0	0	1	0	0	0	0	0	TM6	SJA-4					S003753637 Acidithiobacillus ferrooxidans; MCF59 Lineage=Root;rootrank;Bacteria;domain;"Proteobacteria";phylum;Gammaproteobacteria;class;Acidithiobacillales;order;Acidithiobacillaceae;family;Acidithiobacillus;genus	82.95	1.00E-94	348
1969	0	0	0	4	10	0	0	0	2	TM7	MJK0					S003617966 endosymbiont of Ridgeia piscesae; LF8CM1b5 Lineage=Root;rootrank;Bacteria;domain;"Proteobacteria";phylum;Gammaproteobacteria;class;unclassified_Gammaproteobacteria;	83.63	1.00E-98	361
2840	0	0	0	0	0	0	1	0	0	TM7	MJK0					S003617966 endosymbiont of Ridgeia piscesae; LF8CM1b5 Lineage=Root;rootrank;Bacteria;domain;"Proteobacteria";phylum;Gammaproteobacteria;class;unclassified_Gammaproteobacteria;	83.7	1.00E-92	340
2036	3	0	0	0	0	0	0	0	0	TM7	TM7-					S000641663 Nemalionopsis tortuosa; SAG B 42.94 Lineage=Root;rootrank;Bacteria;domain;Cyanobacteria/Chloroplast;phylum;Chloroplast;class;Chloroplast;family;unclassified_Chloroplast;	80.3	2.00E-77	291
351	1	0	0	0	0	0	0	0	0	TM7	TM7-					S002304636 Dichotomicrobium thermohalophilum; type strain: DSM5002 Lineage=Root;rootrank;Bacteria;domain;"Proteobacteria";phylum;Alphaproteobacteria;class;Rhizobiales;order;unclassified_Rhizobiales;	82.59	1.00E-58	228
1275	0	0	0	1	0	0	0	0	0	TM7	TM7-3	I025	Rs-045			S000006983 Legionella londiniensis (T); NCTC 12374 = ATCC 49505 Lineage=Root;rootrank;Bacteria;domain;"Proteobacteria";phylum;Gammaproteobacteria;class;Legionellales;order;Legionellaceae;family;Legionella;genus	79.95	4.00E-68	259
2821	0	0	0	5	4	0	0	22	1	TM7	TM7-3	I025	Rs-045			S000410447 Desulfomicrobium thermophilum (T); P6.2 Lineage=Root;rootrank;Bacteria;domain;"Proteobacteria";phylum;Deltaproteobacteria;class;Desulfovibrionales;order;Desulfomicrobiaceae;family;Desulfomicrobium;genus	78.02	7.00E-56	219
585	0	0	2	1	2	1	0	17	1	TM7	TM7-3	I025	Rs-045			S000569921 Pelotomaculum isophthalicicum (T); JI Lineage=Root;rootrank;Bacteria;domain;Firmicutes;phylum;Clostridia;class;Clostridiales;order;Peptococcaceae 2;family;Pelotomaculum;genus	81.6	9.00E-80	298
1492	0	0	3	0	1	0	1	16	4	TM7	TM7-3	I025	Rs-045			S002440627 Clostridium tunisiense; N71 Lineage=Root;rootrank;Bacteria;domain;Firmicutes;phylum;Clostridia;class;Clostridiales;order;Clostridiaceae 1;family;Clostridium sensu stricto;genus	77.72	7.00E-56	219
958	0	0	0	0	0	0	1	0	0	TM7	TM7-3	I025	Rs-045			S003754531 Clostridium lundense; DTY1 Lineage=Root;rootrank;Bacteria;domain;Firmicutes;phylum;Clostridia;class;Clostridiales;order;Clostridiaceae 1;family;Clostridium sensu stricto;genus	80.14	2.00E-52	207
1576	0	0	0	0	1	0	0	0	2	TM7	TM7-3	I025	Rs-045			S003756855 Desulfovibrio hydrothermalis AM13 = DSM 14728 Lineage=Root;rootrank;Bacteria;domain;"Proteobacteria";phylum;Deltaproteobacteria;class;Desulfovibrionales;order;Desulfovibrionaceae;family;Desulfovibrio;genus	79.36	9.00E-65	248
2940	0	0	0	0	0	0	0	0	1	TM7						S000007073 Clostridium thermopalmarium; JW/JH-Fiji-1 Lineage=Root;rootrank;Bacteria;domain;Firmicutes;phylum;Clostridia;class;Clostridiales;order;Clostridiaceae 1;family;Clostridium sensu stricto;genus	80.65	2.00E-72	274
2457	0	1	0	1	0	0	0	0	0	TM7						S000010081 Desulfothermus naphthae (T); type strain: TD3 Lineage=Root;rootrank;Bacteria;domain;"Proteobacteria";phylum;Deltaproteobacteria;class;Desulfovibrionales;order;Desulfohalobiaceae;family;Desulfothermus;genus	79.78	1.00E-68	261
2551	0	0	1	0	0	0	0	0	1	TM7						S000438120 Rubrobacter radiotolerans (T); JCM 2153-T Lineage=Root;rootrank;Bacteria;domain;"Actinobacteria";phylum;Actinobacteria;class;Rubrobacteridae;subclass;Rubrobacterales;order;"Rubrobacterineae";suborder;Rubrobacteraceae;family;Rubrobacter;genus	81.23	6.00E-82	305
2015	0	0	0	0	1	0	0	0	0	TM7						S000496332 Nitratiruptor tergarcus (T); MI55-1 Lineage=Root;rootrank;Bacteria;domain;"Proteobacteria";phylum;Epsilonproteobacteria;class;Nautiliales;order;Nautiliaceae;family;Nitratiruptor;genus	82.7	7.00E-86	318
2942	0	0	0	0	0	0	0	0	1	TM7						S000576711 Clostridium lundense (T); DSM 17049 Lineage=Root;rootrank;Bacteria;domain;Firmicutes;phylum;Clostridia;class;Clostridiales;order;Clostridiaceae 1;family;Clostridium sensu stricto;genus	81.1	5.00E-77	289
1535	0	0	0	0	1	0	0	0	0	TM7						S000617673 Cyanoptyche gloeocystis; SAG 34.90 Lineage=Root;rootrank;Bacteria;domain;Cyanobacteria/Chloroplast;phylum;Chloroplast;class;Chloroplast;family;unclassified_Chloroplast;	86.48	3.00E-69	263
252	0	0	0	0	1	0	0	0	0	TM7						S000871061 Pelobacter seleniigenes (T); KM Lineage=Root;rootrank;Bacteria;domain;"Proteobacteria";phylum;Deltaproteobacteria;class;Desulfuromonadales;order;Desulfuromonadaceae;family;Pelobacter;genus	82.83	3.00E-94	346
1503	0	0	0	0	0	0	0	2	1	TM7						S000891994 Carboxydocella ferrireducens; 019 Lineage=Root;rootrank;Bacteria;domain;Firmicutes;phylum;Clostridia;class;Clostridiales;order;Clostridiales_Incertae Sedis XVI;family;Carboxydocella;genus	80.51	5.00E-78	292
1628	0	1	8	102	64	0	0	12	6	TM7						S002288919 Halothermothrix orenii H 168; H 168; DSM 9562 Lineage=Root;rootrank;Bacteria;domain;Firmicutes;phylum;Clostridia;class;Halanaerobiales;order;Halanaerobiaceae;family;Halothermothrix;genus	79.56	3.00E-65	250
2426	0	0	0	0	0	0	0	2	6	TM7						S002290544 Alkaliphilus metalliredigens QYMF Lineage=Root;rootrank;Bacteria;domain;Firmicutes;phylum;Clostridia;class;Clostridiales;order;Clostridiaceae 2;family;Alkaliphilus;genus	82.64	3.00E-90	333
2694	0	0	0	6	0	0	0	4	2	TM7						S002953916 Clostridium sardiniense; 235 Lineage=Root;rootrank;Bacteria;domain;Firmicutes;phylum;Clostridia;class;Clostridiales;order;Clostridiaceae 1;family;Clostridium sensu stricto;genus	81.43	9.00E-80	298
2817	0	1	10	0	2	0	3	2	2	TM7						S003264353 Rhodopseudomonas boonkerdii; NBRC 106595 Lineage=Root;rootrank;Bacteria;domain;"Proteobacteria";phylum;Alphaproteobacteria;class;Rhizobiales;order;Bradyrhizobiaceae;family;unclassified_Bradyrhizobiaceae;	81.27	6.00E-82	305
2257	0	3	0	0	0	0	0	4	1	TM7						S003299639 Clostridium colicanis; AB32 Lineage=Root;rootrank;Bacteria;domain;Firmicutes;phylum;Clostridia;class;Clostridiales;order;Clostridiaceae 1;family;Clostridium sensu stricto;genus	81.42	5.00E-77	289
118	0	0	3	0	3	0	0	1	3	TM7						S003617966 endosymbiont of Ridgeia piscesae; LF8CM1b5 Lineage=Root;rootrank;Bacteria;domain;"Proteobacteria";phylum;Gammaproteobacteria;class;unclassified_Gammaproteobacteria;	83.2	3.00E-89	329
75	0	0	0	0	1	0	0	0	0	TM7						S003617966 endosymbiont of Ridgeia piscesae; LF8CM1b5 Lineage=Root;rootrank;Bacteria;domain;"Proteobacteria";phylum;Gammaproteobacteria;class;unclassified_Gammaproteobacteria;	84.01	3.00E-94	346
2132	0	0	0	0	0	0	0	2	2	Verrucomicrobia	Opitutae					S000428476 Alterococcus agarolyticus (T); ADT3; BCRC17102 Lineage=Root;rootrank;Bacteria;domain;"Verrucomicrobia";phylum;Opitutae;class;Opitutales;order;Opitutaceae;family;Alterococcus;genus	94.37	2.00E-170	599
474	0	0	0	1	0	0	0	0	0	Verrucomicrobia	Opitutae					S000428476 Alterococcus agarolyticus (T); ADT3; BCRC17102 Lineage=Root;rootrank;Bacteria;domain;"Verrucomicrobia";phylum;Opitutae;class;Opitutales;order;Opitutaceae;family;Alterococcus;genus	95.38	5.00E-177	621
3157	0	0	0	0	0	0	1	0	0	Verrucomicrobia	Opitutae					S000428476 Alterococcus agarolyticus (T); ADT3; BCRC17102 Lineage=Root;rootrank;Bacteria;domain;"Verrucomicrobia";phylum;Opitutae;class;Opitutales;order;Opitutaceae;family;Alterococcus;genus	96.5	8.00E-175	614
1876	0	0	0	0	1	0	0	0	0	Verrucomicrobia	Opitutae	Opitutales	Opitutaceae			S002287922 Opitutus terrae PB90-1 Lineage=Root;rootrank;Bacteria;domain;"Verrucomicrobia";phylum;Opitutae;class;Opitutales;order;Opitutaceae;family;Opitutus;genus	95.4	1.00E-177	623

3010	0	0	0	1	0	0	0	0	0	Verrucomicrobia	Opitutae	Opitutales	Opitutaceae		S002287922 Opitutus terrae PB90-1 Lineage=Root;rootrank;Bacteria;domain;"Verrucomicrobia";phylum;Opitutae;class;Opitutales;order;Opitutaceae;family;Opitutus;genus	93.09	1.00E-162	573
735	0	0	0	4	1	1	0	0	0	Verrucomicrobia	Opitutae	Opitutales	Opitutaceae	Opitutus	S002287922 Opitutus terrae PB90-1 Lineage=Root;rootrank;Bacteria;domain;"Verrucomicrobia";phylum;Opitutae;class;Opitutales;order;Opitutaceae;family;Opitutus;genus	96.51	2.00E-175	616
2074	0	0	0	0	1	0	0	0	0	Verrucomicrobia	Opitutae	Opitutales	Opitutaceae	Opitutus	S002287922 Opitutus terrae PB90-1 Lineage=Root;rootrank;Bacteria;domain;"Verrucomicrobia";phylum;Opitutae;class;Opitutales;order;Opitutaceae;family;Opitutus;genus	95.15	7.00E-176	617
1748	0	0	5	0	0	1	1	0	1	Verrucomicrobia	Opitutae	Puniceicoccales	Puniceicoccaceae		S002290651 Coraliomargarita akajimensis DSM 45221 (T) Lineage=Root;rootrank;Bacteria;domain;"Verrucomicrobia";phylum;Opitutae;class;Puniceicoccales;order;Puniceicoccaceae;family;Coraliomargarita;genus	92.09	2.00E-156	553
2032	0	0	1	0	0	0	0	0	2	Verrucomicrobia	Opitutae	Puniceicoccales	Puniceicoccaceae		S002290651 Coraliomargarita akajimensis DSM 45221 (T) Lineage=Root;rootrank;Bacteria;domain;"Verrucomicrobia";phylum;Opitutae;class;Puniceicoccales;order;Puniceicoccaceae;family;Coraliomargarita;genus	87.67	2.00E-120	433
1534	0	0	0	0	1	0	0	0	0	Verrucomicrobia	Opitutae	Puniceicoccales	Puniceicoccaceae		S002290651 Coraliomargarita akajimensis DSM 45221 (T) Lineage=Root;rootrank;Bacteria;domain;"Verrucomicrobia";phylum;Opitutae;class;Puniceicoccales;order;Puniceicoccaceae;family;Coraliomargarita;genus	91.37	4.00E-143	508
142	0	0	0	1	0	0	0	0	5	Verrucomicrobia	Opitutae	Puniceicoccales	Puniceicoccaceae	Pelagicoccus	S000925467 Pelagicoccus croceus (T); N5FB36-5 Lineage=Root;rootrank;Bacteria;domain;"Verrucomicrobia";phylum;Opitutae;class;Puniceicoccales;order;Puniceicoccaceae;family;Pelagicoccus;genus	97.58	0	638
2594	1	0	1	2	1	0	0	4	2	Verrucomicrobia	Opitutae				S000428476 Alterococcus agarolyticus (T); ADT3; BCRC17102 Lineage=Root;rootrank;Bacteria;domain;"Verrucomicrobia";phylum;Opitutae;class;Opitutales;order;Opitutaceae;family;Alterococcus;genus	91.64	8.00E-145	514
2762	0	0	0	0	1	0	0	0	0	Verrucomicrobia	Verrucomicrobiae	Verrucomicrobiales			S000437977 Prosthecobacter dejongeii (T); FC1 Lineage=Root;rootrank;Bacteria;domain;"Verrucomicrobia";phylum;Verrucomicrobiae;class;Verrucomicrobiales;order;Verrucomicrobiaceae;family;Prosthecobacter;genus	84.85	9.00E-110	398
432	0	0	0	1	0	0	0	0	0	Verrucomicrobia	Verrucomicrobiae	Verrucomicrobiales			S000979025 Prosthecobacter debontii; DSM 14044 Lineage=Root;rootrank;Bacteria;domain;"Verrucomicrobia";phylum;Verrucomicrobiae;class;Verrucomicrobiales;order;Verrucomicrobiaceae;family;Prosthecobacter;genus	84.99	3.00E-109	396
1198	0	0	0	0	1	0	0	0	0	WPS-2					S002165556 Thermincola potens JR Lineage=Root;rootrank;Bacteria;domain;Firmicutes;phylum;Clostridia;class;Clostridiales;order;Peptococcaceae 1;family;Thermincola;genus	87.41	3.00E-124	446
905	0	0	0	4	4	2	0	0	2	WS3	PRR-2	CV06			S000569921 Pelotomaculum isophthalicicum (T); JI Lineage=Root;rootrank;Bacteria;domain;Firmicutes;phylum;Clostridia;class;Clostridiales;order;Peptococcaceae 2;family;Pelotomaculum;genus	85.44	2.00E-105	383
2631	0	0	0	1	1	0	0	0	0	WS3	PRR-2	Sediment-			S000550678 Moorella thermoacetica; AMP Lineage=Root;rootrank;Bacteria;domain;Firmicutes;phylum;Clostridia;class;Thermoanaerobacterales;order;Thermoanaerobacteraceae;family;Moorella;genus	85.41	2.00E-105	383
890	0	0	0	6	2	0	0	5	6	WS3	PRR-2	Sediment-			S001933336 Candidatus Methylomirabilis oxyfera Lineage=Root;rootrank;Bacteria;domain;unclassified_Bacteria;	86.65	2.00E-111	403
727	0	0	0	20	3	1	1	2	2	WS3	PRR-2	Sediment-	PRR-0		S003287266 Desulfomonile tiedjei DSM 6799 Lineage=Root;rootrank;Bacteria;domain;"Proteobacteria";phylum;Deltaproteobacteria;class;Syntrophobacterales;order;Syntrophaceae;family;Desulfomonile;genus	84.62	7.00E-106	385
1911	0	0	0	7	1	0	0	0	1	WS3	PRR-2	Sediment-	PRR-0		S003287266 Desulfomonile tiedjei DSM 6799 Lineage=Root;rootrank;Bacteria;domain;"Proteobacteria";phylum;Deltaproteobacteria;class;Syntrophobacterales;order;Syntrophaceae;family;Desulfomonile;genus	86.65	7.00E-111	401
813	0	0	0	3	0	0	1	0	3	WS3	PRR-2	Sediment-	PRR-0		S003287266 Desulfomonile tiedjei DSM 6799 Lineage=Root;rootrank;Bacteria;domain;"Proteobacteria";phylum;Deltaproteobacteria;class;Syntrophobacterales;order;Syntrophaceae;family;Desulfomonile;genus	87.23	3.00E-114	412
2310	0	0	0	1	0	0	0	0	0	WS3	PRR-2	Sediment-	PRR-0		S003287266 Desulfomonile tiedjei DSM 6799 Lineage=Root;rootrank;Bacteria;domain;"Proteobacteria";phylum;Deltaproteobacteria;class;Syntrophobacterales;order;Syntrophaceae;family;Desulfomonile;genus	87.21	2.00E-122	440
2868	0	0	0	1	0	0	0	0	0	WS3	PRR-2	Sediment-	PRR-0		S003287266 Desulfomonile tiedjei DSM 6799 Lineage=Root;rootrank;Bacteria;domain;"Proteobacteria";phylum;Deltaproteobacteria;class;Syntrophobacterales;order;Syntrophaceae;family;Desulfomonile;genus	85.29	1.00E-102	374
592	0	0	0	0	1	0	0	0	0	WS3	PRR-2	wb_H			S001933336 Candidatus Methylomirabilis oxyfera Lineage=Root;rootrank;Bacteria;domain;unclassified_Bacteria;	85.87	2.00E-106	387
2061	0	0	0	1	0	0	0	0	0	WS3	PRR-2				S001933336 Candidatus Methylomirabilis oxyfera Lineage=Root;rootrank;Bacteria;domain;unclassified_Bacteria;	87.24	2.00E-122	440
1018	0	0	0	0	0	0	0	1	0	WS6					S000018082 Gram-positive bacteria SOGA31 Lineage=Root;rootrank;Bacteria;domain;unclassified_Bacteria;	80.6	2.00E-72	274
583	0	0	0	1	0	1	1	0	0	WS6					S000540437 Bacteriovorax marinus; SJ Lineage=Root;rootrank;Bacteria;domain;"Proteobacteria";phylum;Deltaproteobacteria;class;Bdellovibrionales;order;Bacteriovoracaceae;family;Bacteriovorax;genus	78.66	5.00E-63	243
3109	0	0	0	3	0	0	0	0	0	WS6					S003617966 endosymbiont of Ridgeia piscesae; LF8CM1b5 Lineage=Root;rootrank;Bacteria;domain;"Proteobacteria";phylum;Gammaproteobacteria;class;unclassified_Gammaproteobacteria;	78.52	2.00E-62	241
