# Supplementary material for: Microbial Community Structure of Relict Niter-Beds Previously Used for Saltpeter Production
Source: PLoS One. 2014 Aug 11;9(8):e104752. doi: 10.1371/journal.pone.0104752 (PMC4128746; doi:10.1371/journal.pone.0104752)
Supplement: Table S5 — The relative abundances of ammonia-oxidizing bacteria and archaea in 16S rRNA gene pyrotag libraries. (PDF) [file pone.0104752.s010.pdf]

Table S5. The relative abundances of ammonia-oxidizing bacteria and archaea in 16S rRNA gene pyrotag libraries.

OTU ID	Relative abundance (%) in the sample:									Phylogeny assigned within:					BLAST analysis			
	OVA1	OVA2	OVA3	OVA4	OVA5	OVB2	OVB3	OVC1	OVC2	Phylum	Class	Order	Family	Genus	Closest relatives	Identity (%)	e-value	Bit score
**Ammonia-oxidizing bacteria (AOB)**																		
2435	0.013	0.103	0.048	0	0.006	0.067	0.062	0.060	0	*Proteobacteria*	*Gammaproteobacteria*	*Chromatiales*	*Chromatiaceae*	*Nitrosococcus*	*Nitrosococcus halophilus* Nc4	94.4	6.00E-160	572
2701	0.013	0.023	0	0	0	0	0	0	0	*Proteobacteria*	*Gammaproteobacteria*	*Chromatiales*	*Chromatiaceae*	*Nitrosococcus*	*Nitrosococcus watsoni* C-113	94.1	2.00E-159	571
1267	0	0	0	0.005	0	0.010	0.004	0	0	*Proteobacteria*	*Gammaproteobacteria*	*Chromatiales*	*Chromatiaceae*	*Nitrosococcus*	*Nitrosococcus watsoni* C-113	95.4	7.00E-166	592
2080	0	0	0.006	0.005	0	0	0	0	0	*Proteobacteria*	*Betaproteobacteria*	*Nitrosomonadales*	*Nitrosomonadaceae*	*Nitrosomonas*	*Nitrosomonas* sp. Nm59	98.1	9.00E-177	628
673	0	0	0.012	0.784	0.250	0.010	0.009	0.350	0.010	*Proteobacteria*	*Betaproteobacteria*	N.A.[a]	N.A.	N.A.	*Nitrosospira briensis* C-128	100	0	668
Total	0.026	0.126	0.066	0.794	0.255	0.088	0.075	0.410	0.010									
**Ammonia-oxidizing archaea (AOA)**																		
1897	0	0.034	0	0.021	0.017	0.026	0.026	0.035	0.066	*Crenarchaeota*	*Thaumarchaeota*	*Nitrososphaerales*	*Nitrososphaeraceae*	'*Ca*. Nitrososphaera'	*Nitrososphaera* sp. JG1	95.2	7.00E-166	592
1946	0	0	0.018	0.047	0.044	0.036	0	0.017	0.038	*Crenarchaeota*	*Thaumarchaeota*	*Nitrososphaerales*	*Nitrososphaeraceae*	'*Ca*. Nitrososphaera'	*Nitrososphaera viennensis* EN76	98.7	0.00E+00	652
3119	0	0	0	0.026	0.006	0	0.004	0	0	*Crenarchaeota*	*Thaumarchaeota*	*Nitrososphaerales*	*Nitrososphaeraceae*	'*Ca*. Nitrososphaera'	*Nitrososphaera* sp. JG1	93.9	6.00E-160	572
1207	0.090	0.023	0.054	0.062	0.094	0.062	0.149	0.017	0.049	*Crenarchaeota*	*Thaumarchaeota*	*Nitrososphaerales*	*Nitrososphaeraceae*	'*Ca*. Nitrososphaera'	*Nitrososphaera* sp. JG1	96.3	2.00E-172	614
Total	0.090	0.057	0.072	0.156	0.161	0.124	0.180	0.069	0.154									
**AOB/AOA**	0.29	2.20	0.92	5.10	1.59	0.71	0.41	5.94	0.07									

[a]N.A., not assigned by QIIME program
