# Supplementary material for: Microbial Community Structure of Relict Niter-Beds Previously Used for Saltpeter Production
Source: PLoS One. 2014 Aug 11;9(8):e104752. doi: 10.1371/journal.pone.0104752 (PMC4128746; doi:10.1371/journal.pone.0104752)
Supplement: Table S6 — Phylogenetic identification of OTUs derived from bacterial and archaeal amoA gene sequences. (PDF) [file pone.0104752.s011.pdf]

Table S6. Phylogenetic identification of OTUs derived from bacterial and archaeal *amoA* gene sequences

	Relative abundance (%)											
OTU ID	OVA1	OVA2	OVA3	OVA4	OVA5	OVB2	OVB3	OVC1	OVC2	Closest strain	Identity (%)	Accession No
Bacterial *amoA*												
NB_Bamo_01			98.84	96.75	95.27	91.10	91.98	89.78	65.33	*Nitrosospira briensis* Nsp10	95.5	AY123821
NB_Bamo_02				0.01	0.02	0.20	0.71	5.63	31.79	*Nitrosomonas nitrosa*	87.2	AF272404
NB_Bamo_03			1.05	3.15	3.67	8.55	7.06	4.59	2.77	*Nitrosospira tenuis* Nv-12	96.2	U76552
NB_Bamo_04				0.03	0.98				0.08	*Nitrosospira multiformis* C-71	77.4	X90822
NB_Bamo_05			0.04	0.06	0.04	0.07	0.03		0.04	*Nitrosospira tenuis* Nv-1	87.0	AY123824
NB_Bamo_06						0.07	0.13			*Nitrosomonas europaea* ATCC 19178	85.2	JN099309
NB_Bamo_07			0.04				0.01			*Nitrosomonas europaea* ATCC 19178	94.9	JN099309
NB_Bamo_08							0.04			*Nitrosomonas oligotropha*	95.1	AF272406
NB_Bamo_09			0.03		0.01					*Nitrosospira* sp. NpAV	92.8	U92432
NB_Bamo_10						0.02				*Nitrosospira briensis* Nsp10	88.7	AY123821
NB_Bamo_11							0.01			*Nitrosomonas marina*	98.9	AF272405
NB_Bamo_12							0.01			*Nitrosomonas oligotropha*	84.1	AF272406
NB_Bamo_13			0.01							*Nitrosospira* sp. Nsp57	97.5	AY123835
Archaeal *amoA*												
NB_Aamo_01	2.08	10.27	69.76	69.91	71.96	57.90	62.71	79.18	80.23	'*Candidatus* Nitrososphaera gargensis' Ga9.2	90.6	CP002408
NB_Aamo_02	97.26	85.73	23.79	9.72	24.96	41.70	34.99	11.72	17.26	'*Candidatus* Nitrososphaera viennensis' EN76	77.8	FR773159
NB_Aamo_03	0.42	1.30	6.25	19.04	2.75	0.34	2.06	6.00	1.09	'*Candidatus* Nitrososphaera gargensis' Ga9.2	78.6	CP002408
NB_Aamo_04	0.19	2.49	0.15	1.04	0.16			1.55	0.38	'*Candidatus* Nitrososphaera viennensis' EN76	90.9	FR773159
NB_Aamo_05		0.03					0.05	0.79	0.12	'*Candidatus* Nitrososphaera gargensis' Ga9.2	78.4	CP002408
NB_Aamo_06				0.04				0.42	0.49	'*Candidatus* Nitrososphaera gargensis' Ga9.2	85.0	CP002408
NB_Aamo_07	0.02	0.06			0.05	0.07	0.08	0.14	0.08	'*Candidatus* Nitrososphaera gargensis' Ga9.2	78.2	CP002408
NB_Aamo_08				0.04	0.03		0.03		0.26	'*Candidatus* Nitrosopumilus' sp. AR2	82.4	CP003843
NB_Aamo_09			0.03	0.11	0.05			0.08	0.02	'*Candidatus* Nitrosopumilus koreensis' AR1	91.1	CP003842
NB_Aamo_10		0.03					0.05	0.08	0.02	'*Candidatus* Nitrososphaera gargensis' Ga9.2	80.6	CP002408
NB_Aamo_11			0.03	0.07	0.03					'*Candidatus* Nitrosopumilus koreensis' AR1	82.0	CP003842
NB_Aamo_12	0.02	0.06							0.02	'*Candidatus* Nitrososphaera viennensis' EN76	77.7	FR773159
NB_Aamo_13							0.03		0.02	'*Candidatus* Nitrososphaera gargensis' Ga9.2	78.4	CP002408
NB_Aamo_14				0.04						'*Candidatus* Nitrososphaera gargensis' Ga9.2	80.7	CP002408
NB_Aamo_15		0.03								'*Candidatus* Nitrososphaera gargensis' Ga9.2	85.4	CP002408
NB_Aamo_16								0.03		'*Candidatus* Nitrosopumilus koreensis' AR1	84.9	CP003842
